# Copper‐Metallized Porous *N*‐Heterocyclic Carbene Ligand Polymer‐Catalyzed Regio‐ and Stereoselective 1,2‐Carboboration of Alkynes

**DOI:** 10.1002/advs.202308238

**Published:** 2023-12-08

**Authors:** Jun‐Song Jia, Jin‐Rong Luo, Wen‐Hao Li, Fei‐Hu Cui, Ying‐Ming Pan, Hai‐Tao Tang

**Affiliations:** ^1^ State Key Laboratory for Chemistry and Molecular Engineering of Medicinal Resources School of Chemistry and Pharmaceutical Sciences Guangxi Normal University Guilin 541004 P. R. China; ^2^ Department of Chemistry Tsinghua University Beijing 100084 P. R. China

**Keywords:** 1,2‐carboboration, catalyst design, nano‐catalysis, NHC catalysis, stereoselectivity and regioselectivity

## Abstract

Alkenylboronates are highly versatile building blocks and valuable reagents in the synthesis of complex molecules. Compared with that of monosubstituted alkenylboronates, the synthesis of multisubstituted alkenylboronates is challenging. The copper‐catalyzed carboboration of alkynes is an operationally simple and straightforward method for synthesizing bis/trisubstituted alkenylboronates. In this work, a series of copper‐metallized *N*‐Heterocyclic Carbene (NHC) ligand porous polymer catalysts are designed and synthesized in accordance with the mechanism of carboboration. By using CuCl@POL‐NHC‐Ph as the optimal nanocatalyst, this study realizes the β‐regio‐ and stereoselective (*syn*‐addition) 1,2‐carboboration of alkynes (regioselectivity up to >99:1) with satisfactory yields and a wide range of substrates. This work not only overcomes the selectivity of carboboration but also provides a new strategy for the design of nanocatalysts and their application in organic synthesis.

## Graphical abstract

Bis/trisubstituted alkenylboronates are one of the most important motifs in organic synthesis. Herein, the synthesized series of copper metallized porous carbene ligand polymers are reported. Using CuCl@POL‐NHC‐Ph as the optimal nanocatalyst, the highly selective β‐regio‐ and *syn*‐stereoselective 1,2‐carboboration of alkynes are achieved to obtain bis/trisubstituted alkenylboronates.

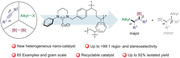

Alkenylboronates play an important role in organic synthesis because they are prevalent in organic chemistry as partners for C─C bond formation reactions and are especially widely used in Suzuki–Miyaura cross‐coupling.^[^ ^1^ ^]^ In addition, alkenylboronates exhibit important biological properties.^[^ ^2^ ^]^ Monosubstituted alkenylboronates can be successfully obtained in one step through the hydroboration of alkynes.^[^ ^3^ ^]^ However, the direct synthesis of bis/trisubstituted alkenylboronates through the multicomponent boration of alkynes in one step has been a challenge.^[^ ^4^ ^]^ The addition of another component not only reduces the yield of the reaction but also affects the selectivity of the reaction and faces the side reaction of direct alkyne hydroboration.

The copper‐catalyzed β‐regioselective 1,2‐carboboration of alkynes has been developed over the last decade. For example, the addition of phosphorus ligands can promote this reaction and provide specific products with satisfactory results (**Scheme** **1**A).^[^ ^4a–f^ ^]^ However, some problems remain to be solved. These problems include limited alkyl reagents, alkyne substrates, and regioselectivity or low yield. Although some reports have been published on the reaction with five‐membered NHC ligands, their results are still unideal.^[^ ^4g–i^ ^]^ The Hoveyda research group systematically investigated the NHC‐Cu‐catalyzed hydroboration of terminal alkynes in 2011.^[^ ^5^ ^]^ By using the Dewar–Chatt–Duncanson model, they found that the strongly donating NHCs make the π* back‐donation of copper to C≡C stronger than the π‐donation of C≡C to copper, facilitating the acquisition of β‐regioselective products. Ring‐expanded *N*‐heterocyclic carbene

**Scheme 1:**
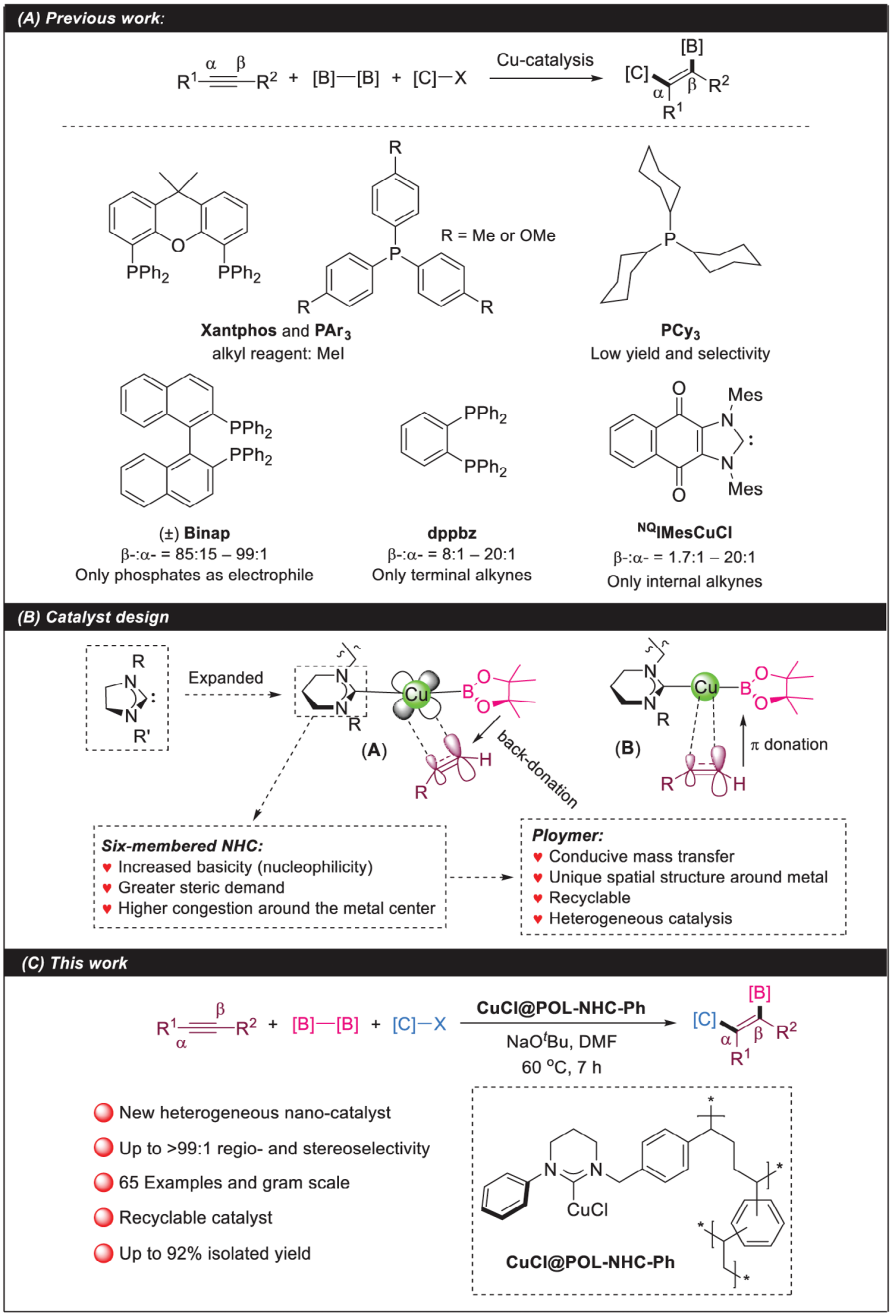
Status of research on alkyne 1,2‐carboboration reactions.

(RE‐NHC) complexes, such as copper(I) halides and copper(I) alkoxides, have been established as having enhanced catalytic activity relative to their five‐membered ring counterparts.^[^ ^6^ ^]^ In 2021, the Liptrot group discovered that (6‐Dipp)‐Cu‐Bpin has good stability and wide reactivity.^[^ ^7^ ^]^ Thus, we assume that RE‐NHCs may promote the selective 1,2‐carboboration of alkynes because they have the properties of increased basicity (nucleophilicity), great steric demand, and high congestion around their metal center (large N–C_NHC_–N angle) (Scheme 1B).^[^ ^8^ ^]^

Nanocatalysts have been widely used in many fields, such as the petroleum and chemical industries, energy, coatings, biology, and environmental protection, because of their high catalytic activity.^[^ ^9^ ^]^ However, chemists tend to focus on their catalytic activity and ignore their selective control of organic reactions. They also show great potential in the control of reaction selectivity, especially when their nanometal supports act as ligands.^[^ ^10^ ^]^ Porous organic ligands (POLs) are a class of highly cross‐linked amorphous polymers, which are mainly obtained by polymerizing organic ligands after modification. POLs with various functionalities can be synthesized successfully with the bottom‐up approach.^[^ ^11^ ^]^ Compared with other supports, POLs have the advantages of high surface area, large pore volume, hierarchical porosity, superior stability, high efficiency, excellent selectivity, and reduced the HOMO‐LUMO gap, which have attracted the attention of many chemists. More importantly, they are not only used as solid carriers, but also as ligands for various metal catalysts.^[^ ^12^ ^]^ As a recyclable catalyst, M/POLs have achieved many organic reactions, such as asymmetric conjugate addition and selective conversion of olefins/alkynes/allenes.^[^ ^13^ ^]^ We believe that the combination of nanocatalysis and POLs can be used to solve the selectivity problem that cannot be solved under homogeneous conditions. Therefore, we attempted to synthesize a series of copper‐metallized six‐membered NHC ligand polymer catalysts and the carboboration of alkynes. Our results showed that the NHC catalysts achieved the 1,2‐carboboration of alkynes with high regioselectivity (β‐:α‐ up to >99:1) and stereoselectivity (*syn*‐addition) in the synthesis of alkenylboronates and that the catalytic system has high efficiency (up to 92% isolated yield) and wide substrate scope (65 examples) for this reaction (Scheme 1C).

We started the development of the 1,2‐carboboration reaction by using phenylacetylene **1a**, 1‐iodobutane **2a,** and B_2_pin_2_ **3a** as model substrates under the standard conditions shown in **Table** **1**. At the outset, we screened three expanded six‐membered CuCl@POL‐NHC complex heterogeneous catalysts (entries 1–3, Table 1). In an argon atmosphere, CuCl@POL‐NHC‐Ph provided the trisubstituted alkenylboronate **4a** with 88% isolated yield and excellent regioselectivity (β‐[B]:α‐[B] > 99:1). The unsaturated‐backbone five‐membered CuCl@POL‐NHC‐BI furnished the product with 70% yield with 94:6 regioselectivity (entry 4, Table 1). Cu(OTf)_2_@POL‐NHC‐Ph provided 38% isolated yield and 96:4 regioselectivity (entry 5, Table 1). CuCl_2_@POL‐NHC‐Ph gave **4a** with 48% isolated yield and >99:1 selectivity (entry 6, Table 1). CuBr@POL‐NHC had unsatisfactory yields in 1,2‐carboboration (entry 7, Table 1). We only obtained trace amounts of products by catalyzing this reaction by using the homogeneous ligand NHC‐Ph combined with CuCl (entry 8, Table 1). The five‐membered carbene polymer catalyst CuCl@POL‐NHC‐PI with the same substituent as CuCl@POL‐NHC‐Ph catalyzed the model reaction to obtain product **4a** with 75% yield and >95:5 selectivity (entry 9, Table 1). POL‐NHC‐Ph without [Cu] loading could not catalyze the reaction (entry 10, Table 1), the results showed that the uncoordinated carbene in POLs had no effect on the reaction. We also screened the type and amount of the base, solvent, the temperature of the reaction, and time (see Supporting Information).

**Table 1:** Optimization of reaction conditions.

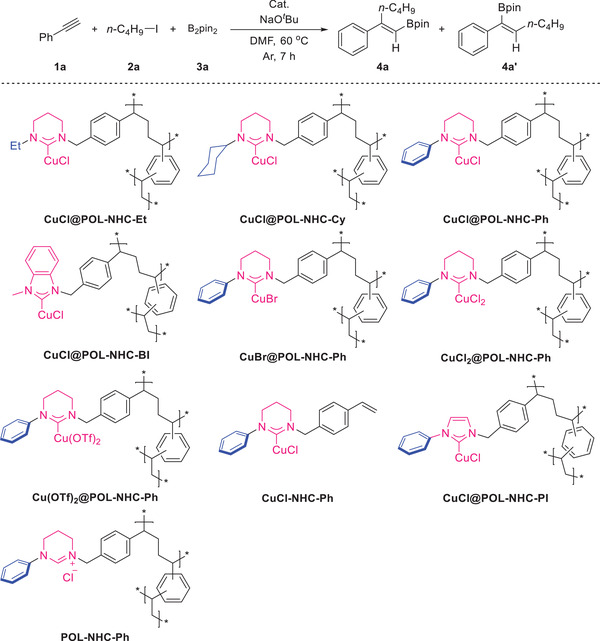			
Entry^a)^	Catalyst	Yield (%)^b)^	Ratio of 4a:4a’^c)^
1	CuCl@POL‐NHC‐Et	74	93:7
2	CuCl@POL‐NHC‐Cy	80	99:1
3	CuCl@POL‐NHC‐Ph	88	>99:1
4	CuCl@POL‐NHC‐BI	70	94:6
5	Cu(OTf)_2_@POL‐NHC‐Ph	38	96:4
6	CuCl_2_@POL‐NHC‐Ph	48	>99:1
7	CuBr@POL‐NHC‐Ph	trace	–
8^d)^	CuCl‐NHC‐Ph	trace	–
9	CuCl@POL‐NHC‐PI	75	>95:5
10	POL‐NHC‐Ph	0	–

^a)^ Reaction conditions: **1a** (0.5 mmol), **2a** (2 equiv), **3a** (1.5 equiv), NaO*^t^* Bu (1.5 equiv), [Cu]@POL‐NHC (20 mg), DMF (0.25 M), 60°C, 7 h;

^b)^ Isolated yield;

^c)^ Determined by using ^1^H NMR spectroscopy;

^d)^ 4 mol% CuCl‐NHC‐Ph. The conversion rate of NHC in [Cu]@POL‐NHC is shown in Table S4 (Supporting Information).

We studied the scope of various alkynes that underwent β‐selective 1,2‐carboboration catalyzed by the heterogeneous catalyst CuCl@POL‐NHC‐Ph under the optimized conditions. The results are summarized in **Scheme** **2**. *Para*‐substituted phenylacetylenes, such as methyl (**1b**), methoxy (**1c**), *tert*‐butyl (**1d**), fluorine (**1e**), chlorine (**1f**), bromine (**1** **g**), and trifluoromethyl (**1** **h**), could react well with good yield and excellent regioselectivities (**4b**–**4 h**). Similarly, when the *meta* position of phenylacetylene was substituted, the reaction also proceeded smoothly to provide the corresponding products with moderate to good yields and high regioselectivities (**4i**–**4k**). We found that the products obtained with the *ortho*‐substitution of phenylacetylene had high regioselectivity (97:3–99:1) but slightly reduced yield, which may be due to steric hindrance (**4l**–**4n**). 4‐Phenylphenylacetylene afforded the target product with 82% yield and 98:2 selectivity (**4o**). Condensed and heterocyclic aromatic hydrocarbons, such as 2‐naphthylacetylene (**1p**), 1‐naphthylacetylene (**1q**), 2‐thiophene (**1r**), and 3‐thiophene (**1s)**, afforded the desired products with good yields (80–86%) and excellent selectivity (96:4–99:1) (**4p**–**4s**). Interestingly, after we reduced B_2_pin_2_ to 1 equivalent, the 1,2‐carboboration of 1,4‐diethynylbenzene provided a product that retained carbon‒carbon triple bonds (**4t**). The coordination structure of ferrocene acetylene was unaffected after the reaction under standard conditions (**4u**). 1‐Ethynylcyclohexene generated the corresponding product with high yield and excellent selectivity (**4v**). We also examined the compatibility of internal alkynes with this catalytic system. Asymmetric internal alkynes afforded a series of trisubstituted alkenylboronates with good yields and β‐selectivities (**4w**–**4z**). Symmetrical alkynes exhibited excellent stereoselectivity (*syn* addition) (**4aa**–**4ae**). Remarkably, the natural product menthol‐modified alkyne substrate afforded alkenylboronates with 66% yield and 99:1 selectivity under the optimal conditions (**4af**). The reaction of the chain‐conjugated 3‐en‐1‐yne with B_2_pin_2_ and 1‐iodobutane can smoothly generate conjugated diene compounds with 80–91% yields and excellent regioselectivity (**4ag**–**4ai**). Finally, we explored the substrate range of alkyl alkynes. Alkenylboronates **4aj**–**4al** were synthesized from oxygen‐ and nitrogen‐substituted propargyl with 75%–82% yield and 88:12–99:1 regioselectivity. Although the desired products (**4am**–**4ao**) were afforded with good yields with 4‐phenyl‐1‐butyne, 1‐decyne, and 5‐chloro‐1‐pentyne, their selectivity decreased (86:14–91:9). The internal alkyl alkyne 2‐octyne provided **4ap** and **4ap'** with a selectivity of 76:24 and yield of 81%.

**Scheme 2:**
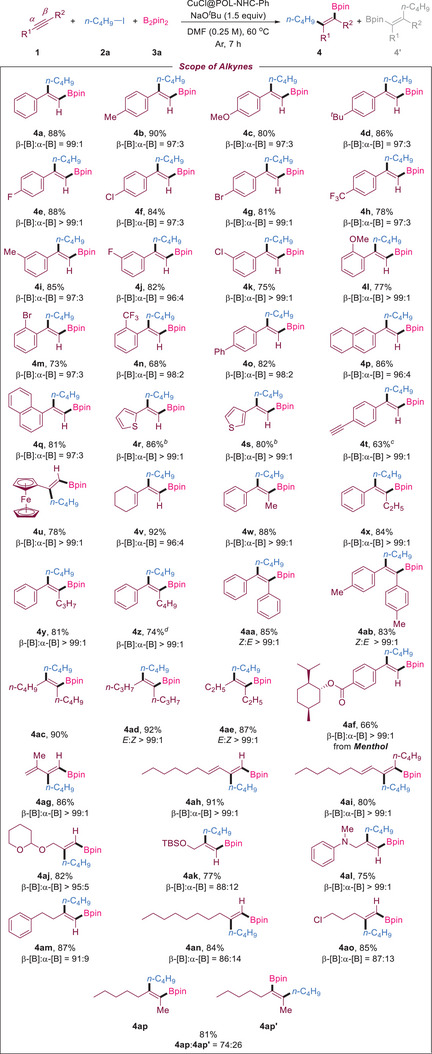
Scope of alkynes for 1,2‐carboboration with 1‐iodobutane and B_2_pin_2_. [a] Reaction conditions: **1** (0.5 mmol), **2a** (2 equiv), **3a** (1.5 equiv), NaO*^t^* Bu (1.5 equiv), CuCl@POL‐NHC‐Ph (20 mg, 4.67 wt.% Cu), DMF (0.25 M), 60°C, 7 h. Isolated yield. The value of β‐[B]:α‐[B] was determined by ^1^H NMR. [b] Room temperature. [c] 1 Equiv B_2_pin_2_. [d] The reaction time was 12 h.

Next, the scope of electrophiles and diborates was investigated (**Scheme** **3**). Unactivated alkyl iodides, such as iodomethane (**2b**), iodoethane (**2c**), 2‐iodopropane (**2d**), and 1‐iodo‐2‐methylpropane (**2e**), afforded the corresponding disubstituted alkenylboronates with moderate to good yields and high regioselectivities (up to 99:1). The reaction using (iodomethyl)cyclopropane alone produced the desired borylalkylation product and no ring‐opening product (**2f**), indicating that the reaction proceeded through a nonradical process.^[^ ^4^, ^14^ ^]^ Long‐chain iodoalkanes (**2g**,**h**) can also smoothly provide alkenylboronates with good yields and selectivities of 96:4 and 99:1. The 2‐phenyl‐substituted ethyl iodide (**2i**) can also smoothly undergo the reaction to furnish the corresponding product. Alkyl iodide reagents with other substituent groups, such as chlorine (**2j**), ‐CF_3_ (**2k**), ester (**2l**), and silyl ether (**2m**), all afforded alkenylboronates with moderate yields (50%–73%) and good selectivity (96:4–99:1). In addition to iodine substitution reagents, 1‐bromobutane (**2n**), benzyl bromide (**2o**), benzyl chloride (**2p**), and phosphate esters (**2q**) can be compatible with the catalytic system (63%–81% yield, 93:7–99:1 regioselectivity). We also explored the scope and limitations of diborate **3**. Highly sterically hindered six‐membered cyclic boronates, such as B_2_nep_2_ (**3b**), B_2_mpd_2_ (**3c**), and bis(2,4‐dimethylpentane‐2,4‐glycolato)diboron (**3d**), gave the product in good yield and 95:5–99:1 selectivity under standard conditions. B_2_pai_2_ (**3e**) can also react smoothly to generate the corresponding alkenylboronate products.

**Scheme 3:**
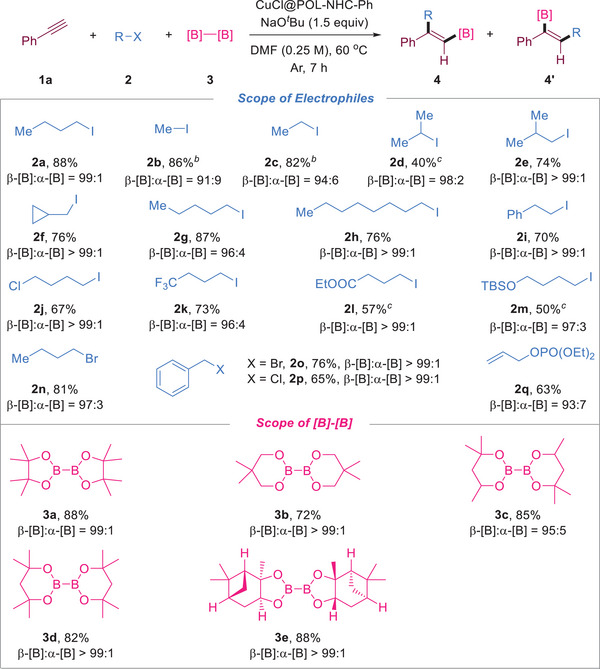
Scope of the electrophile and [B]–[B] reaction with phenylacetylene. [a] Reaction conditions: **1a** (0.5 mmol), **2** (2 equiv), **3** (1.5 equiv), NaO*^t^* Bu (1.5 equiv), CuCl@POL‐NHC‐Ph (20 mg, 4.67 wt.% Cu), DMF (0.25 M), 60 °C, 7 h. Isolated yield. The value of β‐[B]:α‐[B] was determined by ^1^H NMR. [b] Room temperature. [c] The reaction time was 12 h.

We demonstrated the synthetic utility of the disubstituted alkenylboronates synthesized through the above method (**Scheme** **4**A). First, we performed a gram‐scale synthesis of **4a** under standard conditions. After we scaled up the amount of alkynes by a factor of 20, **4a** was still obtained with 85% (2.43 g) isolated yield and >97:3 regioselectivity. The Suzuki–Miyaura coupling of **4a** with 4‐iodoanisole afforded **5** in 94% yield. Vinyl azide **6** was prepared through a copper‐mediated reaction with sodium azide with 62% yield. Furthermore, compound **7**, a valuable enol ether for various organic transformations, was obtained readily with 80% yield. Conjugated dienyl ester **8** could be prepared through the oxidative Heck coupling of **4a** and *tert*‐butyl acrylate (*EE*:*EZ* = 95:5). Vinyl halides (**9** and **10**), which are important coupling partners in various transition‐metal‐catalyzed syntheses, were obtained with good yields of 81% and 85%. Additionally, we synthesized 2‐ethynyl‐5‐propylthiophene **11**, which reacted with methyl iodide and B_2_pin_2_ at room temperature to generate alkenylboronate **12**. The hydrolysis of the ester group was completed after the Suzuki–Miyaura coupling of **12** and methyl 4‐iodobenzoate to obtain product **13**. Product **13** and the drug molecule **namirotene** (**CBS‐211A**) are isomers (Scheme 4B). Alkyne **14** reacted with ethyl iodide and B_2_pin_2_ to generate trisubstituted alkenylboronate **15** (74% yield, >99:1). The nonsteroidal estrogen drug diethylstilbestrol was acquired via the Suzuki coupling of **15** with 4‐iodoanisole followed by deprotection (Scheme 4B).

**Scheme 4:**
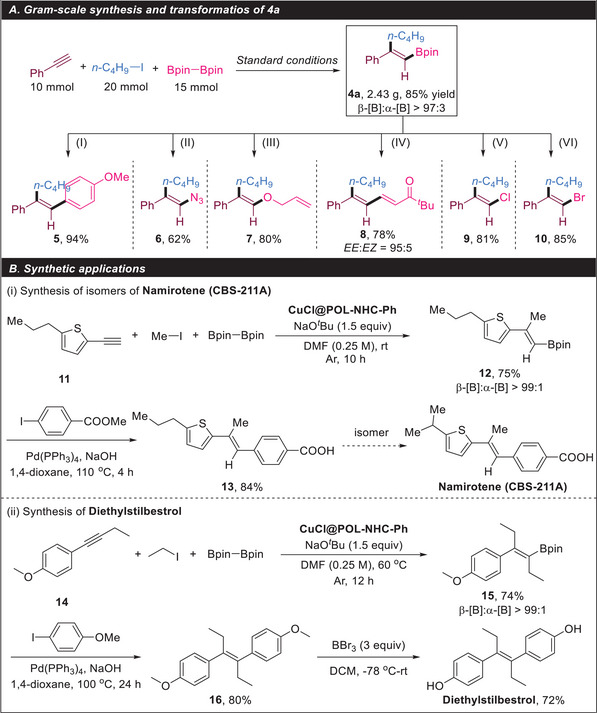
Practicality of alkyne 1,2‐carboboration. A) Gram‐scale synthesis and transformations of **4a**: (I) 4‐iodoanisole, Pd(PPh_3_)_4_, NaOH, 1,4‐dioxane, 100 °C; (II) NaN_3_, CuSO_4_, MeOH, 50 °C, 12 h; (III) allyl alcohol, Cu(OAc)_2_, NEt_3_, rt, 16 h; (IV) *tert*‐butyl acrylate, Pd(OAc)_2_, 1,10‐phenanthroline, O_2_, DMA, 80°C, 12 h; (V) CuCl_2_, THF/MeOH/H_2_O, 100 °C, 24 h; (VI) CuBr_2_, EtOH/H_2_O, 100 °C, 24 h. (B) Synthetic applications.

As shown in **Figure** **1** (left), we performed a leaching experiment to investigate whether the reaction was homogeneous or heterogeneous.^[^ ^15^ ^]^ When the carboboration of phenylacetylene with 1‐iodobutane and B_2_pin_2_ was performed for 2 h, the GC‐MS yield was 39%. When the reaction was continued for 7 h, the final GC‐MS yield was 91% (black line). The reaction was conducted for 2 h, the catalyst was removed through filtration, and the solution was further reacted under the same conditions for 7 h. The final GC‐MS yield was 49% (red line). In addition, by washing and post‐treating the used catalyst, we recycled the nanocatalyst for five cycles (Figure 1 (right)). Leaching and cycle tests demonstrated that the reaction is likely a heterogeneously catalyzed process.

**Figure 1:**
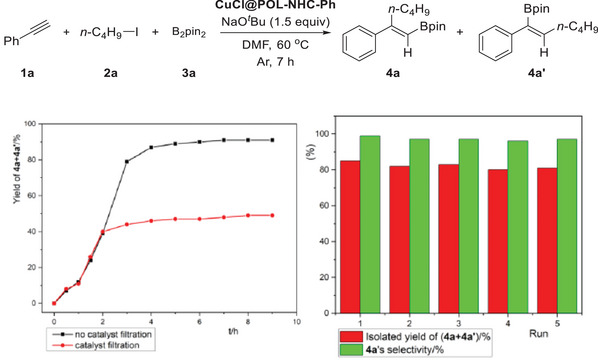
Results of leaching (left) and cycle (right) experiments. Reaction conditions: **1a** (0.5 mmol), **2a** (2 equiv), **3a** (1.5 equiv), NaO*^t^* Bu (1.5 equiv), recycled CuCl@POL‐NHC‐Ph (20 mg), DMF (0.25 M), 60 °C, 7 h. Isolated yield. The filtering time is ≈1 min.

The prepared POL‐NHC‐Ph and CuCl@POL‐NHC‐Ph were characterized to study their structure–reactivity relationship (**Figure** **2**). Scanning electron microscopy (SEM) revealed that POL‐NHC‐Ph and CuCl@POL‐NHC‐Ph were porous (Figure 2a). The results of transmission electron microscopy (TEM), energy‐dispersive X‐ray spectroscopy (EDS), and elemental mapping showed that CuCl was loaded on POL‐NHC‐Ph in the form of nanoparticles (Figure 2b–d). N_2_ adsorption–desorption analysis revealed that CuCl@POL‐NHC‐Ph had a Brunauer–Emmett–Teller (BET) surface area of 151.90 m^2^/g and a corresponding total pore volume of 0.31 cm^3^ g^−1^ (Figure 2e,f). Adsorption experiments showed that the catalyst had adsorption effect on phenylacetylene, iodobutane and B_2_pin_2_ (Figure S9, Supporting Information). We performed X‐ray photoelectron spectroscopy (XPS) on our catalyst to check the valence of the copper species. In contrast to the CuCl 2p photoemission peak (Cu 2p_3/2_ and Cu 2p_1/2_ binding energies of 932.28 and 952.28 eV, respectively), the XPS peak of CuCl@POL‐NHC‐Ph had a positive shift of 1.4 eV (Cu 2p_3/2_ and Cu 2p_1/2_ binding energies of 933.68 and 953.68 eV, respectively),^[^ ^16^ ^]^ indicating coordination between CuCl and POL‐NHC‐Ph (Figure 2g). Carbene is a severely electron‐deficient structure. When the carbene carbon coordinates with CuCl, the lone pair electrons of the 3d orbital of copper may partially feed back into the empty p orbital of the carbene carbon. Therefore, the electron loss of copper occurs, and the XPS spectrum shows a positive shift. The TG curves of POL‐NHC‐Ph and CuCl@POL‐NHC‐Ph demonstrated that the polymer remained intact at temperatures of up to 200 °C (Figure 2h). We also performed the same characterization on metal‐free POL‐NHC‐Ph and used CuCl@POL‐NHC‐Ph (see Figures S1–S7, Supporting Information). In addition, to obtain information regarding the oxidation state of used copper nanoparticles, the catalyst was analyzed by using XPS, which provided evidence for the presence of Cu(I) and Cu(II) with an estimated ratio of 1.14:1. Inductively coupled plasma‐mass spectrometry revealed that the copper loading of CuCl@POL‐NHC‐Ph was 4.67 wt.% (Figure S8, Supporting Information). The results of solid‐state NMR and infrared spectroscopy of CuCl@POL‐NHC‐Ph showed that the structure of carbene in the polymer remained intact (Figures S10 and S11, Supporting Information). The characterization results revealed no significant change in the catalyst after use, revealing the superior stability of the catalyst.

**Figure 2:**
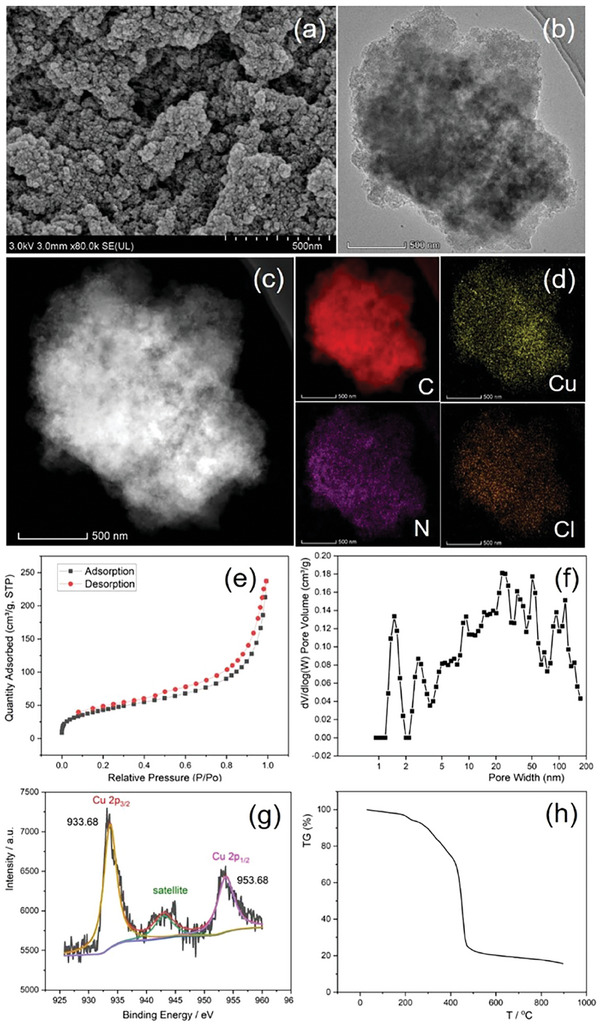
Characterization results. a) Scanning electron microscopy image of CuCl@POL‐NHC‐Ph. b) TEM image of CuCl@POL‐NHC‐Ph. c) HAADF image of CuCl@POL‐NHC‐Ph. d) Energy‐dispersive X‐ray spectroscopy elemental mapping analysis of CuCl@POL‐NHC‐Ph. e) N_2_ adsorption–desorption isotherms, f) pore size distribution curves, g) Cu 2p XPS spectra of fresh CuCl@POL‐NHC‐Ph, and h) thermogravimetric analysis of CuCl@POL‐NHC‐Ph.

We conducted DFT calculations to explain the differences among the four NHC catalysts. We analyzed the natural orbitals of the polymer subunits **C1**, **C2**, **C3**, and **C4** (**Figure** **3**). We found that the gap between the highest occupied molecular orbital (HOMO) and lowest unoccupied molecular orbital (LUMO) of **C3** was considerably smaller than that of **C1**, **C2**, and **C4** (Figure 3A). A small gap means that the catalyst facilitates electron transition and that the catalyst has high activity. It also suggests that the catalyst is highly reactive when interacting with other reactants.^[^ ^10e^ ^]^ Surprisingly, the HOMO–LUMO gap of the tetramer of **C3** decreased to 386.1 KJ mol^−1^, suggesting that polymerization may improve the activity of the catalyst (Figure 3B).

**Figure 3:**
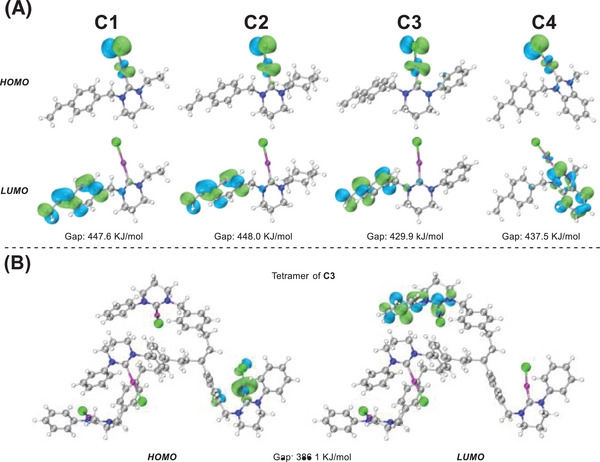
Results of DFT calculation.

On the basis of the selectivity results of our experiments and related reports on copper‐catalyzed alkyne carboboration reactions,^[^ ^4^, ^17^ ^]^ we proposed a possible reaction process (**Scheme** **5**). The experimental results of unactivated iodoalkane **2f** showed that the reaction mechanism was not a free radical process. The catalytically active species **B** was generated by catalyst **A** under the action of NaO*^t^* Bu. Subsequently, **B** reacted with biborate **3** to form intermediate **C**. The β‐*syn*‐addition of **C** and alkyne **1** coordinates provided the vinyl cuprate intermediate **E**. Finally, **E** was integrated with electrophile **2** to obtain the desired product **4** and regeneration catalyst **A**.

**Scheme 5:**
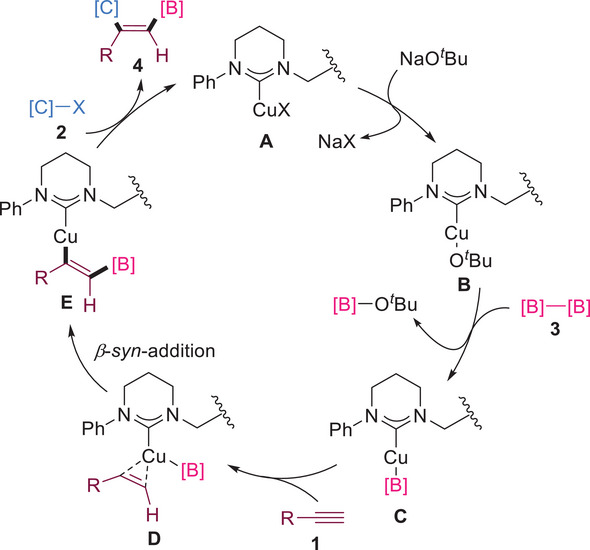
Possible catalytic cycles of copper‐catalyzed 1,2‐carboboration.

In summary, we developed a highly regio‐ and stereoselective method for the 1,2‐carboboration of alkynes with a novel recyclable and highly stable heterogeneous nanocatalyst, CuCl@POL‐NHC‐Ph. We characterized the prepared POL‐NHC‐Ph and CuCl@POL‐NHC‐Ph to study their structure–reactivity relationships. TEM images showed that copper NPs were highly discretely distributed in this porous polymer. This distribution pattern resulted in the high activity of this nanocatalyst. The reaction had broad substrate adaptability and good yields. Additionally, the results of DFT calculations showed that among the catalysts, the Cu‐NHC complex **C3** had the smallest HOMO–LUMO gap, indicating that **C3** may have high catalytic activity. We also proposed a possible mechanism for the CuCl@POL‐NHC‐Ph‐catalyzed β‐selective 1,2‐carboboration of alkynes.

## Conflict of Interest

The authors declare no conflict of interest.

## Supporting information

Supporting Information

## Data Availability

The data that support the findings of this study are available in the supplementary material of this article.
