# Supplementary material for: Copper‐Metallized Porous N‐Heterocyclic Carbene Ligand Polymer‐Catalyzed Regio‐ and Stereoselective 1,2‐Carboboration of Alkynes
Source: Adv Sci (Weinh). 2023 Dec 8;11(7):2308238. doi: 10.1002/advs.202308238 (PMC10870022; doi:10.1002/advs.202308238)
Supplement: Supplementary file 1 — Supporting Information [file ADVS-11-2308238-s001.pdf]

ADVANCED SCIENCE
Open Access

Supporting Information

for *Adv. Sci.*, DOI 10.1002/advs.202308238

Copper-Metallized Porous *N*-Heterocyclic Carbene Ligand Polymer-Catalyzed Regio- and Stereoselective 1,2-Carboboration of Alkynes

Jun-Song Jia, Jin-Rong Luo, Wen-Hao Li, Fei-Hu Cui, Ying-Ming Pan and Hai-Tao Tang**

Supporting Information
©Wiley-VCH 2021
69451 Weinheim, Germany

Copper-Metallized Porous *N*-Heterocyclic Carbene Ligand Polymer-Catalyzed Regio- and Stereoselective 1,2-Carboboration of Alkynes

Jun-Song Jia,[a] Jin-Rong Luo,[a] Wen-Hao Li,[b] Fei-Hu Cui,[a] Ying-Ming Pan*,[a] Hai-Tao-Tang*[a]

[a] J.-S. Jia, J.-R. Luo, F.-H. Cui, Prof. Dr. Y.-M. Pan, Prof. Dr. H.-T. Tang
State Key Laboratory for Chemistry and Molecular Engineering of Medicinal Resources, School of Chemistry and Pharmaceutical Sciences
Guangxi Normal University
Guilin, 541004, P.R. China
E-mail: panym@mailbox.gxnu.edu.cn; httang@gxnu.enu.cn

[b] W.-H. Li
Departmentof Chemistry, TsinghuaUniversity
Beijing, 100084, P.R. China
E-mail: liwh20@mails.tsinghua.edu.cn

Abstract: Alkenylboronates are highly versatile building blocks and valuable reagents in the synthesis of complex molecules. Compared with that of monosubstituted alkenylboronates, the synthesis of multisubstituted alkenylboronates is challenging. The copper-catalyzed carboboration of alkynes is an operationally simple and straightforward method for synthesizing bis/trisubstituted alkenylboronates. In this work, we designed and synthesized a series of copper-metallized NHC ligand porous polymer catalysts in accordance with the mechanism of carboboration. By using CuCl@POL-NHC-Ph as the optimal nanocatalyst, we realized the β-regio- and stereoselective (*syn*-addition) 1,2-carboboration of alkynes (regioselectivity up to >99:1) with satisfactory yields and a wide range of substrates. Our work not only overcomes the selectivity of carboboration but also provides a new strategy for the design of nanocatalysts and their application in organic synthesis.

Table of Contents

1. General information 3
2. Experimental Procedures 3
 2.1. Synthesis of [Cu]@POL-NHC 3
 2.2 Synthesis of 2-ethynyl-5-propylthiophene (**11**) 7
 2.3 Synthesis of (*E*)-dec-3-en-1-yne (1ah) and (*E*)-tetradec-7-en-5-yne (**1ai**) 8
 2.4 Synthesis of *N*-methyl-*N*-(prop-2-yn-1-yl)aniline (**1al**) 9
 2.5 Synthesis of 1-(But-1-yn-1-yl)-4-methoxybenzene (**14**) 9
3. Results and Discussion 9
 3.1 Optimization studies 9
 3.2 Transformations of the **4a** 11
 3.3 Synthesis of isomer **13** of **Namirotene** (**CBS-211A**) 13
 3.4 Synthesis of **Diethylstilbestrol** 14
 3.5 Characterization of catalyst 15

3.6 Copper percentage of polymer-supported catalyst 18
3.7 Estimate the amount of Cu(II) in the used Cu(I) nano-catalyst. 18
3.8 ^{1}H NMR adsorption experiments 19
3.9 Solid-state ^{13}C NMR test of POL-NHC-Ph 21
3.10 Infrared test of NHC-Ph and POL-NHC-Ph 21
3.11 Control experiment 22
3.12 Computational details 22
3.13 Characterization data of alkenylboronates 22
3.14 NMR Spectra 40
4. References 156
5. Author Contributions 156

1. General information

Reagents were purchased from commercial suppliers and used without further purification. THF, toluene, and hexane, ethyl ether were used after dried by molecular sieve. THF, toluene, and hexane, ethyl ether were used after dried by molecular sieve. Column chromatography on silica gel (300−400 mesh) was carried out using technical grade 60−90℃ petroleum ether (distillated prior to use) and analytical grade EtOAc (without further purification). ^{1}H NMR and ^{13}C NMR spectra were recorded on a 400 or 600 MHz spectrometer. Chemical shifts were reported in ppm. ^{1}H NMR spectra were referenced to $CDCl_3$ (7.26 ppm), and ^{13}C-NMR spectra were referenced to $CDCl_3$ (77.0 ppm). Peak multiplicities were designated by the following abbreviations: s, singlet; d, doublet; t, triplet; m, multiplet; brs, broad singlet and *J*, coupling constant in Hz. The Glove Box & Gas Purification System is produced by Mikrouna; HRMS spectra were recorded with Aglient 7250& JEOL-JMS-T100LP AccuTOF and Aglient 7250& JEOL-JMS-T100LP AccuTOF and Micromass QTOF2 Quadrupole/Time-of-Flight Tandem mass spectrometer using electron spray ionization. Scanning electron microscope (SEM) was performed on SU8010 (Hitachi, Japan); Thermogravimetric analysis (TGA) was carried out using a thermal analyzer (NETZSCH STA 449 F3), during which the sample was heated at the rate of 10 K min^{-1} from room temperature up to 1173 K under a nitrogen atmosphere; Transmission electron microscopy (TEM) images and energy dispersive X-ray spectroscopy (EDS) mapping were obtained from the Talos 200S transmission electron microscope (Thermo Fisher Scientific, USA). X-ray photoelectron spectroscopy (XPS) measurement was performed on an ESCALABMK II X-ray photoelectron spectrometer (Thermo Fisher Scientific, USA). The Brunauer–Emmett–Teller (BET) was obtained from the ASAP-2460 (Micromeritics, USA); The Cu content passed the Inductively Coupled Plasma Mass (ICP-MS) spectrometry of FLexar-NexION300X of PekinElmer in the United States.

2. Experimental Procedures

2.1. Synthesis of [Cu]@POL-NHC

2.1.1 Synthesis of Cu metallized porous organic polymers CuCl@POL-NHC-Et and CuCl@POL-NHC-Cy.

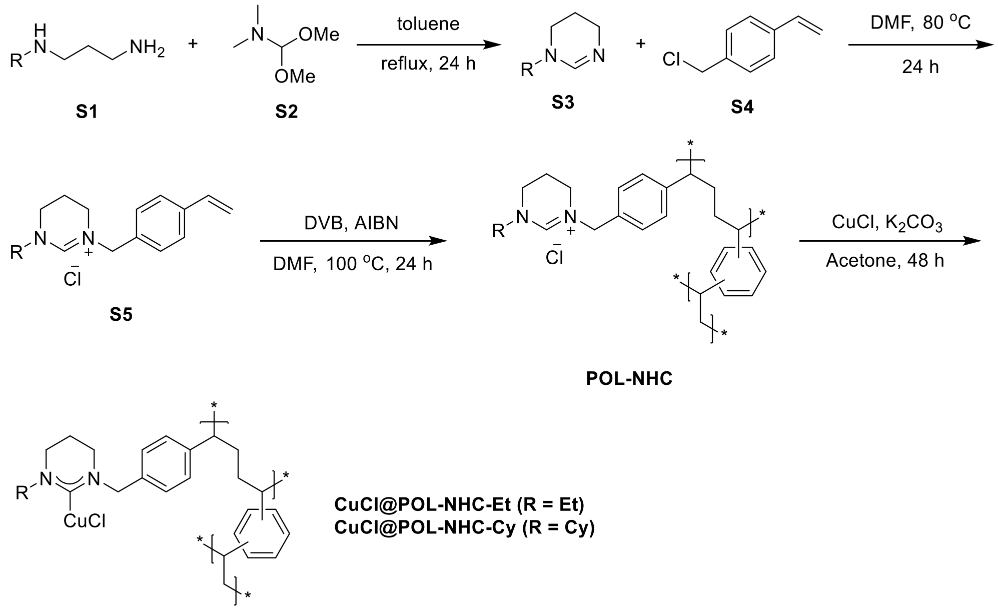

The **S5** was synthesized following similar literature reports.[1] *N*,*N*-Dimethylformamide dimethyl acetal **S2** (52.5 mmol, 1.05 equiv) was added to the mixture of **S1** (50 mmol, 1 equiv) in anhydrous toluene (50 mL, 1 M) at room temperature, and the mixture was stirred at 80 °C for 24 h. The mixture was evaporated in vacuo, and the residue was distilled under reduced pressure to give **S3** as a colorless liquid.

To a solution of **S3** (30 mmol, 1 equiv) in DMF (30 mL) was added slowly 1-(chloromethyl)-4-vinylbenzene **S4** (30.3 mmol, 1.01 equiv) at rt and the resulting mixture was stirred at 80 °C for 8 h. ***POL-NHC-Et:*** the residue was purified by column chromatography (MeOH:DCM = 15:1, R_f = 0.2) to obtain **S5** (R = Et) as a colourless oil liquid (5.8 g, 73%); ***POL-NHC-Cy:*** diethyl ether was added to obtain a white crystalline solid, which was filtered off. The solid was washed with diethyl ether (3 × 20 mL), and dried under vacuum to obtain white solid product **S5** (R = Cy) (7.75 g, 81%).

In a 50 mL Schlenk tube, divinylbenzene (DVB) (2.6 g, 20 mmol, 2 equiv) and **S5** (10 mmol, 1 equiv) were added to a solution containing 82 mg of 2,2'-azobis(isobutyronitrile) (AIBN) (0.5 mmol, 0.05 equiv) and 20 mL DMF (0.5 M). The mixture was hydrothermally treated at 100 °C for 24 h. When the reaction was completed, the solution was filtered and washed with MeOH and EA 3 times. Then the white solid POL-NHC-Et/Cy was obtained.

The CuCl@POL-NHC-Et and CuCl@POL-NHC-Cy was synthesized following similar literature reports.[2]A vial was charged with POL-NHC-Et/Cy (200 mg), CuCl (20 mg, 1 equiv) and K_2CO_3 (2.0 equiv). The mixture was dissolved in acetone (10 mL) and stirred at 60 °C for 48 h. The solution was then filtered through silica which was washed with DCM and EA (3 × 5 mL). The filtered solid was dried under vacuum to obtain catalyst CuCl@POL-NHC-Et/Cy.

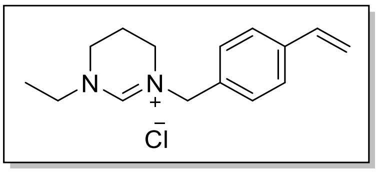

3-Ethyl-1-(4-vinylbenzyl)-3,4,5,6-tetrahydropyrimidin-1-ium chloride (**C1**)
^{1}H NMR (600 MHz, DMSO-*d*$_6$): δ 8.66 (s, 1H), 7.54−7.50 (m, 2H), 7.39−7.36 (m, 2H), 6.75 (dd, J_1 = 10.9 Hz, J_2 = 17.6 Hz, 1H), 5.87 (dd, J_1 = 0.4 Hz, J_2 = 17.6 Hz, 1H), 5.30 (d, *J* = 11.4 Hz, 1H), 4.61 (s, 2H), 3.49 (q, *J* = 7.1 Hz, 2H), 3.34 (t, *J* = 5.9 Hz, 2H), 3.17 (t, *J* = 5.6 Hz, 2H), 1.92−1.89 (m, 2H), 1.24 (t, *J* = 7.2 Hz, 3H);
^{13}C NMR (151 MHz, DMSO-*d*$_6$): δ 145.0, 130.6, 128.4, 125.5, 120.8, 118.9, 106.1, 50.1, 42.4, 34.5, 34.4, 20.8, 4.6;
HRMS-ESI: *m/z* $[M]^+$ calcd for $C_{15}H_{21}N_2$: 229.1699, found 229.1707.

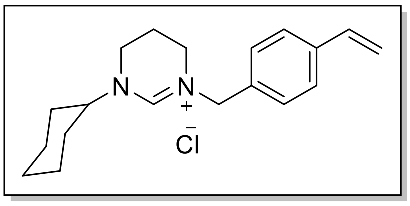

3-Cyclohexyl-1-(4-vinylbenzyl)-3,4,5,6-tetrahydropyrimidin-1-ium chloride (**C2**)
Melting point: 176.0−177.0 °C
^{1}H NMR (600 MHz, DMSO-*d*$_6$): δ 8.75 (s, 1H), 7.56−7.48 (m, 2H), 7.43−7.35 (m, 2H), 6.75 (dd, J_1 = 16.4 Hz, J_2 = 26.5 Hz, 1H), 5.87 (dd, J_1 = 1.1 Hz, J_2 = 26.5 Hz, 1H), 5.30 (dd, J_1 = 1.0 Hz, J_2 = 16.5 Hz, 1H), 4.66 (s, 2H), 3.51−3.41 (m, 1H), 3.37 (t, *J* = 8.4 Hz, 2H), 3.18 (t, *J* = 8.6 Hz, 2H), 1.92−1.78 (m, 6H), 1.65−1.52 (m, 3H), 1.35−1.24 (m, 2H), 1.18−1.09 (m, 1H);
^{13}C NMR (151 MHz, DMSO-*d*$_6$): δ 152.6, 127.7, 136.6, 134.5, 129.1, 127.0, 115.4, 63.8, 57.5, 42.9, 30.5, 25.3, 25.0, 19.1;
HRMS-ESI: *m/z* $[M]^+$ calcd for $C_{19}H_{27}N_2$: 283.2169, found 283.2173.

2.1.2 Synthesis of Synthesis of Cu metallized porous organic polymers [Cu]@POL-NHC-Ph.

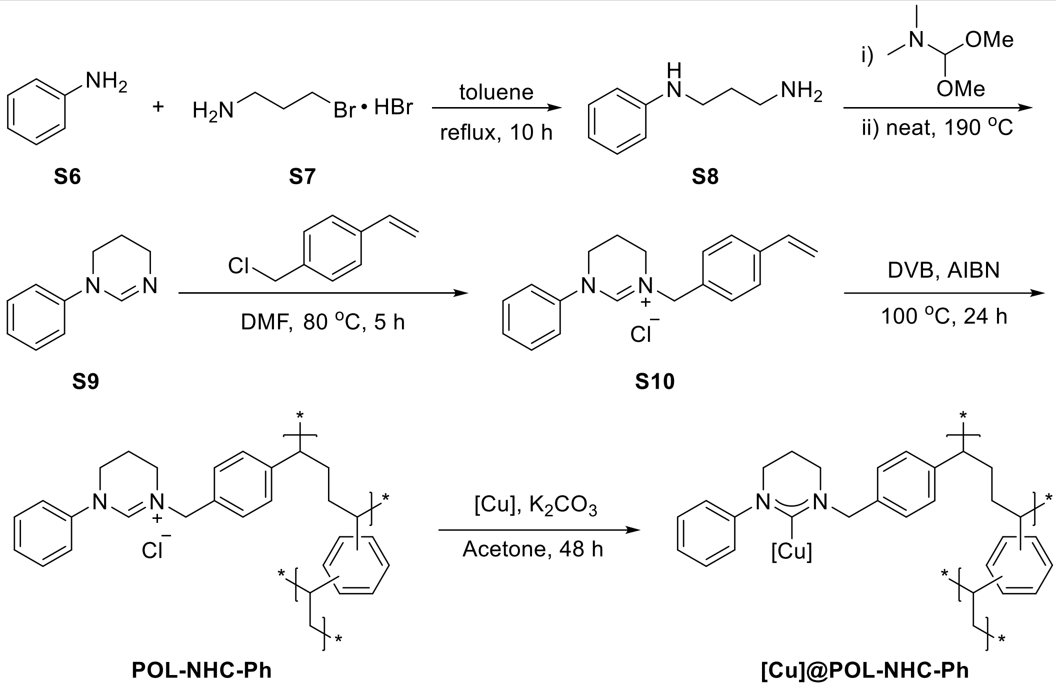

The **S10** was synthesized following similar literature reports.[3] In a 50 mL Schlenk tube, to a suspension of 3-bromopropylamine hydrobromide (**S7**) (11 g, 50 mmol, 1 equiv) and toluene (20 mL, 2.5 M) was added aniline (9.3 g, 100 mmol, 2 equiv) and the mixture was heated at reflux for 10 h. The cooled solution was opened to H_2O (50 mL) and 2 M KOH soln (50 mL) was added. The aqueous layer was extracted with Et_2O (3 × 30 mL) and the extracts were washed with aq sat. NaCl soln (100 mL) and dried with Na_2SO_4, filtered and concentrated. The residual yellow oil was purified by column chromatography (EA:MeOH:Et_3N = 17:2:1, R_f = 0.3) to give the diamine **S8** (6.8 g, 90%) as a light yellow oil.

N,*N*−dimethylformamide dimethylacetal (1.8 g, 15.1 mmol, 1.18 equiv) was added to **S8** (1.92 g, 12.8 mmol, 1 equiv). The mixture was stirred at 90 °C for 0.5 h. Volatiles were removed under reduced pressure and dry the product under vacuum for 2 h. After drying, the product is heated to 190 °C under Ar atmosphere and solvent−free conditions to react for 30 min. After cooling to room temperature, the residue was purified by column chromatography (EA:MeOH:Et_3N = 100:10:1, R_f = 0.3) to obtain 1-phenyl-1,4,5,6-tetrahydropyrimidine **S9** (1.19 g, 58%) as a brown liquid.

To a solution of **S9** (1.6 g, 10 mmol, 1 equiv) in DMF (10 mL, 1 M) was added slowly 4-vinylbenzyl chloride (1.54 g, 10.1 mmol, 1.01 equiv) at rt and the resulting mixture was stirred at 80 °C for 8 h. The residue was purified by column chromatography (DCM:MeOH = 15:1, R_f = 0.2) to obtain **S10** (2.5 g, 8 mmol, 80% yield) as a colorless liquid.

In a 50 mL Schlenk tube, DVB (1.56 g, 12 mmol, 2 equiv) and **S10** (1.88 g, 6 mmol, 1 equiv) were added to a solution containing 49 mg of AIBN (0.3 mmol, 0.05 equiv) and 12 mL DMF (0.5 M). The mixture was hydrothermally treated at 100 °C for 24 h. When the reaction was completed, the solution was filtered and washed with EA and MeOH 3 times. Then the pure white solid POL-NHC was obtained.

The [Cu]@POL-NHC-Ph ([Cu] = CuCl, $Cu(OTf)_2$, $CuCl_2$ and CuBr) was synthesized following similar literature reports.[2] A vial was charged with POL-NHC-Ph (200 mg), [Cu] (20 mg, 1.0 equiv) and K_2CO_3 (2.0 equiv). The mixture was dissolved in acetone (10 mL) and stirred at 60 °C for 48 h. The solution was then filtered through silica which was washed with DCM (3 × 5 mL). The filtered solid was dried under vacuum to obtain catalyst [Cu]@POL-NHC-Ph.

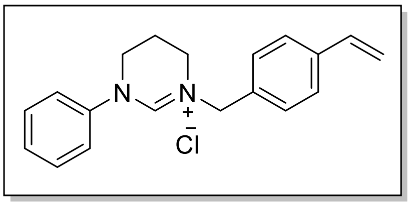

3-Phenyl-1-(4-vinylbenzyl)-3,4,5,6-tetrahydropyrimidin-1-ium chloride (**C3**)
^{1}H NMR (600 MHz, DMSO-*d₆*): δ 9.26 (s, 1H), 7.61−7.58 (m, 2H), 7.57−7.52 (m, 4H), 7.52−7.48 (m, 2H), 7.43−7.41 (m, 1H), 6.76 (dd, J_1 = 10.9 Hz, J_2 = 17.6 Hz, 1H), 5.88 (d, *J* = 17.7 Hz, 1H), 5.30 (d, *J* = 11.1 Hz, 1H), 4.88 (s, 2H), 3.87 (t, *J* = 5.6 Hz, 2H), 3.33 (t, *J* = 5.7 Hz, 2H), 2.14−2.08 (m, 2H);
^{13}C NMR (151 MHz, DMSO-d_6): δ 153.2, 142.3, 127.9, 136.6, 134.0, 130.2, 129.5, 128.1, 127.0, 122.2, 115.5, 58.0, 45.3, 42.9, 19.0;
HRMS−ESI: *m/z* $[M]^+$ calcd for $C_{19}H_{21}N_2$: 277.1699, found 277.1712.

2.1.3 Synthesis of Cu metallized porous organic polymers CuCl@POL-NHC-BI.

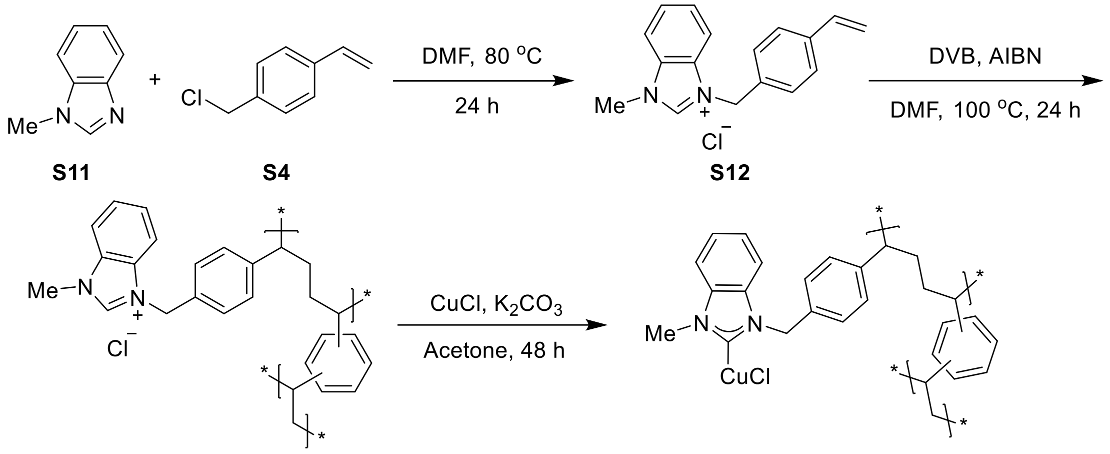

S11 **S4** **S12**

POL-NHC-BI **[Cu]@POL-NHC-BI**

To a solution of **S11** (4.0 g, 30 mmol, 1 equiv) in DMF (30 mL, 1 M) was added slowly 1-(chloromethyl)-4-vinylbenzene **S4** (4.62 g, 30.3 mmol, 1.01 equiv) at rt and the resulting mixture was stirred at 80 °C for 8 h. Diethyl ether was added to obtain a white crystalline solid, which was filtered off. The solid was washed with diethyl ether (3 × 20 mL), and dried under vacuum to obtain product **S12** (7.26 g, 85% yield).

DVB (2.6 g, 20 mmol, 2 equiv) and **S12** (2.85 g, 10 mmol, 1 equiv) were added to a solution containing 82.1 mg AIBN (0.5 mmol, 0.05 equiv) and DMF (20 mL, 0.5 M). The mixture was hydrothermally treated at 100 °C for 24 h. When the reaction was completed, the solution was filtered and washed with EA and MeOH 3 times. Then the pure white solid POL-NHC-BI was obtained.

The CuCl@POL-NHC-BI was synthesized following similar literature reports.[2] A vial was charged with POL-NHC-BI (200 mg), CuCl (20 mg, 1 equiv) and K_2CO_3 (2.0 equiv). The mixture was dissolved in acetone (10 mL) and stirred at 60 °C for 48 h. The solution was then filtered through silica which was washed with DCM (3 × 5 mL). The filtered solid was dried under vacuum to obtain catalyst CuCl@POL-NHC-BI.

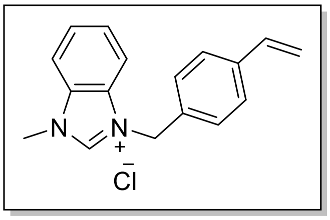

1-Methyl-3-(4-vinylbenzyl)-1H-benzo[d]imidazol-3-ium chloride (**C4**)
Melting point: 109.0−111.0°C
^{1}H NMR (600 MHz, DMSO-d_6): δ 10.10 (s, 1H), 8.07−8.01 (m, 1H), 7.99−7.94 (m, 1H), 7.71−7.62 (m, 2H), 7.55−7.48 (m, 4H), 6.72 (dd, J_1 = 16.4 Hz, J_2 = 26.5 Hz, 1H), 5.85 (dd, J_1 = 1.2 Hz, J_2 = 26.5 Hz, 1H), 5.80 (s, 2H), 5.28 (dd, J_1 = 1.02 Hz, J_2 = 16.4 Hz, 1H), 4.11 (s, 3H);
^{13}C NMR (151 MHz, DMSO-d_6): δ 143.5, 138.0, 136.4, 134.1, 132.5, 131.1, 129.2, 127.2, 127.1, 127.05, 127.0, 115.7, 114.24, 114.2, 49.9, 33.9;
HRMS-ESI: *m/z* $[M]^+$ calcd for $C_{17}H_{17}N_2$: 249.1386, found 249.1395.

2.1.4 Synthesis of Cu metallized porous organic polymers CuCl@POL-NHC-PI.

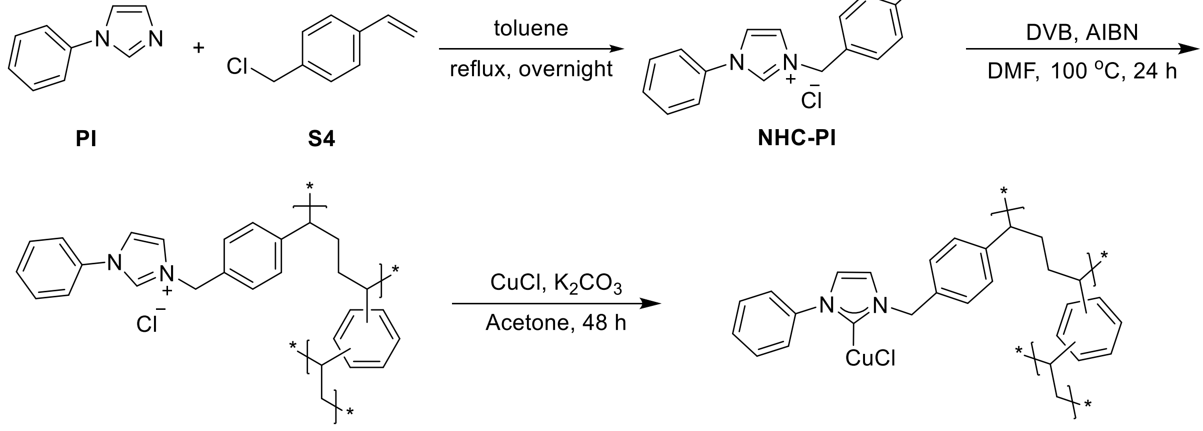

PI **S4** **NHC-PI**

POL-NHC-PI **[Cu]@POL-NHC-PI**

A mixture of phenyl imidazole (PI) (1.44 g, 10 mmol, 1 equiv) and 1-(chloromethyl)-4-vinylbenzene **S4** (1.53 g, 10 mmol, 1 equiv) in toluene (30 mL) was refluxed overnight in a round bottom flask equipped with a condenser. After cooling to room temperature NHC-PI was obtained through filtration as white solid (2.11 g, 71% yield).

DVB (1.3 g, 10 mmol, 2 equiv) and NHC-PI (1.48 g, 5 mmol, 1 equiv) were added to a solution containing 41 mg AIBN (0.25 mmol, 0.05 equiv) and DMF (10 mL, 0.5 M). The mixture was hydrothermally treated at 100 °C for 24 h. When the reaction was completed, the solution was filtered and washed with EA and MeOH 3 times. Then the pure white solid POL-NHC-PI was obtained.

The CuCl@POL-NHC-PI was synthesized following similar literature reports.[2] A vial was charged with POL-NHC-BI (200 mg), CuCl (20 mg, 1 equiv) and K_2CO_3 (2.0 equiv). The mixture was dissolved in acetone (10 mL) and stirred at 60 °C for 48 h. The solution was then filtered through silica which was washed with DCM (3 × 5 mL). The filtered solid was dried under vacuum to obtain catalyst CuCl@POL-NHC-PI.

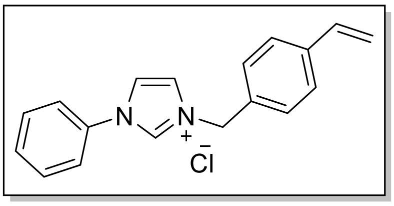

1-Phenyl-3-(4-vinylbenzyl)-1H-imidazol-3-ium chloride
Melting point: 56.5−58.2°C
^{1}H NMR (400 MHz, DMSO-d_6): δ 10.39 (s, 1H), 8.39 (s, 1H), 8.11 (s, 1H), 7.84 (d, *J* = 7.52 Hz, 2H), 7.64 (t, *J* = 7.08 Hz, 2H), 7.60−7.50 (m, 5H), 6.74 (dd, J_1 = 11 Hz, J_2 = 17.6 Hz, 1H), 5.87 (d, J = 17.64 Hz, 1H), 5.56 (s, 2H), 5.29 (d, *J* = 10.88 Hz, 1H);
^{13}C NMR (101 MHz, DMSO-d_6): δ 138.2, 136.5, 136.1, 135.3, 134.6, 130.7, 130.3, 129.6, 127.2, 123.7, 122.3, 122.0, 115.8, 52.5;
HRMS-ESI: *m/z* [M]$^+$ calcd for $C_{18}H_{17}N_2$: 261.1386, found 261.1380.

2.1.5 Synthesis of porous organic polymers A.

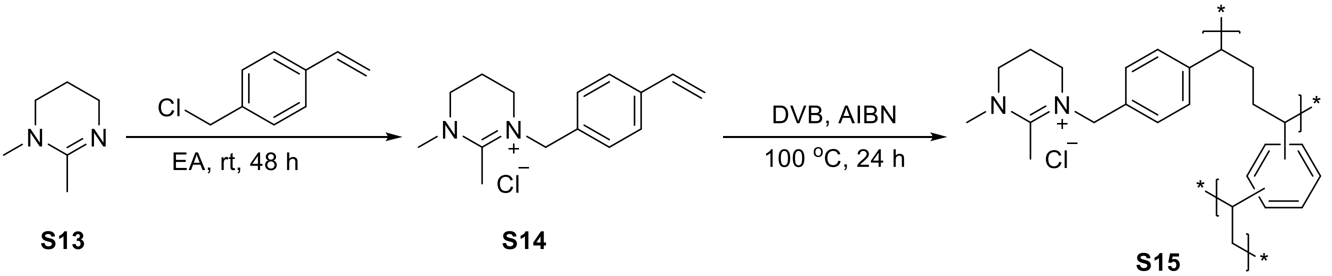

S13 (1.52 g, 10 mmol, 1 equiv) and 1-(chloromethyl)-4-vinylbenzene **S4** (2.02 g, 16 mmol, 1.6 equiv) were added to a flask (50 mL) containing ethyl acetate (EA) (10 mL) and a magnetic stirring bar. The reaction mixture was stirred at room temperature for 48 h The residue was purified by column chromatography (DCM:MeOH = 15:1) to obtain **S14** (2.2 g, 8.3 mmol, 80% yield) as a colorless liquid.

DVB (1.3 g, 10 mmol, 2 equiv) and **S14** (1.32 g, 5 mmol, 1 equiv) were added to a solution containing 41 mg AIBN (0.25 mmol, 0.05 equiv) and DMF (10 mL, 0.5 M). The mixture was hydrothermally treated at 100 °C for 24 h. When the reaction was completed, the solution was filtered and washed with EA and MeOH 3 times. Then the pure white solid **S15** was obtained.

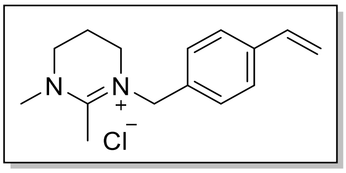

2,3-Dimethyl-1-(4-vinylbenzyl)-3,4,5,6-tetrahydropyrimidin-1-ium chloride
^{1}H NMR (400 MHz, DMSO-d_6): δ 7.59−7.43 (m, 2H), 7.40−7.25 (m, 2H), 6.76 (dd, J1 = 10.96 Hz, J2 = 17.48, 1H), 5.87 (d, J = 17.68 Hz, 1H), 5.29 (d, J = 10.88 Hz, 1H), 4.48 (s, 1H), 3.54−3.44 (4, H), 3.23 (s, 3H), 2.37 (s, 3H), 2.03−1.97 (m, 2H);
^{13}C NMR (101 MHz, DMSO-d_6): δ 162.6, 137.2, 136.8, 135.3, 127.6, 127.1, 115.2, 55.9, 49.0, 47.2, 41.1, 19.5, 16.9.
HRMS-ESI: *m/z* [M]$^+$ calcd for $C_{15}H_{21}N_2$: 229.1699, found 229.1693.

2.2 Synthesis of 2-ethynyl-5-propylthiophene (11)

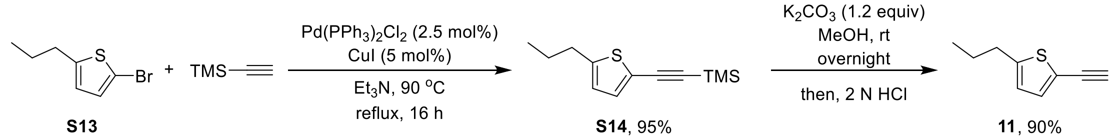

The **S15** was synthesized following similar literature reports.[4] In an argon−flushed two-necked flask and equipped with a stirring bar and a septum were added 2-bromo-5-propylthiophene **S13** (2.05 g, 10 mmol, 1 equiv), $Pd(PPh_3)_2Cl_2$ (175.5 mg, 2.5 mol%), and CuI (95.2 mg, 5 mol%). The flask was flushed with argon before the addition of anhydrous and degassed Et_3N (50 mL). Trimethylsilylacetylene (1.47 g, 15 mmol, 1.5 equiv) was then added drowise. The resulting mixture was heated to reflux and stirred for 16 h, then cooled down to room temperature, filtered over celite and concentrated under reduced pressure. The residue was purified by flash column chromatography (PE, R_f = 0.4) to afford **S14** as a colorless oil (2.1 g, 95%).

In a 25 mL one-necked flask equipped with a stirring bar and a septum were added **S14** (2.0 g, 9 mmol, 1 equiv), MeOH (20 mL) and K_2CO_3 (1.49 g, 10.8 mmol, 1.2 equiv). The reaction was stirred overnight at rt under an argon atmosphere, then quenched with a 2 N aqueous HCl solution (30 mL). The aqueous phase was extracted with diethyl ether (3 × 25 mL). The combined organic layers were dried with $MgSO_4$, filtered and concentrated under reduced pressure at rt. The crude residue was passed through a short pad of silica (PE, R_f = 0.7) to afford 2-propyl-5-ethynyl-thiophene (**11**) as a yellow liquid (1.22 g, 90%), which was reengaged in the next step without further purification.

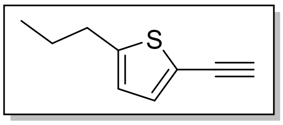

2-Ethynyl-5-propylthiophene (11)
^{1}H NMR (400 MHz, $CDCl_3$): δ 7.12−7.08 (m, 1H), 6.66−6.62 (m, 1H), 3.29 (s, 1H), 2.76 (t, *J* = 7.7 Hz, 2H), 1.73−1.66 (m, 2H), 0.97 (t, *J* = 7.4 Hz, 3H);
^{13}C NMR (101 MHz, $CDCl_3$): δ 148.3, 133.1, 124.0, 119.2, 80.4, 77.4, 32.1, 24.7, 13.6;
HRMS-ESI: *m/z* $[M+H]^+$ calcd for $C_9H_{10}S$: 151.0576, found 151.0575.

2.3 Synthesis of (*E*)-dec-3-en-1-yne (1ah) and (*E*)-tetradec-7-en-5-yne (1ai)

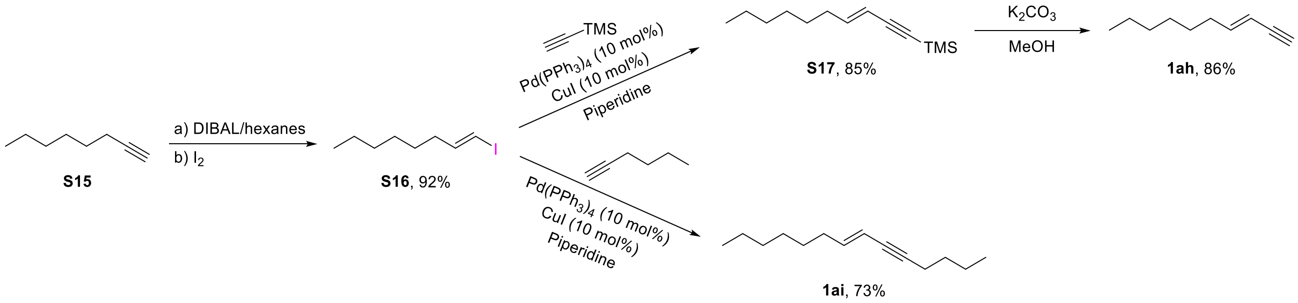

S15 was synthesized according to existing literature reports.[5] To neat 1-octyne (6 mL, 40.7 mmol, 1 equiv) was added DIBAL (1.0 M in hexanes, 40.7 mL, 1 equiv) slowly so the temperature stays below 40 ºC. The reaction is then heated to 50 ºC for 3 h, cooled to rt and the hexane is removed under vacuum. THF (18 mL) is added and the solution is cooled to -50 ºC, and iodine (10.3 g, 40.7 mmol) in THF (20 mL) is slowly added. The mixture is then warmed to rt, wherein it loses almost all of the brownish-red iodine color. The reaction is then quenched with addition of 20% sulfuric acid in a dropwise fashion. When the exotherm slows, the reaction is poured in a mixture of ice and 20% sulfuric acid. The mixture is then extracted with pentane, and the organic extracts were extracted with sodium thiosulfate, and then sodium bicarbonate, dried over $MgSO_4$, and the solvent was removed in vacuo. The residue was purified by flash column chromatography (PE, R_f = 0.9) to afford **S16** as a colorless oil (8.92 g, 92%).

Synthesis of 1ah:[6]

$Pd(PPh_3)_4$ (404.4 mg, 10 mol%) was added at room temperature to a solution of (*E*)-1-iodo-1-octene (833 mg, 3.5 mmol, 1 equiv) in piperidine (35 mL) and stirred for 5 min. Then, a solution of trimethyl trimethylsilylacetylene (3.5 mmol, 344 mg, 1 equiv) was added, followed by the addition of CuI (66.7 mg, 10 mol%). After stirring at room temperature overnight, NH_4Cl (sat. aq. solution) and pentane were added to the reaction mixture. The phases were separated and the aqueous phase was extracted with pentane (× 2). The combined organic layers were washed with brine, dried over magnesium sulfate, filtered and concentrated. The crude product was purified by column chromatography (pentane, R_f = 0.7) affording compound **S17** (620 mg, 85% yield) as a colorless oil.

In a 25 mL one-necked flask equipped with a stirring bar and a septum were added **S17** (553 mg, 2.66 mmol, 1 equiv), MeOH (13.3 mL) and K_2CO_3 (404 mg, 2.93 mmol, 1.1 equiv). The reaction was stirred 4 h at rt under an argon atmosphere, then quenched with a 2 N aqueous HCl solution (10 mL). The aqueous phase was extracted with diethyl ether (3 × 10 mL). The combined organic layers were dried with $MgSO_4$, filtered and concentrated under reduced pressure at rt. The residue was purified by flash column chromatography (PE, R_f = 0.9) to afford **1ah** as a colorless oil (312 mg, 86%).

Synthesis of 1ai:

Same as the first step of synthesizing **1ah**, **1ai** as a colorless oil (73% yield).

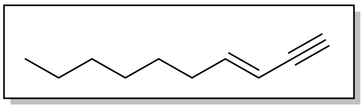

(*E*)-dec-3-en-1-yne (1ah)
^{1}H NMR (400 MHz, $CDCl_3$):δ 6.29−6.19 (m, 1H), 5.50−5.40 (m, 1H), 2.76 (d, *J* = 2.16 Hz, 1H), 2.13−2.08 (m, 2H), 1.41−1.35 (m, 2H), 1.31−1.24 (m, 6H), 0.88 (t, *J* = 6.72 Hz, 3H);
^{13}C NMR (101 MHz, $CDCl_3$): δ 146.9, 108.4, 82.5, 75.5, 33.0, 31.6, 28.7, 28.5, 22.5, 14.0.

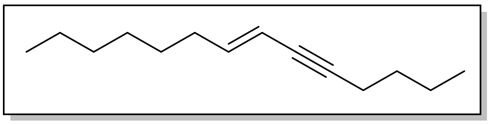

(E)-tetradec-7-en-5-yne (1ai)
^{1}H NMR (400 MHz, $CDCl_3$): δ 6.05−5.95 (m, 1H), 5.46−5.35 (m, 1H), 2.28−2.23 (m, 2H), 2.04 (q, *J* = 6.92 Hz, 2H), 1.50−1.45 (m, 2H), 1.42−1.32 (m, 4H), 1.29−1.22 (m, 6H), 0.90−0.84 (m, 6H);
^{13}C NMR (101 MHz, $CDCl_3$): δ 143.0, 109.8, 88.4, 79.1, 32.9, 31.6, 30.9, 28.80, 28.76, 22.6, 21.9, 19.0, 14.0, 13.5.

2.4 Synthesis of *N*-methyl-*N*-(prop-2-yn-1-yl)aniline (1al)

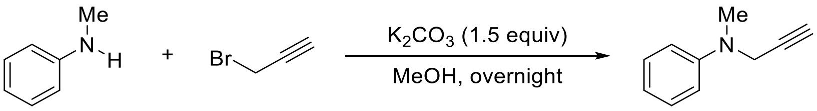

1al, 82%

N-methyl-*N*-(prop-2-yn-1-yl)aniline (**1al**) was synthesized following literature procedure.[7] A mixture of *N*-methylaniline (589 mg, 5.5 mmol, 1.1 equiv), 3-bromo-1-propyne (595 mg, 5 mmol, 1 equiv), and potassium carbonate (1.04 g, 7.5 mmol, 1.5 equiv) in acetonitrile (10 mL) was stirred for 12 h at room temperature. The resulting mixture was filtered through a pad of Celite and the filtrate was concentrated. The residue was purified by chromatography on silica gel (PE, R_f = 0.2) to obtain *N*-methyl-*N*-(prop-2-ynyl)aniline (**1al**) (595 mg, 82% yield) as yellow-orange oil.
^{1}H NMR (400 MHz, $CDCl_3$): δ 7.30−7.23 (m, 2H), 6.89−6.84 (m, 2H), 6.81 (t, *J* = 7.28 Hz, 1H), 4.05 (d, *J* = 2.32 Hz, 2H), 2.97 (s, 3H), 2.17 (t, *J* = 2.36 Hz, 1H);
^{13}C NMR (101 MHz, $CDCl_3$): δ 148.9, 129.1, 118.3, 114.3, 79.2, 72.0, 42.5, 38.6;
HRMS-ESI: m/z [M+H]+ calcd for C10H11N: 146.0964, found 146.0968.

2.5 Synthesis of 1-(But-1-yn-1-yl)-4-methoxybenzene (14)

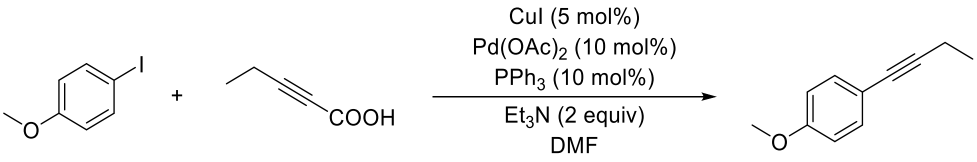

14, 70%

1-(But-1-yn-1-yl)-4-methoxybenzene (**14**) was synthesized following literature procedure.[8] A sealed flask was loaded with 4-iodoanisole (1.17 g, 5 mmol, 1 equiv) and alkyne acid (981 mg, 10 mmol, 2 equiv) in DMF (30 mL), was added copper (I) iodide (48 mg, 0.25 mmol, 0.05 equiv), palladium (II) acetate (113.3 mg, 0.5 mmol, 0.1 equiv), triphenylphosphine (131.1 mg, 0.5 mmol, 0.1 equiv) and triethylamine (1012 mg, 10 mmol, 2 equiv). The flask was filled with argon under argon and sealed. The mixture was stirred for two days at room temperature or for one day at 50 °C. After the reaction was completed, the mixture was extracted three times (3 × 50 mL) with saturated brine and then dried over anhydrous Na_2SO_4. The solvent was removed by vacuum and the crude mixture was purified by flash column chromatography on silica gel using PE (R_f = 0.2) as the eluent to generate the **14** (561 mg, 70%) as yellow oil.
^{1}H NMR (400 MHz, $CDCl_3$): δ 7.37−7.30 (m, 2H), 6.85−6.78 (m, 2H), 3.80 (s, 3H), 2.40 (q, *J* = 7.48 Hz, 2H), 1.23 (t, *J* = 7.56 Hz, 3H);
^{13}C NMR (101 MHz, $CDCl_3$): δ 159.0, 132.8, 116.1, 113.8, 99.0, 79.5, 55.2, 14.0, 13.1;
HRMS-ESI: *m/z* $[M+Na]^+$ calcd for $C_{10}H_{12}O$: 183.0780, found 183.0783.

3. Results and Discussion

3.1 Optimization studies

Table S1. Screening of base and the amount of base [a]

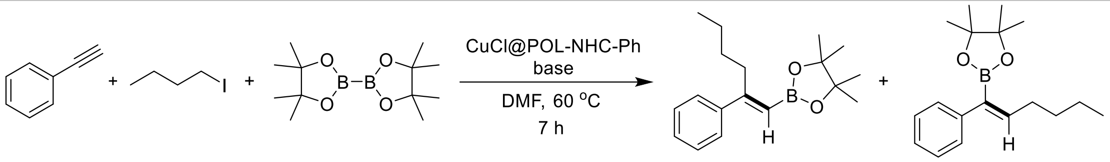

1a **2a** **3a** **4a** **4a'**

Entry	Base	Base equiv	Yield/%[b]	Ratio of **4a**:**4a'**[c]
1	NaO^{t}Bu	1.5	88	>99:1
2	KO^{t}Bu	1.5	trace	−
3	LiO^{t}Bu	1.5	62	95:5
4	NaOMe	1.5	0	−
5	NaO^{t}Bu	1.0	57	92:8
6	NaO^{t}Bu	2.0	76	96:4
7	NaO^{t}Bu	2.5	72	99:1
8[d]	NaO^{t}Bu	1.5	65	96:4
9[e]	NaO^{t}Bu	1.5	85	93:7

[a] Reaction conditions: **1a** (0.5 mmol), **2a** (2 equiv), **3a** (1.5 equiv), CuCl@POL-NHC-Ph (20 mg, 4.67 wt% Cu), base, DMF (0.25 M), 60 °C, 7 h. [b] Isolated yield. [c] Determined using ^{1}H NMR spectroscopy. [d] 1 equiv B_2pin_2; [e] 2 equiv B_2pin_2.

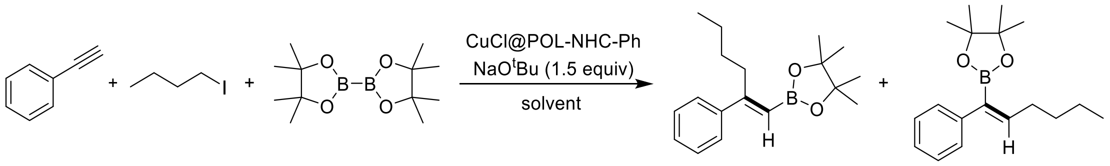

1a **2a** **3** **4a** **4a'**

Table S2. Screening of solvent, reaction temperature and time[a]

Entry	Solvent	T/°C	t/h	Yield/%[b]	Ratio of **4a**:**4a'** [c]
1	DMF	50	7	78	98:2
2	DMF	60	7	88	99:1
3	DMF	70	7	86	97:3
4	DMF	80	7	87	96:4
5	DMF	60	5	86	99:1
6	DMF	60	6	87	99:1
7	DMF	60	8	88	99:1
8	DMA	60	7	42	94:6
9	THF	60	7	trace	−
10	DMSO	60	7	trace	−
11	1,4-dioxane	60	7	0	−
12	toluene	60	7	0	−
13	MeCN	60	7	63	96:4

[a] Reaction conditions: **1a** (0.5 mmol), **2a** (2 equiv), **3a** (1.5 equiv), CuCl@POL-NHC-Ph (20 mg, 4.67 wt% Cu), NaO^{t}Bu (1.5 equiv), solvent (0.25 M). [b] Isolated yield. [c] Determined using ^{1}H NMR spectroscopy.

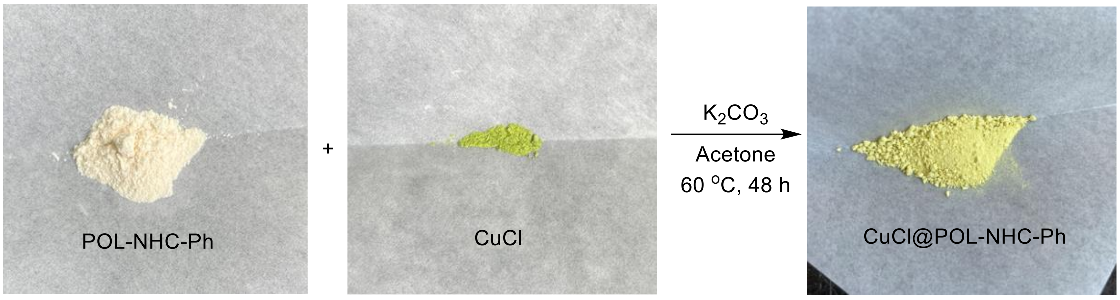

General procedure for 1,2-carboboration of alkynes: In an argon-filled glovebox, the [B]-[B] (0.75 mmol, 1.5 equiv), NaO^{t}Bu (0.75 mmol, 1.5 equiv), CuCl@POL-NHC-Ph (20 mg, 4.67 wt% Cu), and DMF (2 mL, 0.25 M) were added into a 25 mL Schlenk tube with a magnetic stirring bar. After stirring at rt for 10 min, alkynes (0.5 mmol, 1 equiv) and alkyl halide (1 mmol, 2 equiv) were added. The resulting mixture was heated to 60 °C and stirred for 7 h. After the reaction was completed, the solid catalyst was separated by centrifugation and washed with EA. The filtrate was extracted three times (3 × 10 mL) with saturated brine and then dried over anhydrous Na_2SO_4. The solvent was removed by vacuum and the crude mixture was purified by flash column chromatography on silica gel using PE (PE/EA) as the eluent to generate the corresponding product.

Gram-scale synthesis of 4a: In an argon-filled glovebox, the B_2pin_2 (3.81 g, 15 mmol, 1.5 equiv), NaO^{t}Bu (1.44 g, 15 mmol, 1.5 equiv), CuCl@POL-NHC-Ph (400 mg, 4.67 wt% Cu), and DMF (40 mL, 0.25 M) were added into a 25 mL Schlenk tube with a magnetic stirring bar. After stirring at rt for 10 min, phenylacetylene (1.02 g, 10 mmol, 1 equiv) and 1-iodobutane (3.68 g, 20 mmol, 2 equiv) were added. The resulting mixture was heated to 60 °C and stirred for 7 h. After the reaction was completed, the solid catalyst was separated by centrifugation and washed with EA. The filtrate was extracted three times (3 × 50 mL) with saturated brine and then dried over anhydrous Na_2SO_4. The solvent was removed by vacuum and the crude mixture was purified by flash column chromatography on silica gel using PE (PE/EA) as the eluent to generate the corresponding product (2.53 g, 85%).

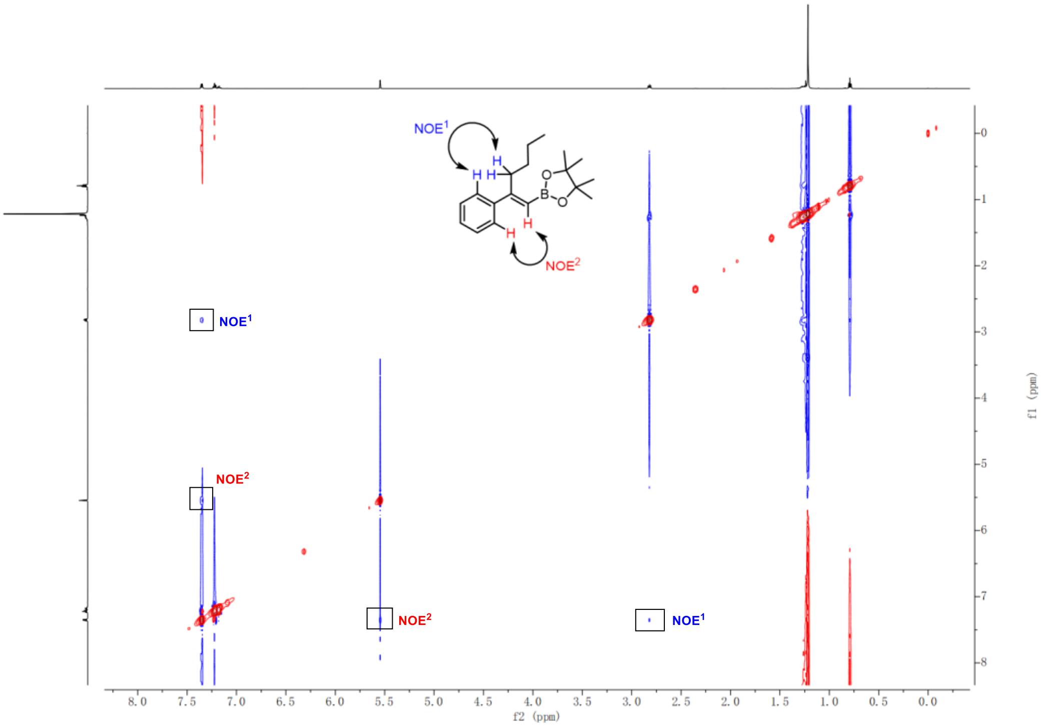

3.2 Transformations of the 4a

3.2.1 Synthesis of (*E*)-1-methoxy-4-(2-phenylhex-1-en-1-yl)benzene (5)

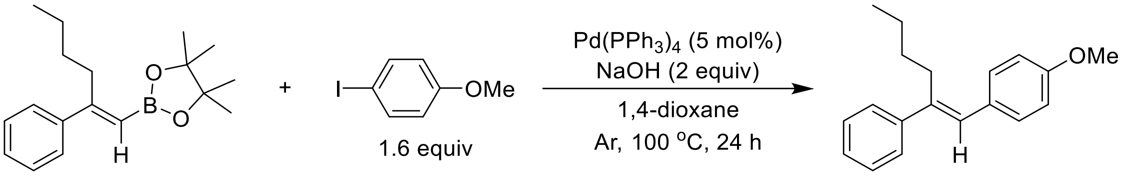

4a **5**, 94%

The **5** was synthesized following similar literature reports.[6] A 25 mL Schlenk tube was charged with **4a** (143.1 mg, 0.5 mmol, 1 equiv), 4-iodoanisole (187.2 mg, 0.8 mmol, 1.6 equiv), $Pd(PPh_3)_4$ (29 mg, 0.025mmol, 5 mol%), NaOH (40 mg, 1 mmol, 2 equiv), degassed 1,4-dioxane (2 mL) under argon. The mixture was heated to 100 °C, 24 h later, stop heating and the mixture was cooled to room temperature. Then the mixture was filtered by a short pad of silica gel and washed with EA. The filtrate was concentrated by rotary evaporation and purified by flash column chromatography (PE:EA = 100:1, R_f = 0.2) to afford **5** (125.2 mg, 94%) as colorless oil.
^{1}H NMR (400 MHz, $CDCl_3$): δ 7.47−7.42 (m, 2H), 7.37−7.32 (m, 2H), 7.29−7.23 (m, 3H), 6.93−6.86 (m, 2H), 6.63 (s, 1H), 3.82 (s, 3H), 2.70 (t, *J* = 7.5 Hz, 2H), 1.46−1.38 (m, 2H), 1.36−1.28 (m, 2H), 0.86 (t, *J* = 7.2 Hz, 3H);
^{13}C NMR (101 MHz, $CDCl_3$): δ 158.2, 143.4, 142.0, 130.9, 129.9, 128.3, 127.6, 126.9, 126.5, 113.6, 55.2, 30.9, 29.9, 22.8, 13.9;
HRMS-ESI: *m/z* [M+Na]$^+$ calcd for $C_{19}H_{22}O$: 267.1743, found 267.1735.

3.2.2 Synthesis of (*E*)-(1-azidohex-1-en-2-yl)benzene (6)

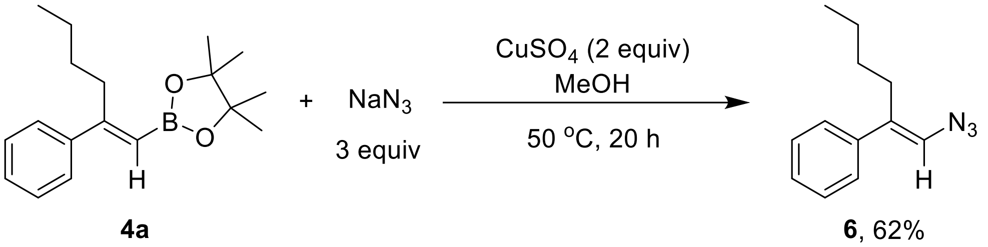

The **6** was synthesized following similar literature reports.[9] A 25 mL Schlenk tube was charged with **4a** (86 mg, 0.3 mmol, 1 equiv), NaN_3 (59 mg, 0.9 mmol, 3 equiv), MeOH (2 mL) and $CuSO_4$ (28.7 mg, 0.18 mmol, 0.6 equiv). The mixture was heated to 50 °C. 20 h later, stop heating and the reaction was cooled to room temperature. The mixture was filtered through a short pad of silica gel and washed with DCM. The filtrate was concentrated by rotary evaporation and purified by flash column chromatography (PE, R_f = 0.7) to afford **6** (37.4 mg, 62%) as colorless oil.
^{1}H NMR (400 MHz, $CDCl_3$): δ 7.32−7.24 (m, 5H), 6.43 (s, 1H), 2.49 (t, J = 7.2 Hz, 2H), 1.35−1.29 (m, 4H), 0.87 (t, J = 7.1 Hz, 3H);
^{13}C NMR (101 MHz, $CDCl_3$): δ 139.2, 132.2, 128.5, 127.1, 126.1, 123.0, 30.2, 28.2, 22.5, 13.9;
HRMS-ESI: m/z $[M+H]^+$ calcd for $C_{12}H_{15}N_3$: 202.1339, found 202.1338.

3.2.3 Synthesis of (*E*)-(1-(vinyloxy)hex-1-en-2-yl)benzene (7)

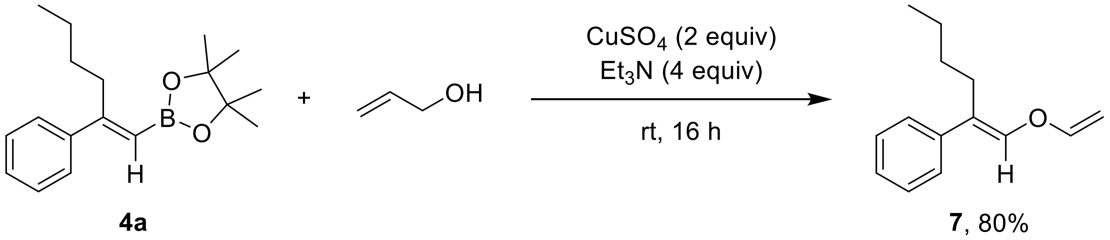

The **7** was synthesized following similar literature reports.[10] A 25 mL Schlenk tube was charged with **4a** (86 mg, 0.3 mmol, 1 equiv), allyl alcohol (1.8 mL), Et_3N (121.4 mg, 1.2 mmol, 4 equiv) and $Cu(OAc)_2$ (109 mg, 0.6 mmol, 2 equiv). The mixture was reacted at room temperature for 16 h. The reaction was diluted in Et_2O and washed with water and brine. The organic phase was dried with Na_2SO_4 and concentrated under vacuum. The crude was purified by flash chromatography (PE:EA = 100:1, R_f = 0.4) to afford **7** (48.6 mg, 80%) as colorless oil.
^{1}H NMR (400 MHz, $CDCl_3$): δ 7.31−7.24 (m, 4H), 7.22−7.13 (m, 1H), 6.35 (s, 1H), 6.04−5.88 (m, 1H), 5.39−5.29 (m, 1H), 5.27−5.20 (m, 1H), 4.35 (d, J = 5.2 Hz, 2H), 2.54 (t, J = 7.0 Hz, 2H), 1.39−1.30 (m, 4H), 0.87 (t, J = 7.1 Hz, 3H);
^{13}C NMR (101 MHz, $CDCl_3$): δ 143.5, 139.9, 134.0, 128.3, 125.8, 120.3, 117.3, 72.9, 30.3, 26.7, 22.6, 13.9;
HRMS-ESI: m/z $[M+H]^+$ calcd for $C_{15}H_{20}O$: 217.1587, found 217.1586.

3.2.4 Synthesis of *tert*-butyl (2*E*,4*E*)-5-phenylnona-2,4-dienoate (8)

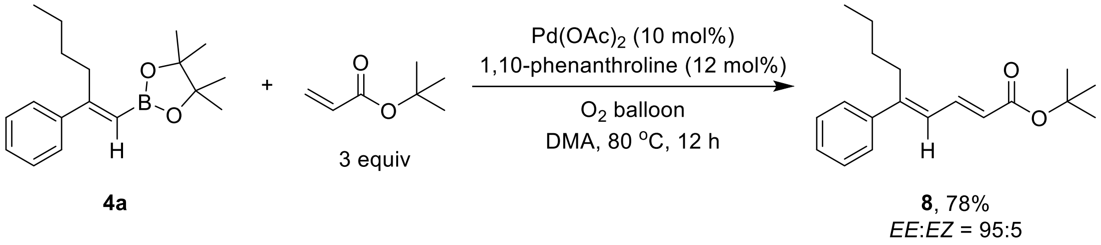

The **8** was synthesized following similar literature reports.[9] A 25 mL Schlenk tube was charged with $Pd(OAc)_2$ (6.8 mg, 0.03 mmol, 10 mol%), 1,10-phenanthroline (6.5 mg, 0.036 mmol, 12 mol%), DMA (2 mL). The mixture was stirred at rt for 30 min before the addition of **4a** (86 mg, 0.3 mmol, 1 equiv) and *tert*-butyl acrylate (115.4 mg, 0.9 mmol, 3 equiv). Then the tube was charged with O_2 balloon and the mixture was heated to 80 °C. 12 h later, stop heating and the mixture was cooled to rt. The mixture was diluted with water and extracted with ether. The organic phase was washed with brine and dried over anhydrous Na_2SO_4. The mixture was filtered and the filtrate was concentrated by rotary evaporation and purified by flash column chromatography (PE:EA = 100:1, R_f = 0.2) to afford **8** (67 mg, 78%) as colorless oil.
^{1}H NMR (400 MHz, $CDCl_3$): δ 7.66 (dd, J_1 = 11.7 Hz, J_2 = 14.9 Hz, 1H), 7.45−7.40 (m, 2H), 7.37−7.26 (m, 3H), 6.43 (d, J = 11.7 Hz, 1H), 5.91 (d, J = 15.0 Hz, 1H), 2.73 (t, J = 7.1 Hz, 2H), 1.51 (s, 9H), 1.42−1.31 (m, 4H), 0.88 (t, J = 7.08 Hz, 3H);
^{13}C NMR (101 MHz, $CDCl_3$): δ 166.8, 150.4, 141.5, 139.5, 128.4, 128.0, 126.4, 125.0, 123.3, 80.1, 31.5, 30.0, 28.2, 22.6, 13.8;
HRMS-ESI: m/z $[M+Na]^+$ calcd for $C_{19}H_{26}O_2$: 309.1825, found 309.1826.

3.2.5 Synthesis of (*E*)-(1-chlorohex-1-en-2-yl)benzene (9)

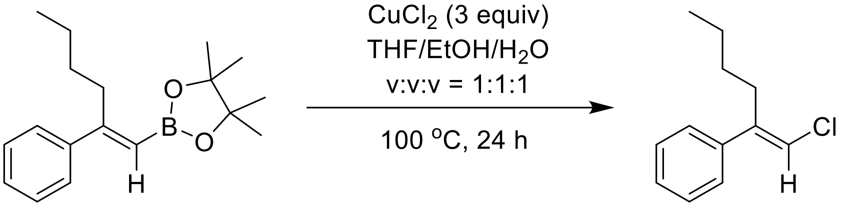

4a **9**, 81%

The **9** was synthesized following similar literature reports.[9] A 25 mL Schlenk tube was charged with **4a** (86 mg, 0.3 mmol, 1equiv), $CuCl_2$ (121 mg, 0.9 mmol, 3 equiv), THF (1 mL), MeOH (1 mL) and H_2O (1 mL). The reaction was carried out at 100 °C for 24 h. After cooled to rt, the reaction was diluted with water and extracted with EA. The organic phase was washed with brine and dried over anhydrous Na_2SO_4. The mixture was filtered and the filtrate was concentrated by rotary evaporation and purified by flash column chromatography (PE, R_f = 0.7) to afford **9** (47.3 mg, 81%) as colorless oil.
^{1}H NMR (400 MHz, $CDCl_3$): δ 7.34−7.26 (m, 5H), 6.20 (s, 1H), 2.67 (t, J = 7.1 Hz, 2H), 1.41−1.30 (m, 4H), 0.88 (t, J = 7.0 Hz, 3H);
^{13}C NMR (101 MHz, $CDCl_3$): δ 143.6, 139.6, 128.5, 127.7, 126.5, 115.4, 30.3, 29.5, 22.4, 13.8;
HRMS-ESI: m/z $[M]^+$ calcd for $C_{12}H_{15}Cl$: 194.0862, found 194.0856.

3.2.6 Synthesis of (*E*)-(1-bromohex-1-en-2-yl)benzene (10)

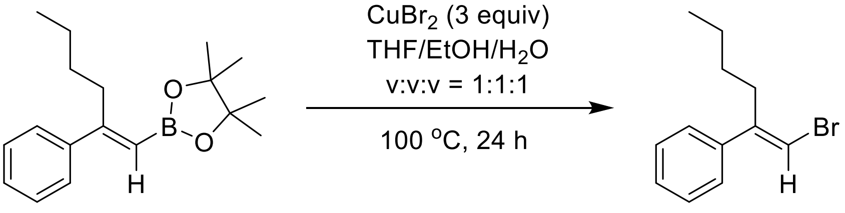

4a **10**, 85%

The **10** was synthesized following similar literature reports.[9] A 25 mL Schlenk tube was charged with **4a** (86 mg, 0.3 mmol, 1 equiv), $CuBr_2$ (201 mg, 0.9 mmol, 3 equiv), EtOH (1 mL) and H_2O (1 mL). The mixture was heated at 100 °C and monitored by TLC. 24 h later, stop heating and the reaction was cooled to room temperature. The reaction was diluted with water and extracted with EA. The organic phase was washed with brine and dried over anhydrous Na2SO4. The mixture was filtered and the filtrate was concentrated by rotary evaporation and purified by flash column chromatography (PE, R_f = 0.7) to afford **10** (61 mg, 85%) as colorless oil.
^{1}H NMR (400 MHz, $CDCl_3$): δ 7.35−7.27 (m, 5H), 6.33 (s, 1H), 2.68 (t, J = 7.2 Hz, 2H), 1.40−1.31 (m, 4H), 0.88 (t, J = 7.0 Hz, 3H);
^{13}C NMR (101 MHz, $CDCl_3$): δ 146.5, 140.3, 128.5, 127.7, 126.5, 104.9, 32.9, 29.5, 22.4, 13.9;
HRMS-ESI: m/z $[M]^+$ calcd for $C_{12}H_{15}Br$: 238.0357, found 238.0350.

3.3 Synthesis of isomer 13 of Namirotene (CBS-211A)

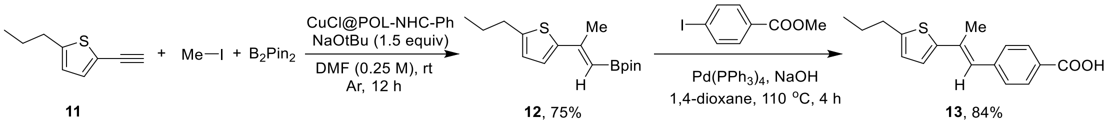

11 **12**, 75% **13**, 84%

In an Argon filled glovebox the B_2pin_2 (761.8 mg, 3 mmol, 1.5 equiv), NaO^tBu (288.3 mg, 3 mmol, 1.5 equiv), CuCl@POL-NHC-Ph (80 mg, 4.67 wt% Cu), and DMF (8 mL, 0.25 M) were added into a 25 mL Schlenk tube with a magnetic stirring bar. After stirring at rt for 10 minutes, 2-ethynyl-5-propylthiophene (300.5 mg, 2 mmol, 1 equiv) and iodomethane (567.8 mg, 4 mmol, 2 equiv) were added. The resulting mixture stirred for 7 h at rt. After the reaction is completed, the solid catalyst was separated by centrifugation and washed with EA. The filtrate was extracted three times (3 × 10 mL) with saturated brine and then dried over anhydrous Na_2SO_4. The solvent was removed by vacuum and the crude mixture was purified by flash column chromatography on silica gel using PE and EA (PE:EA = 100:1, R_f = 0.3) as the eluent to give the corresponding product (438.4 mg, 75% yield).

The **13** was synthesized following similar literature reports.[6] $Pd(PPh_3)_4$ (36 mg, 0.03 mmol, 10 mol%) was added at rt to a solution of vinyl boronate **12** (87.7 mg, 0.3 mmol, 1 equiv), methyl *p*-iodobenzoate (78.6 mg, 0.3 mmol, 1 equiv) and NaOH (0.9 mmol, 2.0 M in H_2O) in dioxane (3 mL). The reaction mixture was stirred at 110 ºC for 4 h. H_2O was added and the mixture was extracted with EA (3 × 5 mL). The solvent was removed under reduced pressure and the residue was purified by column chromatography (PE:EA = 1:2, R_f = 0.3) affording compound **13** as a white solid (72.2 mg, 84% yield).

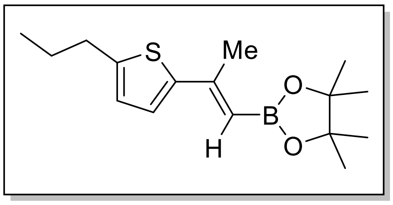

(*E*)-4,4,5,5-tetramethyl-2-(2-(5-propylthiophen-2-yl)prop-1-en-1-yl)-1,3,2-dioxaborolane (**12**)
^{1}H NMR (400 MHz, $CDCl_3$): δ 6.96 (d, *J* = 3.6 Hz, 1H), 6.66 (d, *J* = 3.7 Hz, 1H), 5.69 (s, 1H), 2.73 (t, *J* = 7.5 Hz, 2H), 2.37 (s, 3H), 1.71−1.65 (m, 2H), 1.29 (s, 12H), 0.96 (t, *J* = 7.3 Hz, 3H);
^{13}C NMR (101 MHz, $CDCl_3$): δ 150.8, 146.6, 145.8, 124.8, 124.7, 82.8, 32.4, 24.8, 24.7, 19.3, 13.6;
^{11}B NMR (128 MHz, $CDCl_3$): δ 30.09;
HRMS-ESI: *m/z* $[M+H]^+$ calcd for $C_{16}H_{25}BO_2S$: 292.1777, found 292.1777.

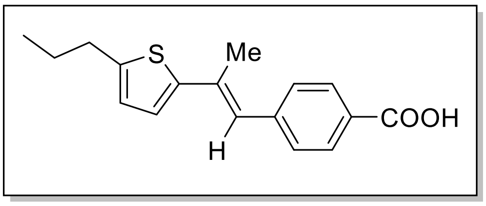

(*E*)-4-(2-(5-propylthiophen-2-yl)prop-1-en-1-yl)benzoic acid (**13**)
Melting point: 134.6-136.0ºC
^{1}H NMR (400 MHz, $CDCl_3$): δ 8.10 (d, *J* = 8.4 Hz, 2H), 7.43 (d, *J* = 8.3 Hz, 2H), 7.00 (d, *J* = 3.6 Hz, 1H), 6.92 (s, 1H), 6.71 (d, *J* = 3.6 Hz, 1H), 2.78 (t, *J* = 7.6 Hz, 2H), 2.30 (d, *J* = 1.1 Hz, 3H), 1.76−1.68 (m, 2H), 1.00 (t, *J* = 7.3 Hz, 3H);
^{13}C NMR (101 MHz, $CDCl_3$): δ 171.7, 145.6, 144.6, 143.5, 133.7, 130.1, 129.2, 126.8, 124.8, 124.1, 123.9, 32.4, 24.8, 17.1, 13.7;
HRMS-ESI: *m/z* $[M-H]^-$ calcd for $C_{17}H_{18}O_2S$: 285.0955, found 285.0954.

3.4 Synthesis of Diethylstilbestrol

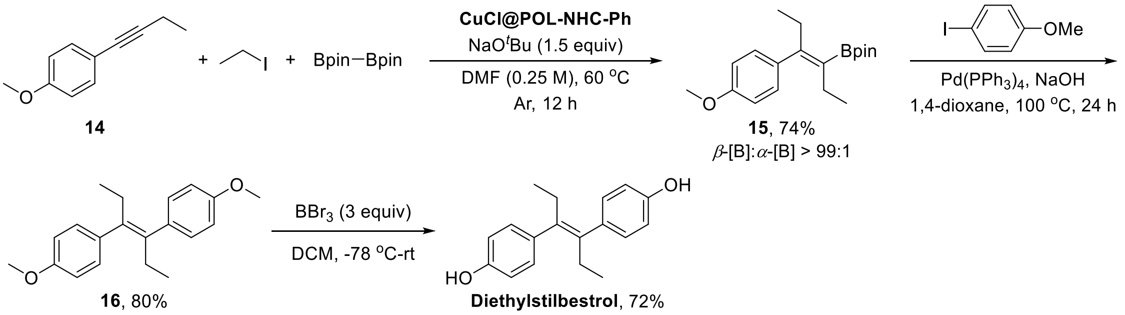

In an Argon filled glovebox the B_2pin_2 (761.8 mg, 3 mmol, 1.5 equiv), NaO^tBu (288.3 mg, 3 mmol, 1.5 equiv), CuCl@POL-NHC-Ph (80 mg, 4.67 wt% Cu), and DMF (8 mL, 0.25 M) were added into a 25 mL Schlenk tube with a magnetic stirring bar. After stirring at rt for 10 minutes, **14** (320 mg, 2 mmol, 1 equiv) and iodoethane (623 mg, 4 mmol, 2 equiv) were added. The resulting mixture stirred for 7 h at rt. After the reaction is completed, the solid catalyst was separated by centrifugation and washed with EA. The filtrate was extracted three times (3 × 10 mL) with saturated brine and then dried over anhydrous Na_2SO_4. The solvent was removed by vacuum and the crude mixture was purified by flash column chromatography on silica gel using PE and EA (PE:EA = 50:1, R_f = 0.2) as the eluent to give the **15** (468 mg, 74% yield) as yellow oil.

The **16** was synthesized following similar literature reports.[6] A 25 mL Schlenk tube was charged with **15** (316 mg, 1 mmol, 1 equiv), 4-iodoanisole (374 mg, 1.6 mmol, 1.6 equiv), $Pd(PPh_3)_4$ (58 mg, 0.025mmol, 5 mol%), NaOH (80 mg, 2 mmol, 2 equiv), degassed 1,4-dioxane (4 mL) under argon. 24 h later, stop heating and the mixture was cooled to room temperature. Then the mixture was filtered by a short pad of silica gel and washed with EA. The filtrate was concentrated by rotary evaporation and purified by flash column chromatography (PE:EA = 500:1, R_f = 0.3) to afford **16** (237 mg, 80%) as white solid.

The **Diethylstilbestol** was synthesized following similar literature reports.[11] The solution of **16** (148 mg, 0.5 mmol, 1 equiv) in dry DCM (3.4 mL) was cooled to -78 ºC and then BBr_3 (376 mg, 1.5 mmol, 3 equiv) was added dropwise under argon atmosphere. The mixture was then stirred for 2 h at room temperature and poured into the mixture of ice/H_2O (350 mL). The mixture was stirred for 30 min and extracted with DCM (2× 5 mL). Combined organic phases were washed with brine (1×10 mL), dried over Na_2SO_4 and concentrated under reduced pressure. The crude product was then purified by column chromatography with PE:EA = 7:1 (R_f = 0.3) as an eluent to afford diethylstilbestrol as an white solid (97 mg, 72%).

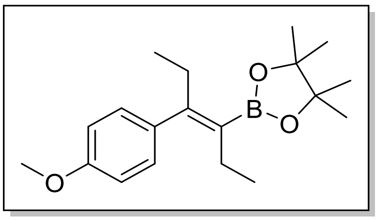

(*Z*)-2-(4-(4-methoxyphenyl)hex-3-en-3-yl)-4,4,5,5-tetramethyl-1,3,2-dioxaborolane (15)
^{1}H NMR (400 MHz, $CDCl_3$): δ 7.06−6.96 (m, 2H), 6.90−6.80 (m, 2H), 3.78 (s, 3H), 2.55 (q, *J* = 7.4 Hz, 2H), 1.97 (q, *J* = 7.48 Hz, 2H), 1.32 (s, 12H), 0.93−0.83 (m, 6H);
^{13}C NMR (101 MHz, $CDCl_3$): δ 157.8, 154.2, 135.2, 129.0, 113.1, 82.9, 54.9, 31.4, 25.4, 24.7, 14.9, 13.7;
^{11}B NMR (128 MHz, $CDCl_3$): δ 30.81;
HRMS-ESI: *m/z* $[M+Na]^+$ calcd for $C_{19}H_{29}BO_3$: 338.2138, found 338.2132.

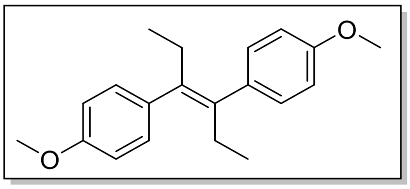

(*E*)-4,4'-(hex-3-ene-3,4-diyl)bis(methoxybenzene) (16)
Melting point: 124−126 ºC;
^{1}H NMR (500 MHz, $CDCl_3$): δ 7.18−7.11 (m, 4H), 6.95−6.90 (m, 4H), 3.85 (s, 6H), 2.15 (q, *J* = 7.4 Hz, 4H), 0.79 (t, *J* = 7.55 Hz, 6H);
^{13}C NMR (125 MHz, $CDCl_3$): δ 157.9, 138.7, 135.0, 129.7, 113.3, 55.1, 28.5, 13.4;
HRMS-ESI: *m/z* $[M+H]^+$ calcd for $C_{20}H_{24}O_2$: 319.1669, found 319.1663.

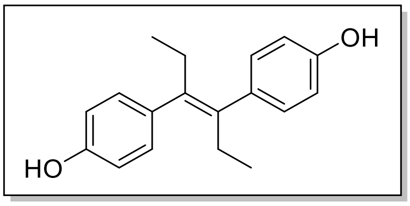

(*E*)-4,4'-(hex-3-ene-3,4−diyl)diphenol (Diethylstilbestrol)
Melting point: 174.8−176.5 ºC;
^{1}H NMR (400 MHz, DMSO-d_6): δ 9.36 (s, 2H), 7.01−6.92 (m, 4H), 6.80−6.71 (m, 4H), 2.05 (q, *J* = 7.36 Hz, 4H), 0.69 (t, *J* = 7.32 Hz, 6H);
^{13}C NMR (101 MHz, DMSO-d_6): δ 156.3, 138.6, 133.2, 130.0, 115.5, 28.7, 13.9;
HRMS-ESI: *m/z* $[M+H]^+$ calcd for $C_{18}H_{20}O_2$: 291.1356, found 291.1352.

3.5 Characterization of catalyst

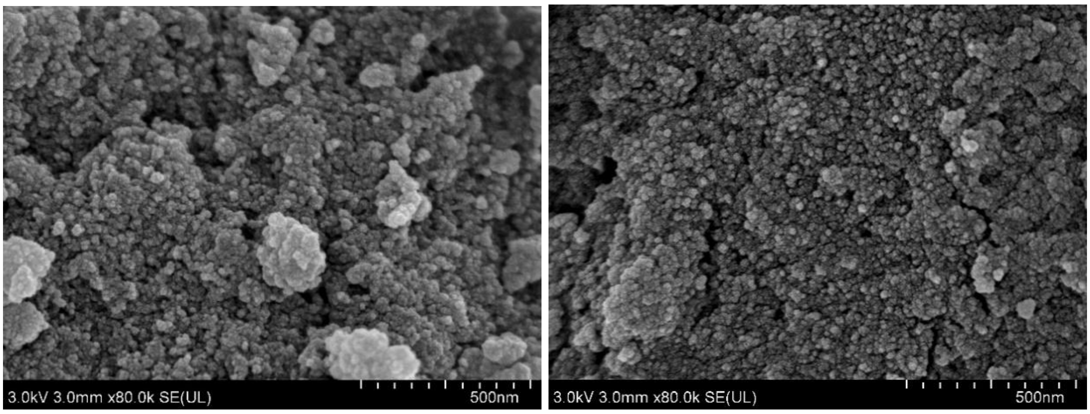

Figure S1. SEM images of POL-NHC-Ph (left) and used CuCl@POL-NHC-Ph (right)

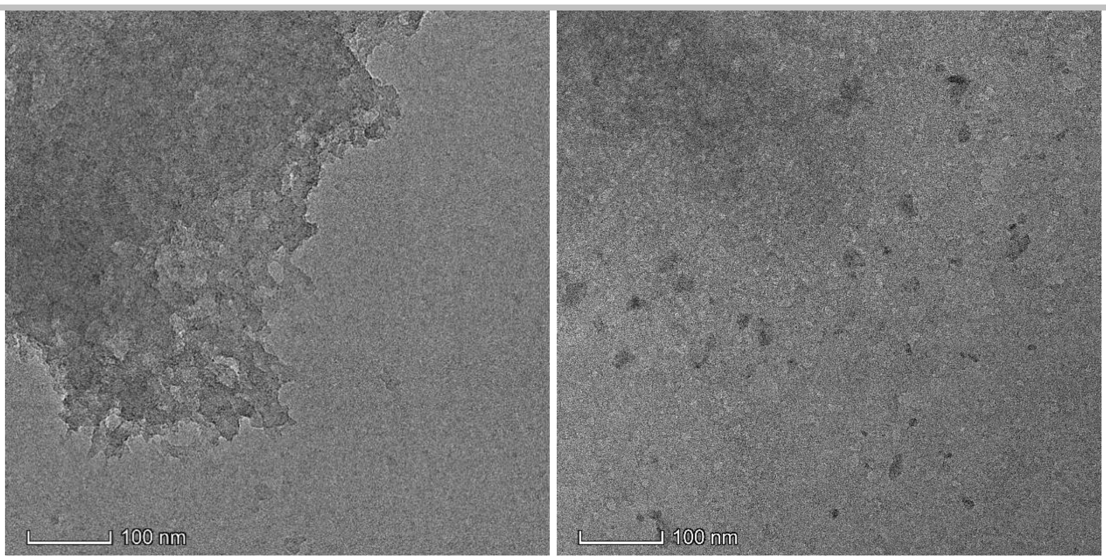

Figure S2. TEM images of POL-NHC-Ph (left) and used CuCl@POL-NHC-Ph (right)

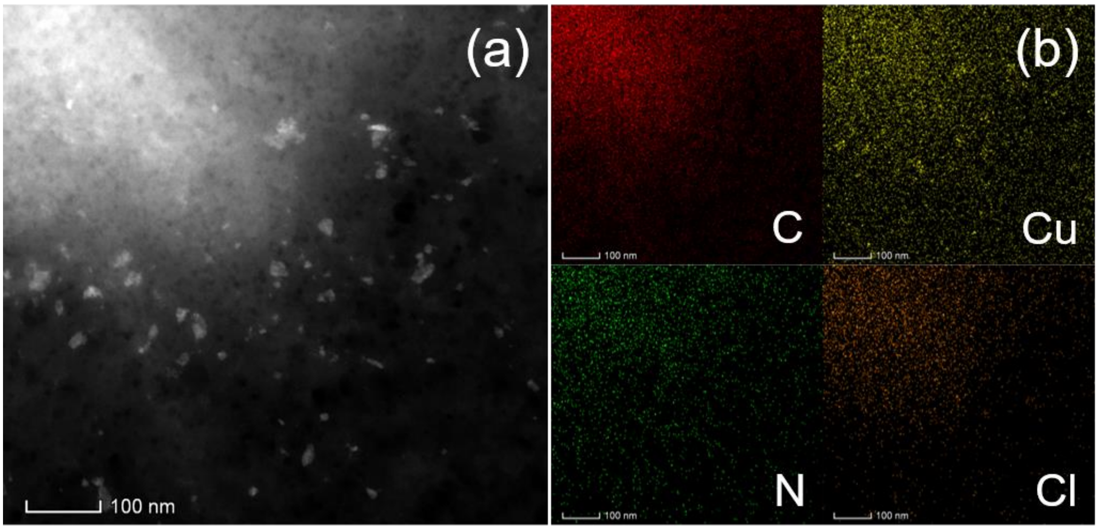

Figure S3. (a) HAADF image of used CuCl@POL-NHC-Ph; (b) EDS elemental mapping analysis of the used CuCl@POL-NHC-Ph

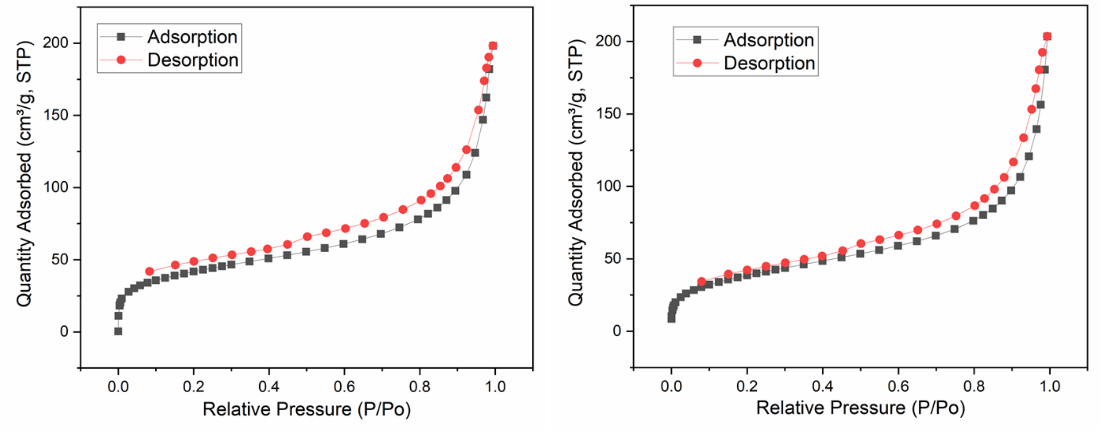

Figure S4. N_2 adsorption–desorption isotherms of POL-NHC-Ph (left) and used CuCl@POL-NHC-Ph (right)

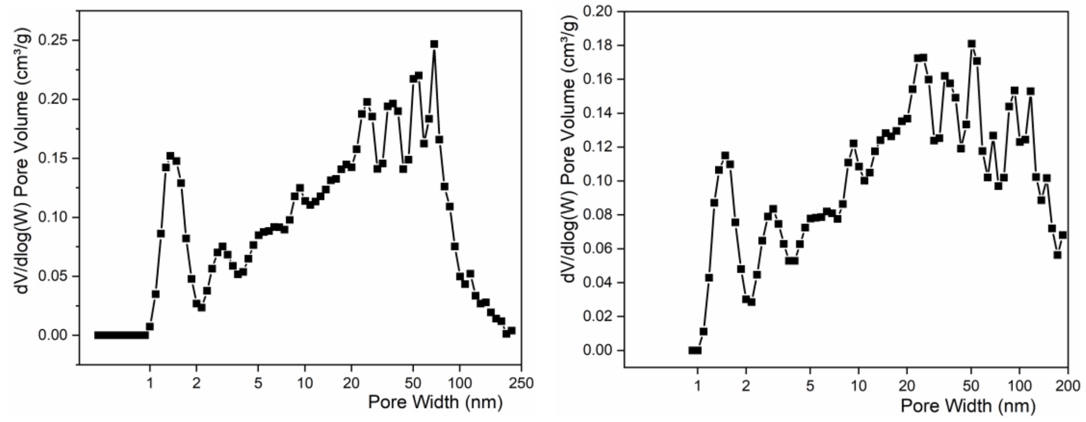

Figure S5. Pore size distribution curves of POL-NHC-Ph (left) and used CuCl@POL-NHC-Ph (right)

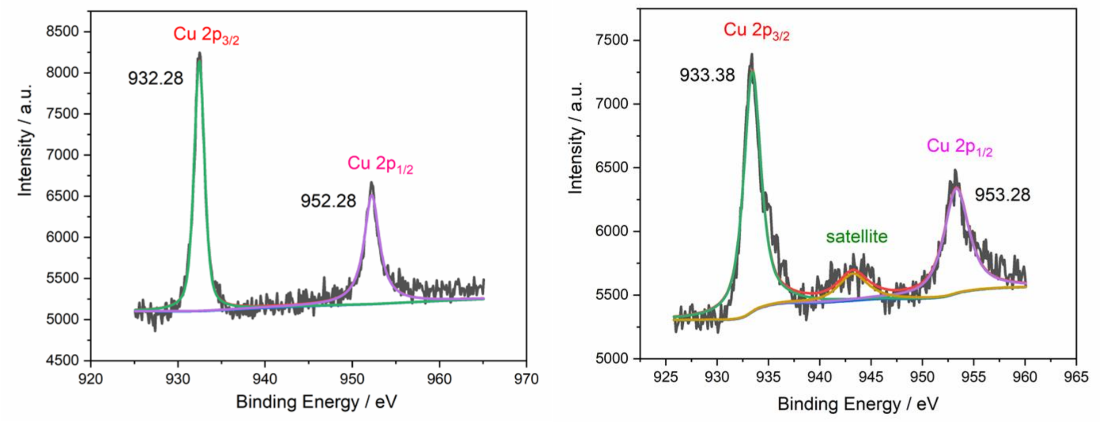

Figure S6. Cu 2p XPS spectra of CuCl and used CuCl@POL-NHC-Ph

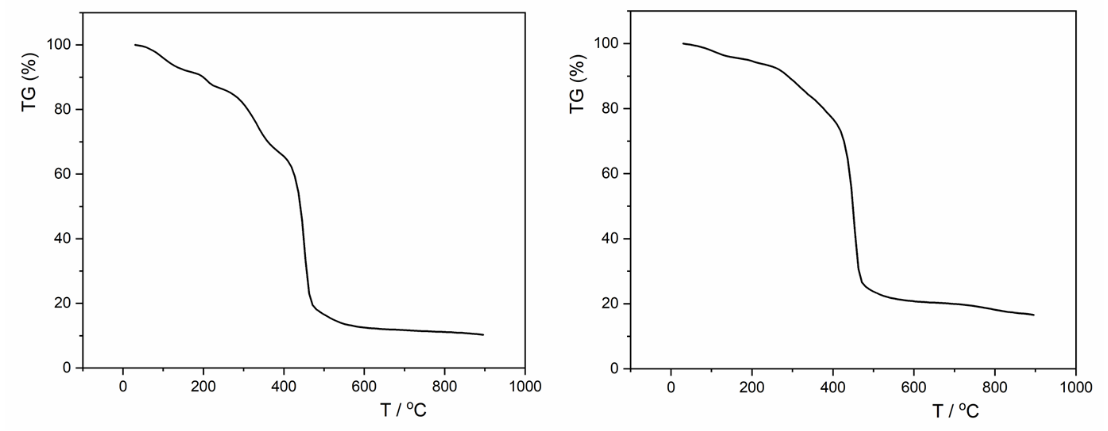

Figure S7. Thermogravimetric analysis of POL-NHC-Ph and CuCl@POL-NHC-Ph.

3.6 Copper percentage of polymer-supported catalyst

The percentage of copper in different polymer-supported was analyzed by ICP-MS.

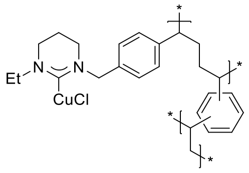

CuCl@POL-NHC-Et

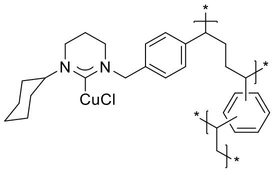

CuCl@POL-NHC-Cy

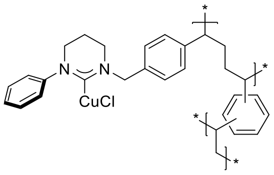

CuCl@POL-NHC-Ph

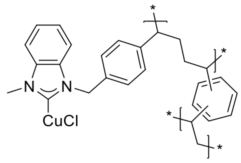

CuCl@POL-NHC-BI

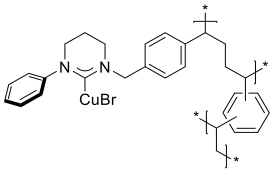

CuBr@POL-NHC-Ph

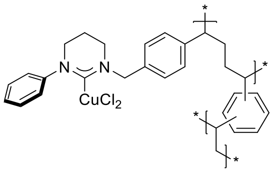

$CuCl_2$@POL-NHC-Ph

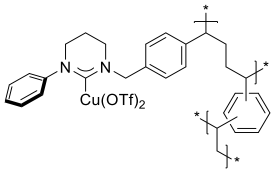

$Cu(OTf)_2$@POL-NHC-Ph

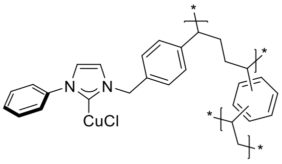

CuCl@POL-NHC-PI

Table S3. Copper percentage of polymer-supported catalyst

Catalyst	wt% Cu	Catalyst	wt% Cu
CuCl@POL-NHC-Et	4.48	CuBr@POL-NHC-Ph	3.91
CuCl@POL-NHC-Cy	4.71	$CuCl_2$@POL-NHC-Ph	4.08
CuCl@POL-NHC-Ph	4.67	$Cu(OTf)_2$@POL-NHC-Ph	1.42
CuCl@POL-NHC-BI	4.62	CuCl@POL-NHC-PI	4.58

Table S4. Conversion rate of NHC

Catalyst	Conv. of POL-NHC/%	Catalyst	Conv. of POL-NHC/%
CuCl@POL-NHC-Et	37	CuBr@POL-NHC-Ph	35
CuCl@POL-NHC-Cy	43	$CuCl_2$@POL-NHC-Ph	37
CuCl@POL-NHC-Ph	42	$Cu(OTf)_2$@POL-NHC-Ph	37
CuCl@POL-NHC-BI	39	CuCl@POL-NHC-PI	40

3.7 Estimate the amount of Cu(II) in the used Cu(I) nano-catalyst.

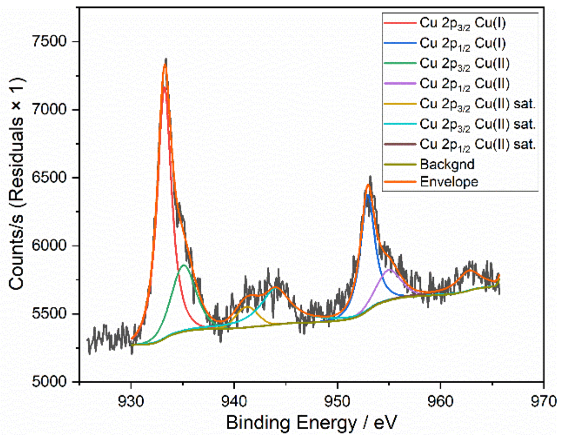

Figure S8. XPS spectrum of the Cu2p state of used CuCl@POL-NHC-Ph nanocatalyst

Table S5. Cu 2p spectrum components of used CuCl@POL-NHC-Ph nanocatalyst

BE, eV	FWHM, eV	AC, at%	
933.2	1.63	53.27	Cu 2p 3/2 Cu(I)
952.9	1.63	0	Cu 2p 1/2 Cu(I)
		Σ 53.27	
935.1	2.77	21.40	Cu 2p 3/2 Cu(II)
955.0	2.77	0	Cu 2p 1/2 Cu(II)
941.2	2.49	5.73	Cu 2p 3/2 Cu(II) satellite
944.0	3.47	19.60	Cu 2p 3/2 Cu(II) satellite
962.7	2.84	0	Cu 2p 1/2 Cu(II) satellite
		Σ 46.73	

The high resolution spectra for the Cu2p region revealed two main peaks located at 933.2 eV and 952.9 eV belonging to Cu(I) as well as peaks at 935.1 eV and 955.0 eV that are characteristic of Cu(II) (**Fig. S8**). Moreover, a collection of satellite features of these peaks are clearly observed at 941.2 eV, 944.0 eV and 962.7 eV, which also indicated the presence of Cu(II) species. The atomic ratio between Cu(I) and Cu(II) can be calculated from their atomic concentrations based on the ratio of the combined integrals of the peaks belonging to Cu(I) to those of the peaks belonging to Cu(II). Thus Cu(I):Cu(II) = 53.27:46.73 = 1.14:1 (Table S5).

3.8 ^{1}H NMR adsorption experiments

The adsorption experiment steps are as follows:

Blank group: 0.5 mmol phenylacetylene, 1-iodobutane or B_2pin_2 were added to a sample bottle, and 2 mL $CDCl_3$ was added. After stirring for 2 h, the ^{1}H NMR was measured.

Experimental group: 0.5 mmol phenylacetylene, 1-iodobutane or B_2pin_2 were added to a sample bottle, then 2 mL $CDCl_3$ and 20 mg CuCl@POL-NHC-Ph were added. After stirring for 2 h, the ^{1}H NMR was measured.

We judged the concentration of the substrate by the signal intensity of the nuclear magnetic hydrogen spectrum. Under the same conditions, the signal of the experimental group was weaker than that of the control group. We believe that it is due to the adsorption effect of CuCl@POL-NHC-Ph on the substrate. The spectrum of the results is shown below:

Adsorption of phenylacetylene

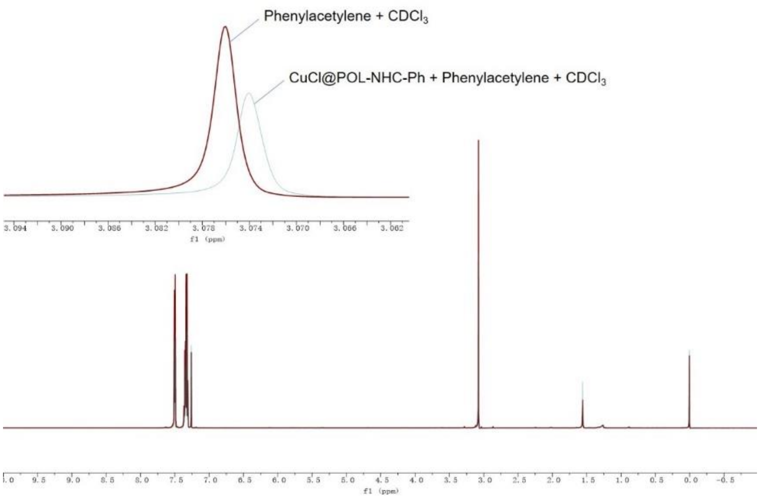

Adsorption of 1-iodobutane

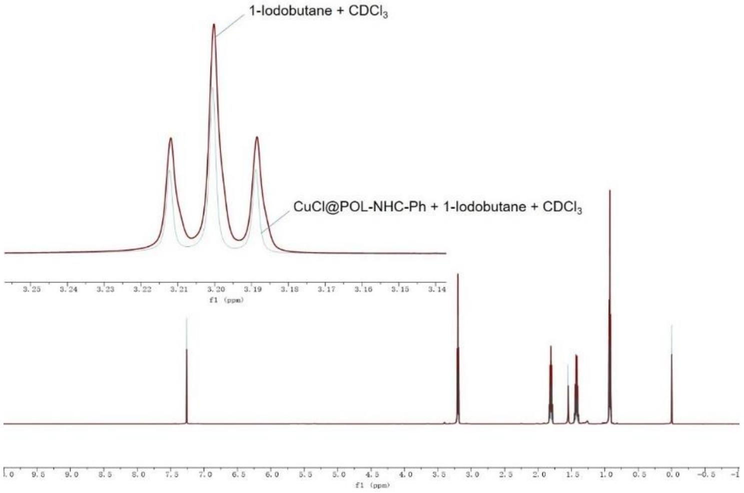

Adsorption of B_2pin_2

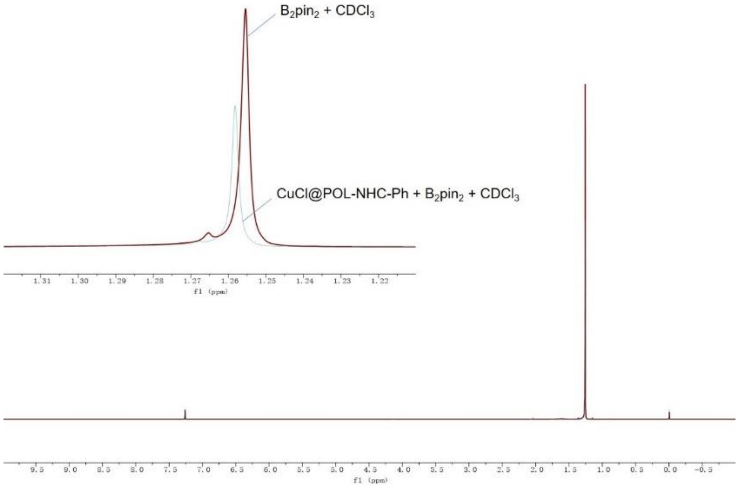

Figure S9. Solid-state ^{13}C NMR test of POL-NHC-Ph

3.9 Solid-state ^{13}C NMR test of POL-NHC-Ph

The results show that the carbon spectrum of the homogeneous ligand NHC-Ph is basically matched with the carbon spectrum of POL-NHC-Ph. The difference in the alkyl region may be due to the influence of the copolymer DVB. It is worth noting that the signal peak of the carbene carbon (δ 152.68) in the homogeneous ligand also appears at the same position in the solid-state NMR, indicating that the carbene structure remains intact in the polymer.

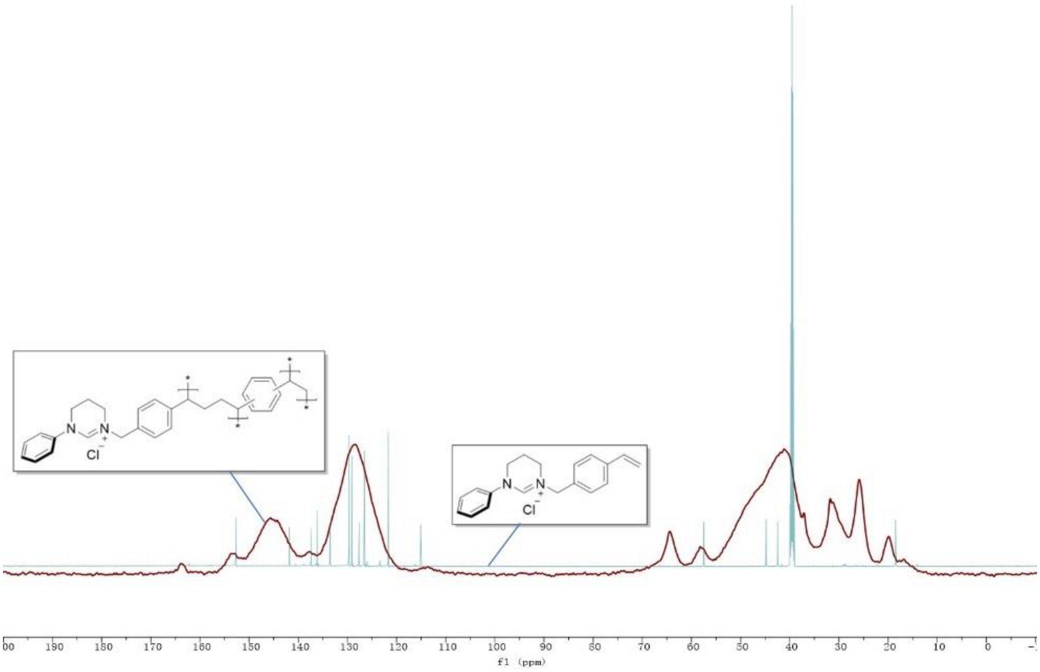

Figure S10. Solid-state ^{13}C NMR test of POL-NHC-Ph

3.10 Infrared test of NHC-Ph and POL-NHC-Ph

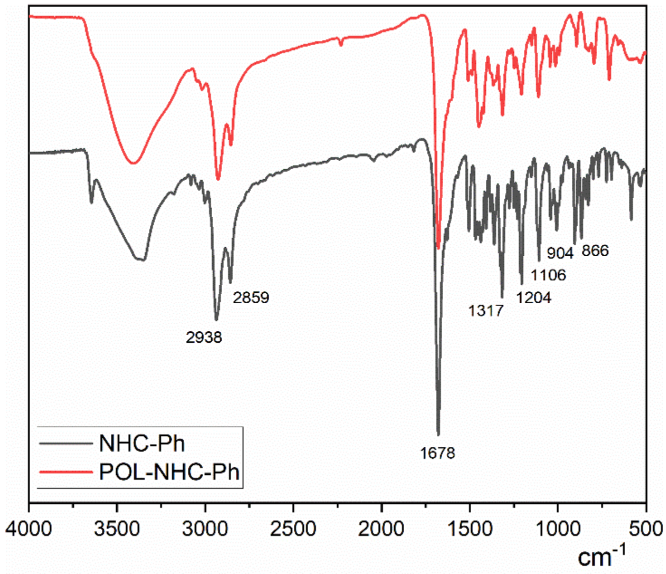

Figure S11. Infrared test of NHC-Ph and POL-NHC-Ph

We tested the infrared spectra of the monomer NHC-Ph and POL-NHC-Ph, respectively. The results are shown below, which indicates that the carbene structure of the polymer is basically maintained. 2938 and 2859 cm^{-1}: –C–H stretching vibration of $–CH_2–$; 1678 cm^{-1}: –C=N stretching vibration; 1317,1204 and 1106 cm^{-1}: In-plane bending vibration of –C–H on benzene ring and skeleton vibration of C–C bond; 904 and 866 cm^{-1}: In-plane bending vibration of benzene ring –C–H. These experimental results have been added to Supporting Information.

3.11 Control experiment

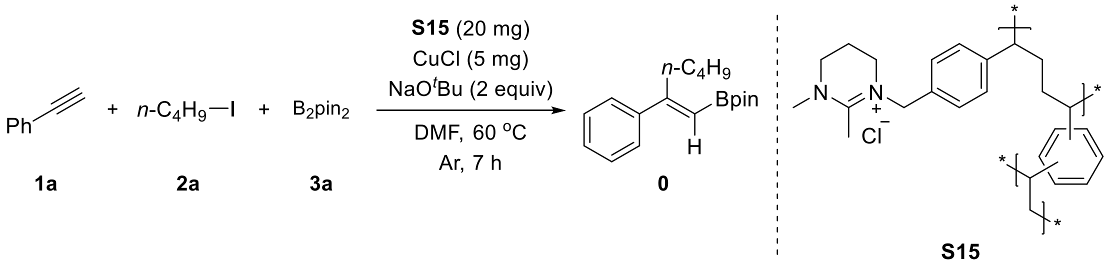

S15

3.12 Computational details

All DFT calculations were performed by using Gaussian 16 package [12]. The geometries optimization and frequencies calculations were computed by employing the B3LYP functional [13,14]. The 6-311G(d,p) basis set was applied to the H, C, N, and Cl atoms [15], and the SDD pseudopotential basis set was applied for Cu atom. In view of large molecule of polymer, the 6-31G(d) basis set was applied to the H, C, N, and Cl atoms. The Grimme's DFT-D3 with BJ-damping method was applied to correct the van der Waals interaction [16]. The analysis of electronic structure was performed by using Multiwfn 3.8 (dev) code [17]. The isosurface maps of various orbitals were rendered by means of Visual Molecular Dynamics (VMD) software [18] based on the files exported by Multiwfn. The isosurface of HOMO-LUMO orbitals was set to 0.05.

3.13 Characterization data of alkenylboronates

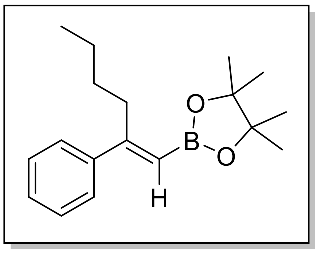

(*E*)-4,4,5,5-tetramethyl-2-(2-phenylhex-1-en-1-yl)-1,3,2-dioxaborolane (**4a**) was isolated (126 mg, 0.44 mmol, 88%) as a colorless oil after chromatograph on silica with PE and EA (eluent: PE:EA = 50:1, R_f = 0.3);
^{1}H NMR (400 MHz, $CDCl_3$): δ 7.46−7.41 (m, 2H), 7.33−7.23 (m, 3H), 5.63 (s, 1H), 2.90 (t, *J* = 7.0 Hz, 2H), 1.36−1.28 (m, 16H), 0.87 (t, *J* = 7.0 Hz, 3H);
^{13}C NMR (101 MHz, $CDCl_3$): δ 163.5, 143.4, 128.1, 127.6, 126.3, 116.0, 82.7, 33.0, 31.9, 24.8, 22.4, 13.8;
^{11}B NMR (128 MHz, $CDCl_3$): δ 30.0;
HRMS-ESI: *m/z* $[M+H]^+$ calcd for $C_{18}H_{27}BO_2$: 287.2177, found 287.2177.

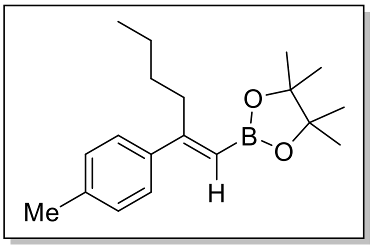

(*E*)-4,4,5,5-tetramethyl-2-(2-(p-tolyl)hex-1-en-1-yl)-1,3,2-dioxaborolane (**4b**) was isolated (135 mg, 0.45 mmol, 90%) as a colorless oil after chromatograph on silica with PE and EA (eluent: PE: EA = 50:1, R_f = 0.3);
^{1}H NMR (400 MHz, $CDCl_3$): δ 7.38−7.31 (m, 2H), 7.15−7.09 (m, 2H), 5.61 (s, 1H), 2.89 (t, *J* = 7.1 Hz, 2H), 2.33 (s, 3H), 1.37−1.28 (m, 16H), 0.87 (t, *J* = 7.1 Hz, 3H);
^{13}C NMR (101 MHz, $CDCl_3$): δ 163.4, 140.3, 137.5, 128.8, 126.2, 115.2, 82.7, 32.9, 32.0, 24.8, 22.4, 21.1, 13.9;
^{11}B NMR (128 MHz, $CDCl_3$): δ 30.0;
HRMS-ESI: *m/z* $[M+H]^+$ calcd for $C_{19}H_{29}BO_2$: 301.2333, found 301.2335.

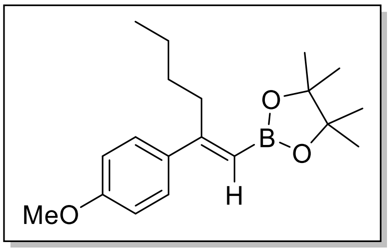

(*E*)-2-(2-(4-methoxyphenyl)hex-1-en-1-yl)-4,4,5,5-tetramethyl-1,3,2-dioxaborolane (**4c**) was isolated (126 mg, 0.4 mmol, 80%) as a colorless oil after chromatograph on silica with PE and EA (eluent: PE: EA = 50:1, R_f = 0.2);
^{1}H NMR (400 MHz, $CDCl_3$): δ 7.45−7.39 (m, 2H), 6.88−6.84 (m, 2H), 5.60 (s, 1H), 3.80 (s, 3H), 2.90 (t, *J* = 7.1 Hz, 2H), 1.40−1.29 (m, 16H), 0.89 (t, *J* = 7.1 Hz, 3H);
^{13}C NMR (101 MHz, $CDCl_3$): δ 162.8, 159.3, 135.4, 127.4, 114.1, 113.4, 82.6, 55.1, 32.7, 32.0, 24.8, 22.4, 13.8;
^{11}B NMR (128 MHz, $CDCl_3$): δ 31.5;
HRMS-ESI: *m/z* $[M+Na]^+$ calcd for $C_{19}H_{29}BO_3$: 339.2102, found 339.2104.

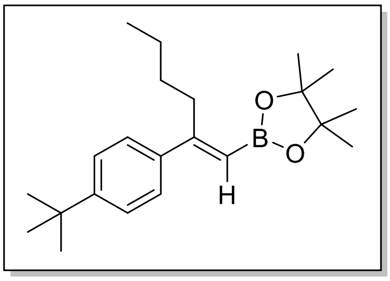

(*E*)-2-(2-(4-(*tert*-butyl)phenyl)hex-1-en-1-yl)-4,4,5,5-tetramethyl-1,3,2-dioxaborolane (**4d**) was isolated (147 mg, 0.43 mmol, 86%) as a colorless oil after chromatograph on silica with PE and EA (eluent: PE: EA = 50:1, R_f = 0.3);
^{1}H NMR (400 MHz, $CDCl_3$): δ 7.46−7.39 (m, 2H), 7.38−7.32 (m, 2H), 5.68 (s, 1H), 2.92 (t, *J* = 7.1 Hz, 2H), 1.44−1.36 (m, 4H), 1.34 (s, 9H), 1.32 (s, 12H), 0.91 (t, *J* = 7.1 Hz, 3H);
^{13}C NMR (101 MHz, $CDCl_3$): δ 163.2, 150.6, 140.1, 125.9, 125.0, 115.0, 82.7, 34.4, 32.8, 32.2, 31.3, 24.8, 22.5, 13.9;

^{11}B NMR (128 MHz, $CDCl_3$): δ 30.4

HRMS-ESI: *m/z* $[M+H]^+$ calcd for $C_{22}H_{35}BO_2$: 343.2803, found 343.2806.

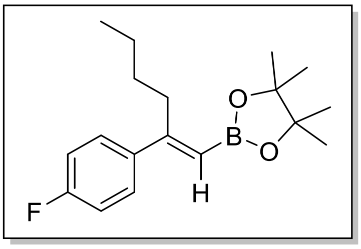

(*E*)-2-(2-(4-fluorophenyl)hex-1-en-1-yl)-4,4,5,5-tetramethyl-1,3,2-dioxaborolane (**4e**) was isolated (134 mg, 0.44 mmol, 88%) as a light yellow oil after chromatograph on silica with PE and EA (eluent: PE: EA = 50:1, R_f = 0.3);

^{1}H NMR (400 MHz, $CDCl_3$): δ 7.43−7.37 (m, 2H), 7.01−6.96 (m, 2H), 5.57 (s, 1H), 2.87 (t, *J* = 6.9 Hz, 2H), 1.36−1.29 (m, 16H), 0.87 (t, *J* = 6.9 Hz, 3H);

^{13}C NMR (101 MHz, $CDCl_3$): δ 162.5 (d, *J* = 245.1 Hz), 162.3, 139.3 (d, *J* = 3.3 Hz), 127.9 (d, *J* = 7.9 Hz), 116.3, 114.9 (d, *J* = 21.1 Hz), 82.9, 33.0, 31.8, 24.8, 22.4, 13.8;

^{19}F NMR (565 MHz, $CDCl_3$): δ -114.8 (s);

^{11}B NMR (128 MHz, $CDCl_3$): δ 30.6;

HRMS-ESI: *m/z* $[M+Na]^+$ calcd for $C_{18}H_{26}BFO_2$: 327.1902, found 327.1885.

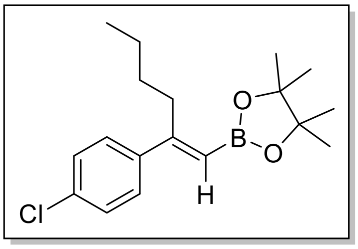

(*E*)-2-(2-(4-chlorophenyl)hex-1-en-1-yl)-4,4,5,5-tetramethyl-1,3,2-dioxaborolane (**4f**) was isolated (135 mg, 0.42 mmol, 84%) as a light yellow oil after chromatograph on silica with PE and EA (eluent: PE: EA = 50:1, R_f = 0.3);

^{1}H NMR (600 MHz, $CDCl_3$): δ 7.29−7.25 (m, 2H), 7.21−7.17 (m, 2H), 5.51 (s, 1H), 2.78 (t, *J* = 7.1 Hz, 2H), 1.26−1.21 (M, 16H), 0.79 (t, *J* = 7.1 Hz, 3H);

^{13}C NMR (151 MHz, $CDCl_3$): δ 162.0, 141.7, 128.3, 127.6, 116.7, 82.9, 32.8, 31.7, 24.8, 22.3, 13.8;

^{11}B NMR (192 MHz, $CDCl_3$): δ 30.0;

HRMS-ESI: *m/z* $[M+H]^+$ calcd for $C_{18}H_{26}BClO_2$: 321.1787, found 321.1785.

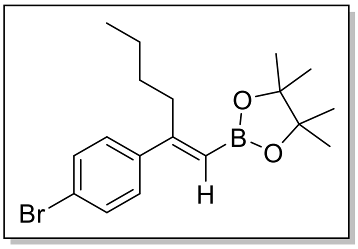

(*E*)-2-(2-(4-bromophenyl)hex-1-en-1-yl)-4,4,5,5-tetramethyl-1,3,2-dioxaborolane (**4g**) was isolated (148 mg, 0.41 mmol, 81%) as a yellow oil after chromatograph on silica with PE and EA (eluent: PE: EA = 50:1, R_f = 0.3);

^{1}H NMR (600 MHz, $CDCl_3$): δ 7.44−7.42 (m, 2H), 7.31−7.28 (m, 2H), 5.60 (s, 1H), 2.86 (t, *J* = 7.1 Hz, 2H), 1.33−1.29 (m, 16H), 0.87 (t, *J* = 7.1 Hz, 3H);

^{13}C NMR (151 MHz, $CDCl_3$): δ 162.1, 142.2, 131.2, 128.0, 121.7, 116.8, 82.9, 32.8, 31.7, 24.8, 22.3, 13.8;

^{11}B NMR (192 MHz, $CDCl_3$): δ 29.8;

HRMS-ESI: *m/z* $[M+Na]^+$ calcd for $C_{18}H_{26}BBrO_2$: 387.1101, found 387.1124.

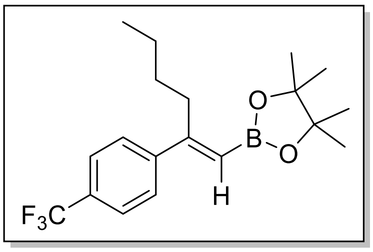

(*E*)-4,4,5,5-tetramethyl-2-(2-(4-(trifluoromethyl)phenyl)hex-1-en-1-yl)-1,3,2-dioxaborolane (**4h**) was isolated (138 mg, 0.39 mmol, 78%) as a light yellow oil after chromatograph on silica with PE and EA (eluent: PE: EA = 50:1, R_f = 0.3);

^{1}H NMR (600 MHz, $CDCl_3$): δ 7.58−7.54 (m, 2H), 7.52−7.49 (m, 2H), 5.64 (s, 1H), 2.89 (t, *J* = 7.1 Hz, 2H), 1.33−1.30 (m, 16H), 0.87 (t, *J* = 7.0 Hz, 3H);
^{13}C NMR (151 MHz, $CDCl_3$): δ 161.9, 147.0, 129.5 (q, *J* = 32.1 Hz), 127.4, 126.6, 125.1 (q, *J* = 3.8 Hz), 123.3, 118.2, 83.1, 33.0, 31.6, 24.8, 22.3, 13.8;
^{19}F NMR (565 MHz, $CDCl_3$): δ −62.5 (s);
^{11}B NMR (192 MHz, $CDCl_3$): δ 30.2;
HRMS-ESI: *m/z* $[M+Na]^+$ calcd for $C_{19}H_{26}BF_3O_2$: 377.1870, found 377.1885.

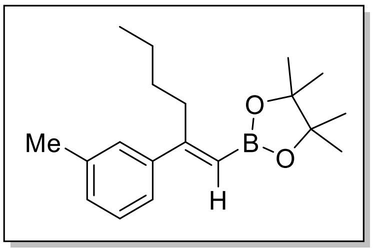

(*E*)-4,4,5,5-tetramethyl-2-(2-(*m*-tolyl)hex-1-en-1-yl)-1,3,2-dioxaborolane (**4i**) was isolated (128 mg, 0.43 mmol, 85%) as a colorless oil after chromatograph on silica with PE and EA (eluent: PE: EA = 50:1, R_f = 0.3);
^{1}H NMR (400 MHz, $CDCl_3$): δ 7.27−7.17 (m, 3H), 7.10−7.05 (m, 1H), 5.61 (s, 1H), 2.88 (t, *J* = 7.2 Hz, 2H), 2.34 (s, 3H), 1.38−1.28 (m, 16 H), 0.87 (t, *J* = 7.0 Hz, 3H);
^{13}C NMR (101 MHz, $CDCl_3$): δ 163.7, 143.4, 137.5, 128.4, 127.9, 127.2, 123.4, 115.8, 82.8, 33.0, 31.9, 24.8, 22.4, 21.4, 13.9;
^{11}B NMR (128 MHz, $CDCl_3$): δ 29.9;
HRMS-ESI: *m/z* $[M+H]^+$ calcd for $C_{19}H_{29}BO_2$: 300.2370, found 300.2358.

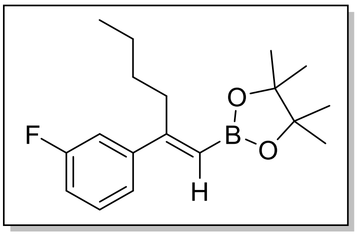

(*E*)-2-(2-(3-fluorophenyl)hex-1-en-1-yl)-4,4,5,5-tetramethyl-1,3,2-dioxaborolane (**4j**) was isolated (125 mg, 0.41 mmol, 82%) as a colorless oil after chromatograph on silica with PE and EA (eluent: PE: EA = 50:1, R_f = 0.3);
^{1}H NMR (400 MHz, $CDCl_3$): δ 7.29−7.23 (m, 1H), 7.22−7.19 (m, 1H), 7.15−7.10 (m, 1H), 6.99−6.92 (m, 1H), 5.63 (s, 1H), 2.86 (t, *J* = 7.2 Hz, 2H), 1.37−1.29 (m, 16H), 0.88 (t, *J* = 7.0 Hz, 3H);
^{13}C NMR (101 MHz, $CDCl_3$): δ 162.8 (d, *J* = 243.5 Hz), 162.0 (d, *J* = 2.0 Hz), 145.8 (d, *J* = 7.1 Hz), 129.5 (d, *J* = 8.3 Hz), 122.0 (d, *J* = 2.8 Hz), 114.4 (d, *J* = 21.1 Hz), 113.2 (d, *J* = 21.8 Hz), 83.0, 33.0, 31.8, 24.8, 22.4, 13.8;
^{19}F NMR (376 MHz, $CDCl_3$): δ −113.8 (s);
^{11}B NMR (128 MHz, $CDCl_3$): δ 29.6;
HRMS-ESI: *m/z* $[M+H]^+$ calcd for $C_{18}H_{26}BFO_2$: 304.2119, found 304.2110.

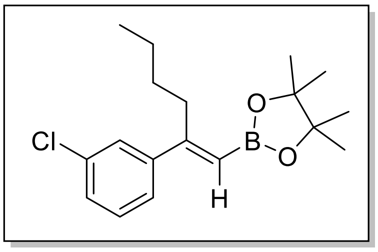

(*E*)-2-(2-(3-chlorophenyl)hex-1-en-1-yl)-4,4,5,5-tetramethyl-1,3,2-dioxaborolane (**4k**) was isolated (120 mg, 0.38 mmol, 75%) as a colorless oil after chromatograph on silica with PE and EA (eluent: PE: EA = 50:1, R_f = 0.3);
^{1}H NMR (600 MHz, $CDCl_3$): δ 7.41 (s, 1H), 7.31−7.28 (m, 1H), 7.25−7.22 (m, 2H), 5.61 (s, 1H), 2.86 (t, *J* = 7.0 Hz, 2H), 1.34−1.29 (m, 16H), 0.87 (t, *J* = 7.1 Hz, 3H);
^{13}C NMR (151 MHz, $CDCl_3$): δ 161.9, 145.3, 134.1, 129.4, 127.6, 126.6, 124.5, 117.4， 83.0, 32.9, 31.7, 24.8, 22.3, 13.8;
^{11}B NMR (192 MHz, $CDCl_3$): δ 30.0;
HRMS-ESI: *m/z* $[M+H]^+$ calcd for $C_{18}H_{26}BClO_2$: 320.1823, found 320.1829.

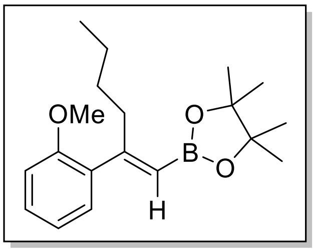

(*E*)-2-(2-(2-methoxyphenyl)hex-1-en-1-yl)-4,4,5,5-tetramethyl-1,3,2-dioxaborolane (**4l**) was isolated (122 mg, 0.39 mmol, 77%) as a colorless oil after chromatograph on silica with PE and EA (eluent: PE: EA = 50:1, R_f = 0.2);
^{1}H NMR (400 MHz, $CDCl_3$): δ 7.26−7.20 (m, 1H), 7.13−7.08 (m, 1H), 6.92−6.86 (m, 1H), 6.86−6.81 (m, 1H), 5.35 (s, 1H), 3.79 (s, 3H), 2.85 (t, *J* = 6.9 Hz, 2H), 1.29 (s, 12H), 1.27−1.22 (m, 4H), 0.84 (t, *J* = 7.1 Hz, 3H);
^{13}C NMR (101 MHz, $CDCl_3$): δ 164.7, 155.9, 134.6, 129.9, 128.3, 120.3, 118.7, 110.5, 82.7, 55.3, 34.0, 31.6, 24.8, 2.4, 13.9;
^{11}B NMR (128 MHz, $CDCl_3$): δ 29.8;
HRMS-ESI: *m/z* $[M+H]^+$ calcd for $C_{19}H_{29}BO_3$: 316.2319, found 316.2299.

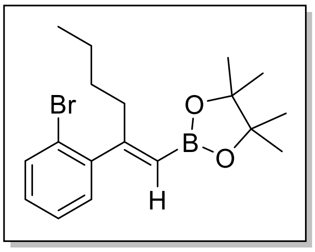

(*E*)-2-(2-(2-bromophenyl)hex-1-en-1-yl)-4,4,5,5-tetramethyl-1,3,2-dioxaborolane (**4m**) was isolated (133 mg, 0.37 mmol, 73%) as a colorless oil after chromatograph on silica with PE and EA (eluent: PE: EA = 50:1, R_f = 0.3);
^{1}H NMR (400 MHz, $CDCl_3$): δ 7.55−7.51 (m, 1H), 7.26−7.21 (m, 1H), 7.13−7.07 (m, 2H), 5.28 (s, 1H), 2.81 (t, *J* = 7.3 Hz, 2H), 1.34−1.28 (m, 16H), 0.86 (t, *J* = 7.1 Hz, 3H);
^{13}C NMR (101 MHz, $CDCl_3$): δ 164.5, 146.2, 132.6, 129.7, 128.1, 126.8, 121.0, 119.6, 82.9, 35.0, 31.2, 24.8, 22.5, 13.9;
^{11}B NMR (128 MHz, $CDCl_3$): δ 29.8;
HRMS-ESI: *m/z* $[M+Na]^+$ calcd for $C_{18}H_{26}BBrO_2$: 386.1138, found 386.1116.

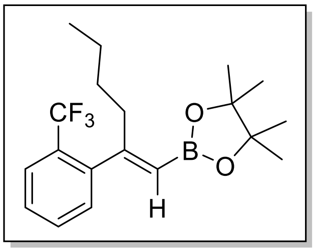

(*E*)-4,4,5,5-tetramethyl-2-(2-(2-(trifluoromethyl)phenyl)hex-1-en-1-yl)-1,3,2-dioxaborolane (**4n**) was isolated (120 mg, 0.34 mmol, 68%) as a colorless oil after chromatograph on silica with PE and EA (eluent: PE: EA = 50:1, R_f = 0.3);
^{1}H NMR (600 MHz, $CDCl_3$): δ 7.62 (d, *J* = 7.9 Hz, 1H), 7.45 (t, *J* = 7.5 Hz, 1H), 7.34 (t, *J* = 7.7 Hz, 1H), 7.17 (d, *J* = 7.7 Hz, 1H), 5.25 (s, 1H), 2.73 (t, *J* = 7.1 Hz, 2H), 1.31−1.28 (m, 16H), 0.86 (t, *J* = 6.7 Hz, 3H);
^{13}C NMR (151 MHz, $CDCl_3$): δ 163.0, 144.6, 131.0, 129.8, 127.0 (q, *J* = 29.7 Hz), 126.8, 126.1, (q, *J* = 5.1 Hz), 125.2, 123.3, 119.5, 82.9, 36.1, 31.3, 24.82, 24.8, 13.8;
^{19}F NMR (565 MHz, $CDCl_3$): δ -57.2 (s);
^{11}B NMR (192 MHz, $CDCl_3$): δ 29.9;
HRMS-ESI: *m/z* $[M+H]^+$ calcd for $C_{19}H_{26}BF_3O_2$: 354.2087, found 354.2111.

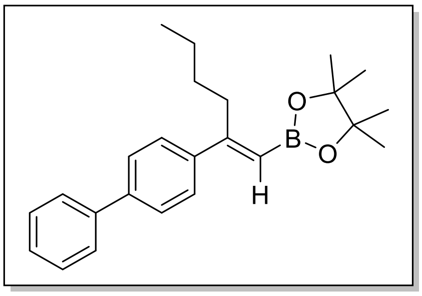

(*E*)-2-(2-([1,1'-biphenyl]-4-yl)hex-1-en-1-yl)-4,4,5,5-tetramethyl-1,3,2-dioxaborolane (**4o**) was isolated (149 mg, 0.41 mmol, 82%) as a colorless oil after chromatograph on silica with PE and EA (eluent: PE: EA = 50:1, R_f = 0.3);

^{1}H NMR (400 MHz, $CDCl_3$): δ 7.63−7.60 (m, 2H), 7.58−7.52 (m, 4H), 7.46−7.42 (m, 2H), 7.36−7.32 (m, 1H), 5.71 (s, 1H), 2.94 (t, *J* = 7.1 Hz, 2H), 1.44−1.36 (m, 4H), 1.32 (s, 12H), 0.90 (t, *J* = 7.1 Hz, 3H);
^{13}C NMR (101 MHz, $CDCl_3$): δ 162.9, 142.1, 140.7, 140.4, 128.7, 127.2, 127.0, 126.82, 126.77, 82.9, 32.9, 32.1, 24.8, 22.5, 13.9;
^{11}B NMR (128 MHz, $CDCl_3$): δ 30.7;
HRMS-ESI: *m/z* $[M+H]^+$ calcd for $C_{24}H_{31}BO_2$: 362.2526, found 362.2515.

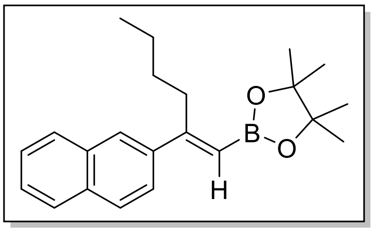

(*E*)-4,4,5,5-tetramethyl-2-(2-(naphthalen-2-yl)hex-1-en-1-yl)-1,3,2-dioxaborolane (**4p**) was isolated (145 mg, 0.43 mmol, 86%) as a colorless oil after chromatograph on silica with PE and EA (eluent: PE: EA = 50:1, R_f = 0.3);
^{1}H NMR (400 MHz, $CDCl_3$): δ 7.89 (s, 1H), 7.82−7.75 (m, 3H), 7.62−7.57 (m, 1H), 7.46−7.40 (m, 2H), 5.79 (s, 1H), 3.02 (t, *J* = 7.2 Hz, 2H), 1.44−1.34 (m, 4H), 1.30 (s, 12H), 0.88 (t, *J* = 7.2 Hz, 3H);
^{13}C NMR (101 MHz, $CDCl_3$): δ 163.3, 140.6, 133.2, 132.9, 128.3, 127.6, 127.4, 126.0, 125.9, 125.3, 124.8, 116.7, 82.8, 32.9, 32.0, 24.8, 22.4, 13.9;
^{11}B NMR (128 MHz, $CDCl_3$): δ 30.2;
HRMS-ESI: *m/z* $[M+H]^+$ calcd for $C_{22}H_{29}BO_2$: 336.2370, found 336.2361.

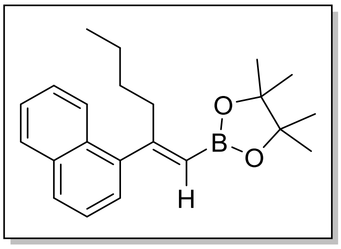

(*E*)-4,4,5,5-tetramethyl-2-(2-(naphthalen-1-yl)hex-1-en-1-yl)-1,3,2-dioxaborolane (**4q**) was isolated (136 mg, 0.41 mmol, 81%) as a colorless oil after chromatograph on silica with PE and EA (eluent: PE: EA = 50:1, R_f = 0.3);
^{1}H NMR (400 MHz, $CDCl_3$): δ 8.06−8.02 (m, 1H), 7.86−7.83 (m, 1H), 7.76 (d, *J* = 8.2 Hz, 1H), 7.48−7.42 (m, 3H), 7.27 (dd, J_1 = 1.0 Hz, J_2 = 7.0 Hz, 1H), 5.47 (s, 1H), 2.93 (t, *J* = 7.3 Hz, 2H), 1.37−1.31 (m, 16H), 0.85 (t, *J* = 7.2 Hz, 3H);
^{13}C NMR (101 MHz, $CDCl_3$): δ 164.3, 143.8, 133.6, 130.7, 128.1, 127.0, 126.1, 125.6, 125.5, 125.0, 124.2, 82.9, 36.7, 31.6, 24.9, 22.6, 13.9;
^{11}B NMR (128 MHz, $CDCl_3$): δ 30.1;
HRMS-ESI: *m/z* $[M+H]^+$ calcd for $C_{22}H_{29}BO_2$: 336.2370, found 336.2362.

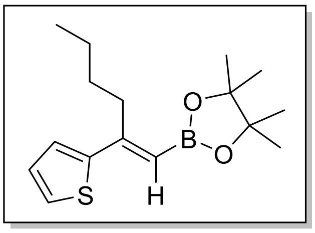

(*E*)-4,4,5,5-tetramethyl-2-(2-(thiophen-2-yl)hex-1-en-1-yl)-1,3,2-dioxaborolane (**4r**) was isolated (126 mg, 0.43 mmol, 86%) as a colorless oil after chromatograph on silica with PE and EA (eluent: PE: EA = 50:1, R_f = 0.3);
^{1}H NMR (400 MHz, $CDCl_3$): δ 7.22−7.19 (m, 1H), 7.15−7.13 (m, 1H), 6.98 (dd, J_1 = 5.0 Hz, J_2 = 3.7 Hz, 1H), 5.77 (s, 1H), 2.84 (t, *J* = 7.6, 2H); 1.55−1.50 (m, 2H), 1.42−1.36 (m, 2H), 1.29 (s, 12H), 0.92 (t, *J* = 7.3 Hz, 3H);
^{13}C NMR (101 MHz, $CDCl_3$): δ 155.8, 147.7, 127.6, 125.6, 124.6, 82.9, 33.5, 32.9, 24.8, 22.7, 14.0;
^{11}B NMR (128 MHz, $CDCl_3$): δ 29.9;
HRMS-ESI: *m/z* $[M+H]^+$ calcd for $C_{16}H_{25}BO_2S$: 292.1777, found 292.1771.

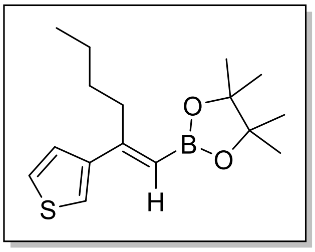

(*E*)-4,4,5,5-tetramethyl-2-(2-(thiophen-3-yl)hex-1-en-1-yl)-1,3,2-dioxaborolane (**4s**) was isolated (117 mg, 0.40 mmol, 80%) as a colorless oil after chromatograph on silica with PE and EA (eluent: PE: EA = 50:1, R_f = 0.3);
^{1}H NMR (400 MHz, $CDCl_3$): δ 7.33−7.30 (m, 1H), 7.29−7.26 (m, 1H), 7.25−7.22 (m, 1H), 5.72 (s, 1H), 2.83 (t, *J* = 7.5 Hz, 2H), 1.51−1.44 (m, 2H), 1.39−1.34 (m, 2H), 1.29 (s, 12H), 0.9 (t, *J* = 7.2 Hz, 3H);
^{13}C NMR (101 MHz, $CDCl_3$): δ 157.2, 144.6, 126.2, 125.3, 121.8, 82.8, 33.3, 32.6, 24.8, 22.7, 13.9;
^{11}B NMR (128 MHz, $CDCl_3$): δ 30.3;
HRMS-ESI: *m/z* $[M+H]^+$ calcd for $C_{16}H_{25}BO_2S$: 292.1777, found 292.1769.

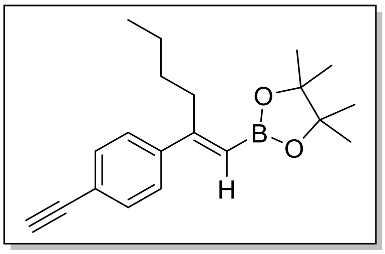

(*E*)-2-(2-(4-ethynylphenyl)hex-1-en-1-yl)-4,4,5,5-tetramethyl-1,3,2-dioxaborolane (**4t**) was isolated (98 mg, 0.31 mmol, 63%) as a colorless oil after chromatograph on silica with PE and EA (eluent: PE: EA = 50:1, R_f = 0.3);
^{1}H NMR (400 MHz, $CDCl_3$): δ 7.46−7.42 (m, 2H), 7.41−7.37 (m, 2H), 5.63 (s, 1H), 3.09 (s, 1H), 2.87 (t, *J* = 7.0 Hz, 2H), 1.31−1.29 (m, 16H), 0.87 (t, *J* = 7.0 Hz, 3H);
^{13}C NMR (101 MHz, $CDCl_3$): δ 162.4, 143.8, 132.0, 126.3, 121.3, 83.6, 83.0, 77.6, 32.8, 31.8, 24.8, 22.4, 13.8;
^{11}B NMR (128 MHz, $CDCl_3$): δ 29.7;
HRMS-ESI: *m/z* $[M+H]^+$ calcd for $C_{20}H_{27}BO_2$: 310.2213, found 310.2203.

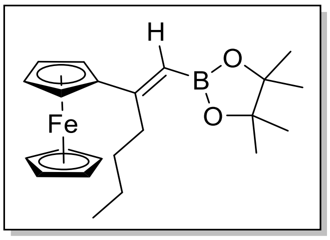

(*E*)-(1-butyl-2-(4,4,5,5-tetramethyl-1,3,2-dioxaborolan-2-yl)ethenyl)-ferrocene (**4u**) was isolated (154 mg, 0.39 mmol, 78%) as a colorless oil after chromatograph on silica with PE and EA (eluent: PE: EA = 50:1, R_f = 0.3);
^{1}H NMR (600 MHz, $CDCl_3$): δ 5.48 (s, 1H), 4.50 (t, *J* = 1.8 Hz, 2H), 4.31 (t, *J* = 1.8 Hz, 2H), 4.07 (s, 5H), 2.64 (t, *J* = 7.7 Hz, 2H), 1.50−1.45 (m, 2H), 1.39−1.34 (m, 2H), 1.22 (s, 12H), 0.92 (t, *J* = 7.3 Hz, 3H);
^{13}C NMR (151 MHz, $CDCl_3$): δ 162.7, 111.1, 86.4, 82.7, 69.89, 69.86, 67.0, 34.2, 33.3, 25.1, 22.9, 14.3;
^{11}B NMR (192 MHz, $CDCl_3$): δ 31.4;
HRMS-ESI: *m/z* $[M+H]^+$ calcd for $C_{22}H_{31}BFeO_2$: 382.1793, found 382.1755.

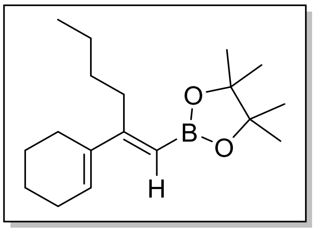

(*E*)-2-(2-(cyclohex-1-en-1-yl)hex-1-en-1-yl)-4,4,5,5-tetramethyl-1,3,2-dioxaborolane (**4v**) was isolated (138 mg, 0.48 mmol, 92%) as a colorless oil after chromatograph on silica with PE and EA (eluent: PE: EA = 50:1, R_f = 0.3);
^{1}H NMR (600 MHz, $CDCl_3$): δ 6.04 (t, *J* = 3.6 Hz, 1H), 5.29 (s, 1H), 2.60 (t, *J* = 7.3 Hz, 2H), 2.17−2.12 (m, 4H), 1.65−1.61 (m, 2H), 1.56−1.52 (m, 2H), 1.39−1.31 (m, 4H), 1.24 (s, 12H), 0.89 (t, *J* = 7.2 Hz, 3H);
^{13}C NMR (151 MHz, $CDCl_3$): δ 163.7, 137.5, 126.7, 110.7, 82.5, 33.4, 31.0, 26.1, 24.7, 23.0, 22.8, 22.0, 13.9;
^{11}B NMR (192 MHz, $CDCl_3$): δ 30.3;
HRMS-ESI: *m/z* $[M+H]^+$ calcd for $C_{18}H_{31}BO_2$: 290.2526, found 290.2521.

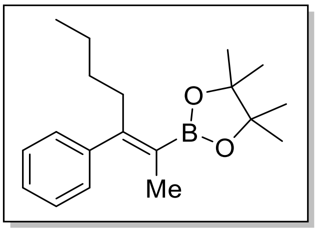

(*Z*)-4,4,5,5-tetramethyl-2-(3-phenylhept-2-en-2-yl)-1,3,2-dioxaborolane (**4w**) was isolated (132 mg, 0.44 mmol, 88%) as a colorless oil after chromatograph on silica with PE and EA (eluent: PE: EA = 50:1, R_f = 0.3);
^{1}H NMR (400 MHz, $CDCl_3$): δ 7.33−7.28 (m, 2H), 7.23−7.17 (m, 1H), 7.10−7.05 (m, 2H), 2.63 (t, *J* = 7.0 Hz, 2H), 1.56 (s, 3H), 1.31 (s, 12H), 1.28−1.23 (m, 4H), 0.84 (t, *J* = 7.0 Hz, 3H);
^{13}C NMR (101 MHz, $CDCl_3$): δ 155.2, 143.4, 127.9, 127.8, 126.1, 122.4, 82.9, 37.6, 31.2, 24.8, 22.5, 18.1, 13.9;
^{11}B NMR (128 MHz, $CDCl_3$): δ 31.0;
HRMS-ESI: *m*/*z* $[M+H]^+$ calcd for $C_{19}H_{29}BO_2$: 300.2370, found 300.2358.

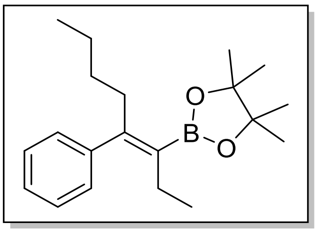

(*Z*)-4,4,5,5-tetramethyl-2-(4-phenyloct-3-en-3-yl)-1,3,2-dioxaborolane (**4x**) was isolated (132 mg, 0.42 mmol, 84%) as a colorless oil after chromatograph on silica with PE and EA (eluent: PE: EA = 50:1, R_f = 0.3);
^{1}H NMR (400 MHz, $CDCl_3$): δ 7.32−7.27 (m, 2H), 7.23−7.18 (m, 1H), 7.09−7.05 (m, 2H), 2.55 (t, *J* = 7.4 Hz, 2H), 1.95 (q, *J* = 7.5 Hz, 2H), 1.32 (s, 12H), 1.28−1.24 (m, 4H), 0.89−0.82 (m, 6H);
^{13}C NMR (101 MHz, $CDCl_3$): δ 153.2, 143.3, 127.9, 127.7, 126.0, 83.0, 38.1, 31.1, 25.4, 24.8, 22.6, 15.0, 13.9;
^{11}B NMR (128 MHz, $CDCl_3$): δ 31.3;
HRMS-ESI: *m*/*z* $[M+H]^+$ calcd for $C_{19}H_{29}BO_2$: 314.2526, found 314.2520.

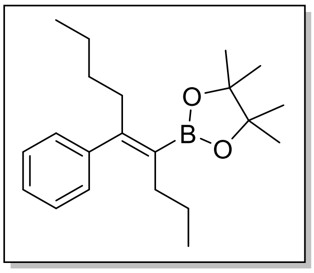

(*Z*)-4,4,5,5-tetramethyl-2-(5-phenylnon-4-en-4-yl)-1,3,2-dioxaborolane (**4y**) was isolated (133 mg, 0.41 mmol, 81%) as a colorless oil after chromatograph on silica with PE and EA (eluent: PE: EA = 50:1, R_f = 0.3);
^{1}H NMR (600 MHz, $CDCl_3$): δ 7.31−7.27 (m, 2H), 7.22−7.18 (m, 1H), 7.08−7.04 (m, 2H), 2.55 (t, *J* = 7.3 Hz, 2H), 1.92 (t, *J* = 7.6 Hz, 2H), 1.32 (s, 12H), 1.28−1.25 (m, 4H), 0.83 (t, *J* = 6.8 Hz, 3H), 0.74 (t, *J* = 7.3 Hz, 3H);
^{13}C NMR (151 MHz, $CDCl_3$): δ 153.3, 143.4, 128.0, 127.7, 125.9, 83.0, 38.2, 34.3, 31.1, 24.8, 23.5, 22.6, 14.1, 13.9;
^{11}B NMR (192 MHz, $CDCl_3$): δ 31.5;
HRMS-ESI: *m*/*z* $[M+Na]^+$ calcd for $C_{21}H_{33}BO_2$: 350.2502, found 350.2489.

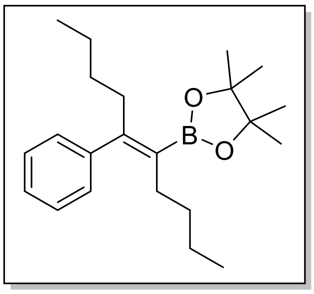

(*Z*)-4,4,5,5-tetramethyl-2-(6-phenyldec-5-en-5-yl)-1,3,2-dioxaborolane (**4z**) was isolated (127 mg, 0.37 mmol, 74%) as a colorless oil after chromatograph on silica with PE and EA (eluent: PE: EA = 50:1, R_f = 0.3);
^{1}H NMR (600 MHz, $CDCl_3$): δ 7.32−7.26 (m, 2H), 7.23−7.18 (m, 1H), 7.09−7.03 (m, 2H), 2.54 (t, *J* = 7.3 Hz, 2H), 1.93 (t, *J* = 7.6 Hz, 2H), 1.33 (s, 12H), 1.26−1.23 (m, 6H), 1.17−1.10 (m, 2H), 0.83 (t, *J* = 6.7 Hz, 3H), 0.74 (t, *J* = 7.3 Hz, 3H);
^{13}C NMR (151 MHz, $CDCl_3$): δ 153.0, 143.3, 128.0, 127.7, 126.0, 83.0, 38.2, 32.6, 31.8, 31.1, 24.8, 22.6, 22.5, 13.94, 13.89;
^{11}B NMR (192 MHz, $CDCl_3$): δ 31.6;
HRMS-ESI: *m*/*z* $[M+Na]^+$ calcd for $C_{22}H_{35}BO_2$: 364.2659, found 364.2632.

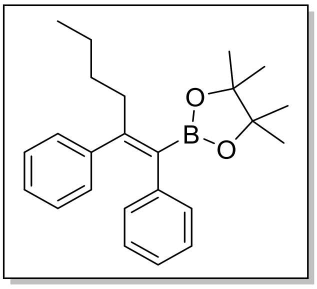

(*Z*)-2-(1,2-diphenylhex-1-en-1-yl)-4,4,5,5-tetramethyl-1,3,2-dioxaborolane (**4aa**) was isolated (154 mg, 0.43 mmol, 85%) as a colorless oil after chromatograph on silica with PE and EA (eluent: PE: EA = 50:1, R_f = 0.3);
^{1}H NMR (400 MHz, $CDCl_3$): δ 7.11−6.99 (m, 5H), 6.99−6.89 (m, 5H), 2.70 (t, *J* = 7.2 Hz, 2H), 1.40−1.27 (m, 16H), 0.86 (t, *J* = 7.0 Hz, 3H);
^{13}C NMR (101 MHz, $CDCl_3$): δ 153.4, 142.2, 141.5, 129.5, 129.0, 127.5, 127.3, 126.1, 125.0, 83.5, 38.6, 31.1, 24.7, 22.7, 13.9;
^{11}B NMR (128 MHz, $CDCl_3$): δ 31.3;
HRMS-ESI: *m/z* $[M+H]^+$ calcd for $C_{24}H_{31}BO_2$: 362.2526, found 362.2527.

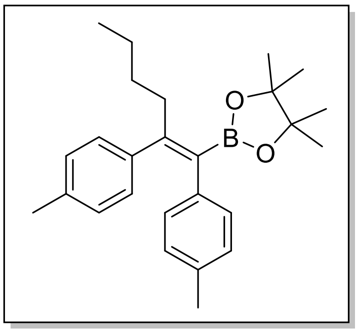

(*Z*)-2-(1,2-di-p-tolylhex-1-en-1-yl)-4,4,5,5-tetramethyl-1,3,2-dioxaborolane (**4ab**) was isolated (162 mg, 0.42 mmol, 83%) as a colorless oil after chromatograph on silica with PE and EA (eluent: PE: EA = 50:1, R_f = 0.3);
^{1}H NMR (400 MHz, $CDCl_3$): δ 6.92−6.80 (m, 8H), 2.46 (t, *J* = 7.2 Hz, 2H), 2.22 (s, 3H), 2.18 (s, 3H), 1.37−1.29 (m, 16H), 0.85 (t, *J* = 7.0 Hz, 3H);
^{13}C NMR (101 MHz, $CDCl_3$): δ 152.5, 139.2, 138.5, 135.5, 134.3, 129.3, 128.9, 128.3, 128.2, 83.4, 38.8, 31.2, 24.7, 22.7, 21.1, 21.0, 13.9;
^{11}B NMR (128 MHz, $CDCl_3$): δ 30.3;
HRMS-ESI: *m/z* $[M+Na]^+$ calcd for $C_{26}H_{35}BO_2$: 412.2659, found 412.2663.

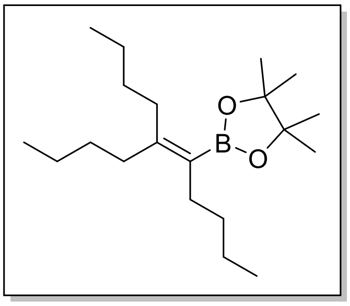

2-(6-Butyldec-5-en-5-yl)-4,4,5,5-tetramethyl-1,3,2-dioxaborolane (**4ac**) was isolated (145 mg, 0.45 mmol, 90%) as a colorless oil after chromatograph on silica with PE and EA (eluent: PE: EA = 100:1, R_f = 0.3);
^{1}H NMR (400 MHz, $CDCl_3$): δ 2.22 (t, *J* = 7.1 Hz, 2H), 2.12−2.01 (m, 4H), 1.37−1.24 (m, 24H), 0.93−0.85 (m, 9H);
^{13}C NMR (101 MHz, $CDCl_3$): δ 153.8, 82.6, 35.8, 33.0, 32.5, 31.7, 31.1, 30.4, 24.8, 23.2, 23.0, 22.9, 14.12, 14.09, 14.0;
^{11}B NMR (128 MHz, $CDCl_3$): δ 31.0;
HRMS-ESI: *m/z* $[M+Na]^+$ calcd for $C_{20}H_{39}BO_2$: 344.2972, found 344.2971.

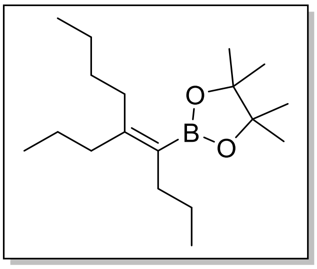

(*E*)-4,4,5,5-tetramethyl-2-(5-propylnon-4-en-4-yl)-1,3,2-dioxaborolane (**4ad**) was isolated (135 mg, 0.46 mmol, 92%) as a colorless oil after chromatograph on silica with PE and EA (eluent: PE: EA = 100:1, R_f = 0.3);

^{1}H NMR (400 MHz, $CDCl_3$): δ 2.23 (t, *J* = 7.1 Hz, 2H), 2.11−1.99 (m, 4H), 1.40−1.25 (m, 20H), 0.93−0.86 (m, 9H);

^{13}C NMR (101 MHz, $CDCl_3$): δ 153.9, 82.6, 35.8, 34.2, 32.9, 32.5, 24.8, 23.8, 23.0, 22.1, 14.6, 14.3, 14.1;

^{11}B NMR (128 MHz, $CDCl_3$): δ 31.1;

HRMS-ESI: *m/z* $[M+Na]^+$ calcd for $C_{18}H_{35}BO_2$: 316.2659, found 316.2659.

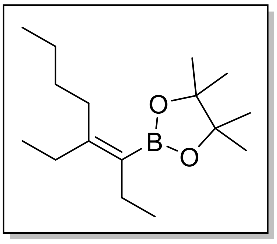

(*E*)-2-(4-ethyloct-3-en-3-yl)-4,4,5,5-tetramethyl-1,3,2-dioxaborolane (**4ae**) was isolated (116 mg, 0.44 mg, 87%) as a colorless oil after chromatograph on silica with PE and EA (eluent: PE: EA = 100:1, R_f = 0.3);

^{1}H NMR (400 MHz, $CDCl_3$): δ 2.25 (t, *J* = 7.1 Hz, 2H), 2.16−2.05 (m, 4H), 1.38−1.29 (m, 4H), 1.27 (s, 12H), 0.99−0.87 (m, 9H);

^{13}C NMR (101 MHz, $CDCl_3$): δ 155.3, 82.6, 35.3, 32.4, 24.8, 24.7, 23.7, 23.0, 15.2, 14.1, 13.5;

^{11}B NMR (128 MHz, $CDCl_3$): δ 31.1;

HRMS-ESI: *m/z* $[M+H]^+$ calcd for $C_{16}H_{31}BO_2$: 266.2526, found 266.2516.

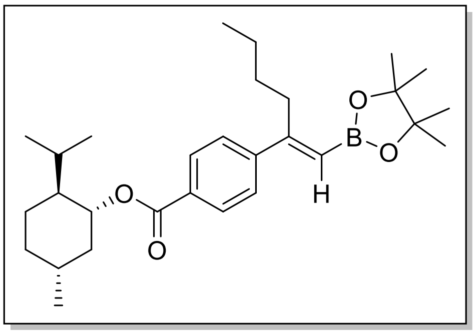

(1*R*,2*S*,5*R*)-2-isopropyl-5-methylcyclohexyl 4-((*E*)-1-(4,4,5,5-tetramethyl-1,3,2-dioxaborolan-2-yl)hex-1-en-2-yl)benzoate (**4af**) was isolated (161 mg, 0.33 mmol, 66%) as a colorless oil after chromatograph on silica with PE and EA (eluent: PE: EA = 50:1, R_f = 0.2);

^{1}H NMR (600 MHz, $CDCl_3$): δ 8.00−7.95 (m, 2H), 7.50−7.45 (m, 2H), 5.67 (s, 1H), 4.95−4.90 (m, 1H), 2.90 (t, *J* = 7.1 Hz, 2H), 2.14−2.09 (m, 1H), 2.00−1.94 (m, 1H), 1.75−1.71 (m, 2H), 1.58−1.52 (m, 2H), 1.33−1.25 (m, 17H), 1.14−1.06 (m, 2H), 0.93−0.91 (m, 6H), 0.86 (t, *J* = 7.1 Hz, 3H), 0.79 (d, *J* = 7.0 Hz, 3H);

^{13}C NMR (151 MHz, $CDCl_3$): δ 165.9, 162.4, 147.8, 129.9, 129.5, 126.3, 118.0, 83.0, 74.7, 47.2, 40.9, 34.3, 32.9, 31.7, 31.4, 26.4, 24.8, 23.5, 22.3, 22.0, 20.8, 16.4, 13.8;

^{11}B NMR (192 MHz, $CDCl_3$): δ 29.2;

HRMS-ESI: *m/z* $[M+Na]^+$ calcd for $C_{29}H_{45}BO_4$: 490.3339, found 490.3339.

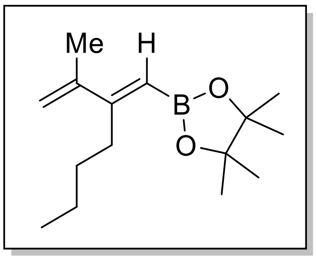

(*E*)-4,4,5,5-Tetramethyl-2-(2-(prop-1-en-2-yl)hex-1-en-1-yl)-1,3,2-dioxaborolane (**4ag**) was isolated (108 mg, 0.43 mmol, 86%) as a colorless oil after chromatograph on silica with PE and EA (eluent: PE: EA = 50:1, R_f = 0.3);

^{1}H NMR (500 MHz, $CDCl_3$): δ 5.41 (s, 1H), 5.20 (s, 1H), 5.05 (s, 1H), 2.62 (t, *J* = 8.0 Hz, 2H), 1.88 (s, 3H), 1.41−1.36 (m, 2H), 1.34−1.30 (m, 2H), 1.26 (s, 12H), 0.89 (t, *J* = 7.3 Hz, 3H);

^{13}C NMR (125 MHz, $CDCl_3$): δ 162.5, 144.4, 114.5, 82.7, 33.1, 31.2, 24.7, 22.8, 21.3, 13.9;

^{11}B NMR (192 MHz, $CDCl_3$): δ 30.25;

HRMS-ESI: *m/z* $[M+Na]^+$ calcd for $C_{15}H_{27}BO_2$: 273.1996, found 273.1998.

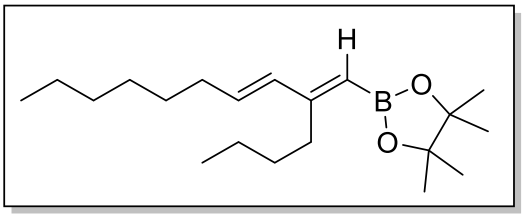

2-((1*E*,3*E*)-2-Butyldeca-1,3-dien-1-yl)-4,4,5,5-tetramethyl-1,3,2-dioxaborolane (**4ah**) was isolated (146 mg, 0.46 mmol, 91%) as a colorless oil after chromatograph on silica with PE and EA (eluent: PE: EA = 50:1, R_f = 0.25);
^{1}H NMR (400 MHz, $CDCl_3$): δ 6.00 (d, *J* = 15.76 Hz, 1H), 5.90−5.81 (m, 1H), 5.18 (s, 1H), 2.56 (t, *J* = 7.16 Hz, 2H), 2.10 (q, *J* = 6.96 Hz, 2H), 1.32−1.23 (m, 24H), 0.93−0.87 (m, 6H);
^{13}C NMR (101 MHz, $CDCl_3$): δ 161.2, 134.9, 133.1, 116.4, 82.5, 32.88, 32.87, 31.7, 30.2, 29.2, 28.8, 24.8, 22.8, 22.6, 14.03, 13.96;
^{11}B NMR (128 MHz, $CDCl_3$): δ 29.58;
HRMS-ESI: *m/z* $[M+Na]^+$ calcd for $C_{15}H_{27}BO_2$: 320.2996, found 320.3007.

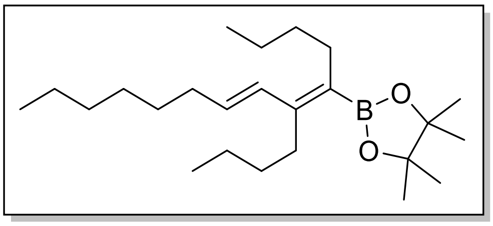

2-((5*Z*,7*E*)-6-Butyltetradeca-5,7-dien-5-yl)-4,4,5,5-tetramethyl-1,3,2-dioxaborolane (**4ai**) was isolated (151 mg, 0.4 mmol, 80%) as a colorless oil after chromatograph on silica with PE and EA (eluent: PE: EA = 50:1, R_f = 0.25);
^{1}H NMR (600 MHz, $CDCl_3$): δ 6.39 (d, *J* = 15.66 Hz, 1H), 5.83−5.75 (m, 1H), 2.46 (t, *J* = 7.56 Hz, 2H), 2.23 (t, *J* = 7.44 Hz, 2H), 2.15−2.11 (m, 2H), 1.41−1.35 (m, 4H), 1.32−1.26 (m, 24H), 0.91−0.87 (m, 9H);
^{13}C NMR (151 MHz, $CDCl_3$): δ 148.6, 132.1, 127.4, 82.7, 33.4, 33.3, 33.0, 32.3, 31.7, 29.8, 29.4, 28.8, 24.8, 23.1, 22.7, 22.6, 14.10, 14.07;
^{11}B NMR (192 MHz, $CDCl_3$): δ 31.66;
HRMS-ESI: *m/z* $[M+Na]^+$ calcd for $C_{24}H_{37}BO_2$: 399.3405, found 399.3422.

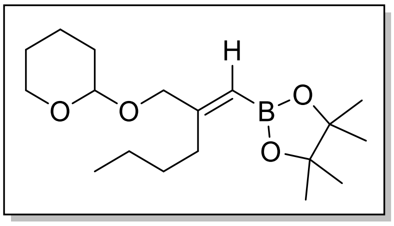

(*E*)-4,4,5,5-Tetramethyl-2-(2-(((tetrahydro-2*H*-pyran-2-yl)oxy)methyl)hex-1-en-1-yl)-1,3,2-dioxaborolane (**4aj**) was isolated (133 mg, 0.41 mmol, 82%) as a colorless oil after chromatograph on silica with PE and EA (eluent: PE: EA = 20:1, R_f = 0.2);
^{1}H NMR (500 MHz, $CDCl_3$): δ 5.45 (t, *J* = 1.5 Hz, 1H), 4.61 (t, *J* = 3.35 Hz, 1H), 4.19 (dd, *J* = 1.6 Hz, 14.95 Hz, 1H), 3.91 (dd, *J* = 1.4 Hz, 14.85 Hz, 1H), 3.85−3.78 (m, 1H), 3.50−3.44 (m, 1H), 2.42−2.28 (m, 2H), 1.86−1.81 (m, 1H), 1.72−1.65 (m, 1H), 1.65−1.53 (m, 2H), 1.52−1.46 (m, 2H), 1.41−1.34 (m, 2H), 1.32−1.27 (m, 2H), 1.23 (s, 12H), 0.87 (t, J = 7.2 Hz, 3H);
^{13}C NMR (125 MHz, $CDCl_3$): δ 162.2, 111.8, 97.6, 82.5, 70.3, 61.7, 31.8, 31.7, 30.4, 25.4, 24.72, 24.68, 22.6, 19.0, 13.8;
^{11}B NMR (192 MHz, $CDCl_3$): δ 30.85;
HRMS-ESI: *m/z* $[M+Na]^+$ calcd for $C_{18}H_{33}BO_4$: 347.2364, found 347.2375.

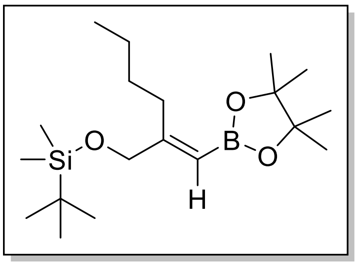

(*E*)-tert-butyldimethyl((2-((4,4,5,5-tetramethyl-1,3,2-dioxaborolan-2-yl)methylene)hexyl)oxy)silane (**4ak**) was isolated (136 mg, 0.39 mmol, 77%) as a colorless oil after chromatograph on silica with PE and EA (eluent: PE: EA = 50:1, R_f = 0.25);
^{1}H NMR (400 MHz, $CDCl_3$): δ 5.47 (s, 1H), 4.10 (s, 2H), 2.32 (t, *J* = 7.20 Hz, 2H), 1.39−1.30 (m, 4H), 1.25 (s, 12H), 0.92−0.88 (m, 12H), 0.05 (s, 6H);
^{13}C NMR (101 MHz, $CDCl_3$): δ 164.8, 82.5, 66.8, 32.3, 31.4, 26.0, 24.8, 18.4, 13.9, −5.4;
^{11}B NMR (128 MHz, $CDCl_3$): δ 30.44;
HRMS-ESI: *m/z* $[M+H]^+$ calcd for $C_{19}H_{39}BO_3Si$: 355.2834, found 355.2812.

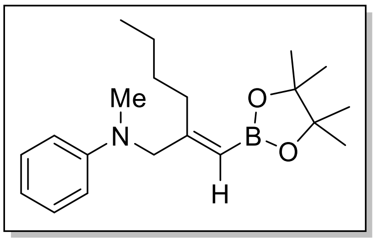

(*E*)-*N*-methyl-*N*-(2-((4,4,5,5-tetramethyl-1,3,2-dioxaborolan-2-yl)methylene)hexyl)aniline (**4al**) was isolated (123 mg, 0.38 mmol, 75%) as a colorless oil after chromatograph on silica with PE and EA (eluent: PE: EA = 30:1, R_f = 0.3);
^{1}H NMR (400 MHz, $CDCl_3$): δ 7.22−7.15 (m, 2H), 6.65 (t, *J* = 7.24 Hz, 1H), 6.60 (d, *J* = 8.16 Hz, 2H), 5.21 (t, *J* = 1.6 Hz, 1H), 3.87 (d, *J* = 1.28 Hz, 2H), 2.94 (s, 3H), 2.40 (t, *J* = 7.48 Hz, 2H), 1.52−1.44 (m, 2H), 1.41−1.35 (m, 2H), 1.25 (s, 12H), 0.95 (t, *J* = 7.24 Hz, 2H);
^{13}C NMR (101 MHz, $CDCl_3$): δ 161.1, 149.4, 129.0, 115.8, 111.6, 82.6, 59.6, 38.4, 32.4, 32.1, 24.8, 22.8, 13.9;
^{11}B NMR (128 MHz, $CDCl_3$): δ 29.72;
HRMS-ESI: *m/z* $[M+Na]^+$ calcd for $C_{20}H_{32}BNO_2$: 329.2635, found 329.2627.

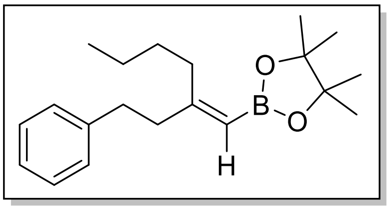

(*E*)-4,4,5,5-Tetramethyl-2-(2-phenethylhex-1-en-1-yl)-1,3,2-dioxaborolane (**4am**) was isolated (137 mg, 0.44 mmol, 87%) as a colorless oil after chromatograph on silica with PE and EA (eluent: PE: EA = 50:1, R_f = 0.3);
^{1}H NMR (400 MHz, $CDCl_3$): δ 7.30−7.23 (m, 2H), 7.21−7.15 (m,2H), 1.37−1.31 (m, 2H), 1.30−1.22 (m, 14H), 0.9 (t, *J* = 7.16 Hz, 3H);
^{13}C NMR (101 MHz, $CDCl_3$): δ 166.6, 142.2, 128.3, 128.2, 125.7, 112.7, 82.5, 40.9, 34.7, 34.4, 31.7, 24.8, 22.6, 13.9;
^{11}B NMR (128 MHz, $CDCl_3$): δ 29.53;
HRMS−ESI: *m/z* $[M+Na]^+$ calcd for $C_{20}H_{31}BO_2$: 337.2309, found 337.2324.

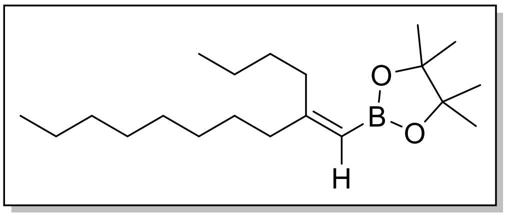

(*E*)-2-(2-butyldec-1-en-1-yl)-4,4,5,5-tetramethyl-1,3,2-dioxaborolane (**4an**) was isolated (135 mg, 0.42 mmol, 84%) as a colorless oil after chromatograph on silica with PE and EA (eluent: PE: EA = 50:1, R_f = 0.3);
^{1}H NMR (400 MHz, $CDCl_3$): δ 5.1 (s, 1H), 2.37 (t, *J* = 7.24 Hz, 2H), 2.12−2.03 (m, 2H), 1.43−1.23 (m, 28H), 0.91−0.85 (m, 6H);
^{13}C NMR (101 MHz, $CDCl_3$): δ 167.8, 112.3, 82.4, 39.1, 34.5, 31.9, 31.8, 29.50, 29.46, 29.2, 27.8, 24.7, 22.64, 22.61, 14.1, 13.9;
^{11}B NMR (128 MHz, $CDCl_3$): δ 29.64;
HRMS-ESI: *m/z* $[M+Na]^+$ calcd for $C_{20}H_{39}BO_2$: 345.2935, found 345.2932.

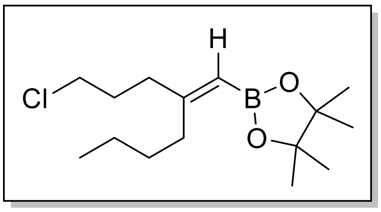

(*E*)-2-(2-(3-chloropropyl)hex-1-en-1-yl)-4,4,5,5-tetramethyl-1,3,2-dioxaborolane (**4ao**) was isolated (122 mg, 0.43 mmol, 85%) as a colorless oil after chromatograph on silica with PE and EA (eluent: PE: EA = 50:1, R_f = 0.3);
^{1}H NMR (400 MHz, $CDCl_3$): δ 5.11 (s, 1H), 3.53 (t, *J* = 6.68 Hz, 2H), 2.39 (t, *J* = 7.24 Hz, 2H), 2.24 (t, *J* = 11.1 Hz, 2H), 1.96−1.81 (m, 2H), 1.41−1.30 (m, 4H), 1.26 (s, 12 H), 0.91 (t, *J* = 7.20 Hz, 3H);
^{13}C NMR (101 MHz, $CDCl_3$): δ 165.3, 82.6, 44.7, 35.9, 34.5, 31.7, 30.6, 24.8, 22.6, 13.9;
^{11}B NMR (128 MHz, $CDCl_3$): δ 30.44;
HRMS-ESI: *m/z* $[M+H]^+$ calcd for $C_{15}H_{28}BClO_2$: 287.1944, found 287.1939.

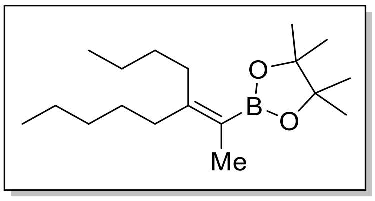

(*Z*)-2-(3-butyloct-2-en-2-yl)-4,4,5,5-tetramethyl-1,3,2-dioxaborolane (**4ap**) was isolated (119 mg, 0.43 mmol, 81%) as a colorless oil after chromatograph on silica with PE and EA (eluent: PE: EA = 50:1, R_f = 0.3);
¹H NMR (400 MHz, $CDCl_3$): δ 2.28 (t, J = 7.32 Hz, 2H), 1.67 (s, 3H), 1.33−1.25 (m, 22H), 0.91−0.87 (m, 6H);
¹³C NMR (101 MHz, $CDCl_3$): δ 156.6, 82.6, 35.7, 32.7, 32.4, 32.3, 27.7, 24.8, 22.9, 22.6, 16.1, 14.0;
¹¹B NMR (128 MHz, $CDCl_3$): δ 31.16;
HRMS-ESI: *m/z* $[M+H]^+$ calcd for $C_{18}H_{35}BO_2$: 294.2839, found 294.2829.

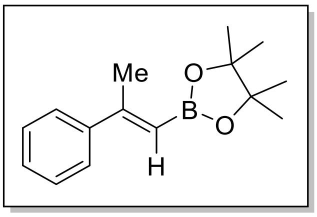

(*E*)-4,4,5,5-tetramethyl-2-(2-phenylprop-1-en-1-yl)-1,3,2-dioxaborolane (**4aq**, from **2b**) was isolated (105 mg, 0.43 mmol, 86%) as a colorless oil after chromatograph on silica with PE and EA (eluent: PE: EA = 50:1, R_f = 0.3);
¹H NMR (400 MHz, $CDCl_3$): δ 7.53−7.46 (m, 2H), 7.33−7.24 (m, 3H), 5.76 (s, 1H), 2.41 (s, 3H), 1.31 (s, 12H);
¹³C NMR (101 MHz, $CDCl_3$): δ 157.7, 143.7, 128.1, 127.9, 125.8, 115.4, 82.9, 24.8, 20.0;
¹¹B NMR (128 MHz, $CDCl_3$): δ 30.2;
HRMS-ESI: *m/z* $[M+Na]^+$ calcd for $C_{15}H_{21}BO_2$: 266.1563, found 266.1571.

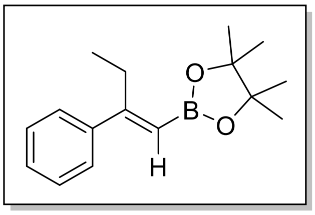

(*E*)-4,4,5,5-tetramethyl-2-(2-phenylbut-1-en-1-yl)-1,3,2-dioxaborolane (4ar, from **2c)** was isolated (106 mg, 0.41 mmol, 82%) as a colorless oil after chromatograph on silica with PE and EA (eluent: PE: EA = 50:1, R_f = 0.3);
¹H NMR (600 MHz, $CDCl_3$): δ 7.46−7.43 (m, 2H), 7.33−7.30 (m, 2H), 7.28−7.25 (m, 1H), 5.61 (s, 1H), 2.90 (q, *J* = 7.5 Hz, 2H), 1.30 (s, 12H), 1.02 (t, *J* = 7.5 Hz, 3H);
¹³C NMR (151 MHz, $CDCl_3$): δ 164.9, 143.0, 128.1, 127.7, 126.3, 115.3, 82.8, 26.6, 24.8, 14.7;
¹¹B NMR (192 MHz, $CDCl_3$): δ 30.2;
HRMS-ESI: *m/z* $[M+Na]^+$ calcd for $C_{16}H_{23}BO_2$: 258.1900, found 258.1890.

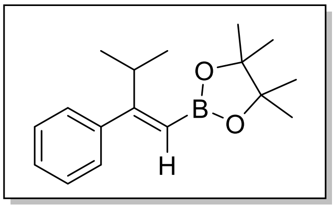

(*E*)-4,4,5,5-tetramethyl-2-(3-methyl-2-phenylbut-1-en-1-yl)-1,3,2-dioxaborolane (**4as**, from **2d**) was isolated (54 mg, 0.2 mmol, 40%) as a colorless oil after chromatograph on silica with PE and EA (eluent: PE: EA = 50:1, R_f = 0.3);
¹H NMR (400 MHz, $CDCl_3$): δ 7.28−7.24 (m, 3H), 7.21−7.17 (m, 2H), 5.25 (s, 1H), 3.55−3.43 (m, 1H), 1.30 (s, 12H), 1.08 (d, *J* = 7.04 Hz, 6H);
¹³C NMR (101 MHz, $CDCl_3$): δ 170.1, 144.2, 127.51, 127.45, 126.6, 82.9, 33.3, 24.8, 22.4;
¹¹B NMR (128 MHz, $CDCl_3$): δ 30.1;
HRMS-ESI: *m/z* $[M+H]^+$ calcd for $C_{17}H_{25}BO_2$: 272.2057, found 272.2066.

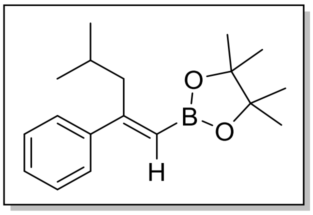

(*E*)-4,4,5,5-tetramethyl-2-(4-methyl-2-phenylpent-1-en-1-yl)-1,3,2-dioxaborolane (**4at**, from **2e**) was isolated (106 mg, 0.37 mmol, 74%) as a colorless oil after chromatograph on silica with PE and EA (eluent: PE: EA = 50:1, R_f = 0.3);
^{1}H NMR (400 MHz, $CDCl_3$): δ 7.44−7.40 (m, 2H), 7.32−7.23 (m, 3H), 5.65 (s, 1H), 2.82 (d, *J* = 7.2 Hz, 2H), 1.60−1.50 (m, 1H), 1.30 (s, 12H), 0.84 (d, *J* = 6.6 Hz, 6H);
^{13}C NMR (101 MHz, $CDCl_3$): δ 162.5, 143.7, 128.1, 127.6, 126.4, 117.3, 82.8, 41.9, 27.5, 24.8, 22.3;
^{11}B NMR (128 MHz, $CDCl_3$): δ 30.0;
HRMS-ESI: *m/z* $[M+H]^+$ calcd for $C_{18}H_{27}BO_2$: 286.2213, found 286.2206.

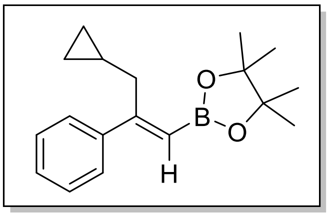

(*E*)-2-(3-cyclopropyl-2-phenylprop-1-en-1-yl)-4,4,5,5-tetramethyl-1,3,2-dioxaborolane (**4au**, from **2f**) was isolated (108 mg, 0.38 mmol, 76%) as a colorless oil after chromatograph on silica with PE and EA (eluent: PE: EA = 50:1, R_f = 0.3);
^{1}H NMR (400 MHz, $CDCl_3$): δ 7.52−7.46 (m, 2H), 7.34−7.23 (m, 3H), 5.63 (s, 1H), 2.84 (d, *J* = 6.9 Hz, 2H), 1.28 (s, 12H), 0.78−0.67 (m, 1H), 0.33−0.28 (m, 2H), 0.19−0.12 (m, 2H);
^{13}C NMR (101 MHz, $CDCl_3$): δ 162.6, 143.5, 128.1, 127.7, 126.5, 116.0, 82.8, 37.2, 24.8, 10.9, 4.5;
^{11}B NMR (128 MHz, $CDCl_3$): δ 30.0;
HRMS-ESI: *m/z* $[M+H]^+$ calcd for $C_{18}H_{25}BO_2$: 284.2057, found 284.2054.

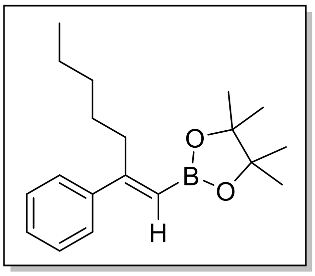

(*E*)-4,4,5,5-tetramethyl-2-(2-phenylhept-1-en-1-yl)-1,3,2-dioxaborolane (**4av**, from **2g**) was isolated (131 mg, 0.44 mmol, 87%) as a colorless oil after chromatograph on silica with PE and EA (eluent: PE: EA = 50:1, R_f = 0.3);
^{1}H NMR (400 MHz, $CDCl_3$): δ 7.46−7.41 (m, 2H), 7.33−7.23 (m, 3H), 5.63 (s, 1H), 2.89 (t, *J* = 7.3 Hz, 2H), 1.40−1.27 (m, 18H), 0.85 (t, *J* = 6.8 Hz, 3H);
^{13}C NMR (101 MHz, $CDCl_3$): δ 163.5, 143.3, 128.1, 127.6, 126.3, 116.1, 82.8, 33.2, 31.5, 29.4, 24.8, 22.3, 14.0;
^{11}B NMR (128 MHz, $CDCl_3$): δ 29.6;
HRMS-ESI: *m/z* $[M+H]^+$ calcd for $C_{19}H_{29}BO_2$: 300.2370, found 300.2369.

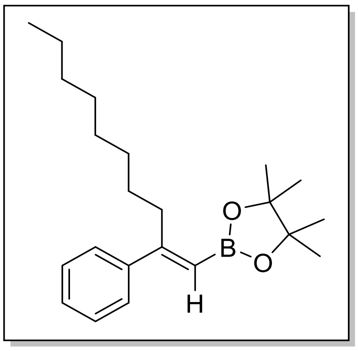

(*E*)-4,4,5,5-tetramethyl-2-(2-phenyldec-1-en-1-yl)-1,3,2-dioxaborolane (**4aw**, from **2h**) was isolated (130 mg, 0.38 mmol, 76%) as a colorless oil after chromatograph on silica with PE and EA (eluent: PE: EA = 50:1, R_f = 0.3);
^{1}H NMR (400 MHz, $CDCl_3$): δ 7.45−7.40 (m, 2H), 7.34−7.23 (m, 3H), 5.62 (s, 1H), 2.88 (t, *J* = 7.2 Hz, 2H), 1.39−1.23 (m, 24H), 0.86 (t, *J* = 6.6 Hz, 3H);

^{13}C NMR (101 MHz, $CDCl_3$): δ 163.6, 143.4, 128.1, 127.7, 126.3, 82.8, 33.3, 31.9, 29.8, 29.4, 29.30, 29.26, 24.8, 22.7, 14.1;
^{11}B NMR (128 MHz, $CDCl_3$): δ 30.4;
HRMS-ESI: *m/z* $[M+Na]^+$ calcd for $C_{22}H_{35}BO_2$: 342.2839, found 342.2834.

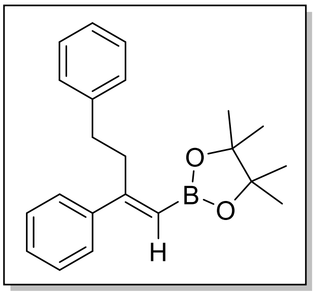

(*E*)-2-(2,4-diphenylbut-1-en-1-yl)-4,4,5,5-tetramethyl-1,3,2-dioxaborolane (**4ax**, from **2i**) was isolated (117 mg, 0.35 mg, 70%) as a colorless oil after chromatograph on silica with PE and EA (eluent: PE: EA = 50:1, R_f = 0.2);
^{1}H NMR (400 MHz, $CDCl_3$): δ 7.52−7.46 (m, 2H), 7.38−7.29 (m, 3H), 7.29−7.24 (m, 2H), 7.22−7.14 (m, 3H), 5.68 (s, 1H), 3.23−3.15 (m, 2H), 2.74−2.65 (m, 2H), 1.30 (s, 12H);
^{13}C NMR (101 MHz, $CDCl_3$): δ 162.3, 142.9, 142.2, 128.5, 128.3, 128.1, 127.9, 126.4, 125.7, 116.8, 82.9, 36.4, 35.6, 24.9;
^{11}B NMR (128 MHz, $CDCl_3$): δ 29.4;
HRMS-ESI: *m/z* $[M+Na]^+$ calcd for $C_{22}H_{27}BO_2$: 356.2033, found 356.2028.

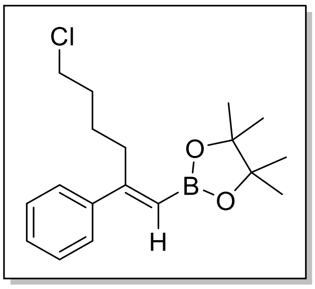

(*E*)-2-(6-chloro-2-phenylhex-1-en-1-yl)-4,4,5,5-tetramethyl-1,3,2-dioxaborolane (**4ay**, from **2j**) was isolated (107 mg, 0.34 mmol, 67%) as a colorless oil after chromatograph on silica with PE and EA (eluent: PE: EA = 50:1, R_f = 0.3);
^{1}H NMR (400 MHz, $CDCl_3$): δ 7.44−7.40 (m, 2H), 7.35−7.27 (m, 3H), 5.65 (s, 1H), 3.18 (t, *J* = 7.2 Hz, 2H), 2.93 (t, *J* = 7.4 Hz, 2H), 1.86−1.78 (m, 2H), 1.50−1.44 (m, 2H), 1.31 (s, 12H);
^{13}C NMR (101 MHz, $CDCl_3$): δ 162.6, 142.9, 128.2, 127.9, 126.3, 83.0, 44.8, 32.1, 32.0, 26.5, 24.8;
^{11}B NMR (128 MHz, $CDCl_3$): δ 29.7;
HRMS-ESI: *m/z* $[M+Na]^+$ calcd for $C_{18}H_{26}BClO_2$: 342.1643, found 342.1653.

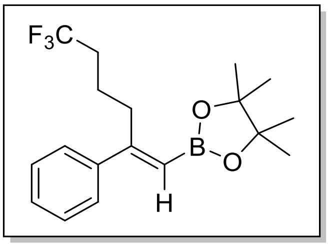

(*E*)-4,4,5,5-tetramethyl-2-(6,6,6-trifluoro-2-phenylhex-1-en-1-yl)-1,3,2-dioxaborolane (**4az**, from **2k**) was isolated (124 mg, 0.37 mmol, 73%) as a colorless oil after chromatograph on silica with PE and EA (eluent: PE: EA = 50:1, R_f = 0.3);
^{1}H NMR (600 MHz, $CDCl_3$): δ 7.44−7.39 (m, 2H), 7.35−7.27 (m, 3H), 5.70 (s, 1H), 2.97 (t, *J* = 7.5 Hz, 2H), 2.12−2.03 (m, 2H), 1.68−1.62 (m, 2H), 1.30 (s, 12H);
^{13}C NMR (151 MHz, $CDCl_3$): δ 161.6, 142.5, 128.4, 128.1, 126.2, 117.5, 83.1, 33.1 (q, *J* = 28.2 Hz), 32.0, 24.8, 21.7 (q, *J* = 2.97 Hz);
^{19}F NMR (565 MHz, $CDCl_3$): δ -66.3 (s);
^{11}B NMR (192 MHz, $CDCl_3$): δ 30.0;
HRMS-ESI: *m/z* $[M+H]^+$ calcd for $C_{18}H_{24}BF_3O_2$: 340.1931, found 340.1936.

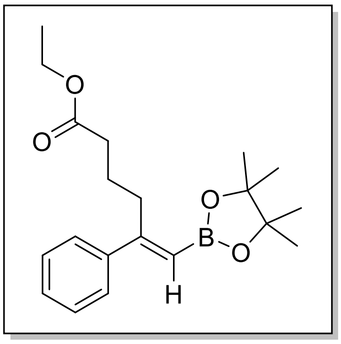

Ethyl (*E*)-5-phenyl-6-(4,4,5,5-tetramethyl-1,3,2-dioxaborolan-2-yl)hex-5-enoate (**4ba**, from **2l**) was isolated (98 mg, 0.29 mmol, 57%) as a colorless oil after chromatograph on silica with PE and EA (eluent: PE: EA = 50:1, R_f = 0.2);
^{1}H NMR (400 MHz, $CDCl_3$): δ 7.47−7.41 (m, 2H), 7.35−7.24 (m, 3H), 5.68 (s, 1H), 4.09 (q, *J* = 7.1 Hz, 2H), 2.94 (t, *J* = 7.4 Hz, 2H), 2.30 (t, *J* = 7.6 Hz, 2H), 1.76−1.68 (m, 2H), 1.30 (s, 12H), 1.22 (t, *J* = 7.2 Hz, 3H);
^{13}C NMR (101 MHz, $CDCl_3$): δ 173.6, 162.1, 142.7, 128.2, 127.9, 126.3, 82.9, 60.1, 33.8, 32.5, 29.7, 24.8, 14.2;
^{11}B NMR (128 MHz, $CDCl_3$): δ 30.1;
HRMS-ESI: *m/z* $[M+Na]^+$ calcd for $C_{20}H_{29}BO_4$: 366.2087, found 366.2089.

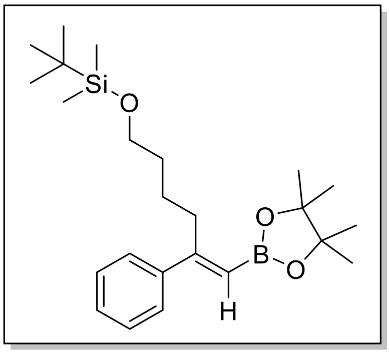

(*E*)-*tert*-butyldimethyl((5-phenyl-6-(4,4,5,5-tetramethyl-1,3,2-dioxaborolan-2-yl)hex-5-en-1-yl)oxy)silane (**4bb**, from **2m**) was isolated (104 mg, 0.25 mmol, 50%) as a colorless oil after chromatograph on silica with PE and EA (eluent: PE: EA = 50:1, R_f = 0.3);
^{1}H NMR (600 MHz, $CDCl_3$): δ 7.44−7.40 (m, 2H), 7.32−7.28 (m, 2H), 7.28−7.26 (m, 1H), 5.63 (s, 1H), 3.56 (t, *J* = 6.7 Hz, 2H), 2.90 (t, *J* = 7.6 Hz. 2H), 1.54−1.50 (m, 2H), 1.42−1.37 (m, 2H), 1.30 (s, 12H), 0.85 (s, 9H), 0 (s, 6H);
^{13}C NMR (151 MHz, $CDCl_3$): δ 163.3, 143.2, 128.1, 127.7, 126.4, 82.9, 63.1, 33.0, 32.5, 25.9, 24.8, 8.3, -5.3;
^{11}B NMR (192 MHz, $CDCl_3$): δ 30.4;
HRMS-ESI: *m/z* $[M+H]^+$ calcd for $C_{24}H_{41}BO_3Si$: 417.2991, found 417.2981.

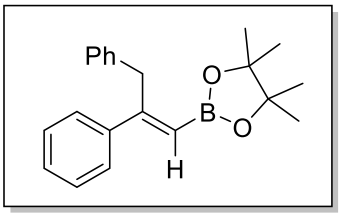

(*E*)-2-(2,3-diphenylprop-1-en-1-yl)-4,4,5,5-tetramethyl-1,3,2-dioxaborolane (**4bc**, from **2o** and **2p**) was isolated (**2o**: 122 mg, 0.38 mmol, 76%; **2p**: 104 mg, 0.33 mmol, 65%) as a colorless oil after chromatograph on silica with PE and EA (eluent: PE: EA = 50:1, R_f = 0.3);
^{1}H NMR (600 MHz, $CDCl_3$): δ 7.41−7.37 (m, 2H), 7.25−7.16 (m, 7H), 7.12−7.08 (m, 1H), 5.85 (s, 1H), 4.27 (s, 2H), 1.29 (s, 12H);
^{13}C NMR (151 MHz, $CDCl_3$): δ 160.3, 142.9, 140.2, 128.5, 128.10, 128.07, 126.6, 125.6, 83.1, 39.3, 24.8;
^{11}B NMR (192 MHz, $CDCl_3$): δ 30.3;
HRMS-ESI: *m/z* $[M+H]^+$ calcd for $C_{21}H_{25}BO_2$: 320.2057, found 320.2054.

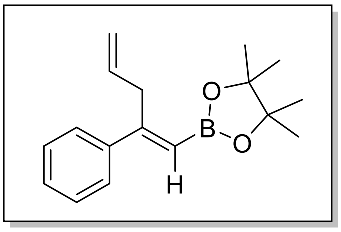

(*E*)-4,4,5,5-tetramethyl-2-(2-phenylpenta-1,4-dien-1-yl)-1,3,2-dioxaborolane (**4bd**, from **2q**) was isolated (85 mg, 0.32 mmol, 63%) as a colorless oil after chromatograph on silica with PE and EA (eluent: PE: EA = 50:1, R_f = 0.3);

^{1}H NMR (400 MHz, $CDCl_3$): δ 7.50−7.46 (m, 2H), 7.33−7.27 (m, 3H), 5.88−5.78 (m, 1H), 5.76 (s, 1H), 5.12−5.05 (m, 1H), 4.97−4.93 (m, 1H), 3.68 (d, J = 6.4 Hz, 2H), 1.30 (s, 12H);
^{13}C NMR (101 MHz, $CDCl_3$): δ 159.9, 142.9, 137.3, 128.1, 127.9, 126.4, 115.5, 83.0, 37.9, 24.8;
^{11}B NMR (128 MHz, $CDCl_3$): δ 30.1;
HRMS-ESI: *m/z* [M+Na]$^+$ calcd for $C_{17}H_{23}BO_2$: 292.1720, found 292.1721.

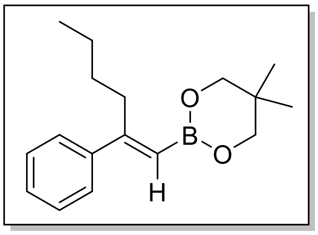

(*E*)-5,5-dimethyl-2-(2-phenylhex-1-en-1-yl)-1,3,2-dioxaborinane (**4be**, from **3b**) was isolated (98 mg, 0.36 mmol, 72%) as a colorless oil after chromatograph on silica with PE and EA (eluent: PE: EA = 50:1, R_f = 0.2);
^{1}H NMR (400 MHz, $CDCl_3$): δ 7.44−7.40 (m, 2H), 7.31−7.23 (m, 3H), 5.52 (s, 1H), 3.69 (s, 4H), 2.87 (t, J = 7.3 Hz, 2H), 1.36−1.29 (m, 4H), 1.0 (s, 6H), 0.86 (t, J = 6.9 Hz, 3H);
^{13}C NMR (101 MHz, $CDCl_3$): δ 161.2, 144.0, 128.0, 127.3, 126.4, 72.0, 32.5, 31.9, 31.6, 22.5, 21.9, 13.9;
^{11}B NMR (128 MHz, $CDCl_3$): δ 26.6;
HRMS-ESI: *m/z* [M+Na]$^+$ calcd for $C_{17}H_{25}BO_2$: 294.1876, found 294.1847.

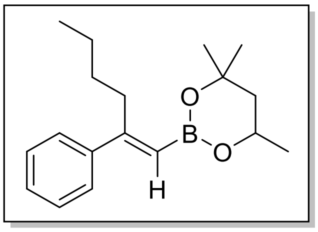

(*E*)-4,4,6-trimethyl-2-(2-phenylhex-1-en-1-yl)-1,3,2-dioxaborinane (4bf, from **3c)** was isolated (123 mg, 0.43 mmol, 85%) as a colorless oil after chromatograph on silica with PE and EA (eluent: PE: EA = 50:1, R_f = 0.3);
^{1}H NMR (400 MHz, $CDCl_3$): δ 7.45−7.40 (m, 2H), 7.30−7.22 (m, 3H), 5.53 (s, 1H), 4.32−4.21 (m, 1H), 2.96−2.78 (m, 2H), 1.80 (dd, J_1 = 2.9 Hz, J_2 = 13.9 Hz, 1H), 1.58−1.51 (m, 1H), 1.38−1.31 (m, 10H), 1.29 (d, J = 6.2 Hz, 3H), 0.88 (t, J = 7.0 Hz, 3H);
^{13}C NMR (101 MHz, $CDCl_3$): δ 160.5, 144.2, 127.9, 127.2, 126.3, 70.8, 64.7, 45.9, 32.5, 32.1, 31.3, 28.2, 23.2, 22.7, 14.0;
^{11}B NMR (128 MHz, $CDCl_3$): δ 26.3;
HRMS-ESI: *m/z* [M+Na]$^+$ calcd for $C_{18}H_{27}BO_2$: 308.2033, found 308.2028.

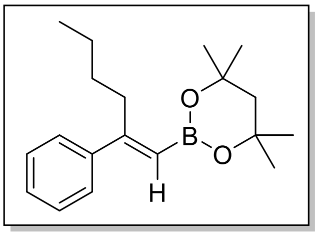

(*E*)-4,4,6,6-tetramethyl-2-(2-phenylhex-1-en-1-yl)-1,3,2-dioxaborinane (**4bi**, from **3d**) was isolated (123 mg, 0.41 mmol, 82%) as a colorless oil after chromatograph on silica with PE and EA (eluent: PE: EA = 50:1, R_f = 0.3);
^{1}H NMR (400 MHz, $CDCl_3$): δ 7.47−7.41 (m, 2H), 7.30−7.21 (m, 3H), 5.56 (s, 1H), 2.88 (t, J = 7.2 Hz, 2H), 1.87 (s, 2H), 1.41−1.31 (m, 16H), 0.88 (t, J = 7.0 Hz, 3H);
^{13}C NMR (101 MHz, $CDCl_3$): δ 160.2, 144.2, 127.9, 127.1, 126.3, 70.6, 48.8, 32.6, 32.2, 31.9, 22.8, 14.0;
^{11}B NMR (128 MHz, $CDCl_3$): δ 26.1;
HRMS-ESI: *m/z* [M+Na]$^+$ calcd for $C_{19}H_{29}BO_2$: 322.2189, found 322.2184.

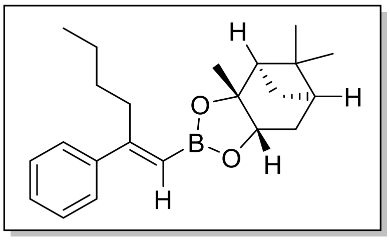

(3a*R*,4*R*,6*R*,7a*S*)-3a,5,5-trimethyl-2-((*E*)-2-phenylhex-1-en-1-yl)hexahydro-4,6-methanobenzo[*d*][1,3,2]dioxaborole (**4bj**, from **3e**) was isolated (144 mg, 0.44 mmol, 88%) as a colorless oil after chromatograph on silica with PE and EA (eluent: PE: EA = 50:1, R_f = 0.3);

^{1}H NMR (400 MHz, $CDCl_3$): δ 7.48−7.41 (m, 2H), 7.34−7.24 (m, 3H), 5.65 (s, 1H), 4.35 (dd, J_1 = 1.9 Hz, J_2 = 8.8 Hz, 1H), 2.96−2.84 (m, 2H), 2.42−2.33 (m, 1H), 2.28−2.20 (m, 1H), 2.10 (t, J = 5.4 Hz, 1H), 1.97−1.89 (m, 2H), 1.44 (s, 3H), 1.38−1.28 (m, 7H), 1.23 (d, J = 10.9 Hz, 1H), 0.87 (s, 3H), 0.86 (t, J = 7.0 Hz, 3H);
^{13}C NMR (101 MHz, $CDCl_3$): δ 163.4, 143.4, 128.1, 127.6, 126.3, 115.5, 85.2, 77.5, 51.4, 39.6, 38.1, 35.6, 33.1, 31.9, 28.7, 27.1, 26.5, 24.0, 22.5, 13.9;
^{11}B NMR (128 MHz, $CDCl_3$): δ 30.0;
HRMS-ESI: *m/z* $[M+Na]^+$ calcd for $C_{22}H_{31}BO_2$: 360.2346, found 360.2338.

3.14 NMR Spectra

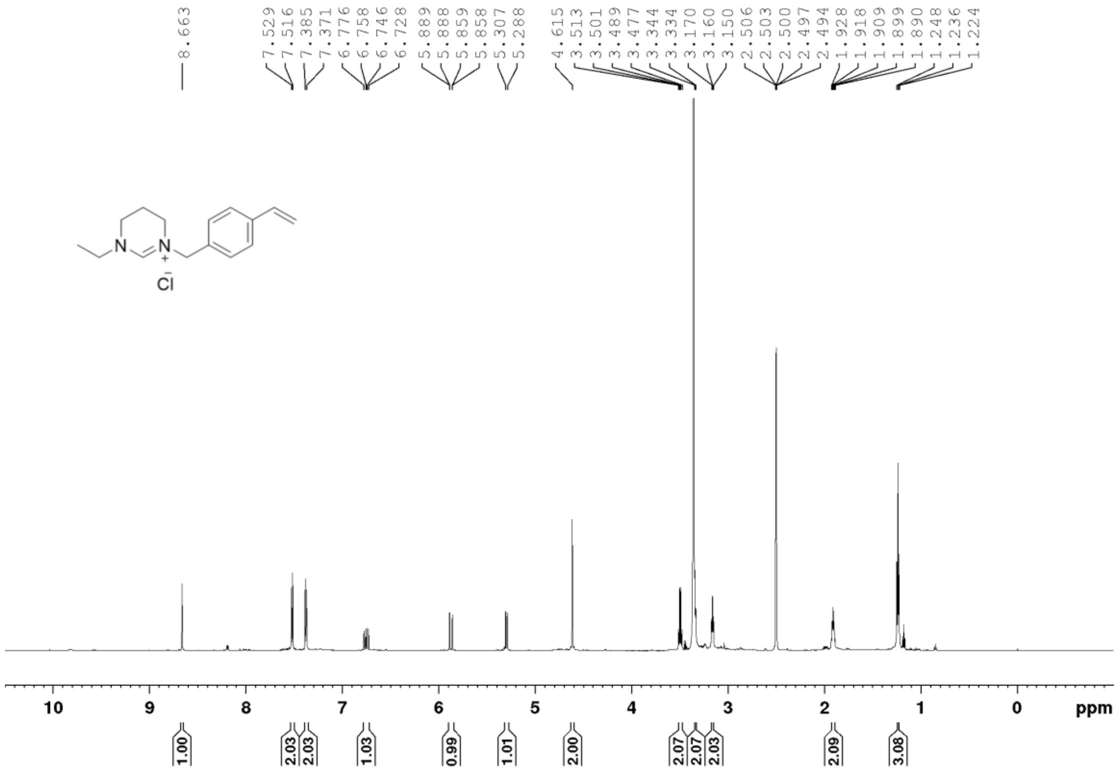

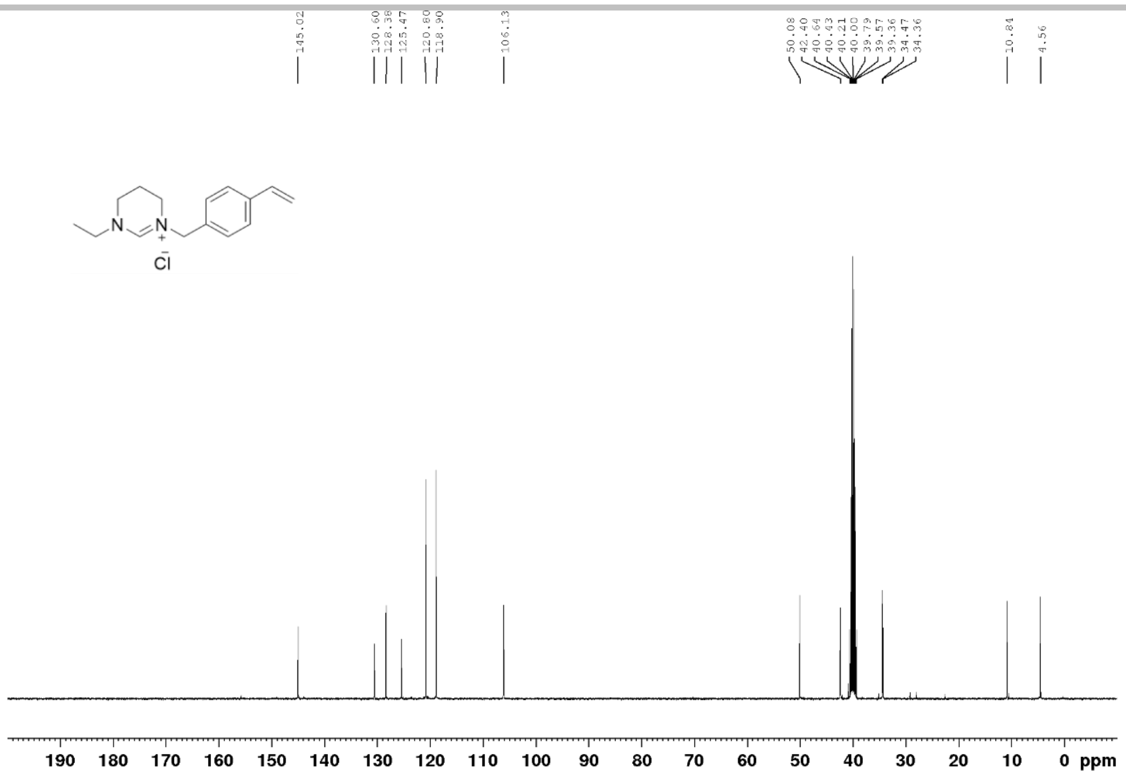

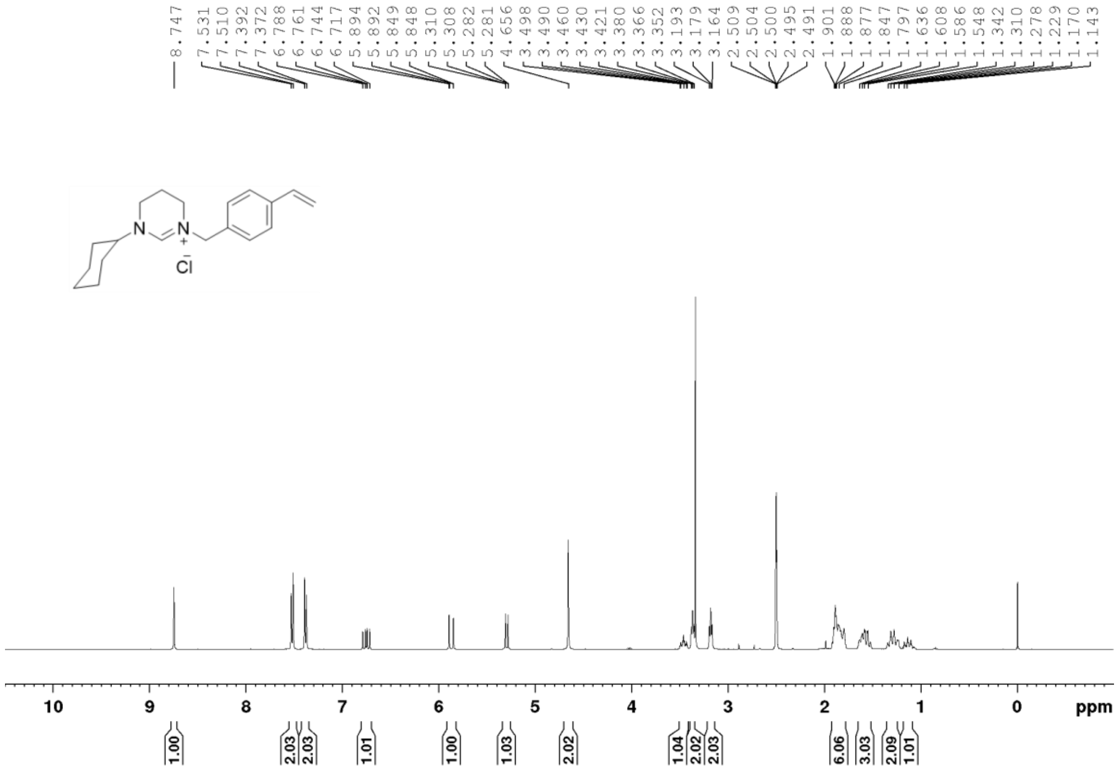

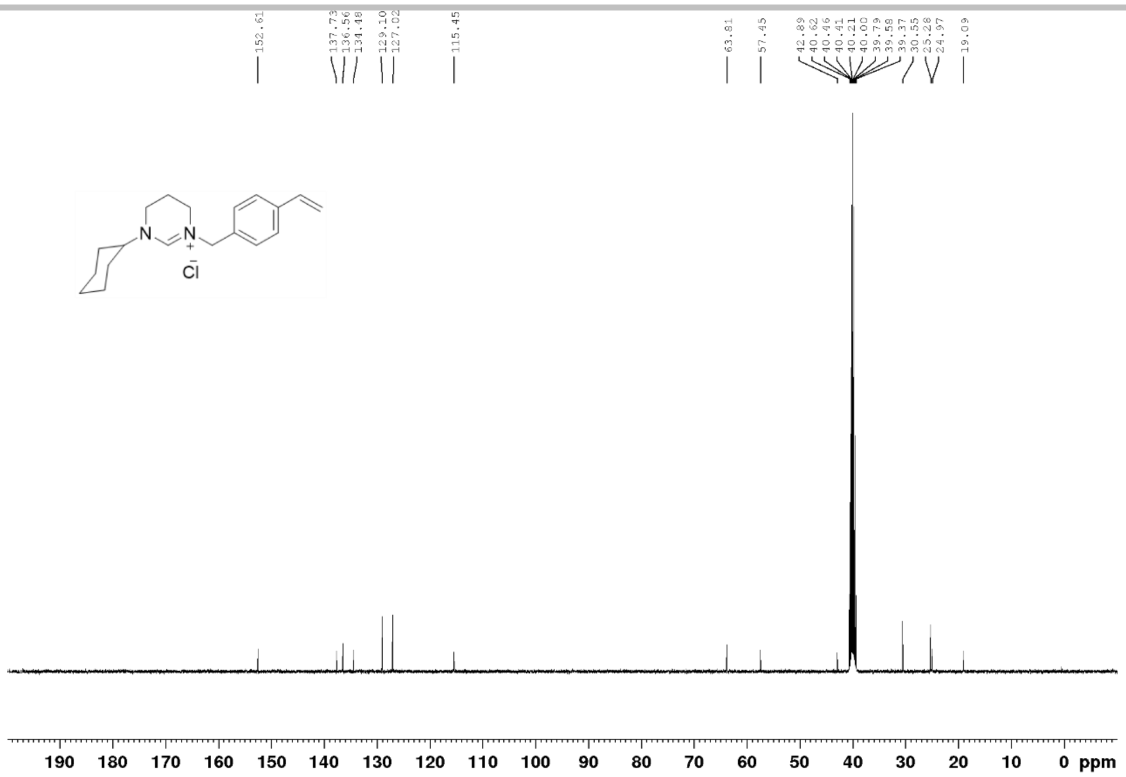
152.61
137.73
136.56
134.48
129.10
127.02
115.45
63.81
57.45
42.89
40.62
40.46
40.41
40.21
40.00
39.79
39.58
39.37
30.55
25.28
24.97
19.09
190 180 170 160 150 140 130 120 110 100 90 80 70 60 50 40 30 20 10 0 ppm

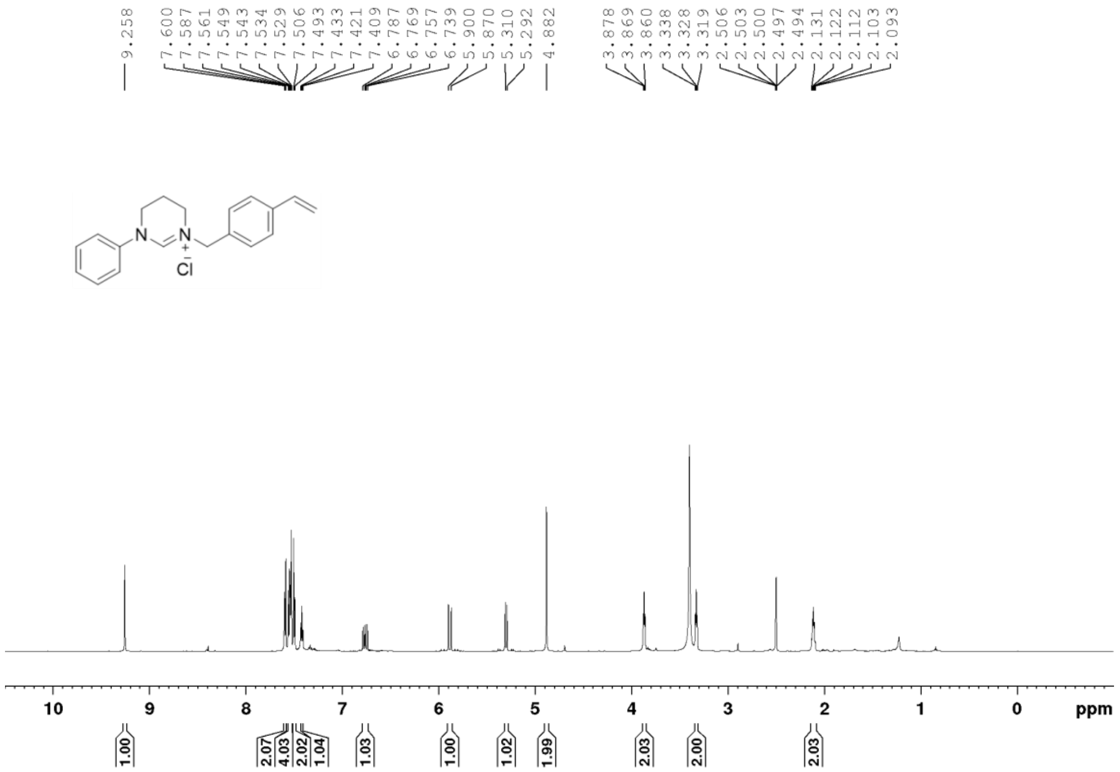
9.258
7.600
7.587
7.561
7.549
7.543
7.534
7.529
7.506
7.493
7.433
7.421
7.409
6.787
6.769
6.757
6.739
5.900
5.870
5.310
5.292
4.882
3.878
3.869
3.860
3.338
3.328
3.319
2.506
2.503
2.500
2.497
2.494
2.131
2.122
2.112
2.103
2.093
10 9 8 7 6 5 4 3 2 1 0 ppm
1.00
2.07
4.03
2.02
1.04
1.03
1.00
1.02
1.99
2.03
2.00
2.03

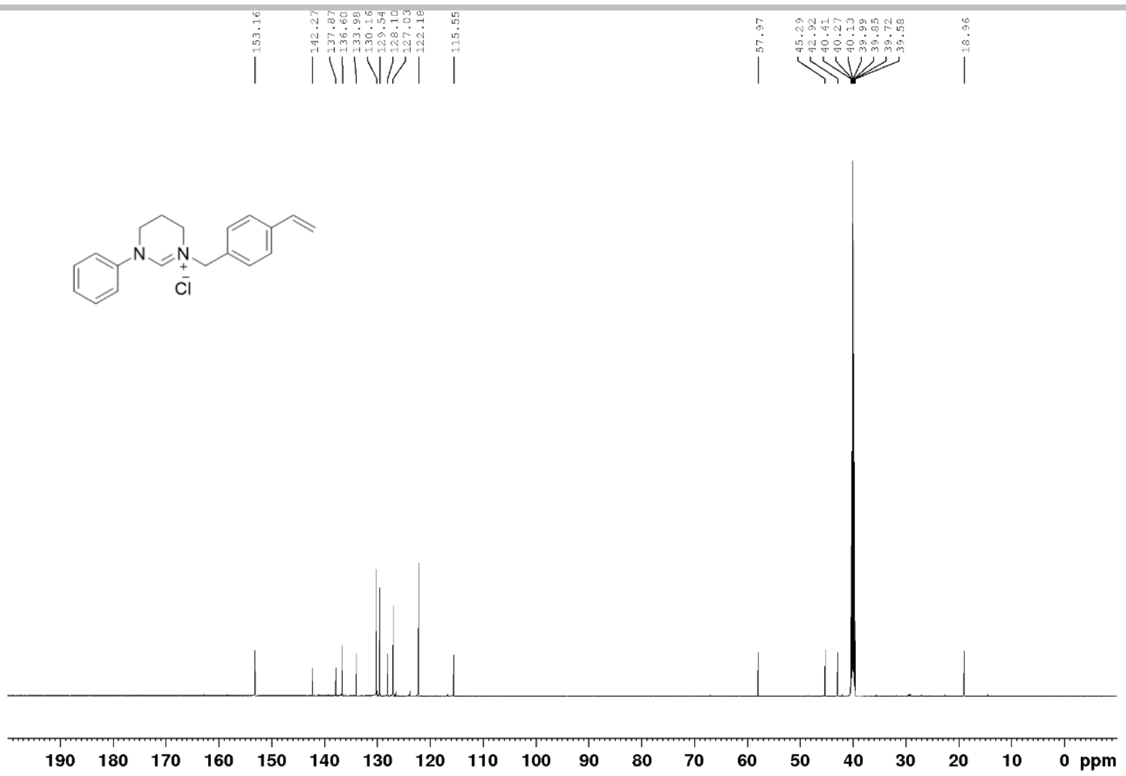
153.16
142.27
137.87
136.60
133.98
130.16
129.54
128.10
127.03
122.18
115.55
57.97
45.29
42.92
40.41
40.27
40.13
39.99
39.85
39.72
39.58
18.96
N
N
+
Cl
−
190 180 170 160 150 140 130 120 110 100 90 80 70 60 50 40 30 20 10 0 ppm

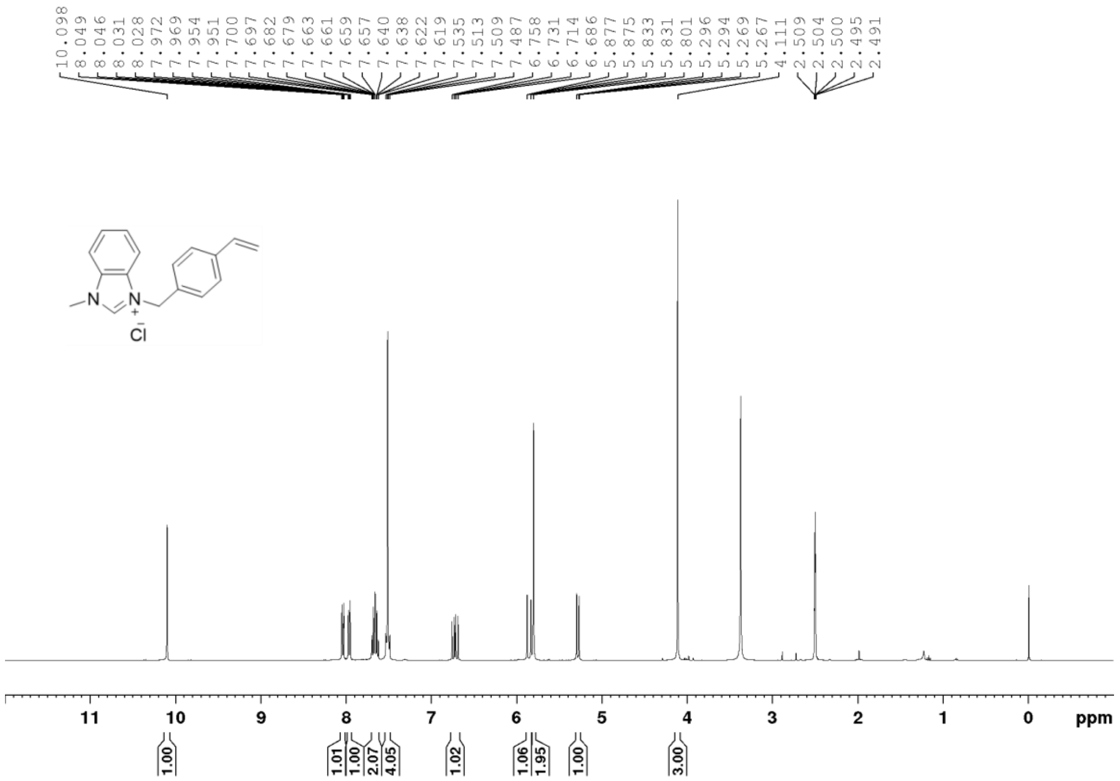
10.098
8.049
8.046
8.031
8.028
7.972
7.969
7.954
7.951
7.700
7.697
7.682
7.679
7.663
7.661
7.659
7.657
7.640
7.638
7.622
7.619
7.535
7.513
7.509
7.487
6.758
6.731
6.714
6.686
5.877
5.875
5.833
5.831
5.801
5.296
5.294
5.269
5.267
4.111
2.509
2.504
2.500
2.495
2.491
N
N
+
Cl
−
11 10 9 8 7 6 5 4 3 2 1 0 ppm
1.00
1.01
1.00
2.07
4.05
1.02
1.06
1.95
1.00
3.00

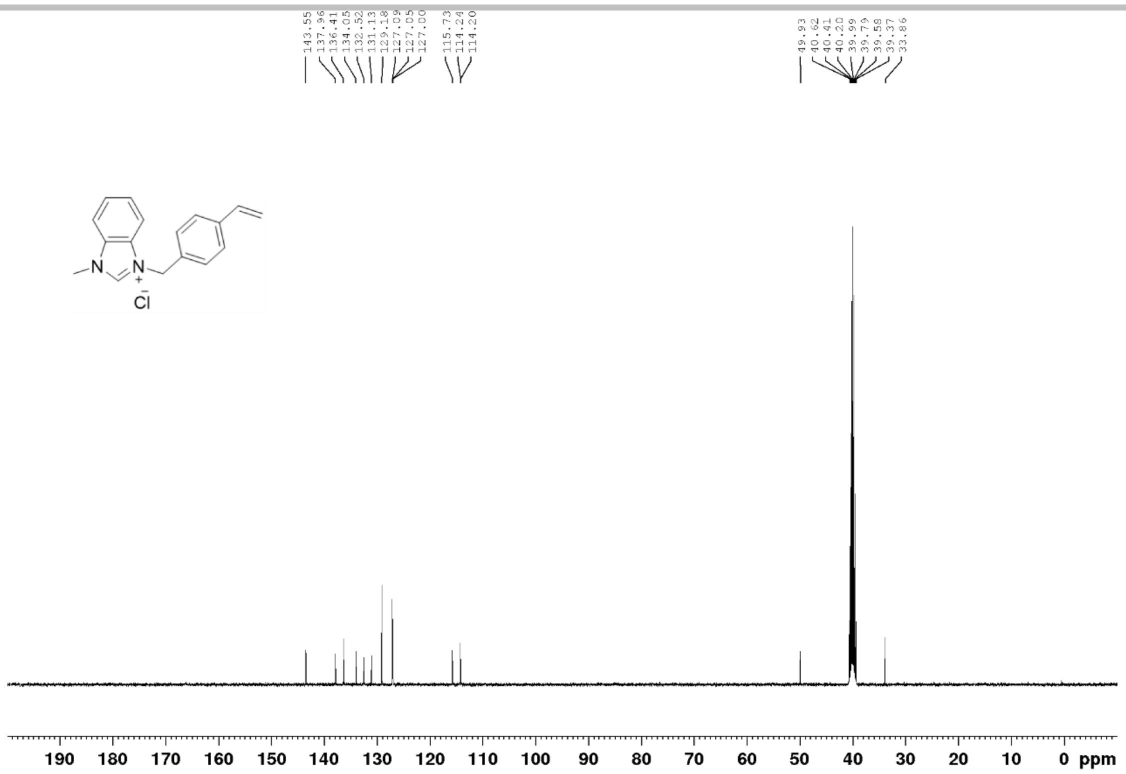
143.55
137.96
136.41
134.05
132.52
131.13
129.18
127.09
127.05
127.00
115.73
114.24
114.20
49.93
40.62
40.41
40.20
39.99
39.79
39.58
39.37
33.86
N
N+
Cl−
190 180 170 160 150 140 130 120 110 100 90 80 70 60 50 40 30 20 10 0 ppm

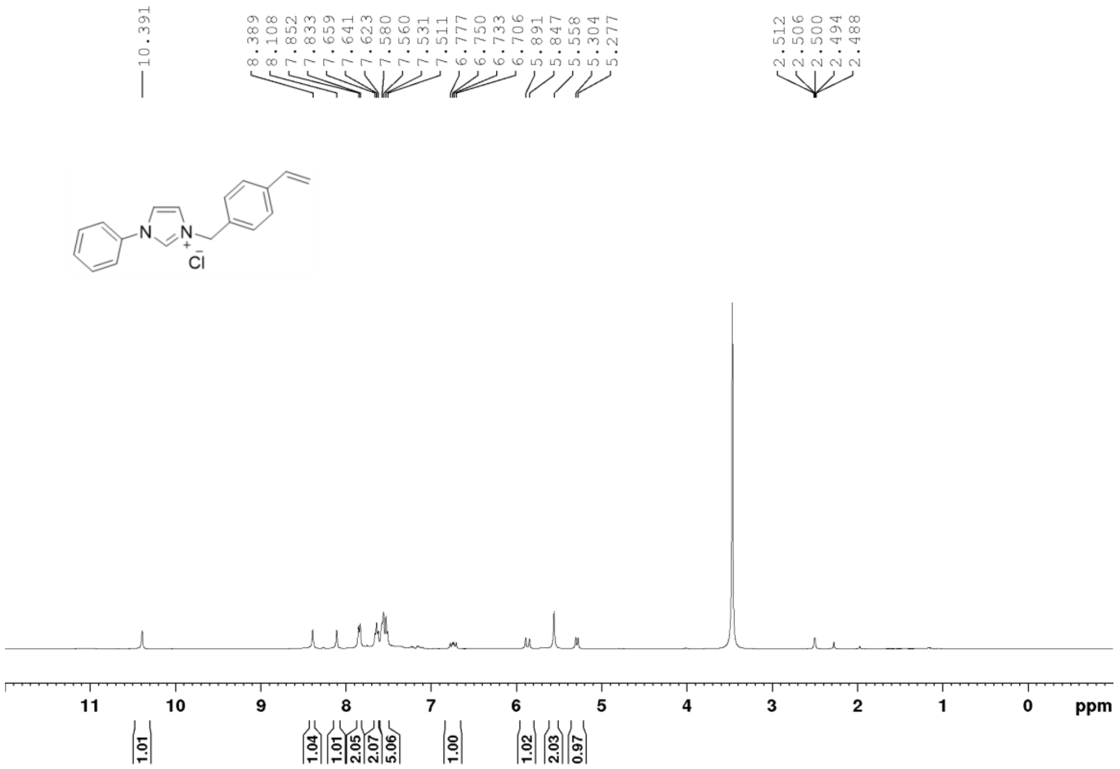
10.391
8.389
8.108
7.852
7.833
7.659
7.641
7.623
7.580
7.560
7.531
7.511
6.777
6.750
6.733
6.706
5.891
5.847
5.558
5.304
5.277
2.512
2.506
2.500
2.494
2.488
N
N+
Cl−
11 10 9 8 7 6 5 4 3 2 1 0 ppm
1.01
1.04
1.01
2.05
2.07
5.06
1.00
1.02
2.03
0.97

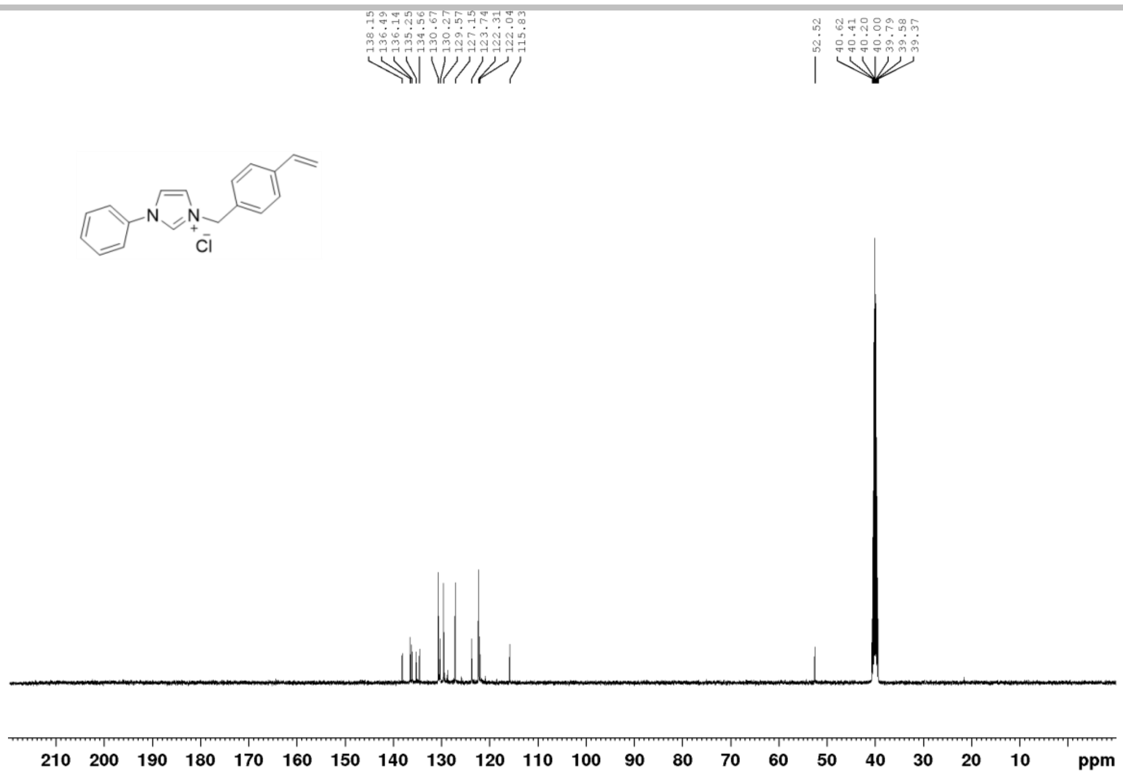
138.15
136.49
136.14
135.25
134.56
130.67
130.27
129.57
127.15
123.74
122.31
122.04
115.83
52.52
40.62
40.41
40.20
40.00
39.79
39.58
39.37
N
N
+
Cl
210 200 190 180 170 160 150 140 130 120 110 100 90 80 70 60 50 40 30 20 10 ppm

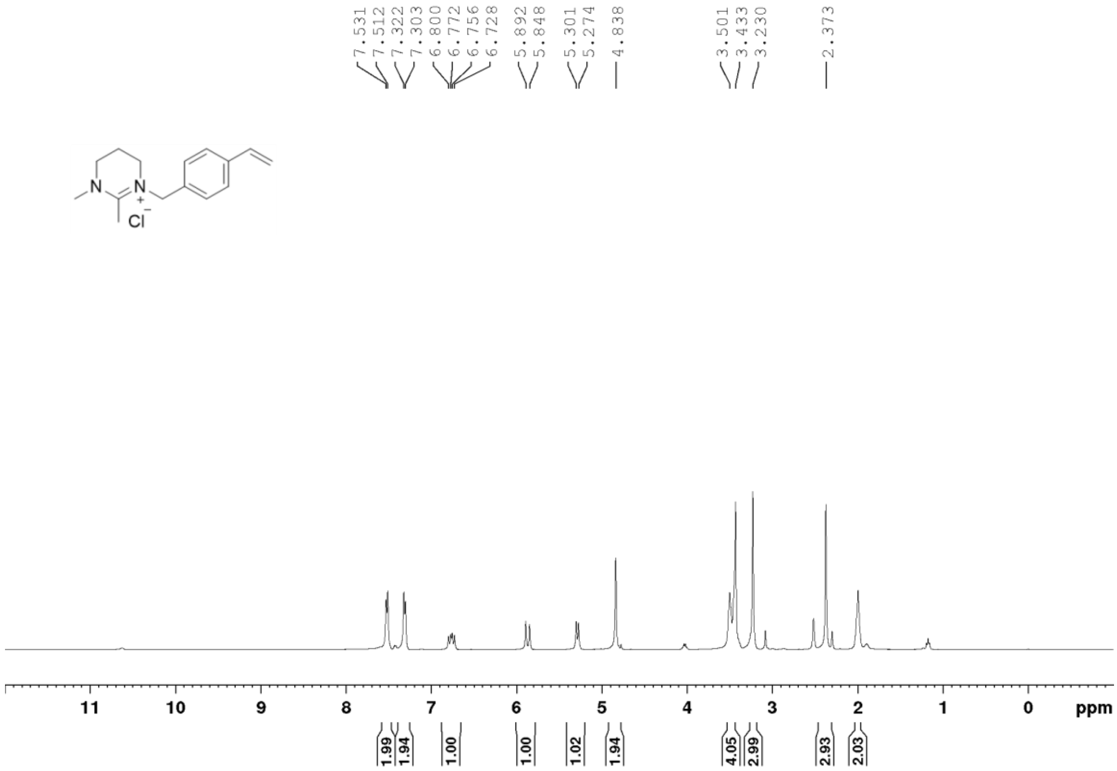
7.531
7.512
7.322
7.303
6.800
6.772
6.756
6.728
5.892
5.848
5.301
5.274
4.838
3.501
3.433
3.230
2.373
N
N
+
Cl
11 10 9 8 7 6 5 4 3 2 1 0 ppm
1.99
1.94
1.00
1.00
1.02
1.94
4.05
2.99
2.93
2.03

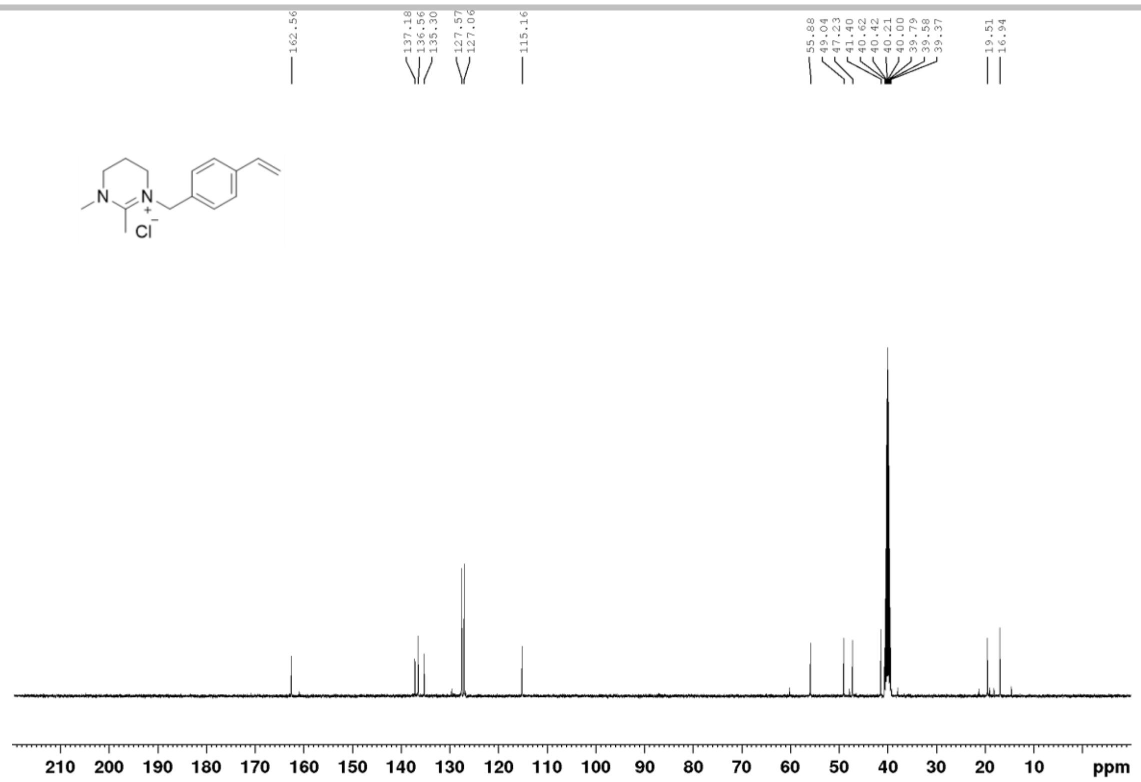
162.56
137.18
136.56
135.30
127.57
127.06
115.16
55.88
49.04
47.23
41.40
40.62
40.42
40.21
40.00
39.79
39.58
39.37
19.51
16.94
N
N
+
Cl
−
210 200 190 180 170 160 150 140 130 120 110 100 90 80 70 60 50 40 30 20 10 ppm

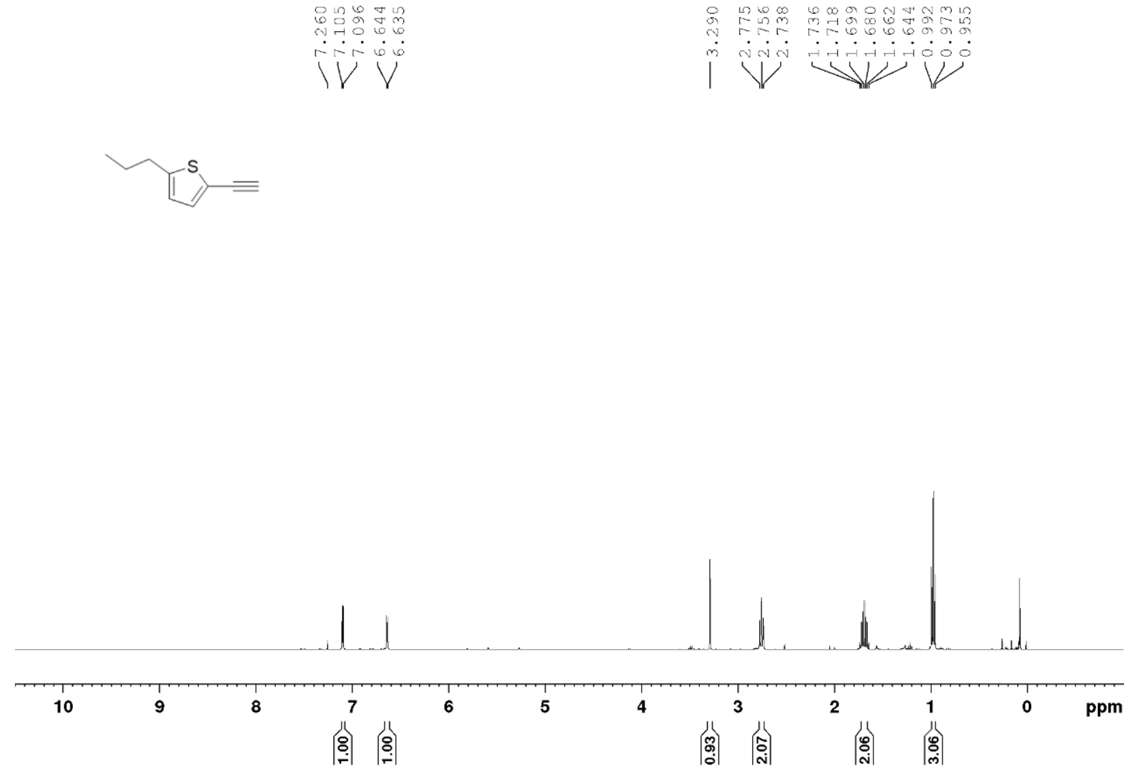
7.260
7.105
7.096
6.644
6.635
3.290
2.775
2.756
2.738
1.736
1.718
1.699
1.680
1.662
1.644
0.992
0.973
0.955
S
10 9 8 7 6 5 4 3 2 1 0 ppm
1.00
1.00
0.93
2.07
2.06
3.06

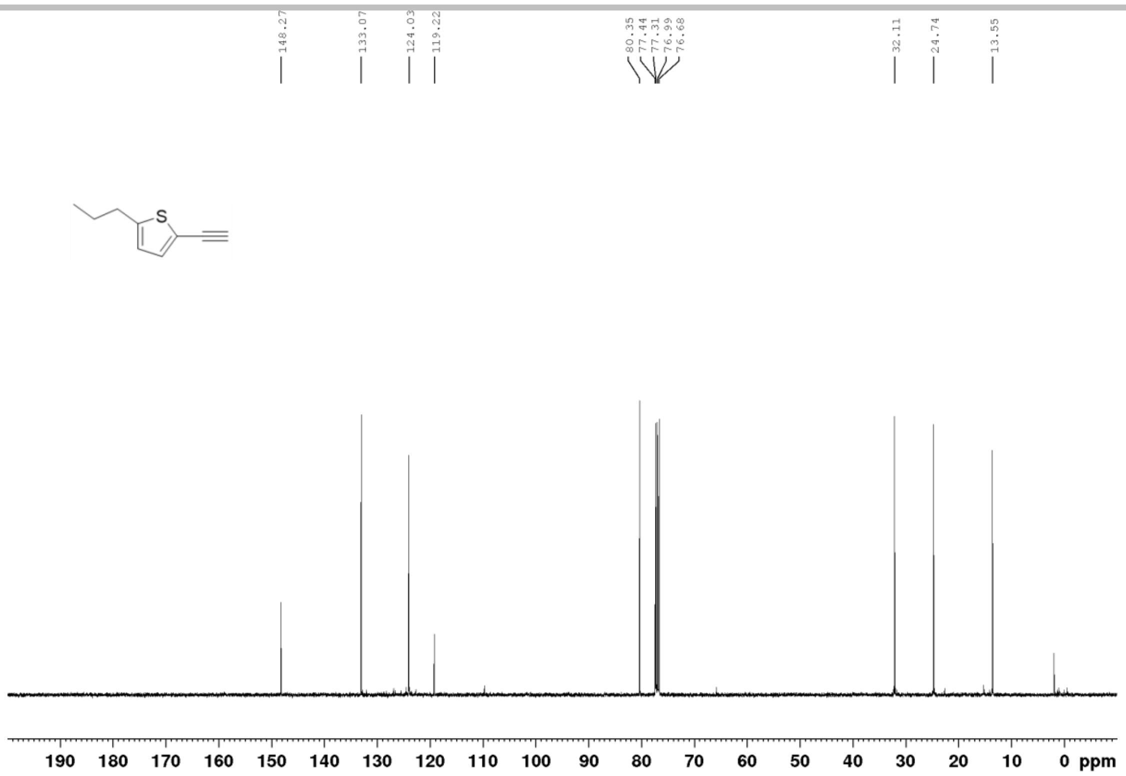
148.27
133.07
124.03
119.22
80.35
77.44
77.31
76.99
76.68
32.11
24.74
13.55
S
190 180 170 160 150 140 130 120 110 100 90 80 70 60 50 40 30 20 10 0 ppm

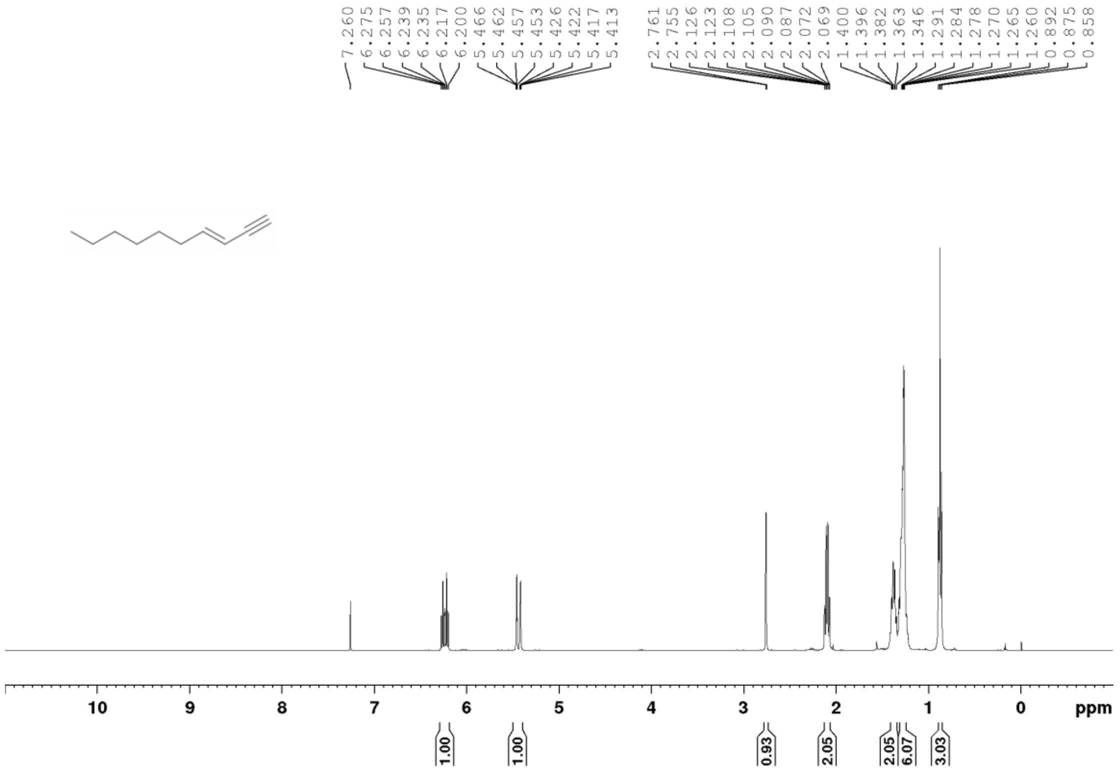
7.260
6.275
6.257
6.239
6.235
6.217
6.200
5.466
5.462
5.457
5.453
5.426
5.422
5.417
5.413
2.761
2.755
2.126
2.123
2.108
2.105
2.090
2.087
2.072
2.069
1.400
1.396
1.382
1.363
1.346
1.291
1.284
1.278
1.270
1.265
1.260
0.892
0.875
0.858
10 9 8 7 6 5 4 3 2 1 0 ppm
1.00
1.00
0.93
2.05
2.05
6.07
3.03

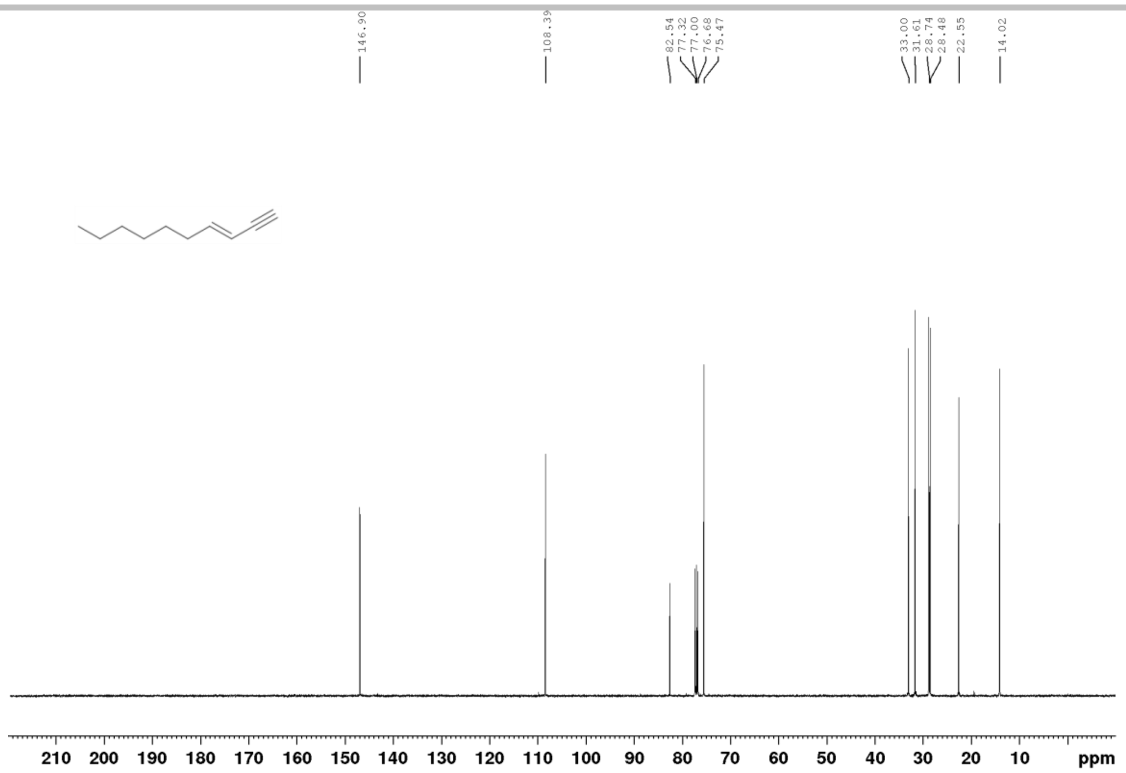
146.90
108.39
82.54
77.32
77.00
76.68
75.47
33.00
31.61
28.74
28.48
22.55
14.02
210 200 190 180 170 160 150 140 130 120 110 100 90 80 70 60 50 40 30 20 10 ppm

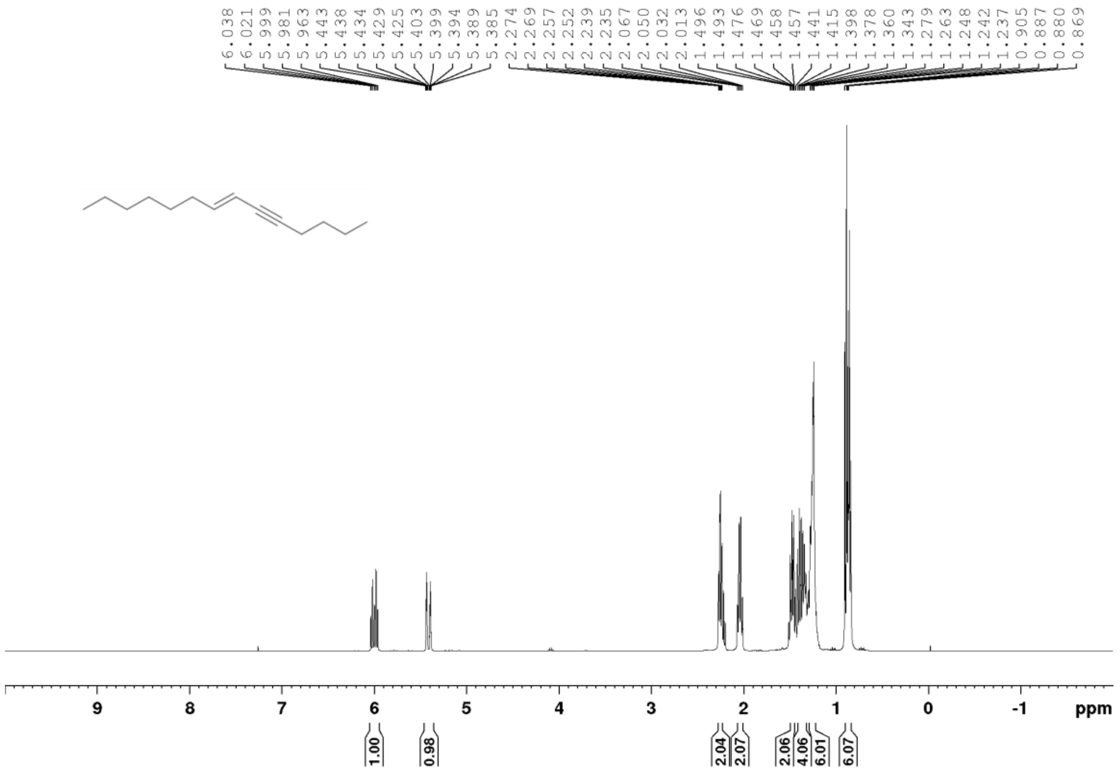
6.038
6.021
5.999
5.981
5.963
5.443
5.438
5.434
5.429
5.425
5.403
5.399
5.394
5.389
5.385
2.274
2.269
2.257
2.252
2.239
2.235
2.067
2.050
2.032
2.013
1.496
1.493
1.476
1.469
1.458
1.457
1.441
1.415
1.398
1.378
1.360
1.343
1.279
1.263
1.248
1.242
1.237
0.905
0.887
0.880
0.869
9 8 7 6 5 4 3 2 1 0 -1 ppm
1.00
0.98
2.04
2.07
2.06
4.06
6.01
6.07

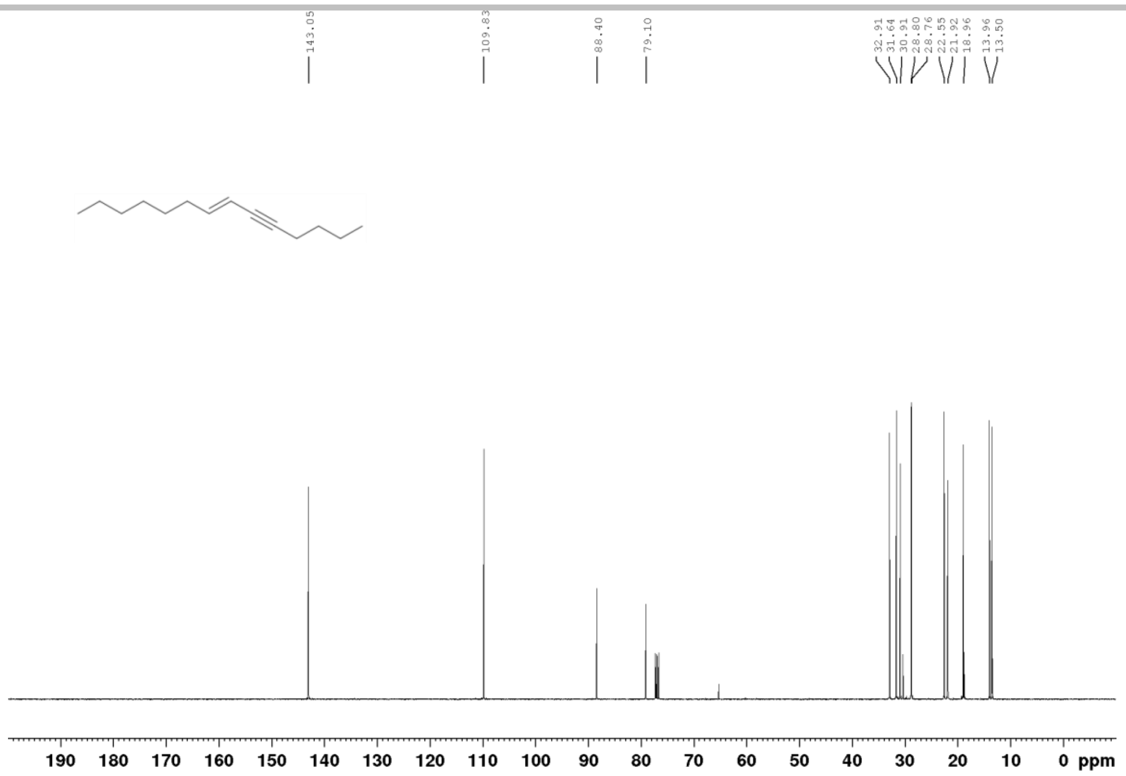
143.05
109.83
88.40
79.10
32.91
31.64
30.91
28.80
28.76
22.55
21.92
18.96
13.96
13.50
190 180 170 160 150 140 130 120 110 100 90 80 70 60 50 40 30 20 10 0 ppm

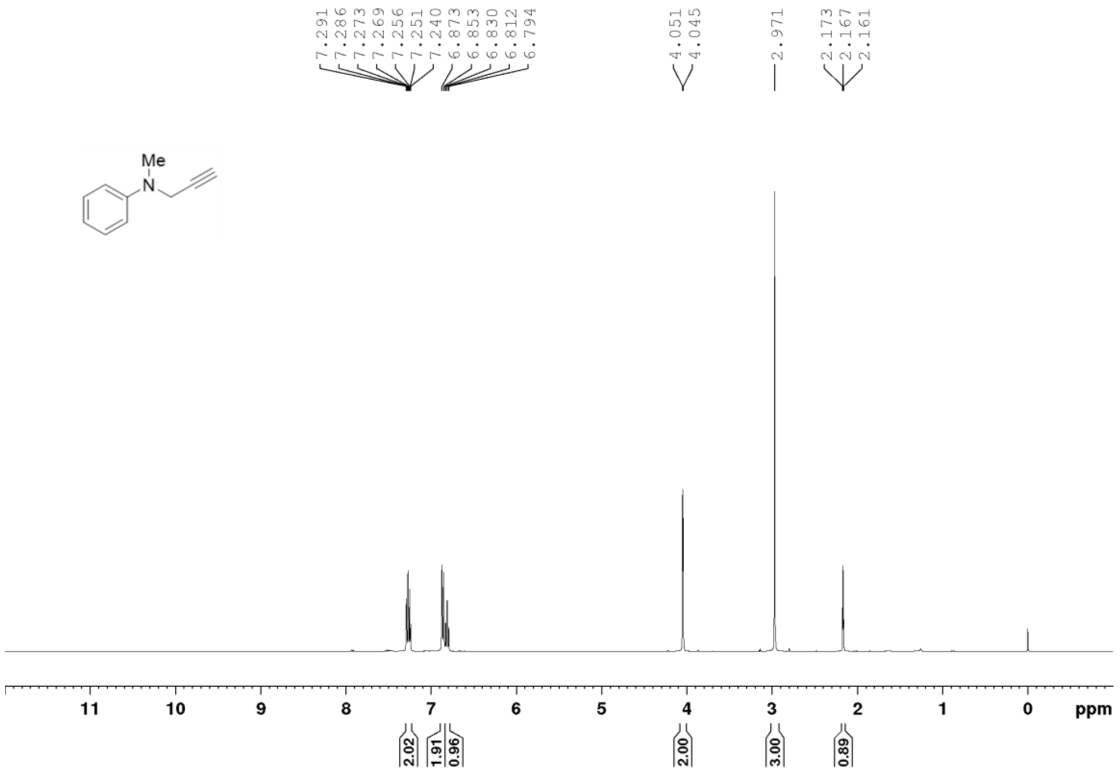
7.291
7.286
7.273
7.269
7.256
7.251
7.240
6.873
6.853
6.830
6.812
6.794
4.051
4.045
2.971
2.173
2.167
2.161
Me
N
11 10 9 8 7 6 5 4 3 2 1 0 ppm
2.02
1.91
0.96
2.00
3.00
0.89

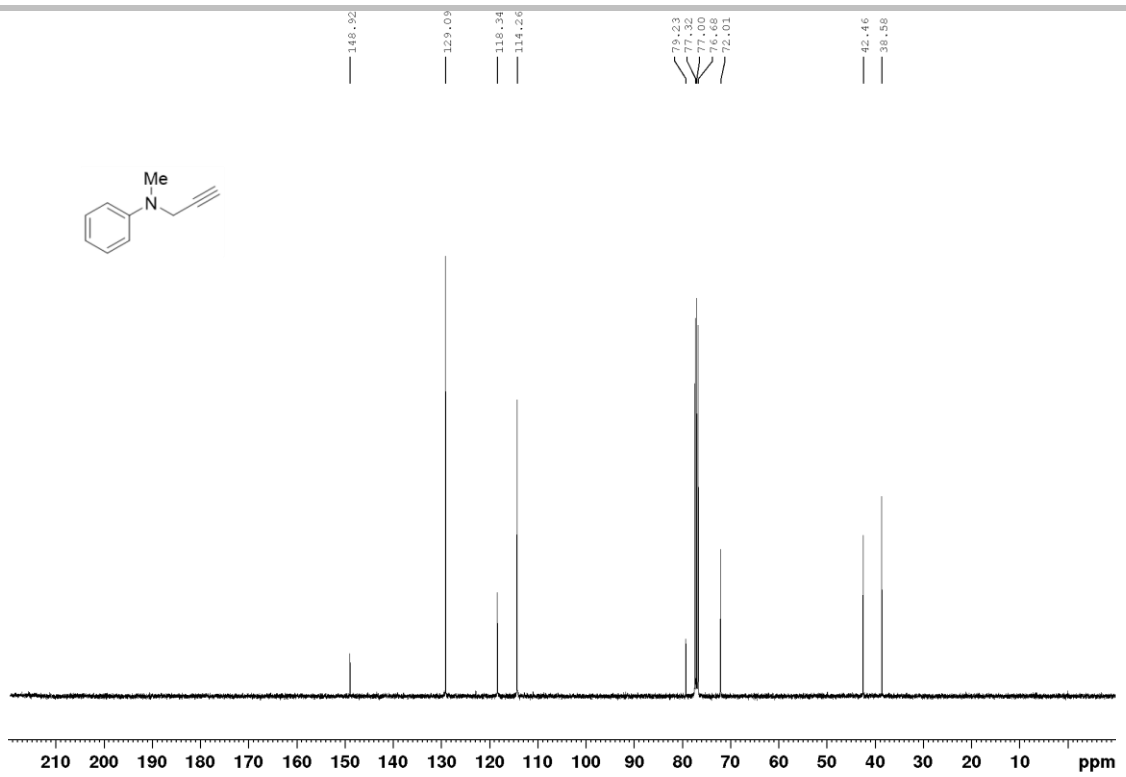
148.92
129.09
118.34
114.26
79.23
77.32
77.00
76.68
72.01
42.46
38.58
Me
N
210 200 190 180 170 160 150 140 130 120 110 100 90 80 70 60 50 40 30 20 10 ppm

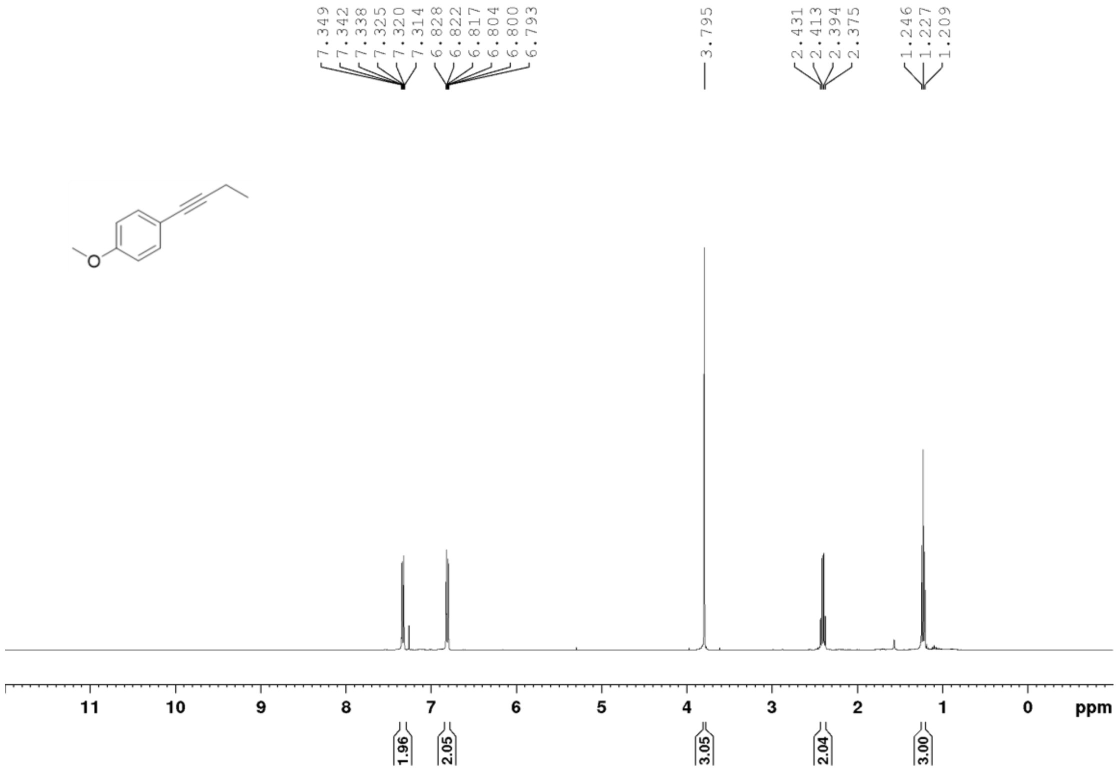
7.349
7.342
7.338
7.325
7.320
7.314
6.828
6.822
6.817
6.804
6.800
6.793
3.795
2.431
2.413
2.394
2.375
1.246
1.227
1.209
O
11 10 9 8 7 6 5 4 3 2 1 0 ppm
1.96
2.05
3.05
2.04
3.00

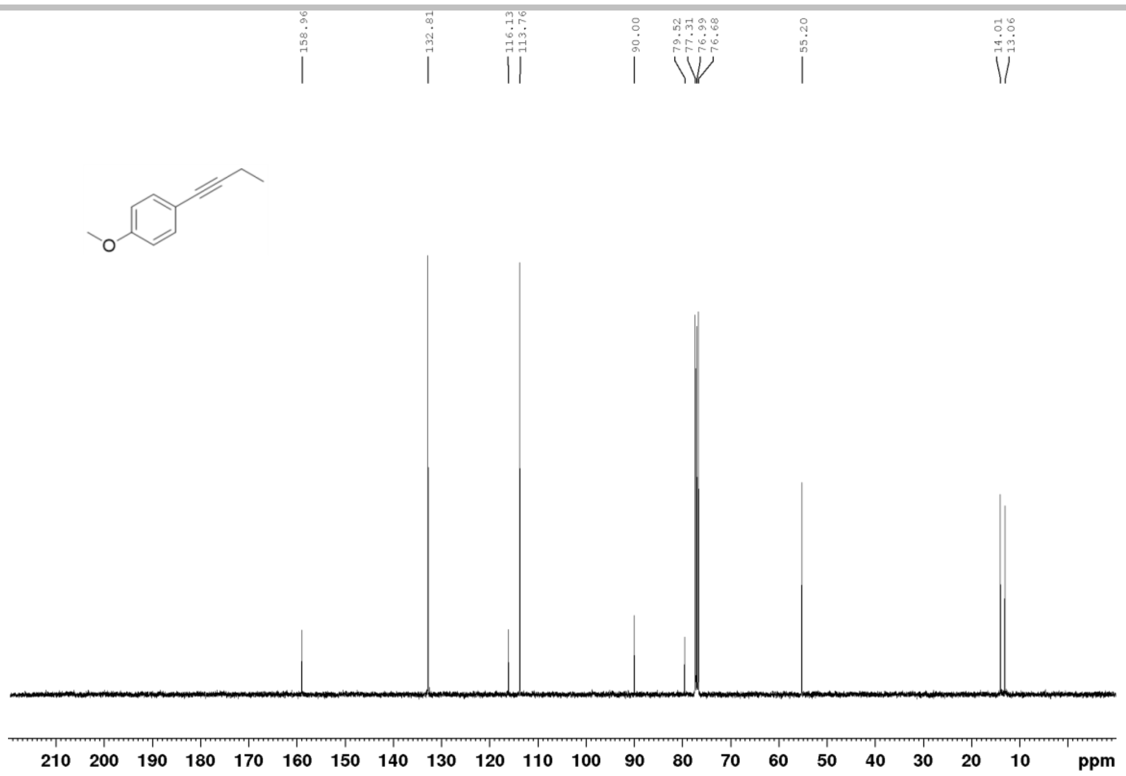
158.96
132.81
116.13
113.76
90.00
79.52
77.31
76.99
76.68
55.20
14.01
13.06
210 200 190 180 170 160 150 140 130 120 110 100 90 80 70 60 50 40 30 20 10 ppm

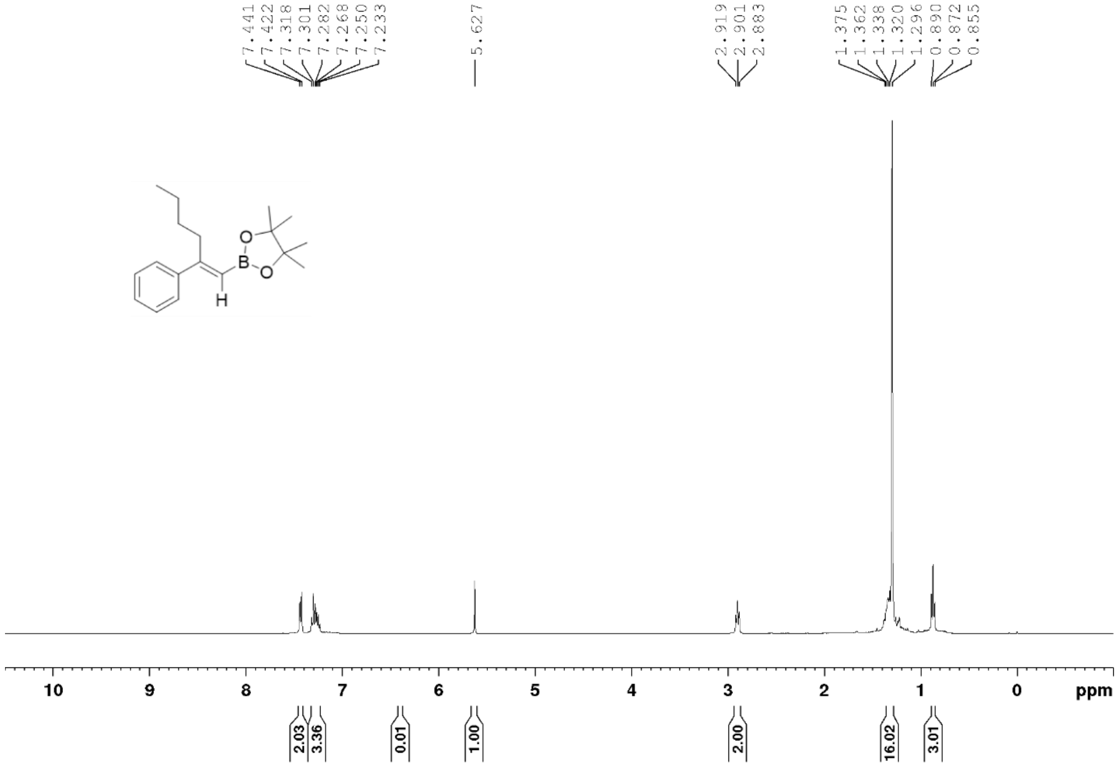
7.441
7.422
7.318
7.301
7.282
7.268
7.250
7.233
5.627
2.919
2.901
2.883
1.375
1.362
1.338
1.320
1.296
0.890
0.872
0.855
B
O
H
10 9 8 7 6 5 4 3 2 1 0 ppm
2.03
3.36
0.01
1.00
2.00
16.02
3.01

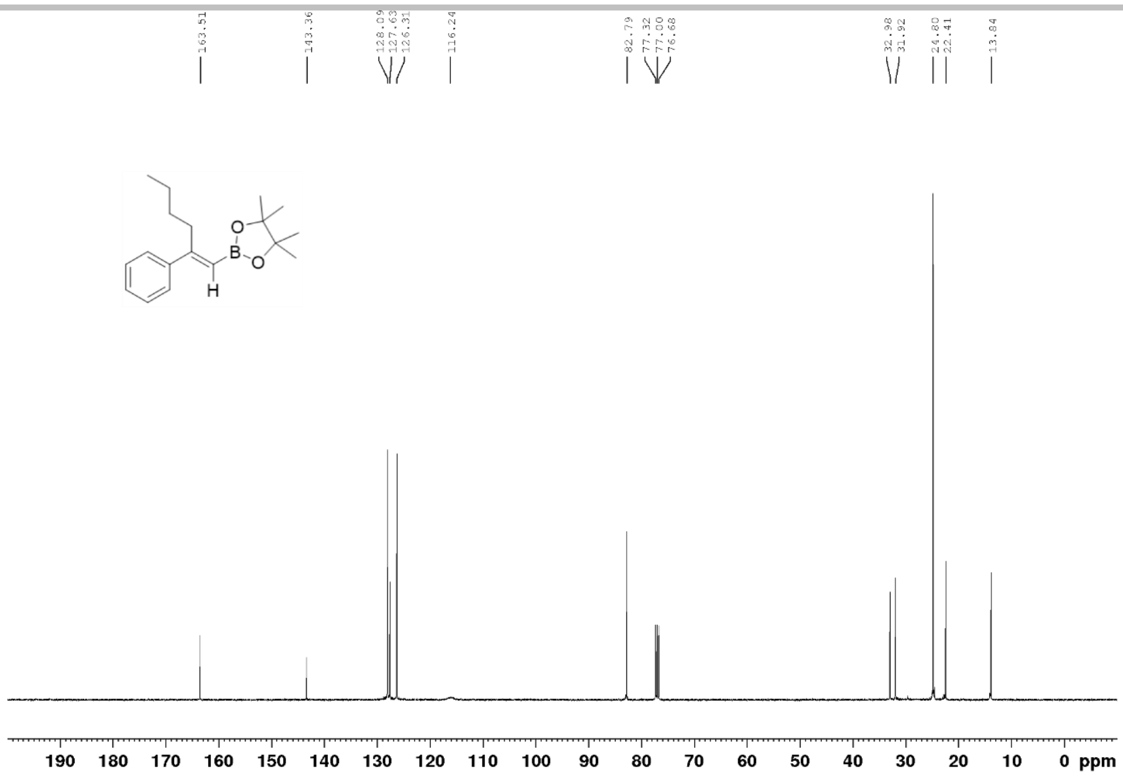
163.51
143.36
128.09
127.63
126.31
116.24
82.79
77.32
77.00
76.68
32.98
31.92
24.80
22.41
13.84
190 180 170 160 150 140 130 120 110 100 90 80 70 60 50 40 30 20 10 0 ppm

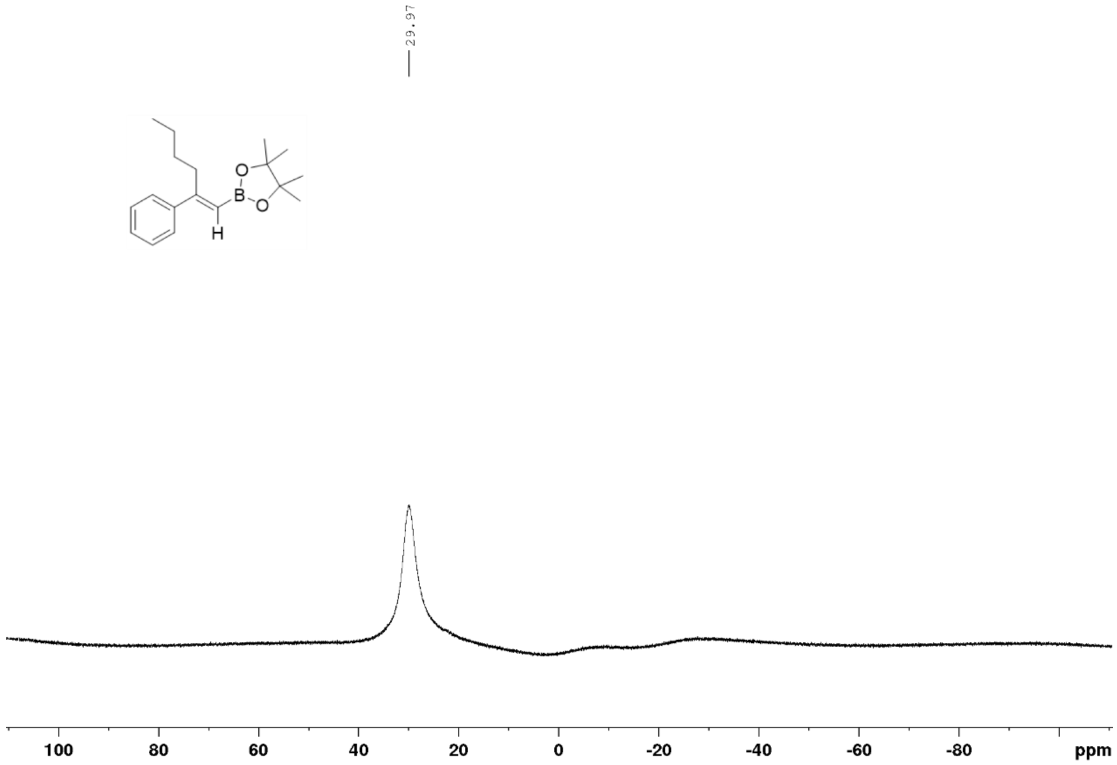
29.97
100 80 60 40 20 0 -20 -40 -60 -80 ppm

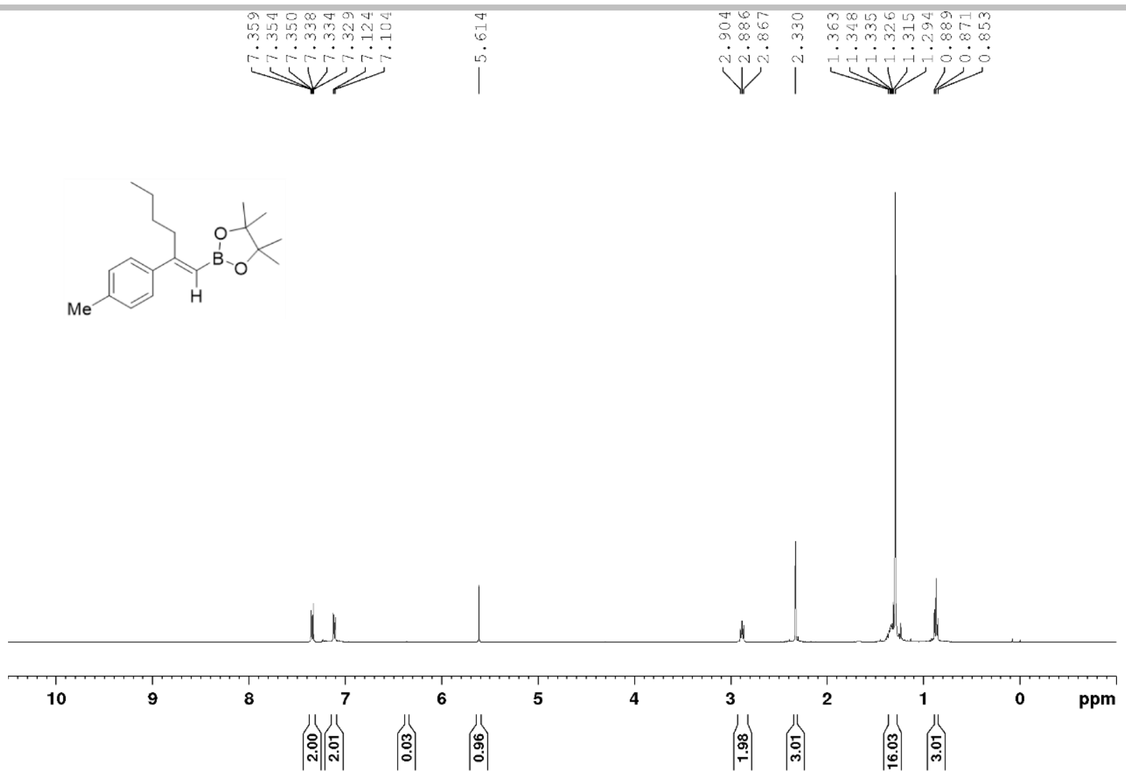
7.359
7.354
7.350
7.338
7.334
7.329
7.124
7.104
5.614
2.904
2.886
2.867
2.330
1.363
1.348
1.335
1.326
1.315
1.294
0.889
0.871
0.853
Me
H
B
O
O
10 9 8 7 6 5 4 3 2 1 0 ppm
2.00
2.01
0.03
0.96
1.98
3.01
16.03
3.01

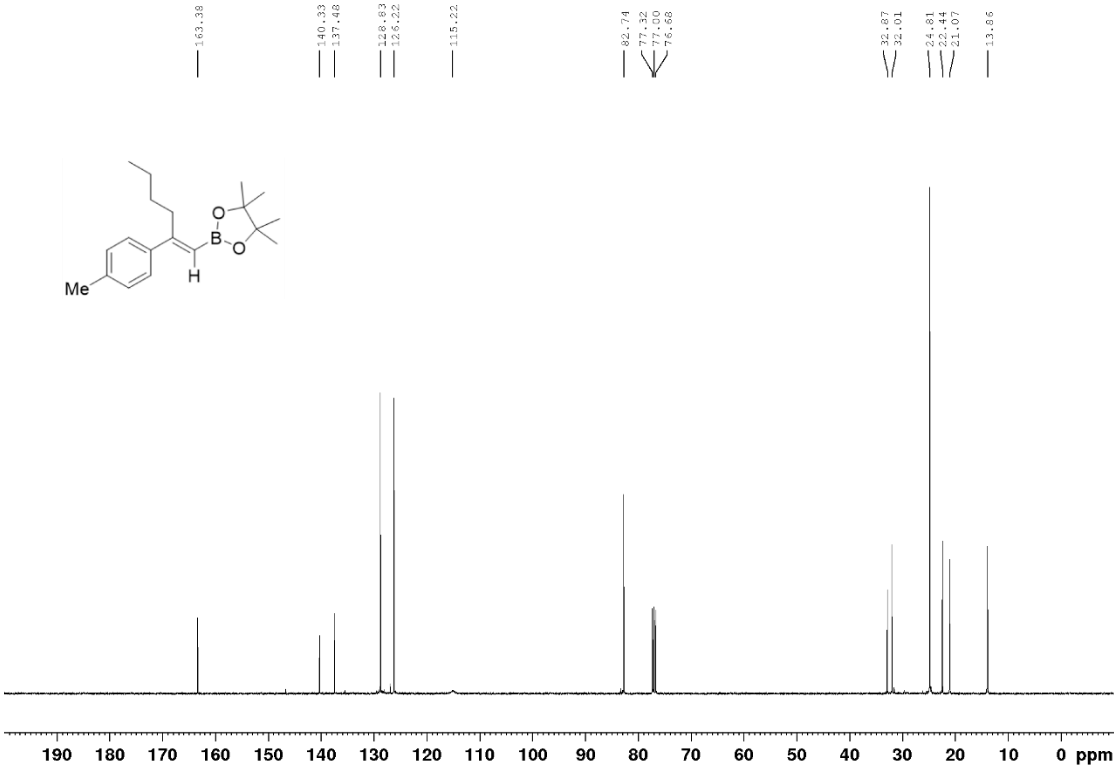
163.38
140.33
137.48
128.83
126.22
115.22
82.74
77.32
77.00
76.68
32.87
32.01
24.81
22.44
21.07
13.86
Me
H
B
O
O
190 180 170 160 150 140 130 120 110 100 90 80 70 60 50 40 30 20 10 0 ppm

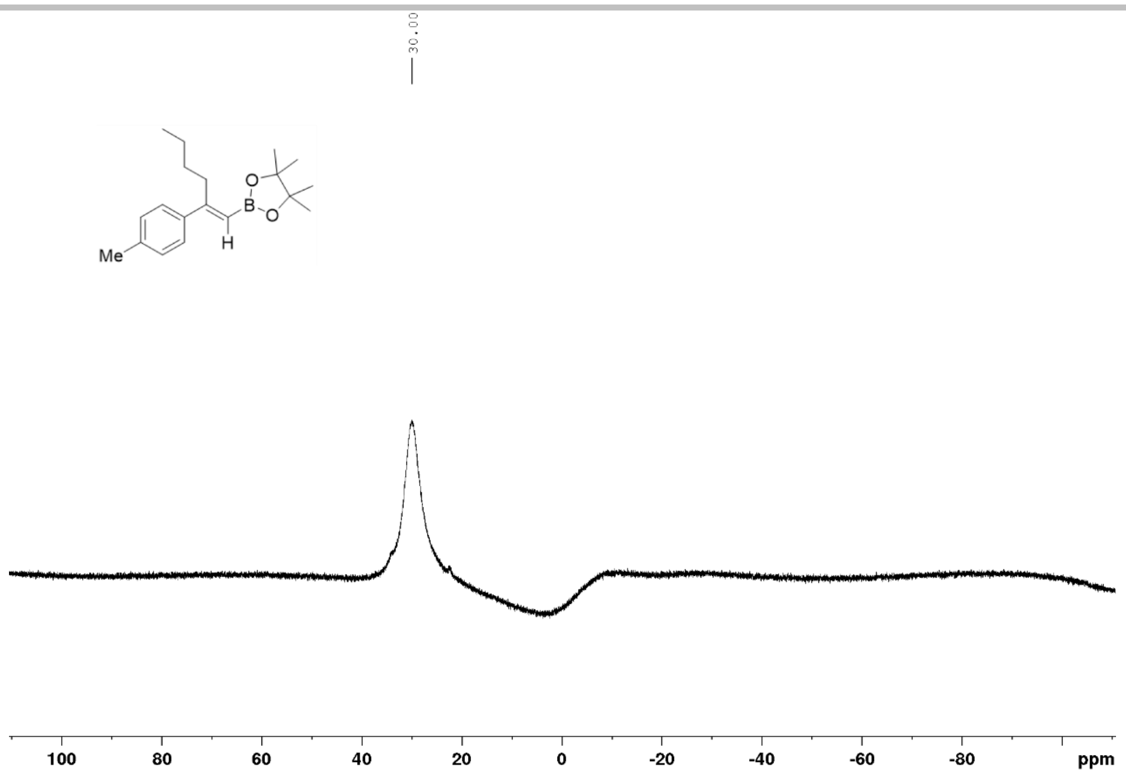
30.00
Me
B
O
O
H
100
80
60
40
20
0
-20
-40
-60
-80
ppm

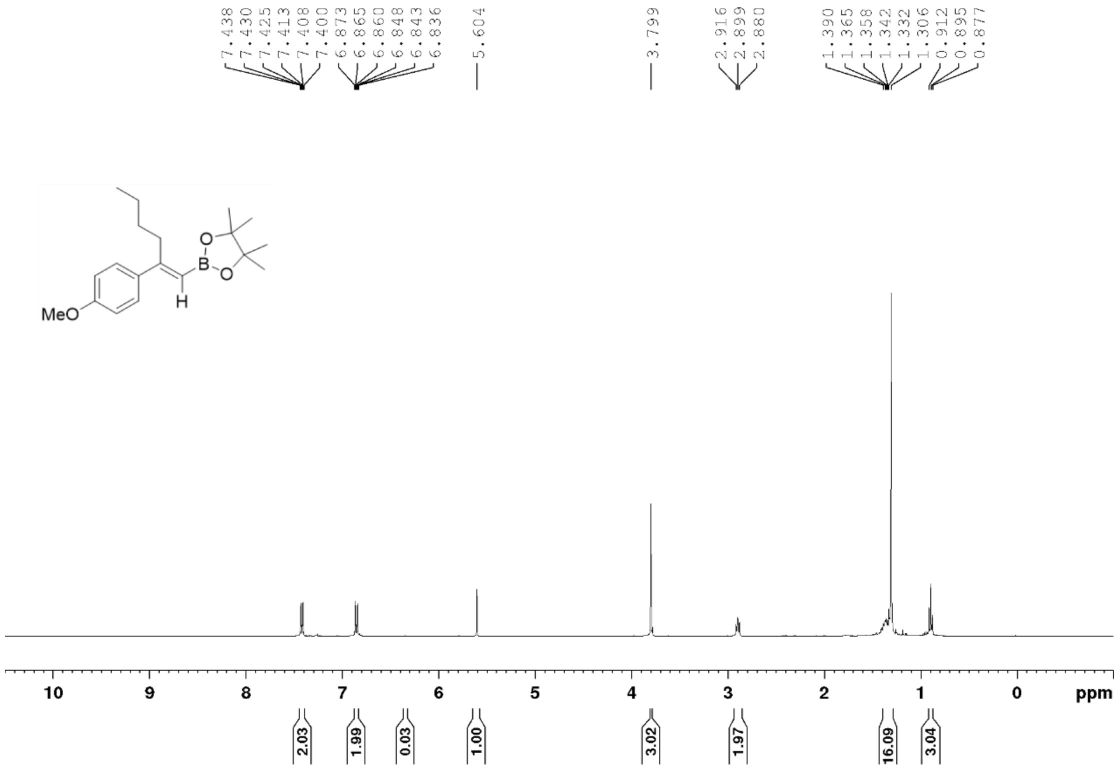
7.438
7.430
7.425
7.413
7.408
7.400
6.873
6.865
6.860
6.848
6.843
6.836
5.604
3.799
2.916
2.899
2.880
1.390
1.365
1.358
1.342
1.332
1.306
0.912
0.895
0.877
MeO
B
O
O
H
10
9
8
7
6
5
4
3
2
1
0
ppm
2.03
1.99
0.03
1.00
3.02
1.97
16.09
3.04

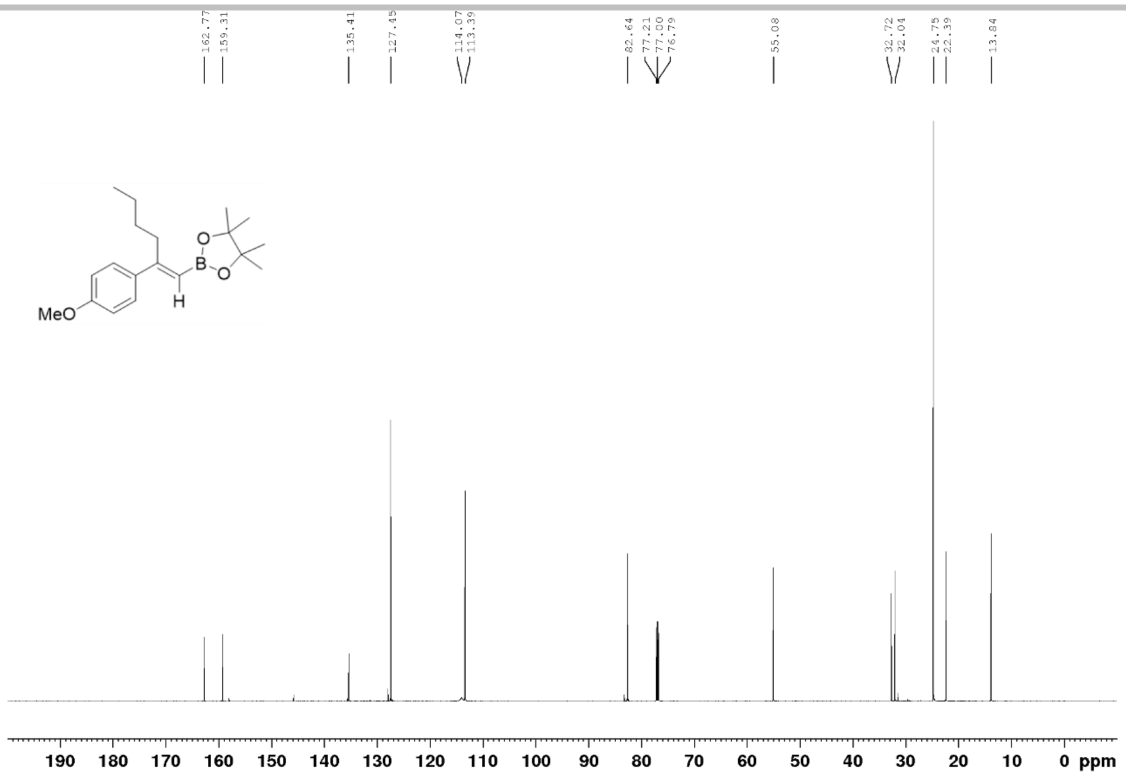
162.77
159.31
135.41
127.45
114.07
113.39
82.64
77.21
77.00
76.79
55.08
32.72
32.04
24.75
22.39
13.84
MeO
B
O
O
H
190 180 170 160 150 140 130 120 110 100 90 80 70 60 50 40 30 20 10 0 ppm

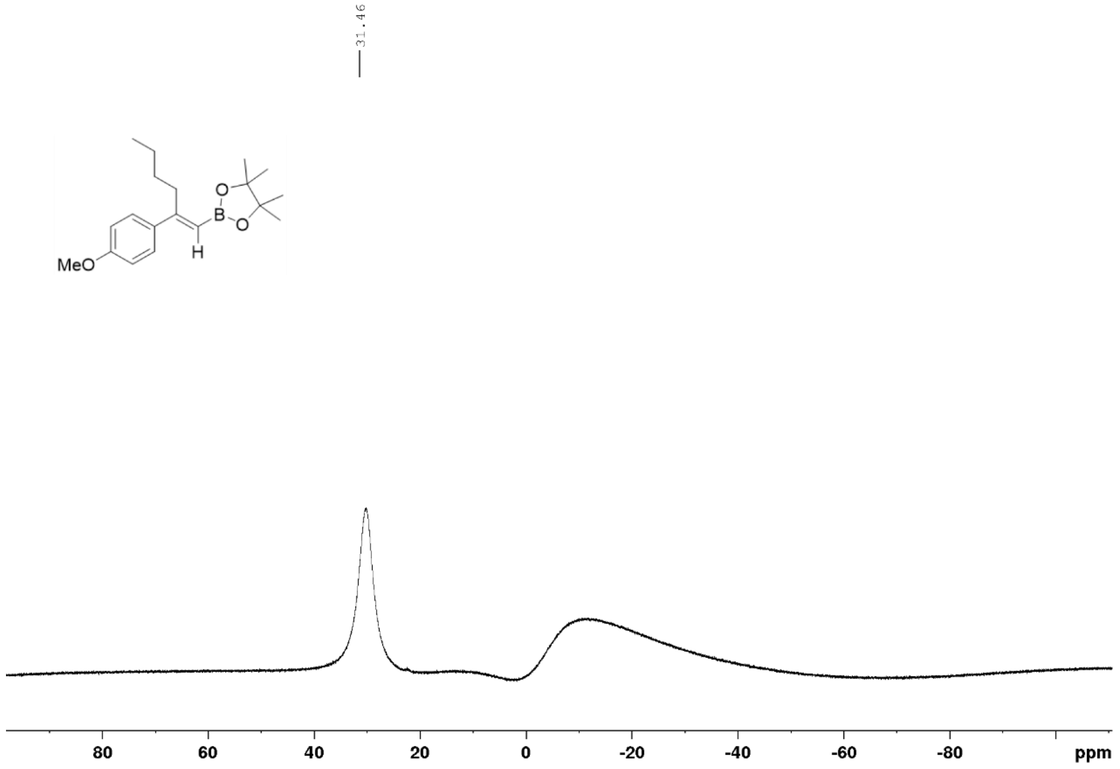
31.46
MeO
B
O
O
H
80 60 40 20 0 -20 -40 -60 -80 ppm

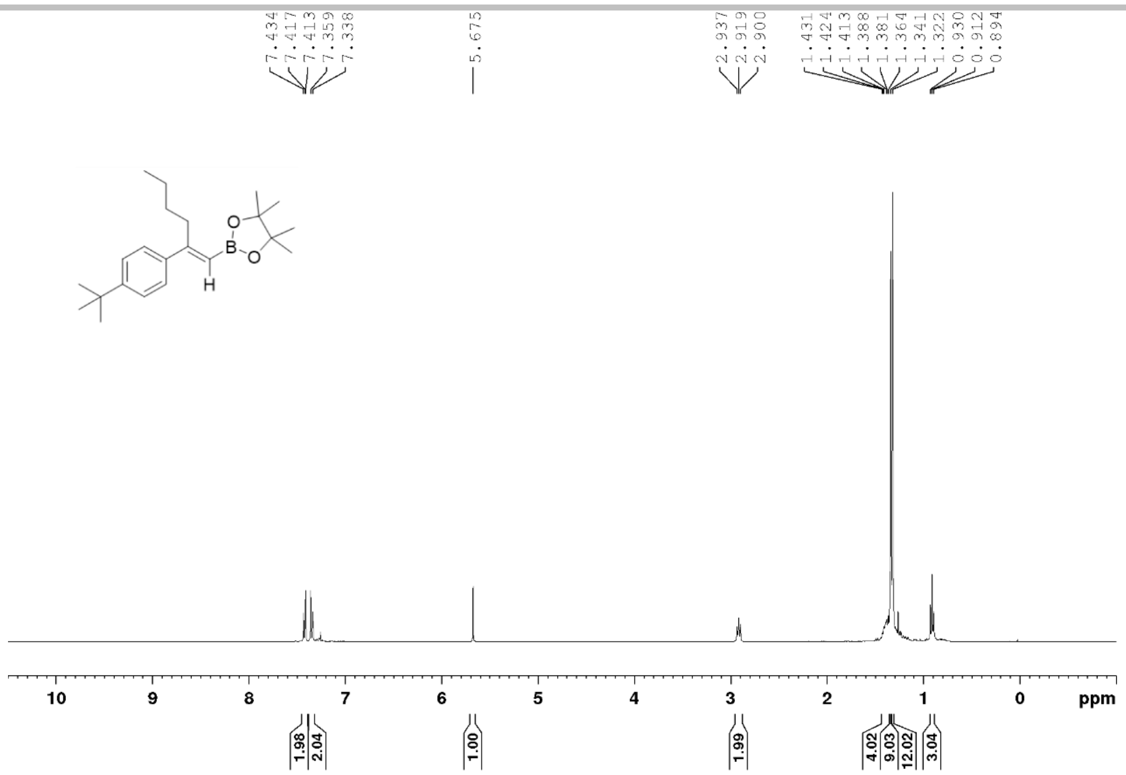
7.434
7.417
7.413
7.359
7.338
5.675
2.937
2.919
2.900
1.431
1.424
1.413
1.388
1.381
1.364
1.341
1.322
0.930
0.912
0.894
H
O
B
O
10
9
8
7
6
5
4
3
2
1
0
ppm
1.98
2.04
1.00
1.99
4.02
9.03
12.02
3.04

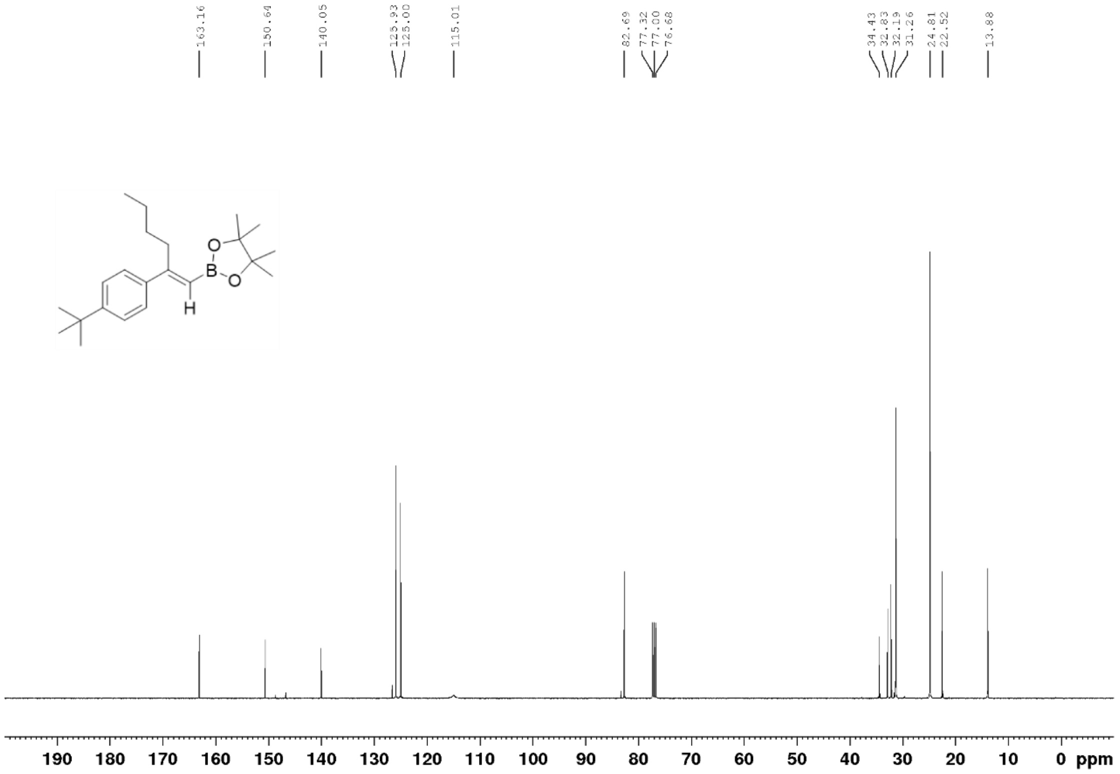
163.16
150.64
140.05
125.93
125.00
115.01
82.69
77.32
77.00
76.68
34.43
32.83
32.19
31.26
24.81
22.52
13.88
H
O
B
O
190
180
170
160
150
140
130
120
110
100
90
80
70
60
50
40
30
20
10
0
ppm

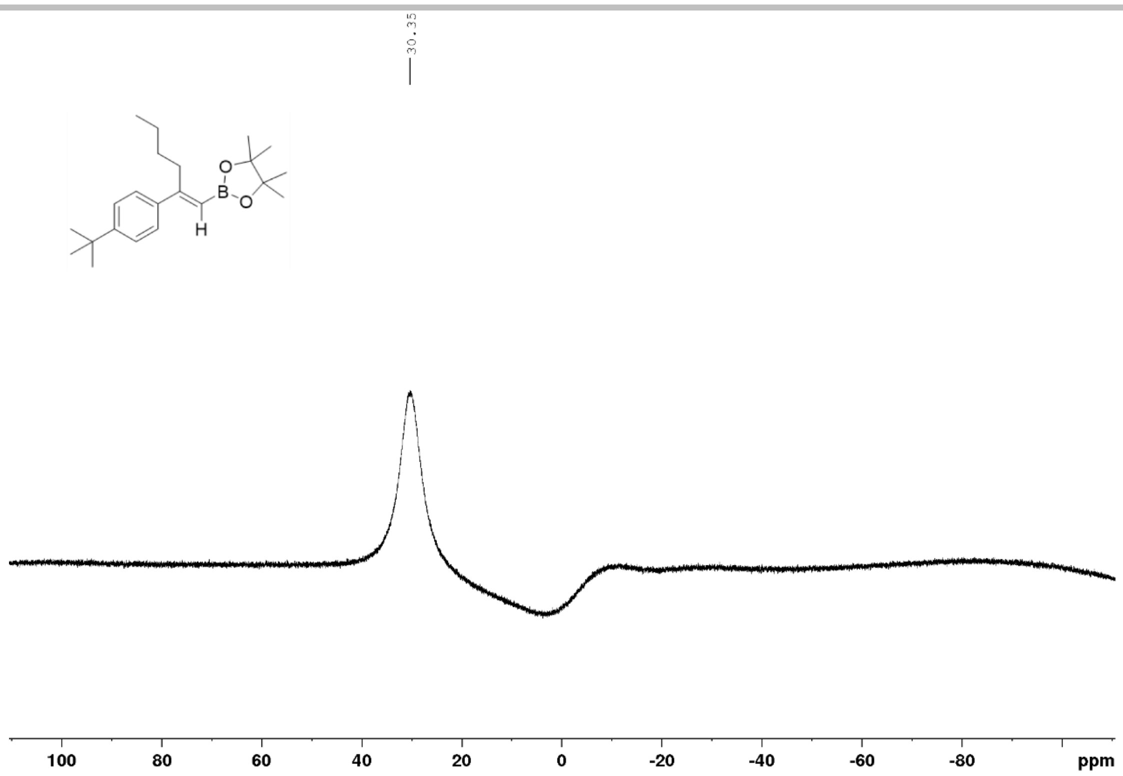
30.35
O
B
O
H
100
80
60
40
20
0
-20
-40
-60
-80
ppm

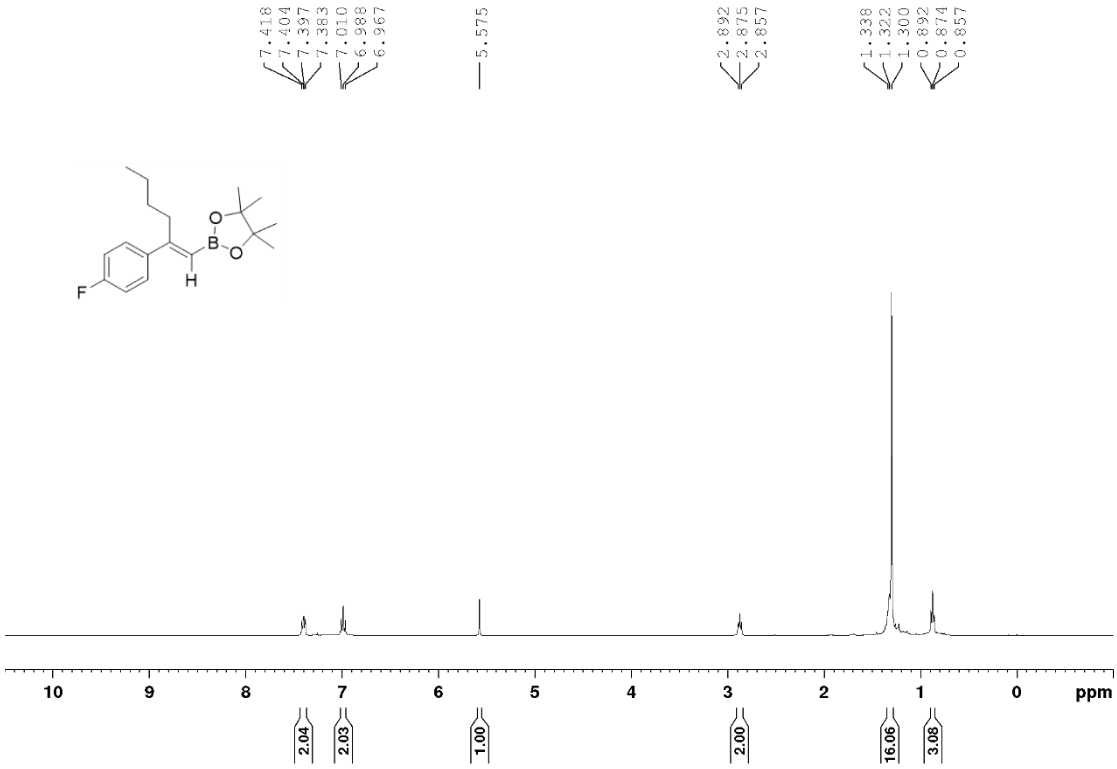
7.418
7.404
7.397
7.383
7.010
6.988
6.967
5.575
2.892
2.875
2.857
1.338
1.322
1.300
0.892
0.874
0.857
O
B
O
F
H
10
9
8
7
6
5
4
3
2
1
0
ppm
2.04
2.03
1.00
2.00
16.06
3.08

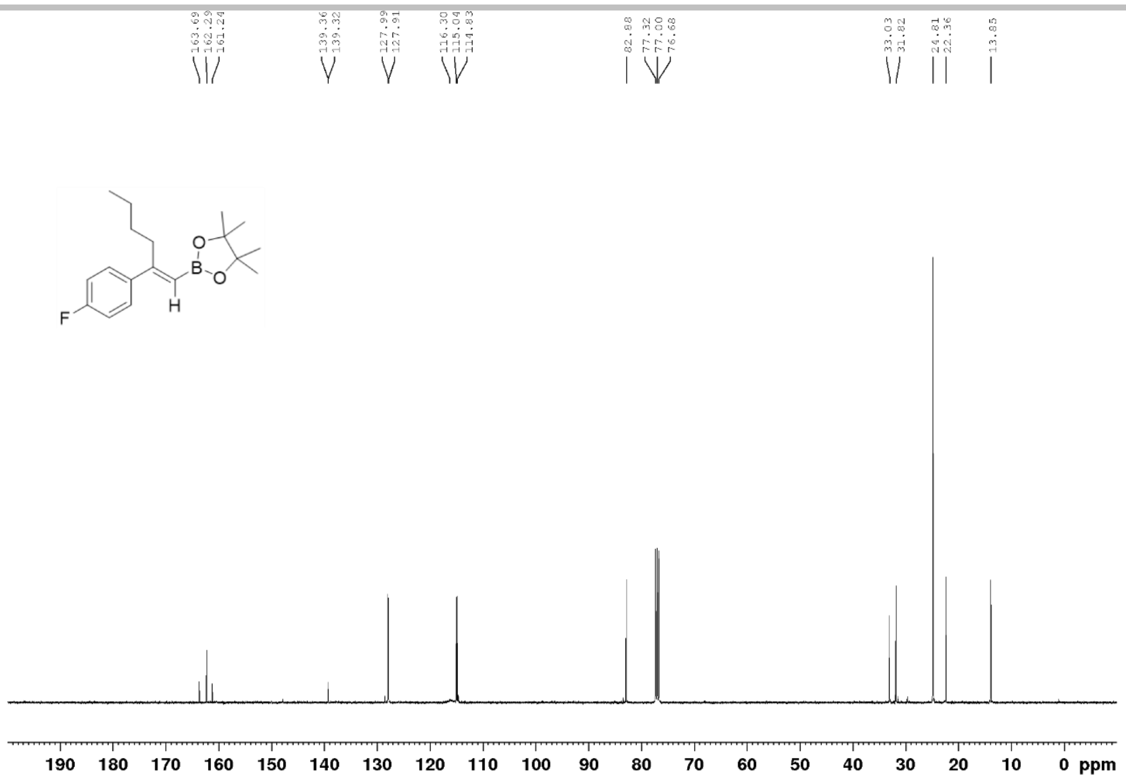
163.69
162.29
161.24
139.36
139.32
127.99
127.91
116.30
115.04
114.83
82.88
77.32
77.00
76.68
33.03
31.82
24.81
22.36
13.85
O
B
O
H
F
190 180 170 160 150 140 130 120 110 100 90 80 70 60 50 40 30 20 10 0 ppm

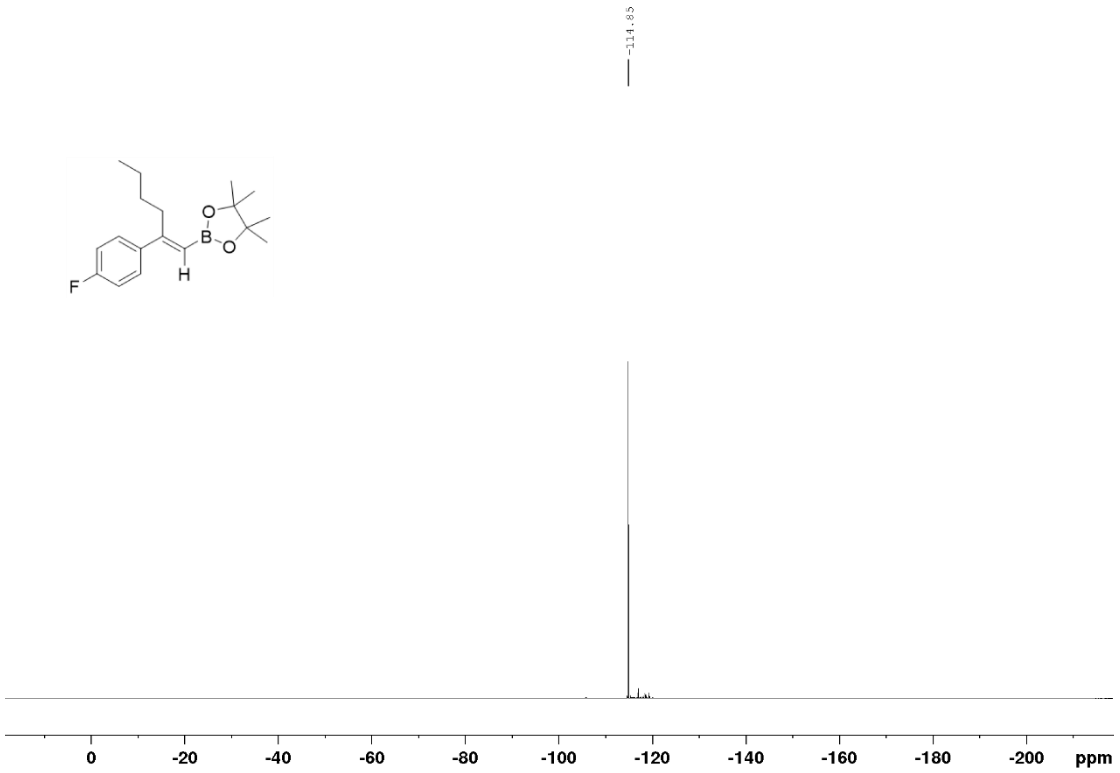
-114.85
O
B
O
H
F
0 -20 -40 -60 -80 -100 -120 -140 -160 -180 -200 ppm

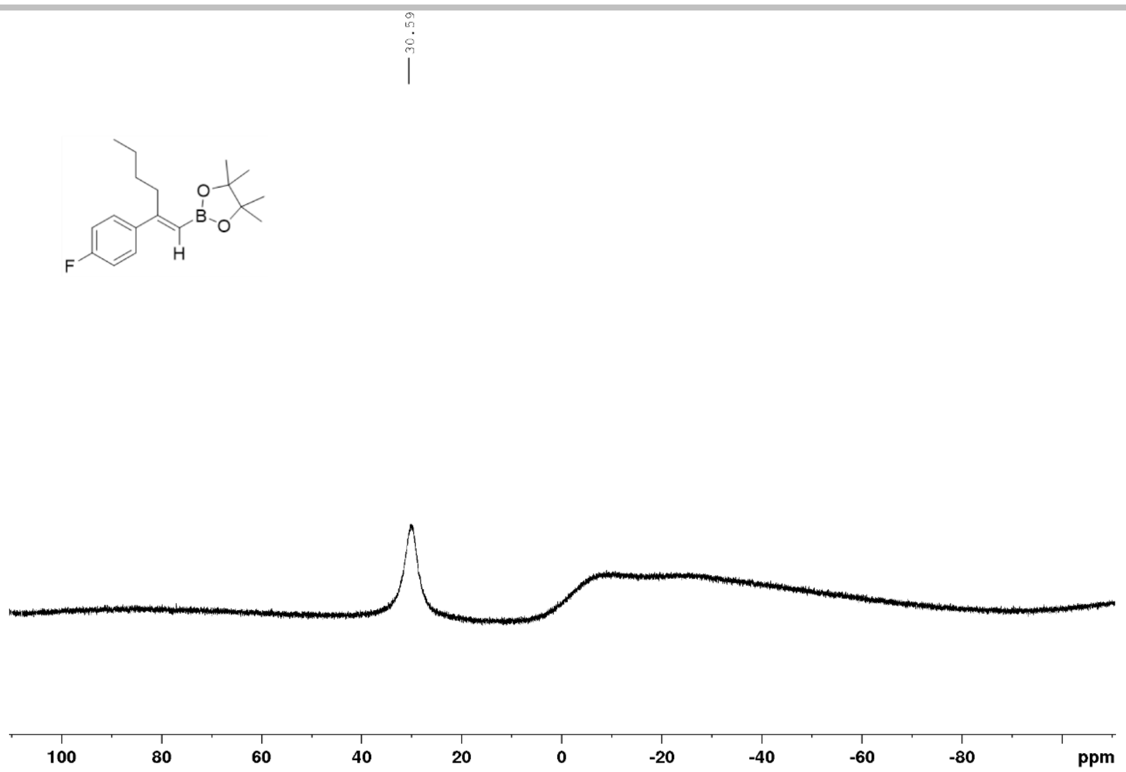
30.59
F
O
B
O
H
100
80
60
40
20
0
-20
-40
-60
-80
ppm

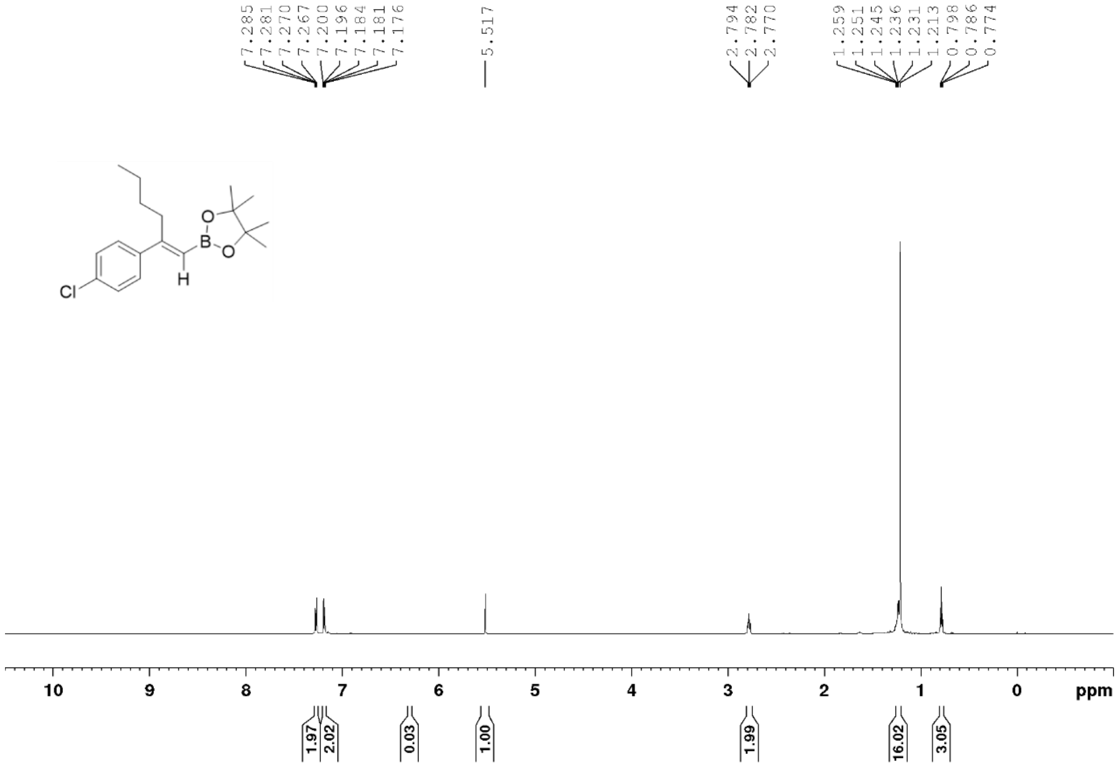
7.285
7.281
7.270
7.267
7.200
7.196
7.184
7.181
7.176
5.517
2.794
2.782
2.770
1.259
1.251
1.245
1.236
1.231
1.213
0.798
0.786
0.774
Cl
O
B
O
H
10
9
8
7
6
5
4
3
2
1
0
ppm
1.97
2.02
0.03
1.00
1.99
16.02
3.05

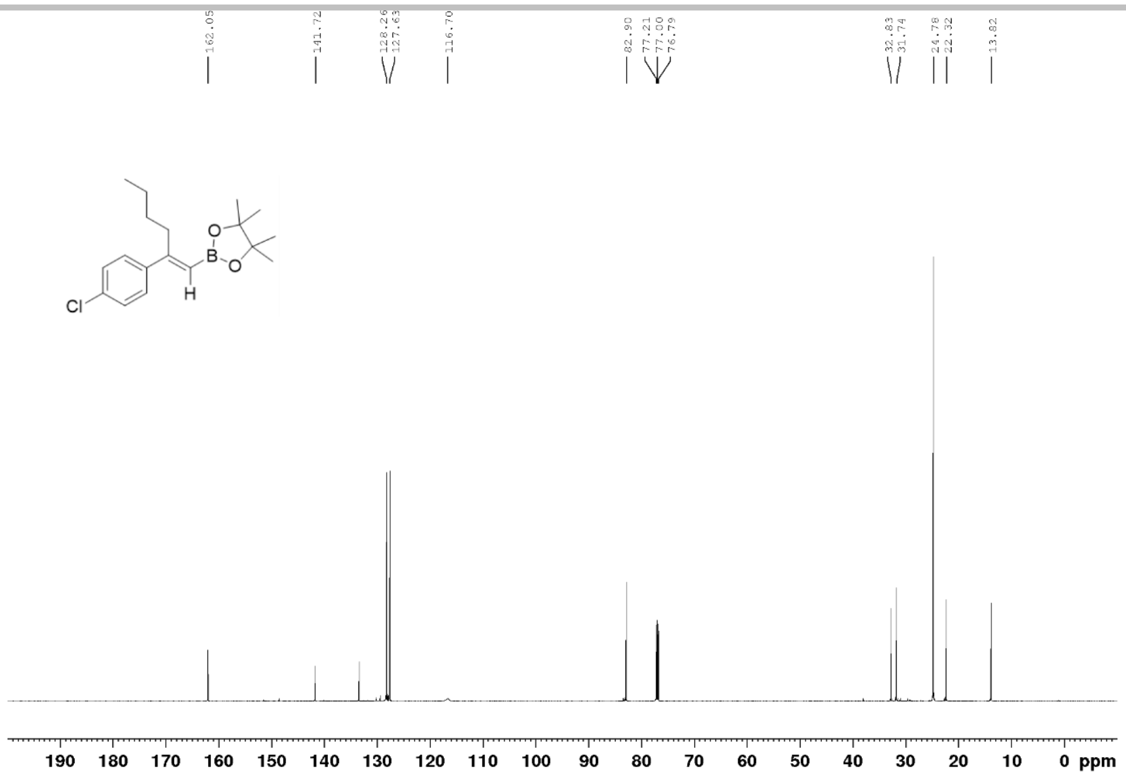
162.05
141.72
128.26
127.63
116.70
82.90
77.21
77.00
76.79
32.83
31.74
24.70
22.32
13.82
Cl
B
O
O
H
190
180
170
160
150
140
130
120
110
100
90
80
70
60
50
40
30
20
10
0
ppm

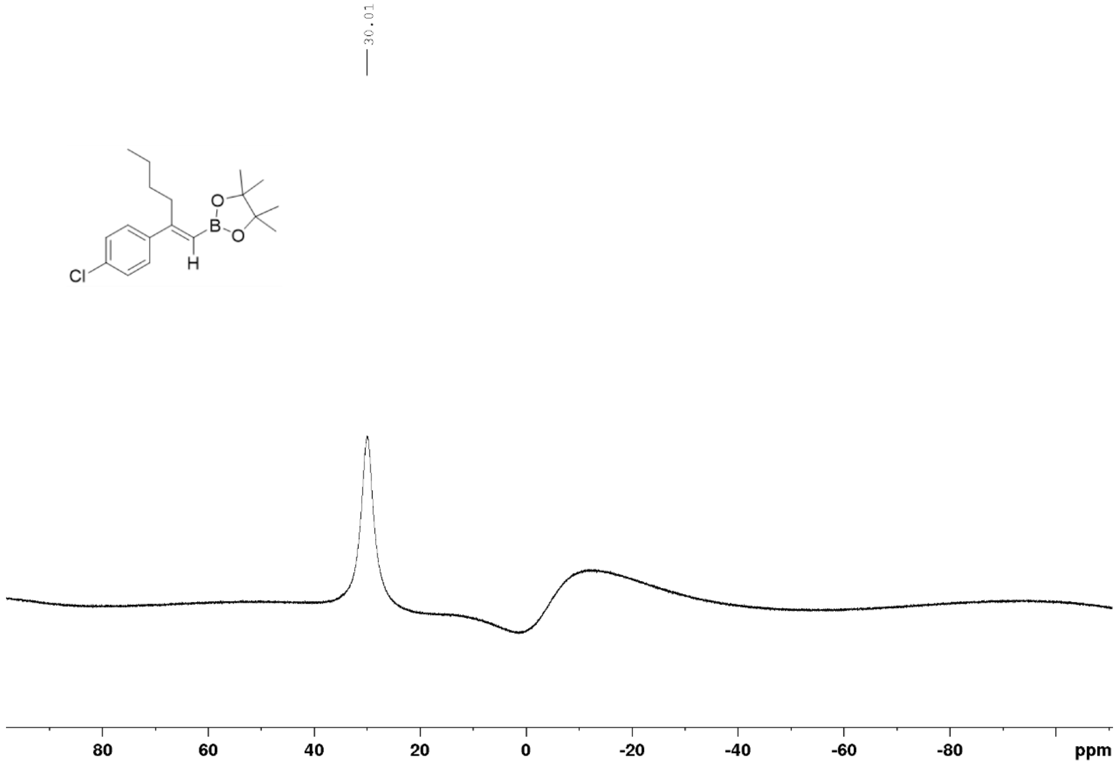
30.01
Cl
B
O
O
H
80
60
40
20
0
-20
-40
-60
-80
ppm

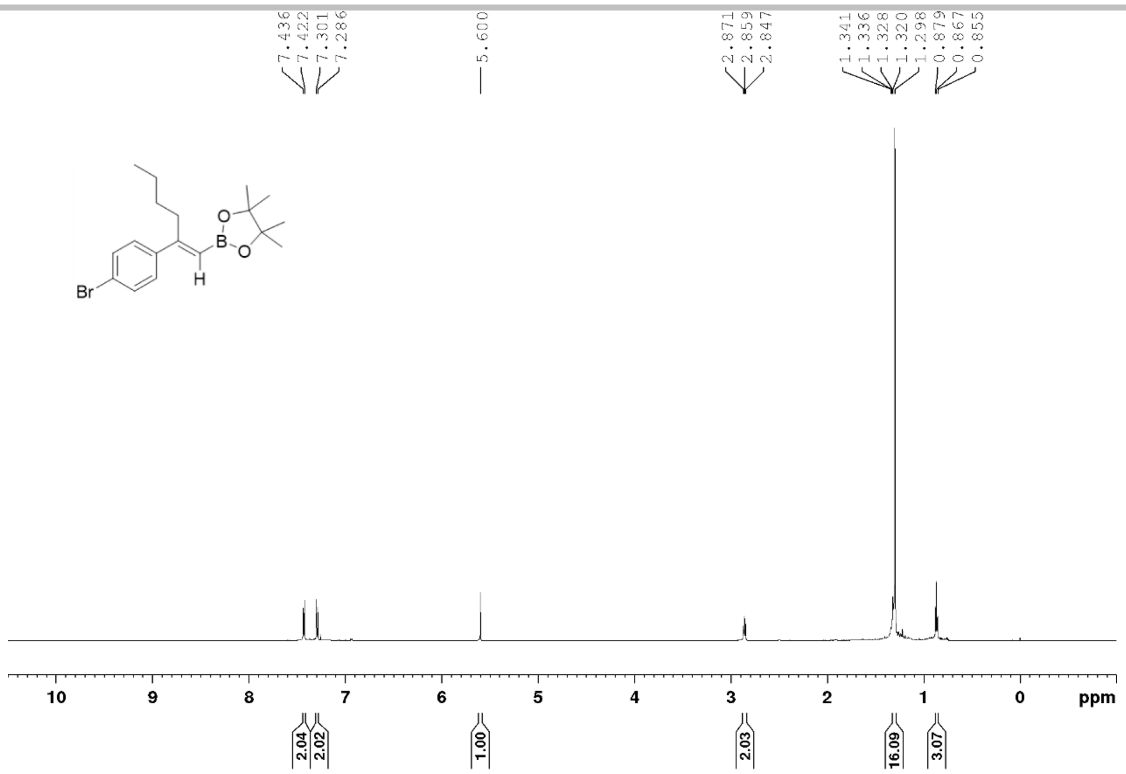
7.436
7.422
7.301
7.286
5.600
2.871
2.859
2.847
1.341
1.336
1.328
1.320
1.298
0.879
0.867
0.855
Br
O
B
O
H
10
9
8
7
6
5
4
3
2
1
0
ppm
2.04
2.02
1.00
2.03
16.09
3.07

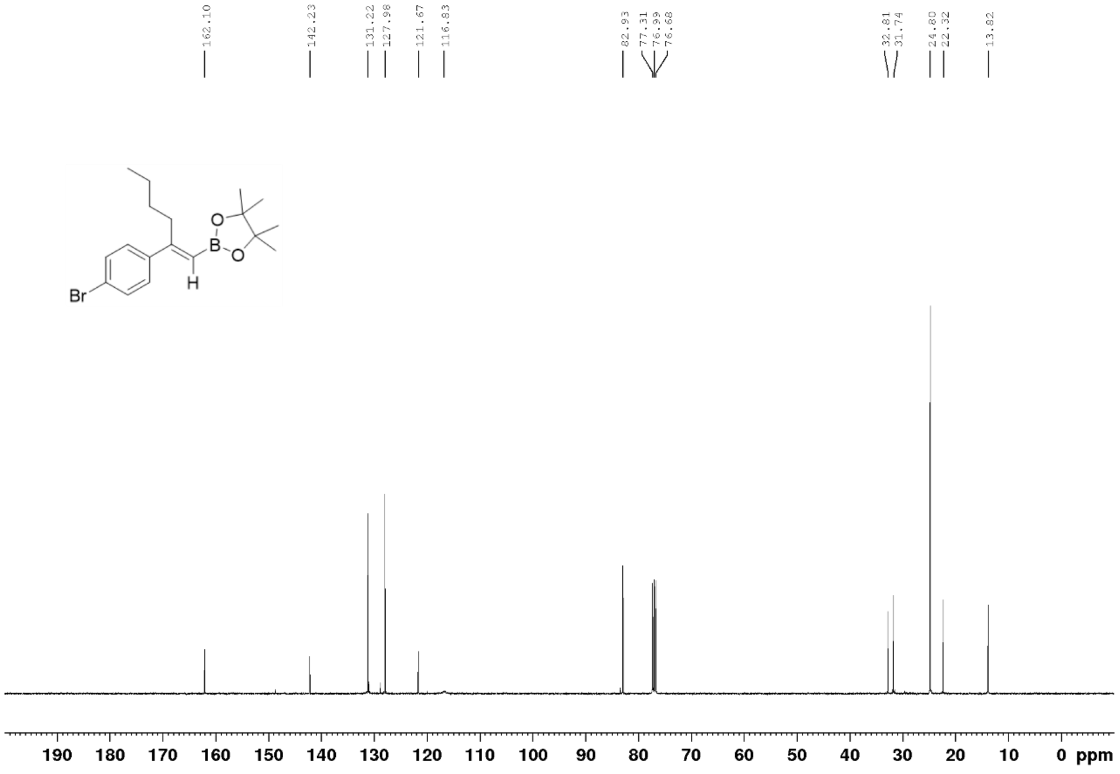
162.10
142.23
131.22
127.98
121.67
116.83
82.93
77.31
76.99
76.68
32.81
31.74
24.80
22.32
13.82
Br
O
B
O
H
190
180
170
160
150
140
130
120
110
100
90
80
70
60
50
40
30
20
10
0 ppm

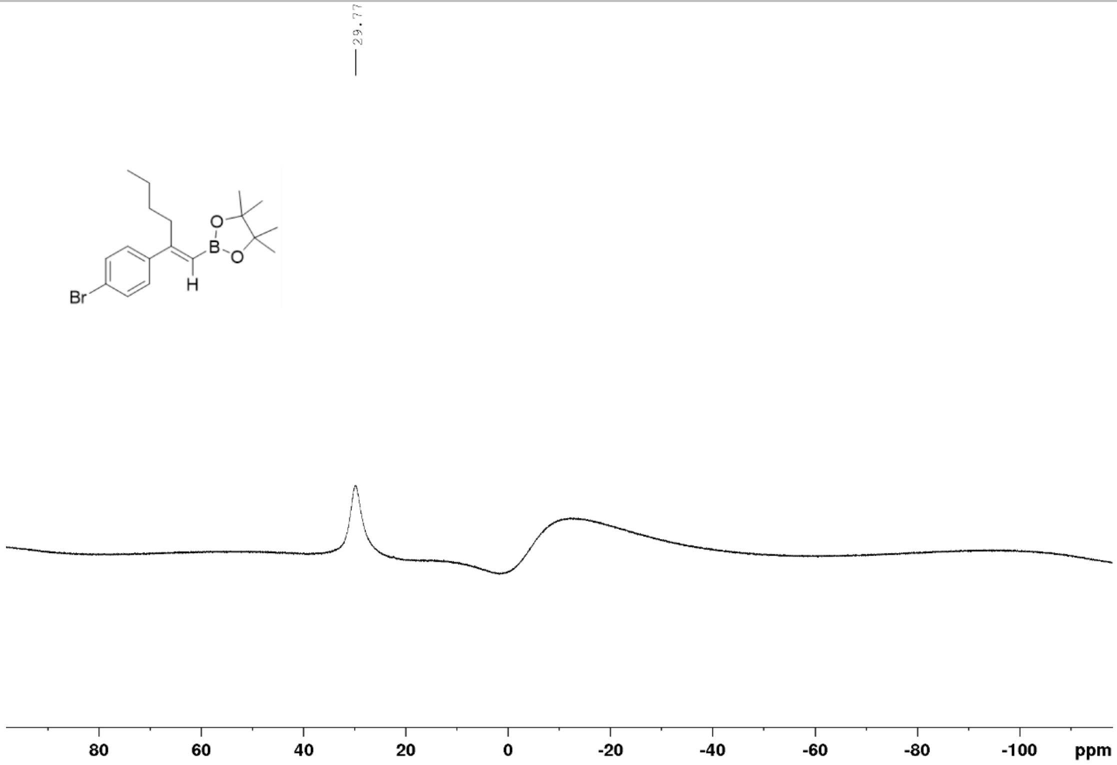
29.77
O
B
O
H
Br
80
60
40
20
0
-20
-40
-60
-80
-100
ppm

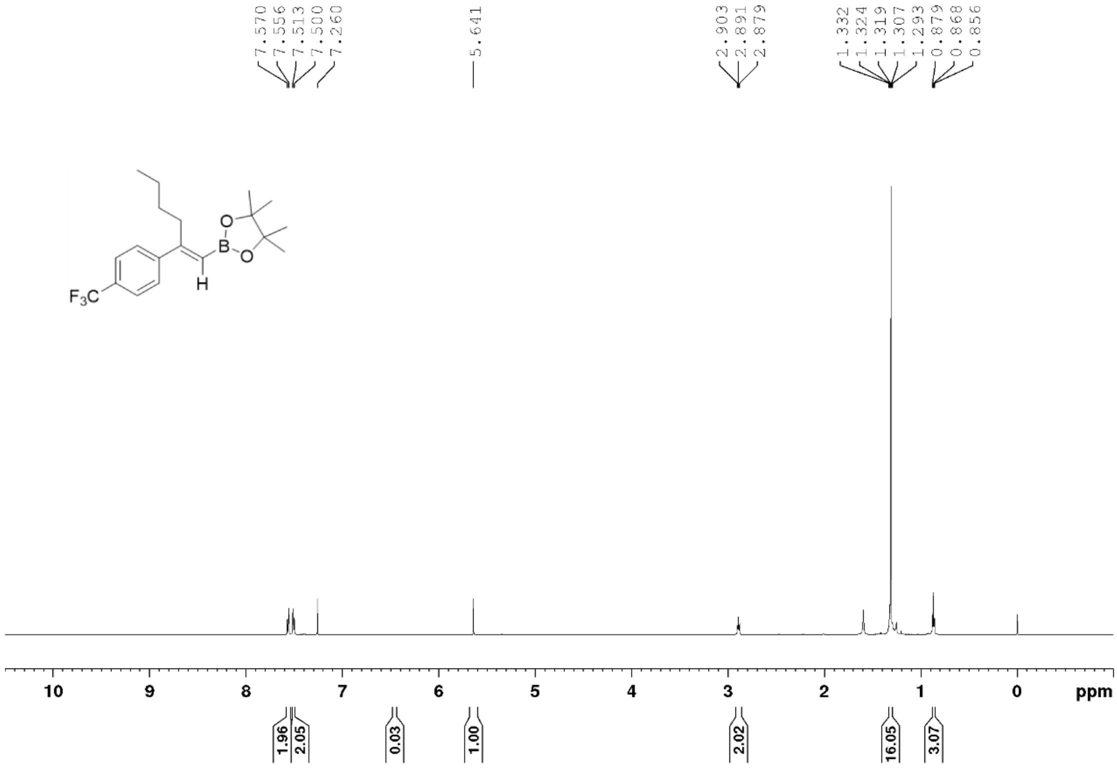
7.570
7.556
7.513
7.500
7.260
5.641
2.903
2.891
2.879
1.332
1.324
1.319
1.307
1.293
0.879
0.868
0.856
O
B
O
H
F_3C
10
9
8
7
6
5
4
3
2
1
0
ppm
1.96
2.05
0.03
1.00
2.02
16.05
3.07

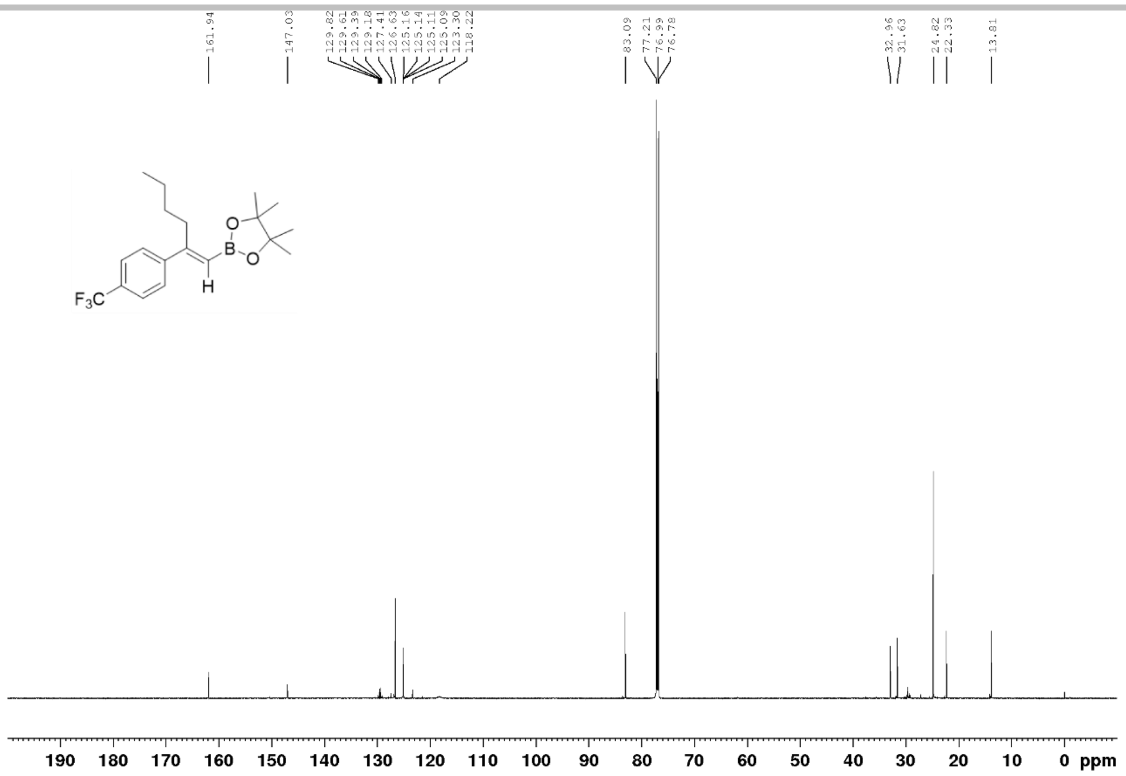
161.94
147.03
129.82
129.61
129.39
129.18
127.41
126.63
125.16
125.14
125.11
125.09
123.30
118.22
83.09
77.21
76.99
76.78
32.96
31.63
24.82
22.33
13.81
190
180
170
160
150
140
130
120
110
100
90
80
70
60
50
40
30
20
10
0
ppm

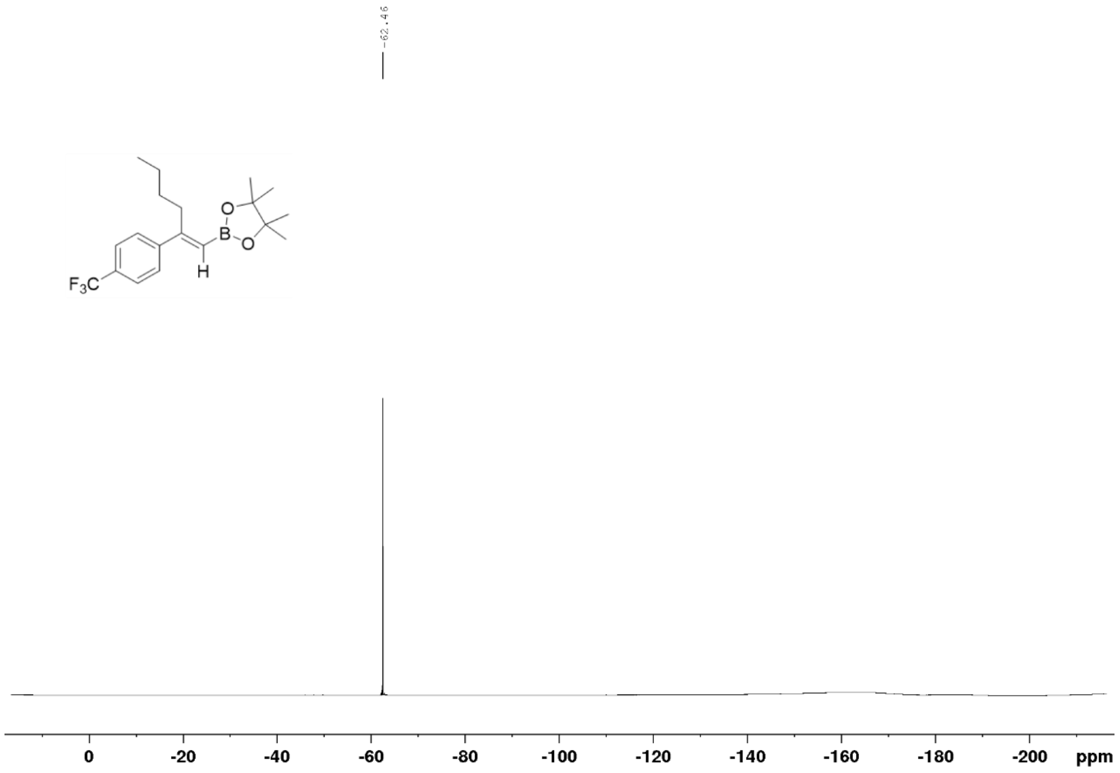
-62.46
0
-20
-40
-60
-80
-100
-120
-140
-160
-180
-200
ppm

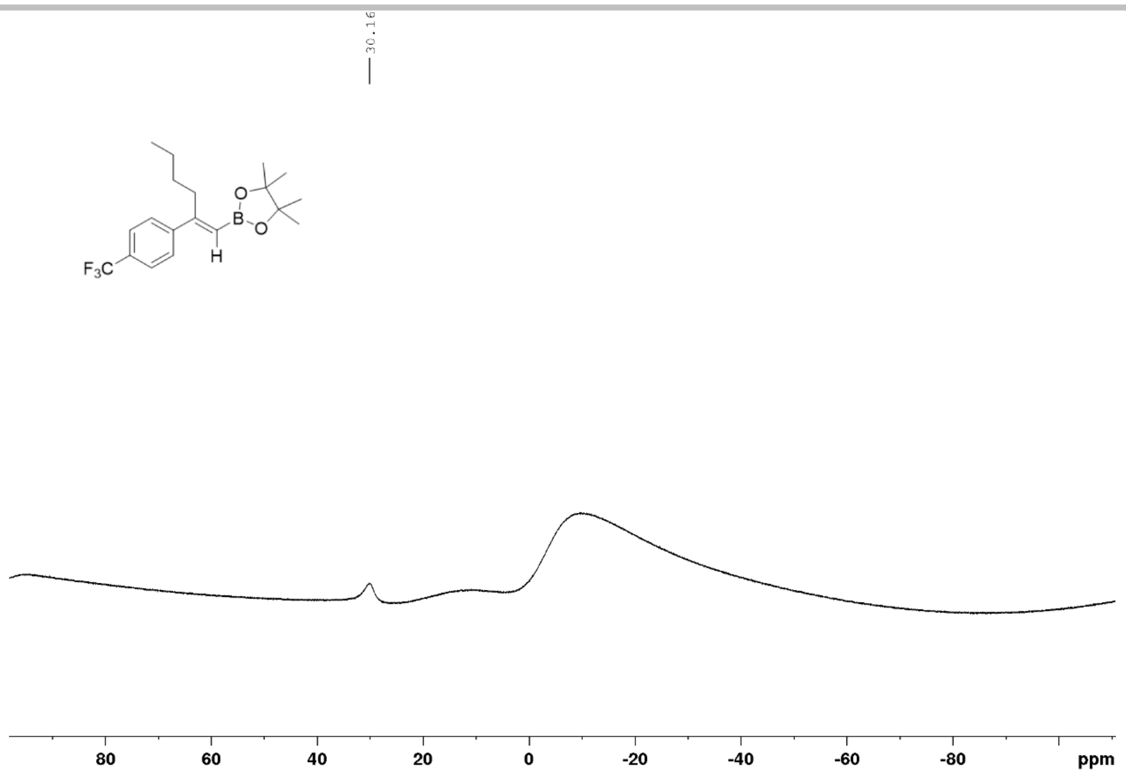
30.16
F3C
O
B
O
H
80
60
40
20
0
-20
-40
-60
-80
ppm

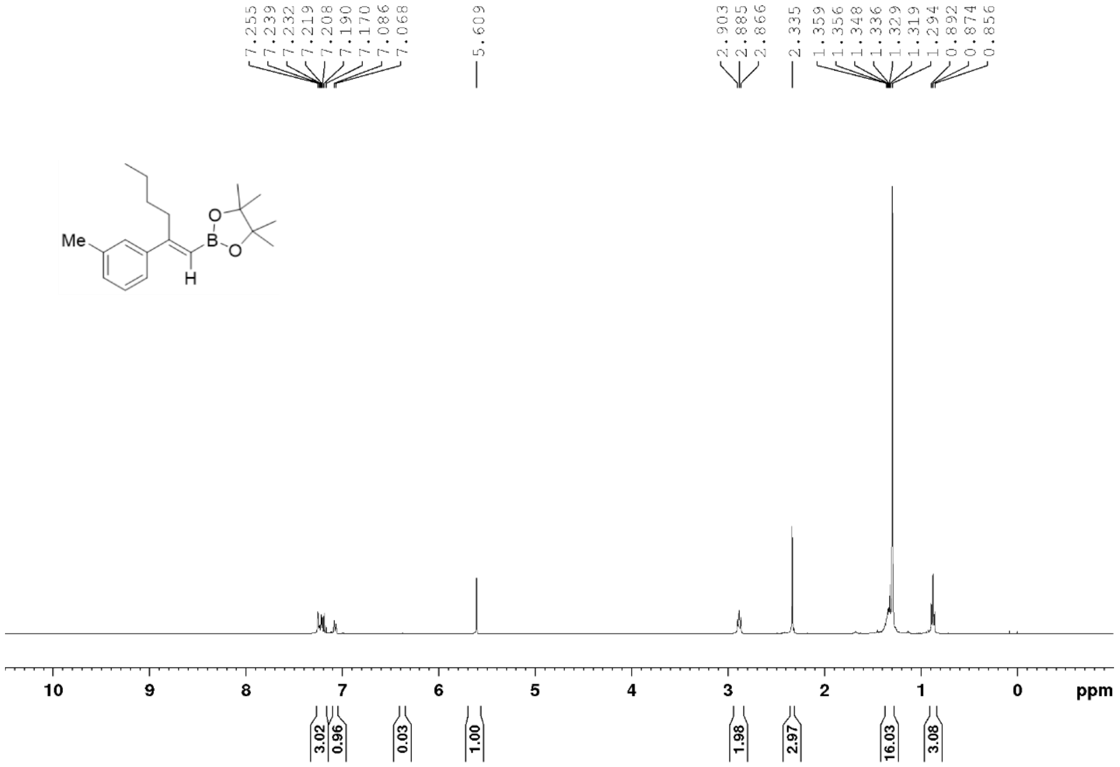
7.255
7.239
7.232
7.219
7.208
7.190
7.170
7.086
7.068
5.609
2.903
2.885
2.866
2.335
1.359
1.356
1.348
1.336
1.329
1.319
1.294
0.892
0.874
0.856
Me
O
B
O
H
10
9
8
7
6
5
4
3
2
1
0
ppm
3.02
0.96
0.03
1.00
1.98
2.97
16.03
3.08

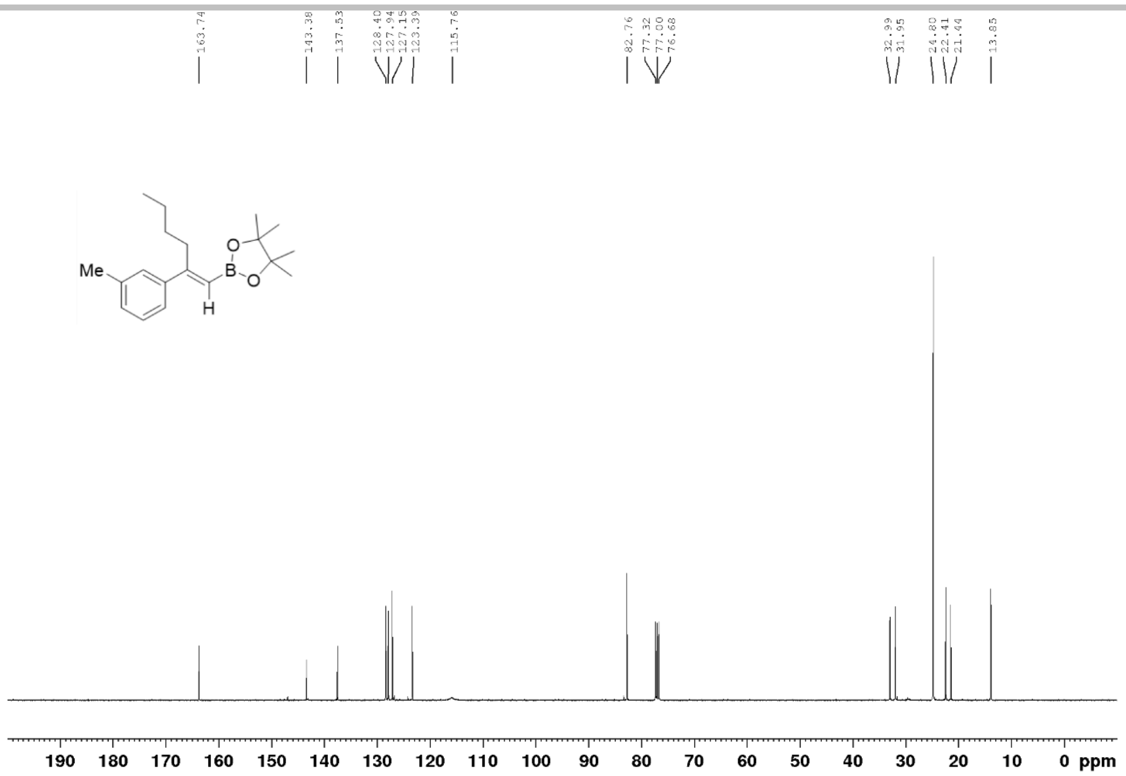
163.74
143.38
137.53
128.40
127.94
127.15
123.39
115.76
82.76
77.32
77.00
76.68
32.99
31.95
24.80
22.41
21.44
13.85
Me
O
B
O
H
190 180 170 160 150 140 130 120 110 100 90 80 70 60 50 40 30 20 10 0 ppm

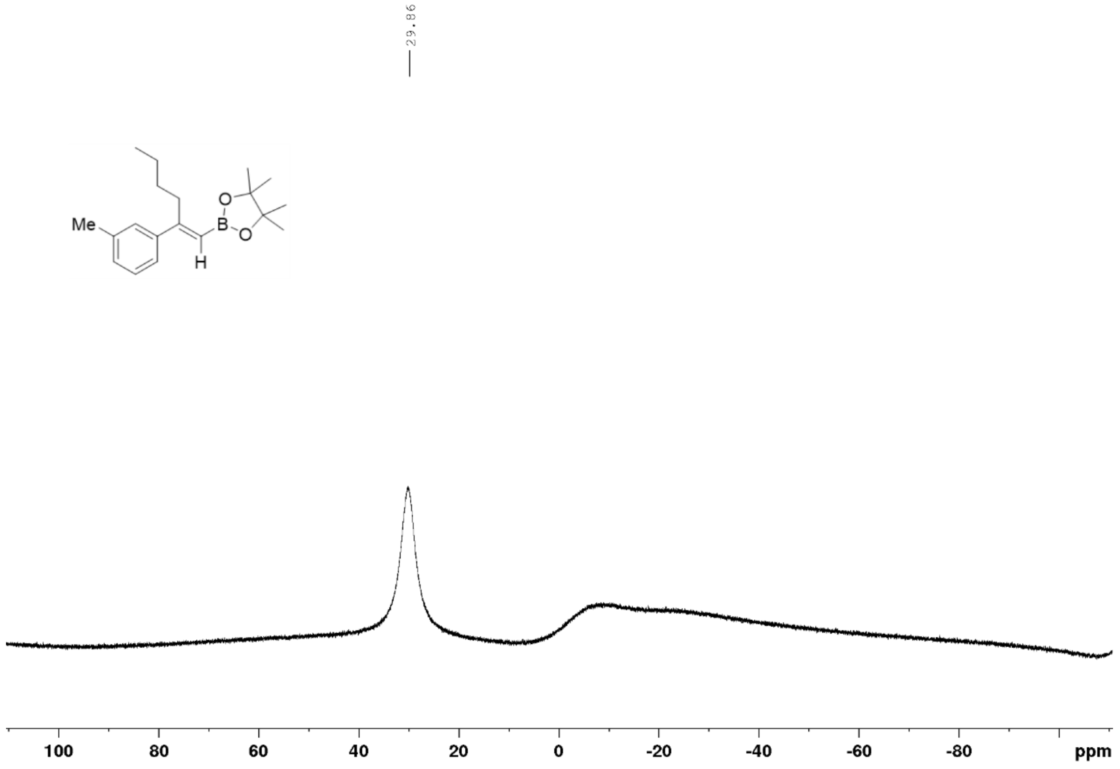
29.86
Me
O
B
O
H
100 80 60 40 20 0 -20 -40 -60 -80 ppm

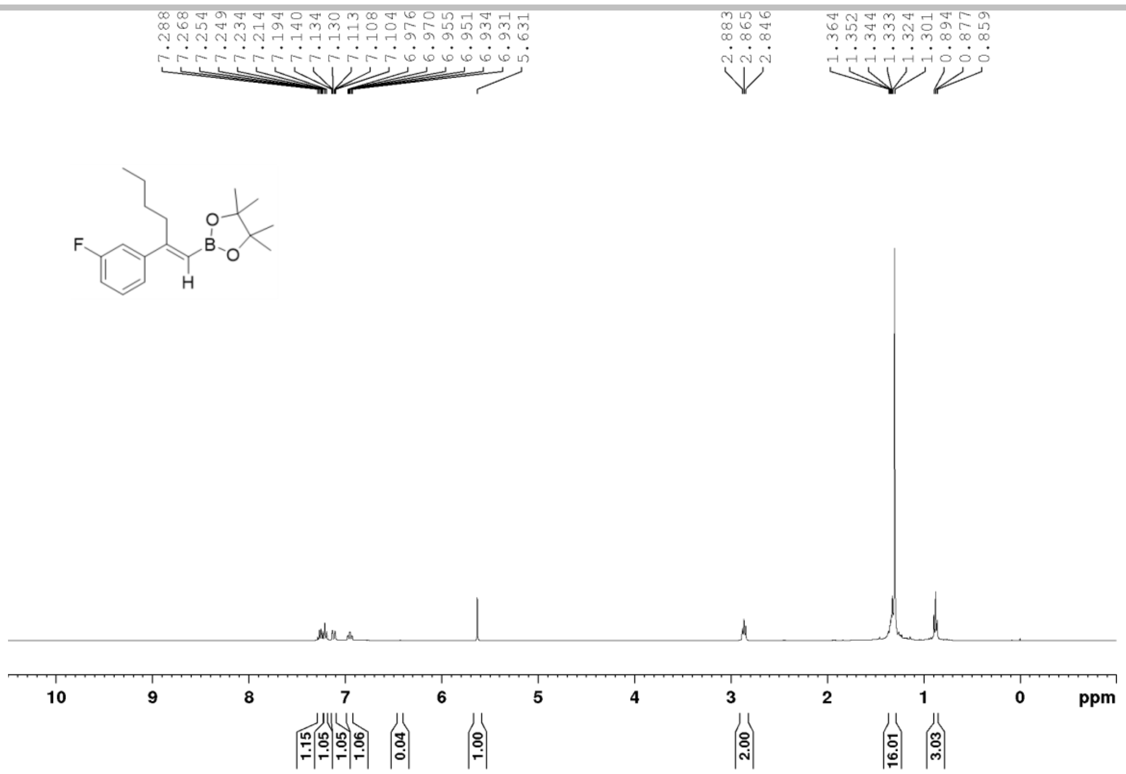
7.288
7.268
7.254
7.249
7.234
7.214
7.194
7.140
7.134
7.130
7.113
7.108
7.104
6.976
6.970
6.955
6.951
6.934
6.931
5.631
2.883
2.865
2.846
1.364
1.352
1.344
1.333
1.324
1.301
0.894
0.877
0.859
F
O
B
O
H
10 9 8 7 6 5 4 3 2 1 0 ppm
1.15
1.05
1.05
1.06
0.04
1.00
2.00
16.01
3.03

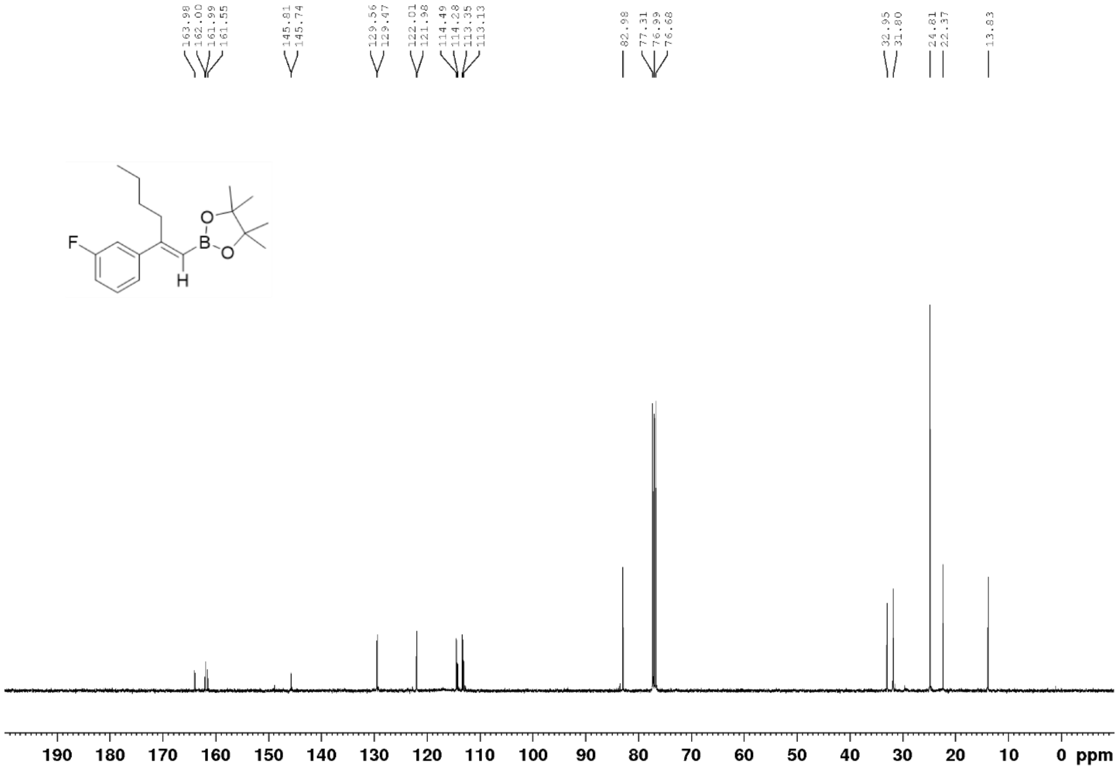
163.98
162.00
161.99
161.55
145.81
145.74
129.56
129.47
122.01
121.98
114.49
114.28
113.35
113.13
82.98
77.31
76.99
76.68
32.95
31.80
24.81
22.37
13.83
F
O
B
O
H
190 180 170 160 150 140 130 120 110 100 90 80 70 60 50 40 30 20 10 0 ppm

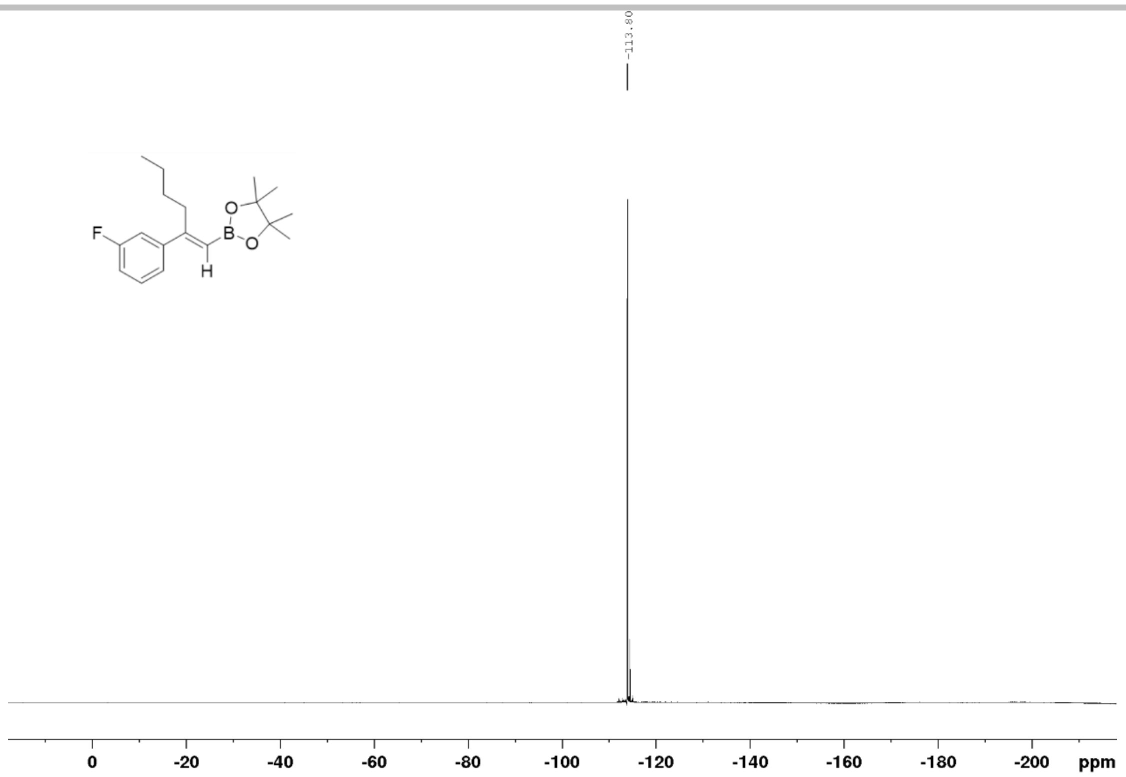
-113.80
F
O
B
O
H
0
-20
-40
-60
-80
-100
-120
-140
-160
-180
-200
ppm

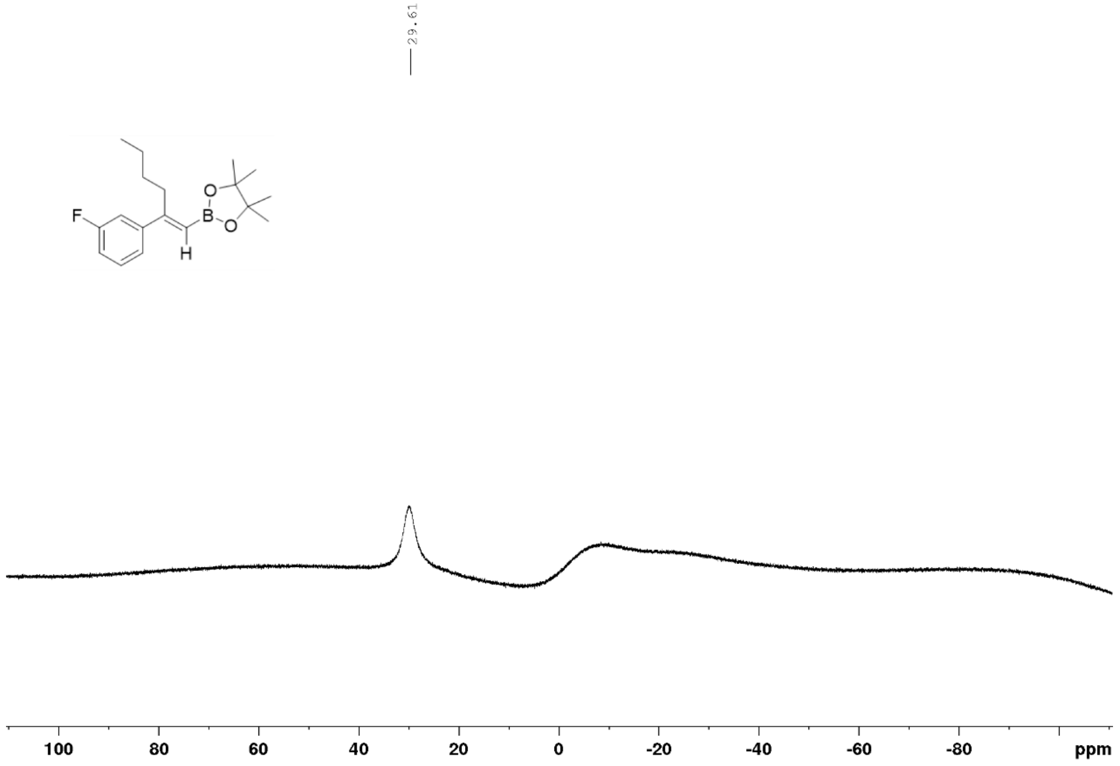
29.61
F
O
B
O
H
100
80
60
40
20
0
-20
-40
-60
-80
ppm

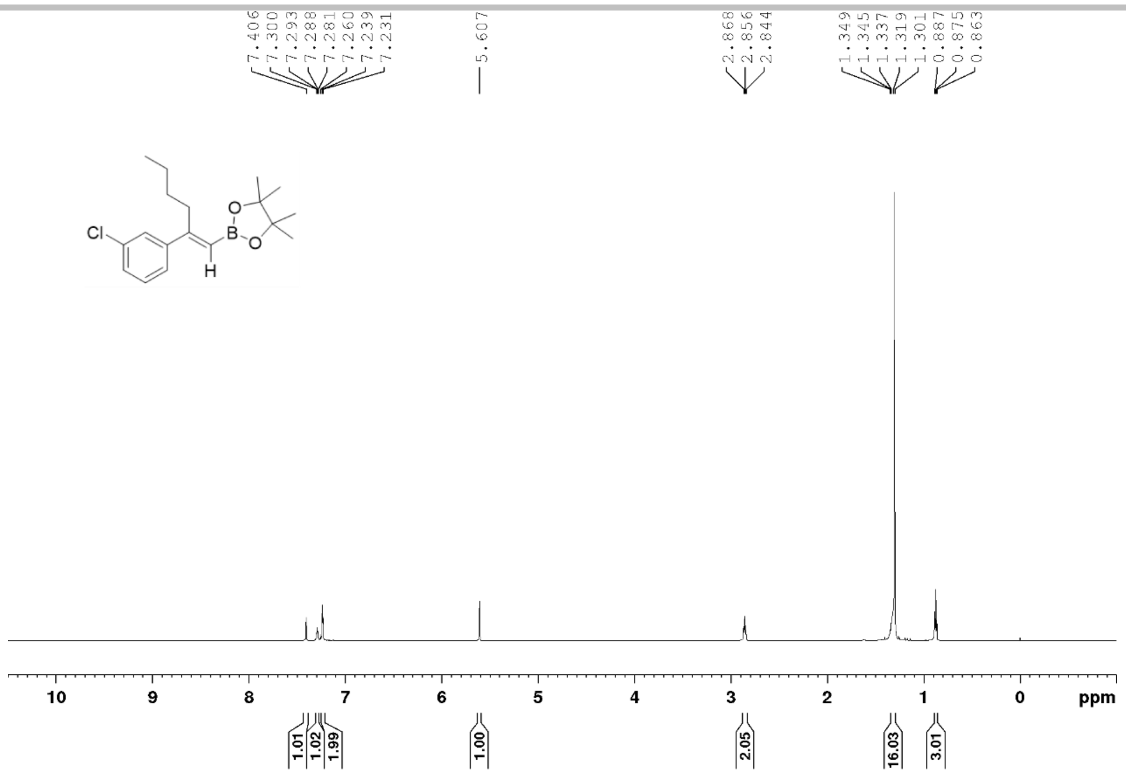
7.406
7.300
7.293
7.288
7.281
7.260
7.239
7.231
5.607
2.868
2.856
2.844
1.349
1.345
1.337
1.319
1.301
0.887
0.875
0.863
Cl
B
O
O
H
10 9 8 7 6 5 4 3 2 1 0 ppm
1.01
1.02
1.99
1.00
2.05
16.03
3.01

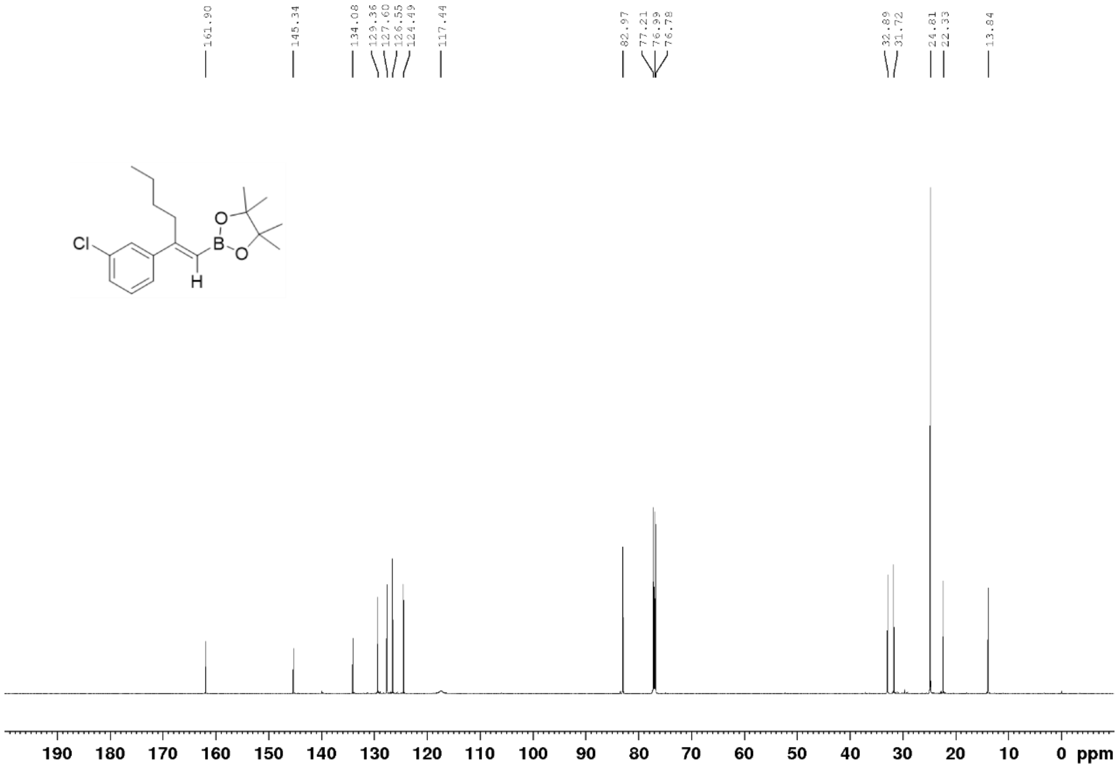
161.90
145.34
134.08
129.36
127.60
126.55
124.49
117.44
82.97
77.21
76.99
76.78
32.89
31.72
24.81
22.33
13.84
Cl
B
O
O
H
190 180 170 160 150 140 130 120 110 100 90 80 70 60 50 40 30 20 10 0 ppm

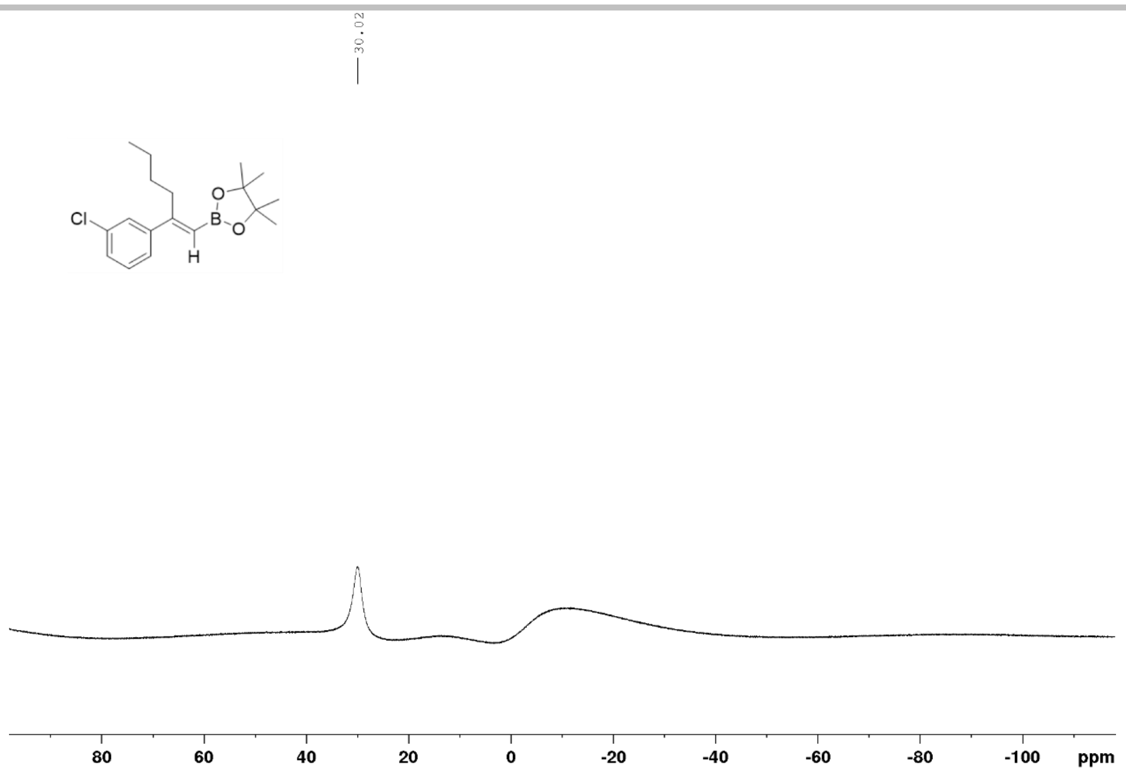

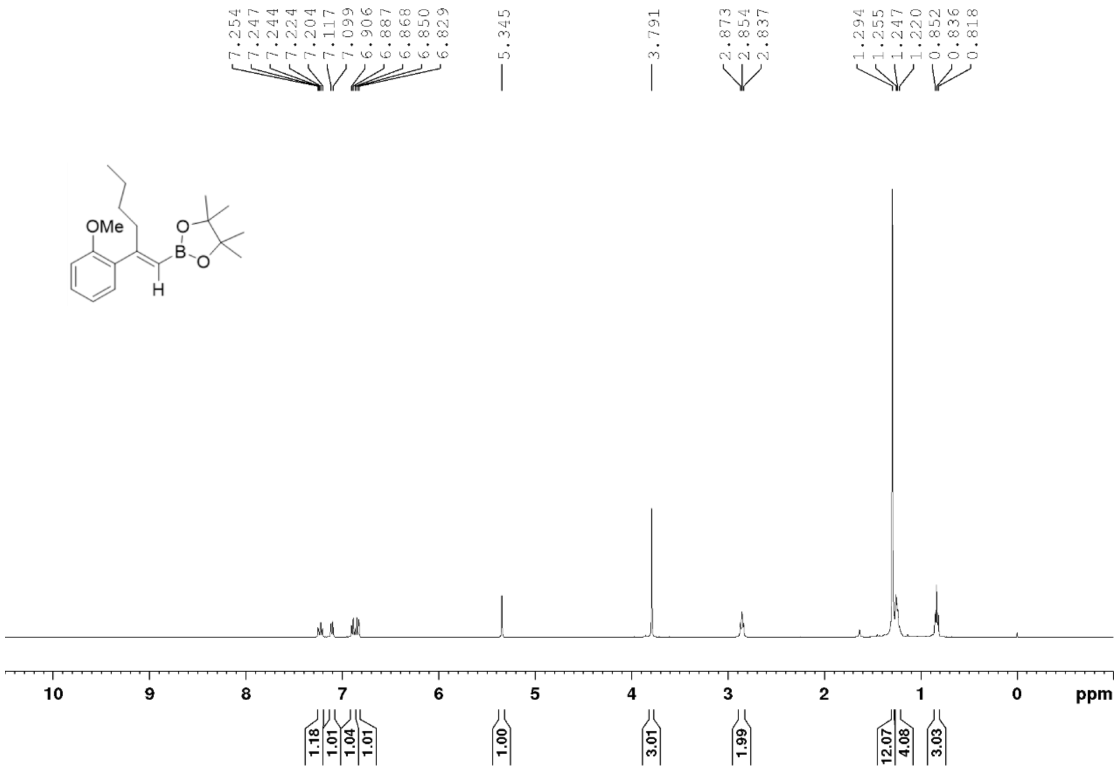

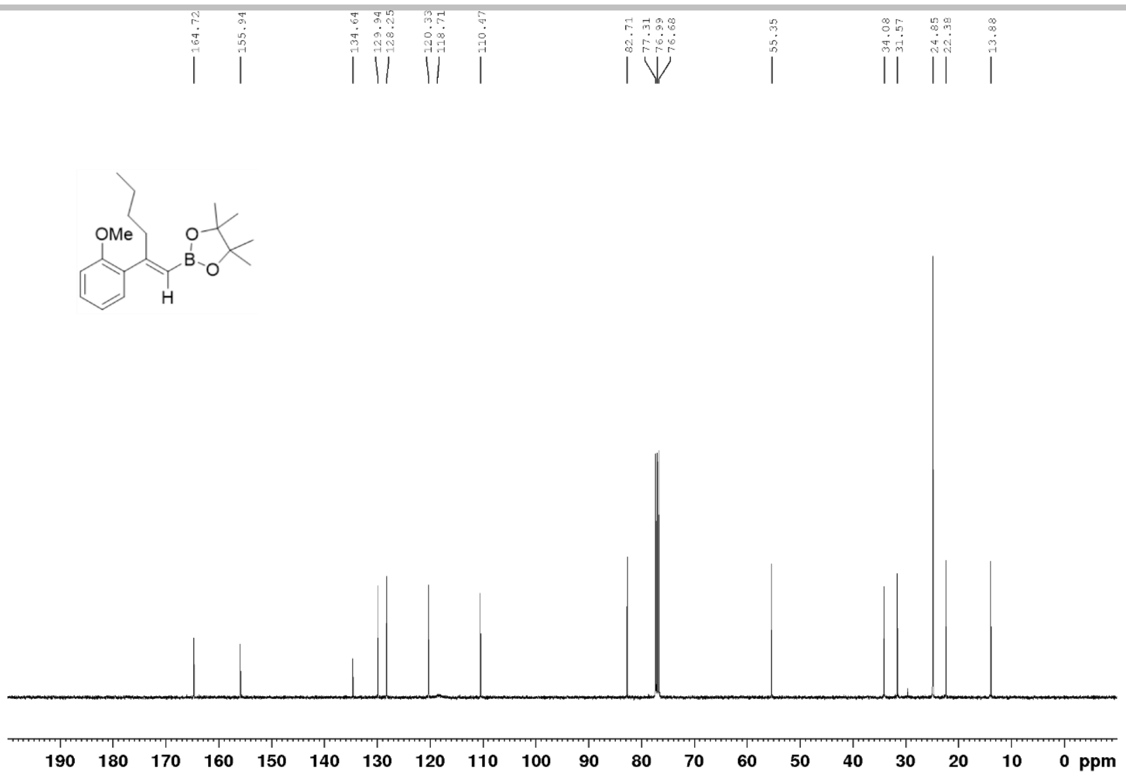
164.72
155.94
134.64
129.94
128.25
120.33
118.71
110.47
82.71
77.31
76.99
76.68
55.35
34.08
31.57
24.85
22.38
13.88
OMe
O
B
O
H
190 180 170 160 150 140 130 120 110 100 90 80 70 60 50 40 30 20 10 0 ppm

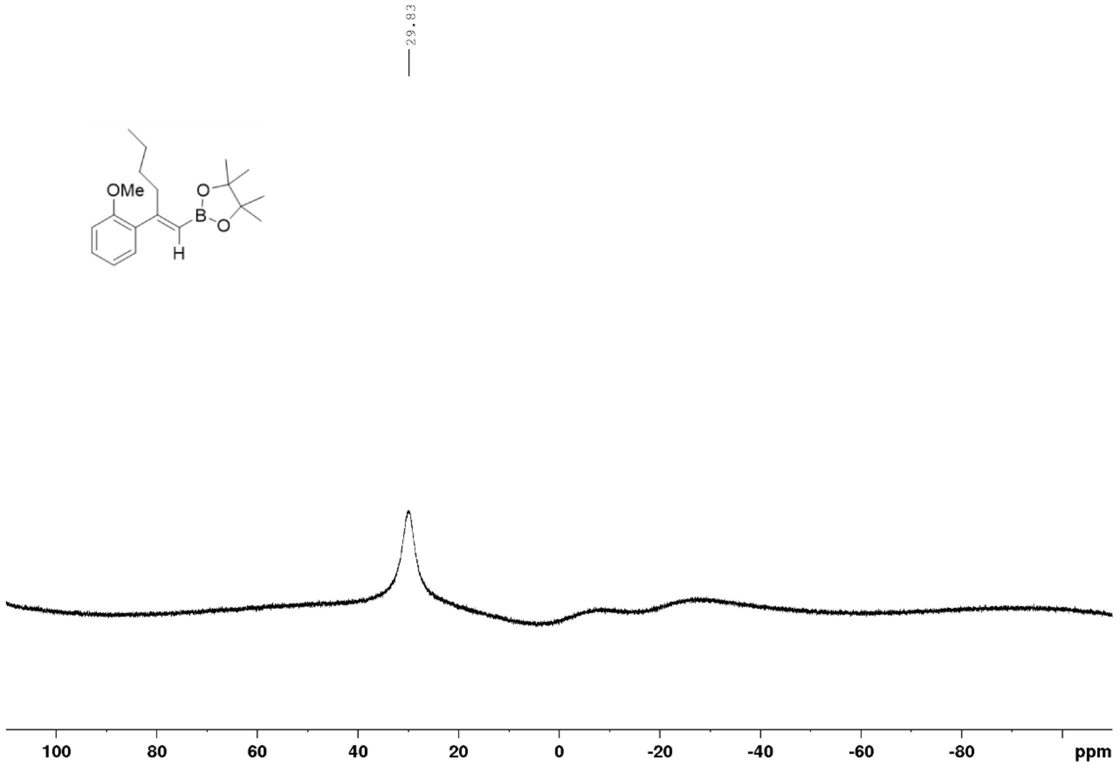
29.83
OMe
O
B
O
H
100 80 60 40 20 0 -20 -40 -60 -80 ppm

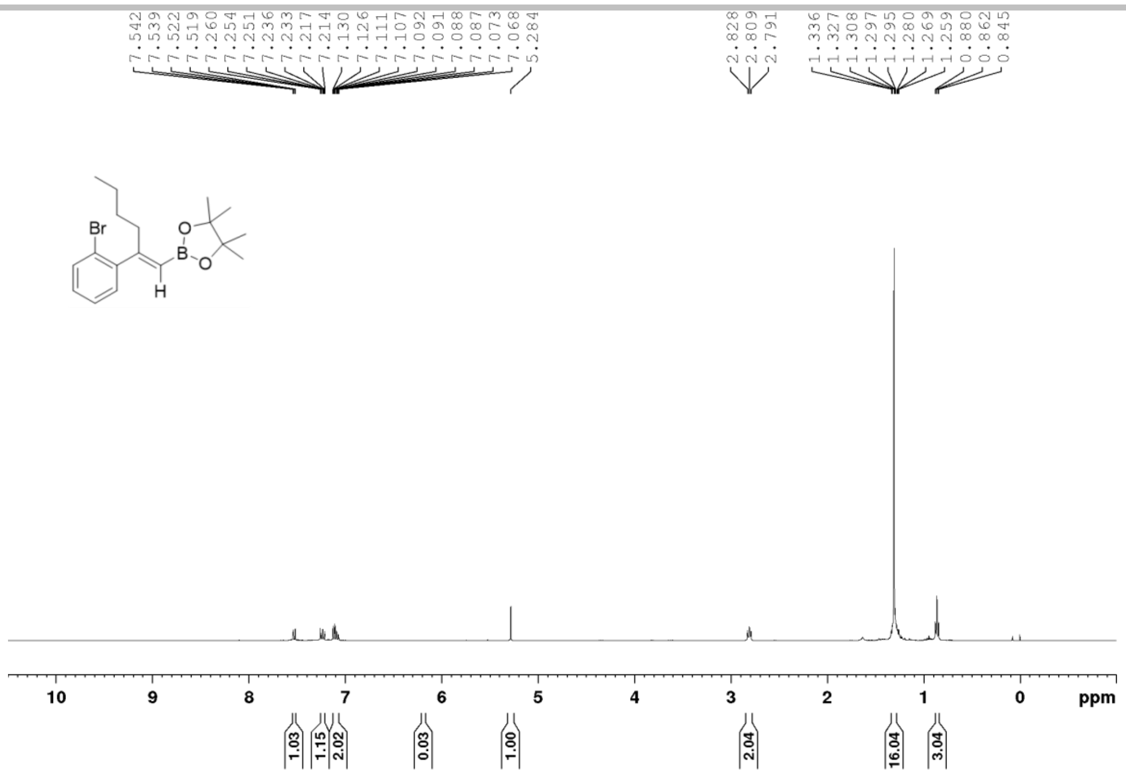

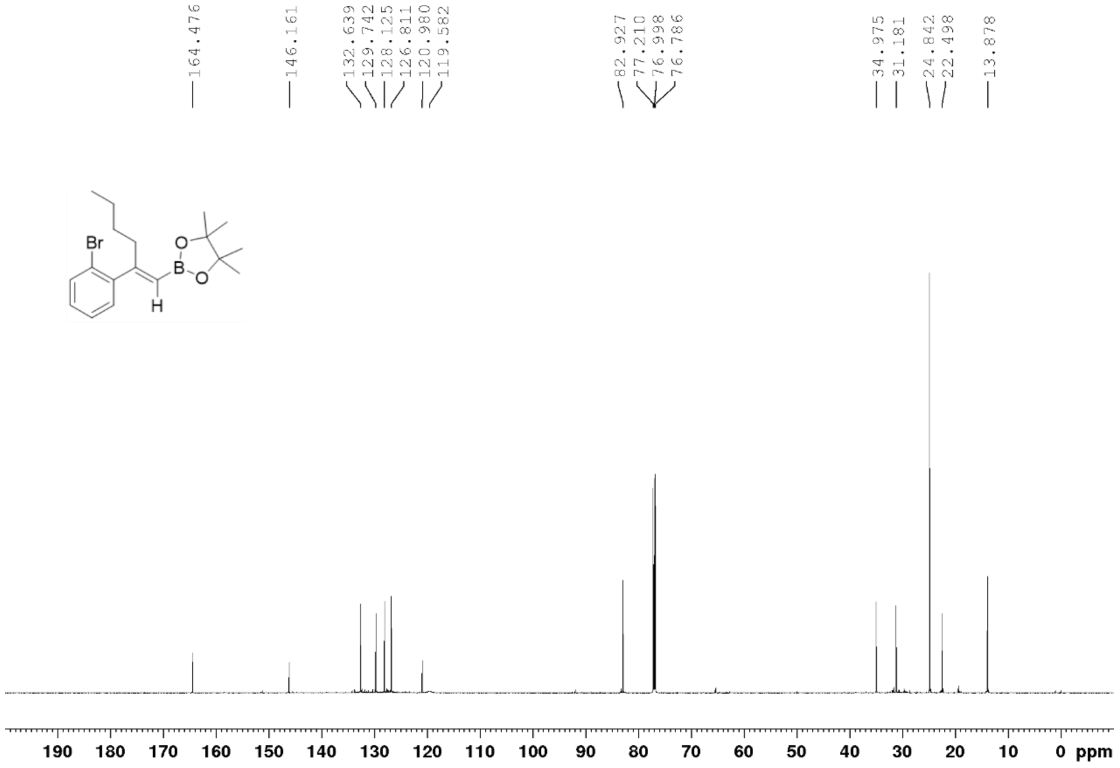

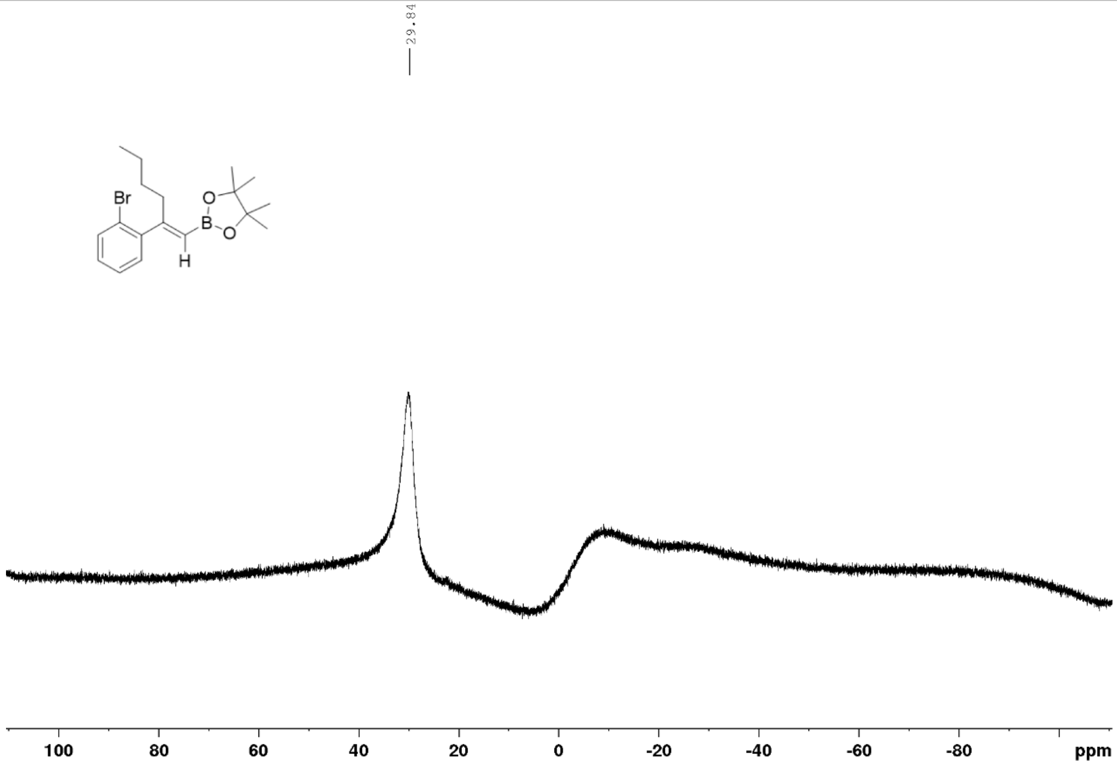
29.84
Br
O
B
O
H
100
80
60
40
20
0
-20
-40
-60
-80
ppm

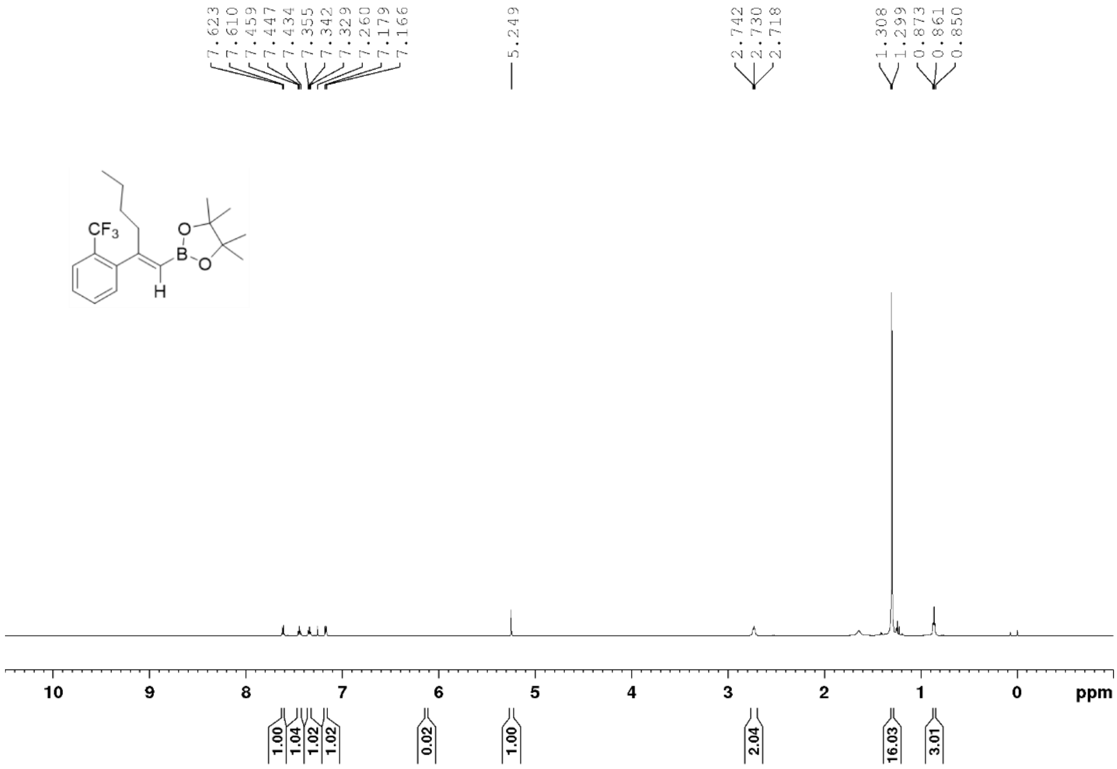
7.623
7.610
7.459
7.447
7.434
7.355
7.342
7.329
7.260
7.179
7.166
5.249
2.742
2.730
2.718
1.308
1.299
0.873
0.861
0.850
CF_3
O
B
O
H
10
9
8
7
6
5
4
3
2
1
0
ppm
1.00
1.04
1.02
1.02
0.02
1.00
2.04
16.03
3.01

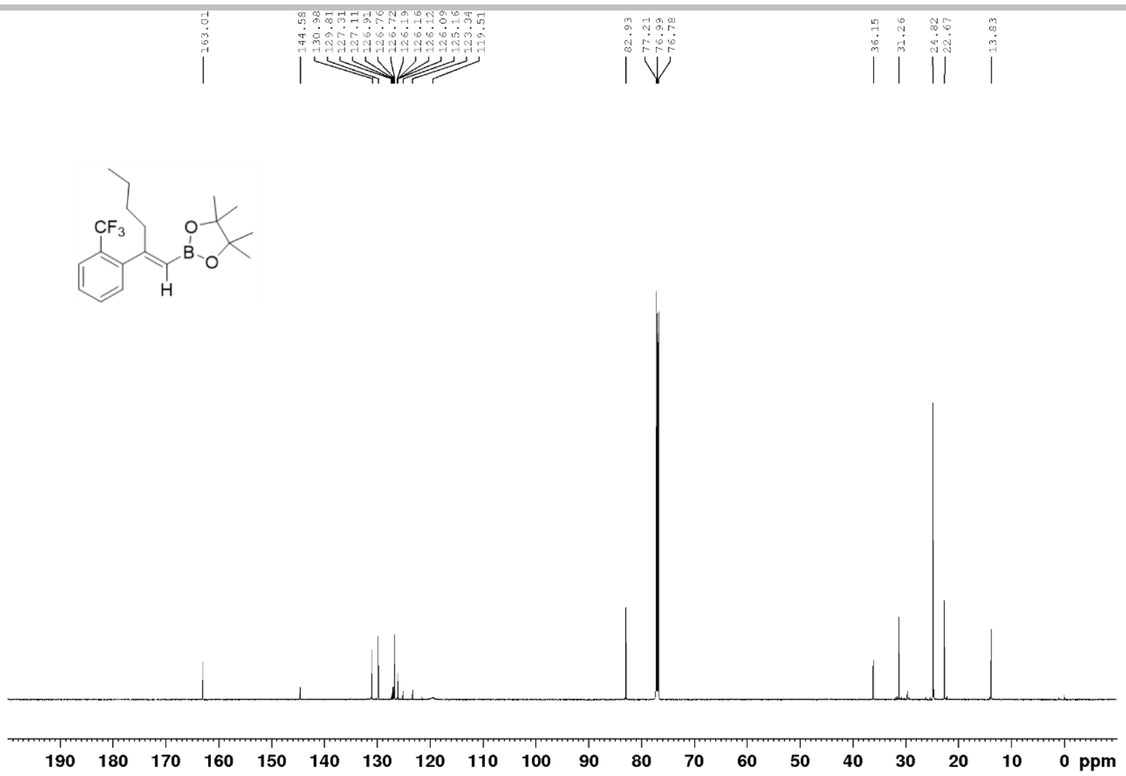
163.01
144.58
130.98
129.81
127.31
127.11
126.91
126.76
126.72
126.19
126.16
126.12
126.09
125.16
123.34
119.51
82.93
77.21
76.99
76.78
36.15
31.26
24.82
22.67
13.83
CF_3
O
B
O
H
190 180 170 160 150 140 130 120 110 100 90 80 70 60 50 40 30 20 10 0 ppm

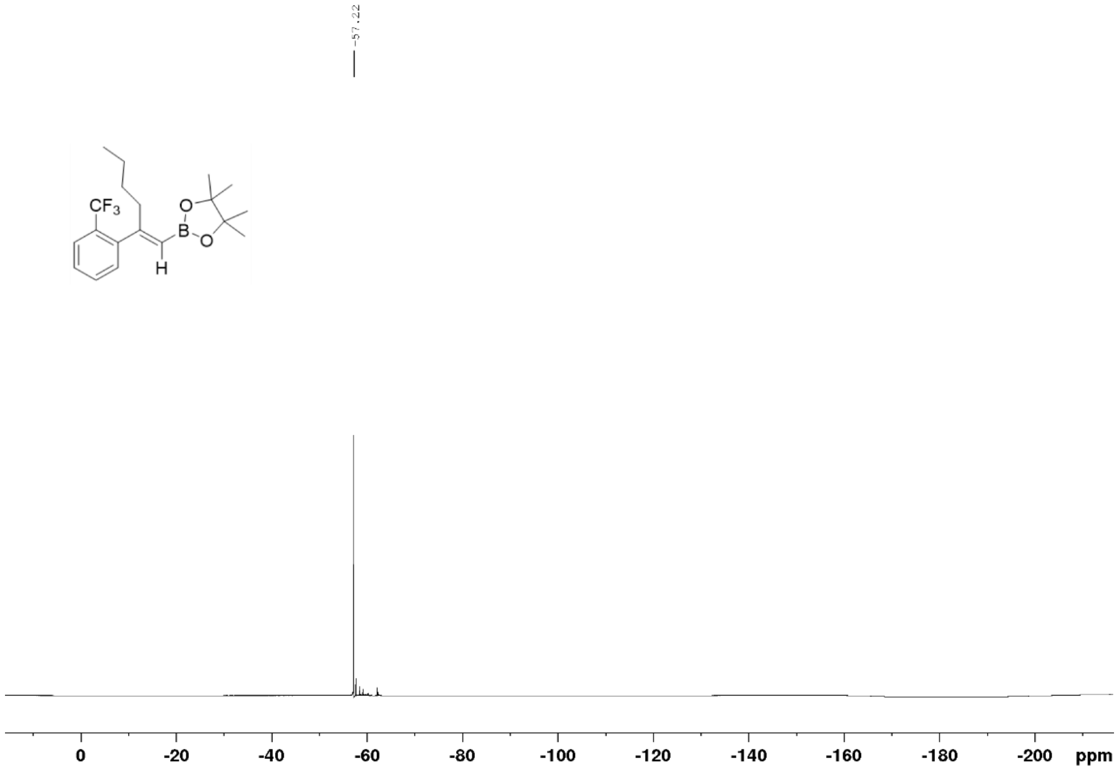
-57.22
CF_3
O
B
O
H
0 -20 -40 -60 -80 -100 -120 -140 -160 -180 -200 ppm

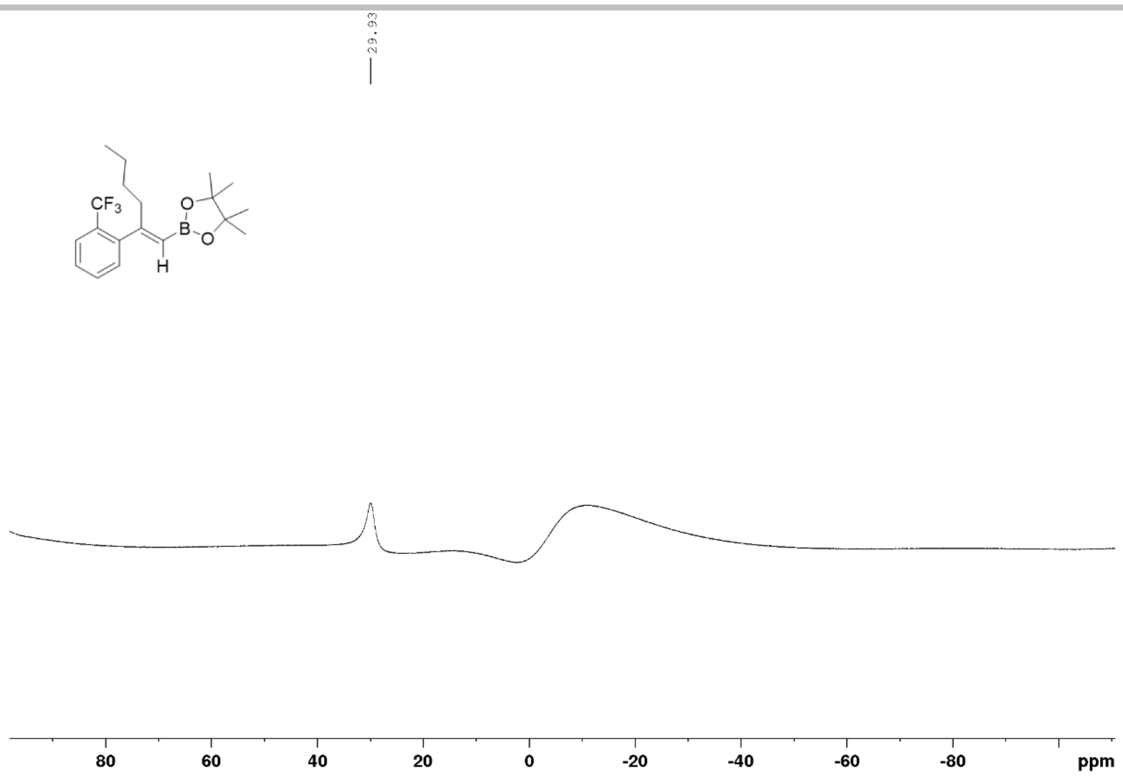
29.93
CF3
O
B
O
H
80
60
40
20
0
-20
-40
-60
-80
ppm

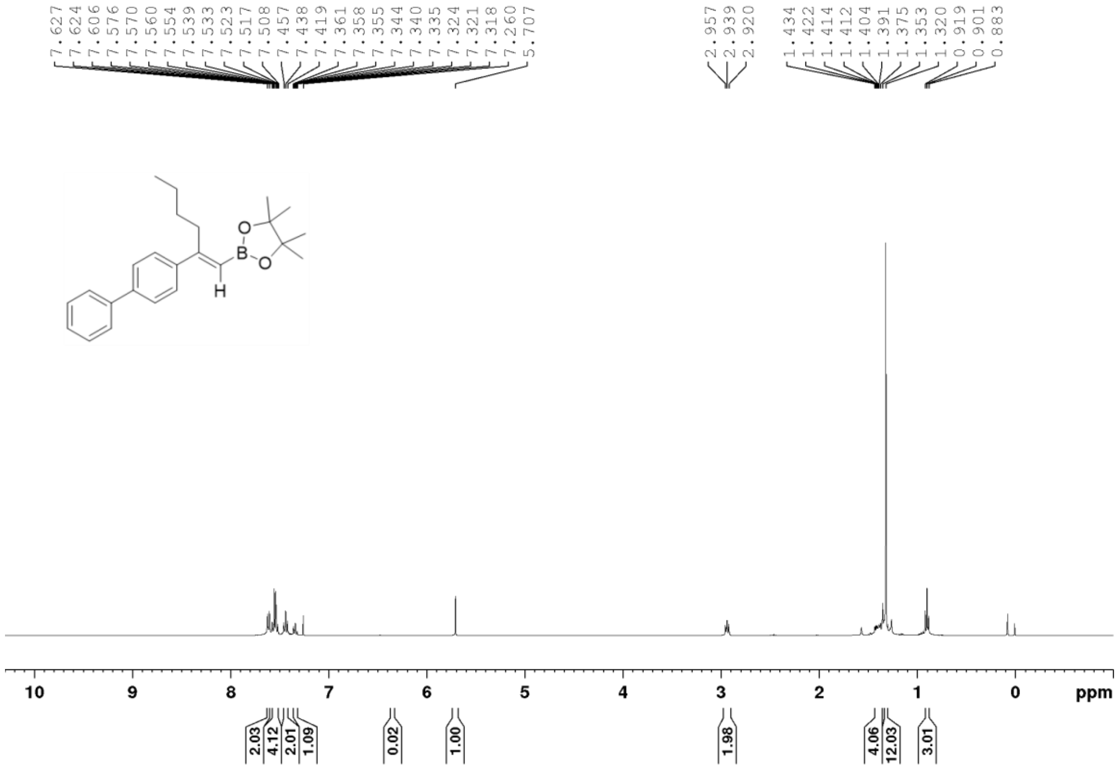
7.627
7.624
7.606
7.576
7.570
7.560
7.554
7.539
7.533
7.523
7.517
7.508
7.457
7.438
7.419
7.361
7.358
7.355
7.344
7.340
7.335
7.324
7.321
7.318
7.260
5.707
2.957
2.939
2.920
1.434
1.422
1.414
1.412
1.404
1.391
1.375
1.353
1.320
0.919
0.901
0.883
O
B
O
H
10
9
8
7
6
5
4
3
2
1
0
ppm
2.03
4.12
2.01
1.09
0.02
1.00
1.98
4.06
12.03
3.01

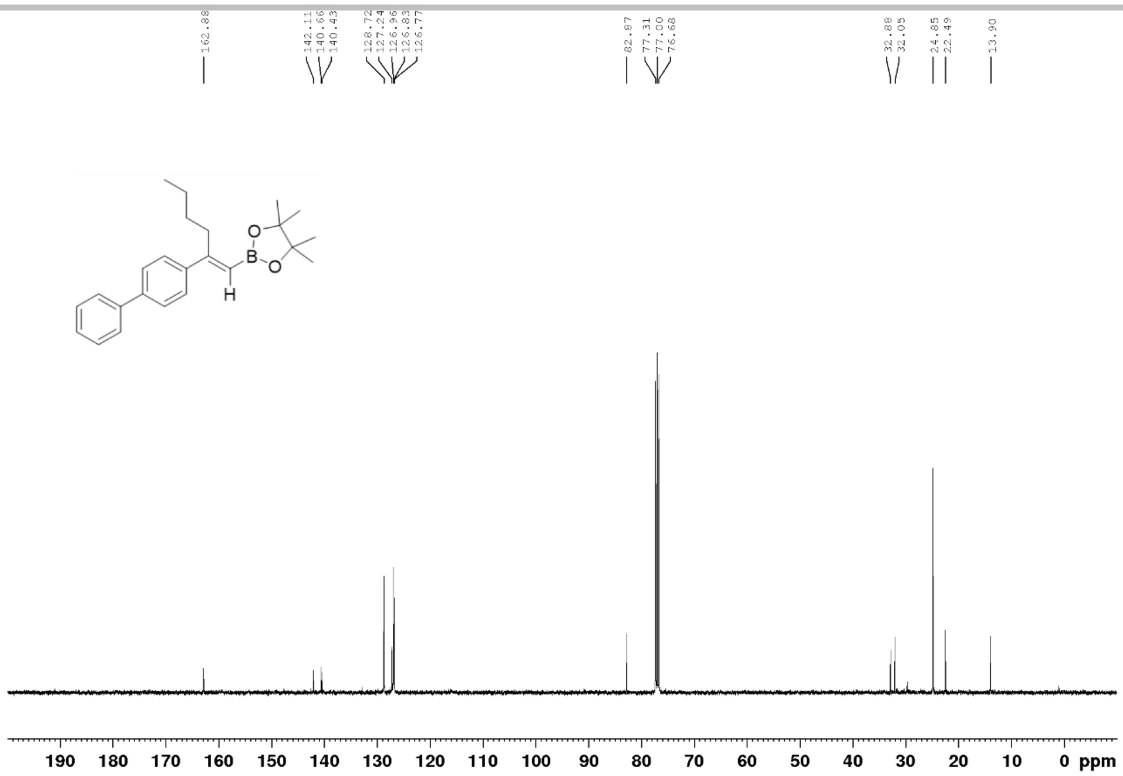
162.88
142.11
140.66
140.43
128.72
127.24
126.96
126.83
126.77
82.87
77.31
77.00
76.68
32.88
32.05
24.85
22.49
13.90
190 180 170 160 150 140 130 120 110 100 90 80 70 60 50 40 30 20 10 0 ppm

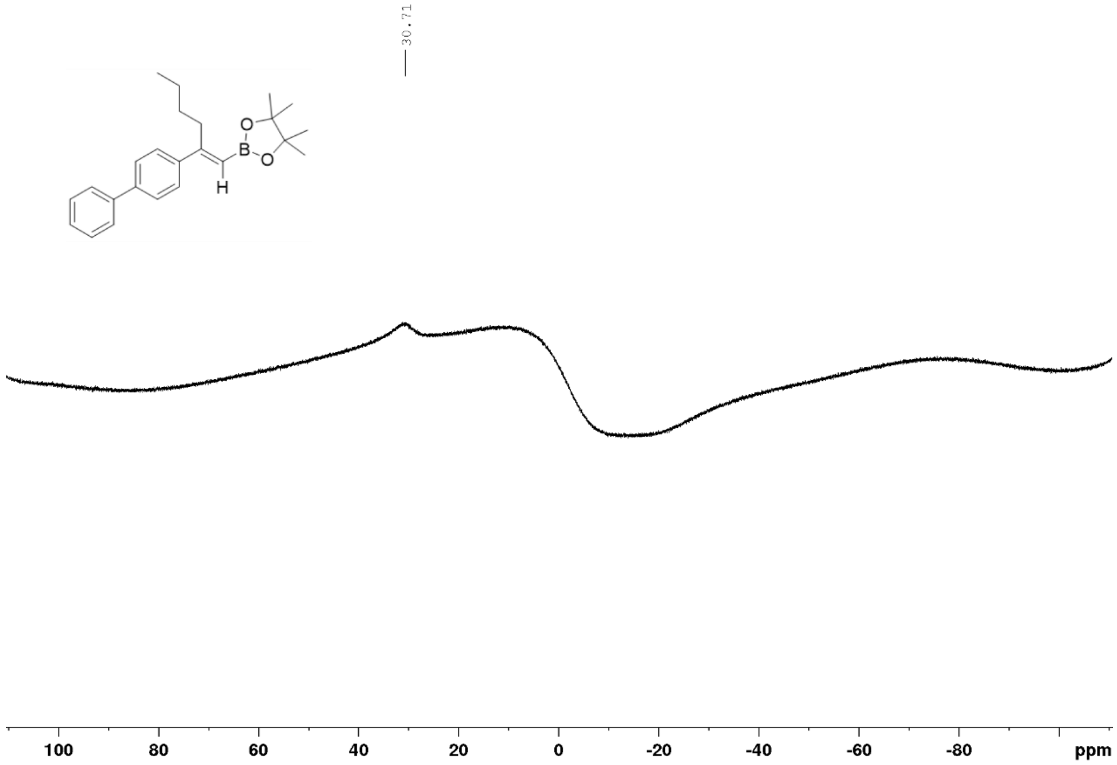
30.71
100 80 60 40 20 0 -20 -40 -60 -80 ppm

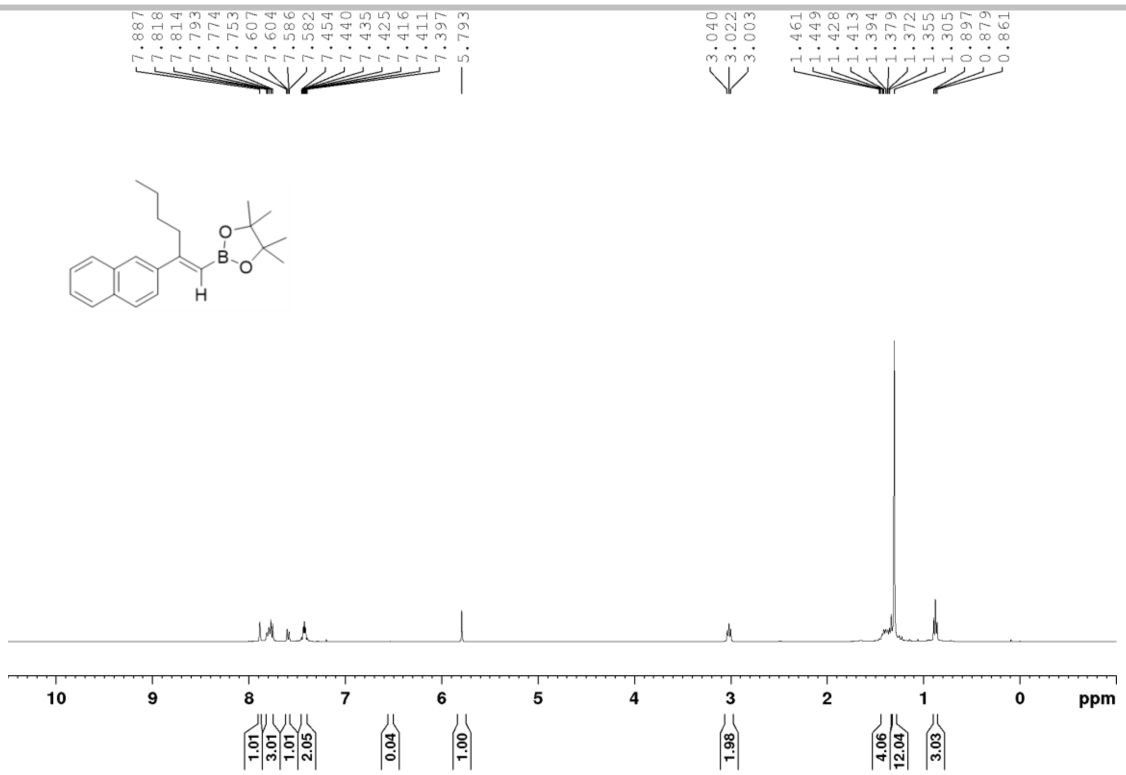
7.887
7.818
7.814
7.793
7.774
7.753
7.607
7.604
7.586
7.582
7.454
7.440
7.435
7.425
7.416
7.411
7.397
5.793
3.040
3.022
3.003
1.461
1.449
1.428
1.413
1.394
1.379
1.372
1.355
1.305
0.897
0.879
0.861
B
O
O
H
10
9
8
7
6
5
4
3
2
1
0
ppm
1.01
3.01
1.01
2.05
0.04
1.00
1.98
4.06
12.04
3.03

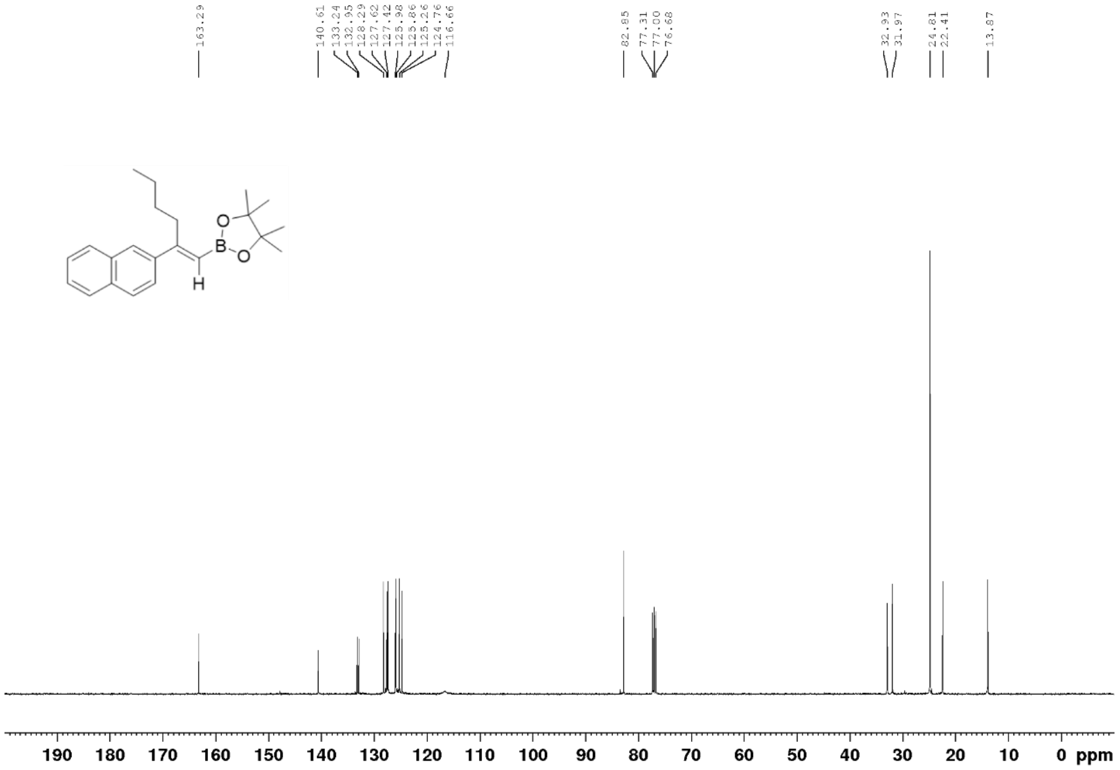
163.29
140.61
133.24
132.95
128.29
127.62
127.42
125.98
125.86
125.26
124.76
116.66
82.85
77.31
77.00
76.68
32.93
31.97
24.81
22.41
13.87
B
O
O
H
190
180
170
160
150
140
130
120
110
100
90
80
70
60
50
40
30
20
10
0 ppm

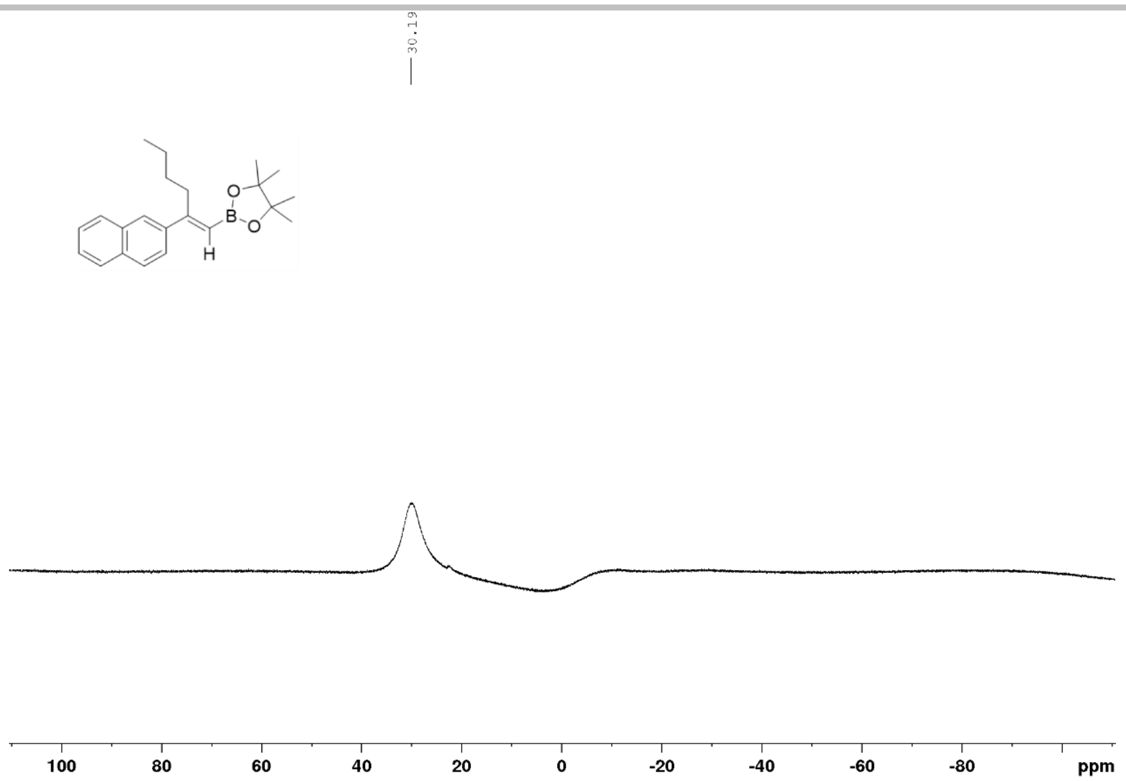
30.19
O
B
O
H
100
80
60
40
20
0
-20
-40
-60
-80
ppm

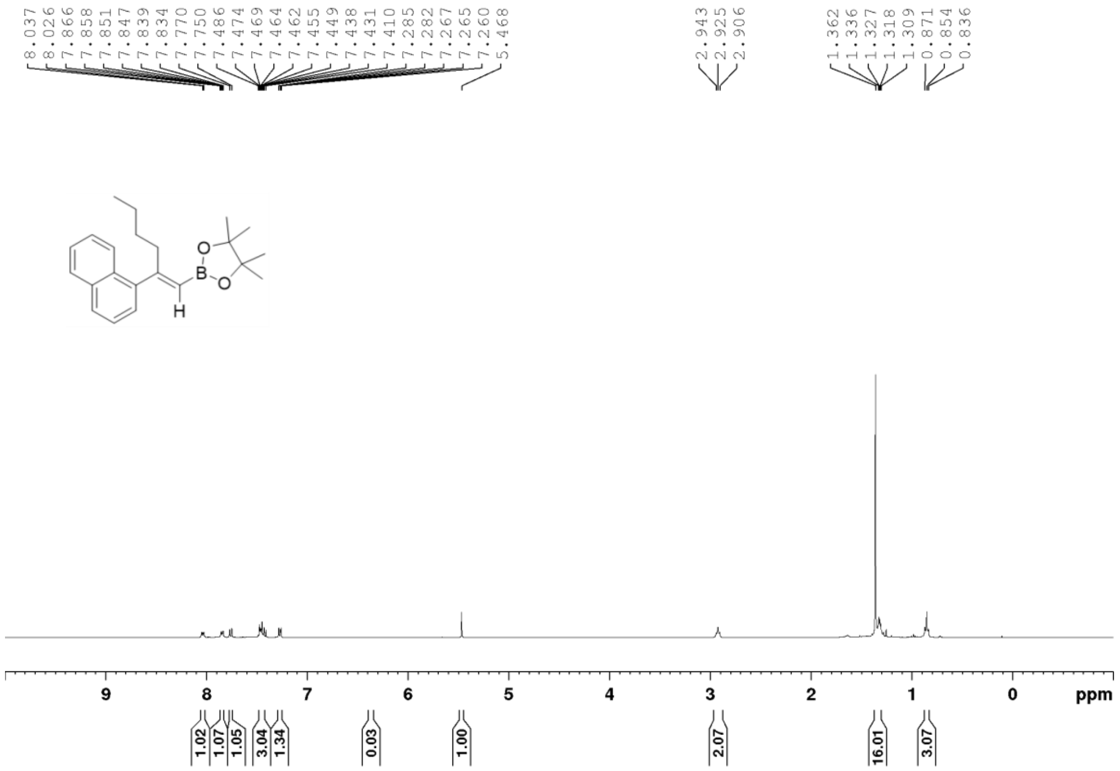
8.037
8.026
7.866
7.858
7.851
7.847
7.839
7.834
7.770
7.750
7.486
7.474
7.469
7.464
7.462
7.455
7.449
7.438
7.431
7.410
7.285
7.282
7.267
7.265
7.260
5.468
2.943
2.925
2.906
1.362
1.336
1.327
1.318
1.309
0.871
0.854
0.836
O
B
O
H
9
8
7
6
5
4
3
2
1
0
ppm
1.02
1.07
1.05
3.04
1.34
0.03
1.00
2.07
16.01
3.07

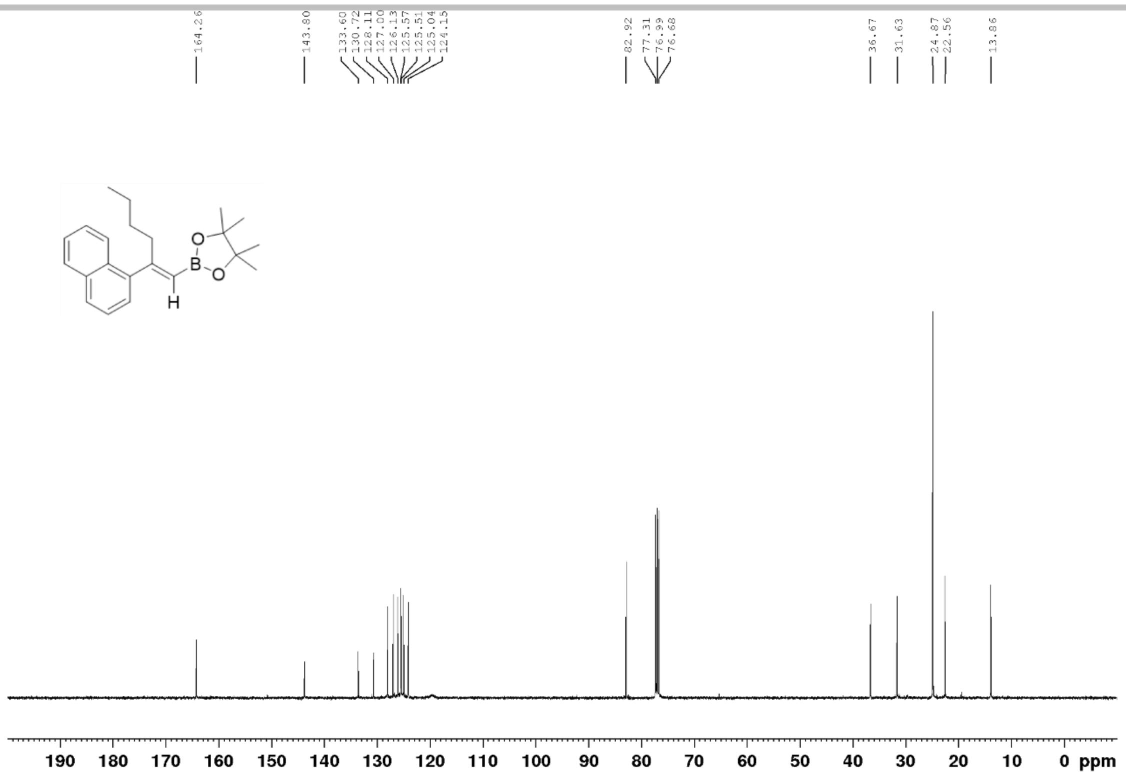
164.26
143.80
133.60
130.72
128.11
127.00
126.13
125.57
125.51
125.04
124.15
82.92
77.31
76.99
76.68
36.67
31.63
24.87
22.56
13.86
H
B
O
O
190 180 170 160 150 140 130 120 110 100 90 80 70 60 50 40 30 20 10 0 ppm

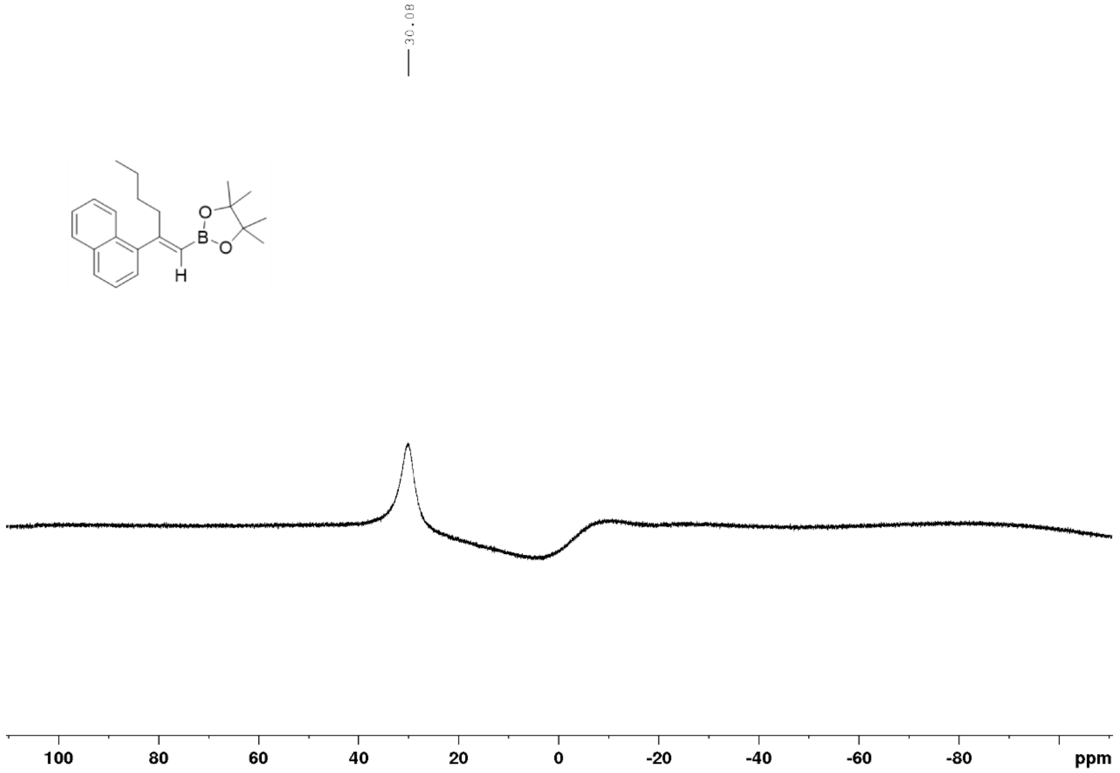
30.08
H
B
O
O
100 80 60 40 20 0 -20 -40 -60 -80 ppm

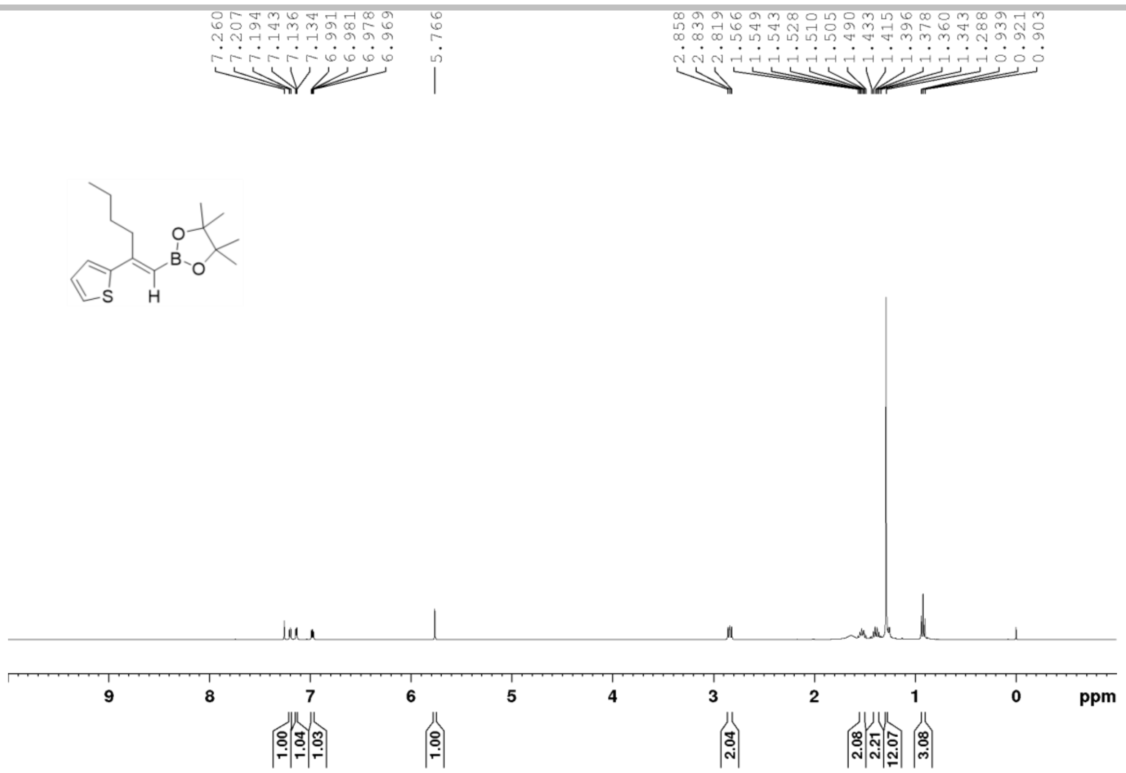
7.260
7.207
7.194
7.143
7.136
7.134
6.991
6.981
6.978
6.969
5.766
2.858
2.839
2.819
1.566
1.549
1.543
1.528
1.510
1.505
1.490
1.433
1.415
1.396
1.378
1.360
1.343
1.288
0.939
0.921
0.903
9
8
7
6
5
4
3
2
1
0
ppm
1.00
1.04
1.03
1.00
2.04
2.08
2.21
12.07
3.08

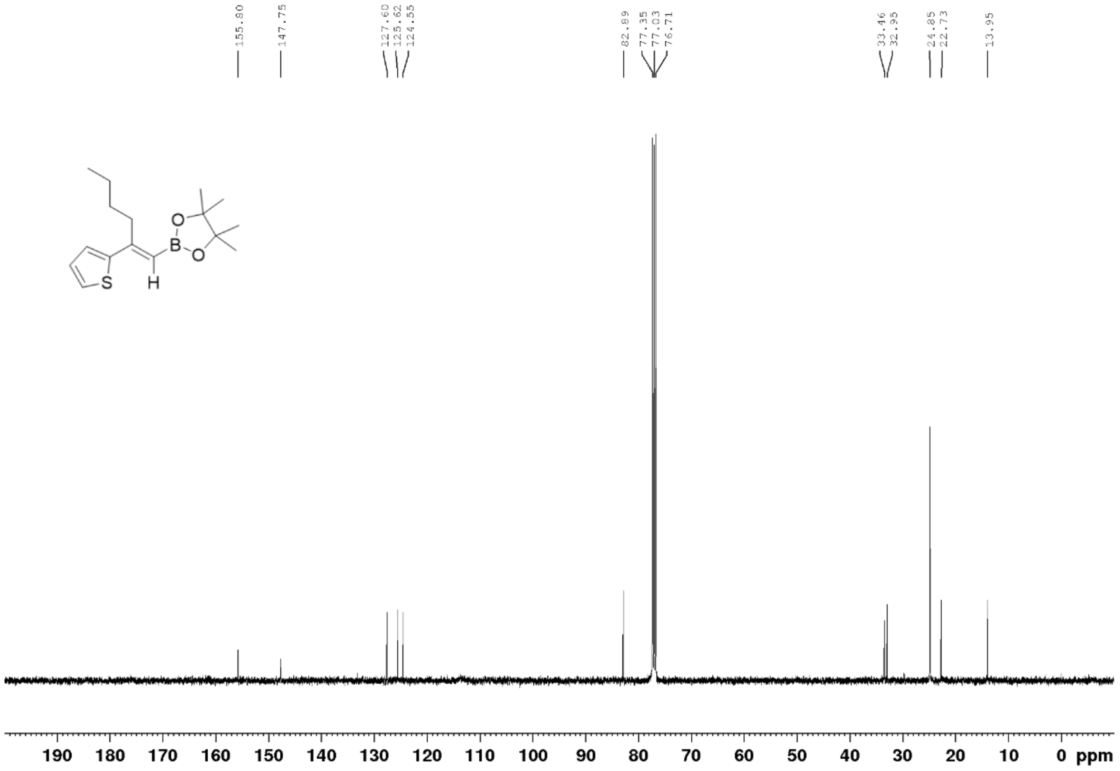
155.80
147.75
127.60
125.62
124.55
82.89
77.35
77.03
76.71
33.46
32.95
24.85
22.73
13.95
190
180
170
160
150
140
130
120
110
100
90
80
70
60
50
40
30
20
10
0 ppm

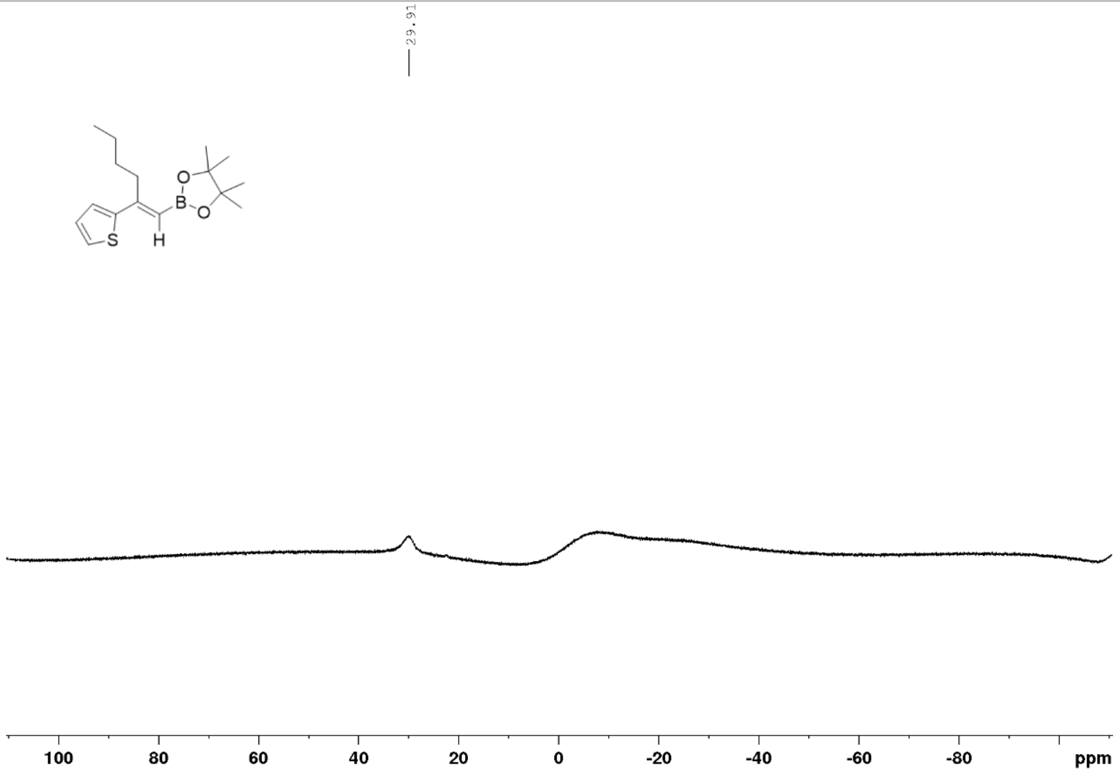
29.91
S
H
B
O
O
100
80
60
40
20
0
-20
-40
-60
-80
ppm

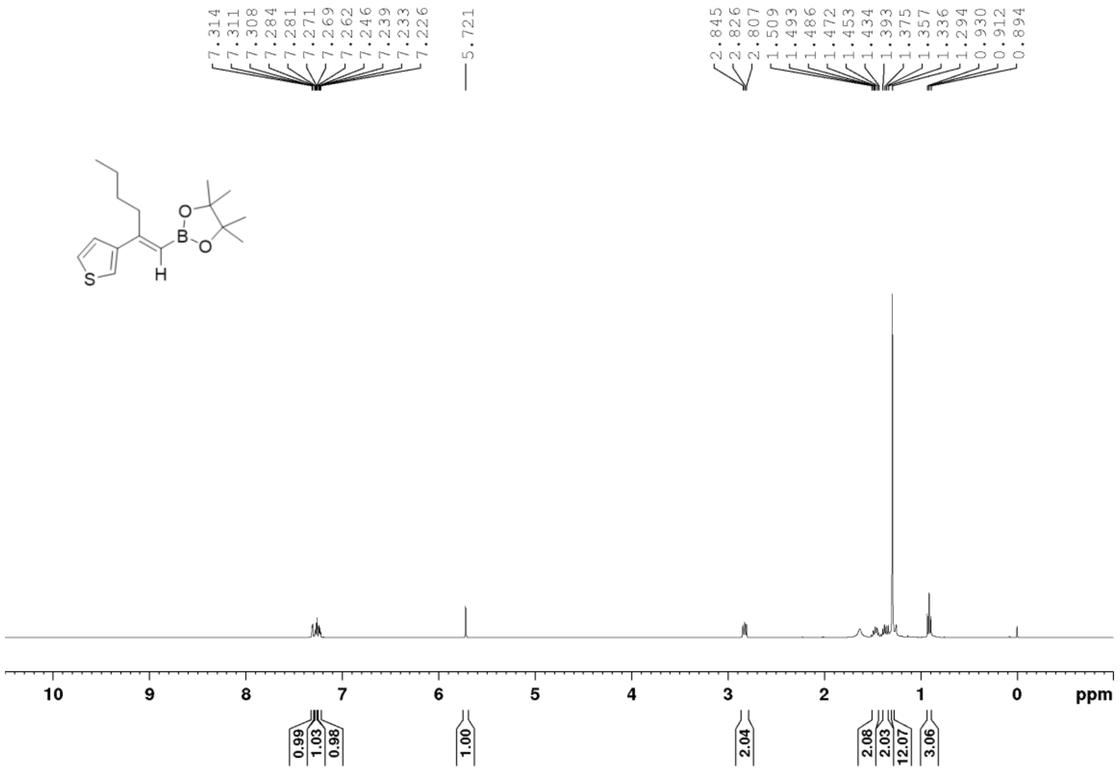
7.314
7.311
7.308
7.284
7.281
7.271
7.269
7.262
7.246
7.239
7.233
7.226
5.721
2.845
2.826
2.807
1.509
1.493
1.486
1.472
1.453
1.434
1.393
1.375
1.357
1.336
1.294
0.930
0.912
0.894
S
H
B
O
O
10
9
8
7
6
5
4
3
2
1
0
ppm
0.99
1.03
0.98
1.00
2.04
2.08
2.03
12.07
3.06

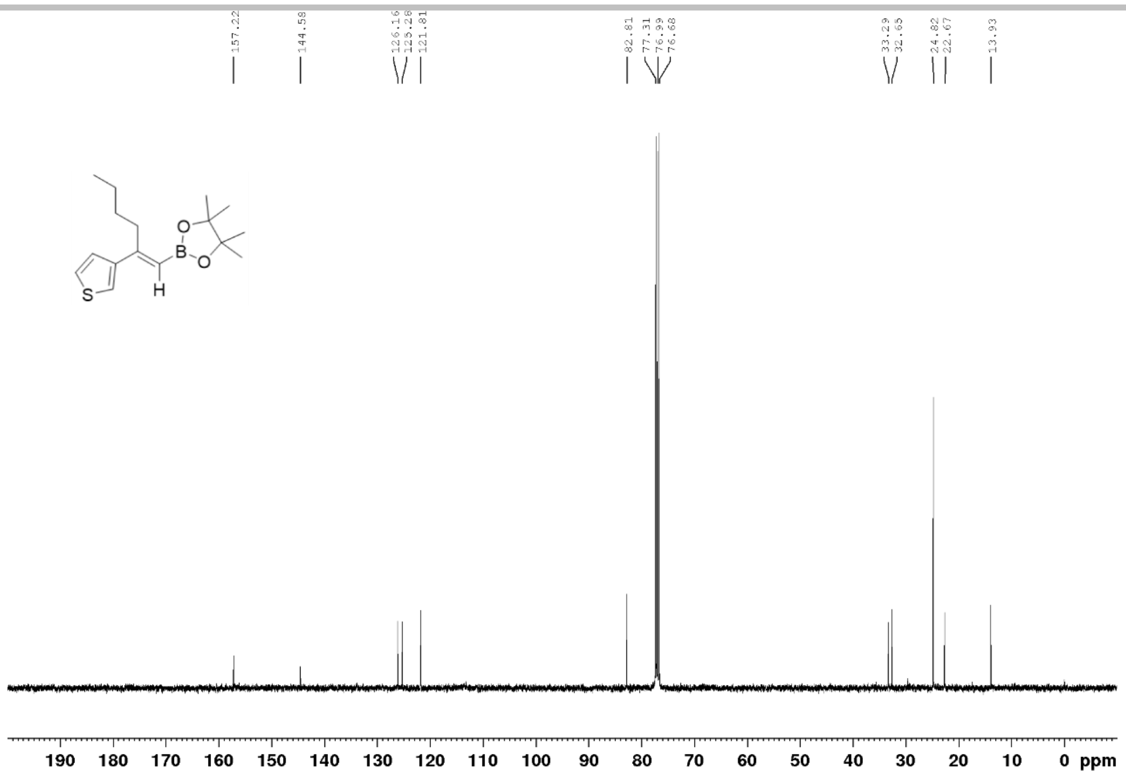
157.22
144.58
126.16
125.26
121.81
82.81
77.31
76.99
76.68
33.29
32.65
24.82
22.67
13.93
190 180 170 160 150 140 130 120 110 100 90 80 70 60 50 40 30 20 10 0 ppm

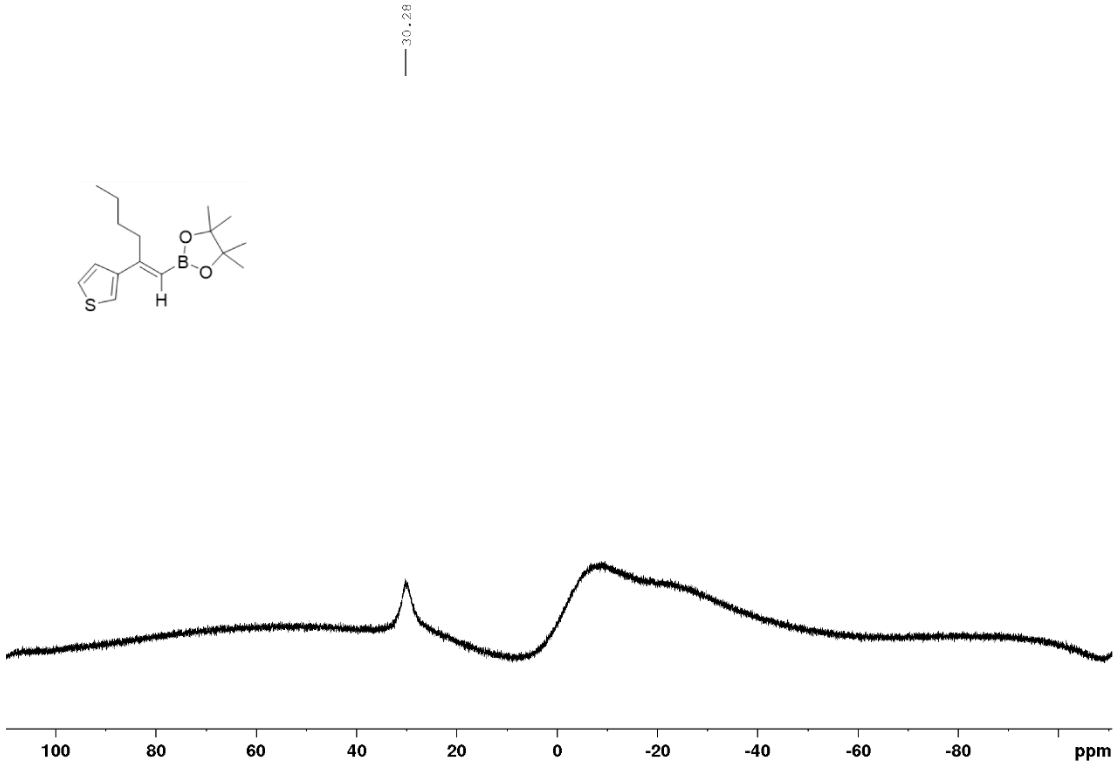
30.28
100 80 60 40 20 0 -20 -40 -60 -80 ppm

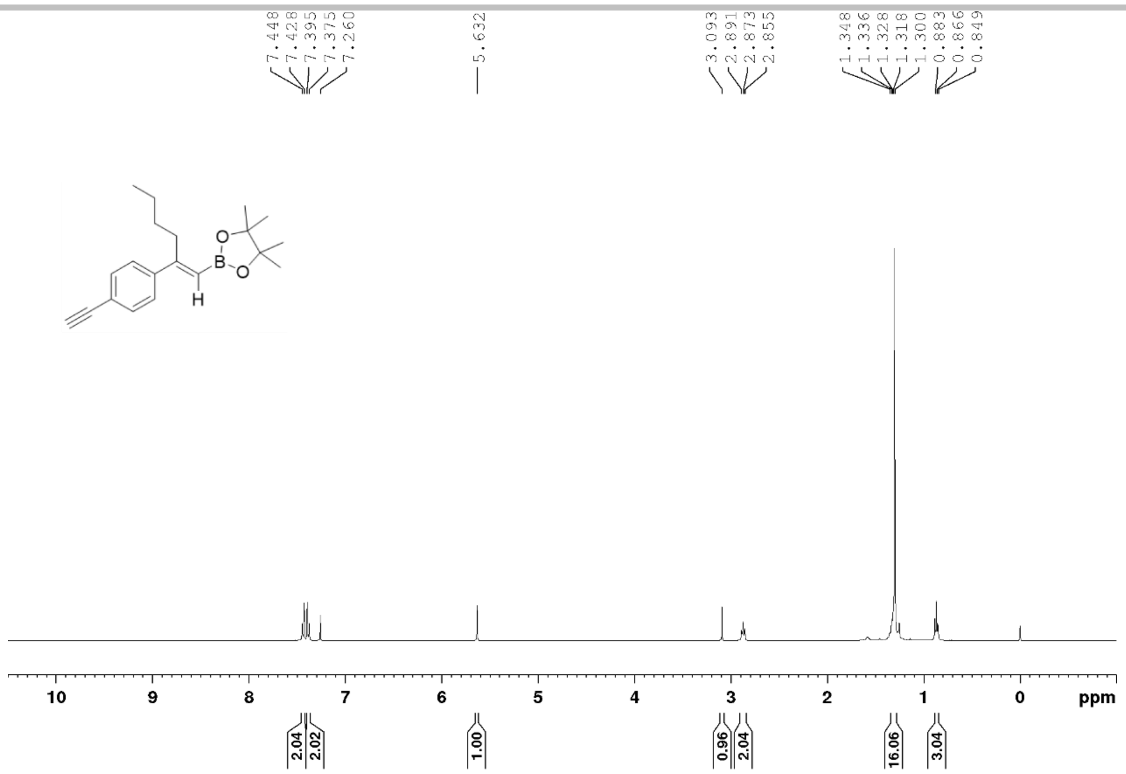
7.448
7.428
7.395
7.375
7.260
5.632
3.093
2.891
2.873
2.855
1.348
1.336
1.328
1.318
1.300
0.883
0.866
0.849
H
B
O
O
10
9
8
7
6
5
4
3
2
1
0
ppm
2.04
2.02
1.00
0.96
2.04
16.06
3.04

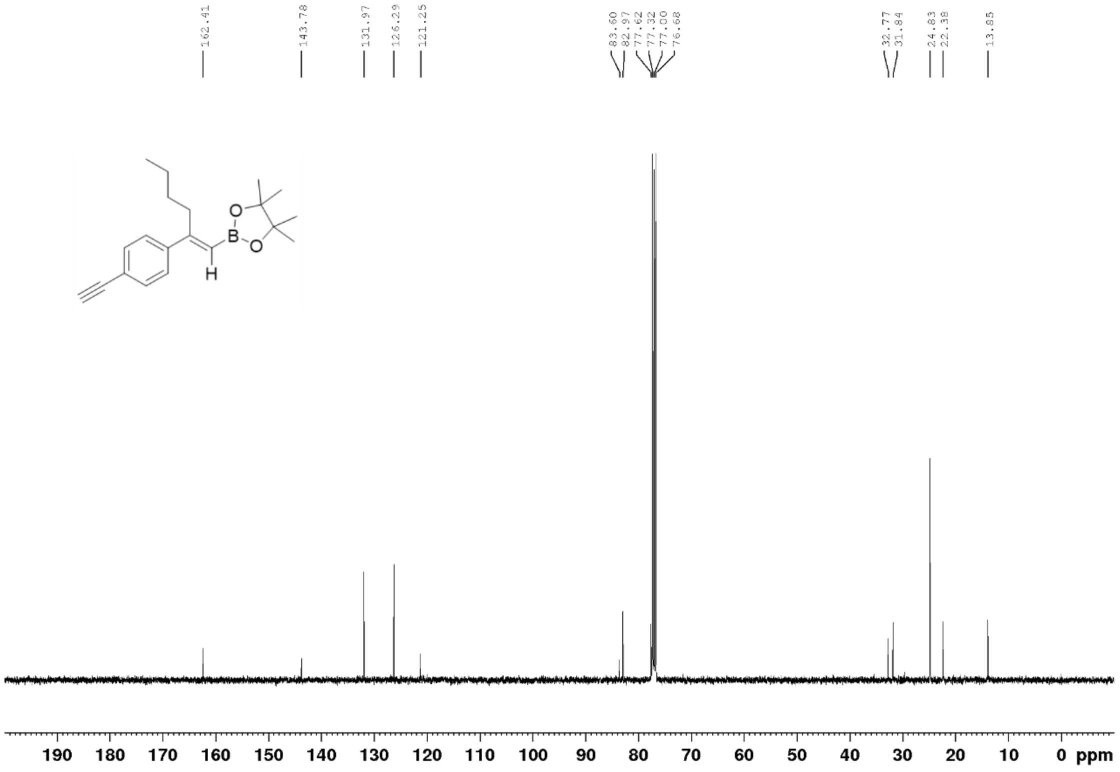
162.41
143.78
131.97
126.29
121.25
83.60
82.97
77.62
77.32
77.00
76.68
32.77
31.84
24.83
22.38
13.85
H
B
O
O
190
180
170
160
150
140
130
120
110
100
90
80
70
60
50
40
30
20
10
0 ppm

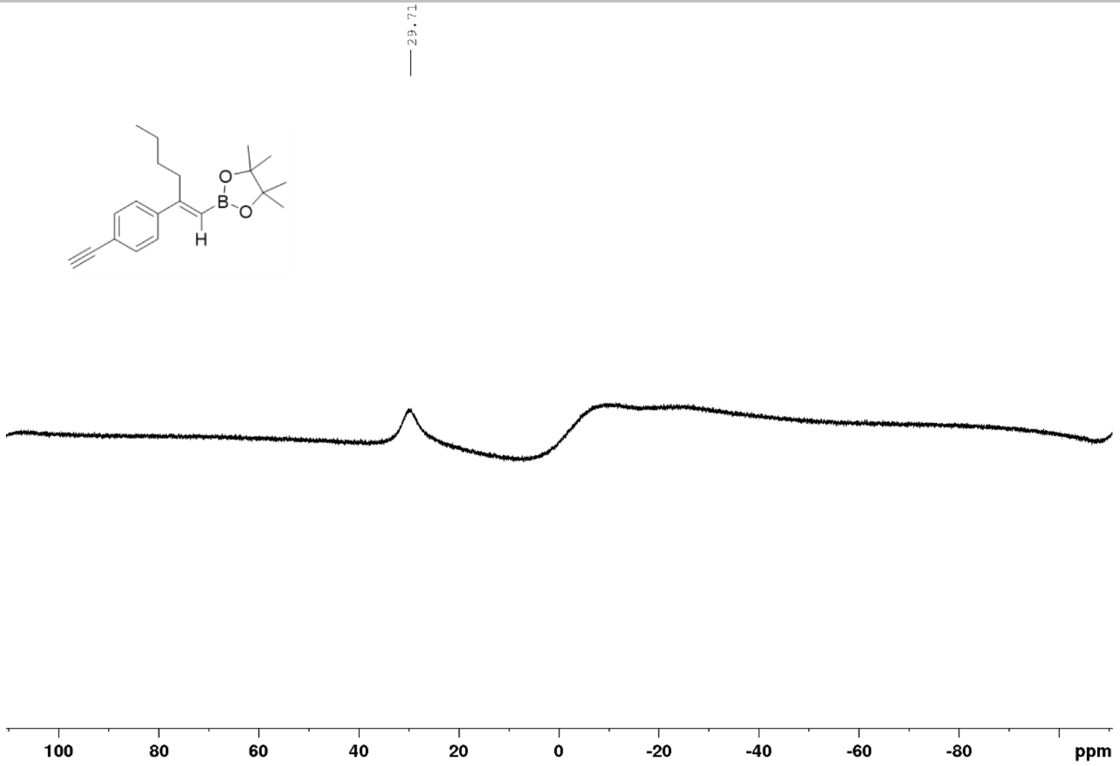
29.71
O
B
O
H
100
80
60
40
20
0
-20
-40
-60
-80
ppm

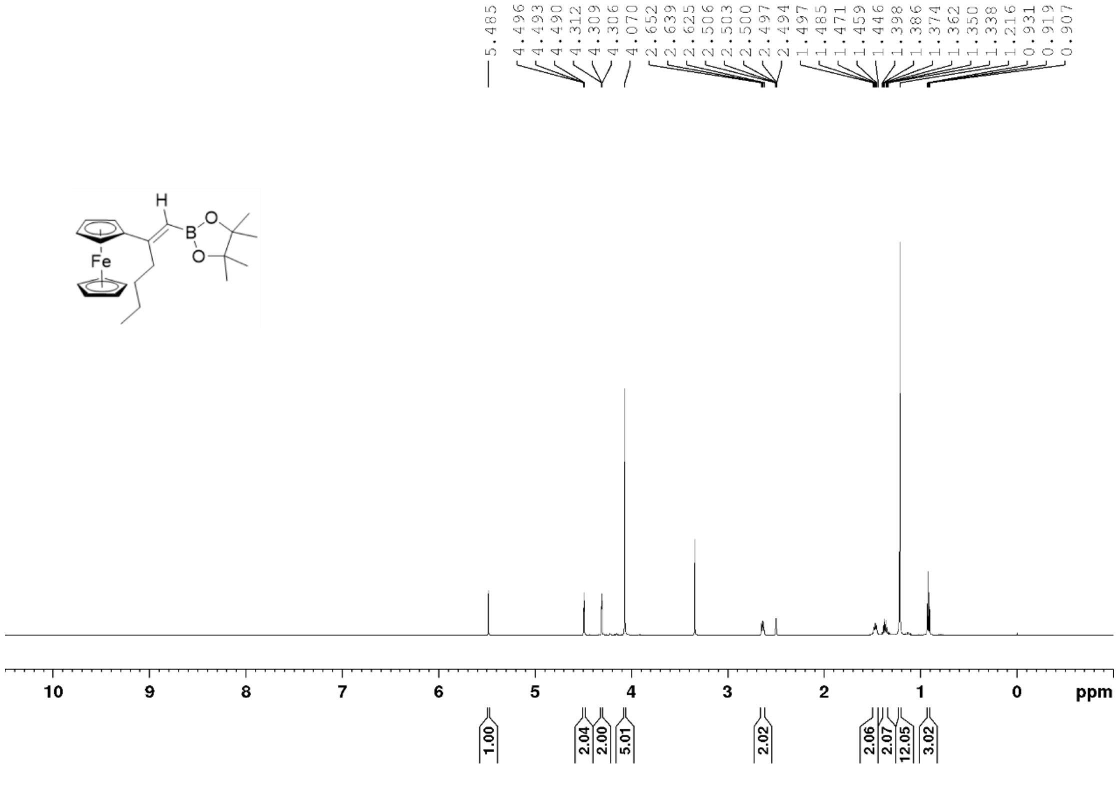
5.485
4.496
4.493
4.490
4.312
4.309
4.306
4.070
2.652
2.639
2.625
2.506
2.503
2.500
2.497
2.494
1.497
1.485
1.471
1.459
1.446
1.398
1.386
1.374
1.362
1.350
1.338
1.216
0.931
0.919
0.907
H
B
O
O
Fe
10
9
8
7
6
5
4
3
2
1
0
ppm
1.00
2.04
2.00
5.01
2.02
2.06
2.07
12.05
3.02

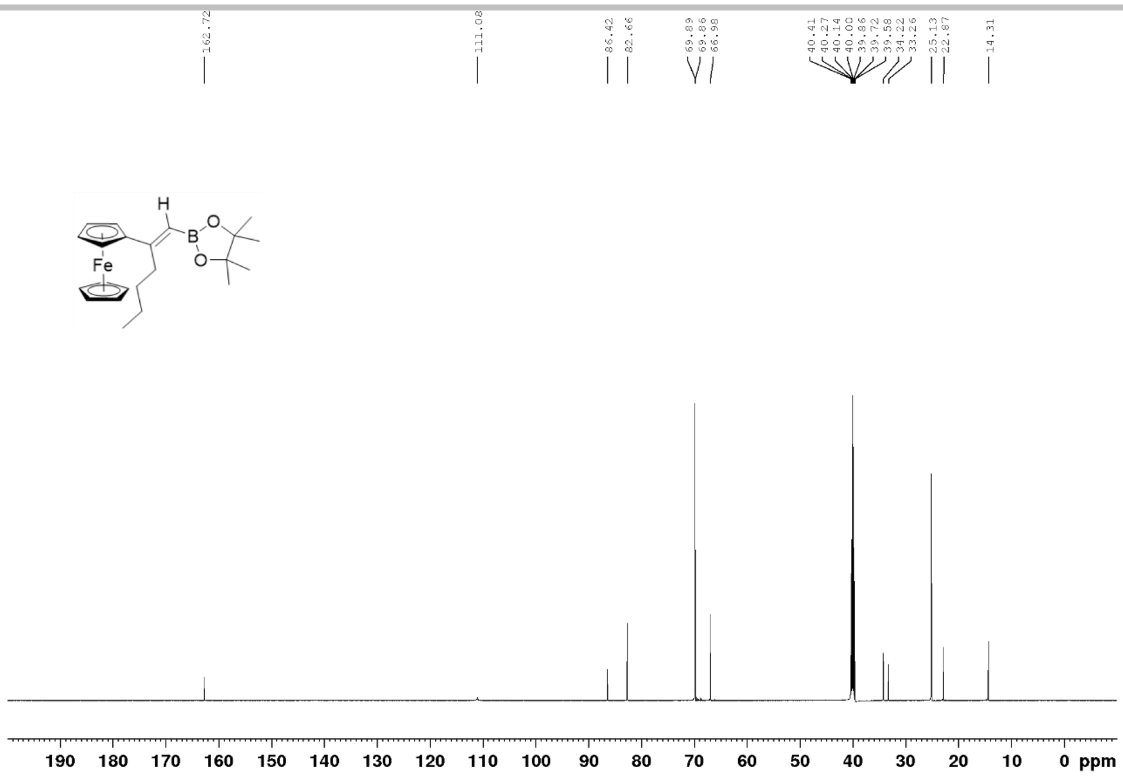

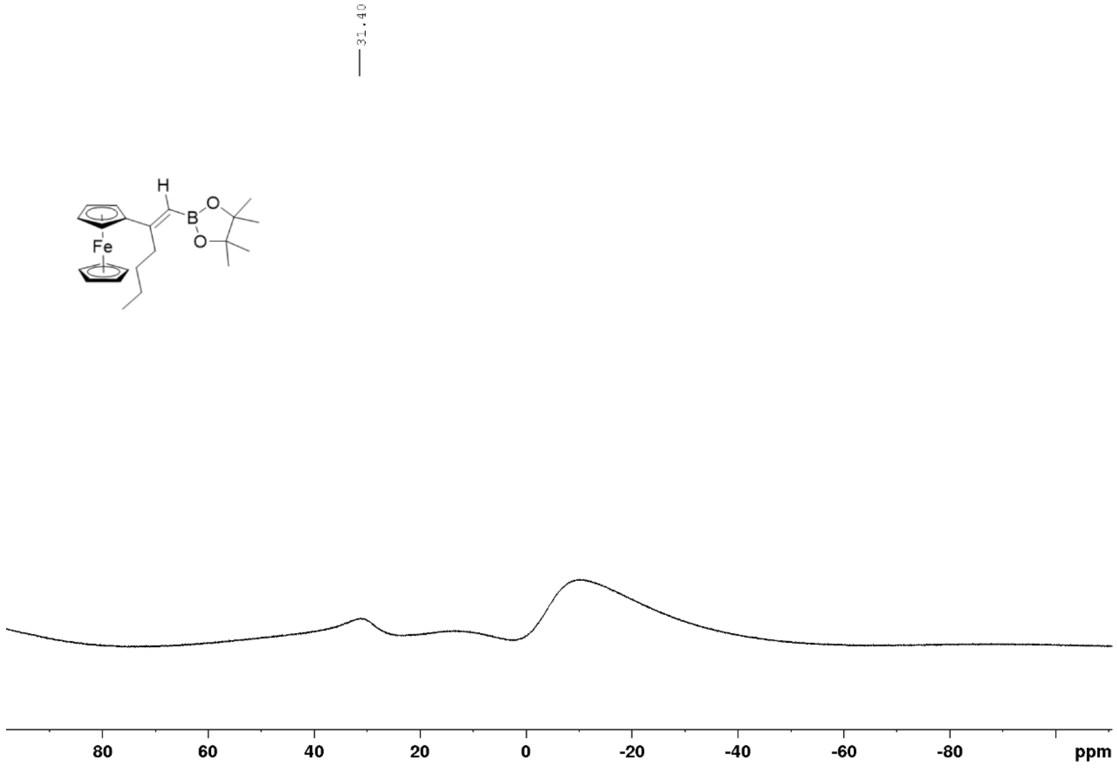

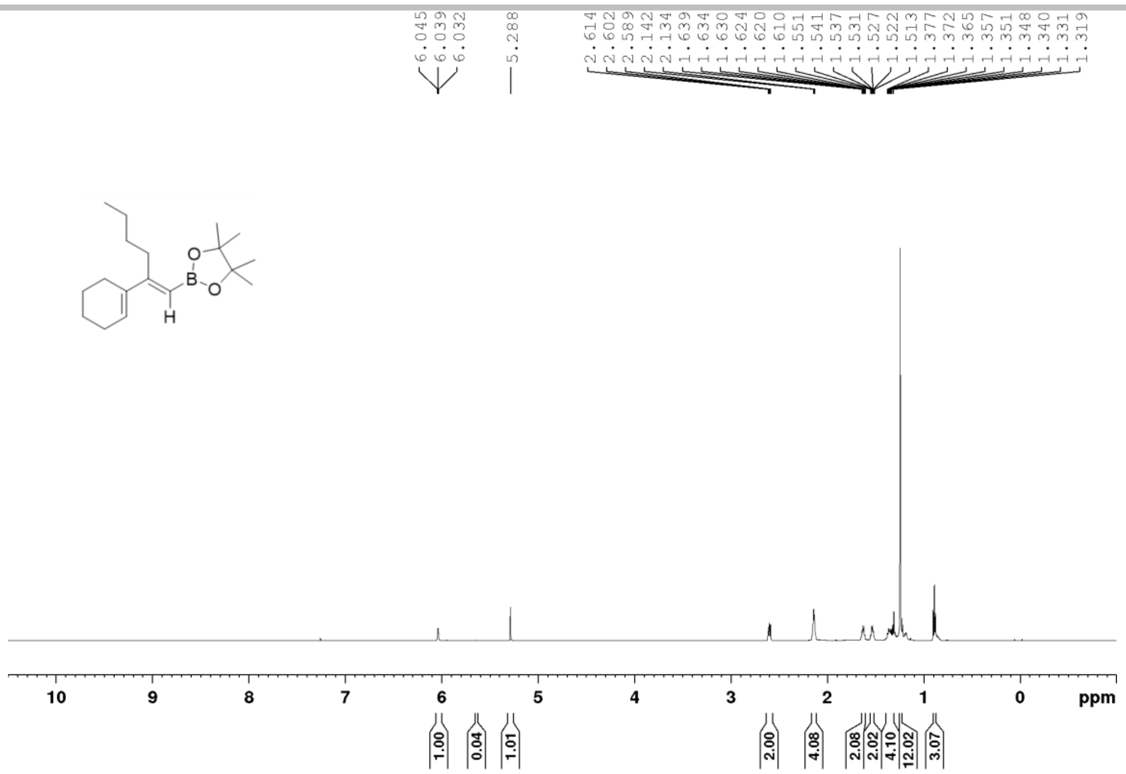

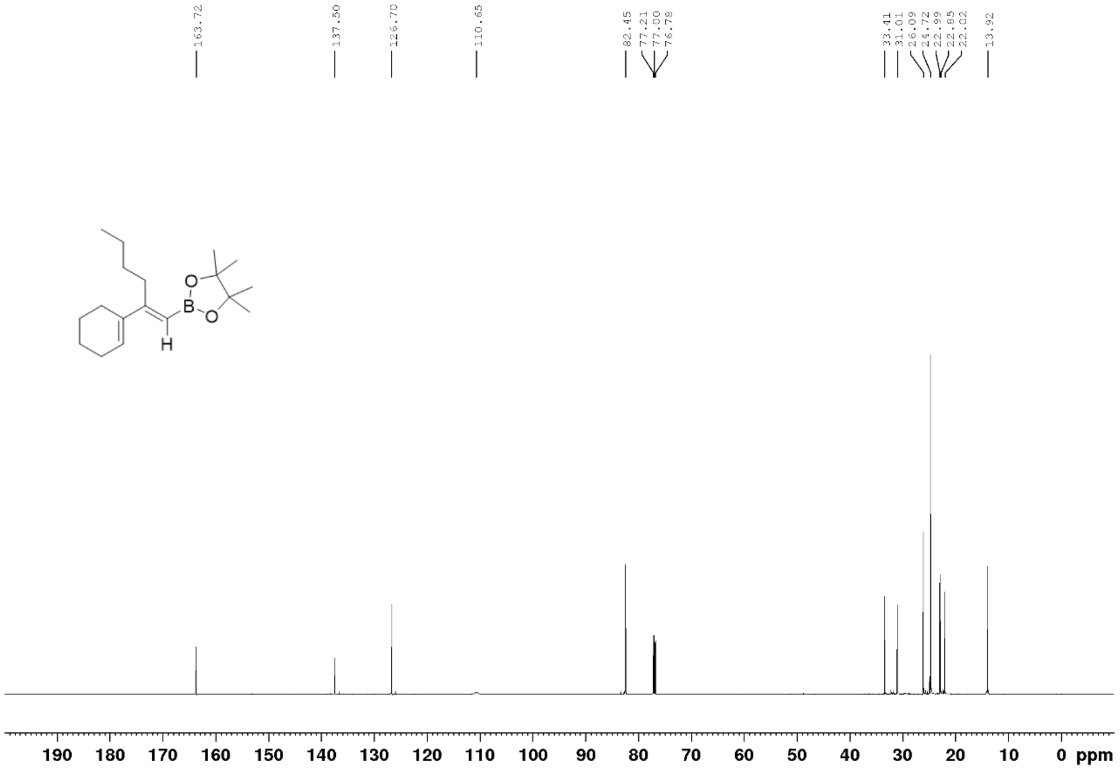

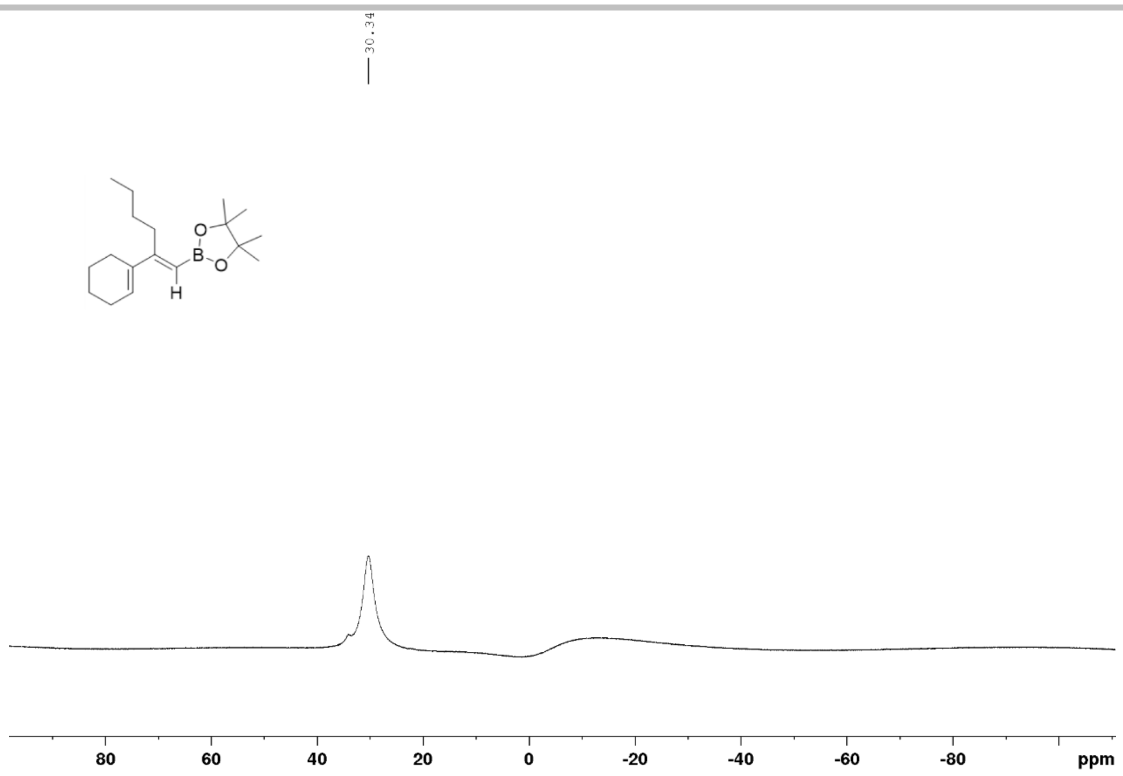
30.34
O
B
O
H
80
60
40
20
0
-20
-40
-60
-80
ppm

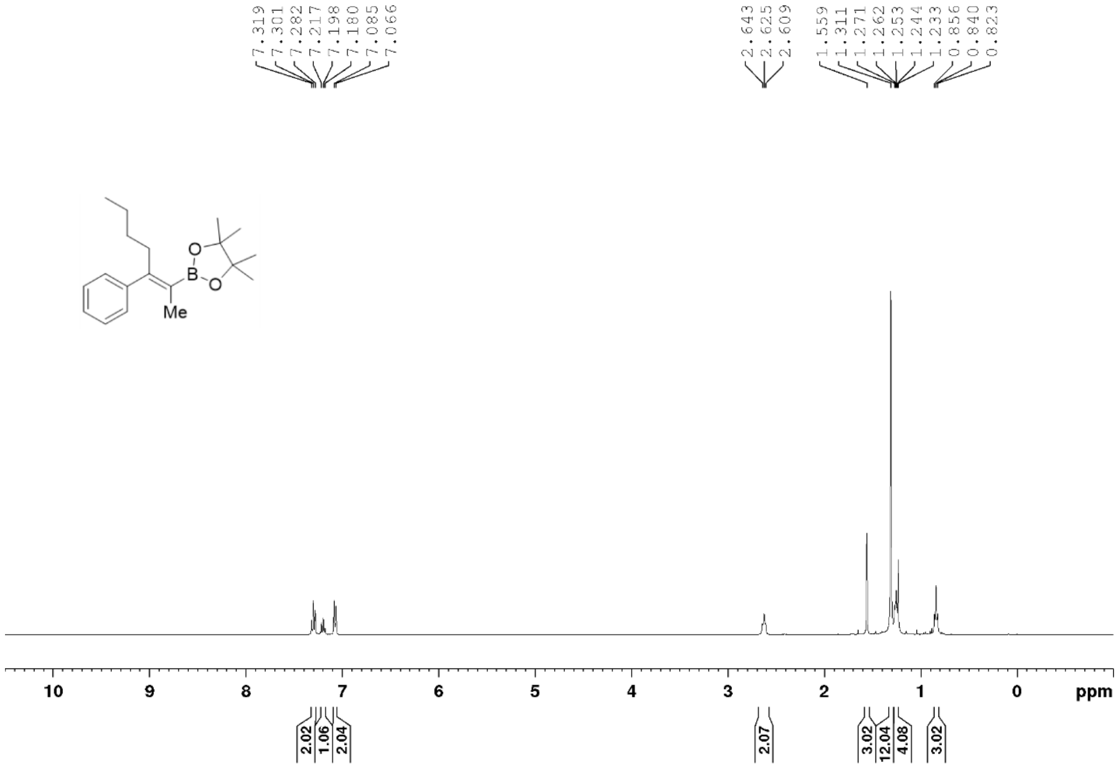
7.319
7.301
7.282
7.217
7.198
7.180
7.085
7.066
2.643
2.625
2.609
1.559
1.311
1.271
1.262
1.253
1.244
1.233
0.856
0.840
0.823
O
B
O
Me
10
9
8
7
6
5
4
3
2
1
0
ppm
2.02
1.06
2.04
2.07
3.02
12.04
4.08
3.02

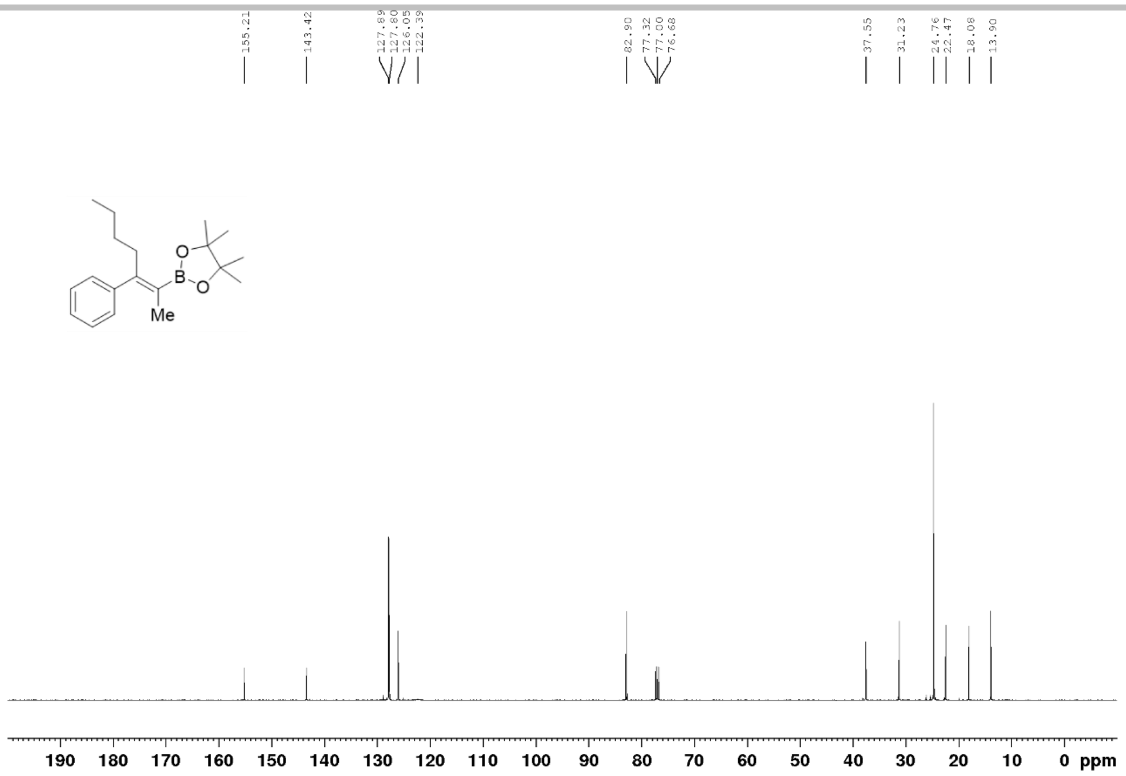
155.21
143.42
127.89
127.80
126.05
122.39
82.90
77.32
77.00
76.68
37.55
31.23
24.76
22.47
18.08
13.90
O
B
O
Me
190 180 170 160 150 140 130 120 110 100 90 80 70 60 50 40 30 20 10 0 ppm

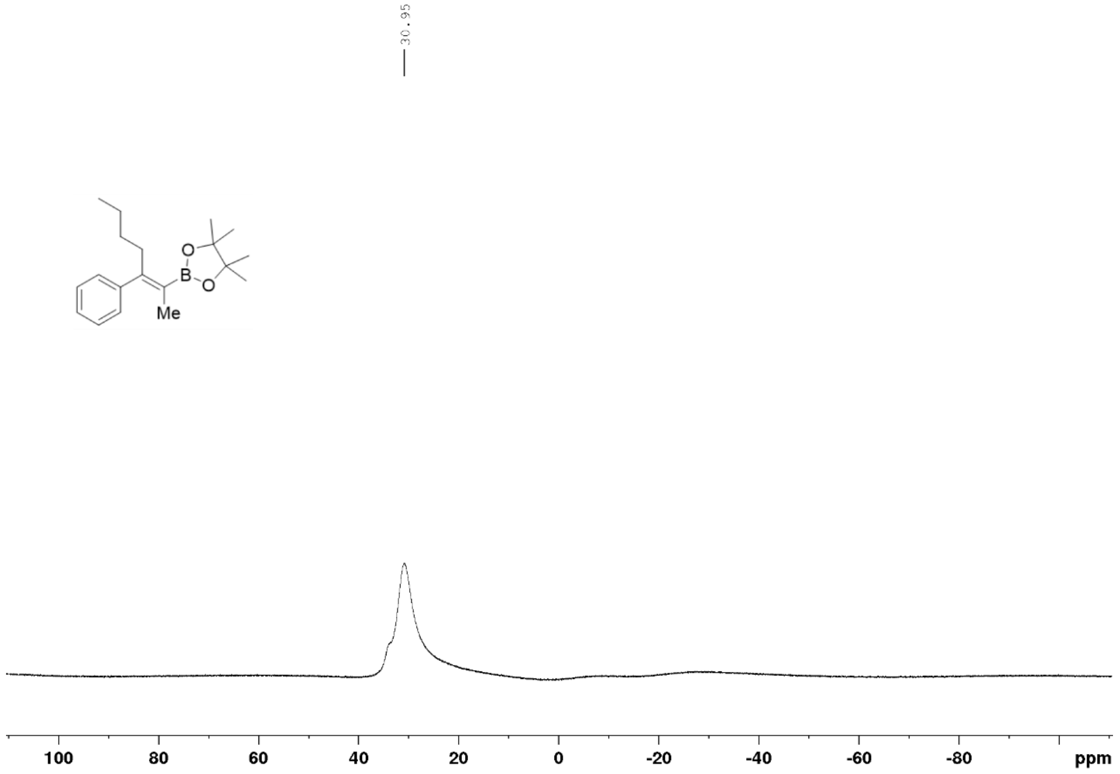
30.95
O
B
O
Me
100 80 60 40 20 0 -20 -40 -60 -80 ppm

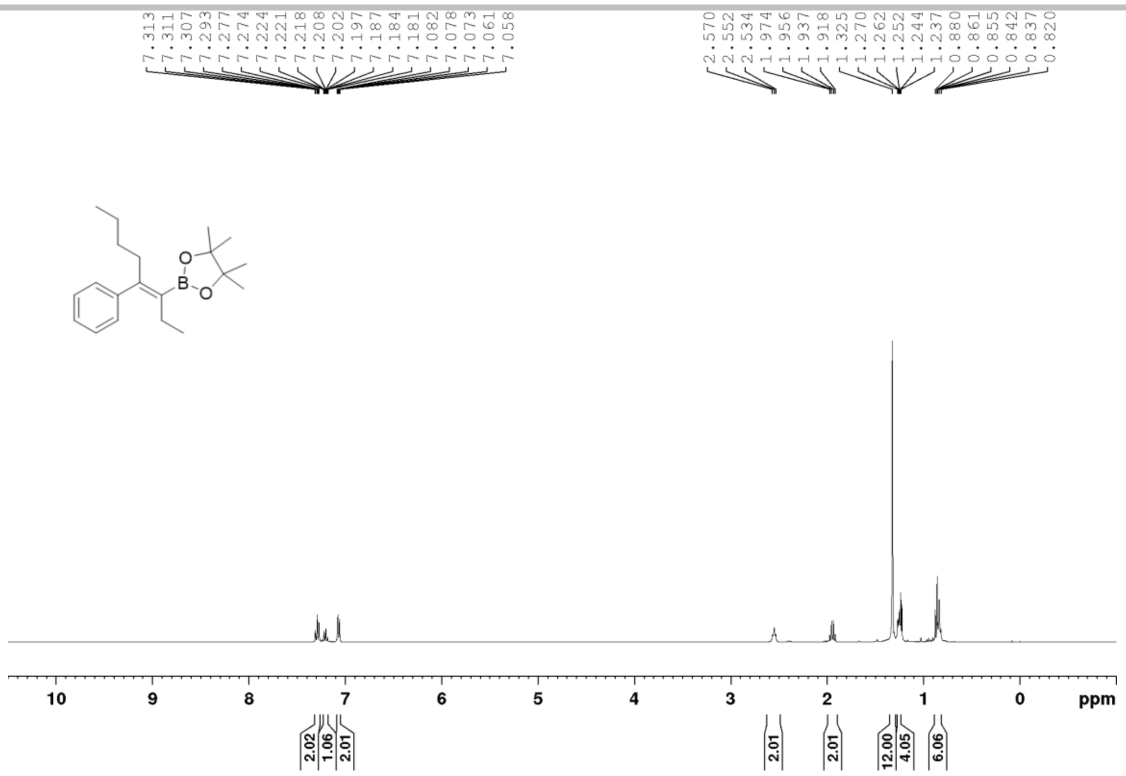

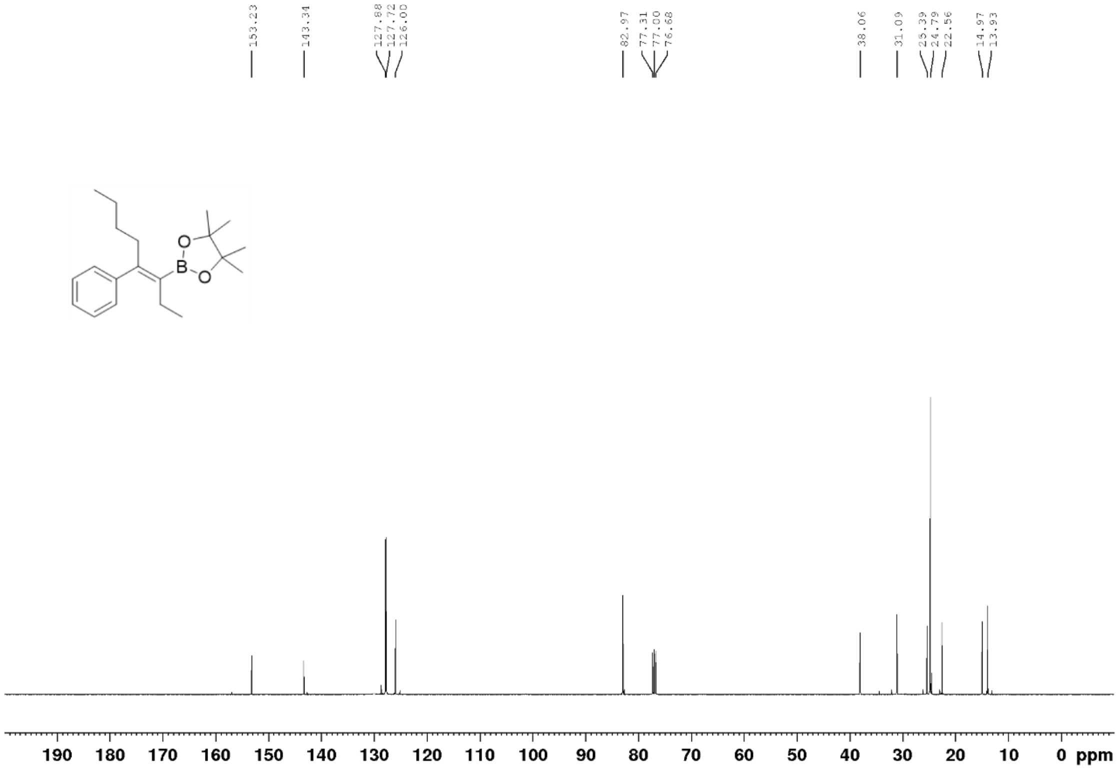

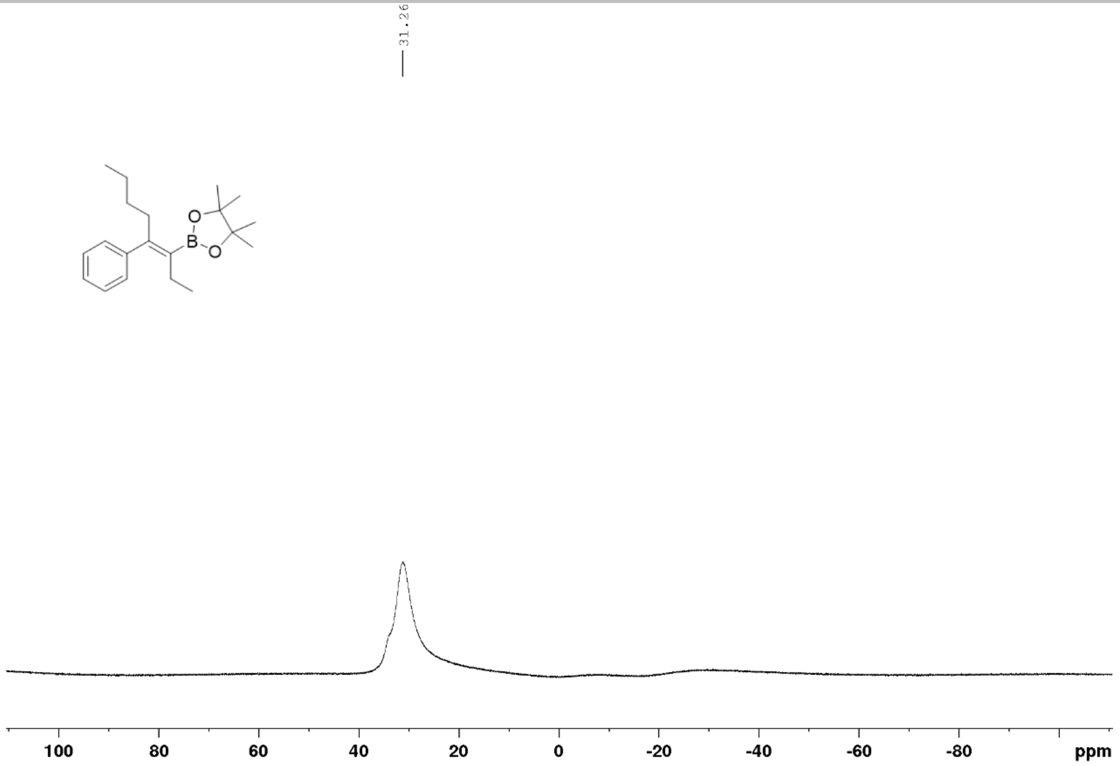
31.26
B
O
O
100
80
60
40
20
0
-20
-40
-60
-80
ppm

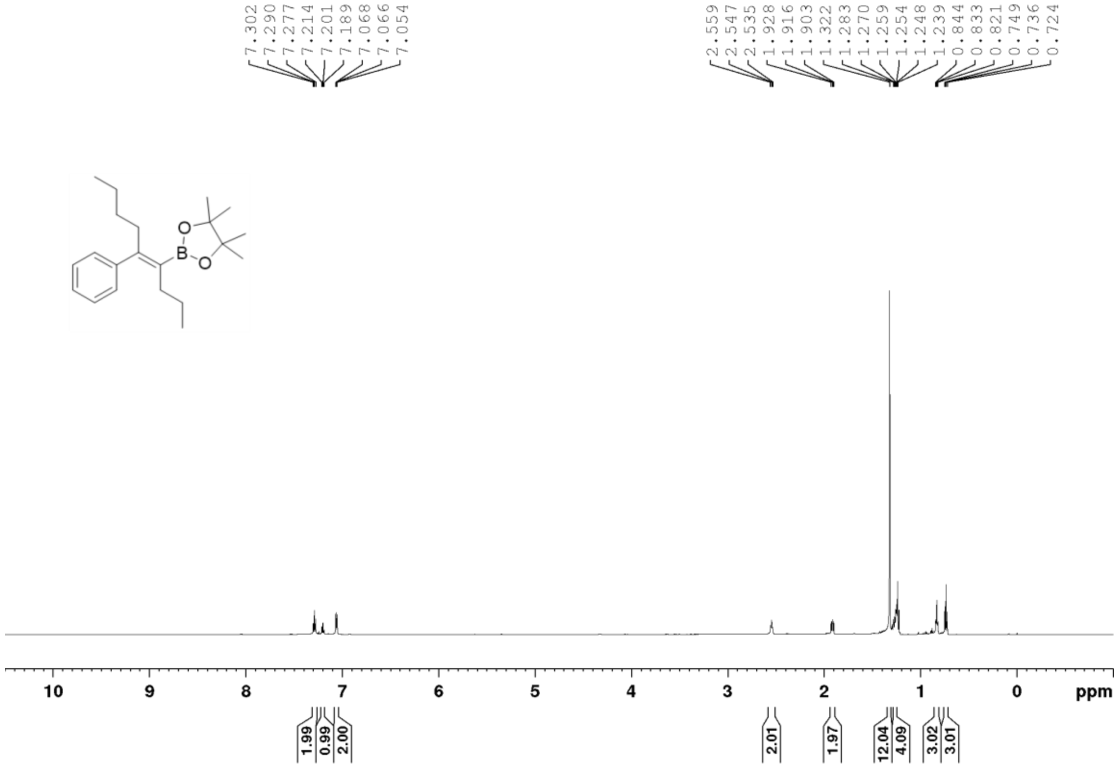
7.302
7.290
7.277
7.214
7.201
7.189
7.068
7.066
7.054
2.559
2.547
2.535
1.928
1.916
1.903
1.322
1.283
1.270
1.259
1.254
1.248
1.239
0.844
0.833
0.821
0.749
0.736
0.724
B
O
O
10
9
8
7
6
5
4
3
2
1
0
ppm
1.99
0.99
2.00
2.01
1.97
12.04
4.09
3.02
3.01

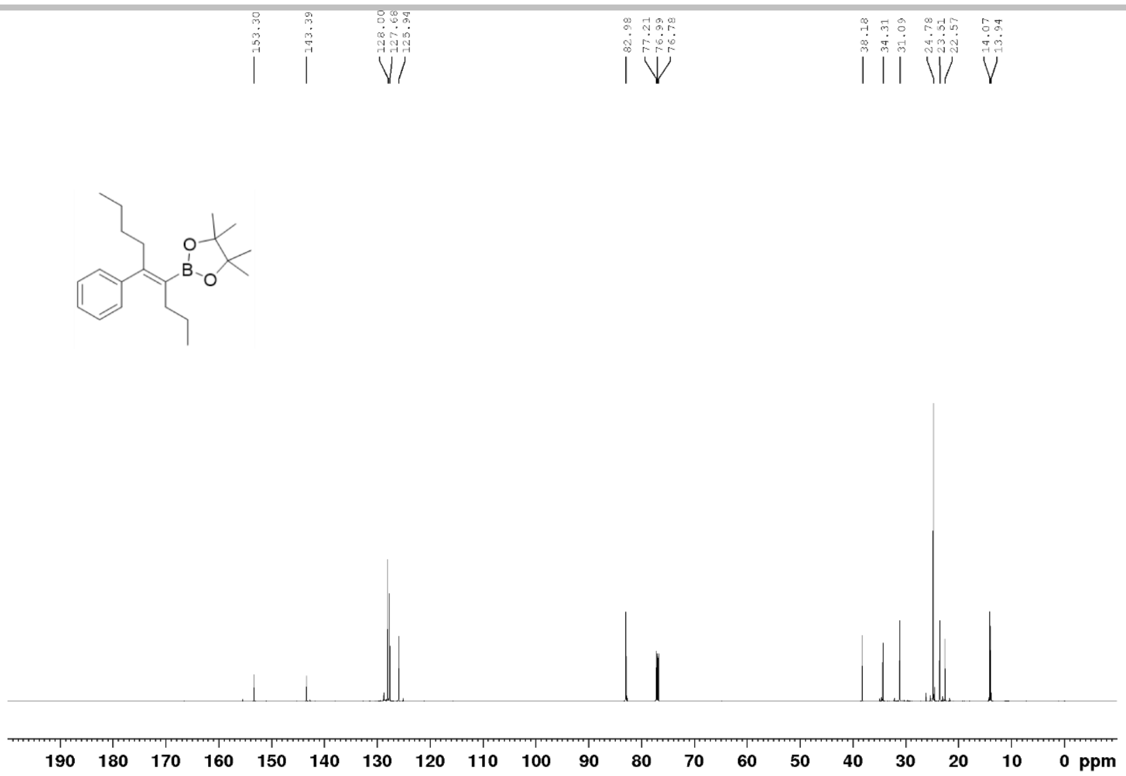
153.30
143.39
128.00
127.68
125.94
82.98
77.21
76.99
76.78
38.18
34.31
31.09
24.78
23.51
22.57
14.07
13.94
O
B
O
190 180 170 160 150 140 130 120 110 100 90 80 70 60 50 40 30 20 10 0 ppm

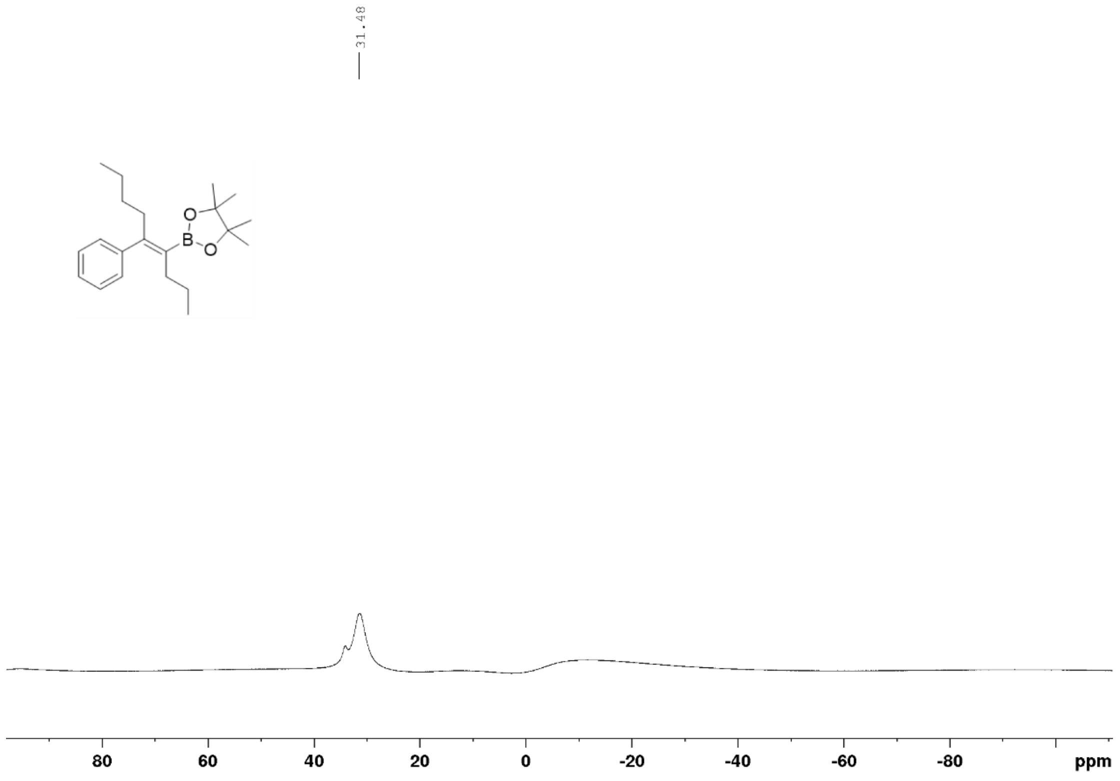
31.48
O
B
O
80 60 40 20 0 -20 -40 -60 -80 ppm

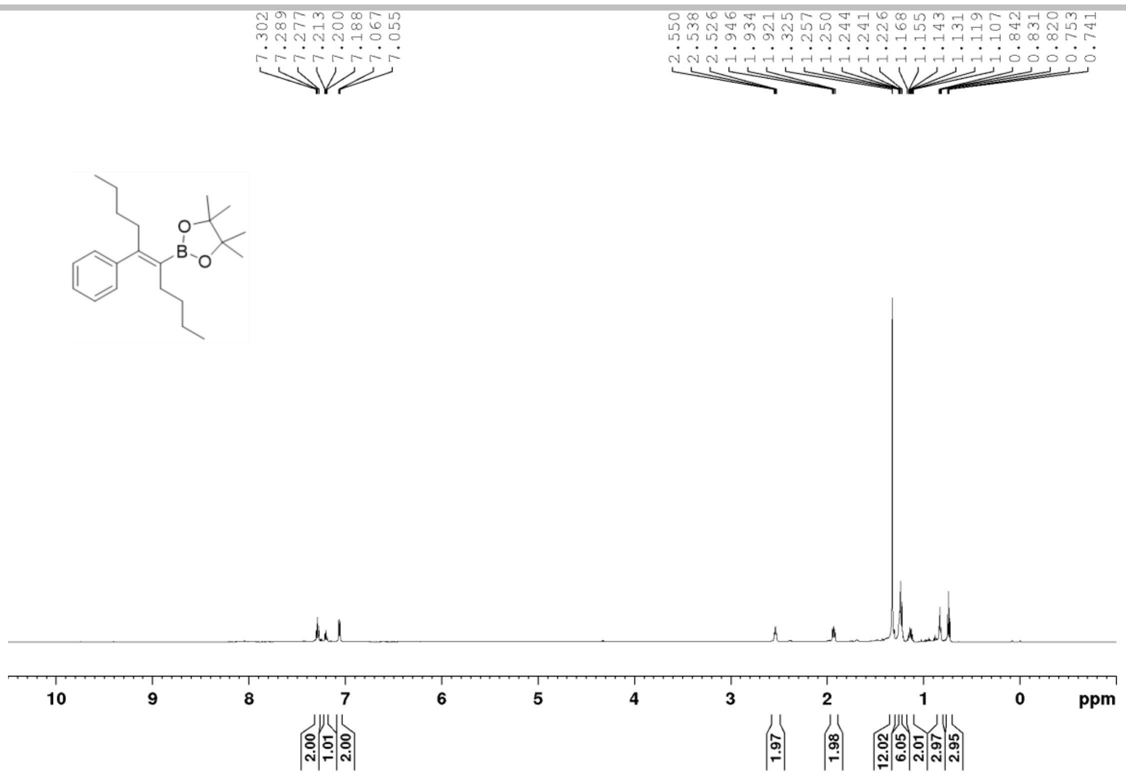
7.302
7.289
7.277
7.213
7.200
7.188
7.067
7.055
2.550
2.538
2.526
1.946
1.934
1.921
1.325
1.257
1.250
1.244
1.241
1.226
1.168
1.155
1.143
1.131
1.119
1.107
0.842
0.831
0.820
0.753
0.741
O
B
O
10 9 8 7 6 5 4 3 2 1 0 ppm
2.00
1.01
2.00
1.97
1.98
12.02
6.05
2.01
2.97
2.95

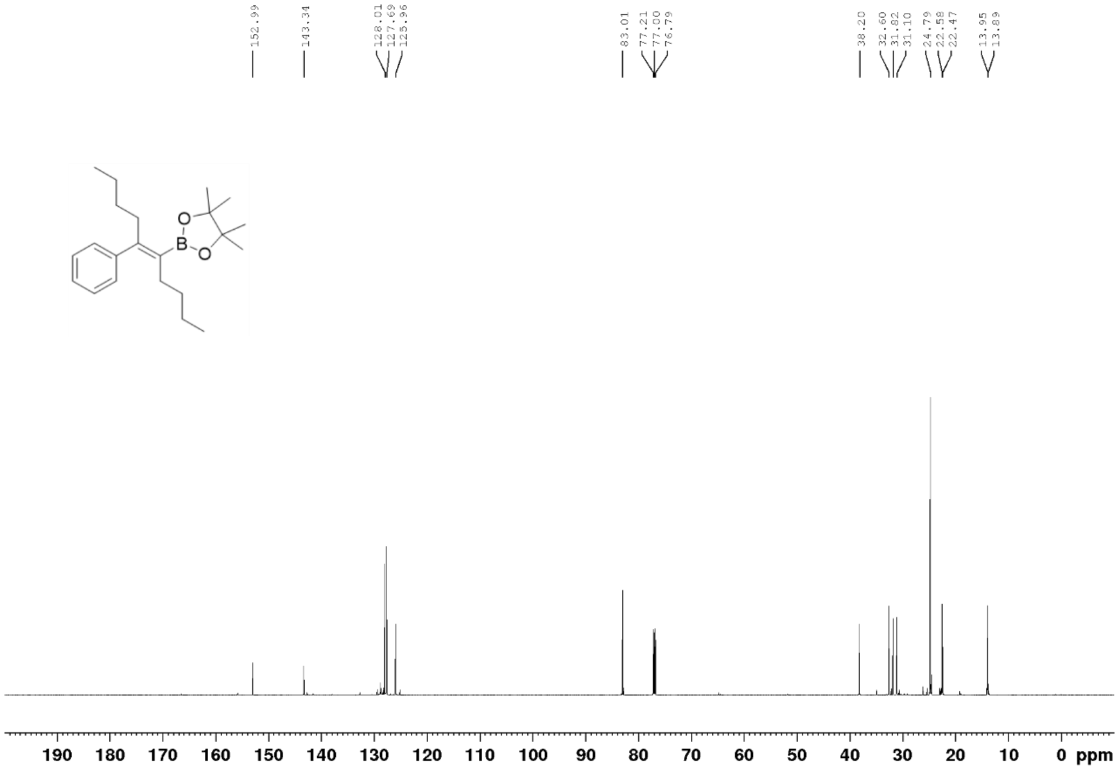
152.99
143.34
128.01
127.69
125.96
83.01
77.21
77.00
76.79
38.20
32.60
31.82
31.10
24.79
22.58
22.47
13.95
13.89
O
B
O
190 180 170 160 150 140 130 120 110 100 90 80 70 60 50 40 30 20 10 0 ppm

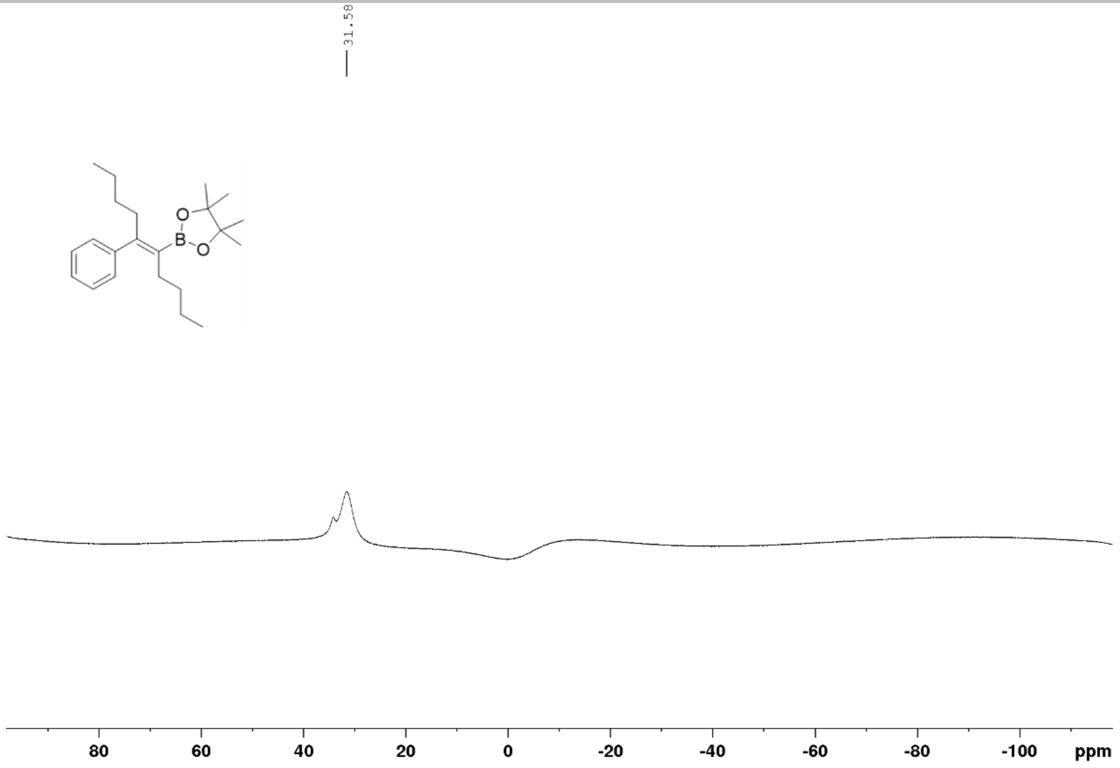
31.58
80
60
40
20
0
-20
-40
-60
-80
-100
ppm

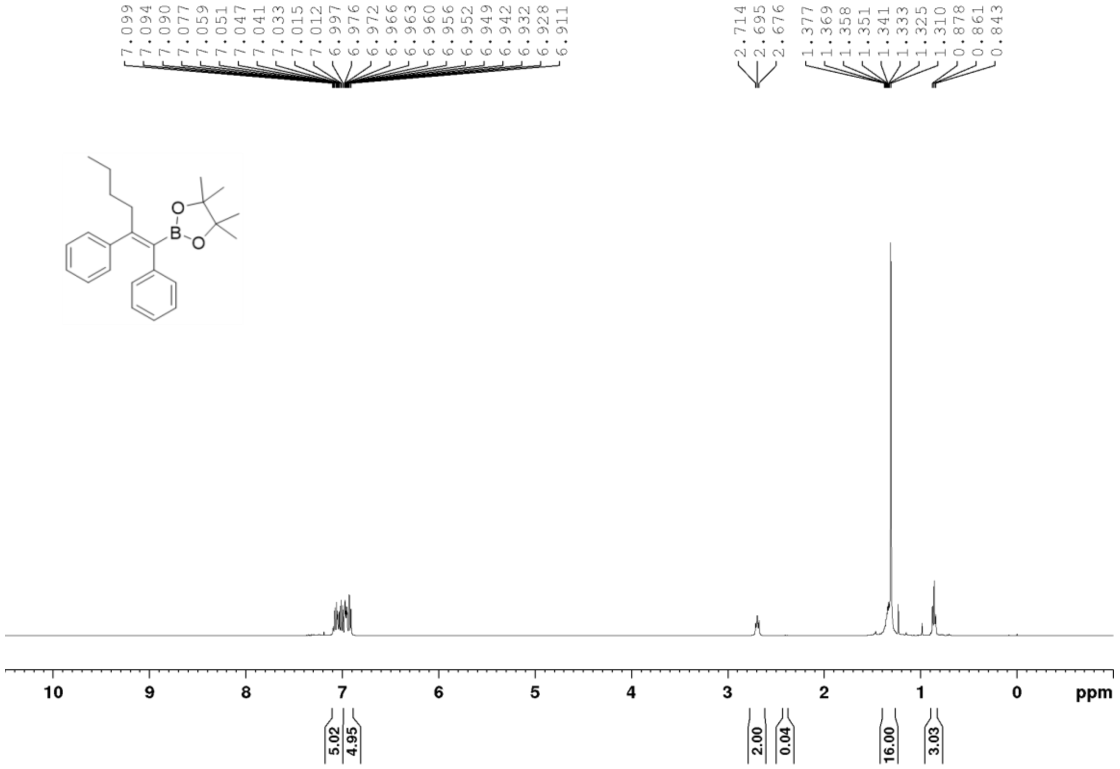
7.099
7.094
7.090
7.077
7.059
7.051
7.047
7.041
7.033
7.015
7.012
6.997
6.976
6.972
6.966
6.963
6.960
6.956
6.952
6.949
6.942
6.932
6.928
6.911
2.714
2.695
2.676
1.377
1.369
1.358
1.351
1.341
1.333
1.325
1.310
0.878
0.861
0.843
10
9
8
7
6
5
4
3
2
1
0
ppm
5.02
4.95
2.00
0.04
16.00
3.03

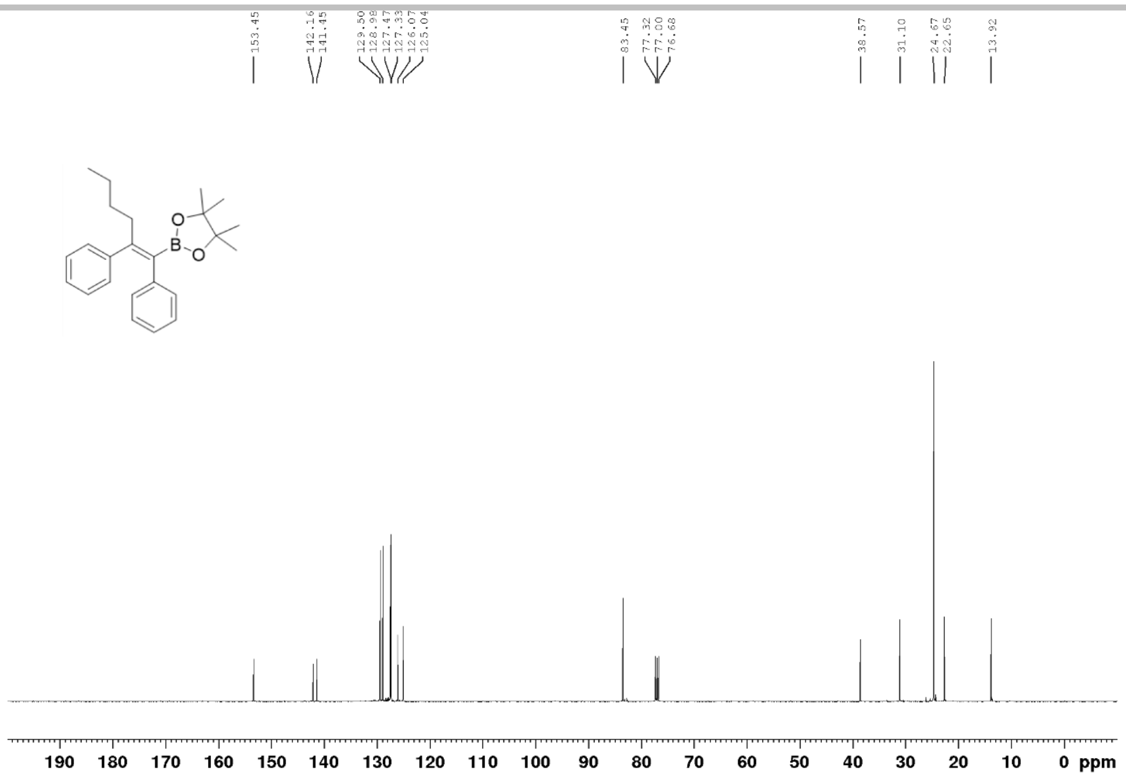
153.45
142.16
141.45
129.50
128.98
127.47
127.33
126.07
125.04
83.45
77.32
77.00
76.68
38.57
31.10
24.67
22.65
13.92
B
O
O
190 180 170 160 150 140 130 120 110 100 90 80 70 60 50 40 30 20 10 0 ppm

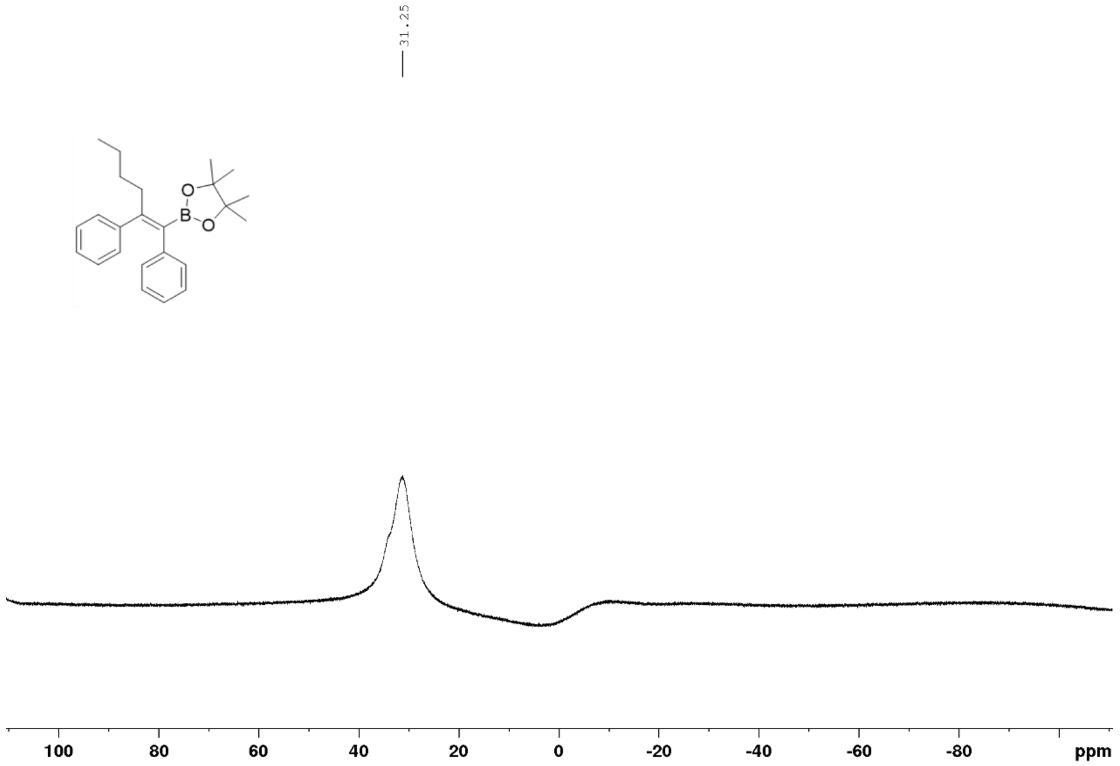
31.25
B
O
O
100 80 60 40 20 0 -20 -40 -60 -80 ppm

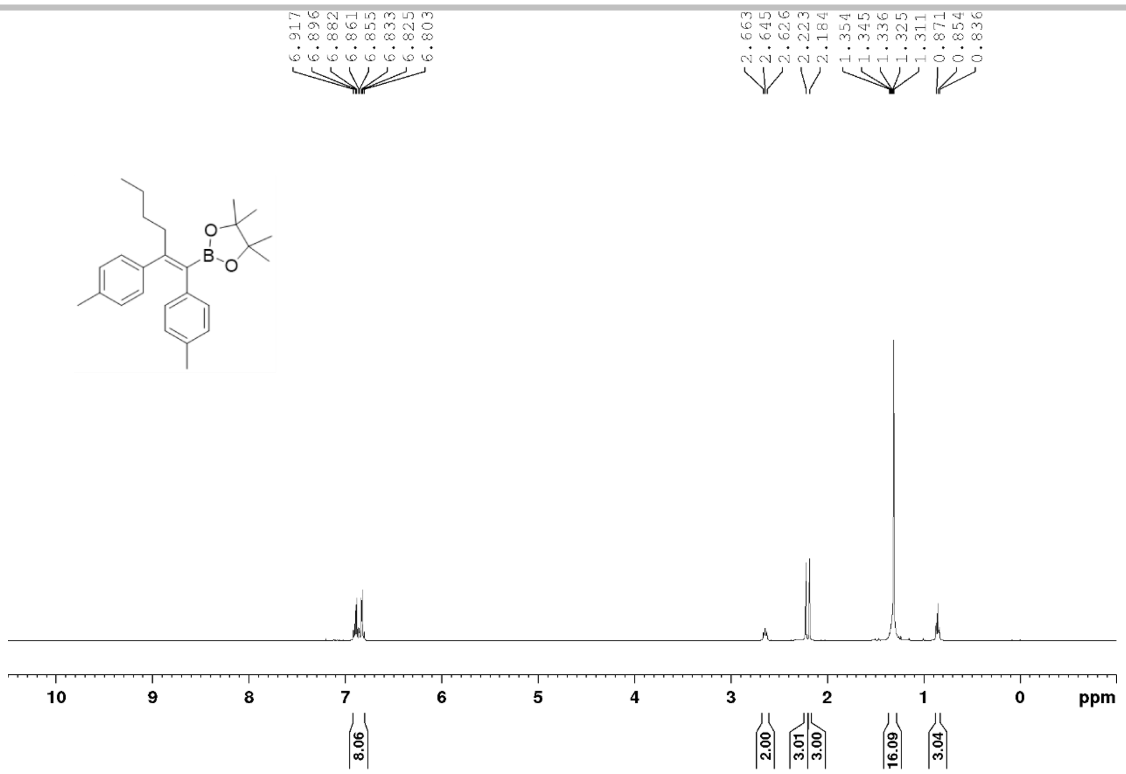
6.917
6.896
6.882
6.861
6.855
6.833
6.825
6.803
2.663
2.645
2.626
2.223
2.184
1.354
1.345
1.336
1.325
1.311
0.871
0.854
0.836
O
B
O
10
9
8
7
6
5
4
3
2
1
0
ppm
8.06
2.00
3.01
3.00
16.09
3.04

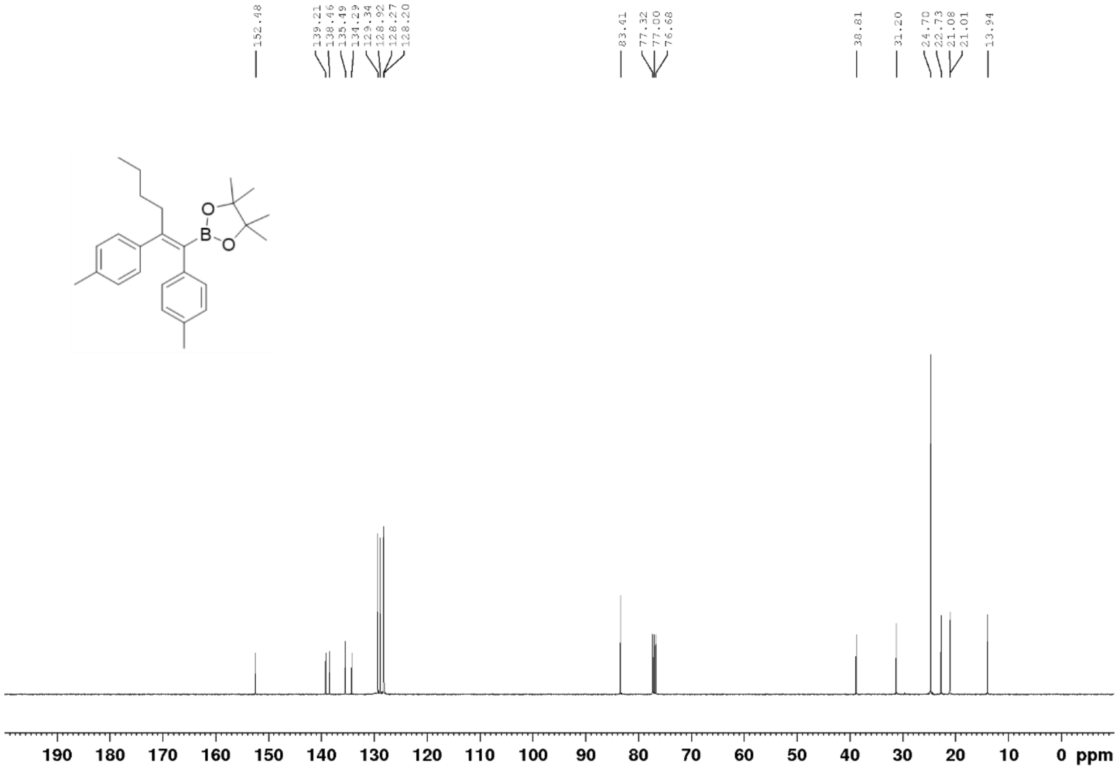
152.48
139.21
138.46
135.49
134.29
129.34
128.92
128.27
128.20
83.41
77.32
77.00
76.68
38.81
31.20
24.70
22.73
21.08
21.01
13.94
O
B
O
190
180
170
160
150
140
130
120
110
100
90
80
70
60
50
40
30
20
10
0
ppm

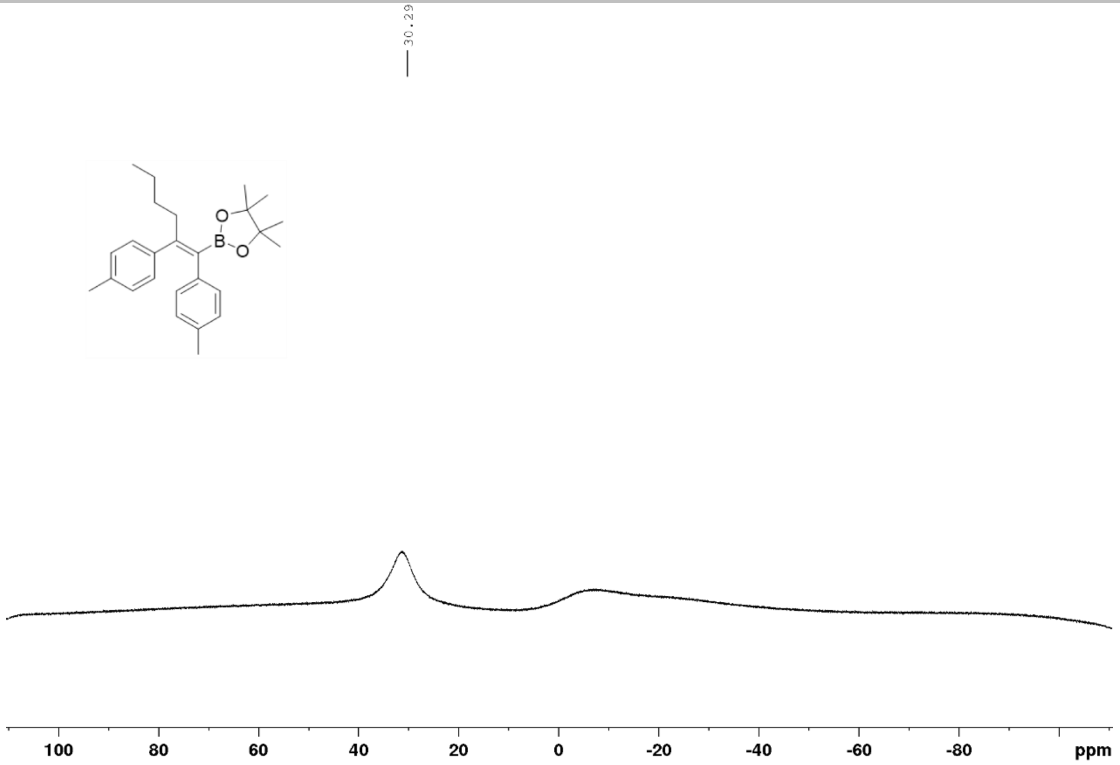
30.29
O
B
O
100
80
60
40
20
0
-20
-40
-60
-80
ppm

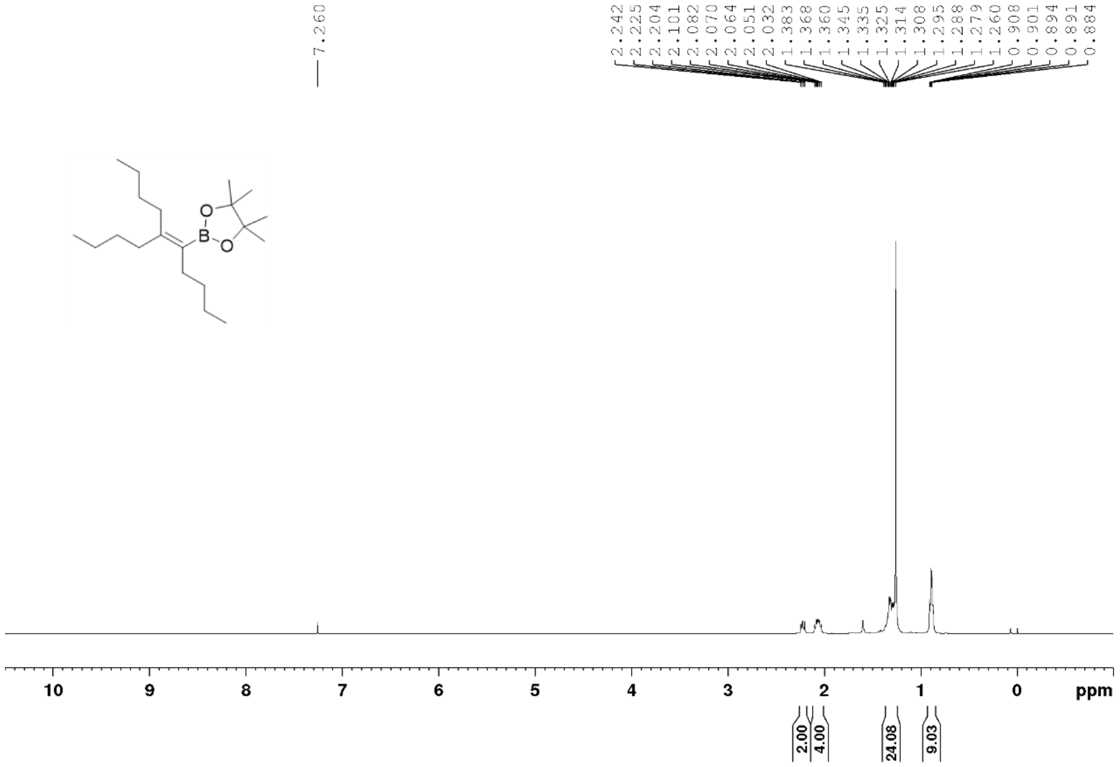
7.260
2.242
2.225
2.204
2.101
2.082
2.070
2.064
2.051
2.032
1.383
1.368
1.360
1.345
1.335
1.325
1.314
1.308
1.295
1.288
1.279
1.260
0.908
0.901
0.894
0.891
0.884
O
B
O
10
9
8
7
6
5
4
3
2
1
0
ppm
2.00
4.00
24.08
9.03

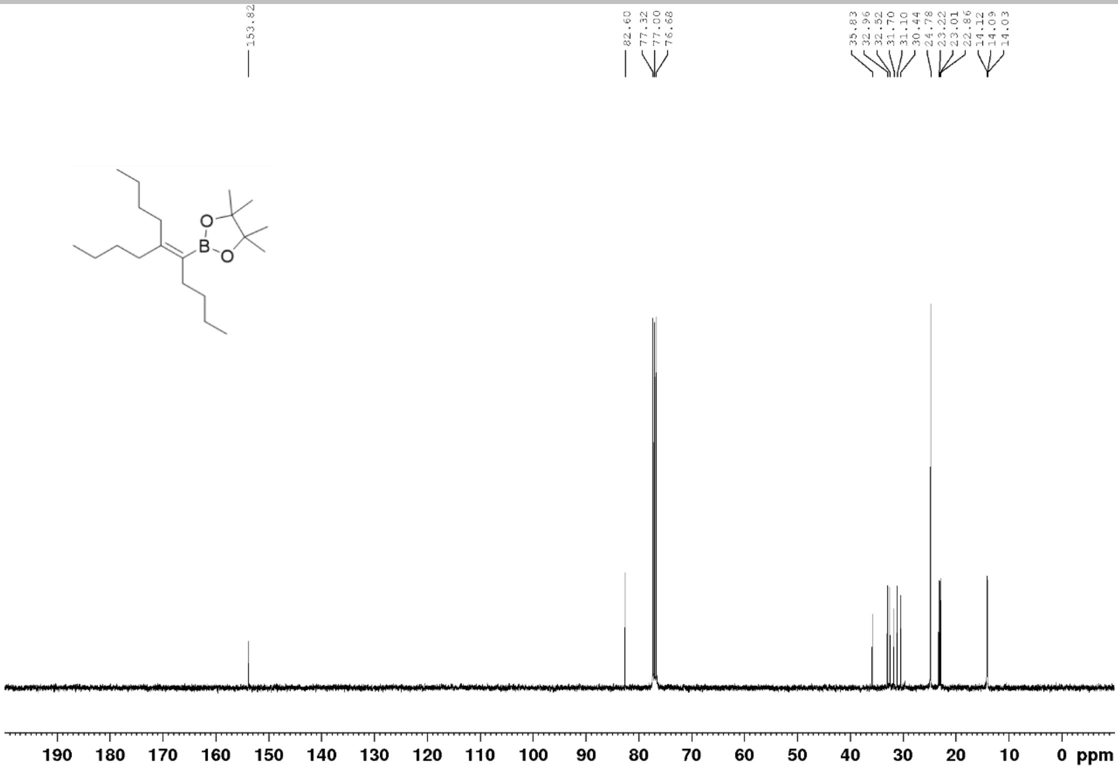
153.82
82.60
77.32
77.00
76.68
35.83
32.96
32.52
31.70
31.10
30.44
24.78
23.22
23.01
22.86
14.12
14.09
14.03
O
B
O
190 180 170 160 150 140 130 120 110 100 90 80 70 60 50 40 30 20 10 0 ppm

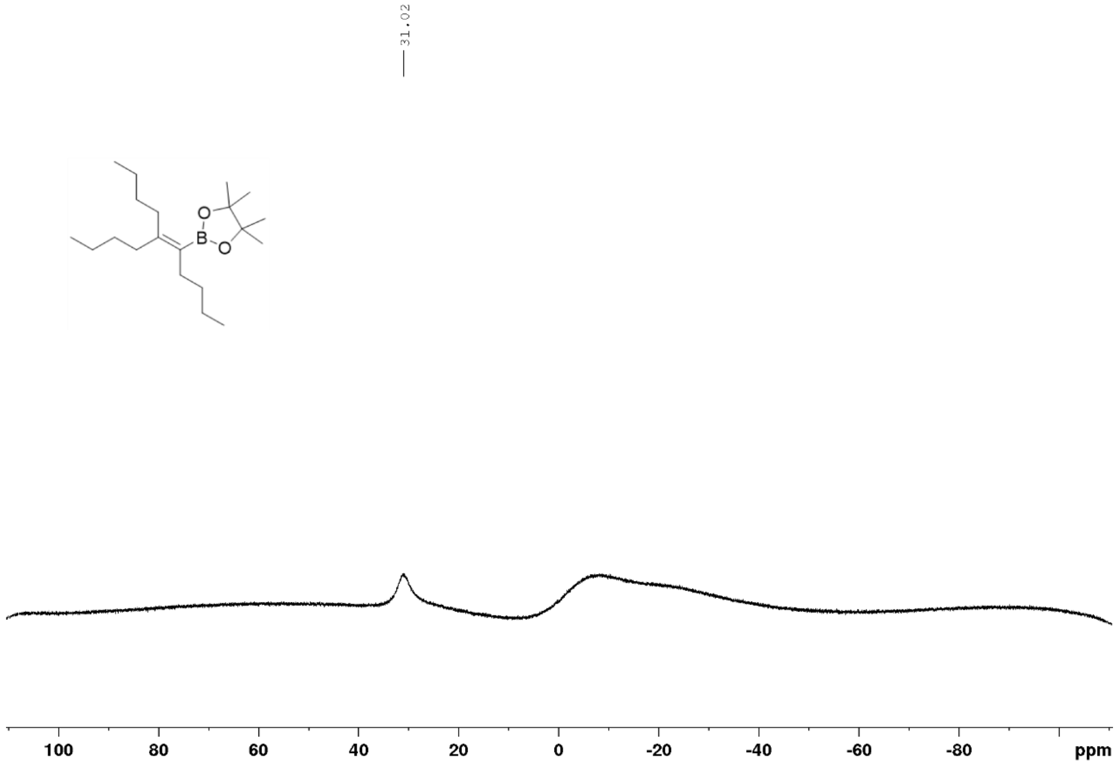
31.02
O
B
O
100 80 60 40 20 0 -20 -40 -60 -80 ppm

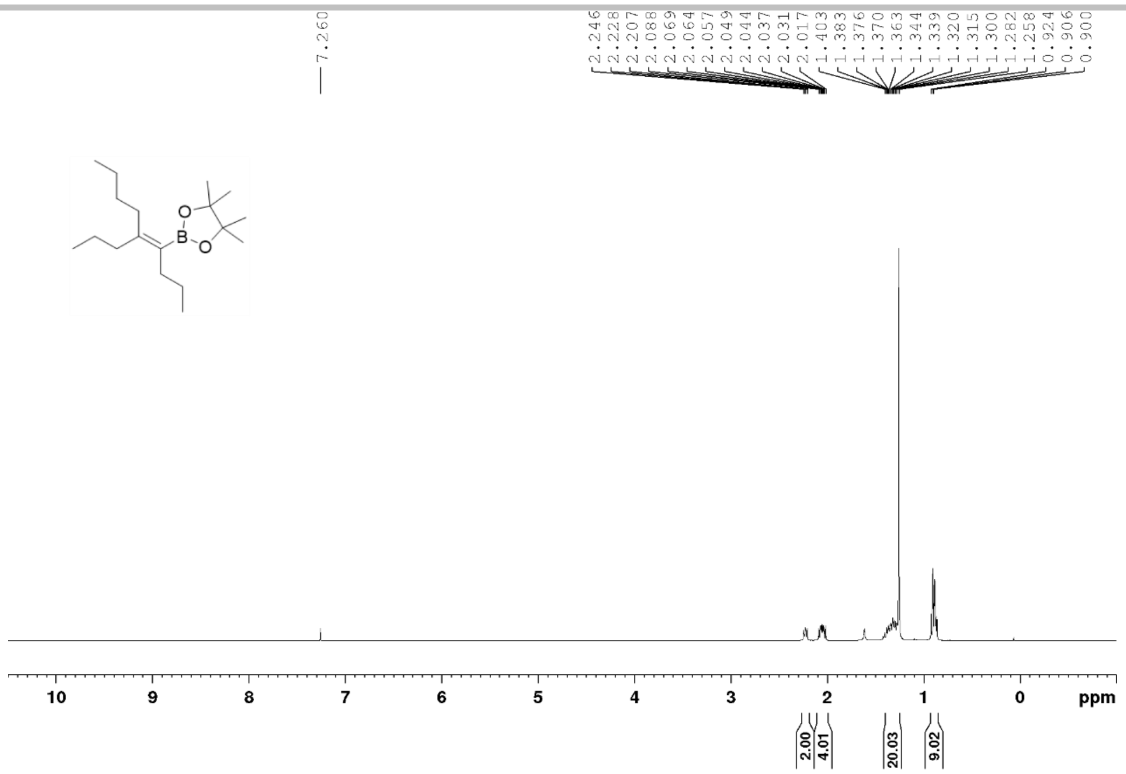

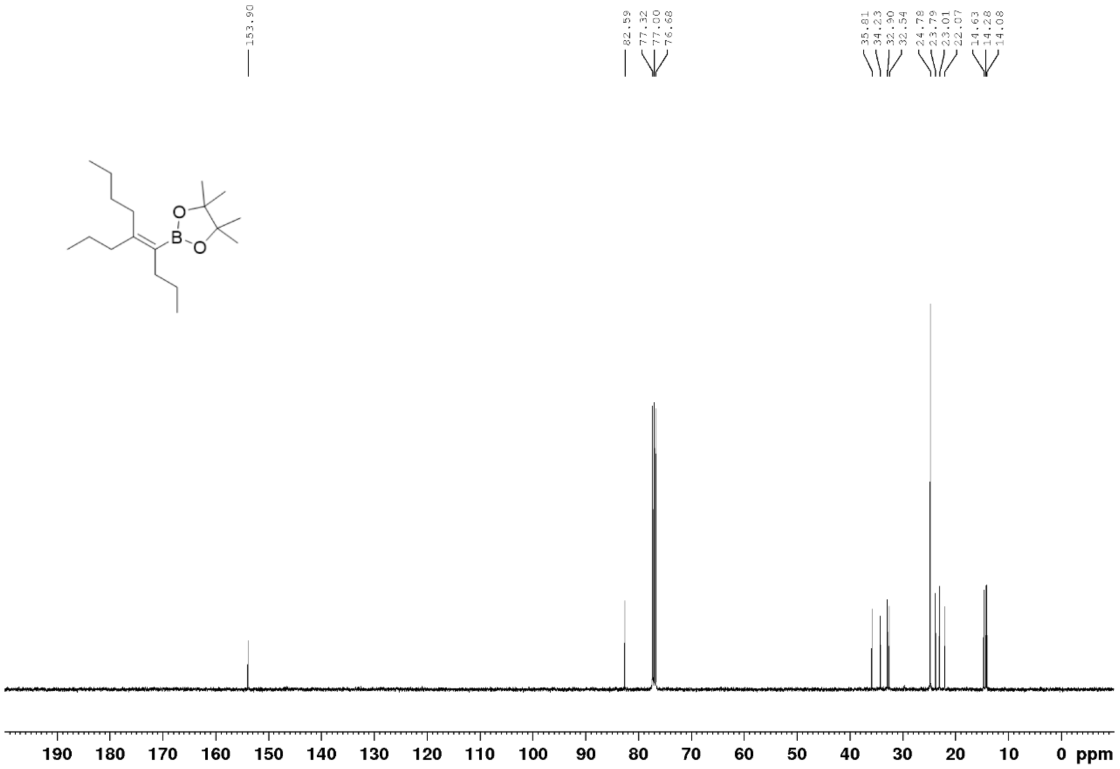

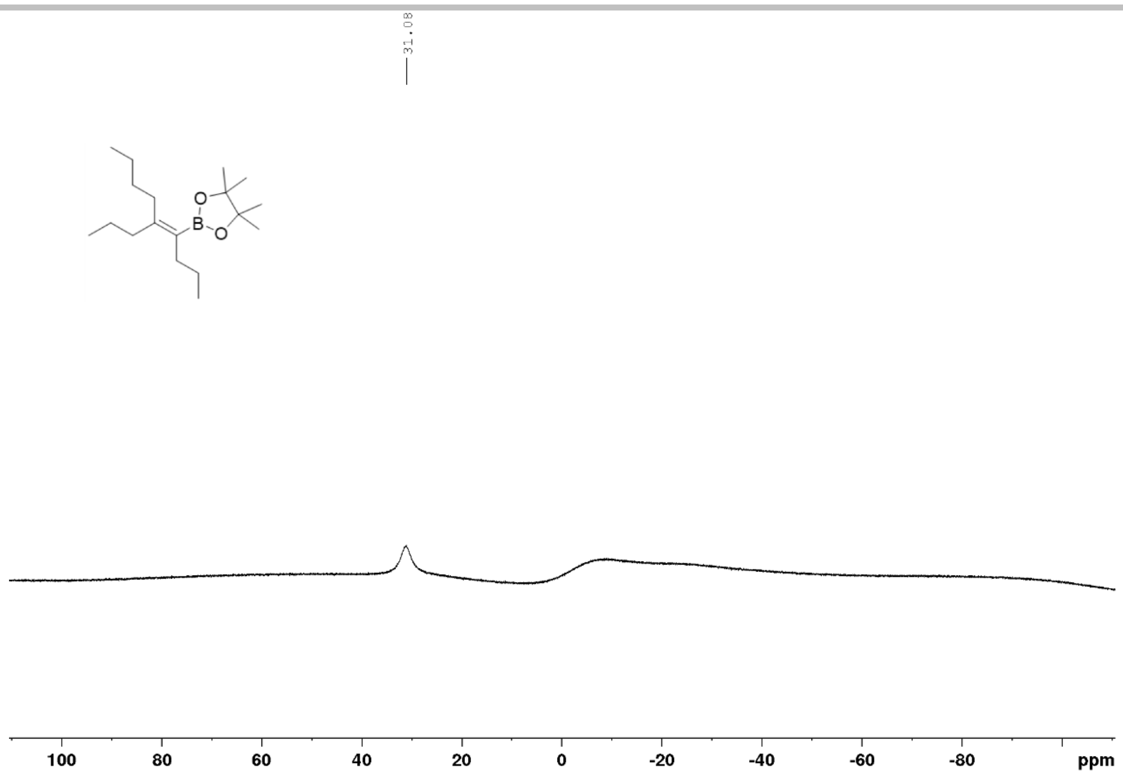
31.08
100
80
60
40
20
0
-20
-40
-60
-80
ppm

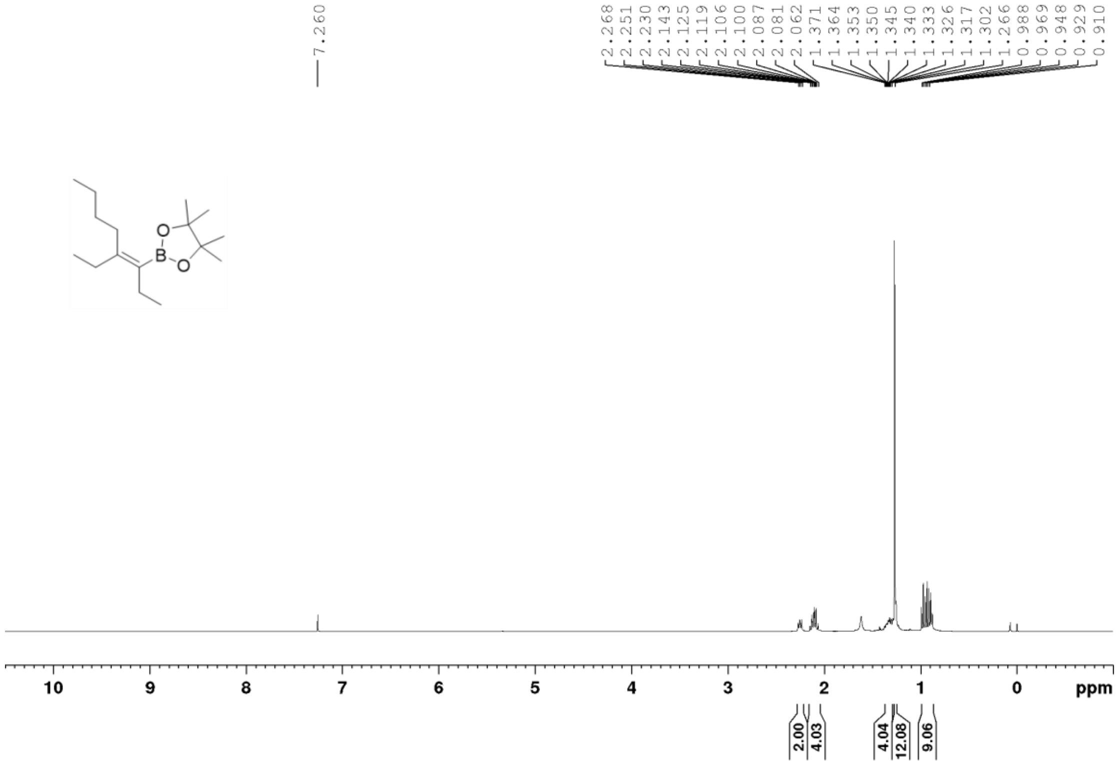
7.260
2.268
2.251
2.230
2.143
2.125
2.119
2.106
2.100
2.087
2.081
2.062
1.371
1.364
1.353
1.350
1.345
1.340
1.333
1.326
1.317
1.302
1.266
0.988
0.969
0.948
0.929
0.910
10
9
8
7
6
5
4
3
2
1
0
ppm
2.00
4.03
4.04
12.08
9.06

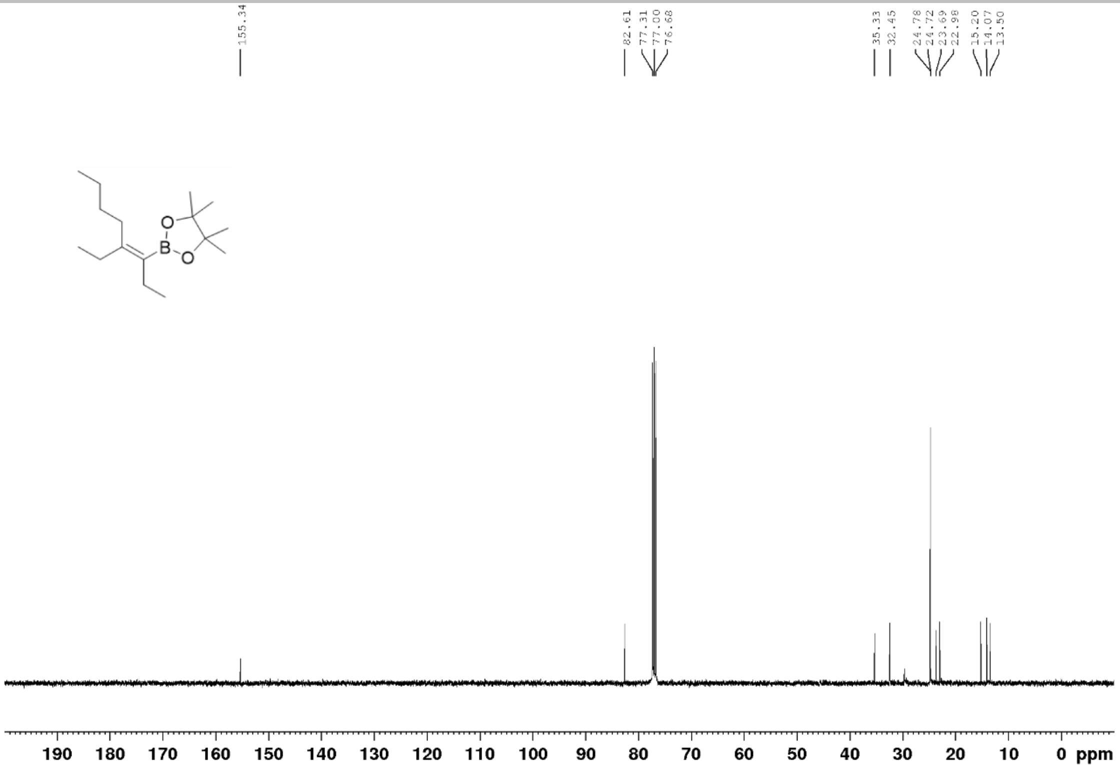
155.34
82.61
77.31
77.00
76.68
35.33
32.45
24.78
24.72
23.69
22.98
15.20
14.07
13.50
190 180 170 160 150 140 130 120 110 100 90 80 70 60 50 40 30 20 10 0 ppm

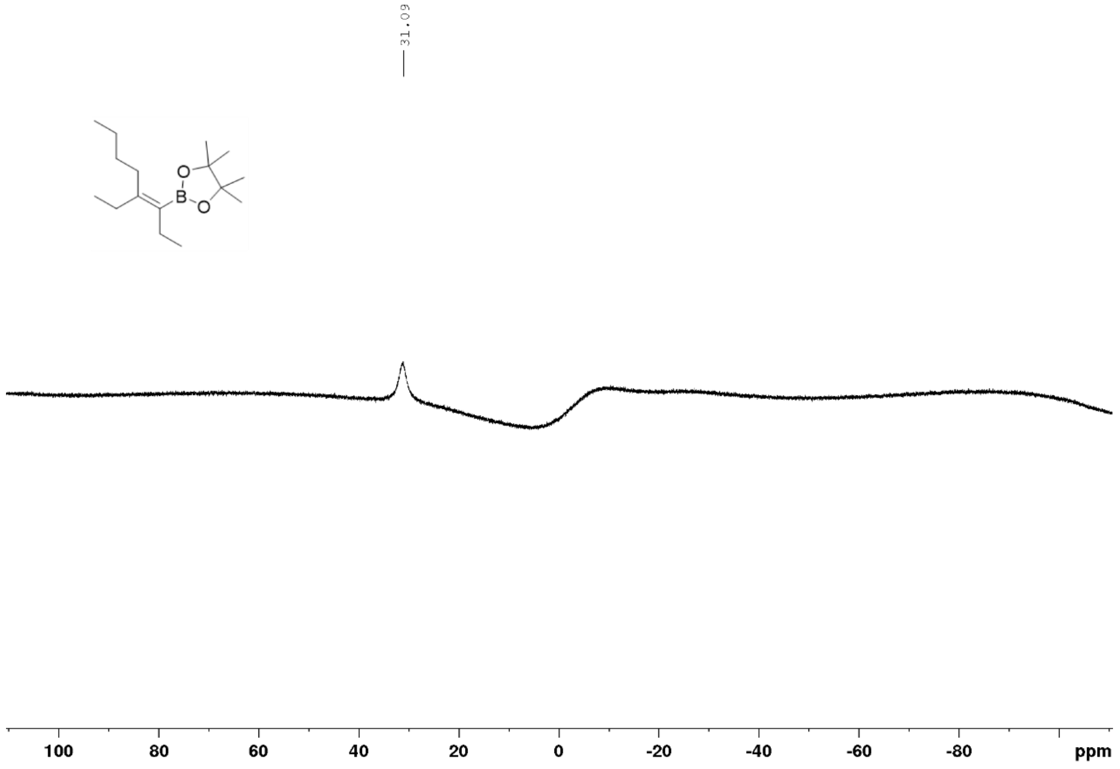
31.09
100 80 60 40 20 0 -20 -40 -60 -80 ppm

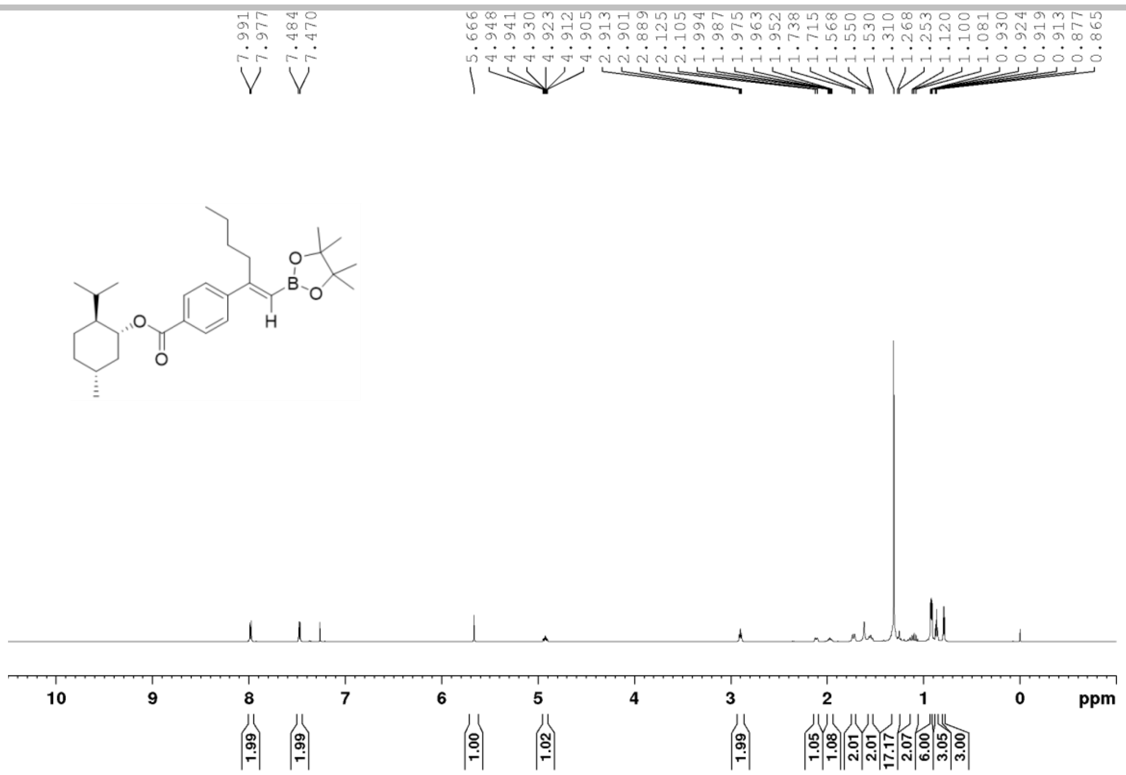
7.991
7.977
7.484
7.470
5.666
4.948
4.941
4.930
4.923
4.912
4.905
2.913
2.901
2.889
2.125
2.105
1.994
1.987
1.975
1.963
1.952
1.738
1.715
1.568
1.550
1.530
1.310
1.268
1.253
1.120
1.100
1.081
0.930
0.924
0.919
0.913
0.877
0.865
10
9
8
7
6
5
4
3
2
1
0
ppm
1.99
1.99
1.00
1.02
1.99
1.05
1.08
2.01
2.01
17.17
2.07
6.00
3.05
3.00
O
B
O
H
O
O

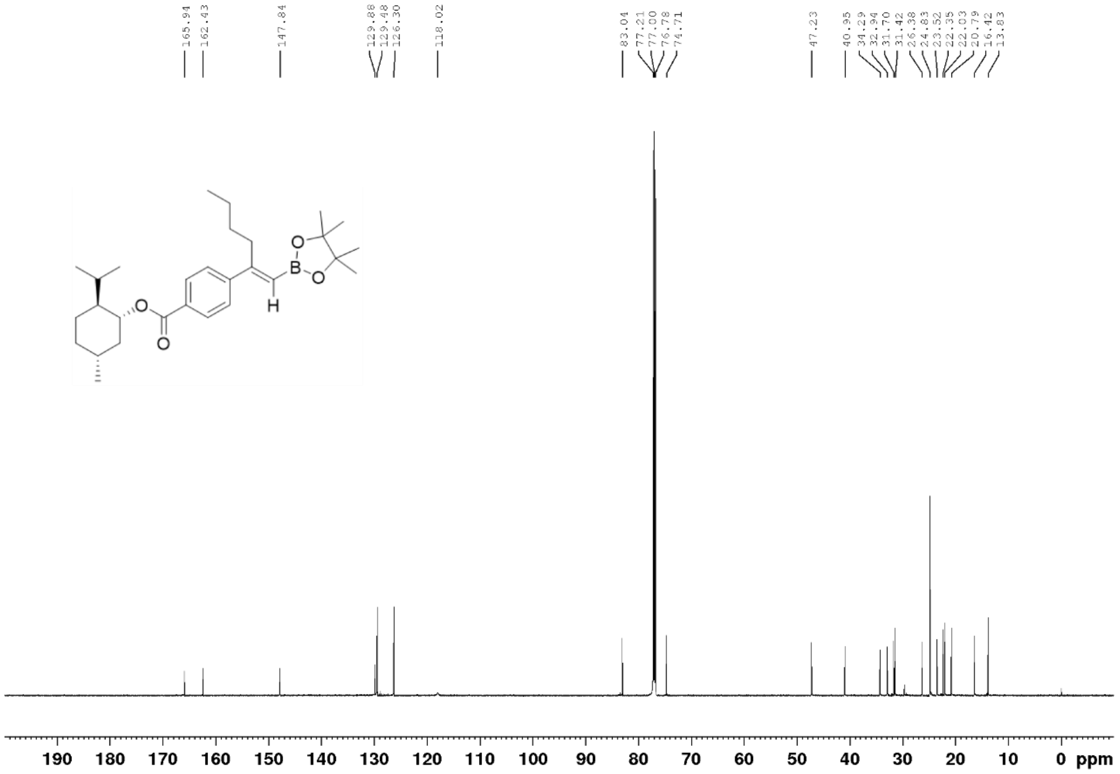
165.94
162.43
147.84
129.88
129.48
126.30
118.02
83.04
77.21
77.00
76.78
74.71
47.23
40.95
34.29
32.94
31.70
31.42
26.38
24.83
23.52
22.35
22.03
20.79
16.42
13.83
190
180
170
160
150
140
130
120
110
100
90
80
70
60
50
40
30
20
10
0
ppm
O
B
O
H
O
O

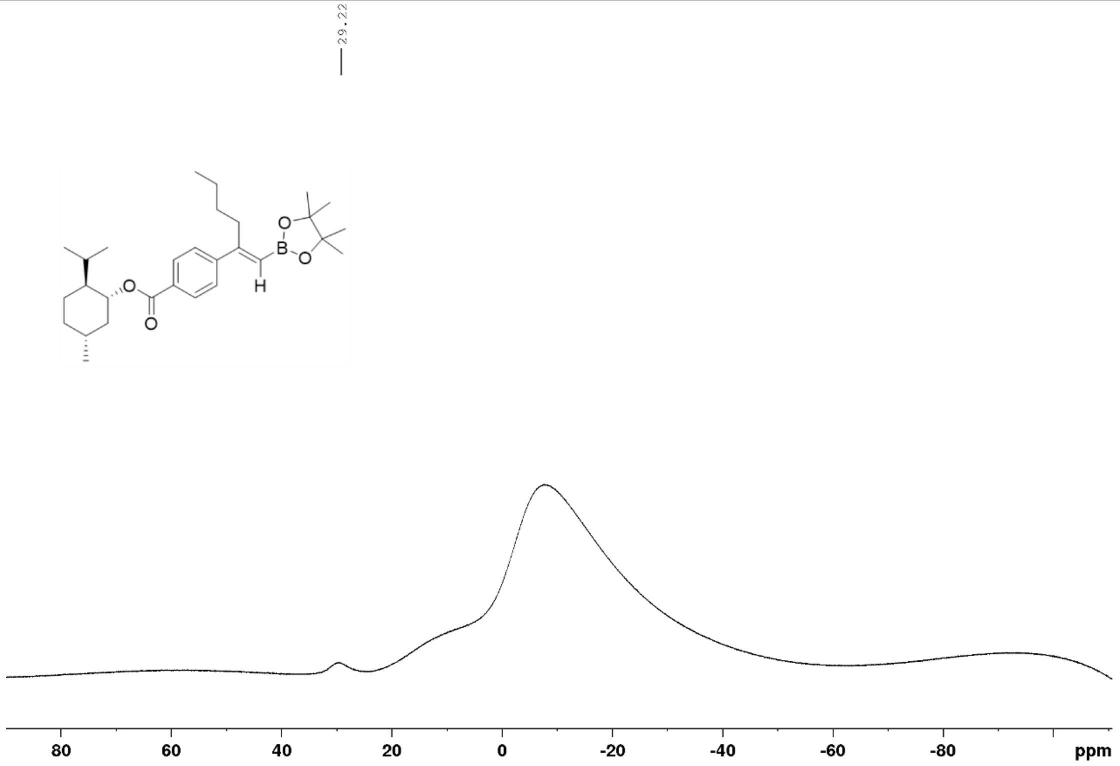
29.22
O
B
O
H
O
O
80
60
40
20
0
-20
-40
-60
-80
ppm

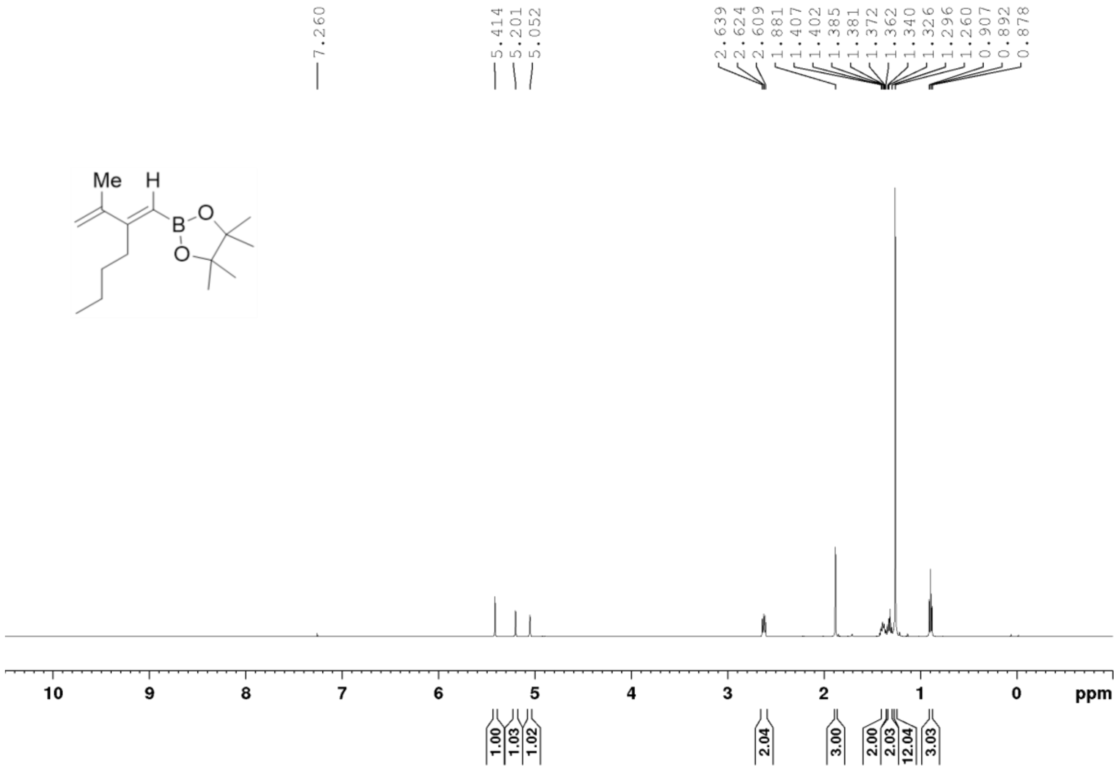
7.260
5.414
5.201
5.052
2.639
2.624
2.609
1.881
1.407
1.402
1.385
1.381
1.372
1.362
1.340
1.326
1.296
1.260
0.907
0.892
0.878
Me
H
B
O
O
10
9
8
7
6
5
4
3
2
1
0
ppm
1.00
1.03
1.02
2.04
3.00
2.00
2.03
12.04
3.03

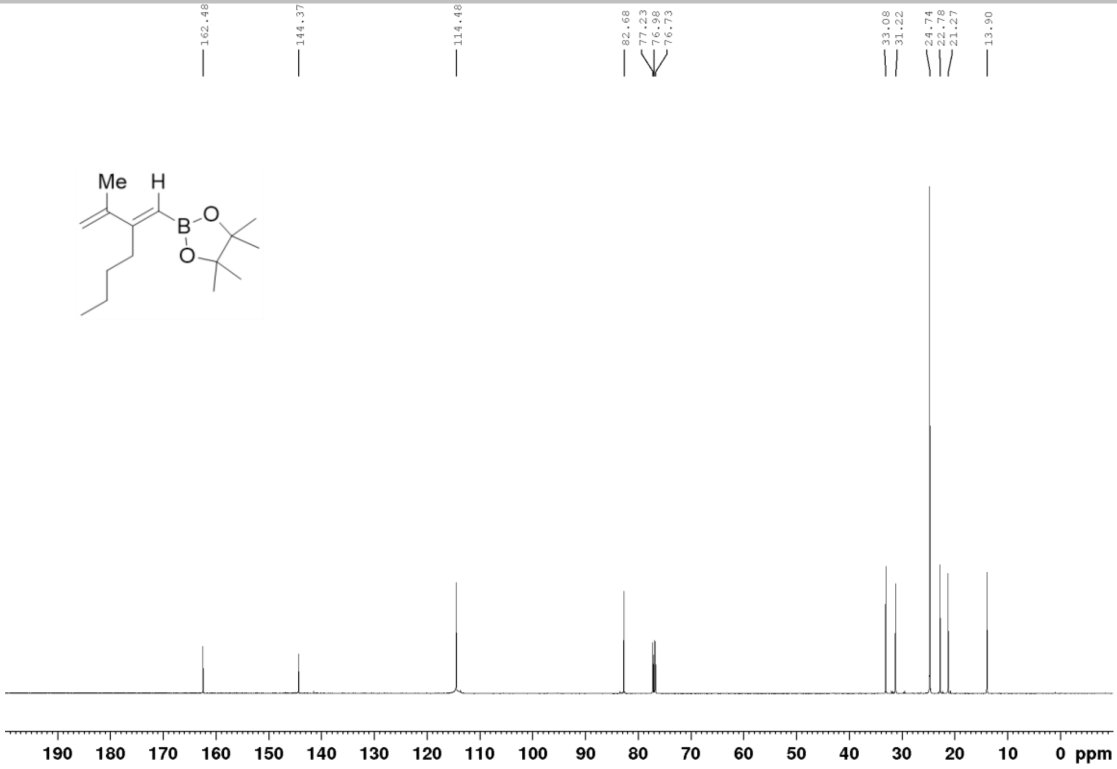
162.48
144.37
114.48
82.68
77.23
76.98
76.73
33.08
31.22
24.74
22.78
21.27
13.90
Me
H
B
O
O
190 180 170 160 150 140 130 120 110 100 90 80 70 60 50 40 30 20 10 0 ppm

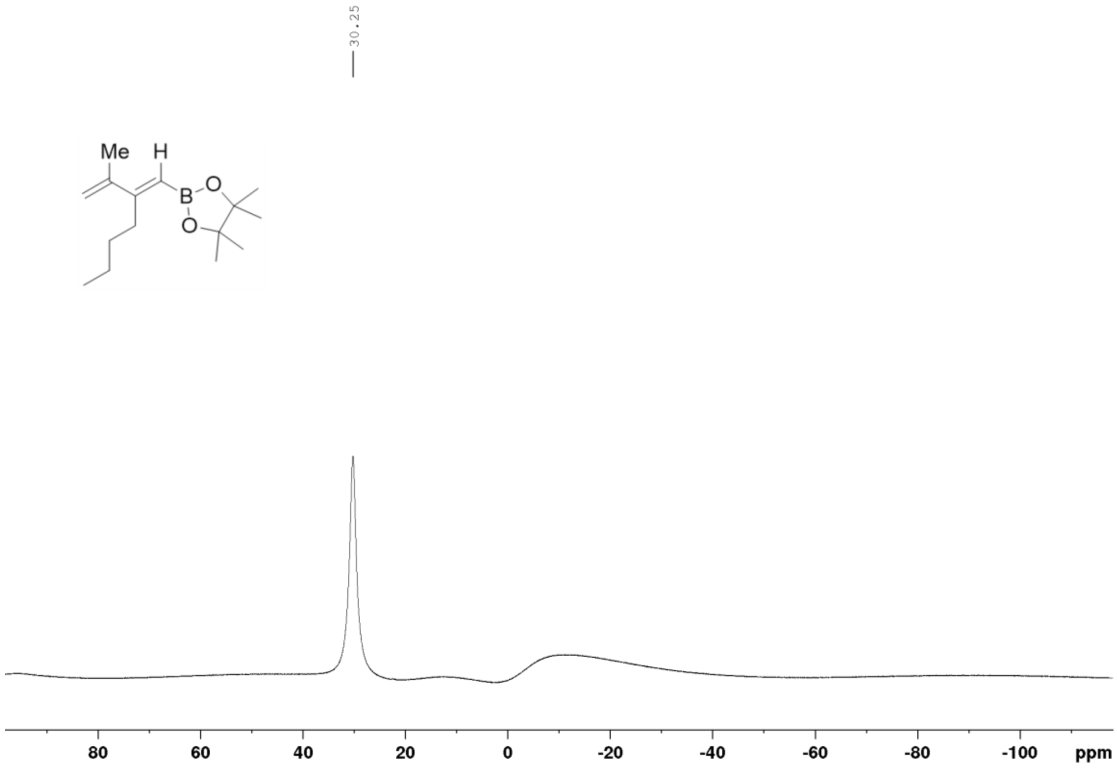
30.25
Me
H
B
O
O
80 60 40 20 0 -20 -40 -60 -80 -100 ppm

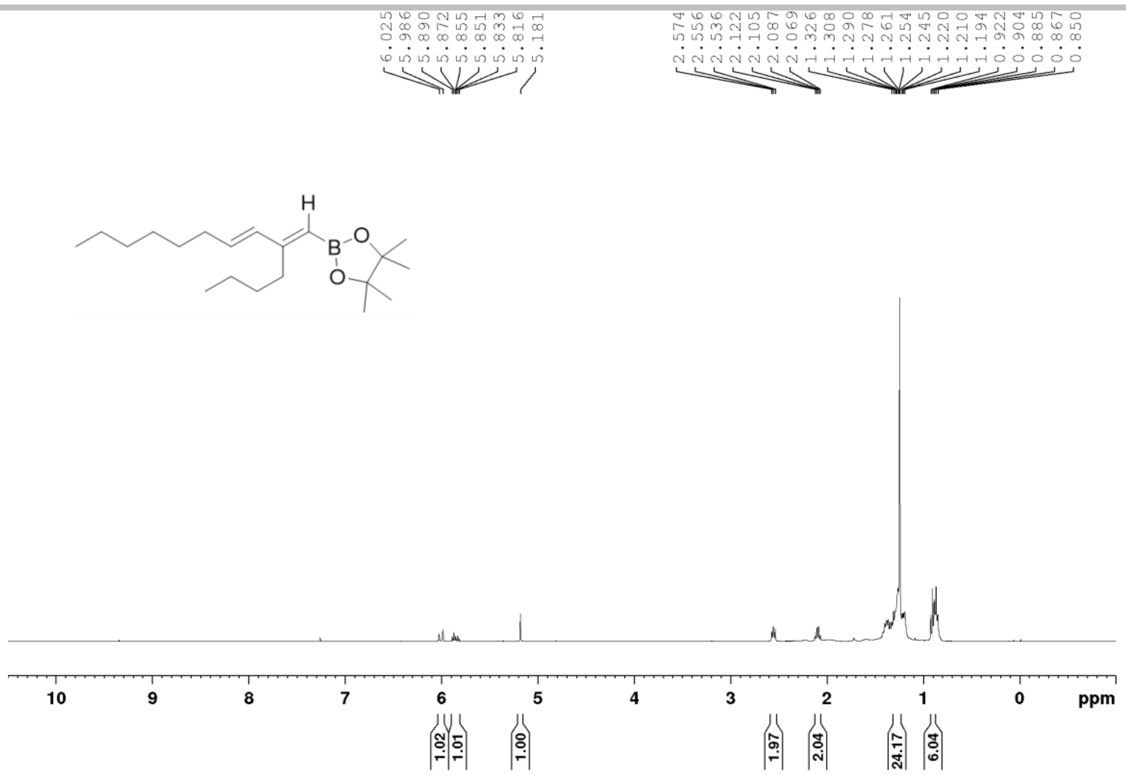
6.025
5.986
5.890
5.872
5.855
5.851
5.833
5.816
5.181
2.574
2.556
2.536
2.122
2.105
2.087
2.069
1.326
1.308
1.290
1.278
1.261
1.254
1.245
1.220
1.210
1.194
0.922
0.904
0.885
0.867
0.850
H
B
O
O
10
9
8
7
6
5
4
3
2
1
0
ppm
1.02
1.01
1.00
1.97
2.04
24.17
6.04

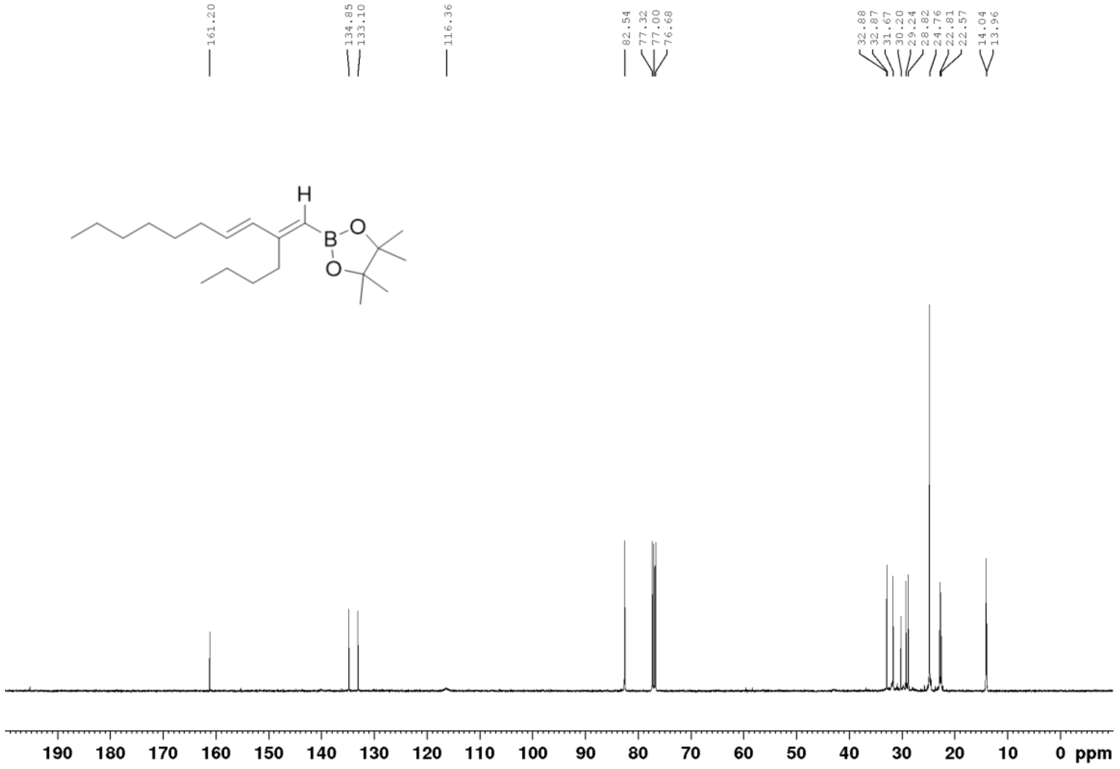
161.20
134.85
133.10
116.36
82.54
77.32
77.00
76.68
32.88
32.87
31.67
30.20
29.24
28.82
24.76
22.81
22.57
14.04
13.96
H
B
O
O
190
180
170
160
150
140
130
120
110
100
90
80
70
60
50
40
30
20
10
0 ppm

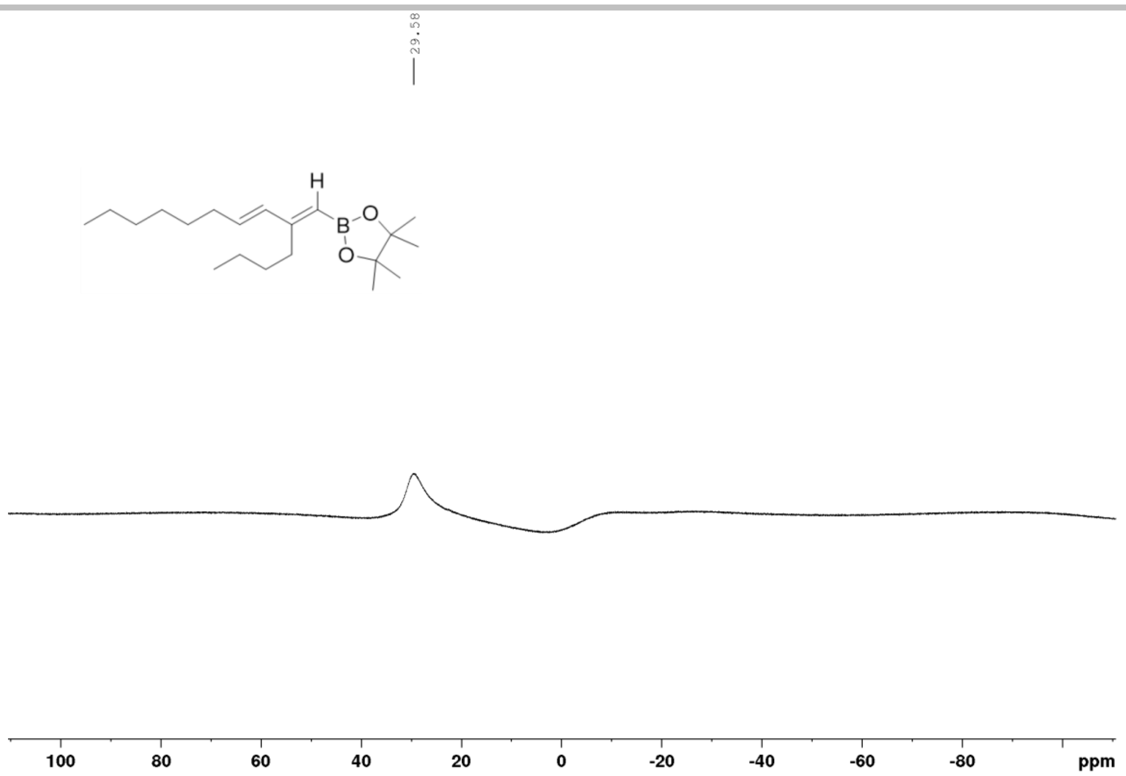
29.58
H
B
O
O
100 80 60 40 20 0 -20 -40 -60 -80 ppm

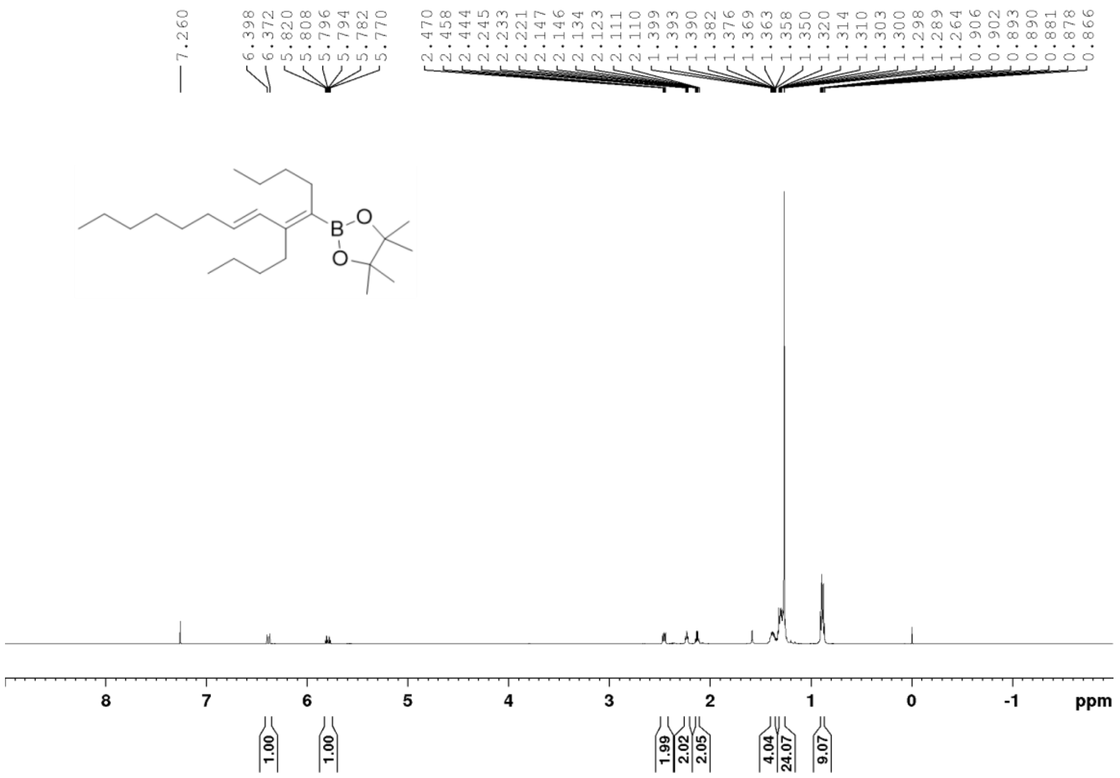
7.260
6.398 6.372 5.820 5.808 5.796 5.794 5.782 5.770
2.470 2.458 2.444 2.245 2.233 2.221 2.147 2.146 2.134 2.123 2.111 2.110 1.399 1.393 1.390 1.382 1.376 1.369 1.363 1.358 1.350 1.320 1.314 1.310 1.303 1.300 1.298 1.289 1.264 0.906 0.902 0.893 0.890 0.881 0.878 0.866
B
O
O
8 7 6 5 4 3 2 1 0 -1 ppm
1.00 1.00 1.99 2.02 2.05 4.04 24.07 9.07

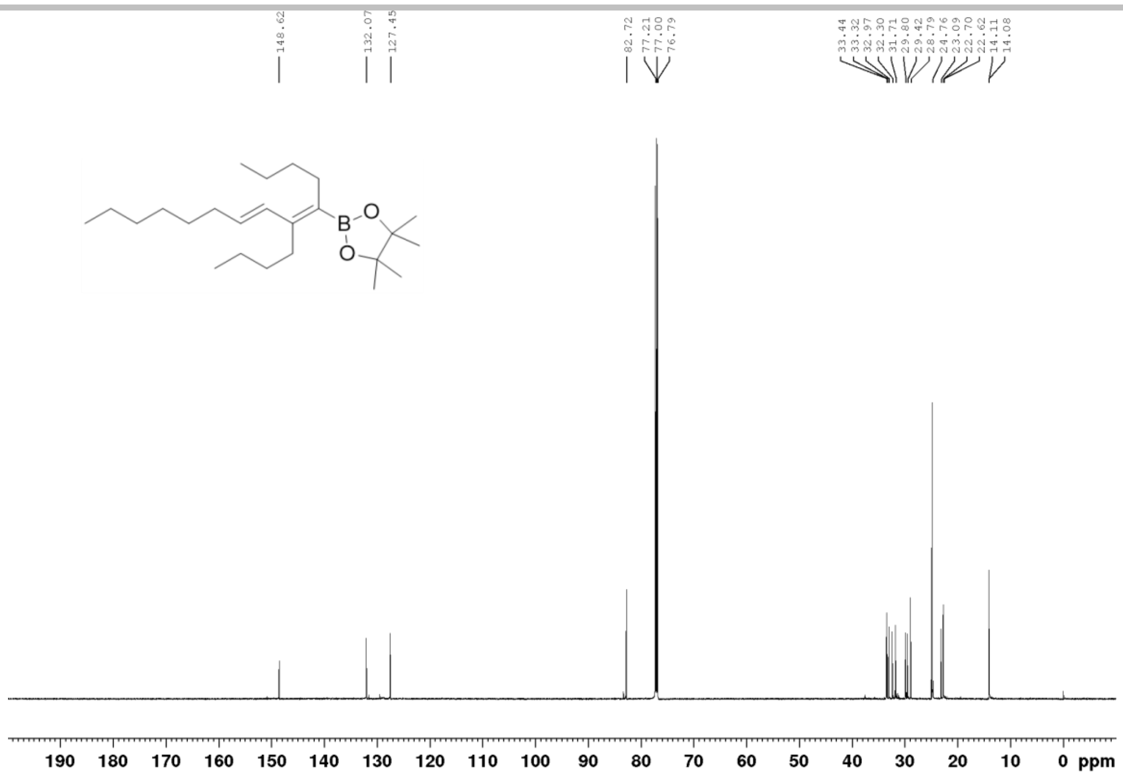
148.62
132.07
127.45
82.72
77.21
77.00
76.79
33.44
33.32
32.97
32.30
31.71
29.80
29.42
28.79
24.76
23.09
22.70
22.62
14.11
14.08
B
O
O
190
180
170
160
150
140
130
120
110
100
90
80
70
60
50
40
30
20
10
0
ppm

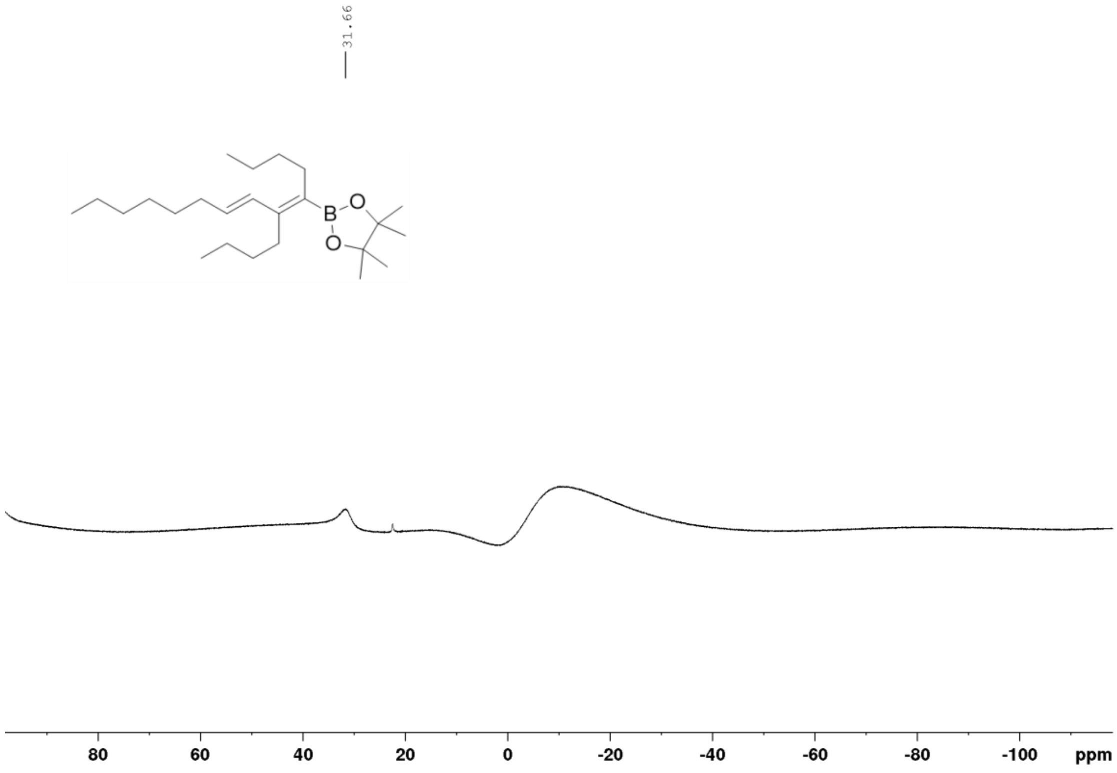
31.66
B
O
O
80
60
40
20
0
-20
-40
-60
-80
-100
ppm

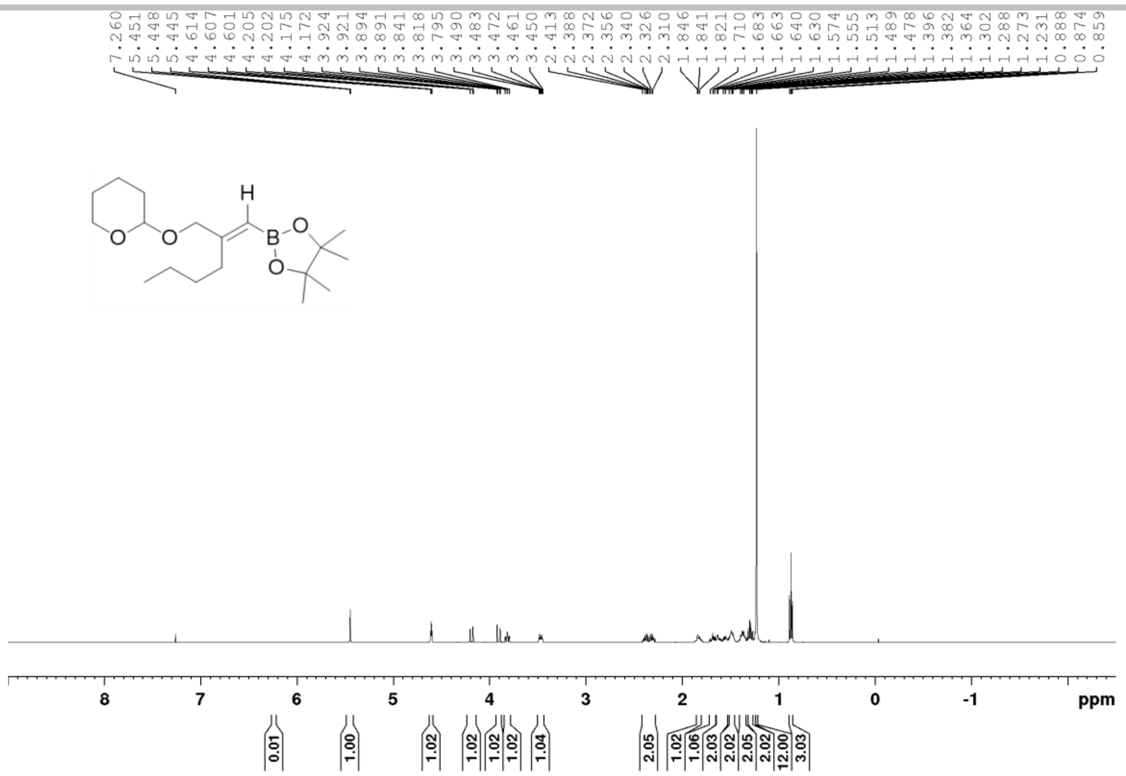

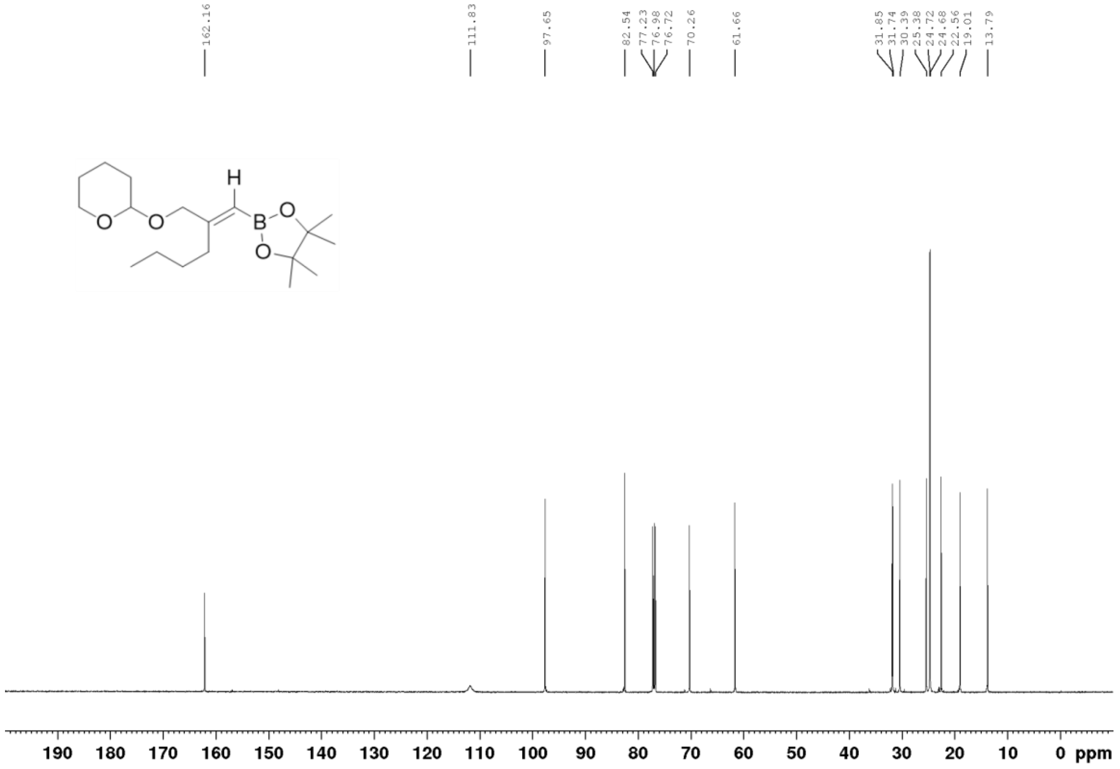

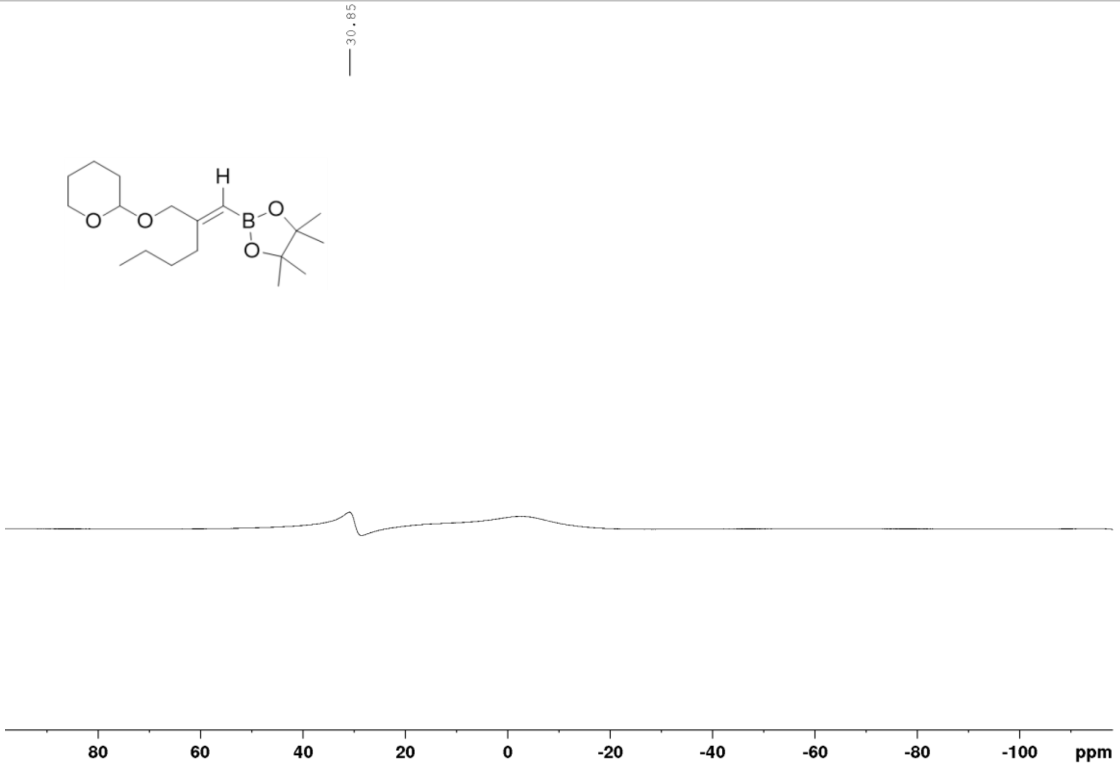
30.85
H
O
O
B
O
O
80
60
40
20
0
-20
-40
-60
-80
-100
ppm

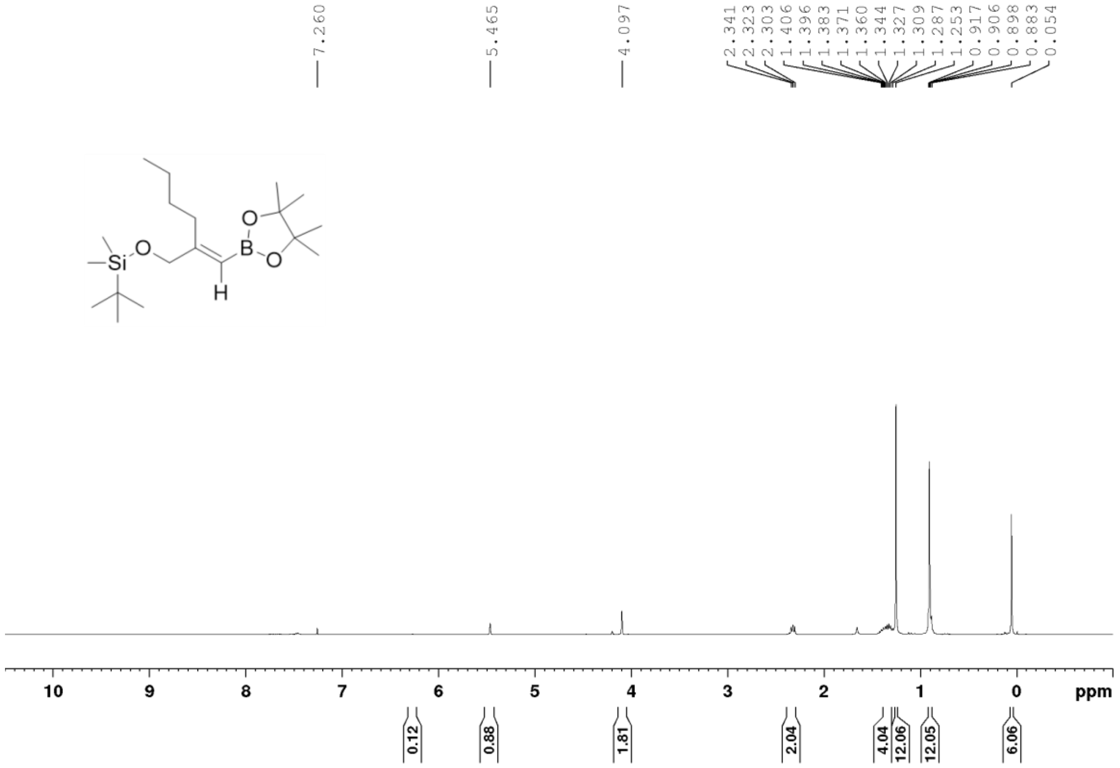
7.260
5.465
4.097
2.341
2.323
2.303
1.406
1.396
1.383
1.371
1.360
1.344
1.327
1.309
1.287
1.253
0.917
0.906
0.898
0.883
0.054
Si
O
O
B
O
H
10
9
8
7
6
5
4
3
2
1
0
ppm
0.12
0.88
1.81
2.04
4.04
12.06
12.05
6.06

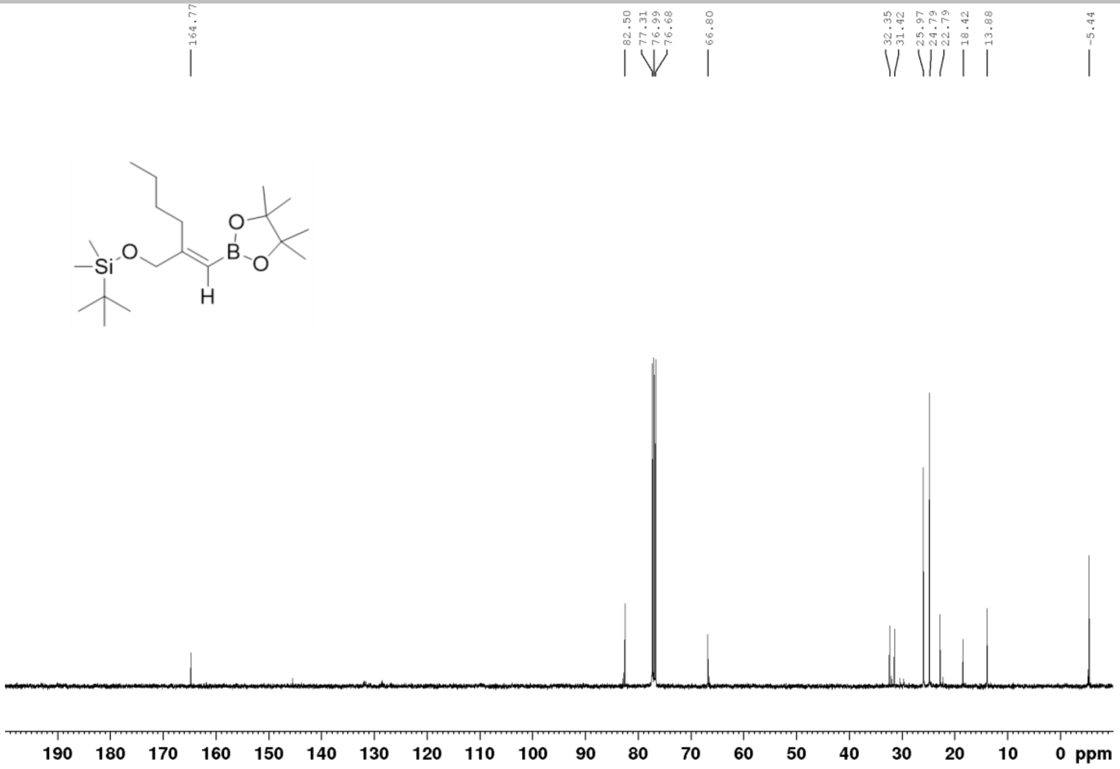
164.77
82.50
77.31
76.99
76.68
66.80
32.35
31.42
25.97
24.79
22.79
18.42
13.88
-5.44
ppm

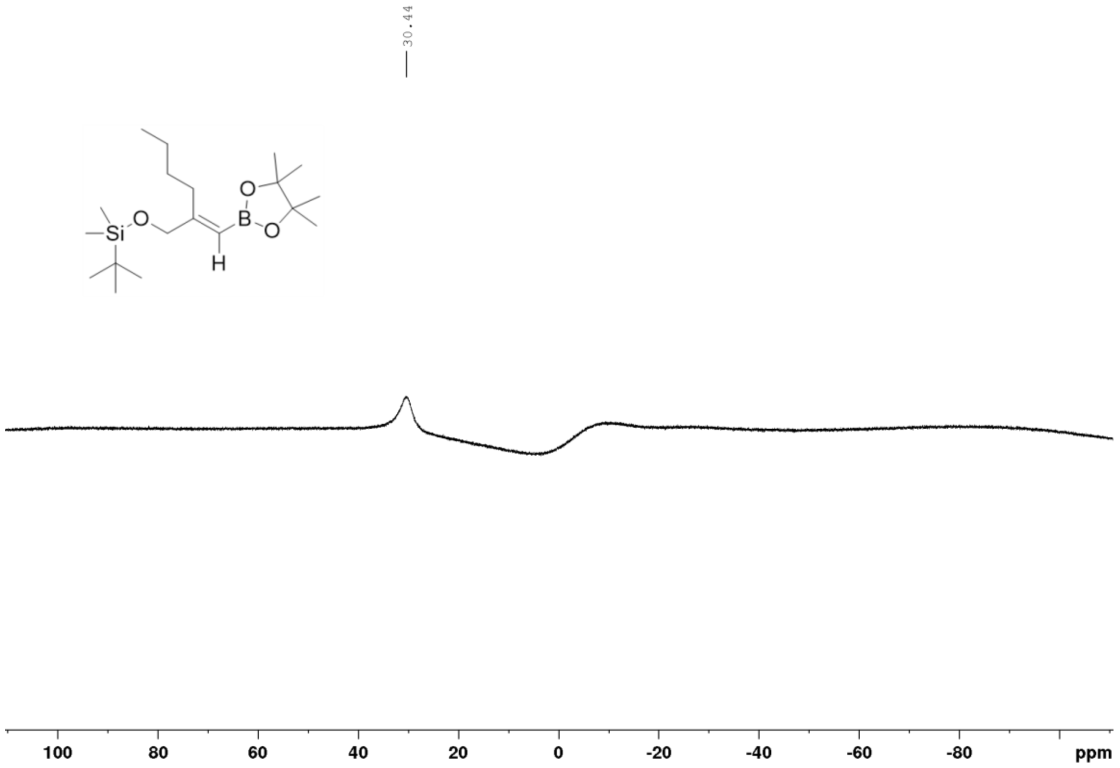
30.44
ppm

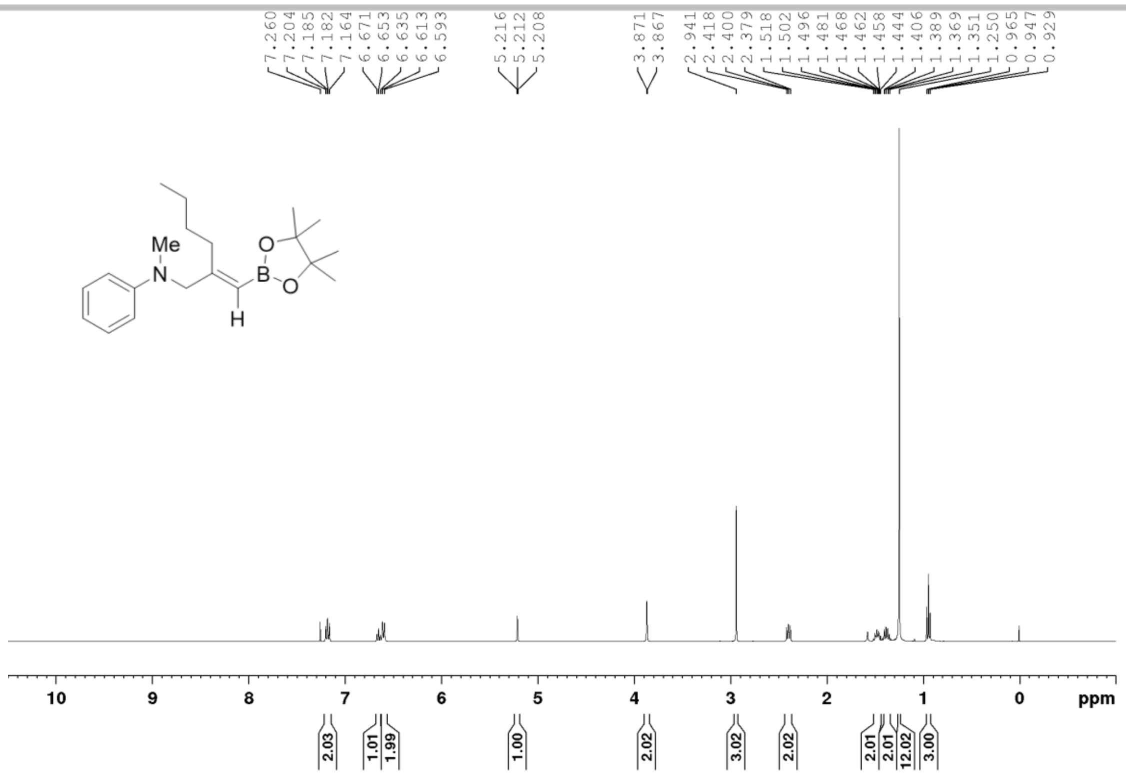
7.260
7.204
7.185
7.182
7.164
6.671
6.653
6.635
6.613
6.593
5.216
5.212
5.208
3.871
3.867
2.941
2.418
2.400
2.379
1.518
1.502
1.496
1.481
1.468
1.462
1.458
1.444
1.406
1.389
1.369
1.351
1.250
0.965
0.947
0.929
Me
N
O
B
O
H
10 9 8 7 6 5 4 3 2 1 0 ppm
2.03
1.01
1.99
1.00
2.02
3.02
2.02
2.01
2.01
12.02
3.00

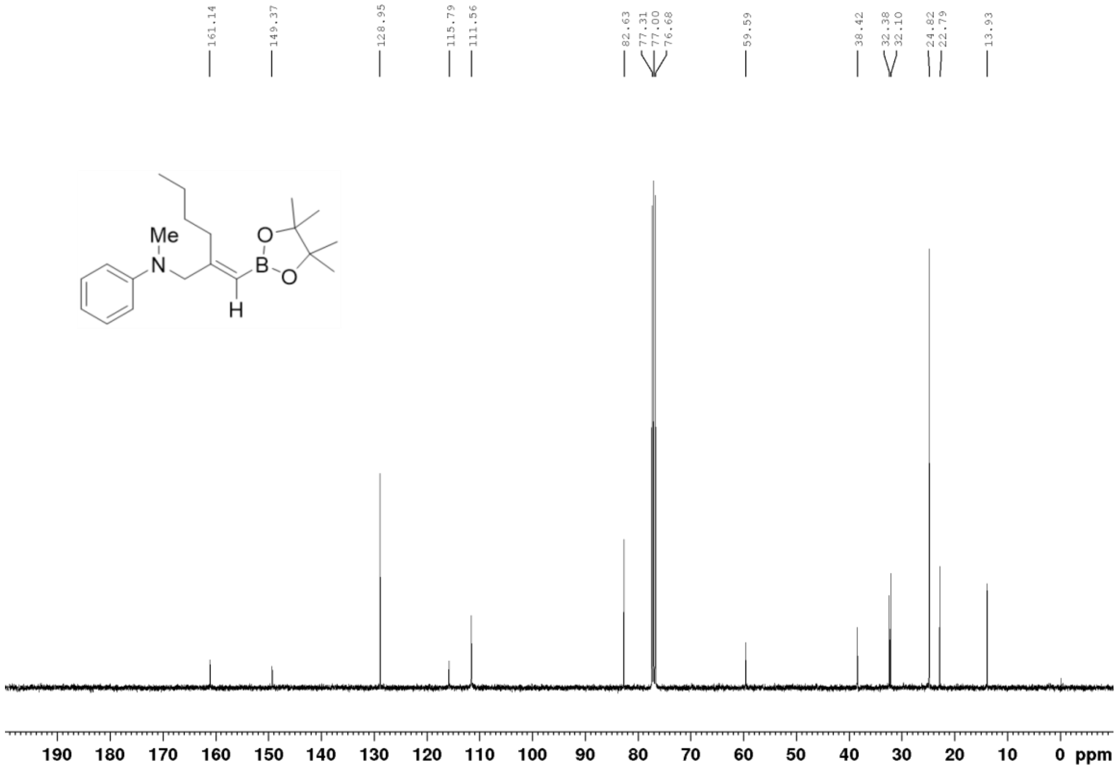
161.14
149.37
128.95
115.79
111.56
82.63
77.31
77.00
76.68
59.59
38.42
32.38
32.10
24.82
22.79
13.93
Me
N
O
B
O
H
190 180 170 160 150 140 130 120 110 100 90 80 70 60 50 40 30 20 10 0 ppm

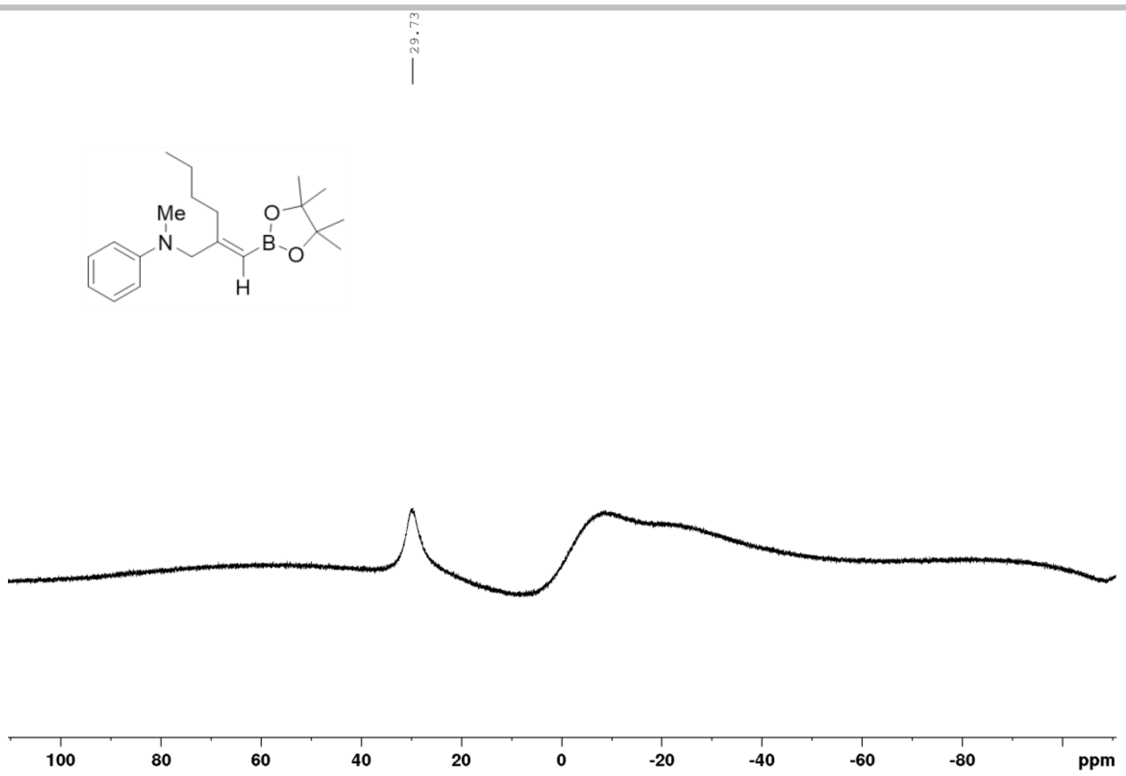
29.73
Me
N
O
B
O
H
100
80
60
40
20
0
-20
-40
-60
-80
ppm

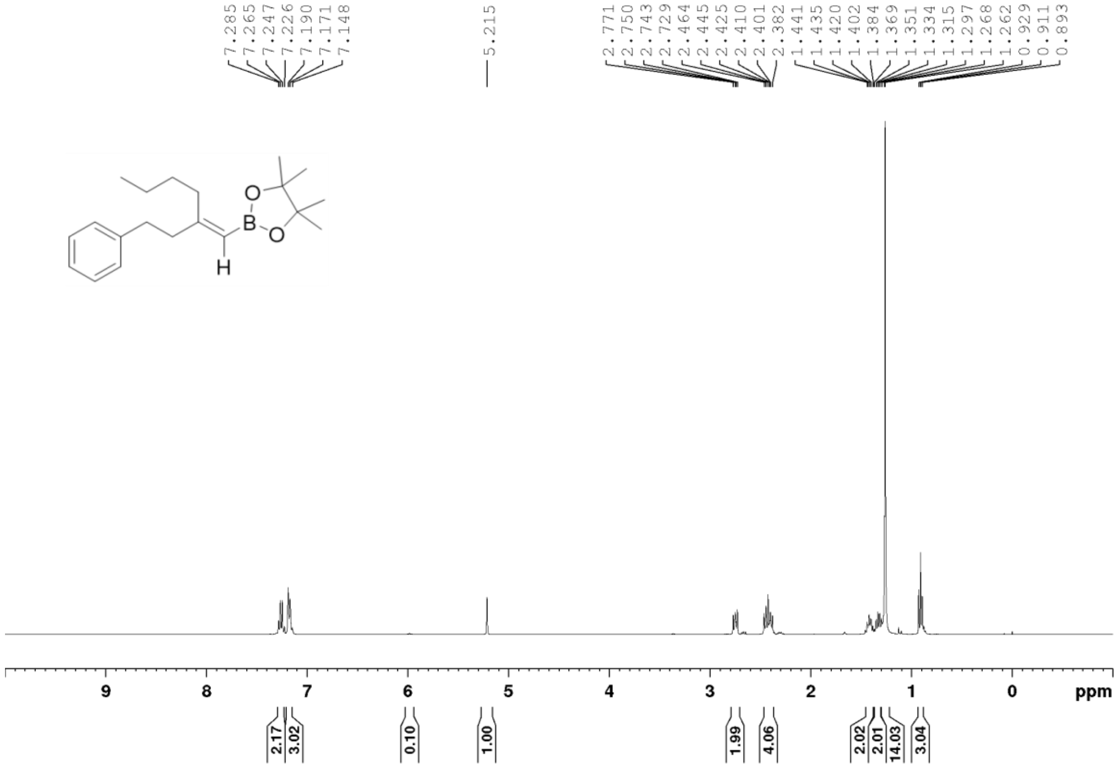
7.285
7.265
7.247
7.226
7.190
7.171
7.148
5.215
2.771
2.750
2.743
2.729
2.464
2.445
2.425
2.410
2.401
2.382
1.441
1.435
1.420
1.402
1.384
1.369
1.351
1.334
1.315
1.297
1.268
1.262
0.929
0.911
0.893
O
B
O
H
9
8
7
6
5
4
3
2
1
0
ppm
2.17
3.02
0.10
1.00
1.99
4.06
2.02
2.01
14.03
3.04

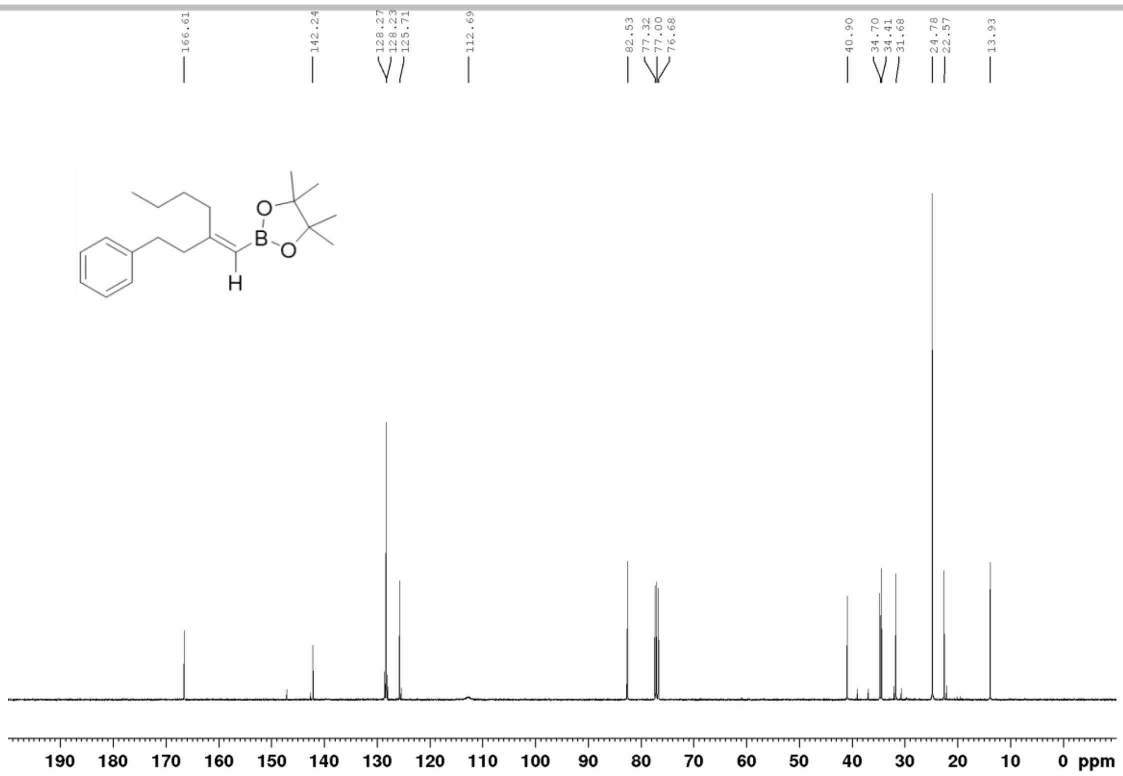
166.61
142.24
128.27
128.23
125.71
112.69
82.53
77.32
77.00
76.68
40.90
34.70
34.41
31.68
24.78
22.57
13.93
B
O
O
H
190 180 170 160 150 140 130 120 110 100 90 80 70 60 50 40 30 20 10 0 ppm

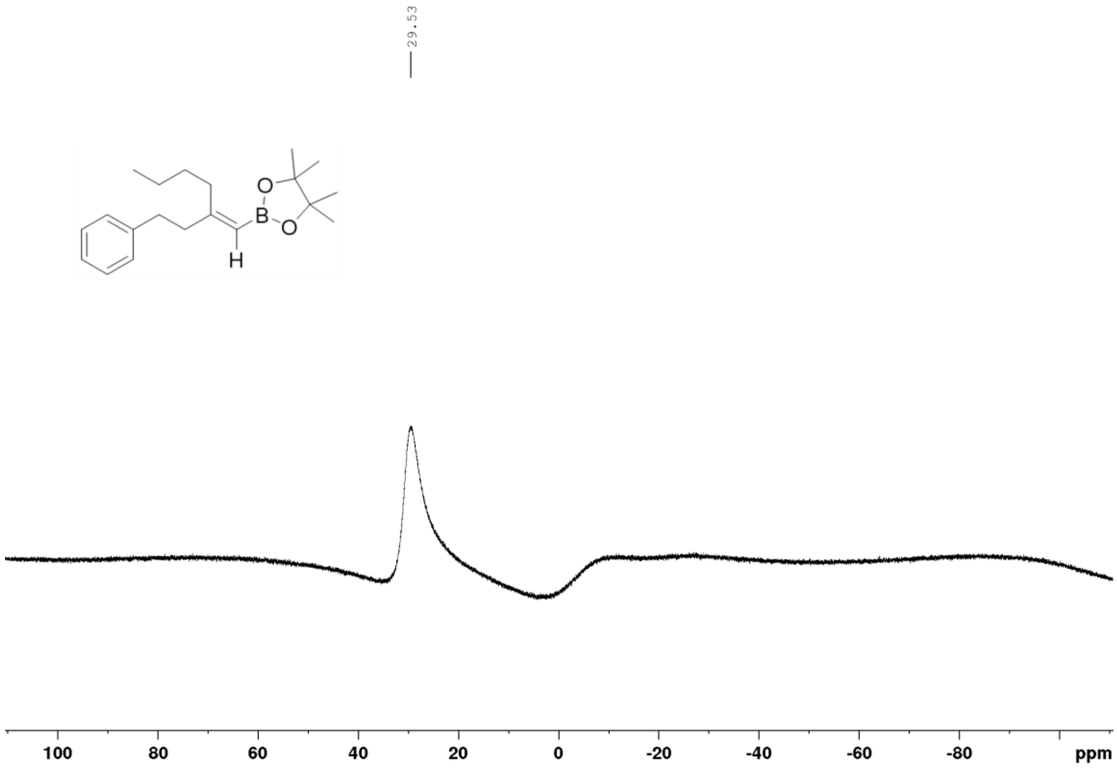
29.53
B
O
O
H
100 80 60 40 20 0 -20 -40 -60 -80 ppm

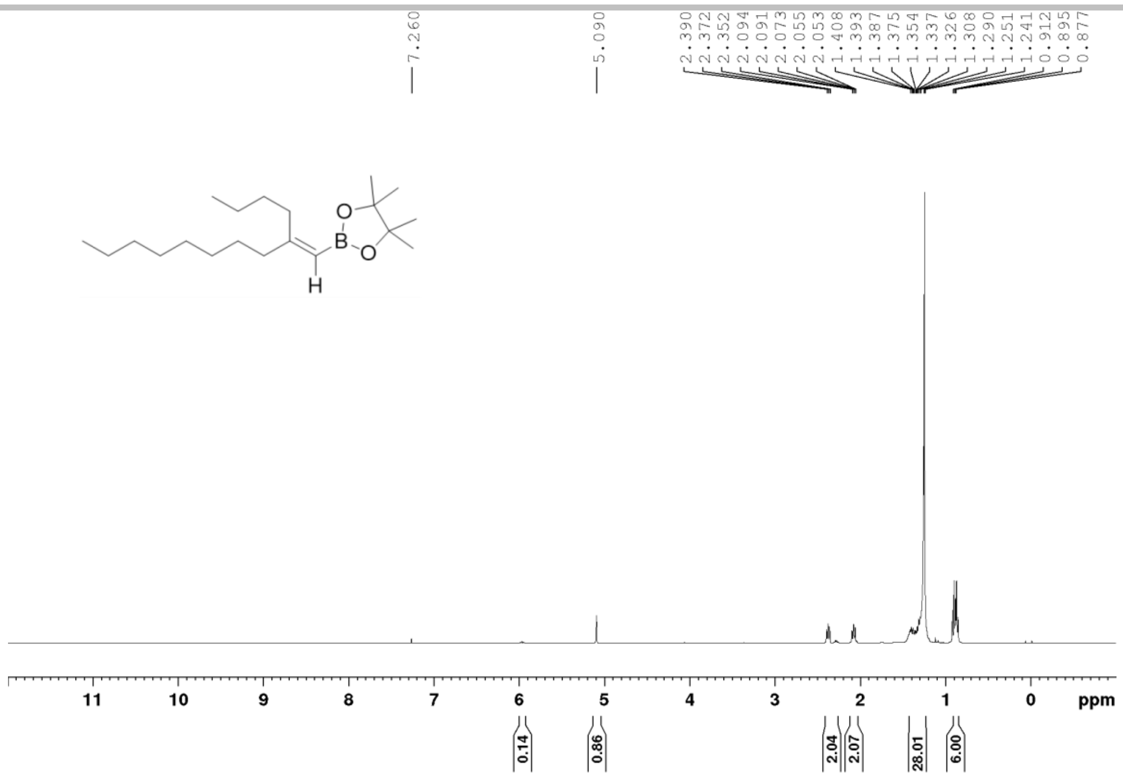
7.260
5.090
2.390 2.372 2.352 2.094 2.091 2.073 2.055 2.053 1.408 1.393 1.387 1.375 1.354 1.337 1.326 1.308 1.290 1.251 1.241 0.912 0.895 0.877
O
B
O
H
11 10 9 8 7 6 5 4 3 2 1 0 ppm
0.14
0.86
2.04
2.07
28.01
6.00

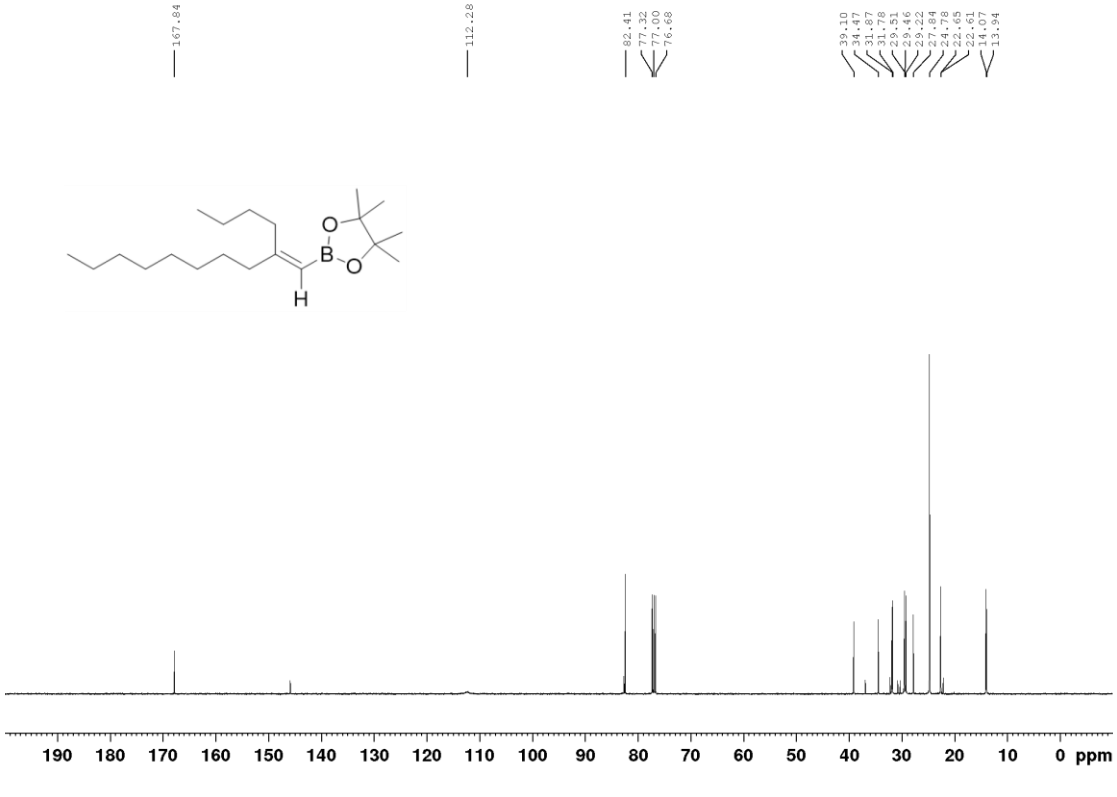
167.84
112.28
82.41 77.32 77.00 76.68
39.10 34.47 31.87 31.78 29.51 29.46 29.22 27.84 24.78 22.65 22.61 14.07 13.94
O
B
O
H
190 180 170 160 150 140 130 120 110 100 90 80 70 60 50 40 30 20 10 0 ppm

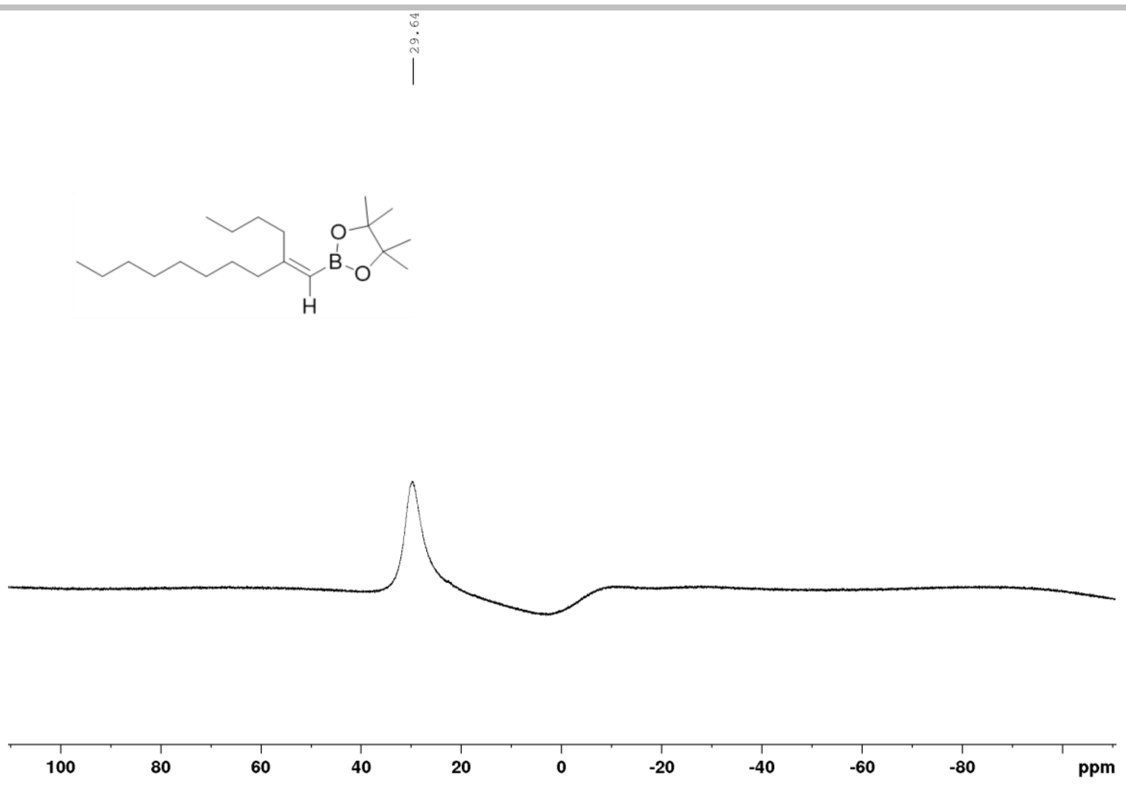
29.64
O
B
O
H
100
80
60
40
20
0
-20
-40
-60
-80
ppm

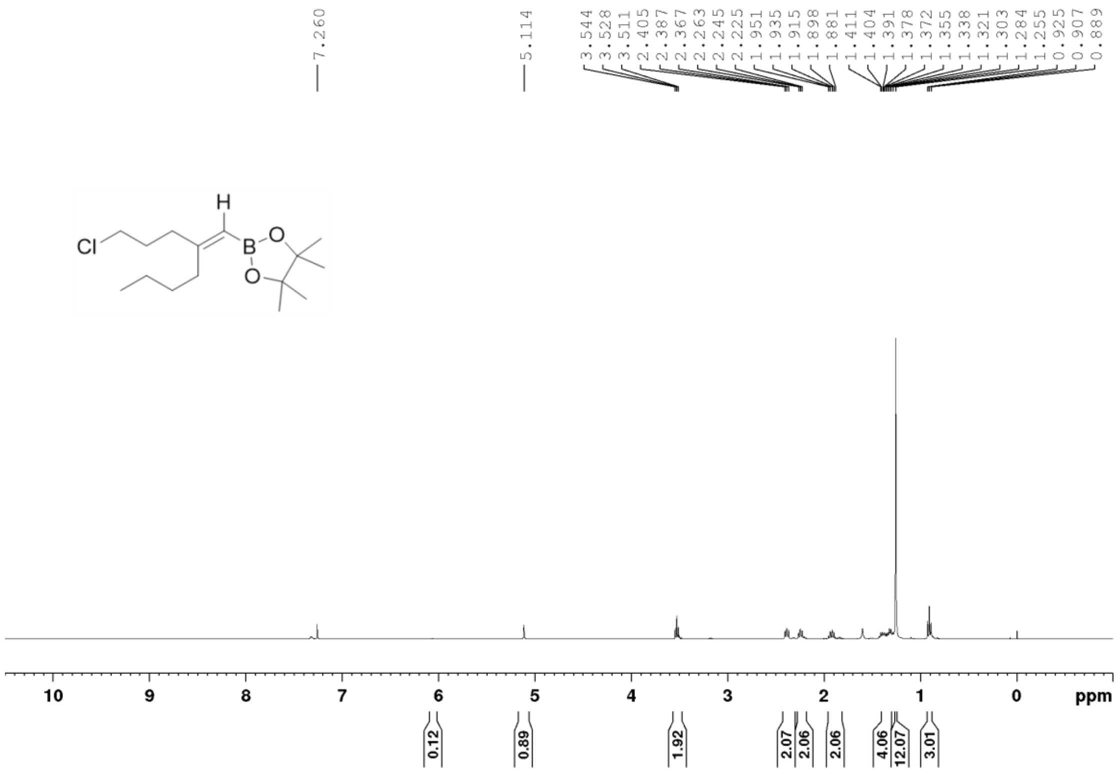
7.260
5.114
3.544
3.528
3.511
2.405
2.387
2.367
2.263
2.245
2.225
1.951
1.935
1.915
1.898
1.881
1.411
1.404
1.391
1.378
1.372
1.355
1.338
1.321
1.303
1.284
1.255
0.925
0.907
0.889
H
Cl
B
O
O
10
9
8
7
6
5
4
3
2
1
0
ppm
0.12
0.89
1.92
2.07
2.06
2.06
4.06
12.07
3.01

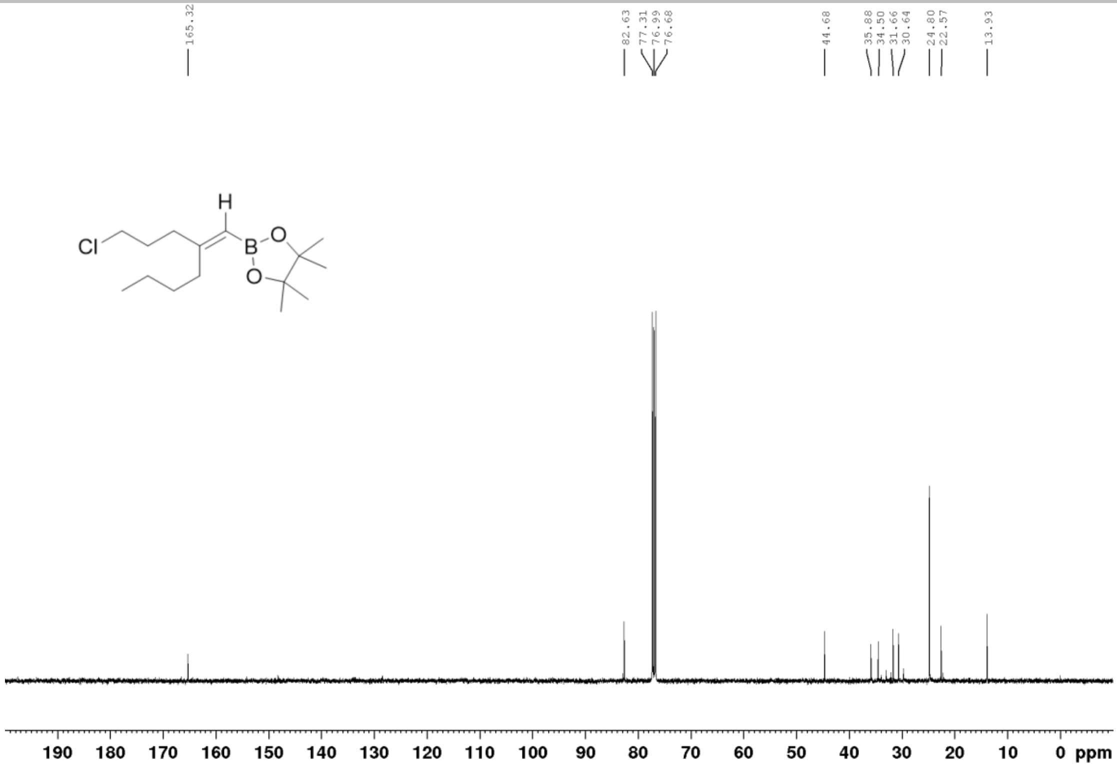
165.32
82.63
77.31
76.99
76.68
44.68
35.88
34.50
31.66
30.64
24.80
22.57
13.93
Cl
H
B
O
O
190 180 170 160 150 140 130 120 110 100 90 80 70 60 50 40 30 20 10 0 ppm

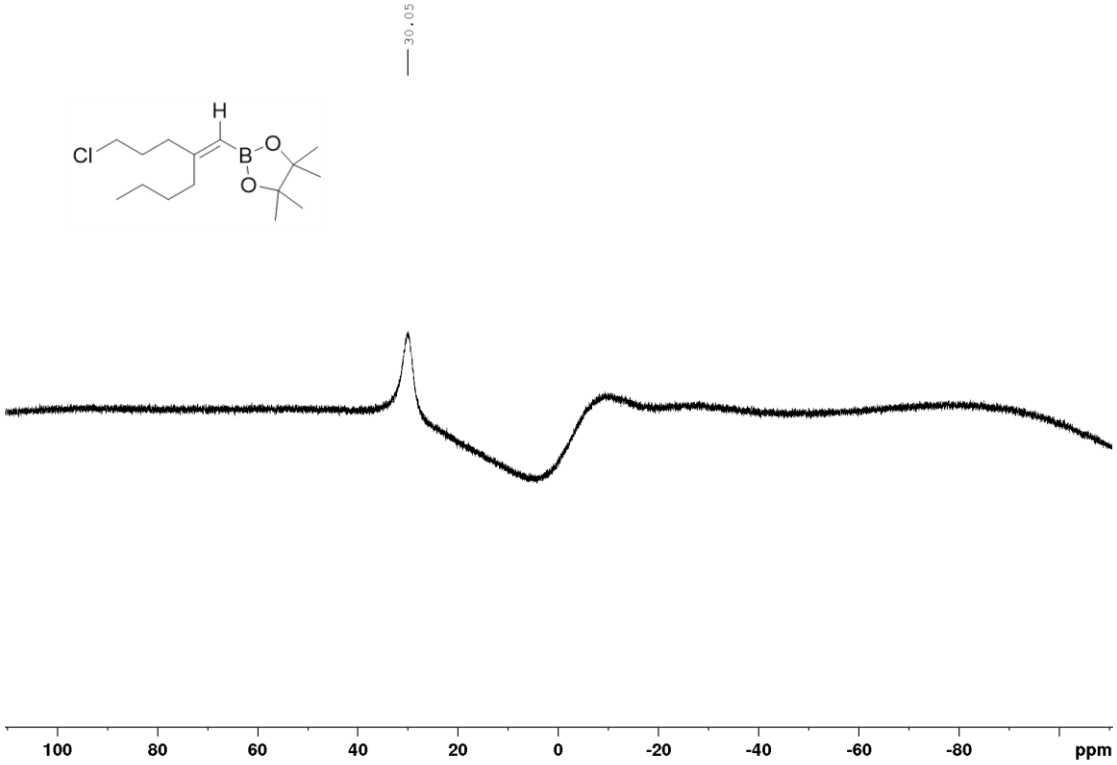
30.05
Cl
H
B
O
O
100 80 60 40 20 0 -20 -40 -60 -80 ppm

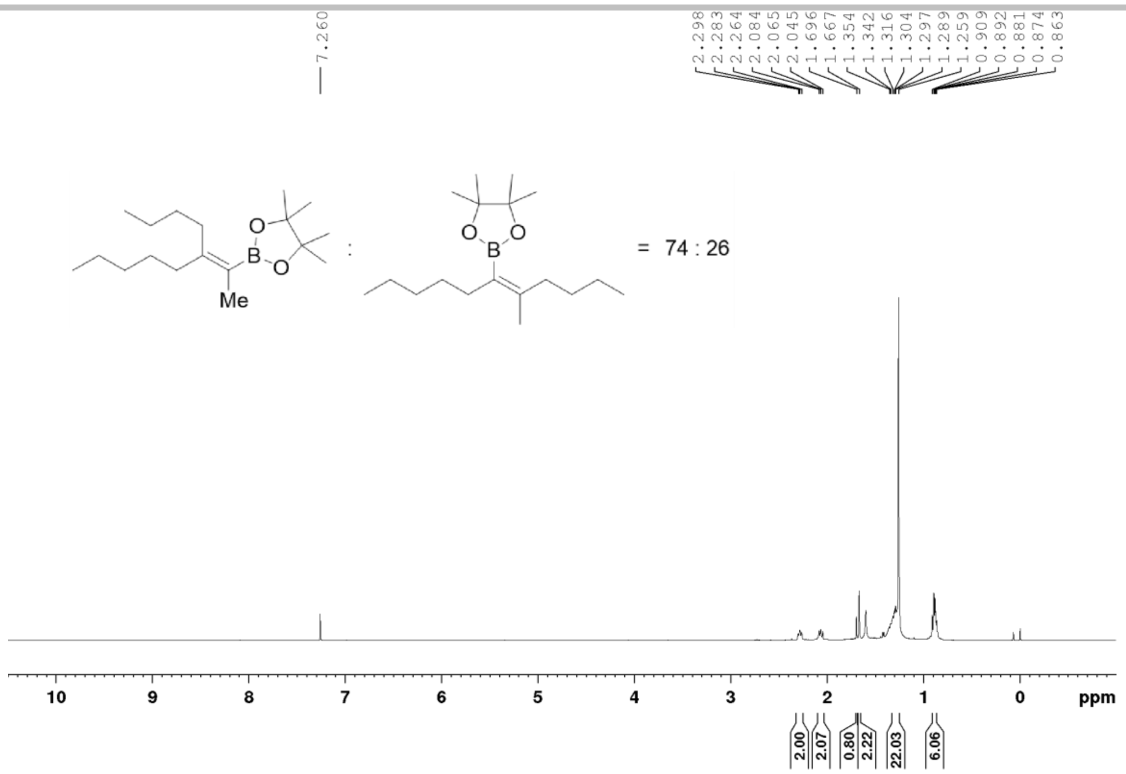
7.260
2.298
2.283
2.264
2.084
2.065
2.045
1.696
1.667
1.354
1.342
1.316
1.304
1.297
1.289
1.259
0.909
0.892
0.881
0.874
0.863
Me
= 74 : 26
10 9 8 7 6 5 4 3 2 1 0 ppm
2.00
2.07
0.80
2.22
22.03
6.06

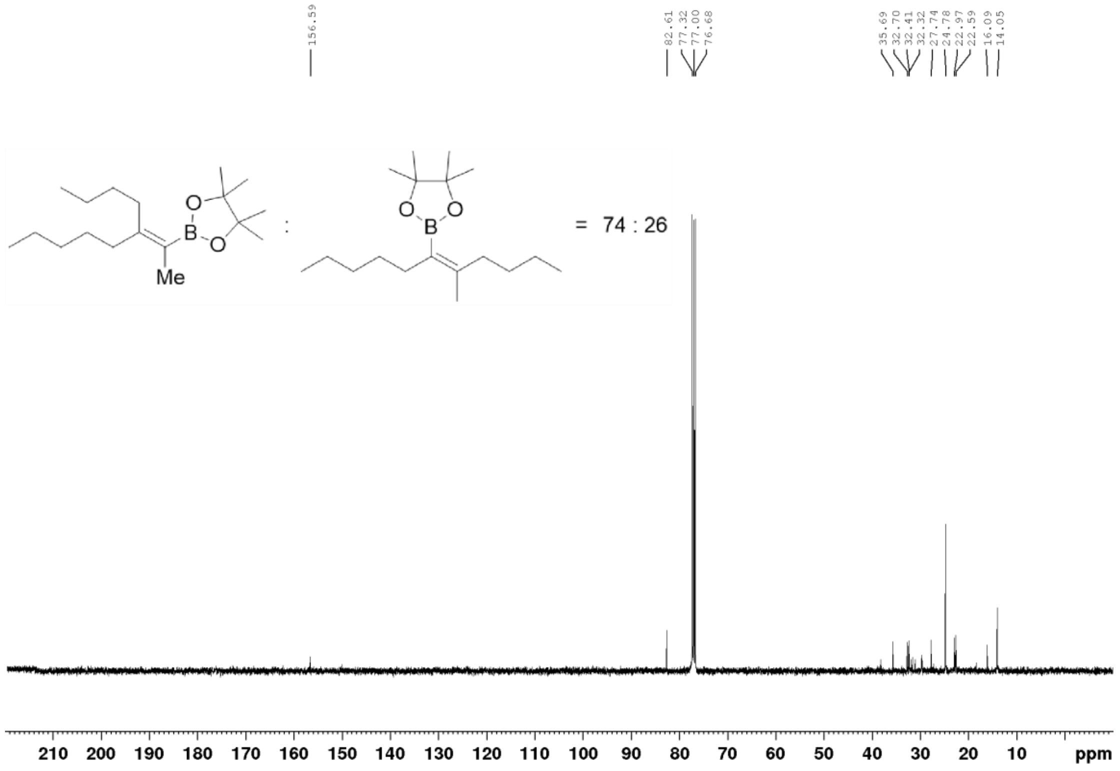
156.59
82.61
77.32
77.00
76.68
35.69
32.70
32.41
32.32
27.74
24.78
22.97
22.59
16.09
14.05
Me
= 74 : 26
210 200 190 180 170 160 150 140 130 120 110 100 90 80 70 60 50 40 30 20 10 ppm

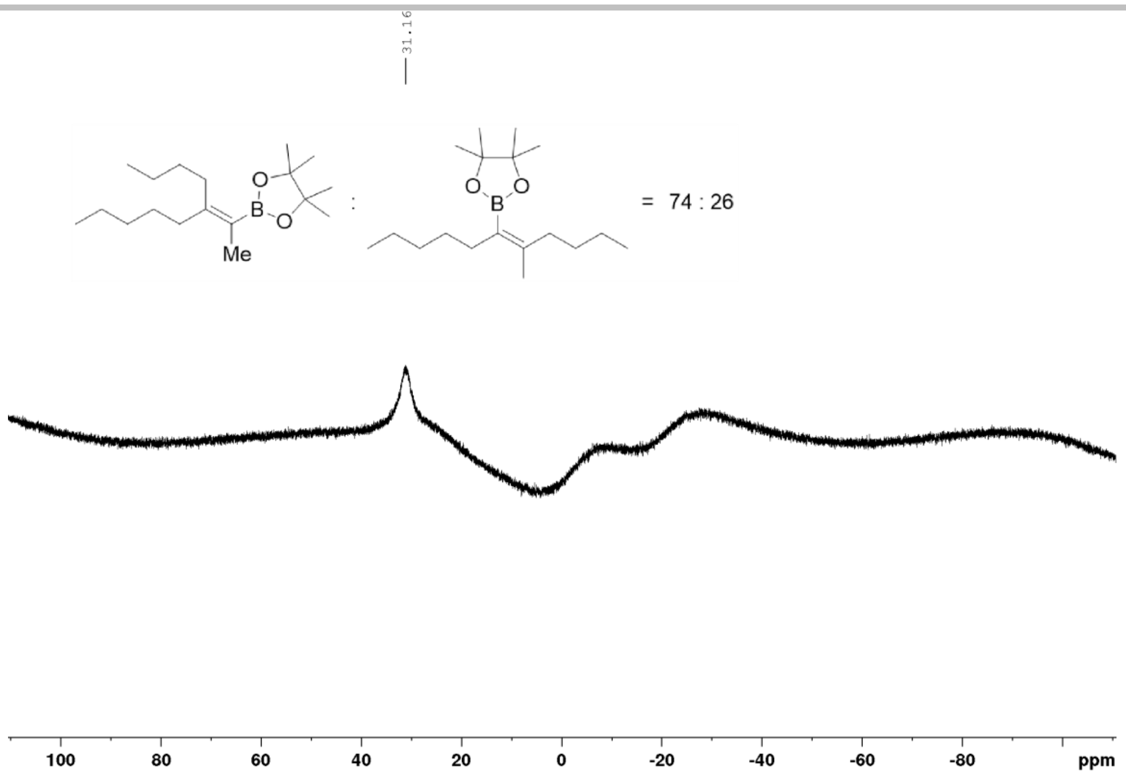
31.16
Me
= 74 : 26
100
80
60
40
20
0
-20
-40
-60
-80
ppm

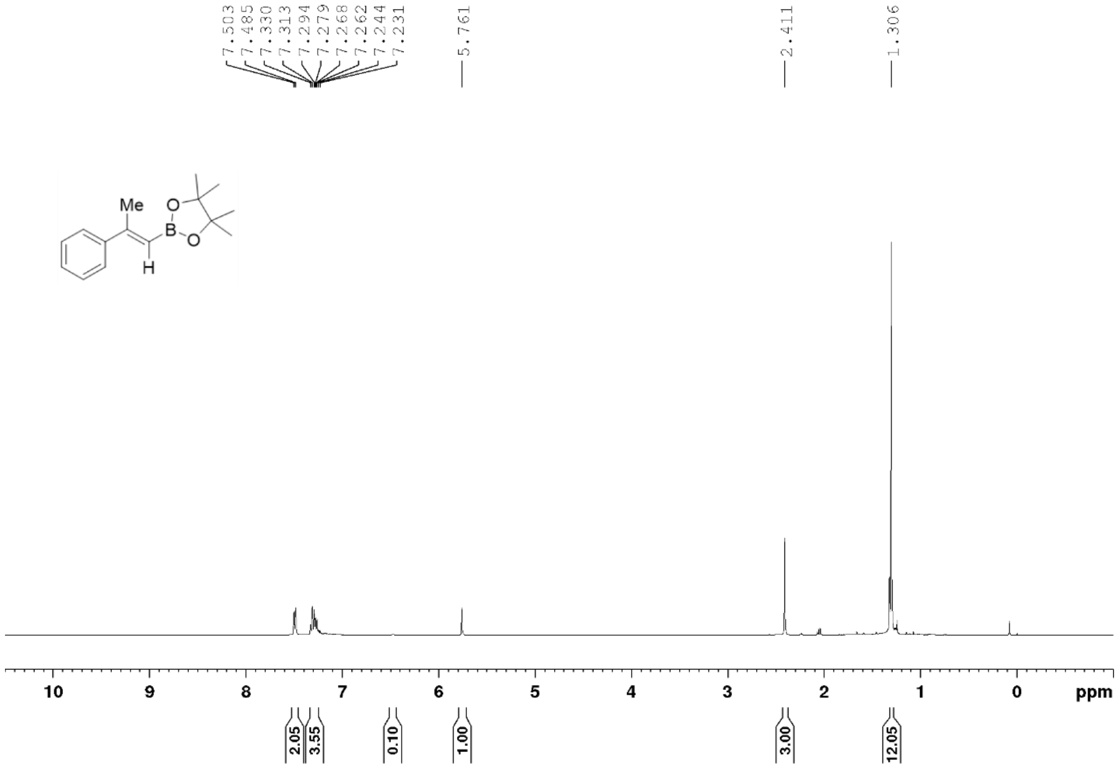
7.503
7.485
7.330
7.313
7.294
7.279
7.268
7.262
7.244
7.231
5.761
2.411
1.306
Me
H
10
9
8
7
6
5
4
3
2
1
0
ppm
2.05
3.55
0.10
1.00
3.00
12.05

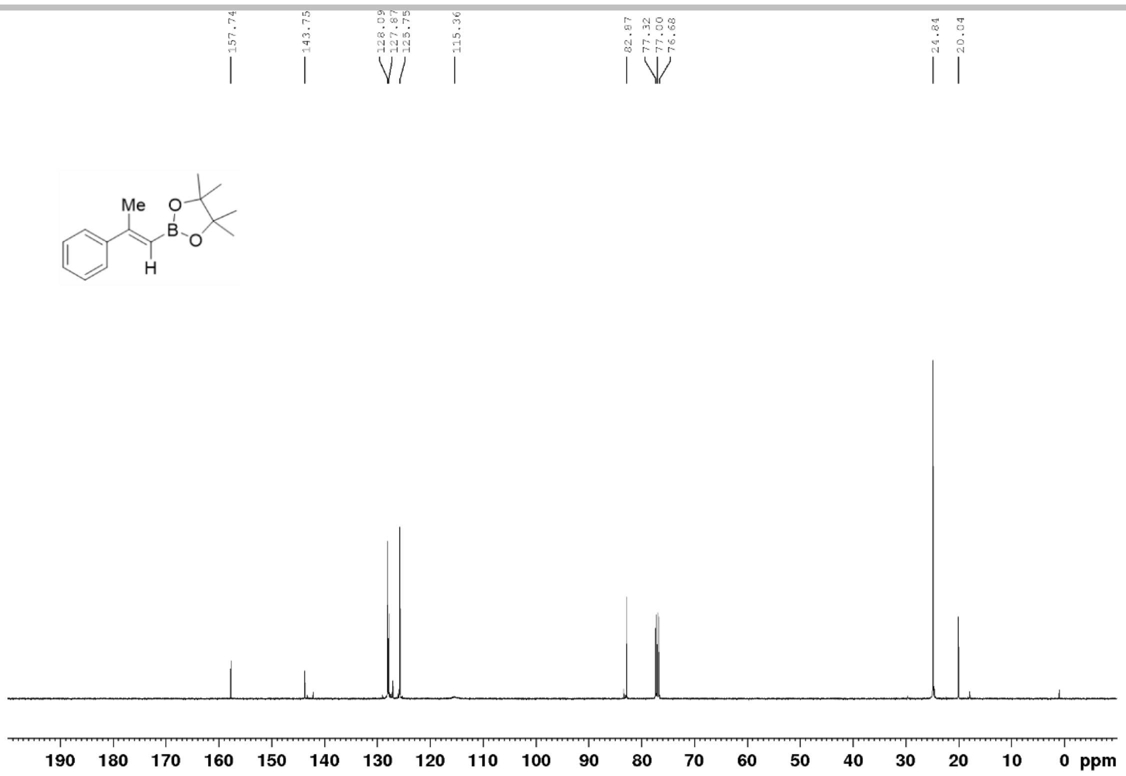
157.74
143.75
128.09
127.87
125.75
115.36
82.87
77.32
77.00
76.68
24.84
20.04
Me
O
B
O
H
190 180 170 160 150 140 130 120 110 100 90 80 70 60 50 40 30 20 10 0 ppm

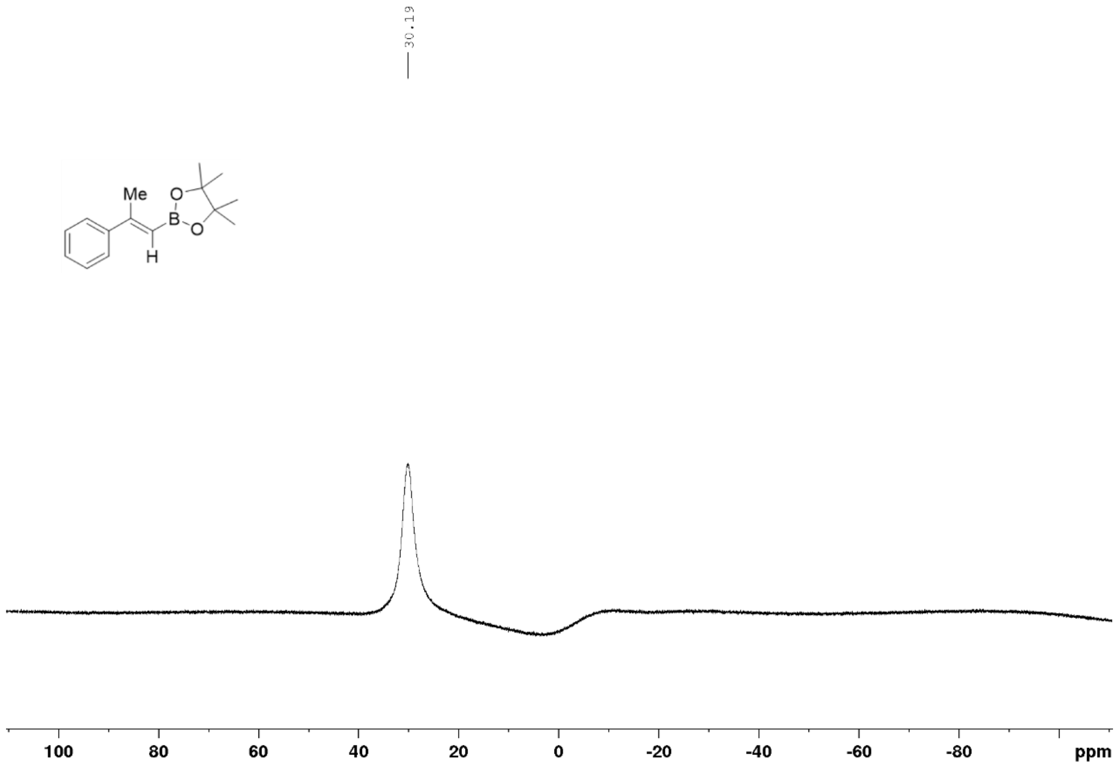
30.19
Me
O
B
O
H
100 80 60 40 20 0 -20 -40 -60 -80 ppm

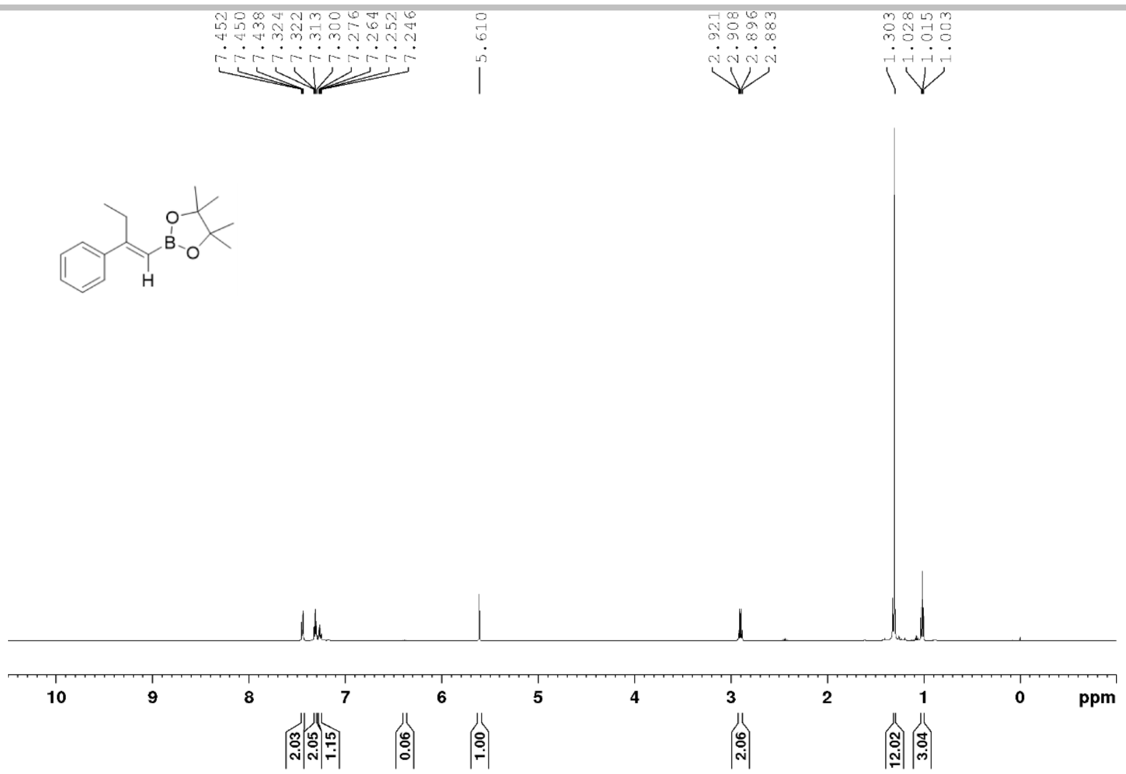
7.452
7.450
7.438
7.324
7.322
7.313
7.300
7.276
7.264
7.252
7.246
5.610
2.921
2.908
2.896
2.883
1.303
1.028
1.015
1.003
10
9
8
7
6
5
4
3
2
1
0
ppm
2.03
2.05
1.15
0.06
1.00
2.06
12.02
3.04

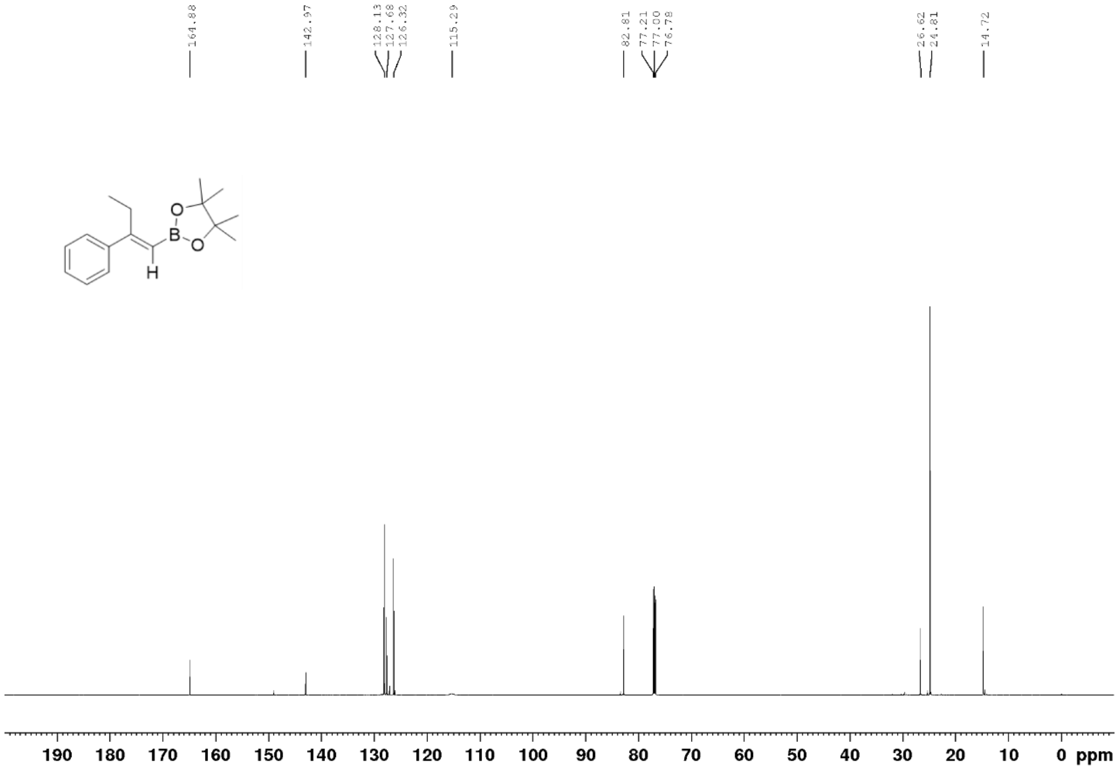
164.88
142.97
128.13
127.68
126.32
115.29
82.81
77.21
77.00
76.78
26.62
24.81
14.72
190
180
170
160
150
140
130
120
110
100
90
80
70
60
50
40
30
20
10
0 ppm

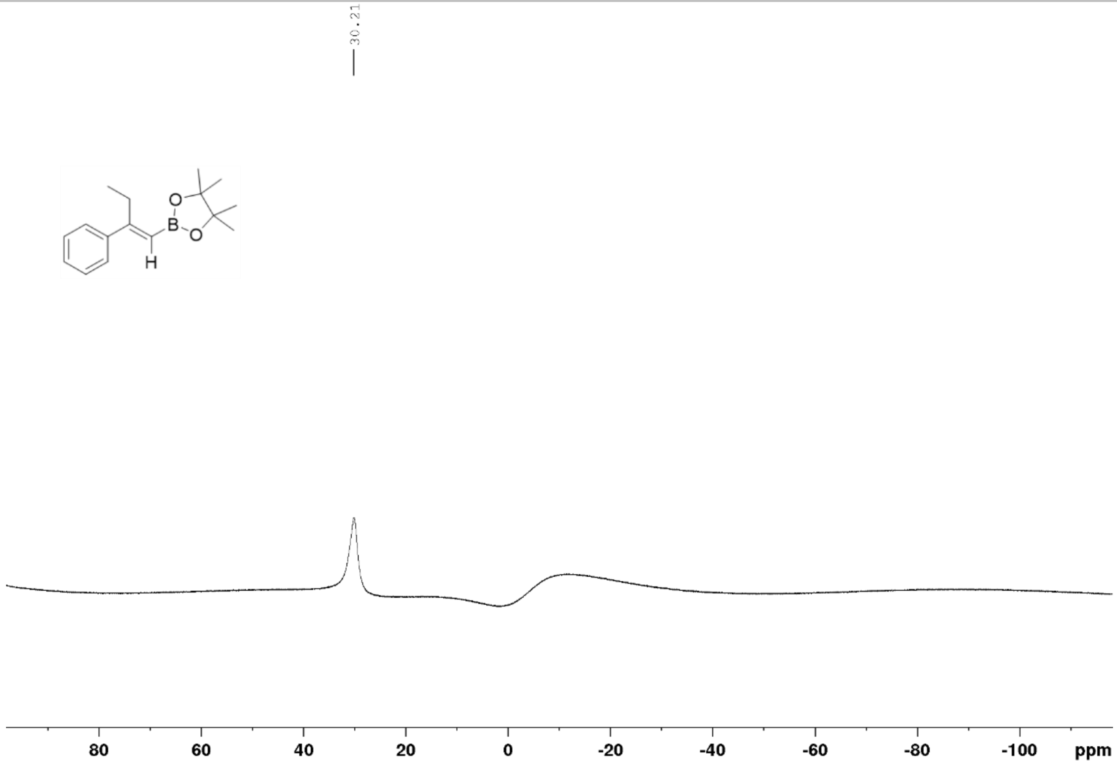
30.21
80
60
40
20
0
-20
-40
-60
-80
-100
ppm

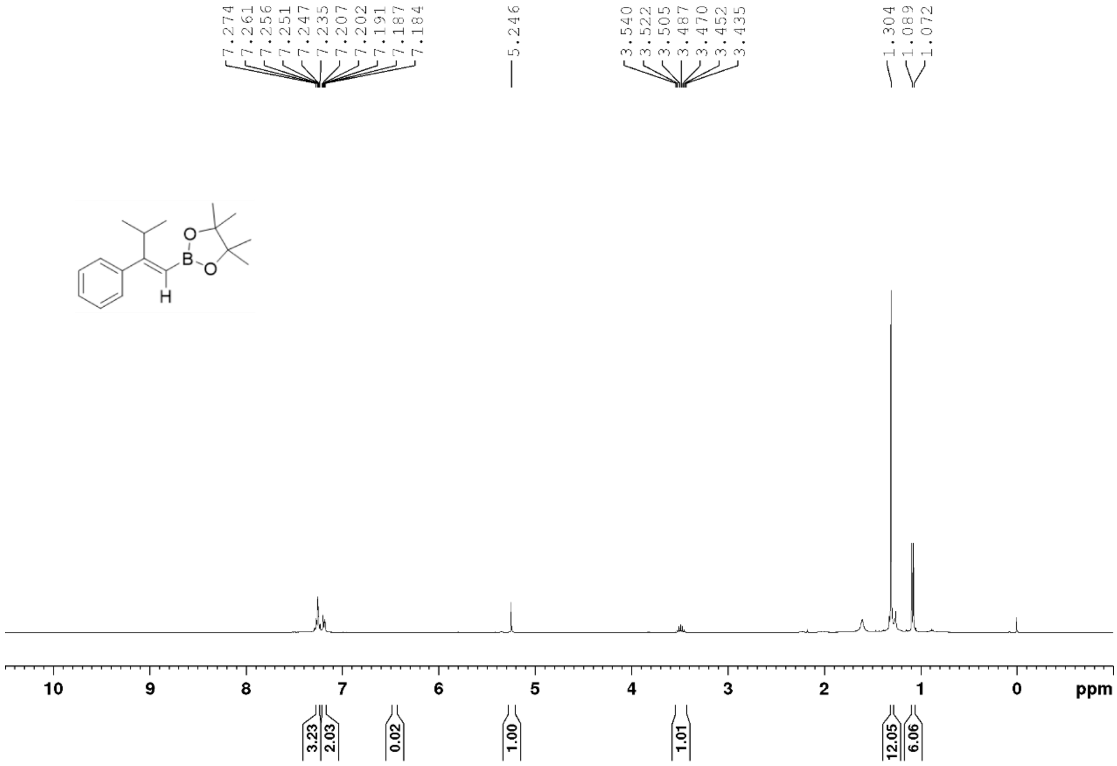
7.274
7.261
7.256
7.251
7.247
7.235
7.207
7.202
7.191
7.187
7.184
5.246
3.540
3.522
3.505
3.487
3.470
3.452
3.435
1.304
1.089
1.072
10
9
8
7
6
5
4
3
2
1
0
ppm
3.23
2.03
0.02
1.00
1.01
12.05
6.06

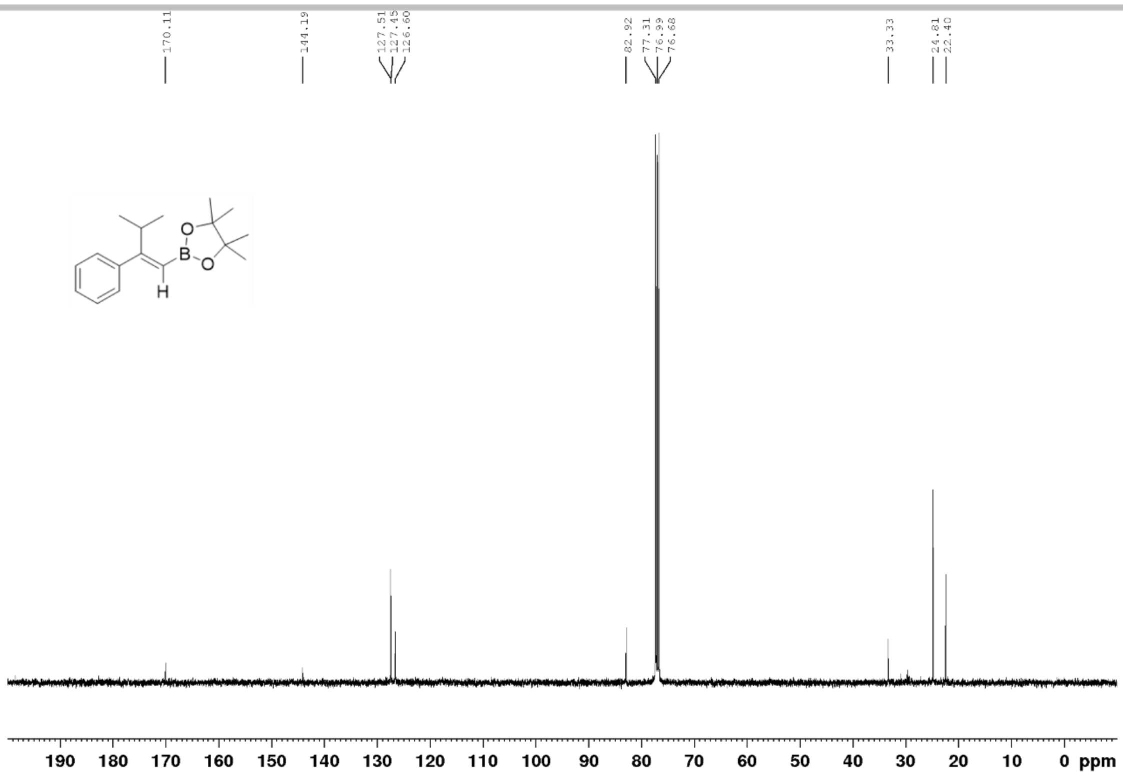
170.11
144.19
127.51
127.45
126.60
82.92
77.31
76.99
76.68
33.33
24.81
22.40
190 180 170 160 150 140 130 120 110 100 90 80 70 60 50 40 30 20 10 0 ppm

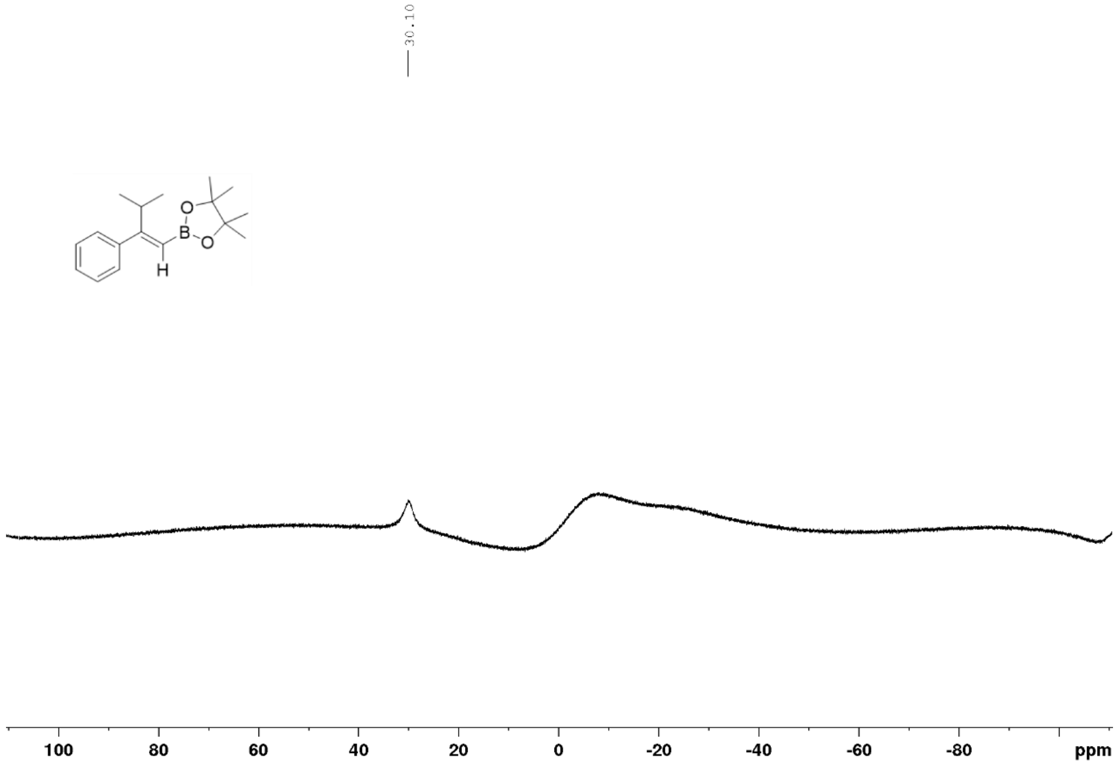
30.10
100 80 60 40 20 0 -20 -40 -60 -80 ppm

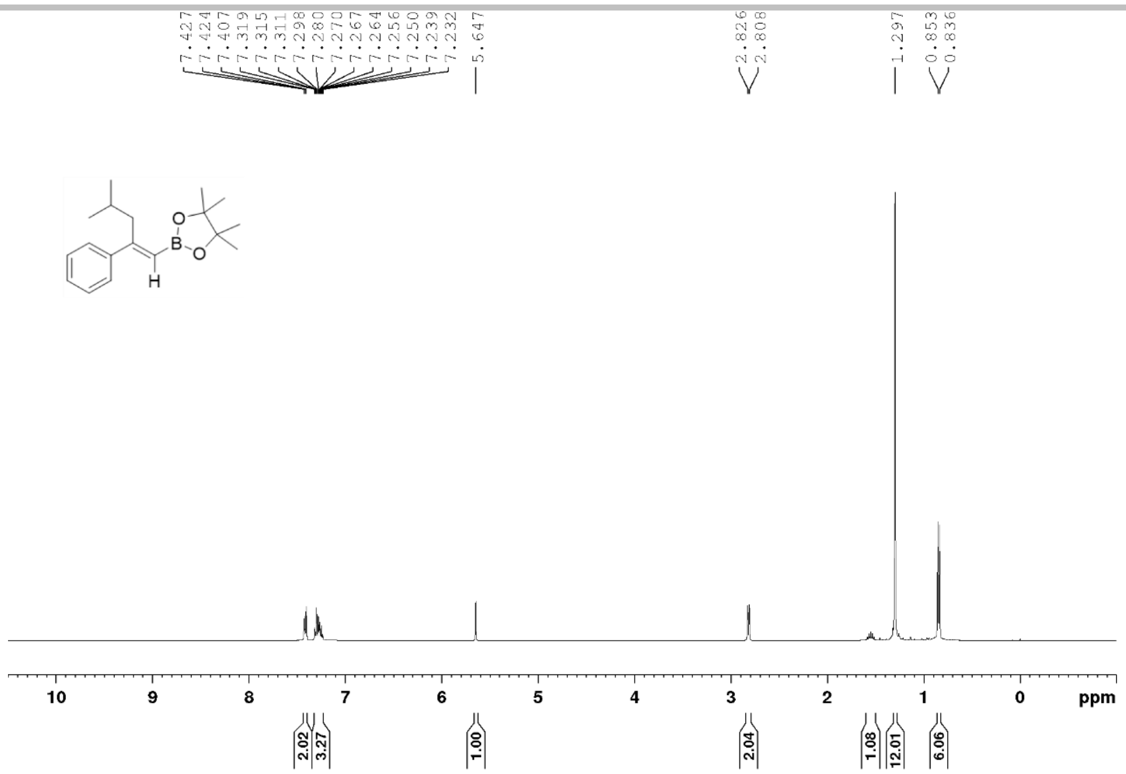

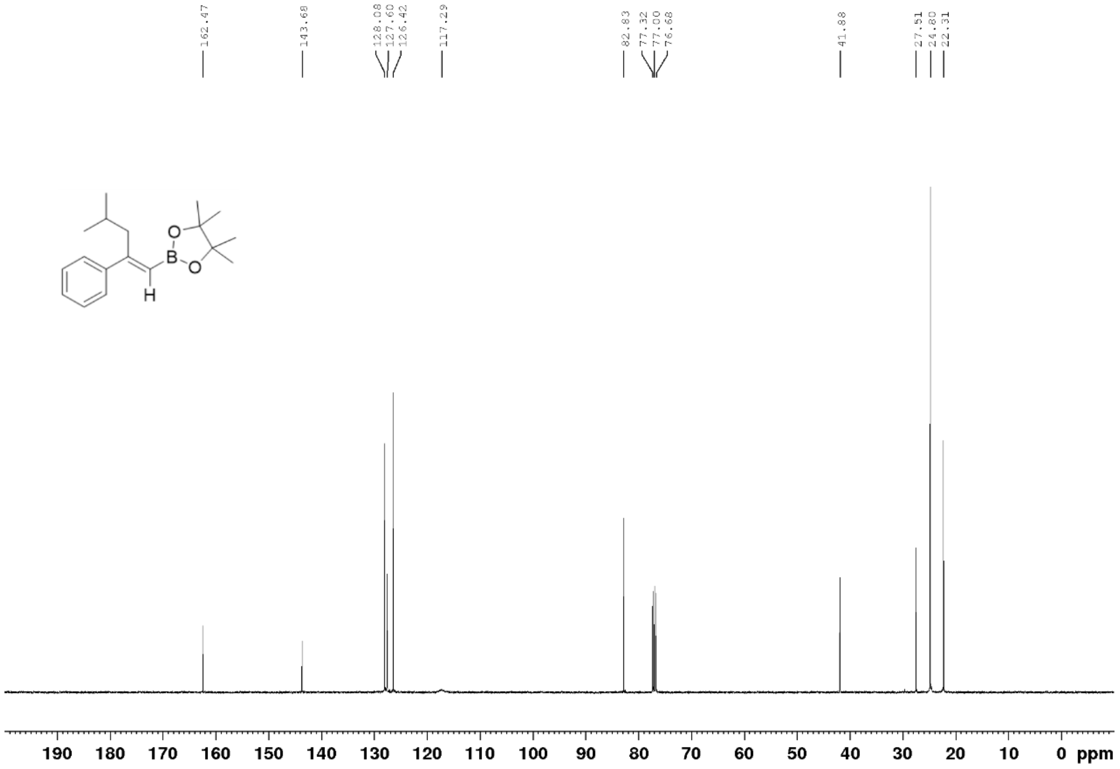

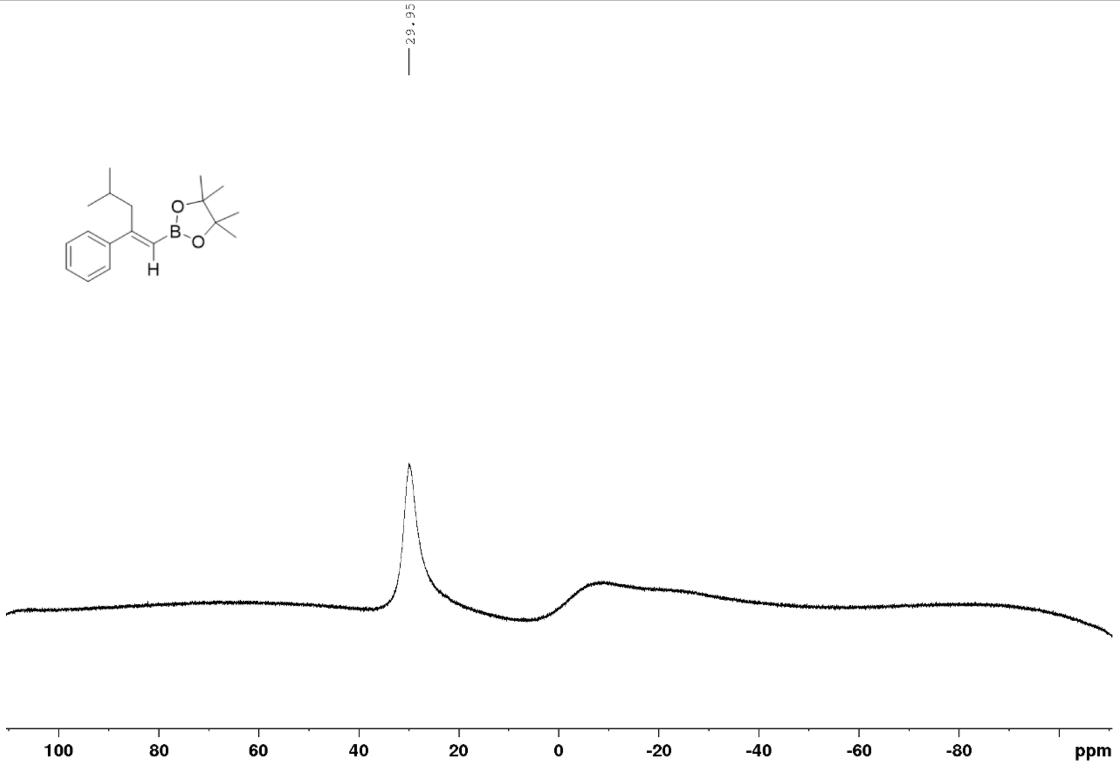
29.95
100
80
60
40
20
0
-20
-40
-60
-80
ppm

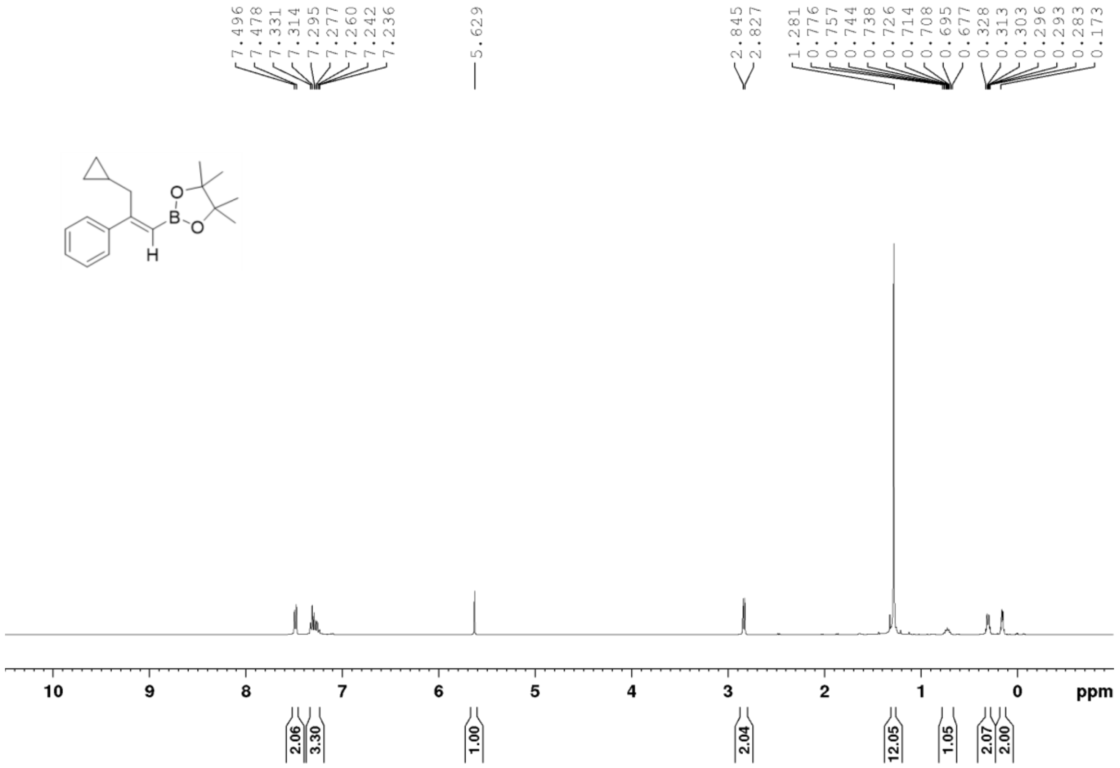
7.496
7.478
7.331
7.314
7.295
7.277
7.260
7.242
7.236
5.629
2.845
2.827
1.281
0.776
0.757
0.744
0.738
0.726
0.714
0.708
0.695
0.677
0.328
0.313
0.303
0.296
0.293
0.283
0.173
10
9
8
7
6
5
4
3
2
1
0
ppm
2.06
3.30
1.00
2.04
12.05
1.05
2.07
2.00

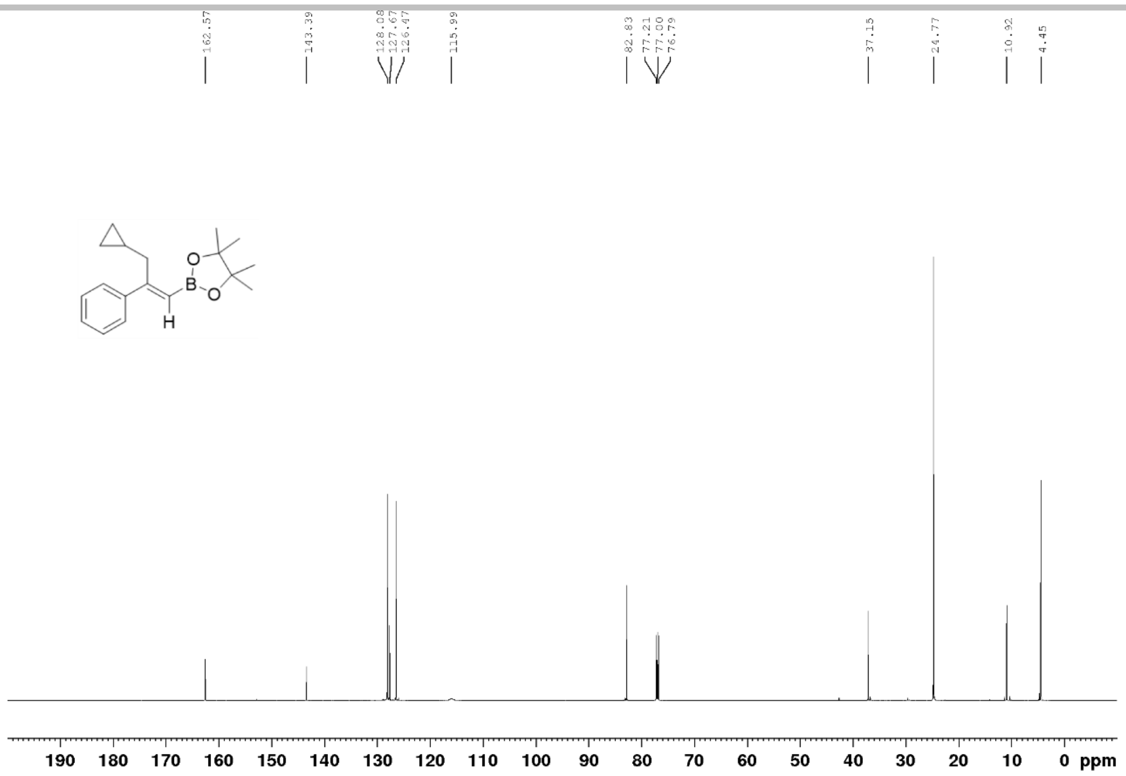
162.57
143.39
128.08
127.67
126.47
115.99
82.83
77.21
77.00
76.79
37.15
24.77
10.92
4.45
190 180 170 160 150 140 130 120 110 100 90 80 70 60 50 40 30 20 10 0 ppm

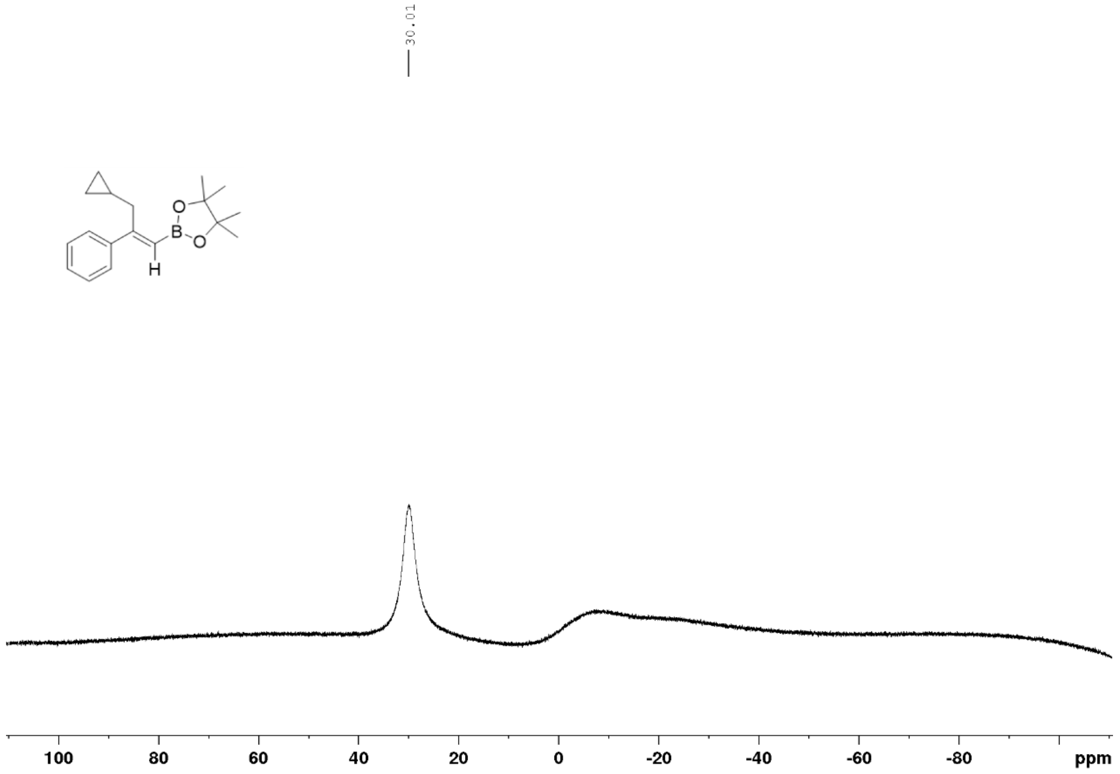
30.01
100 80 60 40 20 0 -20 -40 -60 -80 ppm

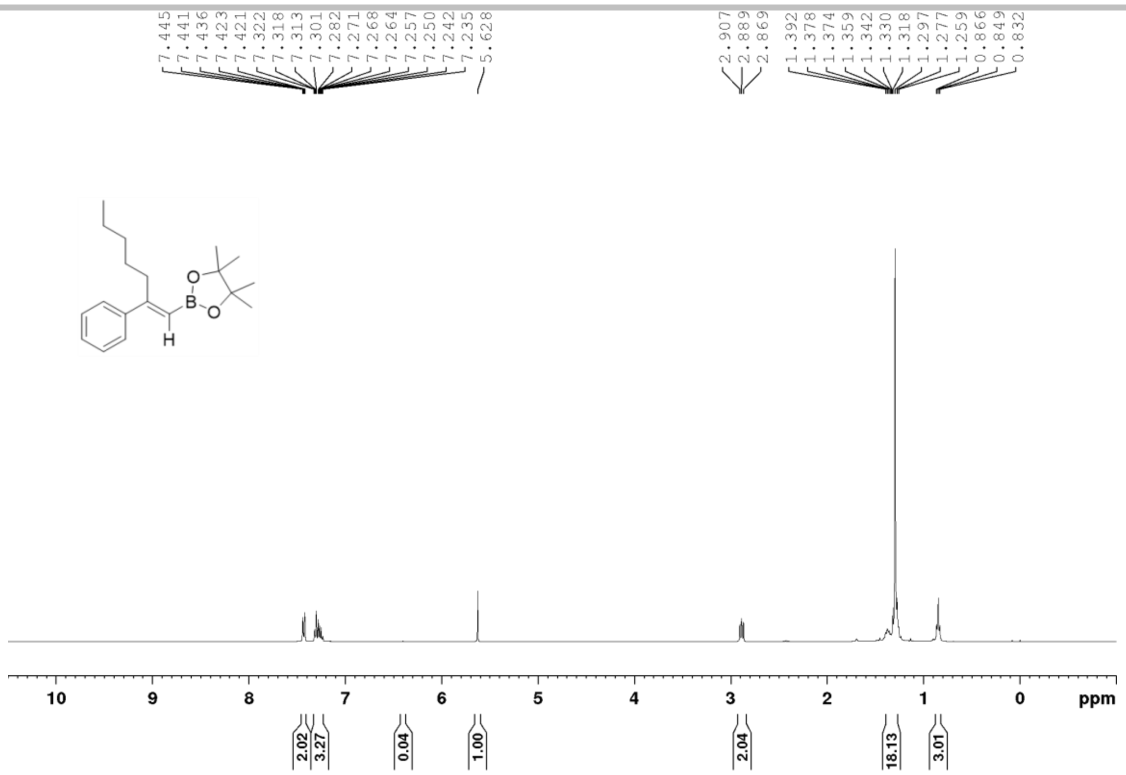
7.445
7.441
7.436
7.423
7.421
7.322
7.318
7.313
7.301
7.282
7.271
7.268
7.264
7.257
7.250
7.242
7.235
5.628
2.907
2.889
2.869
1.392
1.378
1.374
1.359
1.342
1.330
1.318
1.297
1.277
1.259
0.866
0.849
0.832
O
B
O
H
10
9
8
7
6
5
4
3
2
1
0
ppm
2.02
3.27
0.04
1.00
2.04
18.13
3.01

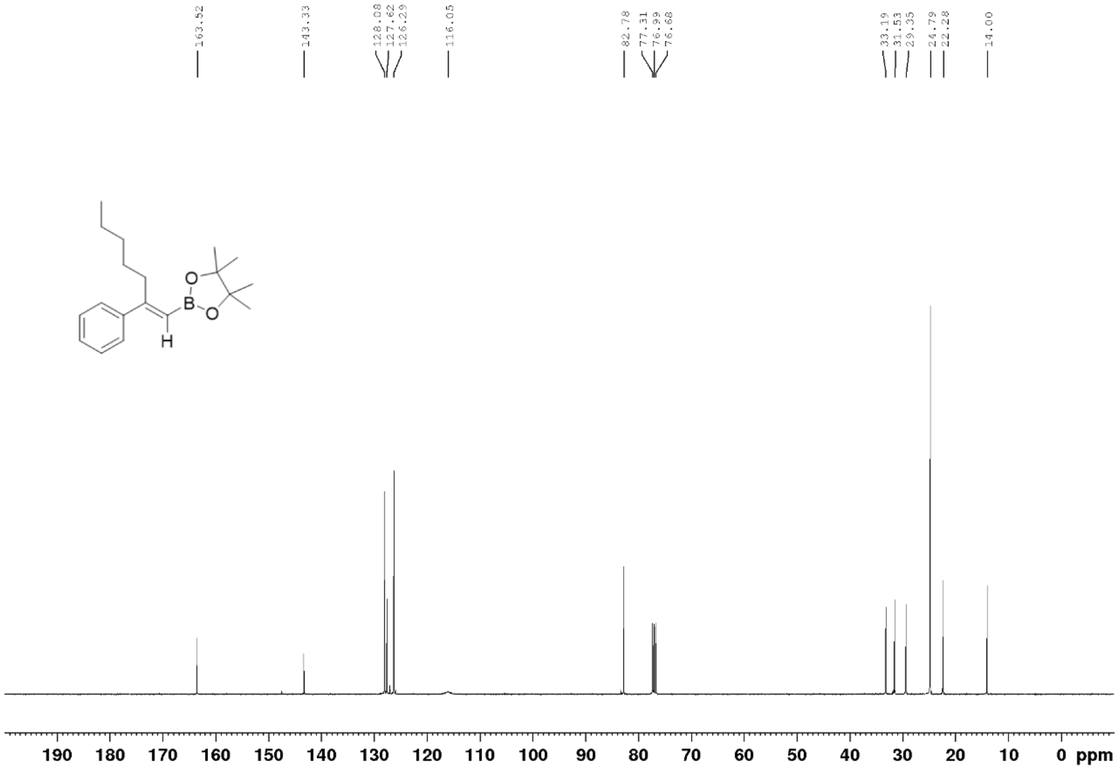
163.52
143.33
128.08
127.62
126.29
116.05
82.78
77.31
76.99
76.68
33.19
31.53
29.35
24.79
22.28
14.00
O
B
O
H
190
180
170
160
150
140
130
120
110
100
90
80
70
60
50
40
30
20
10
0 ppm

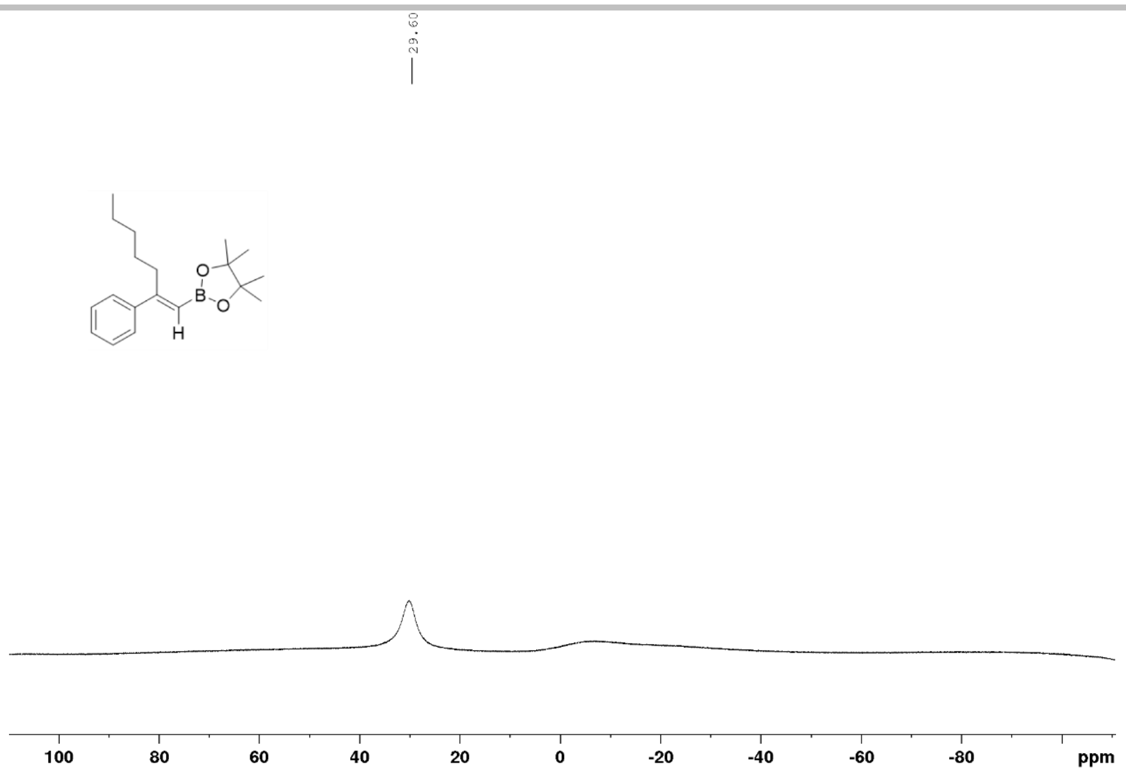
29.60
100
80
60
40
20
0
-20
-40
-60
-80
ppm

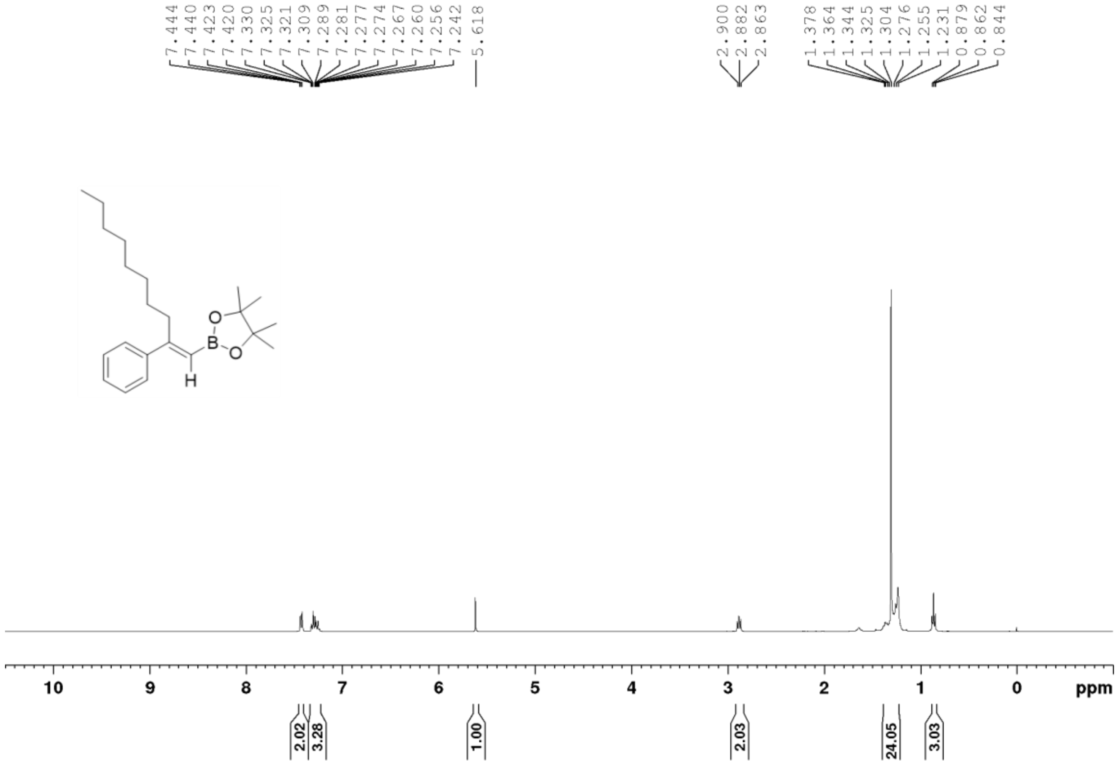
7.444
7.440
7.423
7.420
7.330
7.325
7.321
7.309
7.289
7.281
7.277
7.274
7.267
7.260
7.256
7.242
5.618
2.900
2.882
2.863
1.378
1.364
1.344
1.325
1.304
1.276
1.255
1.231
0.879
0.862
0.844
10
9
8
7
6
5
4
3
2
1
0
ppm
2.02
3.28
1.00
2.03
24.05
3.03

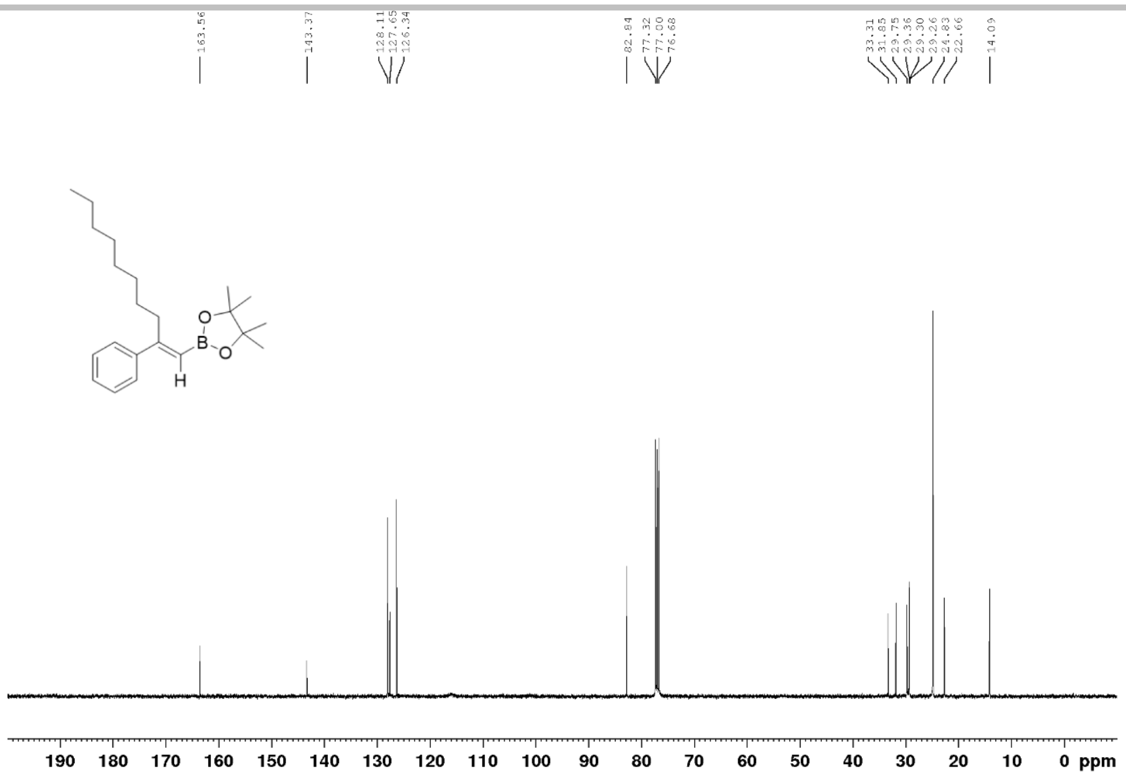
163.56
143.37
128.11
127.65
126.34
82.84
77.32
77.00
76.68
33.31
31.85
29.75
29.36
29.30
29.26
24.83
22.66
14.09
O
B
O
H
190 180 170 160 150 140 130 120 110 100 90 80 70 60 50 40 30 20 10 0 ppm

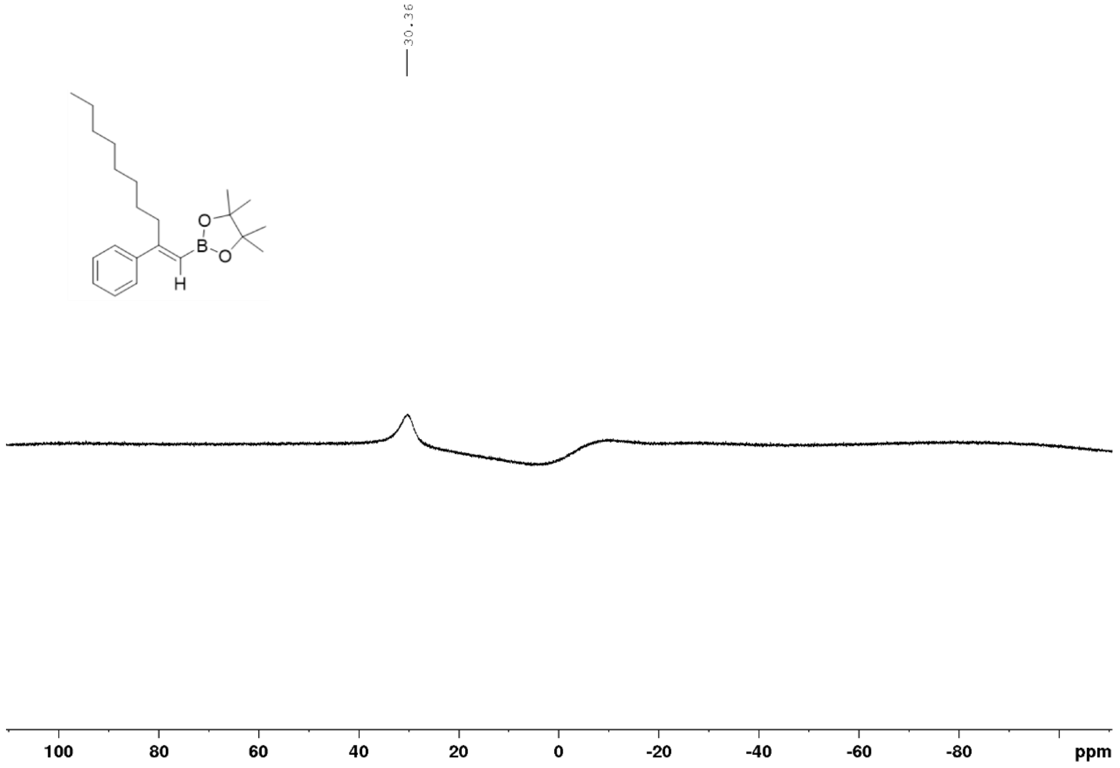
30.36
O
B
O
H
100 80 60 40 20 0 -20 -40 -60 -80 ppm

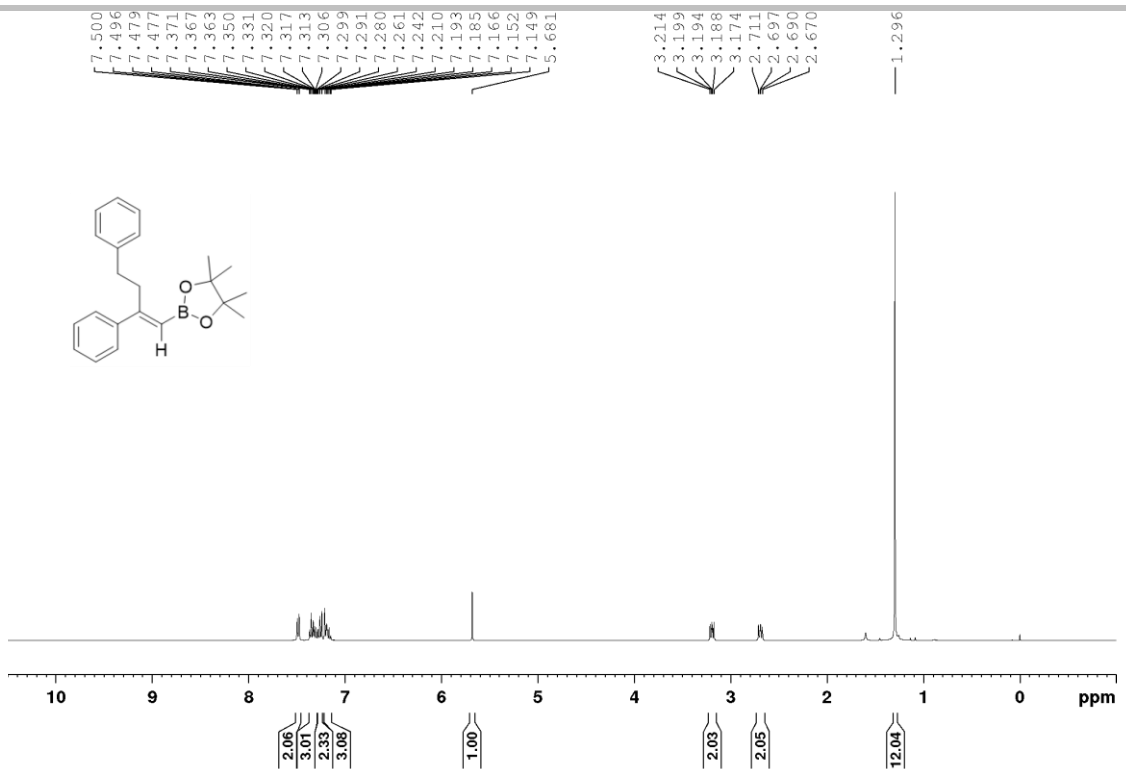

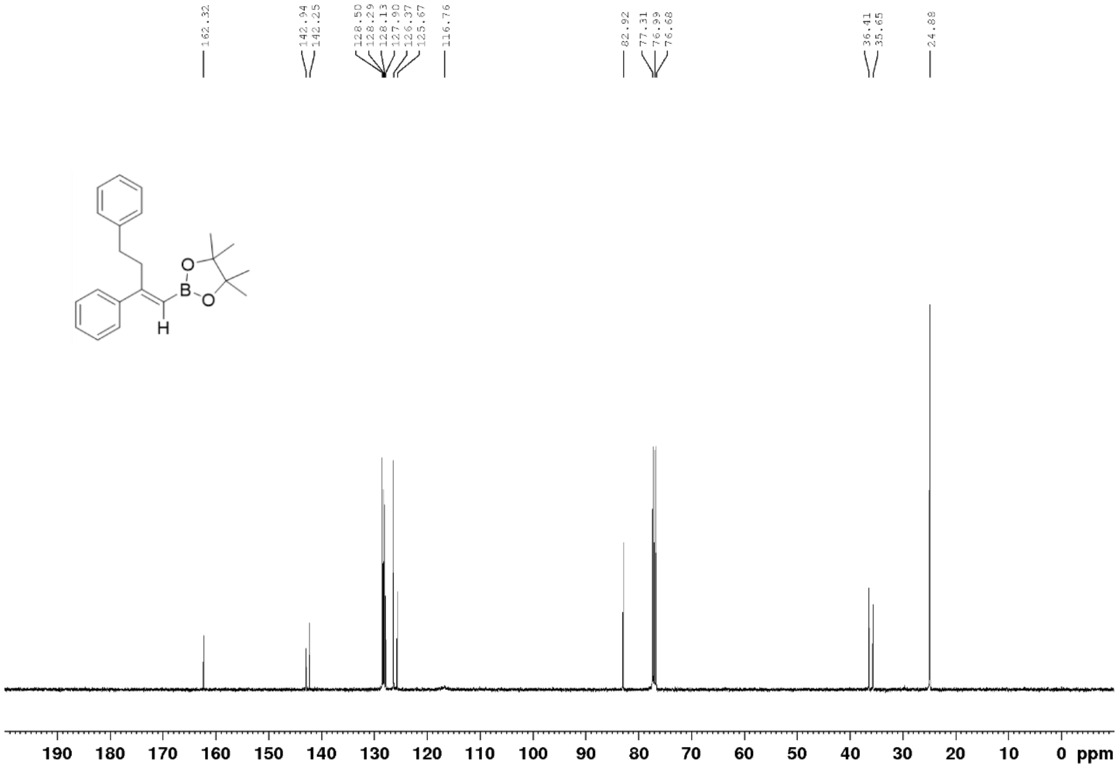

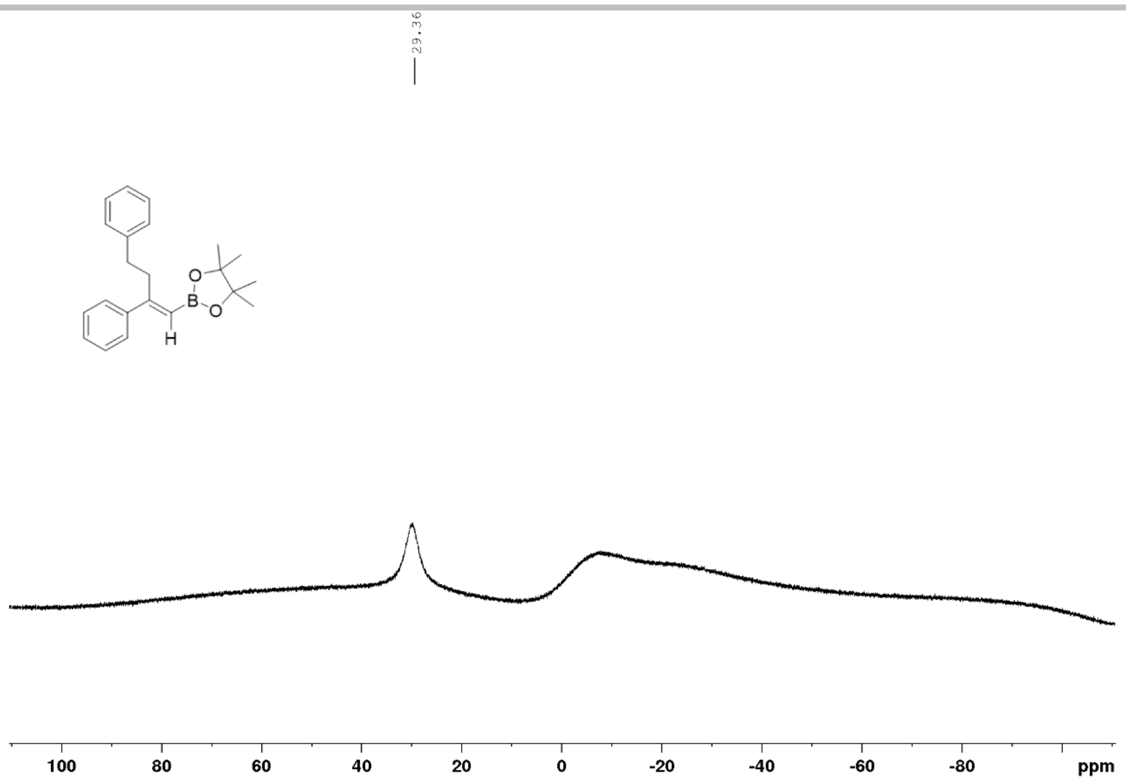
29.36
O
B
O
H
100
80
60
40
20
0
-20
-40
-60
-80
ppm

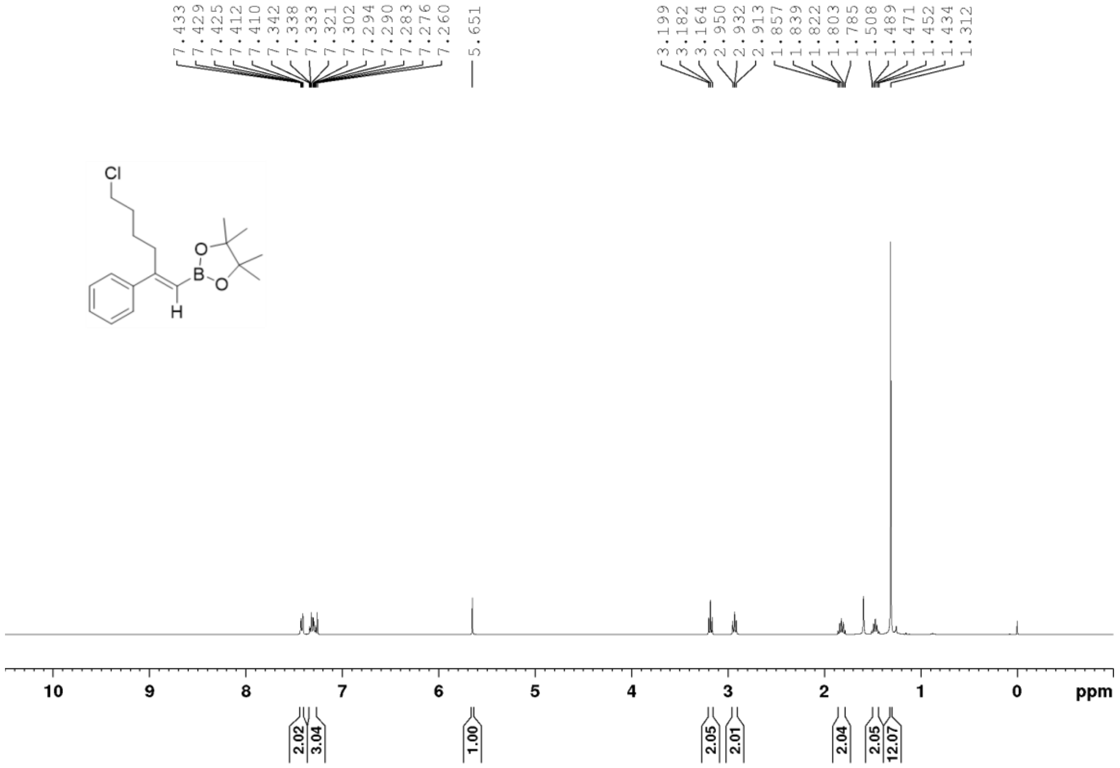
7.433
7.429
7.425
7.412
7.410
7.342
7.338
7.333
7.321
7.302
7.294
7.290
7.283
7.276
7.260
5.651
3.199
3.182
3.164
2.950
2.932
2.913
1.857
1.839
1.822
1.803
1.785
1.508
1.489
1.471
1.452
1.434
1.312
Cl
O
B
O
H
10
9
8
7
6
5
4
3
2
1
0
ppm
2.02
3.04
1.00
2.05
2.01
2.04
2.05
12.07

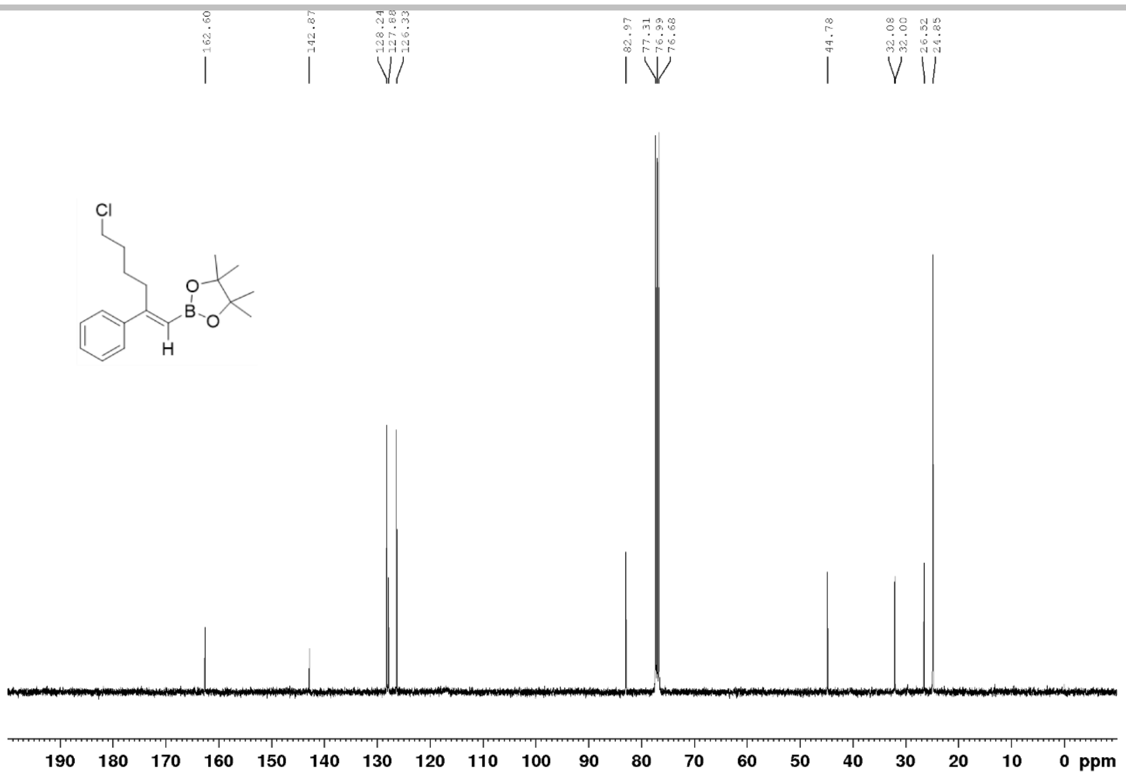
162.60
142.87
128.24
127.88
126.33
82.97
77.31
76.99
76.68
44.78
32.08
32.00
26.52
24.85
Cl
O
B
O
H
190 180 170 160 150 140 130 120 110 100 90 80 70 60 50 40 30 20 10 0 ppm

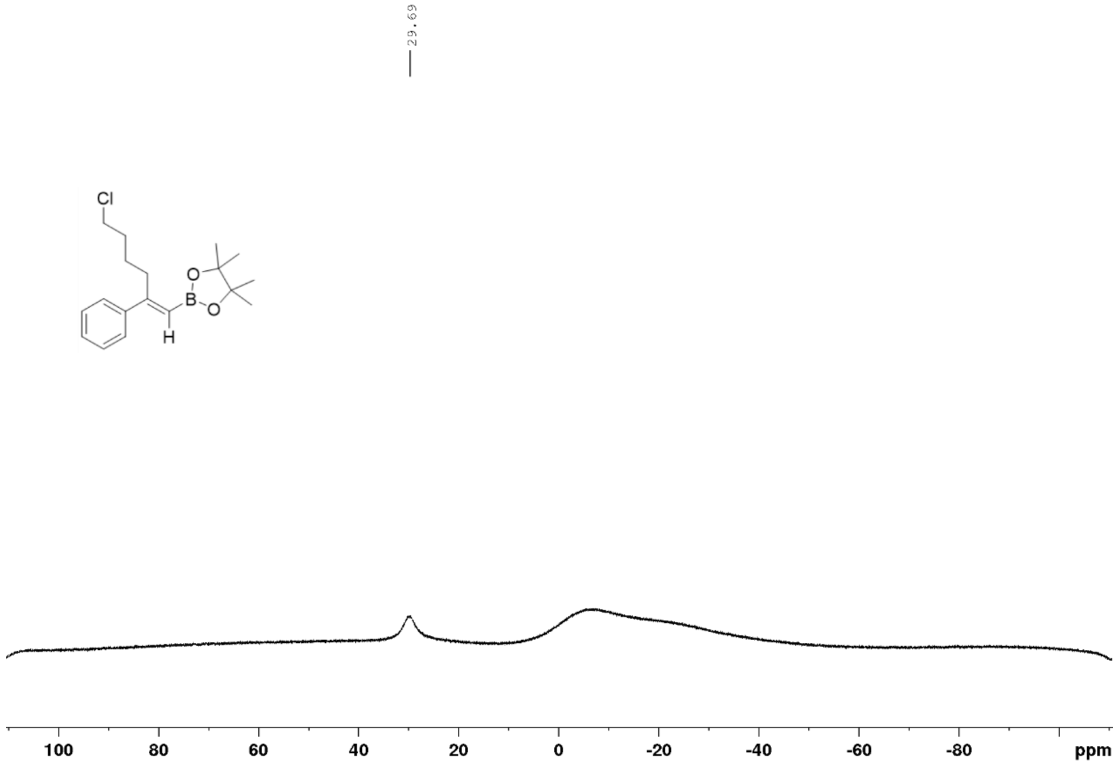
29.69
Cl
O
B
O
H
100 80 60 40 20 0 -20 -40 -60 -80 ppm

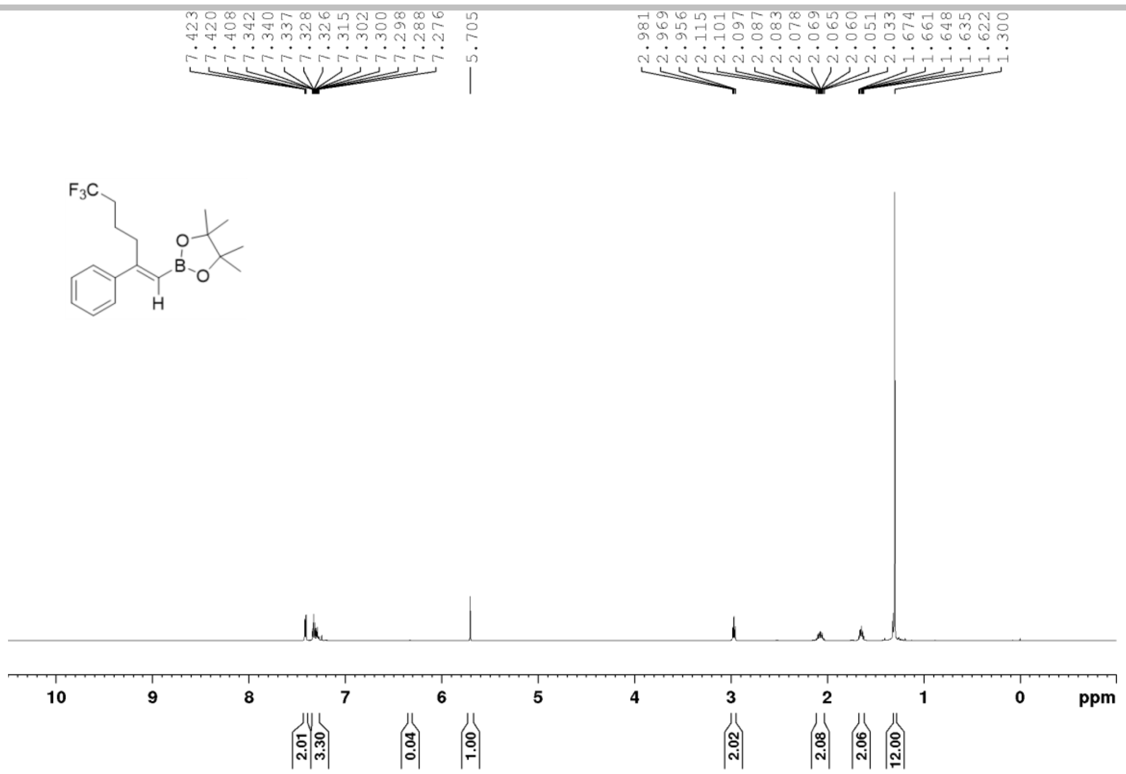

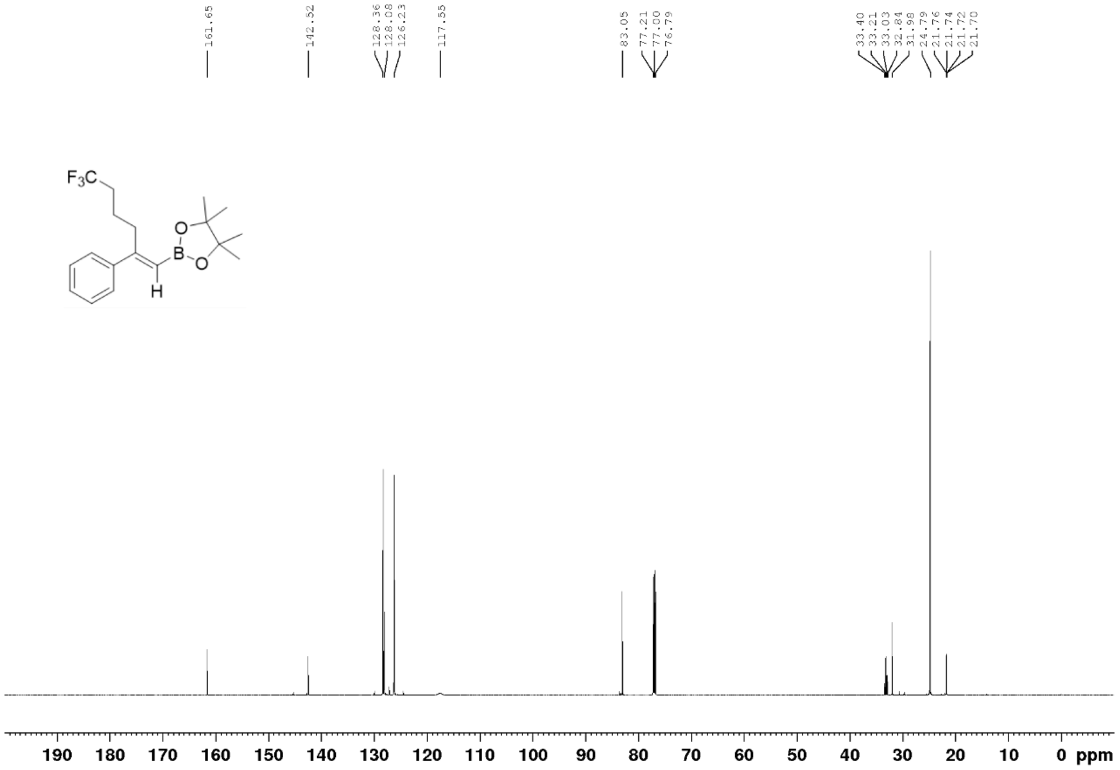

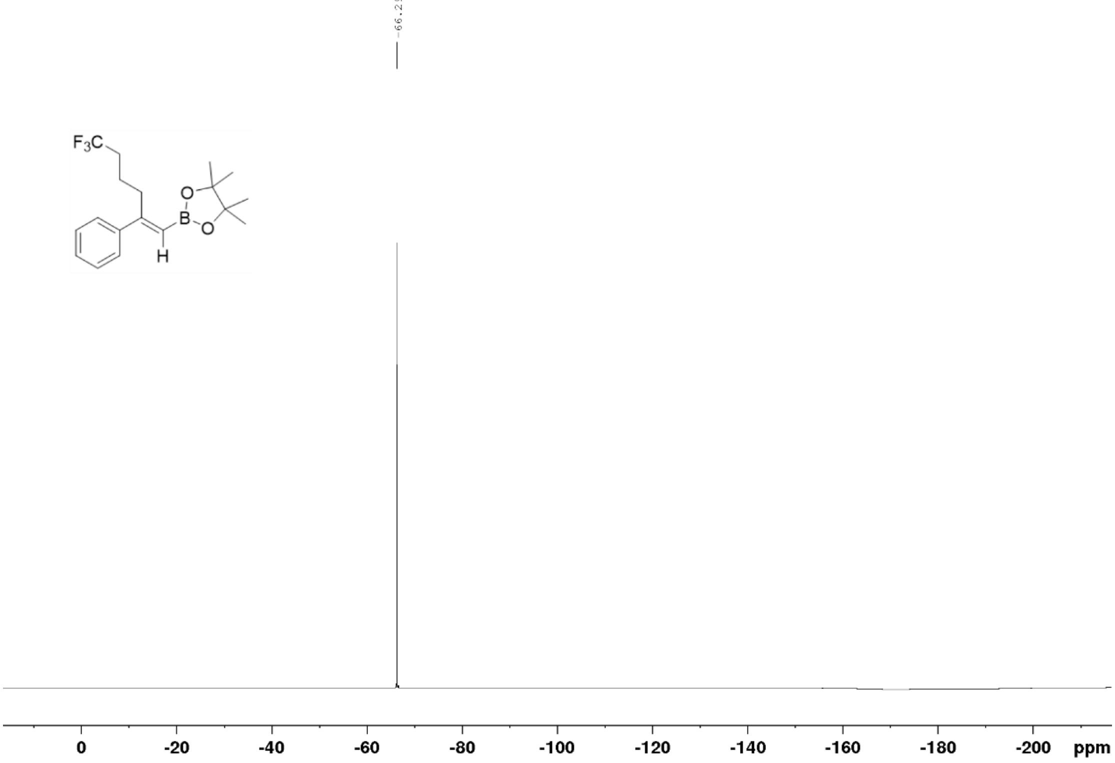
-66.29
F3C
O
B
O
H
0
-20
-40
-60
-80
-100
-120
-140
-160
-180
-200
ppm

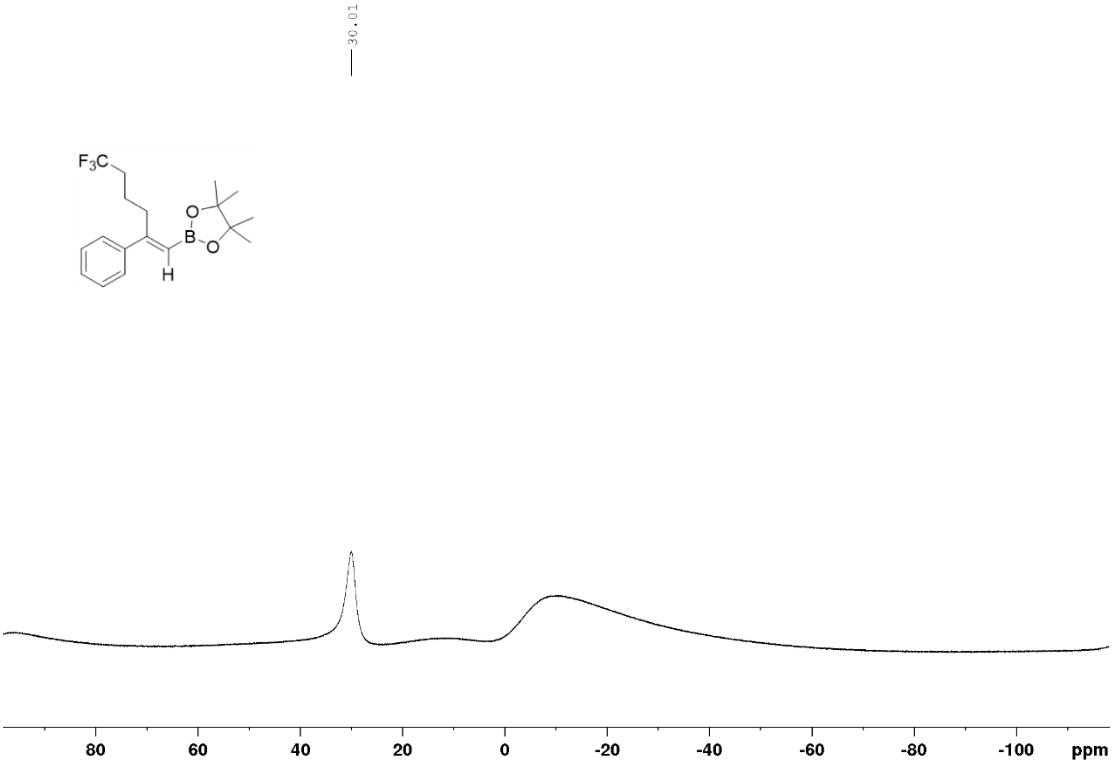
30.01
F3C
O
B
O
H
80
60
40
20
0
-20
-40
-60
-80
-100
ppm

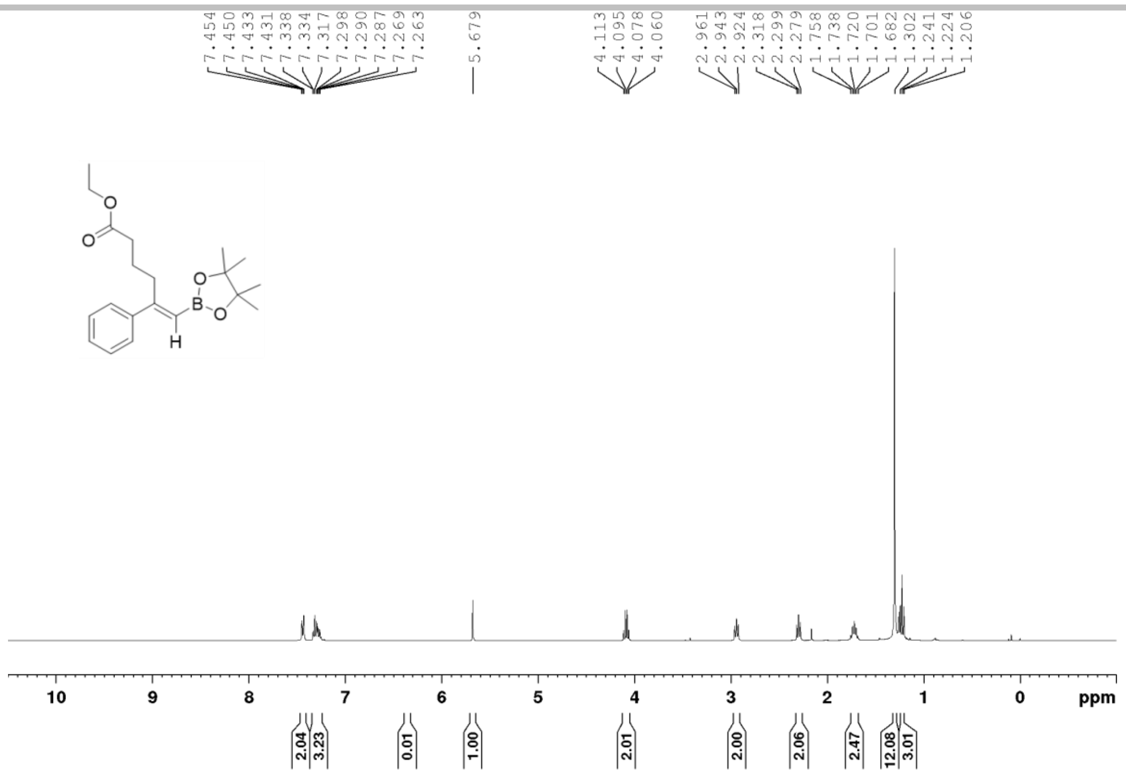
7.454
7.450
7.433
7.431
7.338
7.334
7.317
7.298
7.290
7.287
7.269
7.263
5.679
4.113
4.095
4.078
4.060
2.961
2.943
2.924
2.318
2.299
2.279
1.758
1.738
1.720
1.701
1.682
1.302
1.241
1.224
1.206
O
O
B
O
O
H
10
9
8
7
6
5
4
3
2
1
0
ppm
2.04
3.23
0.01
1.00
2.01
2.00
2.06
2.47
12.08
3.01

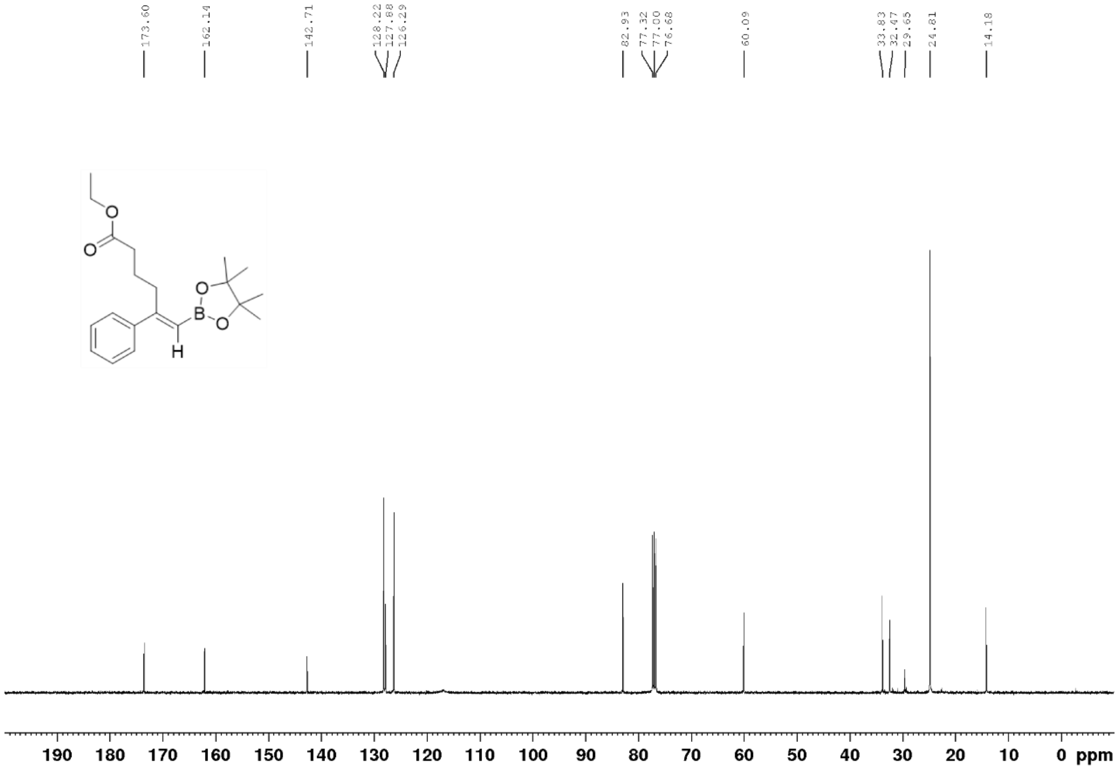
173.60
162.14
142.71
128.22
127.88
126.29
82.93
77.32
77.00
76.68
60.09
33.83
32.47
29.65
24.81
14.18
O
O
B
O
O
H
190
180
170
160
150
140
130
120
110
100
90
80
70
60
50
40
30
20
10
0 ppm

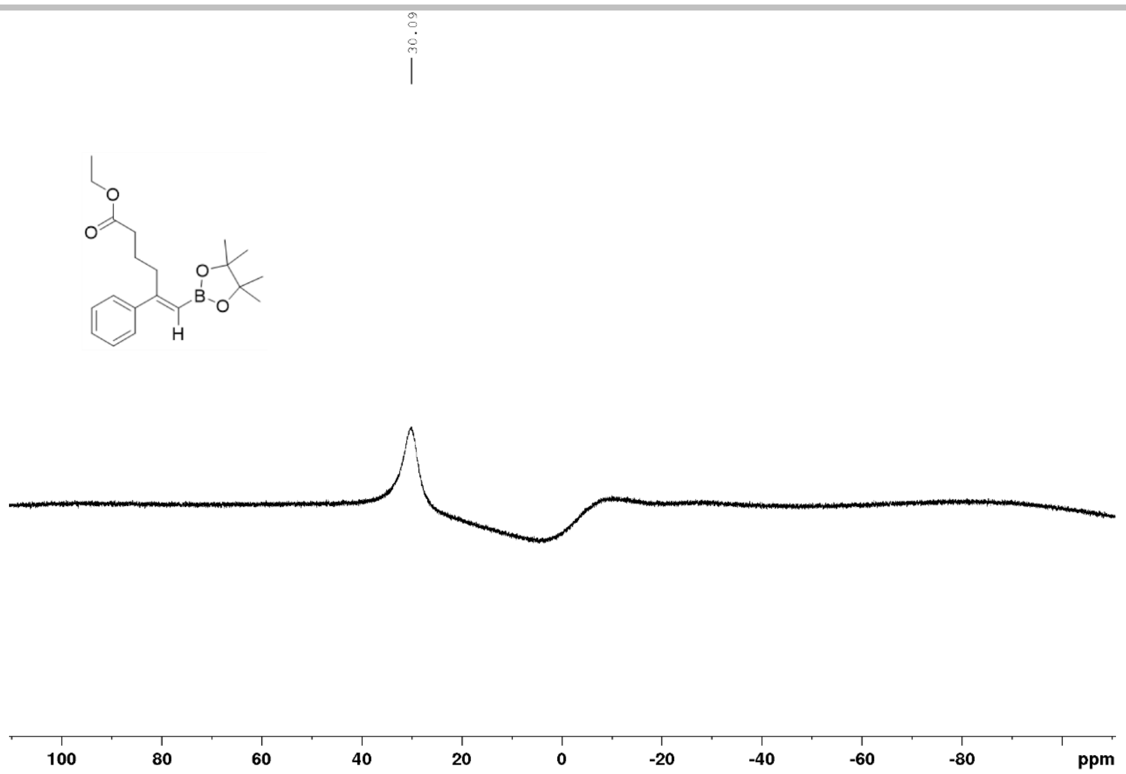
30.09
100
80
60
40
20
0
-20
-40
-60
-80
ppm

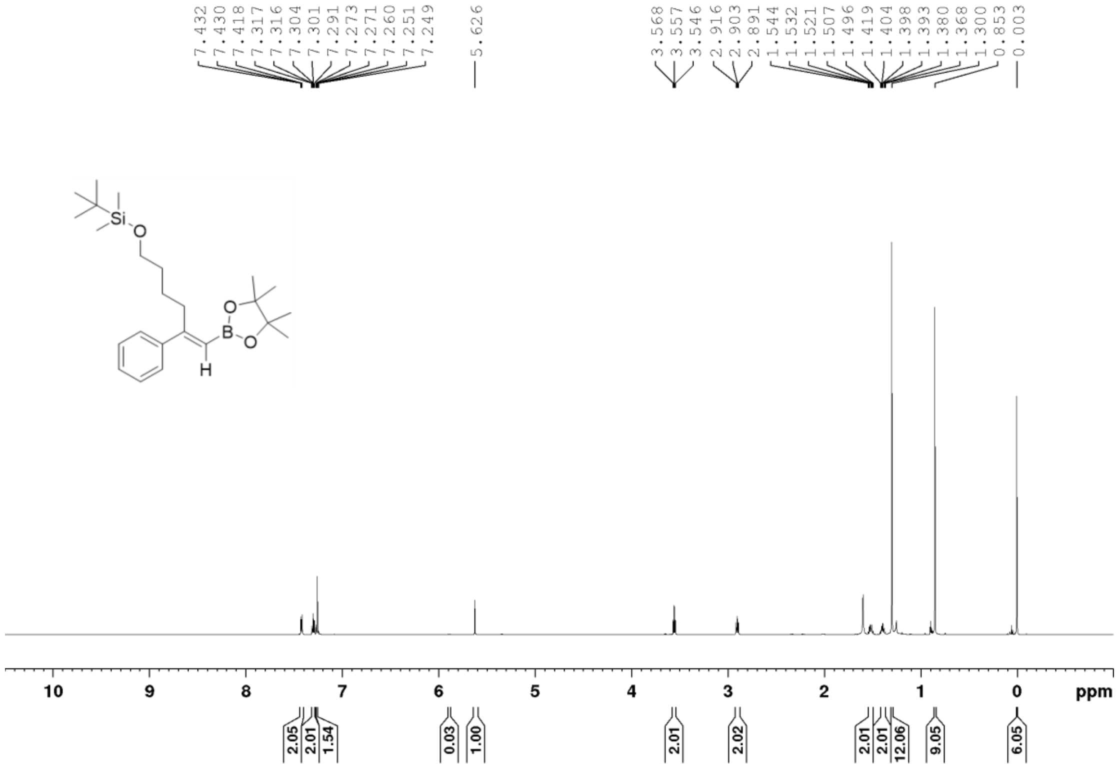
7.432
7.430
7.418
7.317
7.316
7.304
7.301
7.291
7.273
7.271
7.260
7.251
7.249
5.626
3.568
3.557
3.546
2.916
2.903
2.891
1.544
1.532
1.521
1.507
1.496
1.419
1.404
1.398
1.393
1.380
1.368
1.300
0.853
0.003
10
9
8
7
6
5
4
3
2
1
0
ppm
2.05
2.01
1.54
0.03
1.00
2.01
2.02
2.01
2.01
12.06
9.05
6.05

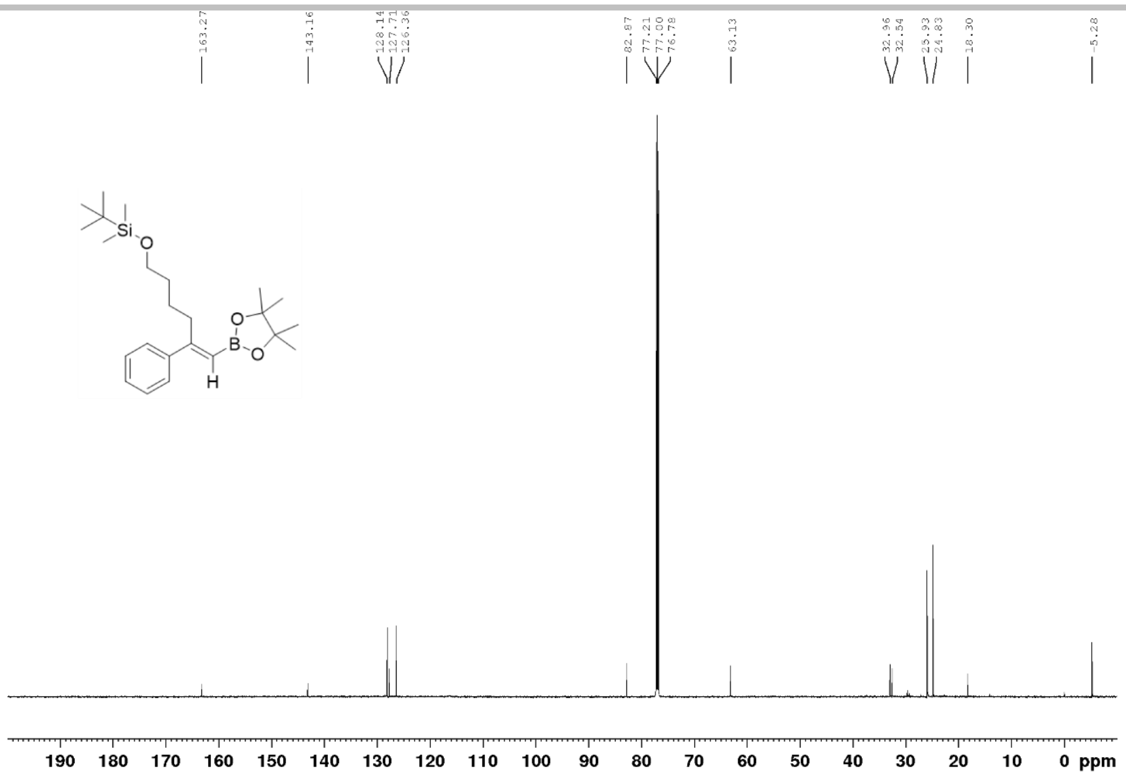
163.27
143.16
128.14
127.71
126.36
82.87
77.21
77.00
76.78
63.13
32.96
32.54
25.93
24.83
18.30
-5.28
190 180 170 160 150 140 130 120 110 100 90 80 70 60 50 40 30 20 10 0 ppm

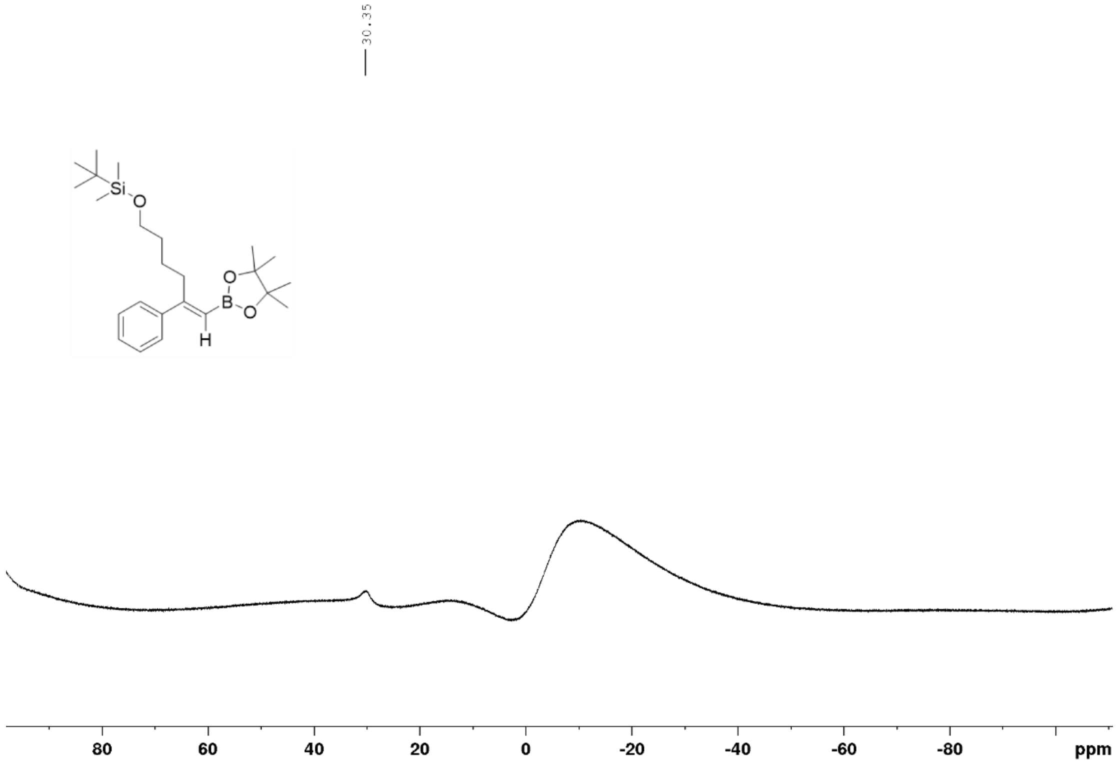
30.35
80 60 40 20 0 -20 -40 -60 -80 ppm

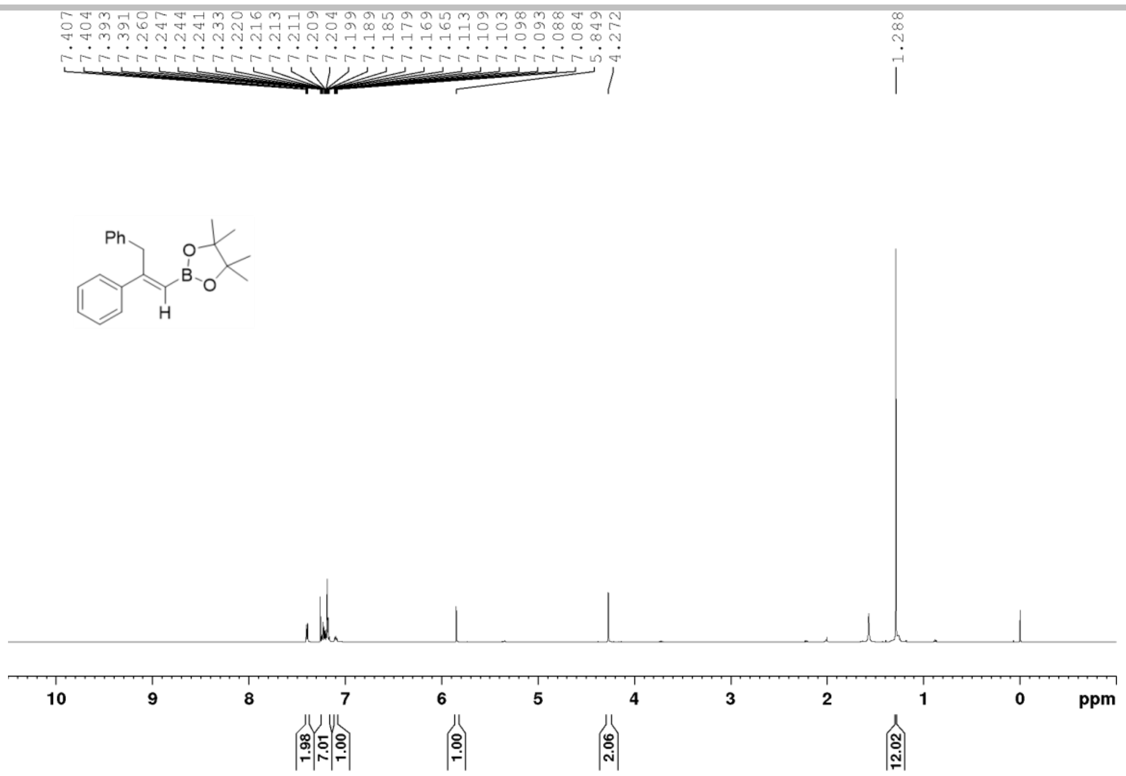
7.407
7.404
7.393
7.391
7.260
7.247
7.244
7.241
7.233
7.220
7.216
7.213
7.211
7.209
7.204
7.199
7.189
7.185
7.179
7.169
7.165
7.113
7.109
7.103
7.098
7.093
7.088
7.084
5.849
4.272
1.288
Ph
O
B
O
H
10 9 8 7 6 5 4 3 2 1 0 ppm
1.98
7.01
1.00
1.00
2.06
12.02

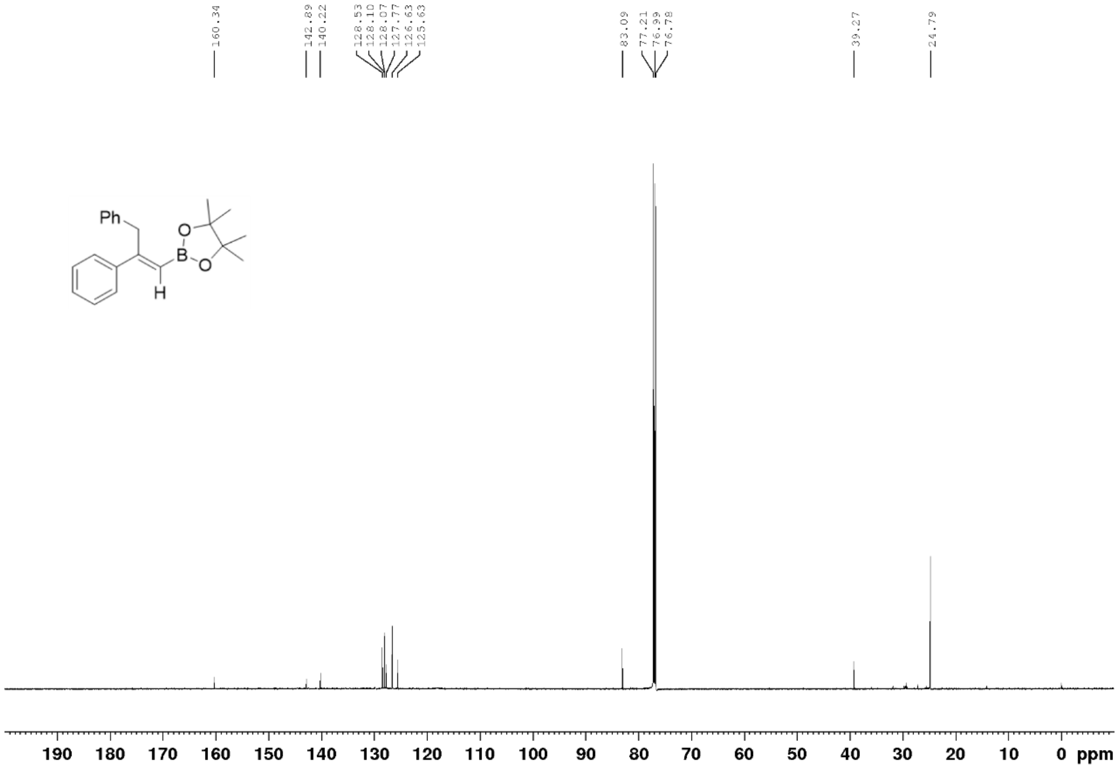
160.34
142.89
140.22
128.53
128.10
128.07
127.77
126.63
125.63
83.09
77.21
76.99
76.78
39.27
24.79
Ph
O
B
O
H
190 180 170 160 150 140 130 120 110 100 90 80 70 60 50 40 30 20 10 0 ppm

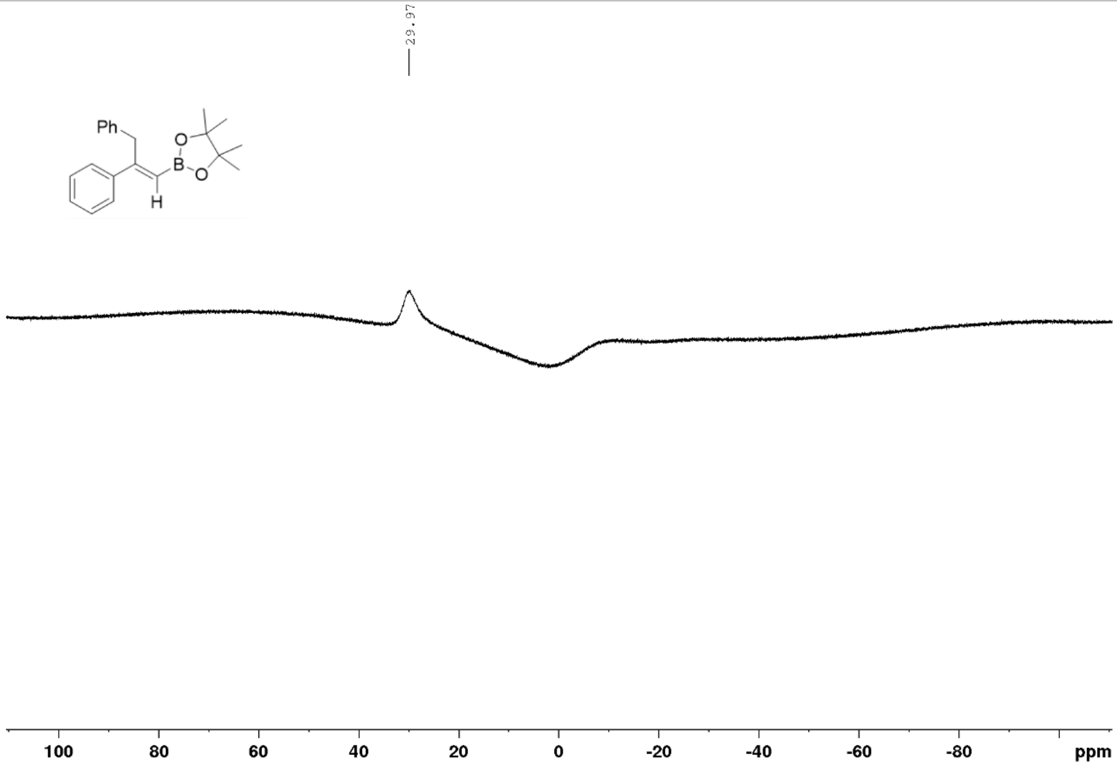
29.97
Ph
B
O
O
H
100
80
60
40
20
0
-20
-40
-60
-80
ppm

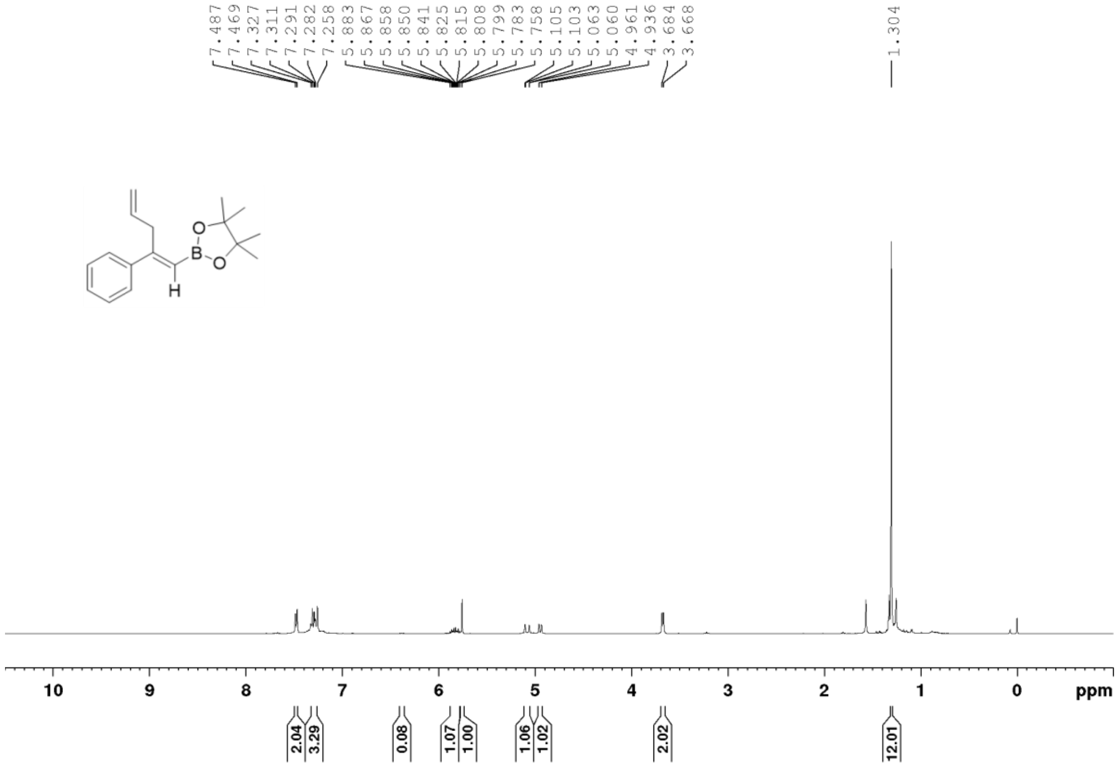
7.487
7.469
7.327
7.311
7.291
7.282
7.258
5.883
5.867
5.858
5.850
5.841
5.825
5.815
5.808
5.799
5.783
5.758
5.105
5.103
5.063
5.060
4.961
4.936
3.684
3.668
1.304
B
O
O
H
10
9
8
7
6
5
4
3
2
1
0
ppm
2.04
3.29
0.08
1.07
1.00
1.06
1.02
2.02
12.01

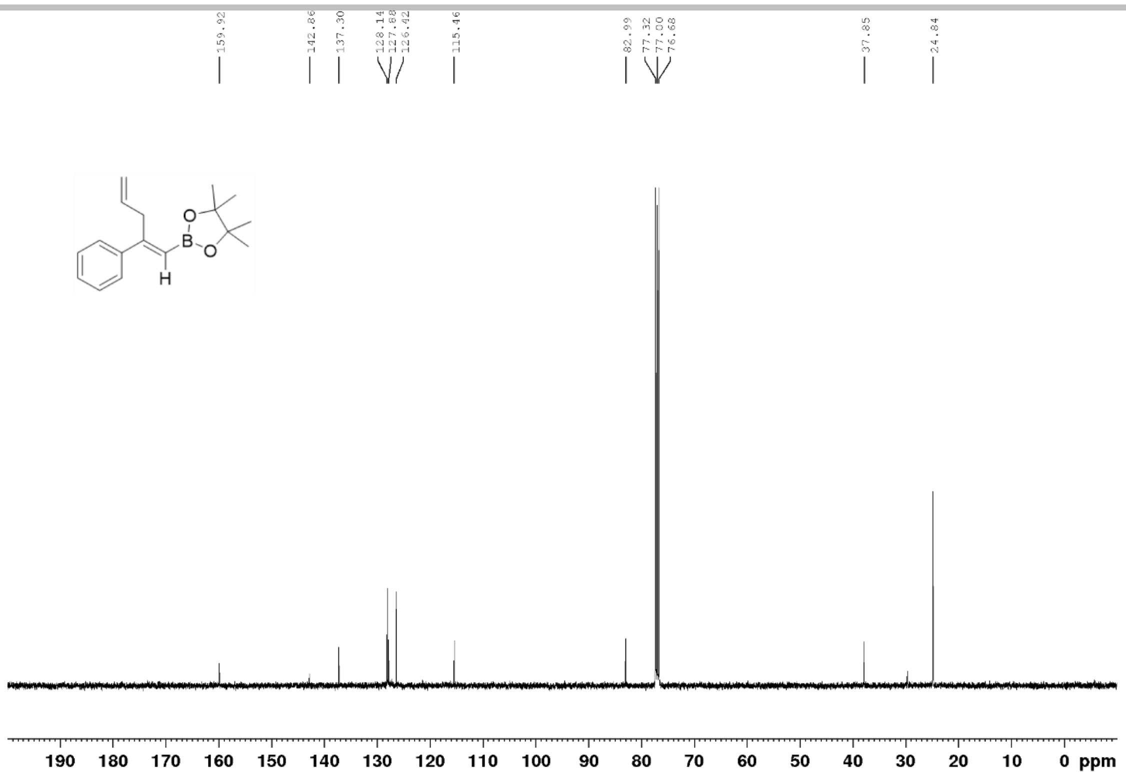
159.92
142.86
137.30
128.14
127.88
126.42
115.46
82.99
77.32
77.00
76.68
37.85
24.84
190 180 170 160 150 140 130 120 110 100 90 80 70 60 50 40 30 20 10 0 ppm

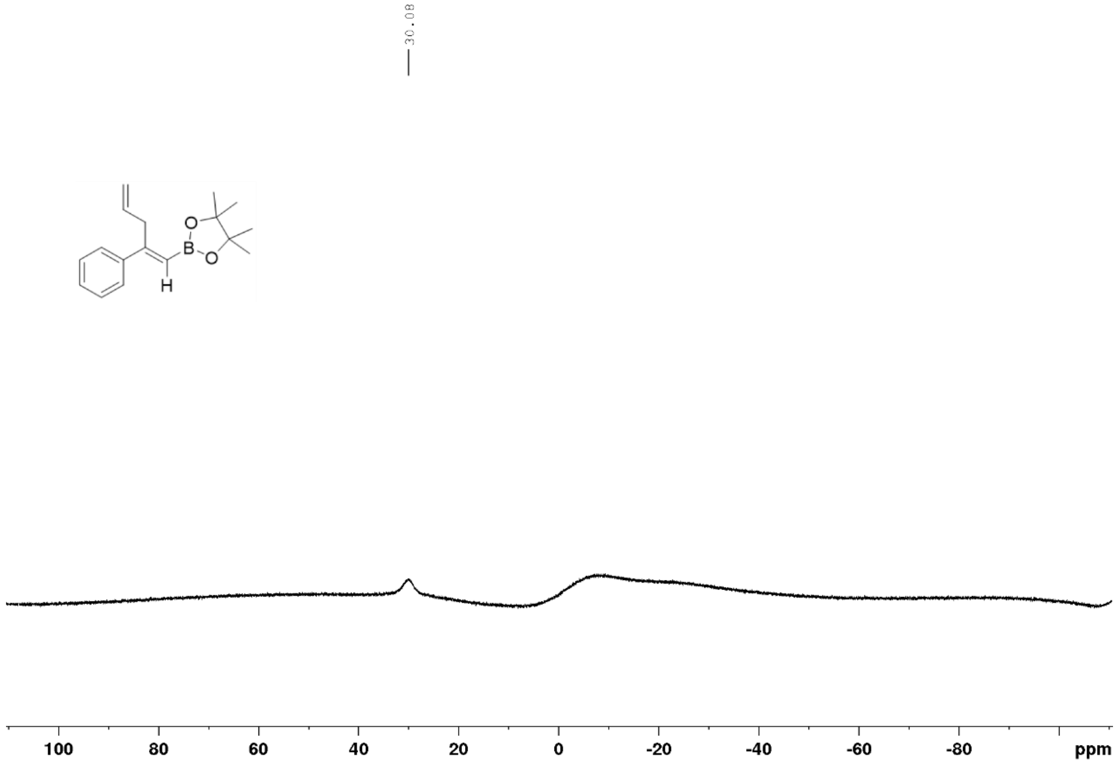
30.08
100 80 60 40 20 0 -20 -40 -60 -80 ppm

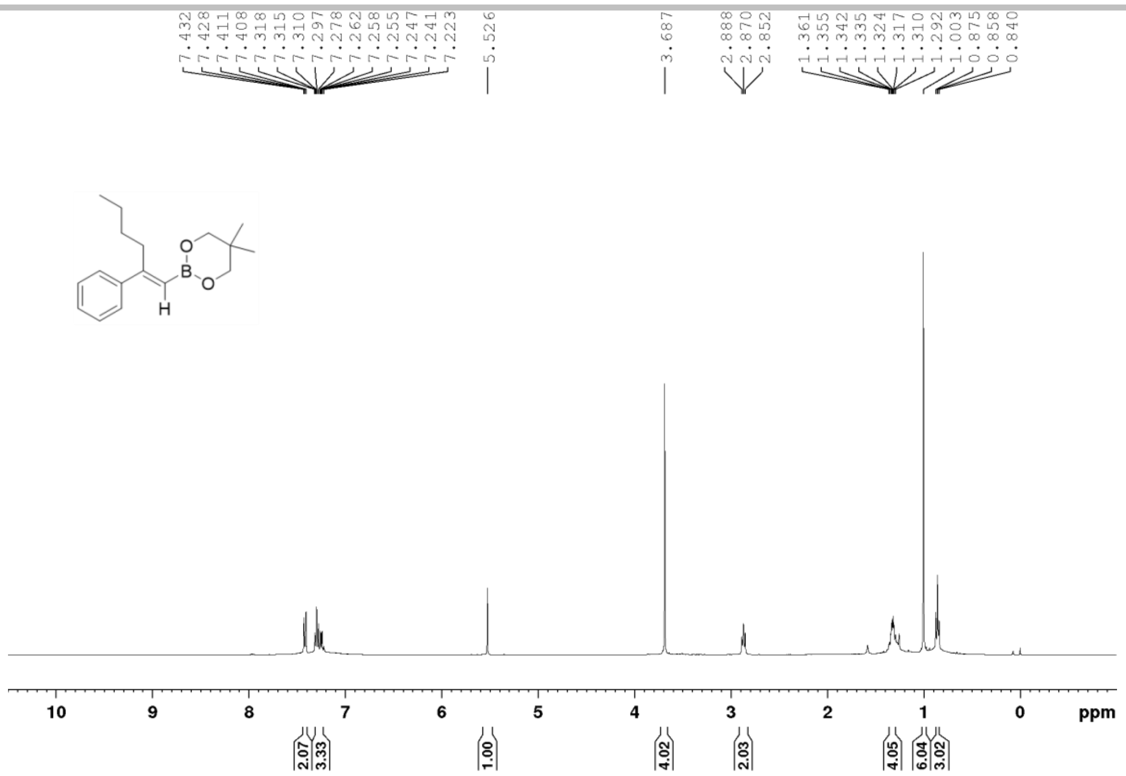
7.432
7.428
7.411
7.408
7.318
7.315
7.310
7.297
7.278
7.262
7.258
7.255
7.247
7.241
7.223
5.526
3.687
2.888
2.870
2.852
1.361
1.355
1.342
1.335
1.324
1.317
1.310
1.292
1.003
0.875
0.858
0.840
O
B
O
H
10
9
8
7
6
5
4
3
2
1
0
ppm
2.07
3.33
1.00
4.02
2.03
4.05
6.04
3.02

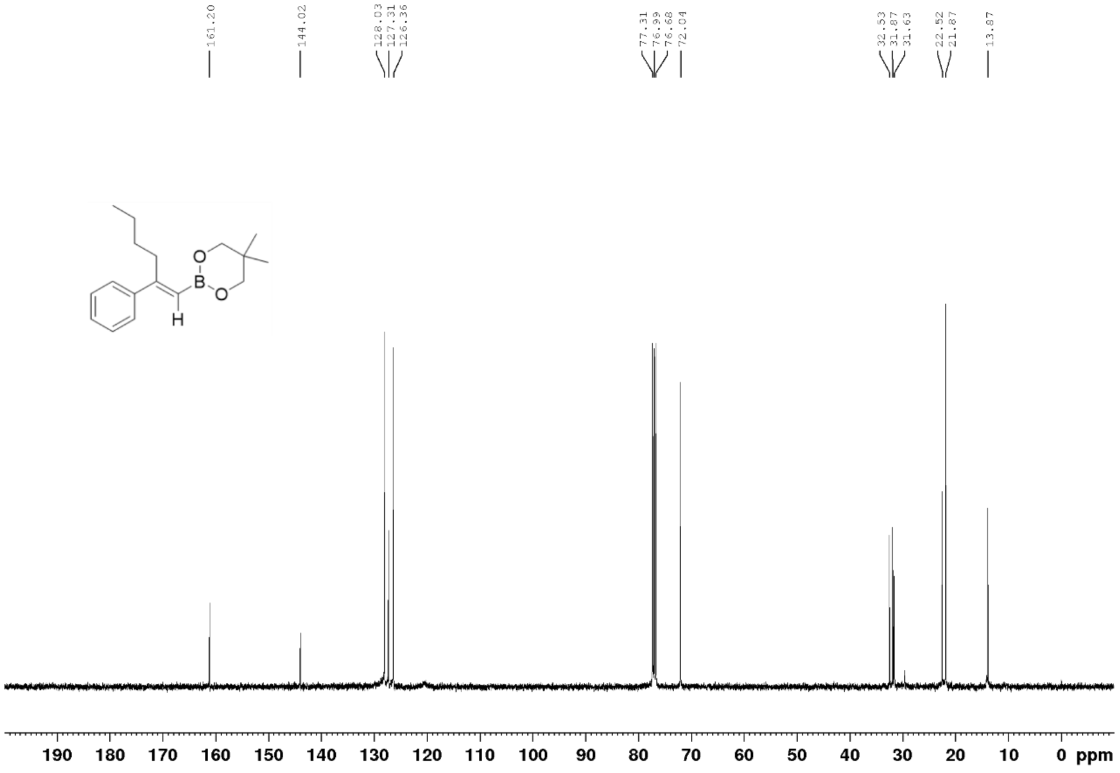
161.20
144.02
128.03
127.31
126.36
77.31
76.99
76.68
72.04
32.53
31.87
31.63
22.52
21.87
13.87
O
B
O
H
190
180
170
160
150
140
130
120
110
100
90
80
70
60
50
40
30
20
10
0 ppm

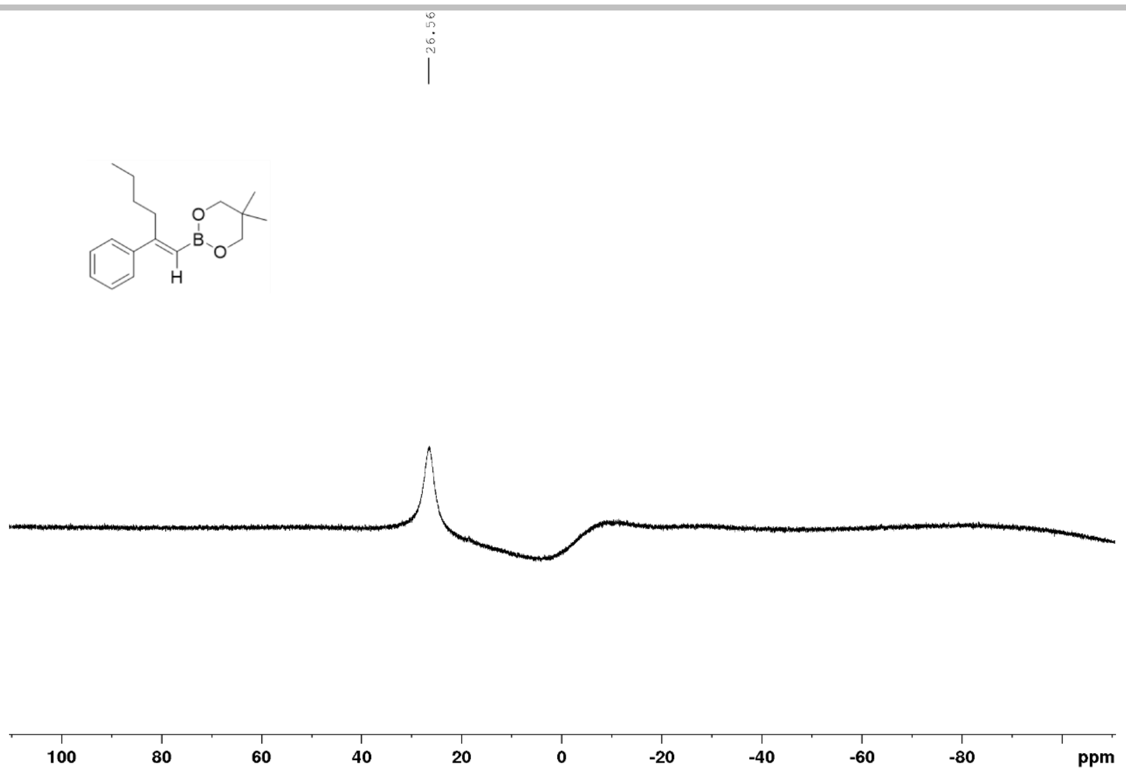
26.56
O
B
O
H
100
80
60
40
20
0
-20
-40
-60
-80
ppm

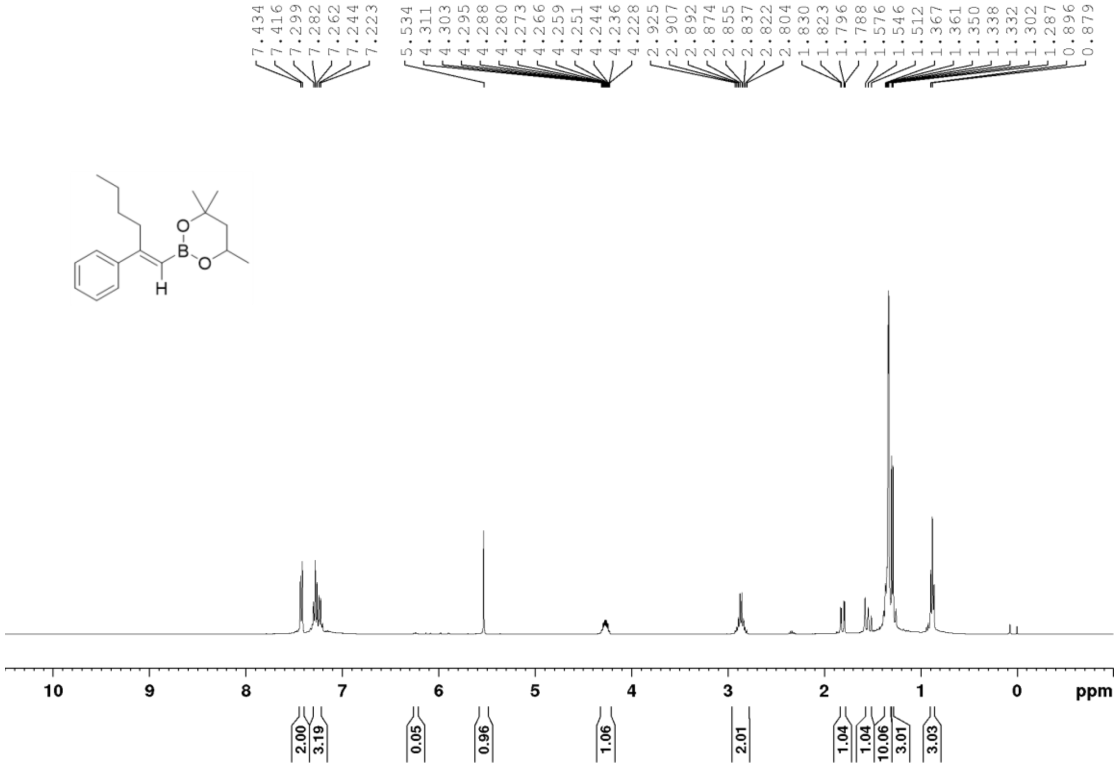
7.434
7.416
7.299
7.282
7.262
7.244
7.223
5.534
4.311
4.303
4.295
4.288
4.280
4.273
4.266
4.259
4.251
4.244
4.236
4.228
2.925
2.907
2.892
2.874
2.855
2.837
2.822
2.804
1.830
1.823
1.796
1.788
1.576
1.546
1.512
1.367
1.361
1.350
1.338
1.332
1.302
1.287
0.896
0.879
O
B
O
H
10
9
8
7
6
5
4
3
2
1
0
ppm
2.00
3.19
0.05
0.96
1.06
2.01
1.04
1.04
10.06
3.01
3.03

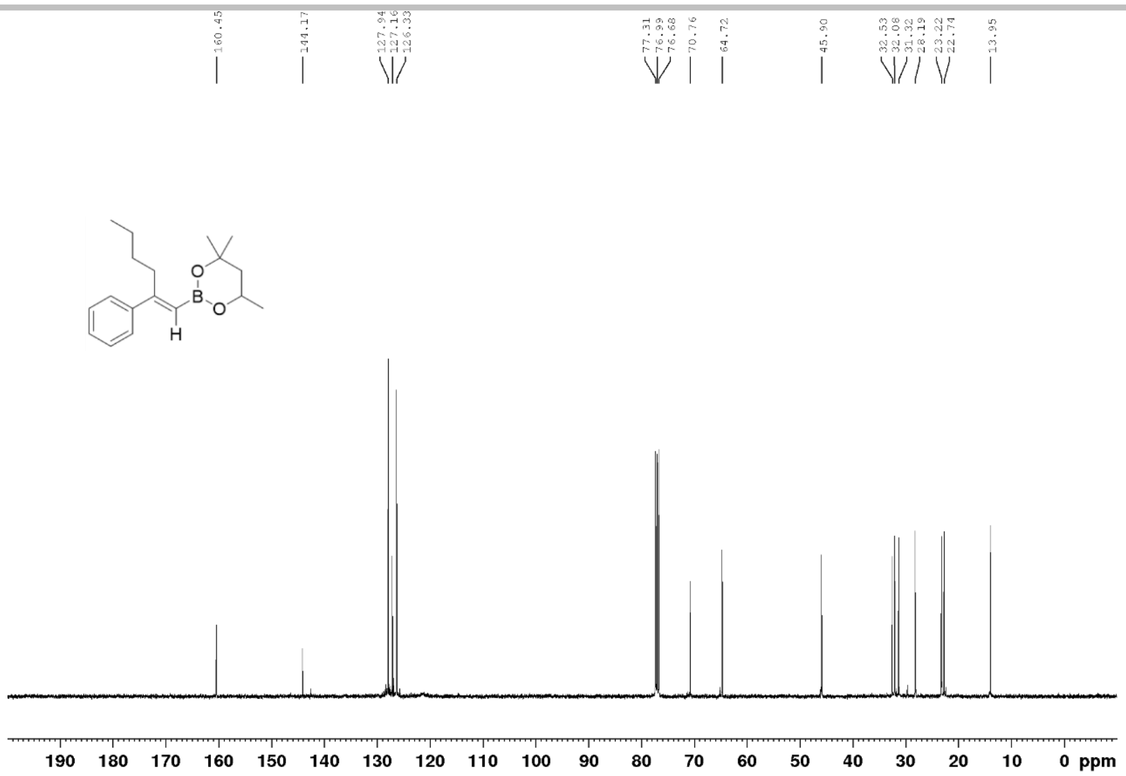
160.45
144.17
127.94
127.16
126.33
77.31
76.99
76.68
70.76
64.72
45.90
32.53
32.08
31.32
28.19
23.22
22.74
13.95
O
B
O
H
190 180 170 160 150 140 130 120 110 100 90 80 70 60 50 40 30 20 10 0 ppm

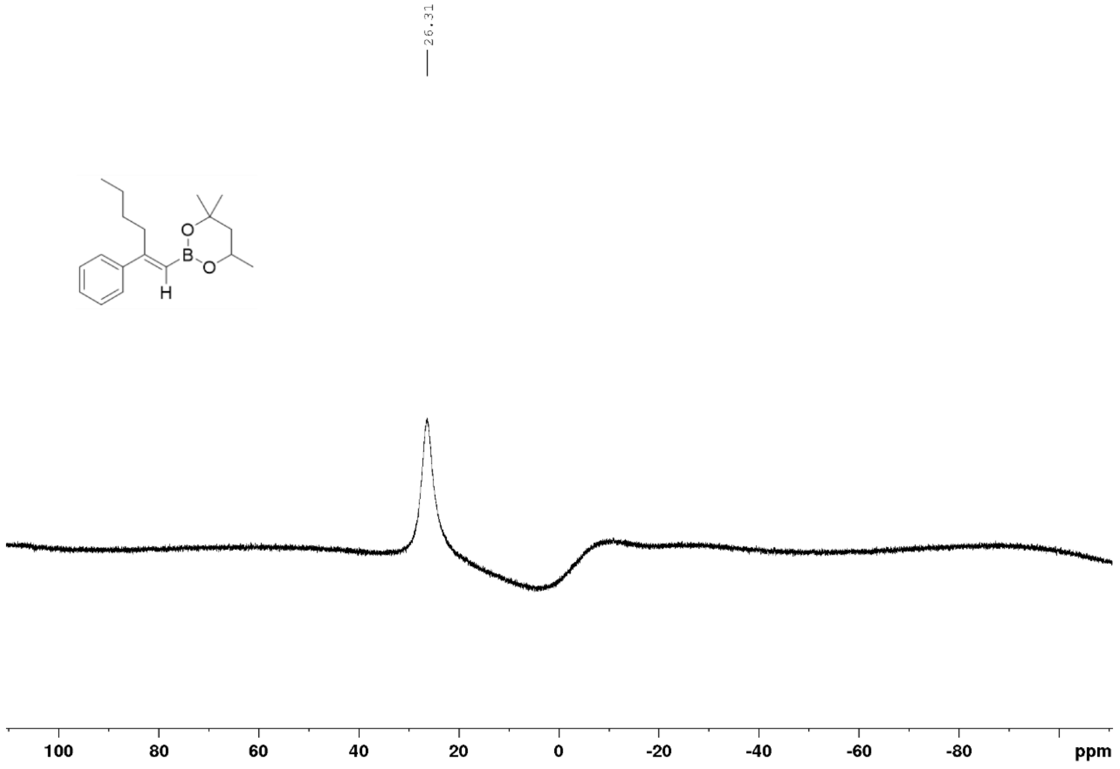
26.31
O
B
O
H
100 80 60 40 20 0 -20 -40 -60 -80 ppm

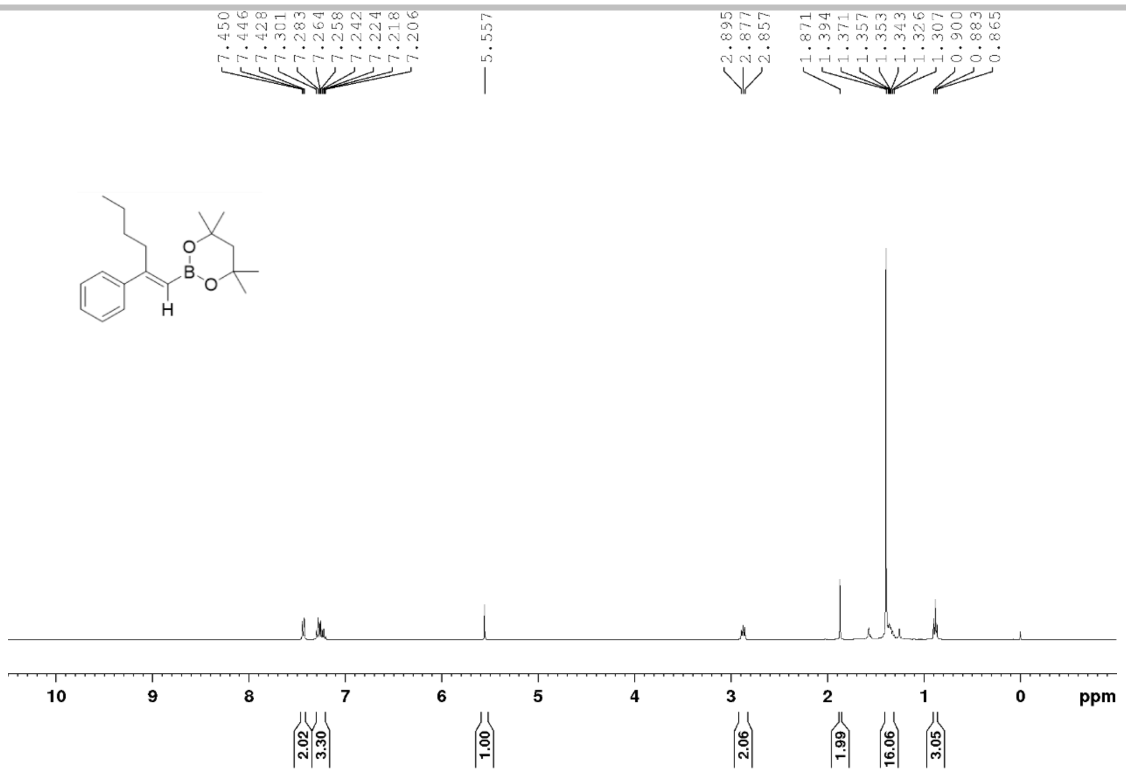
7.450
7.446
7.428
7.301
7.283
7.264
7.258
7.242
7.224
7.218
7.206
5.557
2.895
2.877
2.857
1.871
1.394
1.371
1.357
1.353
1.343
1.326
1.307
0.900
0.883
0.865
O
B
O
H
10
9
8
7
6
5
4
3
2
1
0
ppm
2.02
3.30
1.00
2.06
1.99
16.06
3.05

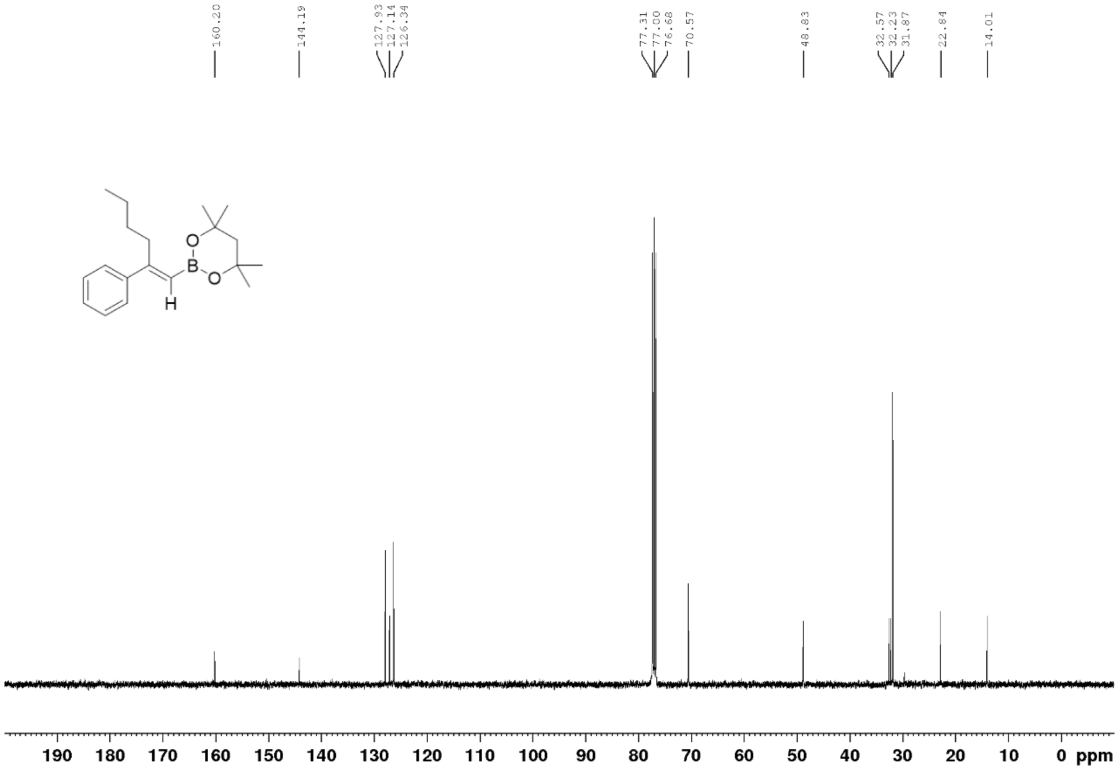
160.20
144.19
127.93
127.14
126.34
77.31
77.00
76.68
70.57
48.83
32.57
32.23
31.87
22.84
14.01
O
B
O
H
190
180
170
160
150
140
130
120
110
100
90
80
70
60
50
40
30
20
10
0 ppm

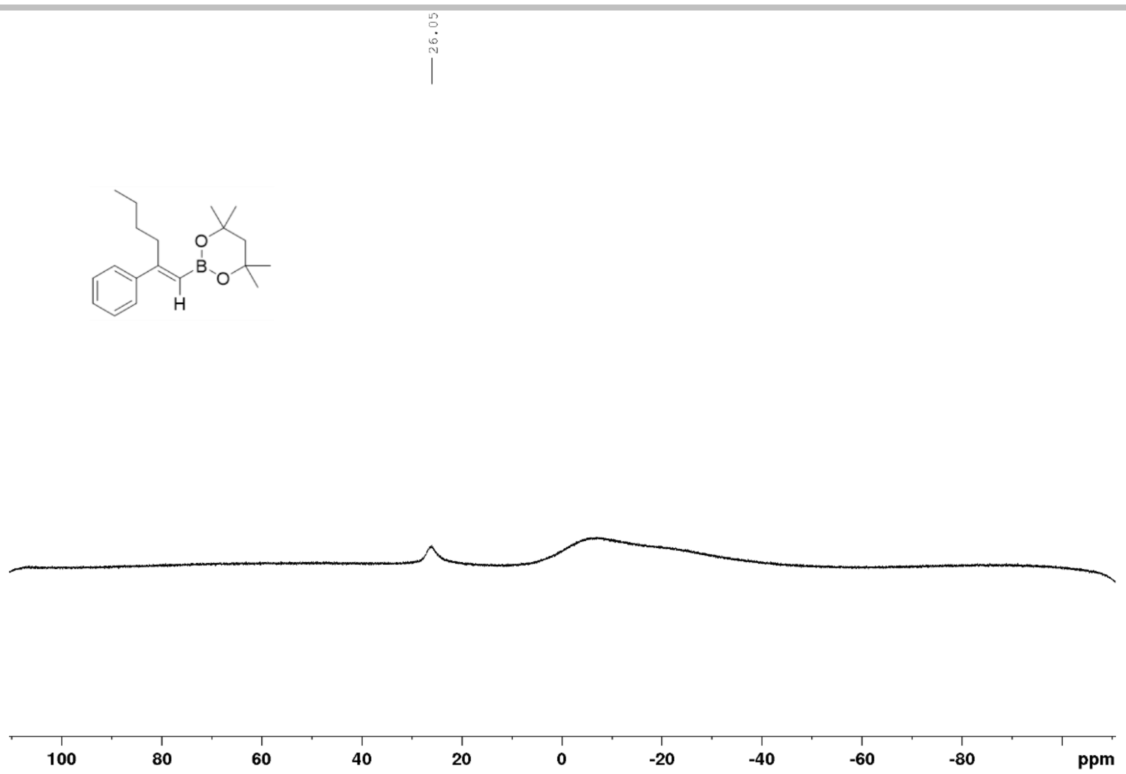
26.05
H
O
B
O
100
80
60
40
20
0
-20
-40
-60
-80
ppm

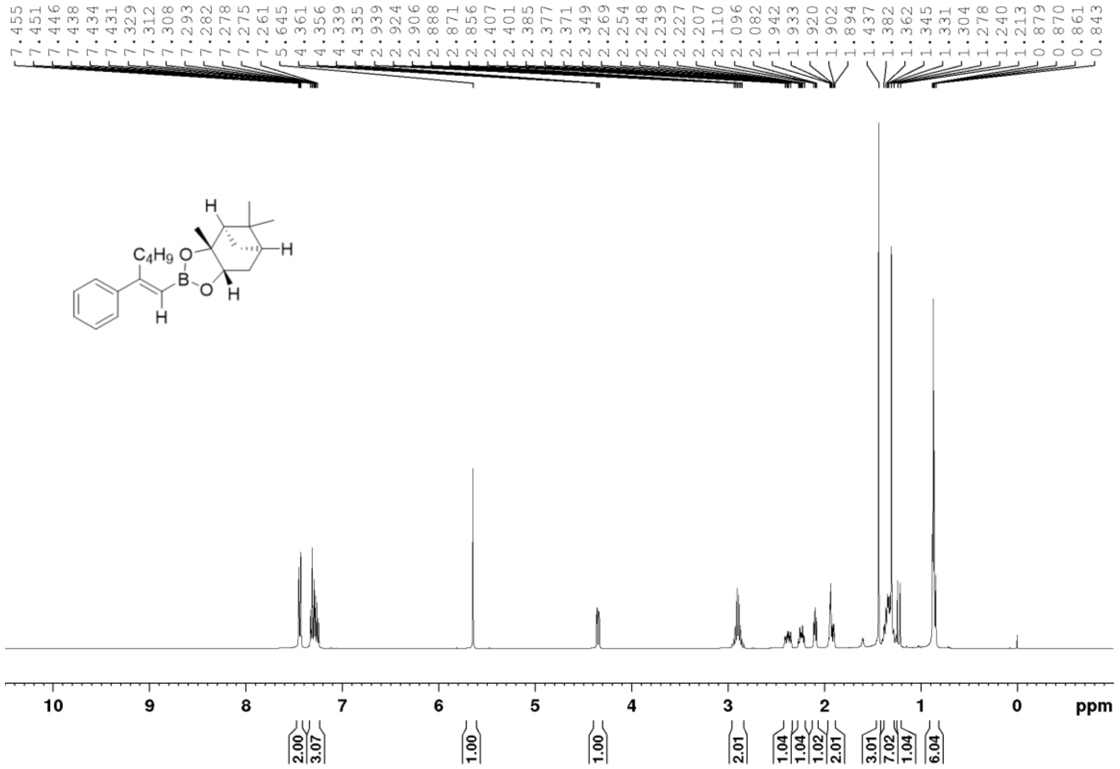
7.455
7.451
7.446
7.438
7.434
7.431
7.329
7.312
7.308
7.293
7.282
7.278
7.275
7.261
5.645
4.361
4.356
4.339
4.335
2.939
2.924
2.906
2.888
2.871
2.856
2.407
2.401
2.385
2.377
2.371
2.349
2.269
2.254
2.248
2.239
2.227
2.207
2.110
2.096
2.082
1.942
1.933
1.920
1.902
1.894
1.437
1.382
1.362
1.345
1.331
1.304
1.278
1.240
1.213
0.879
0.870
0.861
0.843
C_4H_9
O
B
O
H
H
H
H
10
9
8
7
6
5
4
3
2
1
0
ppm
2.00
3.07
1.00
1.00
2.01
1.04
1.04
1.02
2.01
3.01
7.02
1.04
6.04

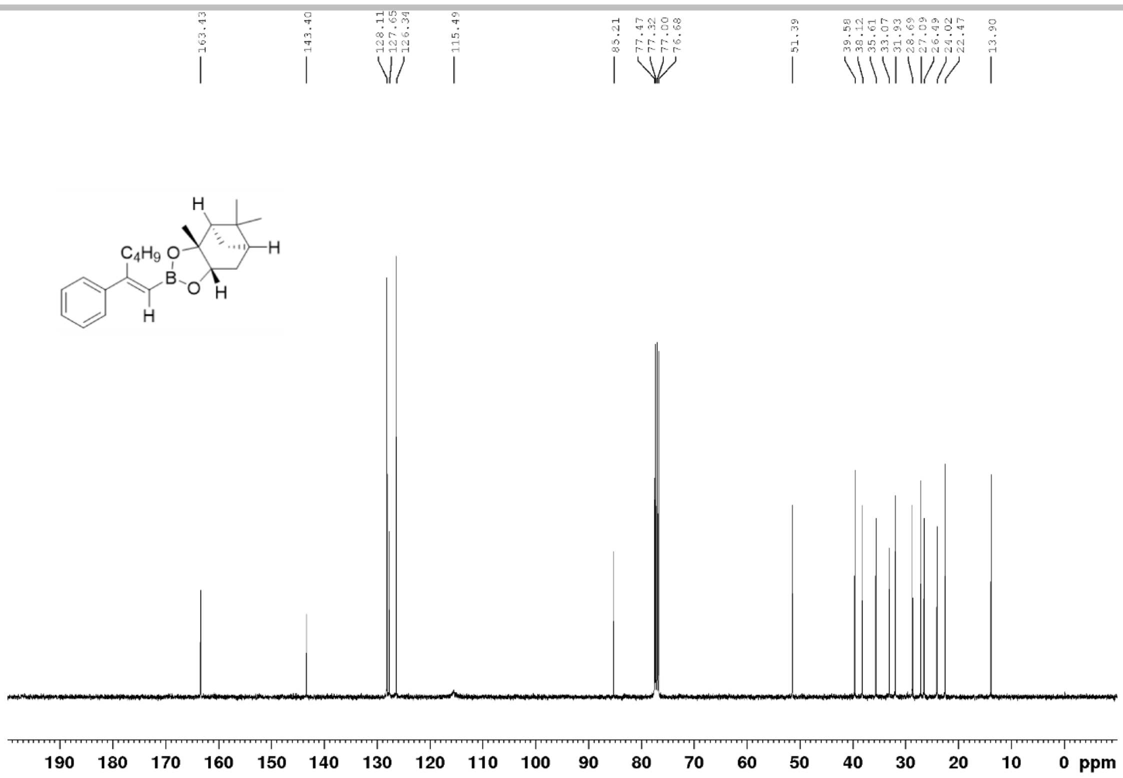
163.43
143.40
128.11
127.65
126.34
115.49
85.21
77.47
77.32
77.00
76.68
51.39
39.58
38.12
35.61
33.07
31.93
28.69
27.09
26.49
24.02
22.47
13.90
C4H9
190 180 170 160 150 140 130 120 110 100 90 80 70 60 50 40 30 20 10 0 ppm

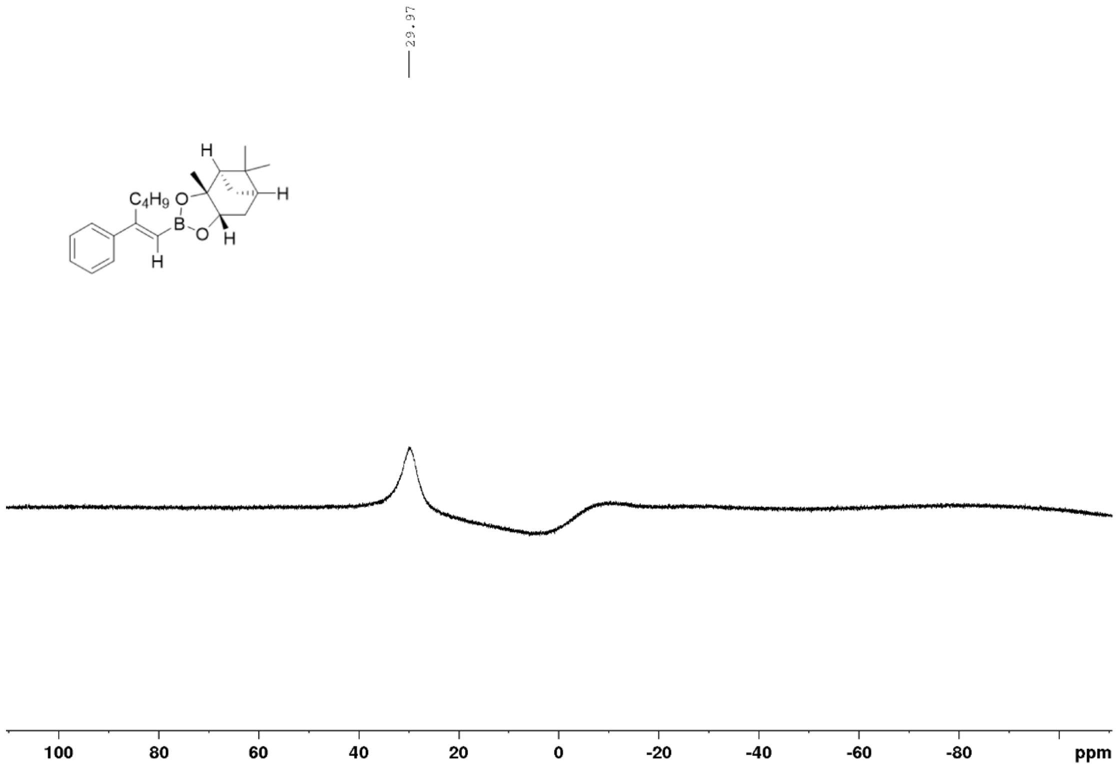
29.97
C4H9
100 80 60 40 20 0 -20 -40 -60 -80 ppm

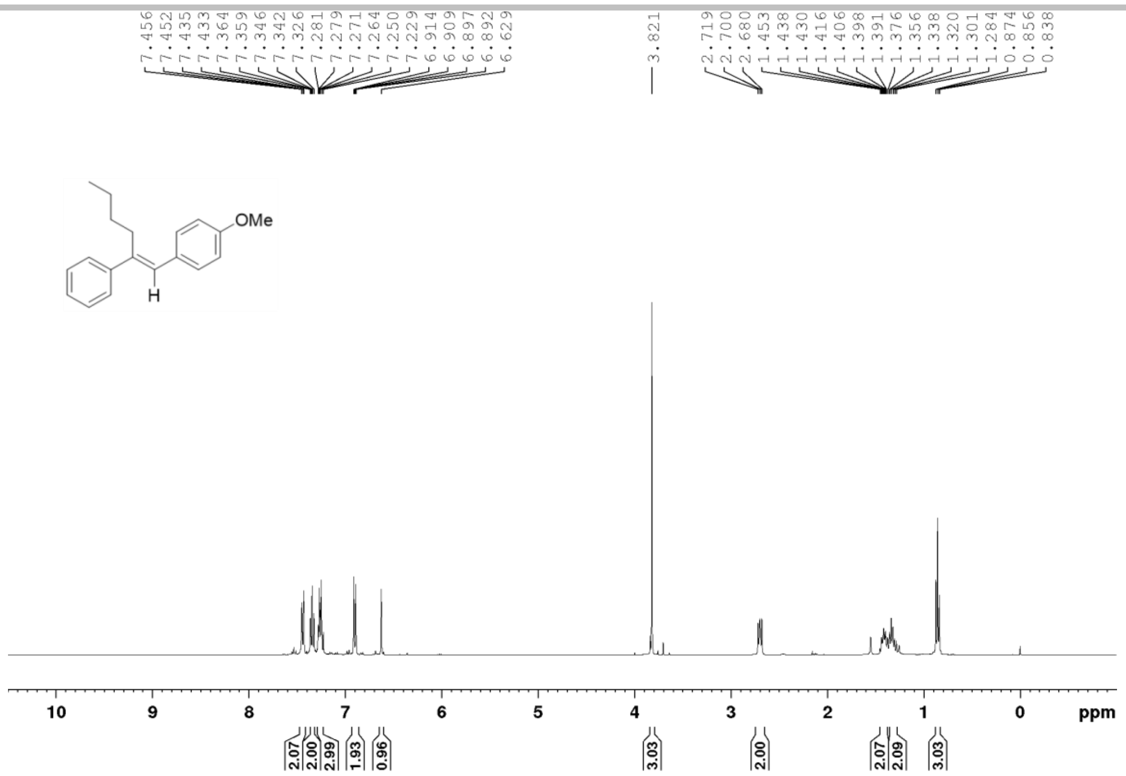
7.456 7.452 7.435 7.433 7.364 7.359 7.346 7.342 7.326 7.281 7.279 7.271 7.264 7.250 7.229 6.914 6.909 6.897 6.892 6.629
3.821
2.719 2.700 2.680 1.453 1.438 1.430 1.416 1.406 1.398 1.391 1.376 1.356 1.338 1.320 1.301 1.284 0.874 0.856 0.838
OMe
H
10 9 8 7 6 5 4 3 2 1 0 ppm
2.07 2.00 2.99 1.93 0.96 3.03 2.00 2.07 2.09 3.03

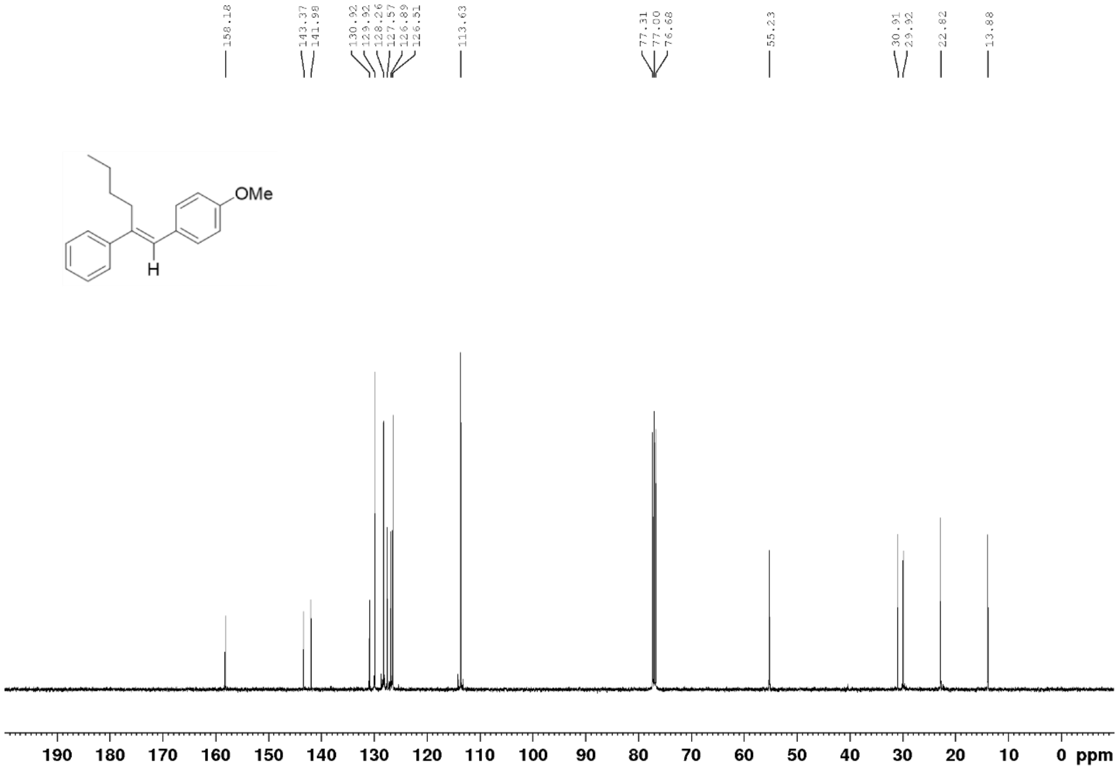
158.18
143.37 141.98
130.92 129.92 128.26 127.57 126.89 126.51
113.63
77.31 77.00 76.68
55.23
30.91 29.92
22.82
13.88
OMe
H
190 180 170 160 150 140 130 120 110 100 90 80 70 60 50 40 30 20 10 0 ppm

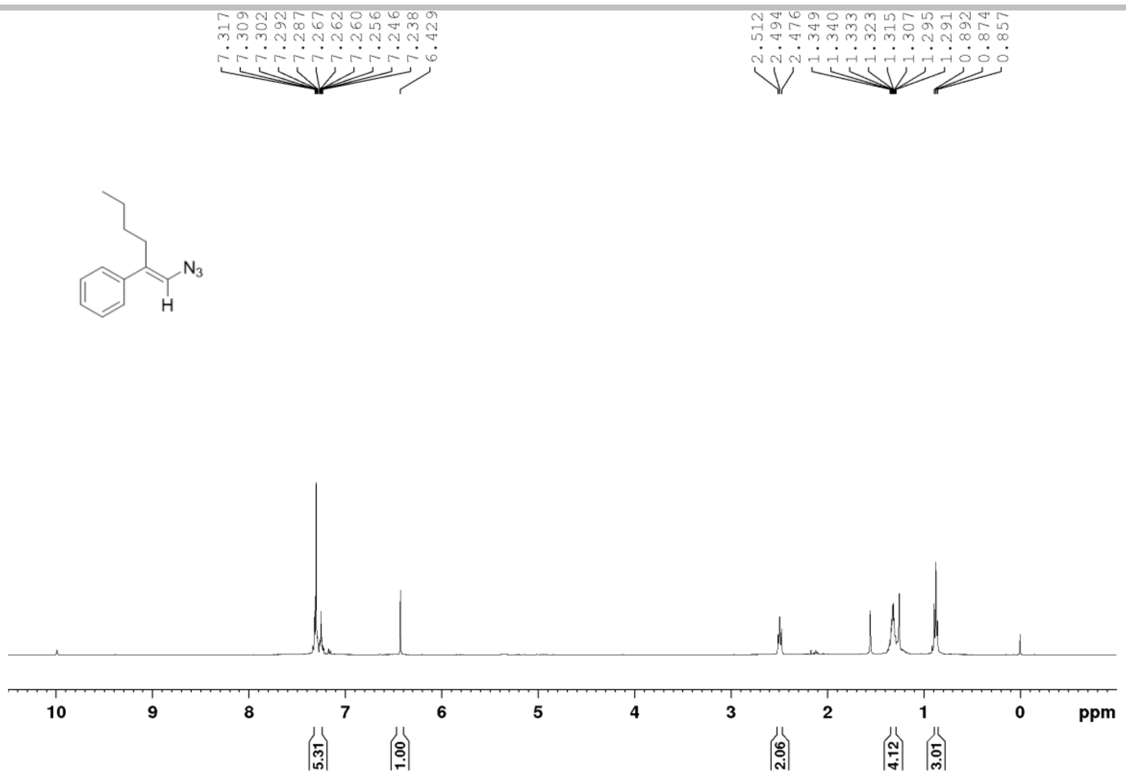
7.317
7.309
7.302
7.292
7.287
7.267
7.262
7.260
7.256
7.246
7.238
6.429
2.512
2.494
2.476
1.349
1.340
1.333
1.323
1.315
1.307
1.295
1.291
0.892
0.874
0.857
N3
H
ppm
5.31
1.00
2.06
4.12
3.01

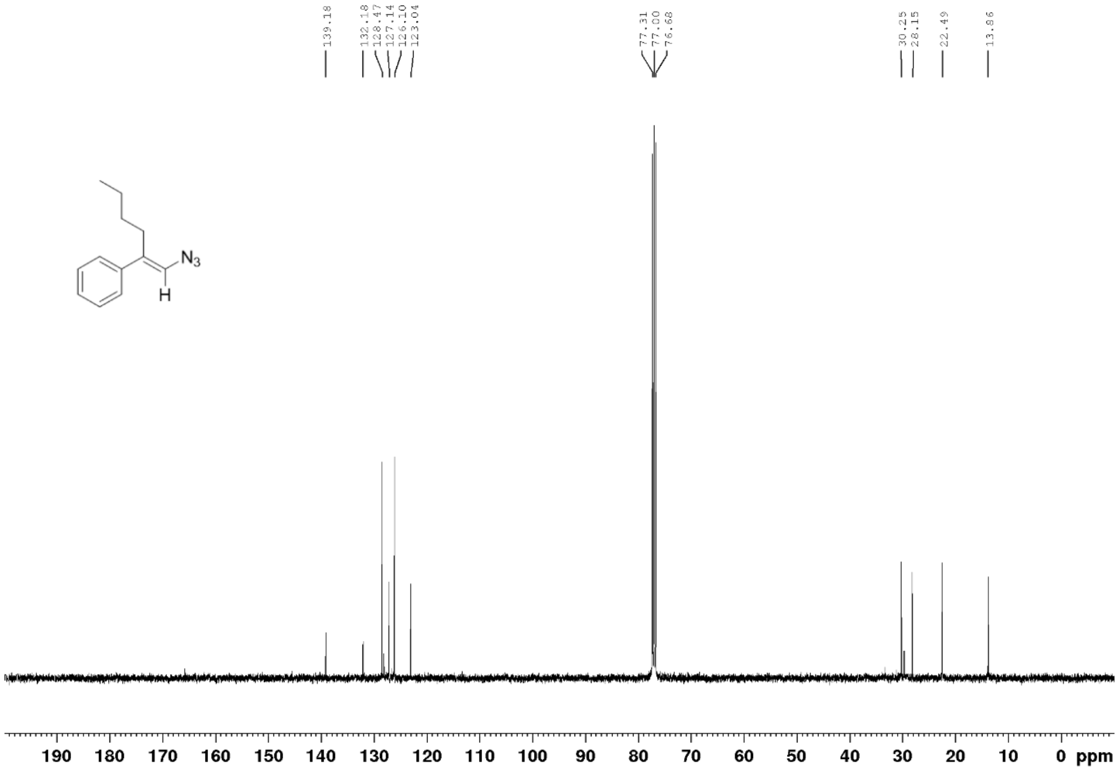
139.18
132.18
128.47
127.14
126.10
123.04
77.31
77.00
76.68
30.25
28.15
22.49
13.86
N3
H
ppm

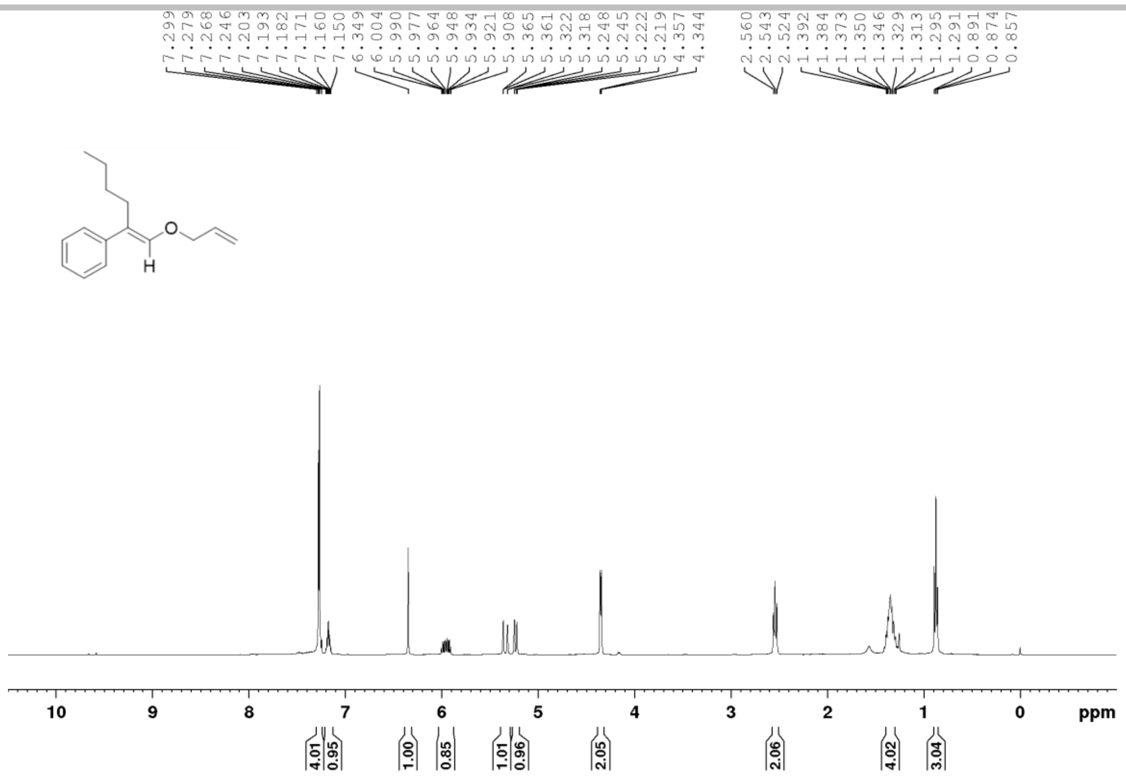
7.299
7.279
7.268
7.246
7.203
7.193
7.182
7.171
7.160
7.150
6.349
6.004
5.990
5.977
5.964
5.948
5.934
5.921
5.908
5.365
5.361
5.322
5.318
5.248
5.245
5.222
5.219
4.357
4.344
2.560
2.543
2.524
1.392
1.384
1.373
1.350
1.346
1.329
1.313
1.295
1.291
0.891
0.874
0.857
O
H
4.01
0.95
1.00
0.85
1.01
0.96
2.05
2.06
4.02
3.04
10 9 8 7 6 5 4 3 2 1 0 ppm

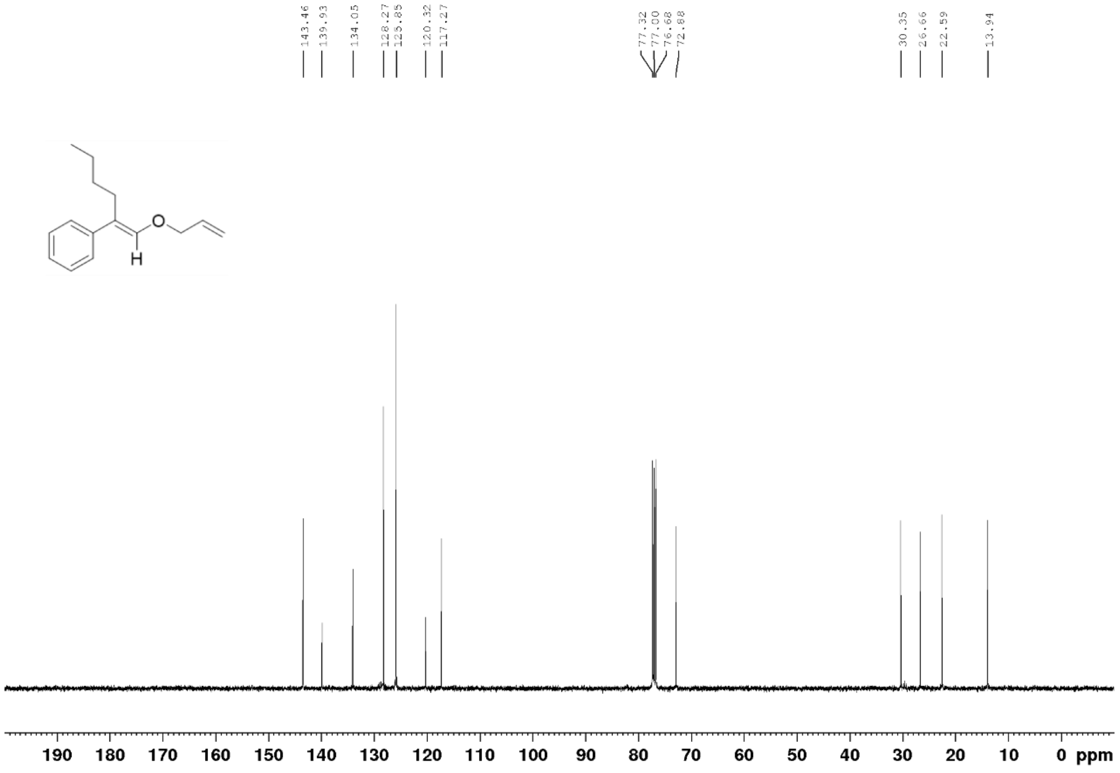
143.46
139.93
134.05
128.27
125.85
120.32
117.27
77.32
77.00
76.68
72.88
30.35
26.66
22.59
13.94
O
H
190 180 170 160 150 140 130 120 110 100 90 80 70 60 50 40 30 20 10 0 ppm

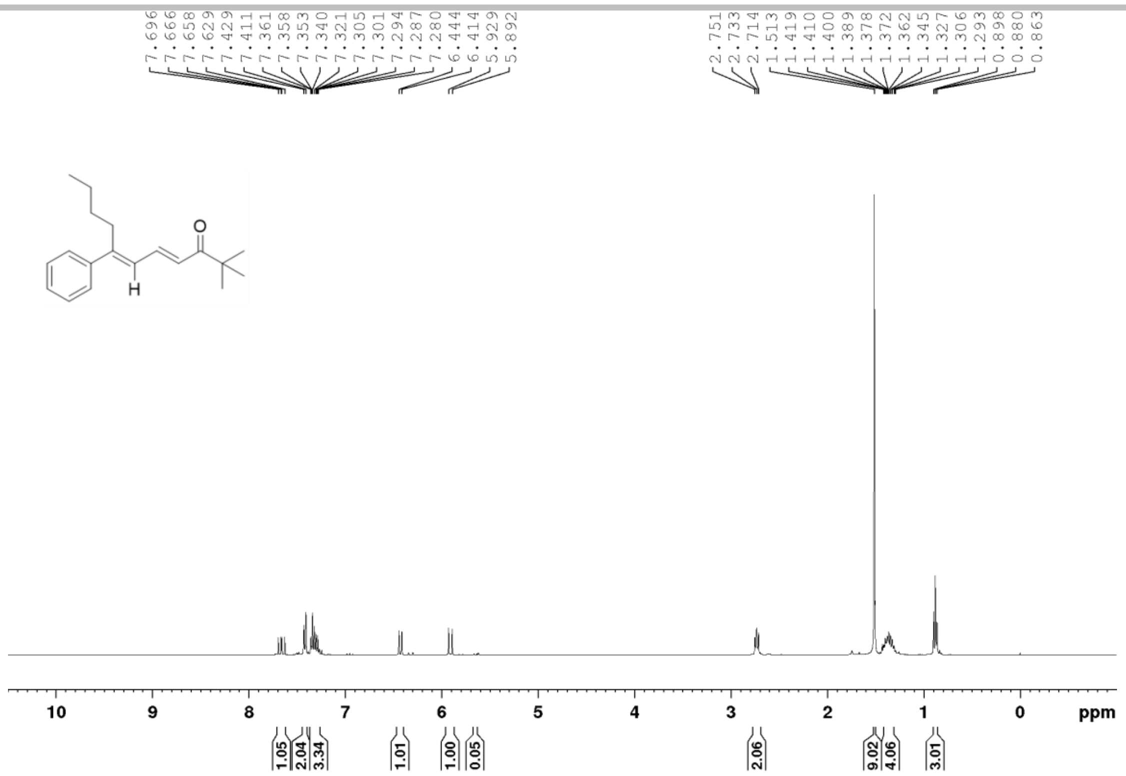
7.696 7.666 7.658 7.629 7.429 7.411 7.361 7.358 7.353 7.340 7.321 7.305 7.301 7.294 7.287 7.280 6.444 6.414 5.929 5.892
2.751 2.733 2.714 1.513 1.419 1.410 1.400 1.389 1.378 1.372 1.362 1.345 1.327 1.306 1.293 0.898 0.880 0.863
O
H
10 9 8 7 6 5 4 3 2 1 0 ppm
1.05 2.04 3.34 1.01 1.00 0.05 2.06 9.02 4.06 3.01

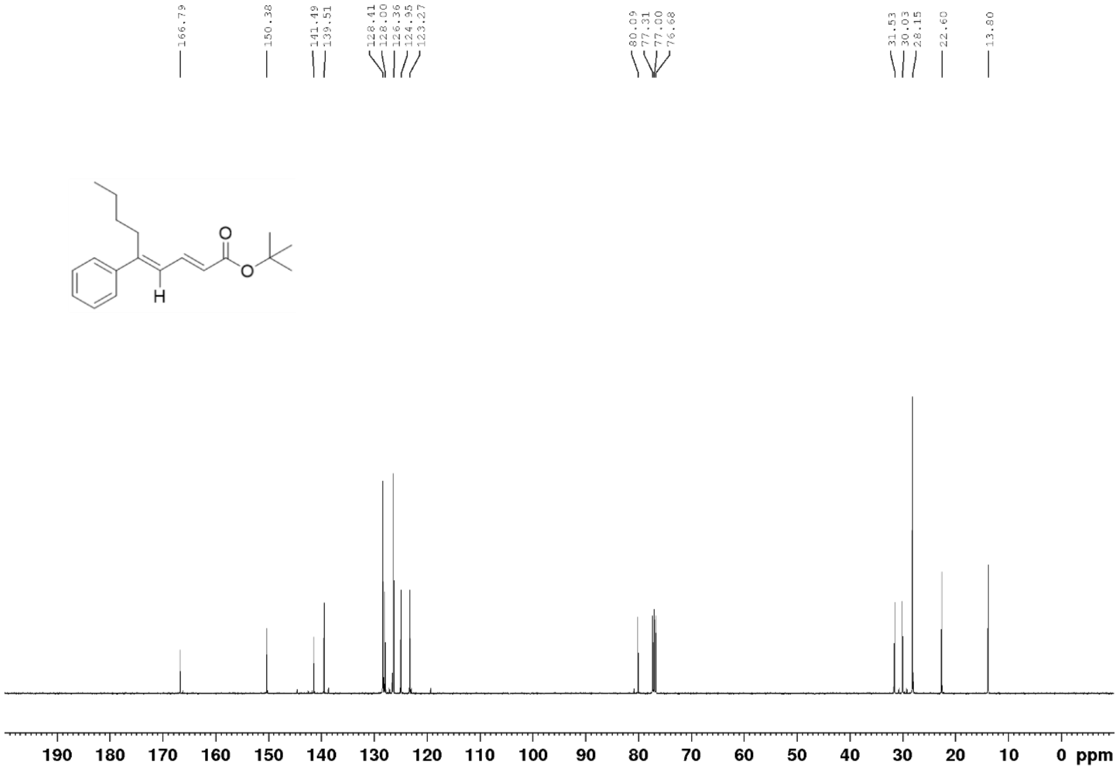
166.79 150.38 141.49 139.51 128.41 128.00 126.36 124.95 123.27 80.09 77.31 77.00 76.68 31.53 30.03 28.15 22.60 13.80
O
O
H
190 180 170 160 150 140 130 120 110 100 90 80 70 60 50 40 30 20 10 0 ppm

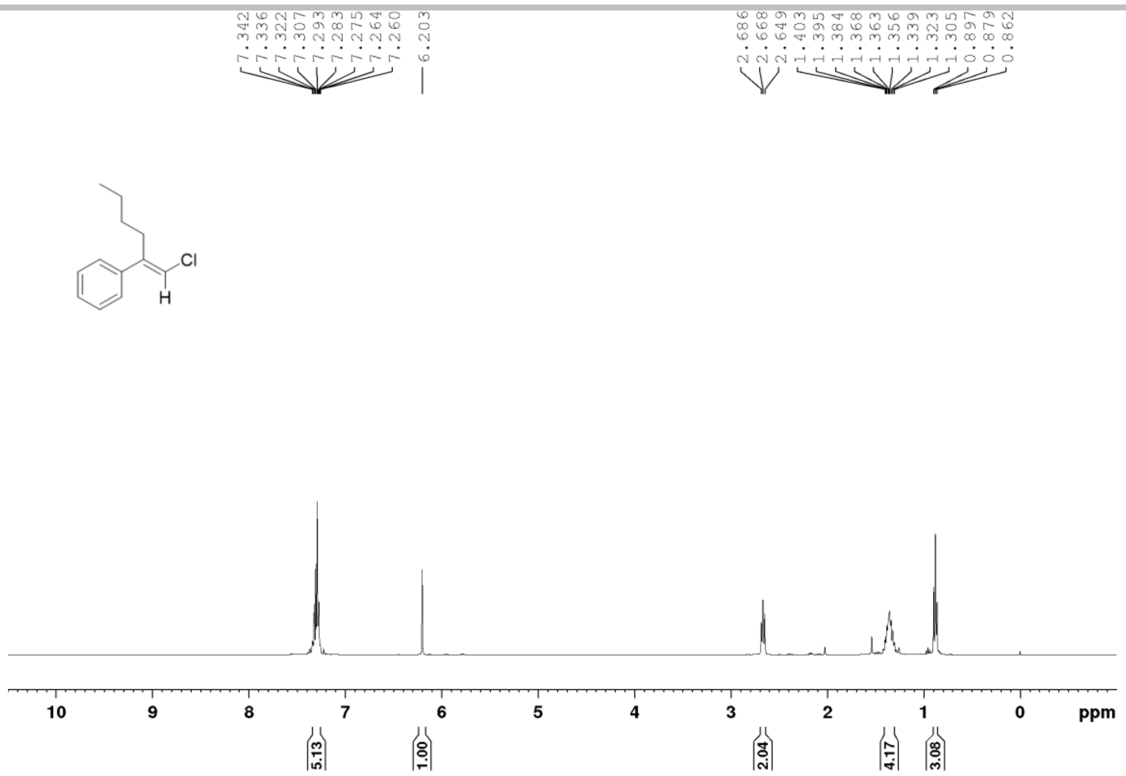
7.342
7.336
7.322
7.307
7.293
7.283
7.275
7.264
7.260
6.203
2.686
2.668
2.649
1.403
1.395
1.384
1.368
1.363
1.356
1.339
1.323
1.305
0.897
0.879
0.862
Cl
H
10 9 8 7 6 5 4 3 2 1 0 ppm
5.13
1.00
2.04
4.17
3.08

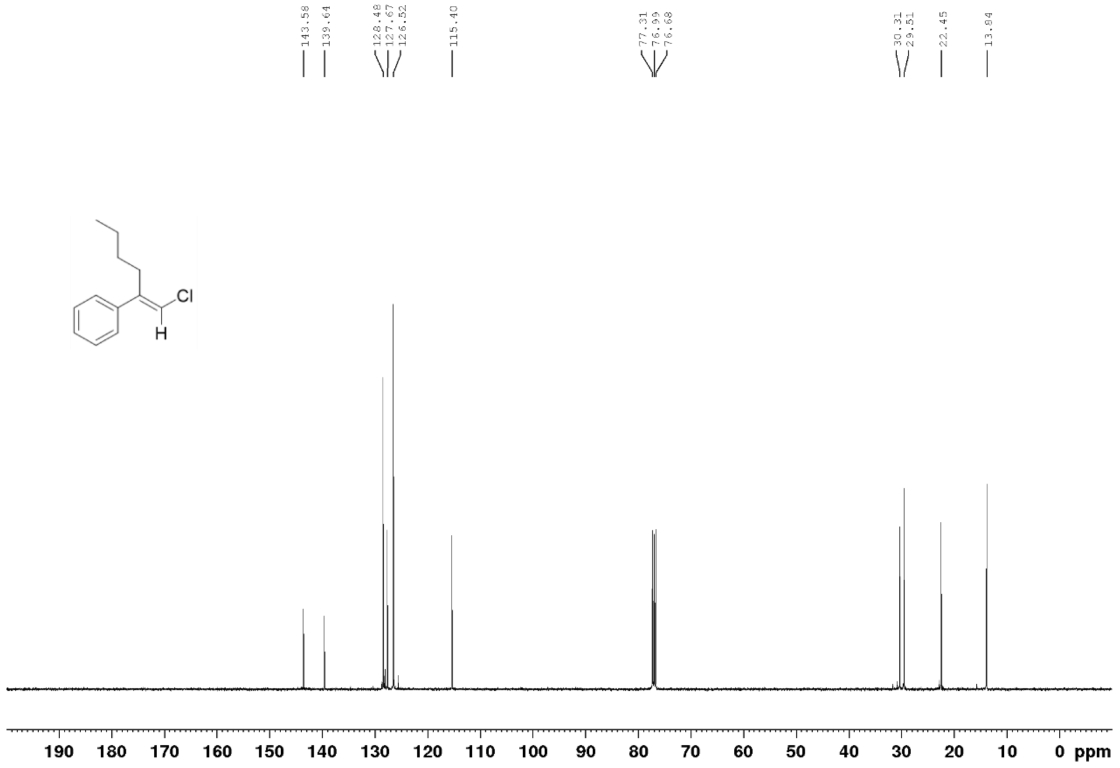
143.58
139.64
128.48
127.67
126.52
115.40
77.31
76.99
76.68
30.31
29.51
22.45
13.84
Cl
H
190 180 170 160 150 140 130 120 110 100 90 80 70 60 50 40 30 20 10 0 ppm

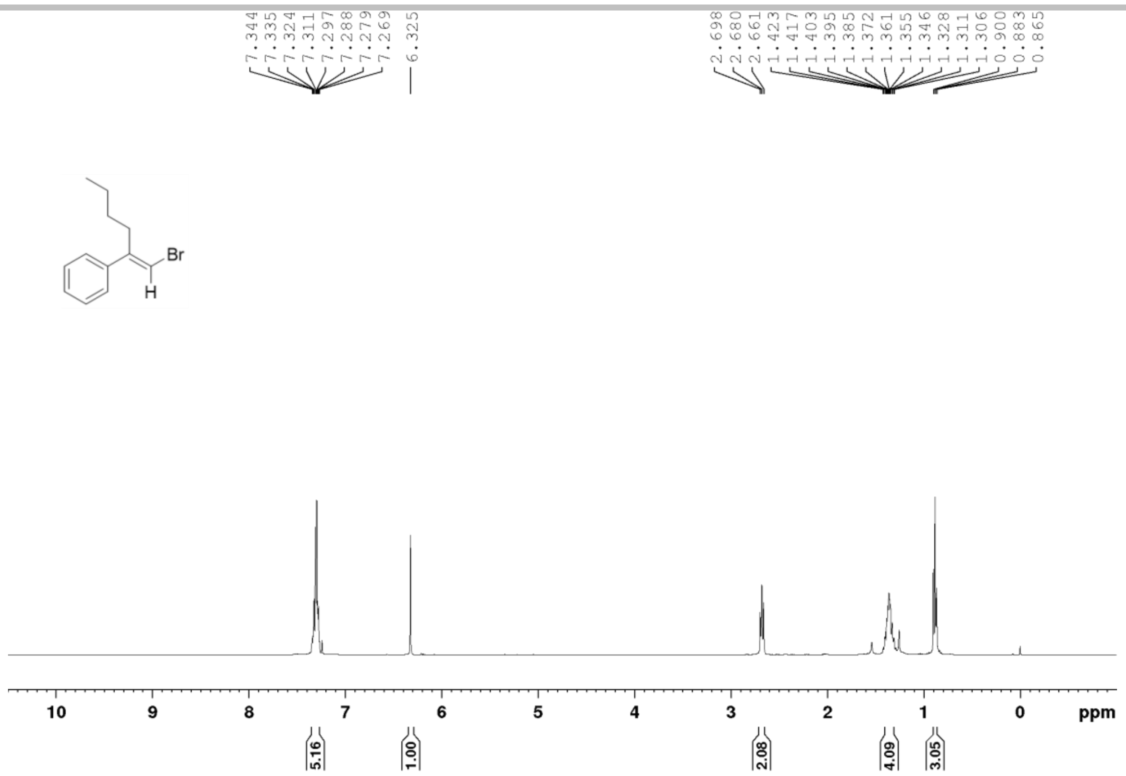
7.344
7.335
7.324
7.311
7.297
7.288
7.279
7.269
6.325
2.698
2.680
2.661
1.423
1.417
1.403
1.395
1.385
1.372
1.361
1.355
1.346
1.328
1.311
1.306
0.900
0.883
0.865
Br
H
10
9
8
7
6
5
4
3
2
1
0
ppm
5.16
1.00
2.08
4.09
3.05

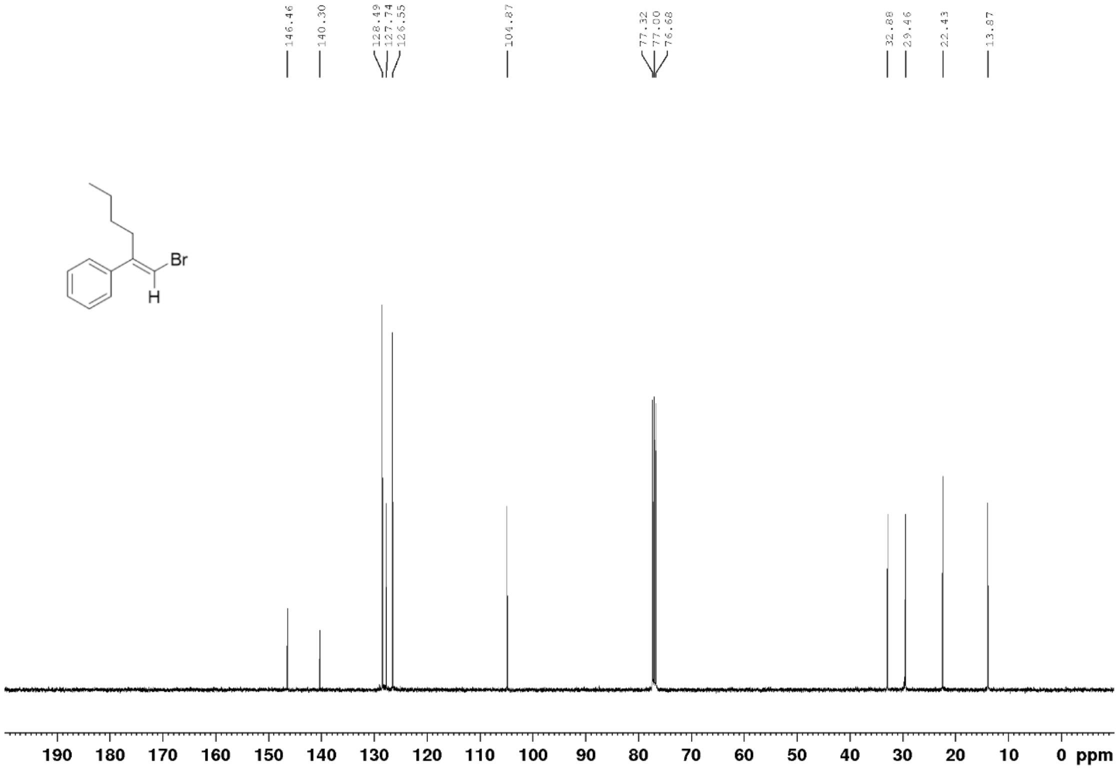
146.46
140.30
128.49
127.74
126.55
104.87
77.32
77.00
76.68
32.88
29.46
22.43
13.87
Br
H
190
180
170
160
150
140
130
120
110
100
90
80
70
60
50
40
30
20
10
0 ppm

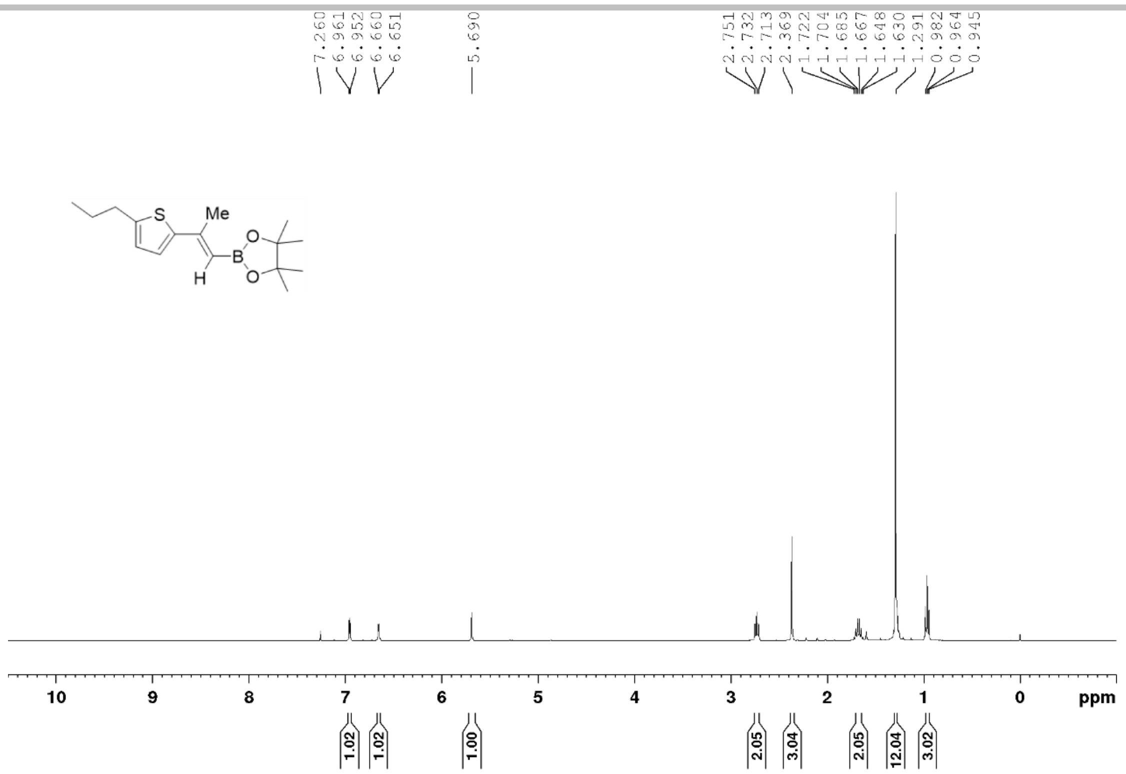
7.260
6.961
6.952
6.660
6.651
5.690
2.751
2.732
2.713
2.369
1.722
1.704
1.685
1.667
1.648
1.630
1.291
0.982
0.964
0.945
S
Me
B
O
O
H
10 9 8 7 6 5 4 3 2 1 0 ppm
1.02
1.02
1.00
2.05
3.04
2.05
12.04
3.02

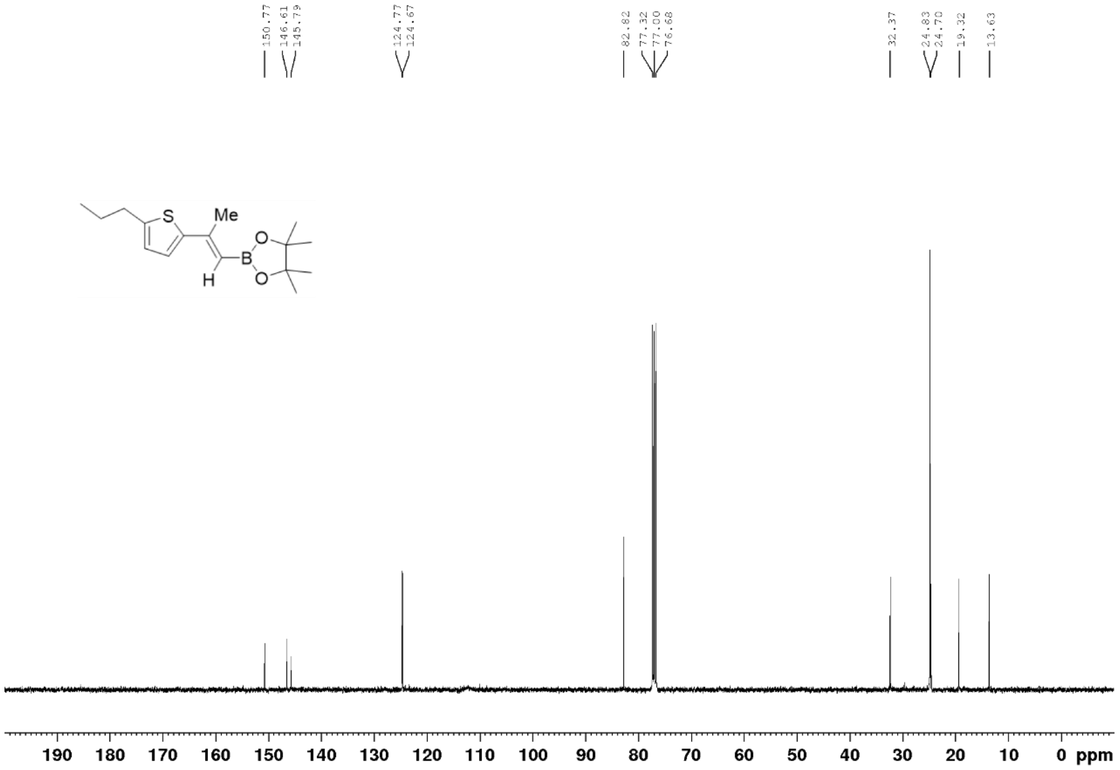
150.77
146.61
145.79
124.77
124.67
82.82
77.32
77.00
76.68
32.37
24.83
24.70
19.32
13.63
S
Me
B
O
O
H
190 180 170 160 150 140 130 120 110 100 90 80 70 60 50 40 30 20 10 0 ppm

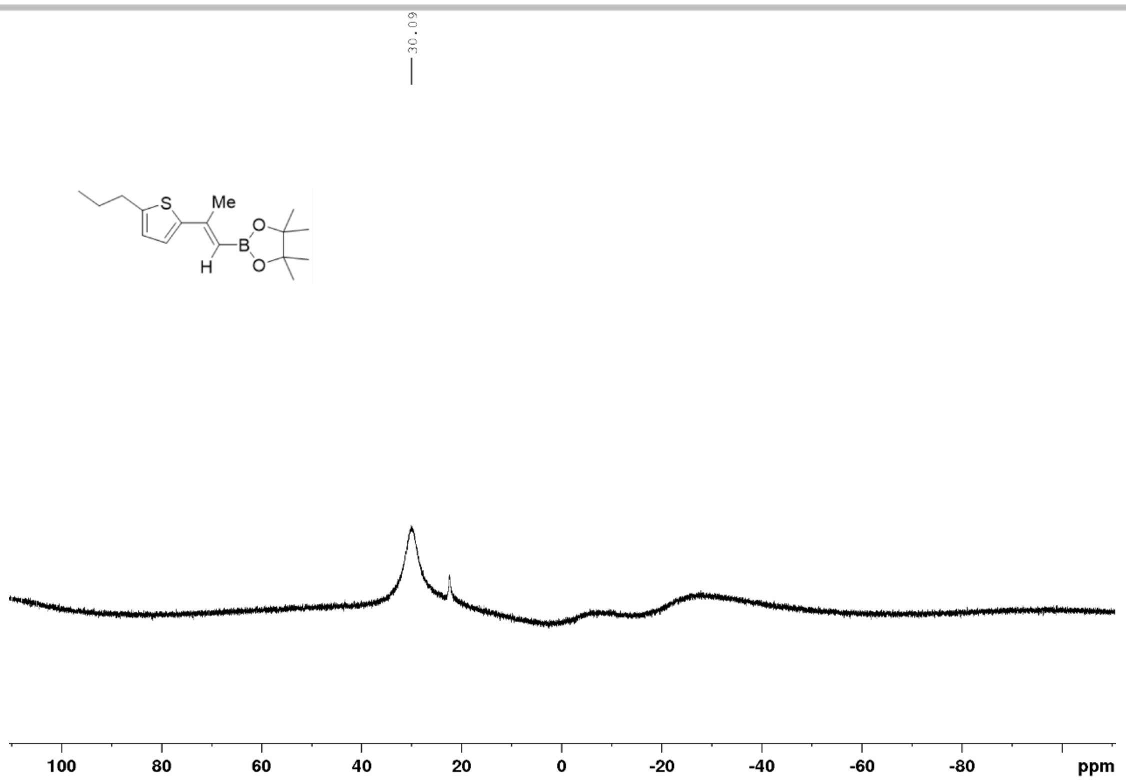
30.09
S
Me
O
B
O
H
100
80
60
40
20
0
-20
-40
-60
-80
ppm

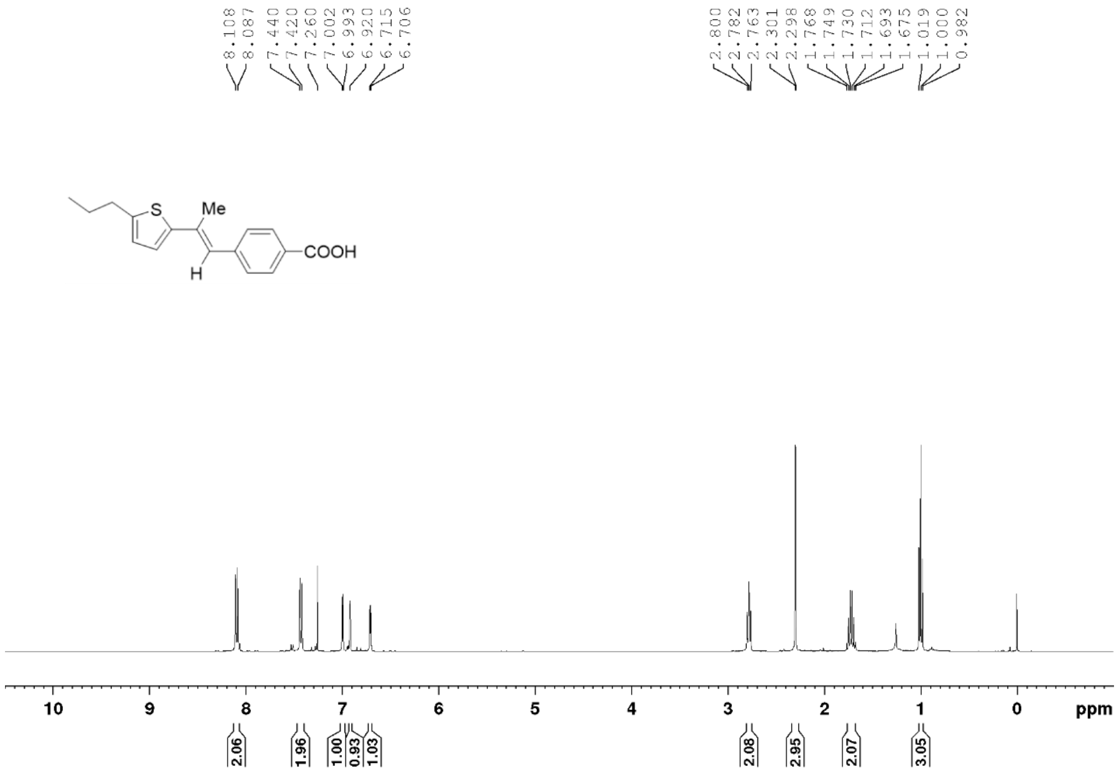
8.108
8.087
7.440
7.420
7.260
7.002
6.993
6.920
6.715
6.706
2.800
2.782
2.763
2.301
2.298
1.768
1.749
1.730
1.712
1.693
1.675
1.019
1.000
0.982
S
Me
COOH
H
10
9
8
7
6
5
4
3
2
1
0
ppm
2.06
1.96
1.00
0.93
1.03
2.08
2.95
2.07
3.05

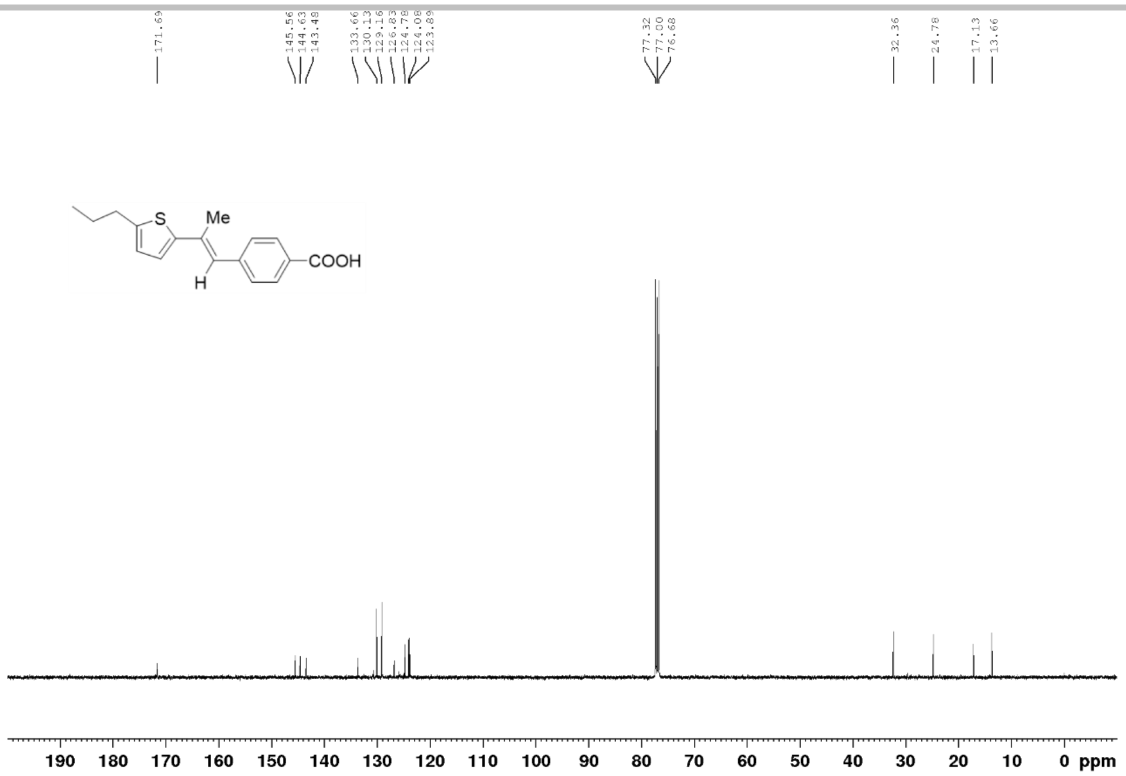
171.69
145.56
144.63
143.48
133.66
130.13
129.16
126.83
124.78
124.08
123.89
77.32
77.00
76.68
32.36
24.70
17.13
13.66
Me
S
H
COOH
190 180 170 160 150 140 130 120 110 100 90 80 70 60 50 40 30 20 10 0 ppm

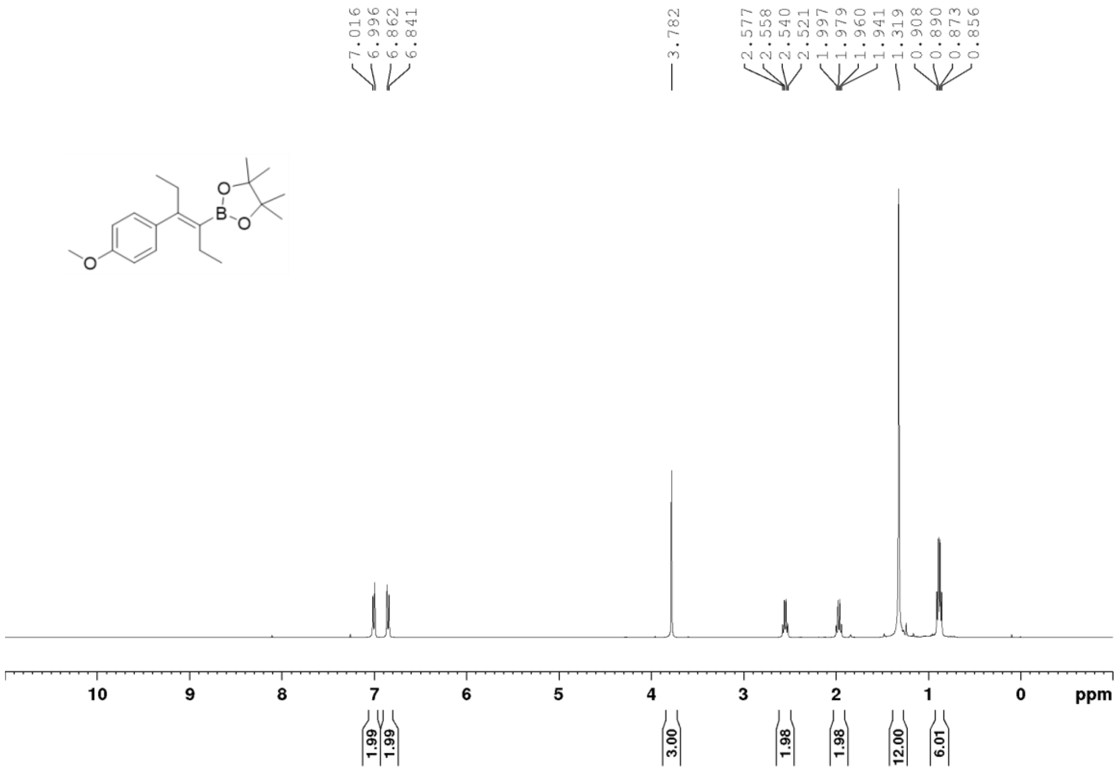
7.016
6.996
6.862
6.841
3.782
2.577
2.558
2.540
2.521
1.997
1.979
1.960
1.941
1.319
0.908
0.890
0.873
0.856
O
B
10 9 8 7 6 5 4 3 2 1 0 ppm
1.99
1.99
3.00
1.98
1.98
12.00
6.01

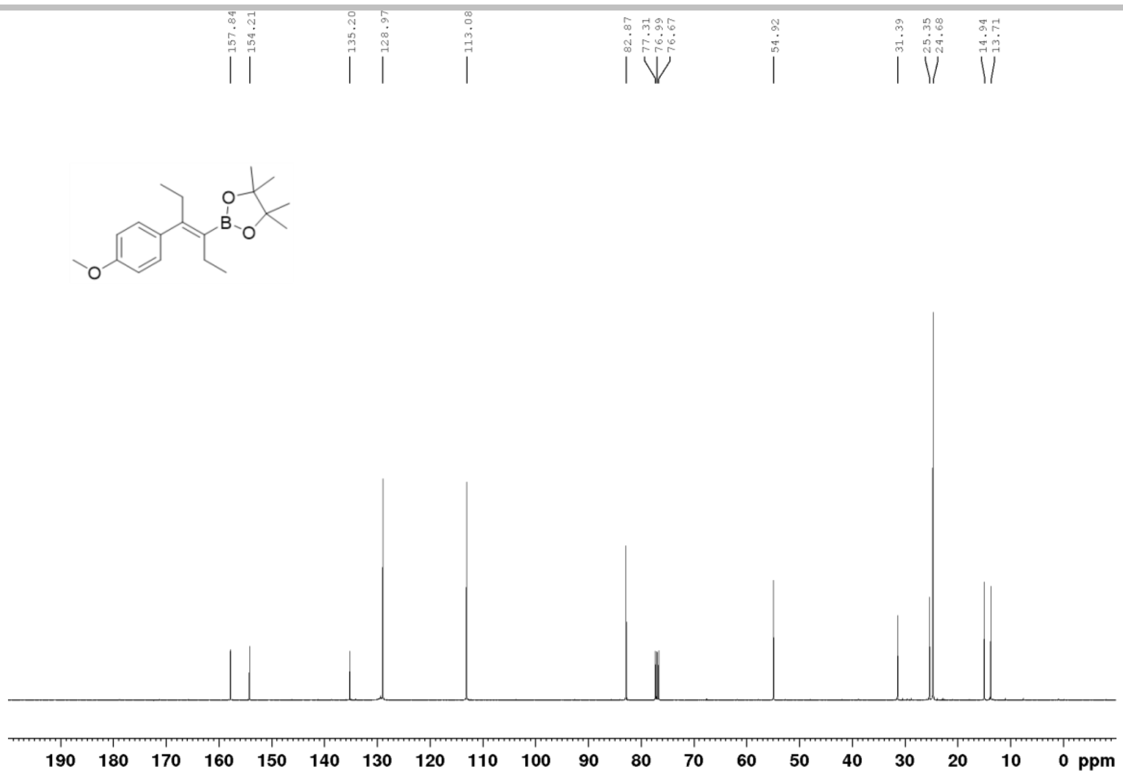
157.84
154.21
135.20
128.97
113.08
82.87
77.31
76.99
76.67
54.92
31.39
25.35
24.68
14.94
13.71
190 180 170 160 150 140 130 120 110 100 90 80 70 60 50 40 30 20 10 0 ppm

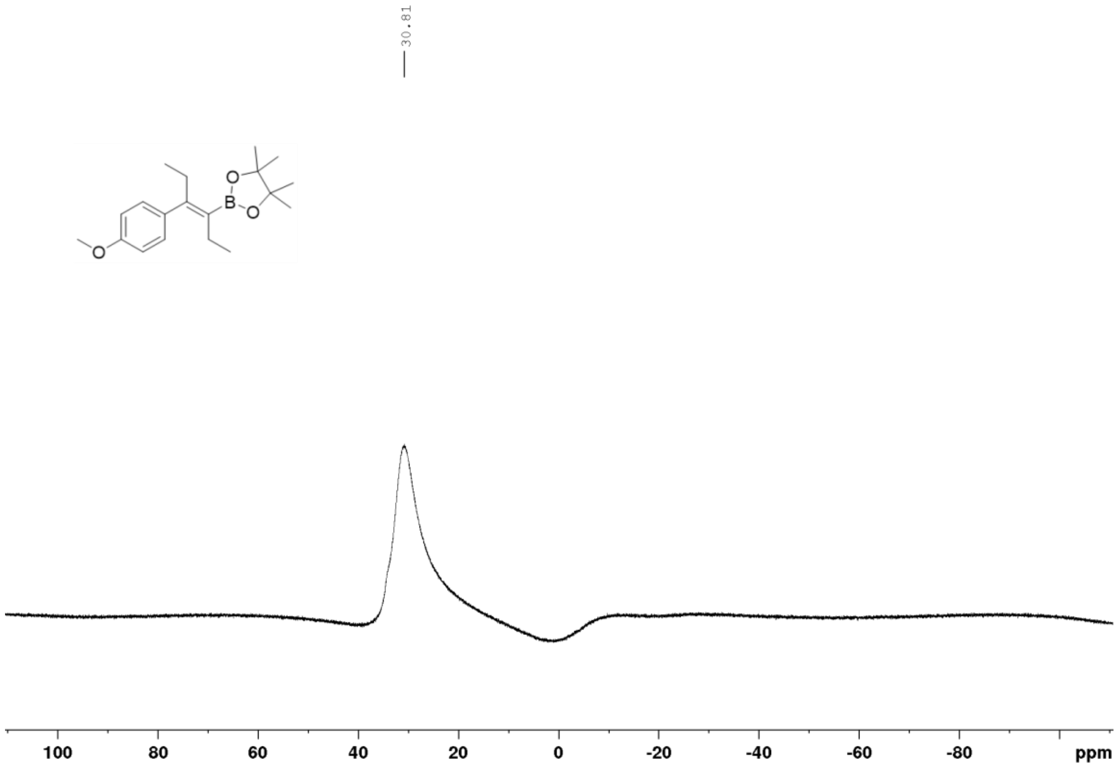
30.81
100 80 60 40 20 0 -20 -40 -60 -80 ppm

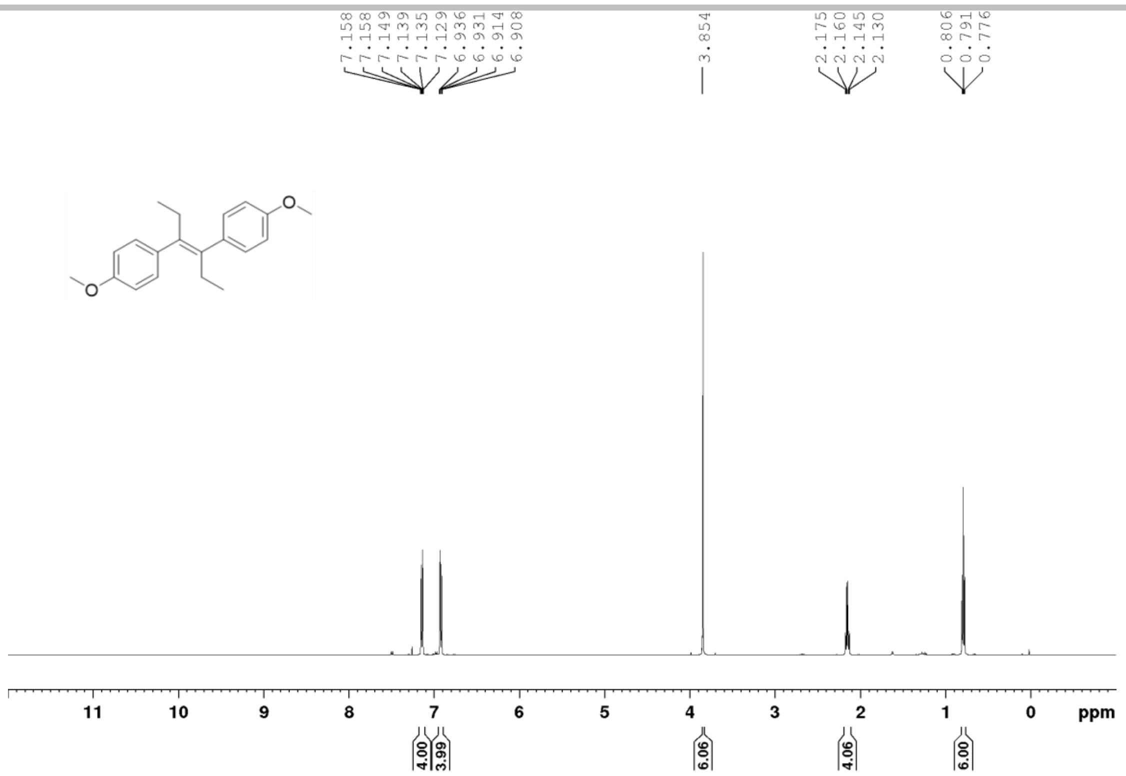
7.158
7.158
7.149
7.139
7.135
7.129
6.936
6.931
6.914
6.908
3.854
2.175
2.160
2.145
2.130
0.806
0.791
0.776
11 10 9 8 7 6 5 4 3 2 1 0 ppm
4.00
3.99
6.06
4.06
6.00

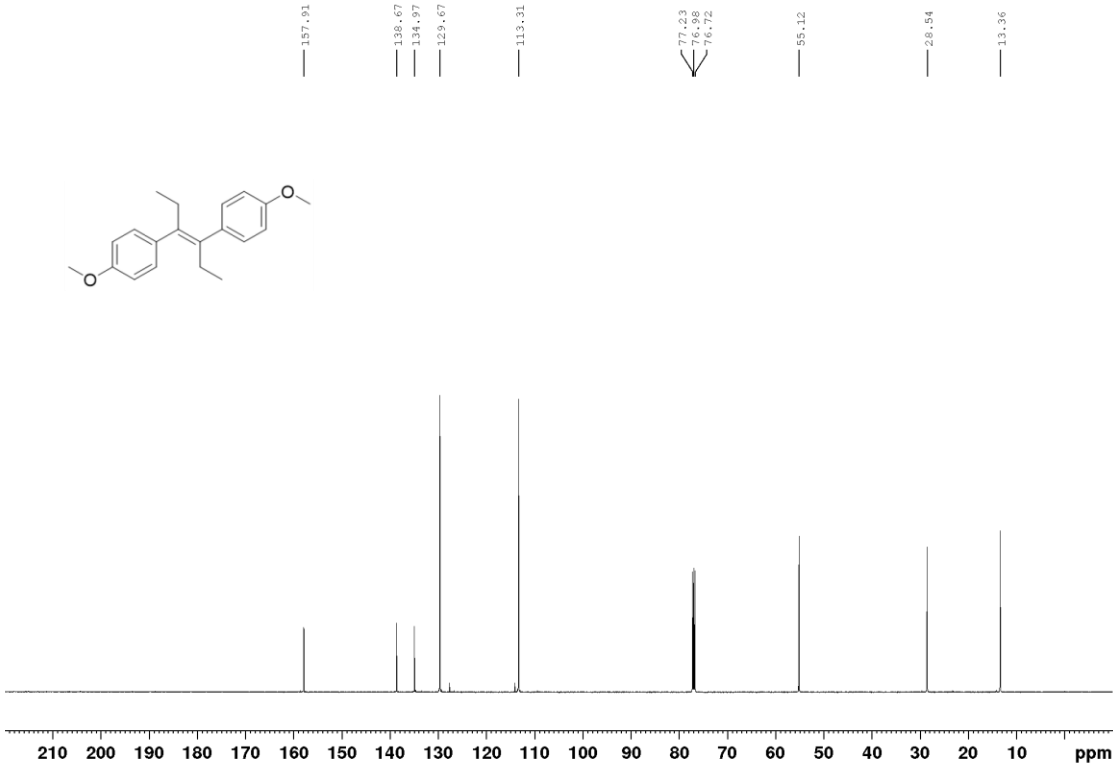
157.91
138.67
134.97
129.67
113.31
77.23
76.98
76.72
55.12
28.54
13.36
210 200 190 180 170 160 150 140 130 120 110 100 90 80 70 60 50 40 30 20 10 ppm

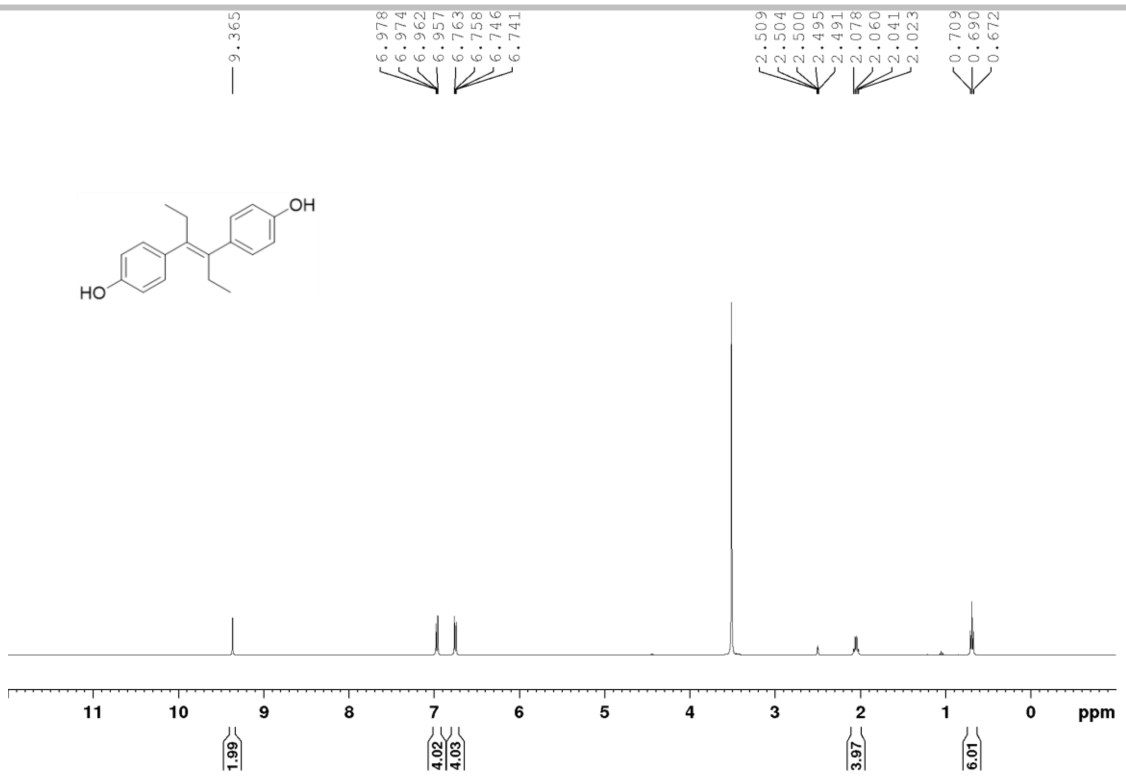

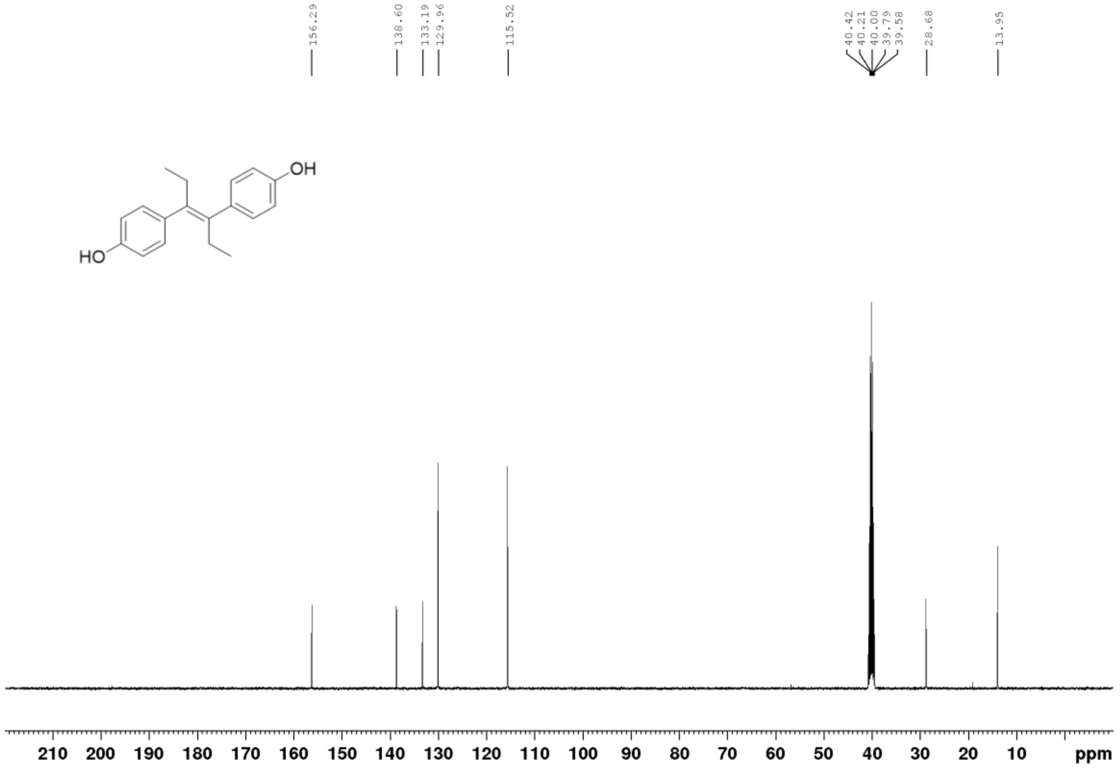

4. References

[1] N. Aoyagi, Y. Furusho, Y. Sei, T. Endo, *Tetrahedron*, **2013**, *69*, 5476−5480.

[2] O. Santoro, A. Collado, A. M. Slawin, S. P. Nolan, C. S. Cazin, *Chem. Commun.*, **2013**, *49*, 10483−10485.

[3] B. Alici, E. Çetinkaya, B. A. Çetinkaya, *Heterocycles*, **1997**, *1*, 29−36.

[4] J. Buendia, B. Darses, P. Dauban, *Angew. Chem. Int. Ed.*, **2015**, *54*, 5697−5701.
[5] B. M. Trost, M. T. Rudd, *Org. Lett*., **2003**, *5*, 4599−4602.
[6] R. Alfaro, A. Parra, J. Alemán, J. L. Garcia Ruano, M. Tortosa, *J. Am. Chem. Soc.,* **2012**, *134*, 15165–15168;
[7] E. Tayama, S. Sugai, *Tetrahedron Lett.,* **2007**, *48*, 6163.
[8] C. Maaliki, Y. Chevalier, E. Thiery, J. Thibonnet. *Tetrahedron Lett.,* **2016**, 57, 3358–3362.
[9] Y.-D. Zhang, X.-Y. Li, Q.-K. Mo, W.-B. Shi, J.-B. Zhao, S.-F. *Angew. Chem. Int. Ed.*, **2022**, *61*, e202208473.
[10] J. Altarejos, D. Sucunza, J. J. Vaquero, J. Carreras, *Eur. J. Org. Chem.*, **2020**, 3024−3029.
[11] Ž. Hodnik, T. Tomašič, D. Smodiš, C. D'Amore, L. P. Mašič, S. Fiorucci, D. Kikelj, *Eur. J. Med. Chem.*, **2015**, *103*, 551–562.
[12] Gaussian 16, Revision A.03, M. J. Frisch, G. W. Trucks, H. B. Schlegel, G. E. Scuseria, M. A. Robb, J. R. Cheeseman, G. Scalmani, V. Barone, G. A. Petersson, H. Nakatsuji, X. Li, M. Caricato, A. V. Marenich, J. Bloino, B. G. Janesko, R. Gomperts, B. Mennucci, H. P. Hratchian, J. V. Ortiz, A. F. Izmaylov, J. L. Sonnenberg, D. Williams-Young, F. Ding, F. Lipparini, F. Egidi, J. Goings, B. Peng, A. Petrone, T. Henderson, D. Ranasinghe, V. G. Zakrzewski, J. Gao, N. Rega, G. Zheng, W. Liang, M. Hada, M. Ehara, K. Toyota, R. Fukuda, J. Hasegawa, M. Ishida, T. Nakajima, Y. Honda, O. Kitao, H. Nakai, T. Vreven, K. Throssell, J. A. Montgomery, Jr., J. E. Peralta, F. Ogliaro, M. J. Bearpark, J. J. Heyd, E. N. Brothers, K. N. Kudin, V. N. Staroverov, T. A. Keith, R. Kobayashi, J. Normand, K. Raghavachari, A. P. Rendell, J. C. Burant, S. S. Iyengar, J. Tomasi, M. Cossi, J. M. Millam, M. Klene, C. Adamo, R. Cammi, J. W. Ochterski, R. L. Martin, K. Morokuma, O. Farkas, J. B. Foresman, and D. J. Fox, Gaussian, Inc., Wallingford CT, 2016.
[13] A.D. Becke, *J. Chem. Phys.* **1993**, *98*, 1372-1377.
[14] P.J. Stephens, F.J. Devlin, C.F. Chabalowski, M.J. Frisch, *J. Chem. Phys.* **1994**, *98*, 11623−11627.
[15] W.J. Hehre, R. Ditchfield, J.A. Pople, *J. Chem. Phys.* **1972**, *56*, 2257−2261.
[16] S. Grimme, J. Antony, S. Ehrlich, H. Krieg, *J. Chem. Phys.* **2010**, *132*, 154104.
[17] T. Lu, F.W. Chen, *J. Comput. Chem.* **2012**, *33*, 580−592.
[18] W. Humphrey, D, Andrew, K. Schulten, *J. Mol. Graph.* **1996**, *14*. 33−38.

5. Author Contributions

Y.-M. Pan and H.-T. Tang directed the project. H.-T. Tang and J.-S. Jia designed the project. J.-S. Jia and J.-R Luo completed the experimental works. co-wrote the manuscript and contributed to scientific discussion.
